# Identification of Potential Therapeutic Targets for *Burkholderia cenocepacia* by Comparative Transcriptomics

**DOI:** 10.1371/journal.pone.0008724

**Published:** 2010-01-15

**Authors:** Deborah R. Yoder-Himes, Konstantinos T. Konstantinidis, James M. Tiedje

**Affiliations:** 1 Center for Microbial Ecology, Michigan State University, East Lansing, Michigan, United States of America; 2 School of Civil and Environmental Engineering and School of Biology, Georgia Institute of Technology, Atlanta, Georgia, United States of America; National Institutes of Health, United States of America

## Abstract

**Background:**

*Burkholderia cenocepacia* is an endemic soil dweller and emerging opportunistic pathogen in patients with cystic fibrosis (CF). The identification of virulence factors and potential therapeutic targets has been hampered by the genomic diversity within the species as many factors are not shared among the pathogenic members of the species.

**Methodology/Principal Findings:**

In this study, global identification of putative virulence factors was performed by analyzing the transcriptome of two related strains of *B. cenocepacia* (one clinical, one environmental) under conditions mimicking cystic fibrosis sputum versus soil. Soil is a natural reservoir for this species; hence, genes induced under CF conditions relative to soil may represent adaptations that have occurred in clinical strains. Under CF conditions, several genes encoding proteins thought to be involved in virulence were induced and many new ones were identified. Our analysis, in combination with previous studies, reveals 458 strain-specific genes, 126 clinical-isolate-specific, and at least four species-specific genes that are induced under CF conditions. The chromosomal distribution of the induced genes was disproportionate to the size of the chromosome as genes expressed under soil conditions by both strains were more frequent on the second chromosome and those differentially regulated between strains were more frequent on the third chromosome. Conservation of these induced genes was established using the 11 available Bcc genome sequences to indicate whether potential therapeutic targets would be species-wide.

**Conclusions/Significance:**

Comparative transcriptomics is a useful way to identify new potential virulence factors and therapeutic targets for pathogenic bacteria. We identified eight genes induced under CF conditions that were also conserved in the Bcc and may constitute particularly attractive therapeutic targets due to their signal sequence, predicted cellular location, and homology to known therapeutic targets.

## Introduction

The *Burkholderia cepacia* complex (Bcc) is comprised of at least 17 related species [1] that have been isolated from a broad array of environments including soil, plant rhizospheres, freshwaters, sterile solutions, cosmetics, and plastics [2]. Members of this complex were previously used for biocontrol of plant root disease as they can substitute for commercial fungicides [3]. In addition, members of this complex can be isolated from the lungs of cystic fibrosis patients [CF, the most common genetic disorder in Caucasian populations], chronic granulomatis disease, and other immunocompromised individuals. Bcc–infected CF patients show a median life expectancy decrease of 15 years compared to the general CF population [4] and Bcc infection is currently a contraindication to lung transplant in several CF clinics due to poor post-transplantation outcomes [5].

The Bcc have large, metabolically diverse, and plastic genomes which may explain their ability to live in such diverse environments. Eighteen genomes representing six Bcc species have been (or are being) sequenced [6]. Of these, five genomes of *B. cenocepacia* have been completed: J2315, AU1054, PC-184 (representing the three major epidemic lineages), HI2424 (a soil isolate), and MC0-3 (a rhizosphere isolate). Their 6.90–8.06 Mbp multireplicon genomes have an Average Nucleotide Identity in their conserved genes [ANI, [7]] ranging from 94–99.8%, revealing considerable genetic breadth within the species. Of the Bcc, *B. cenocepacia* has been the most studied at the molecular level due to its clinical importance.

Studies on the virulence mechanisms of the *B. cenocepacia* have revealed a number of potential or contributing virulence factors including the CepI/R quorum-sensing system [8], [9], a cable pilus and an adhesin [10], [11], [12], flagella [13], [14], [15], [16], [17], [18], siderophores [19], [20], a hemolysin [21], ZmpA and ZmpB proteases [22], [23], exo- and lipopolysaccharides [24], [25], [26], and the extracellular capsule [26]. Additionally, the *Burkholderia* epidemic strain marker (BCESM) is part of the *cci* pathogenicity island and may be involved in person-to-person transmission of epidemic strains [27], [28]. However, many studies have been performed in assay systems that may not well represent the human lung and many genetic markers are not universally conserved among Bcc strains thought to be pathogenic and/or are frequently found in environmental (soil) strains. Thus, a definitive understanding of Bcc virulence remains elusive. Additionally, Bcc species are highly antibiotic resistant and there is currently no vaccine against this group of organisms necessitating new strategies for control of the disease. Two previous studies have identified several hundred genes expressed under conditions mimicking CF sputum though these studies used different strains and conditions, making them difficult to compare [29], [30]. Thus, further studies that identify species- or complex-wide expression patterns are needed in order to identify putative targets for therapeutics that can be used to treat many Bcc infections.

To identify factors at the whole-genome level that may be involved in human virulence, we analyzed the transcriptome of an epidemic strain of *B. cenocepacia*, J2315, under conditions that mimic CF sputum. Strain J2315, the best-studied epidemic isolate, lies within the ET12 lineage of *B. cenocepacia*, and contains genes for the cable pilus and the BCESM [31]. We compared the J2315 CF-induced transcriptome to (i) the transcriptome of J2315 grown in a medium that mimics the soil to identify genes preferentially expressed under CF conditions, and to (ii) the CF-induced transcriptome of a *B. cenocepacia* soil isolate, HI2424 [32], to identify genes that may have been recently acquired or adapted to the human lung. Strain HI2424 bears a striking genetic resemblance to an epidemic strain, AU1054 (99.8% ANI, and 50% of its genome identical at the nucleotide level), from the PHDC lineage, but is a soil isolate that, like AU1054, lacks the genes encoding the cable pilus and BCESM. J2315 and HI2424 are moderately related, sharing 96.5% ANI and over 75% of their genes, but possessing a number of changes in the coding regions of the shared genes. Differences in gene regulation between the latter strains have not been examined, even though these strains are good candidates for a global approach for virulence factor identification. Furthermore, we also compared our transcriptomic profiles to previously published transcriptomic data to define a smaller set of targets for further investigations to better understand *B. cenocepacia* pathogenicity and identify potential therapeutic targets.

## Materials and Methods

### Strains and Growth Conditions

*B. cenocepacia* J2315 and HI2424 were grown in quadruplicate 5 ml cultures of SCFM (CF) medium [33] at 37°C overnight with shaking. This medium has been shown to elicit the same transcriptomic response at many genetic loci as actual CF sputum in *Pseudomonas aeruginosa*, another CF pathogen. Thirty-ml cultures were inoculated 1∶100 with the overnight cultures and incubated with aeration until the optical density at 600 nm (O.D._600)_ reached 1.0. *B. cenocepacia* J2315 was grown in 45 ml cultures of 10% tryptic soy broth overnight at 37°C. Cells were centrifuged for 10 min at 4500×*g* and 3 ml of spent supernatant was used to resuspend the cells. Thirty-ml of a modified soil extract (SE) medium [34] containing 3 mM glucose and 10% soil extract (prepared by autoclaving maize rhizosphere soil) was inoculated 1∶30 with the overnight culture and incubated at 37°C until the O.D._600_ reached 0.8. Cells from all conditions were chilled on ice and collected by centrifugation at 4°C for further processing.

### RNA Purification and Preparation for Microarrays

Total RNA was purified by the RiboPure Kit (Ambion, Austin, TX) with the following exceptions: 2×2 mL of fresh culture was used for each biological replicate; cells were cup-sonicated 3×12 seconds (90% duty cycle, 70% output cycle, W-385 sonicator, Misonix, Inc, Farmingdale, NY) on ice after resuspension in RNAwiz reagent to encourage lysis; a 1-h incubation of the samples with DNase I was used. RNA quality and concentration was measured using Agilent's RNA 6000 Pico kit and 2100 Bioanalyzer machine. Only samples with RNA integrity numbers greater than 8.0 were used for microarray experiments. cDNA generation and labeling was performed using the CyScribe Post-Labeling kit (GE Healthcare) according to the manufacturer's protocol with the following exceptions: Spike-In controls (Agilent) were included in the labeling procedure for quality control purposes; the second dilution of the Spike-In control was added to the primer annealing mix. For primer annealing the following quantities were used: 10 µg total RNA, 2 µl random nonamers, 1 µl anchored oligo(dT), 2 µl Agilent Spike-In control, and water to 11 µl. The cDNA purification was performed by ethanol precipitation and the labeled cDNA was purified using the CyScribe GFX purification kit. For the elution from the GFX columns, water was used instead of the elution buffer from the kit.

### Microarray Hybridization and Analysis

The Agilent microarrays used in this study have been described previously [30], [35]. Briefly, these 60-mer arrays contain 11K spots corresponding to all predicted coding regions in J2315, AU1054, and HI2424. There are also probes corresponding to large intergenic regions from J2315 represented on the array. Hybridization and washing of quadruplicate arrays including a single dye swap was performed according to the “Two-color microarray based gene expression analysis” protocol from Agilent (version 5.5, Feb. 2007) with the following changes: for hybridization the 25x fragmentation buffer was omitted; the mix of cDNAs and 10x Blocking Agent was heat-denatured for 3 min at 98°C and cooled to room temperature before adding the hybridization buffer; the post-hybridization microarray washing included the optional acetonitrile and Stabilization and Drying Solution (Agilent). The microarrays were scanned with the G2565 BA microarray scanner (Agilent) and the Scan Control software version A.7.0.3 Feb 2007 (Agilent). The scanning resolution was set to 5 µm and the scan region was adjusted to 61×21.6 mm. The Extended Dynamic Range function was switched on with 100% and 10% PMT gain settings. The images were analyzed with the Feature Extraction software, version 9.5.1 February 2007 (Agilent). The FE protocol used was GE2_v5_95_Feb07 with the default setting maintained. GeneSpring GX 7.3.1 was used to analyze gene expression data. The data was filtered for flags, then selected based on expression levels greater than 2-fold. The Benjamini-Hochberg False Discovery Rate (FDR) multiple testing correction was applied to all filtered data sets and genes with p-values less than 0.05 were considered significant. Genes that were induced in each comparison are described further in Supplementary Tables S1, S2, S3, S4, and in the downloadable Microsoft Excel File included as Supplementary File S1.

### Conservation of Genes

To determine the presence/absence of homologs of the J2315 genes in the other *Burkholderia* genomes analyzed in this study, the following approach was employed. The J2315 gene sequences were searched against the genomic sequences using the BLASTN algorithm version 2.2.18 (nucleotide level) [36] for matches that provided alignments covering at least 70% of the length of the query sequence (homolog present). The nucleotide identity of the best match for each genome was saved and reported in supplementary Table S5. The BLASTN algorithm was run with the following settings: X = 150 (drop-off value for gapped alignment), q = −1 (penalty for nucleotide mismatch), and F = F (filter for repeated sequences), the rest of the parameters were at default settings. These settings can more robustly detect most, if not all, homologs shared between relatively distantly related genomes (i.e., showing 70–100% ANI) compared to the default settings, which preferentially target very similar sequences [37].

### Chromosomal Distribution Mapping and Testing

For mapping J2315 genes differentially expressed on the J2315 genome, the corresponding gene sequences were searched against the J2315 genome sequence (three chromosomes and one plasmid), using the BLASTN approach described above, to determine all perfect matches (i.e., 100% nucleotide identity, covering 100% of the length of the query sequence) of the query gene. All such perfect matches were considered to contribute to the microarray signal of the gene (i.e., it was not possible to determine the relative contribution of the different identical copies of multi-copied genes) and thus, were included in the reported results. For probes designed against the HI2424 genes, the corresponding HI2424 gene sequence was searched against the J2315 genome, as described above for J2315 genes, to identify the J2315 homolog and map the latter gene on the J2315 genome. The positions of differentially expressed genes was visualized using the GenomeViz software [38]. Chi-squared testing (χ^2^) for gene distribution was performed on all gene lists in Microsoft Excel.

### Signal Peptide Prediction Analysis and Localization Analysis

SignalP v. 3.1 and TatP v. 1.0 signal prediction algorithms [39], [40], [41] were applied to the amino acid sequences of induced genes. Genes with positive D-scores were considered positive for putative signal peptides. The online software pSORTb [42] was used to predict subcellular locales using default settings for Gram negative bacteria.

### Quantitative Real-Time PCR (qRT-PCR)

Twenty-two unlinked genes were chosen for qRT-PCR based on their differential regulation pattern and annotation. Primer sequences are listed in Table S6 in the Supplementary Materials. qRT-PCR reactions were performed as in [29] with the following exceptions: Power SYBR Green RNA-to-Ct 1-Step Kit (Agilent) according to the manufacturer's instructions except 15 µL was used as a final volume for each reaction; 10 ng RNA, 80 ng genomic DNA (positive control), or water (negative control) were used for templates; cycling conditions were: (48°C×30 min)_1 cycle_, (95°C×10 min)_1 cycle_, (95×15 s, 60×1 min)_40 cycles_; the comparative Ct-method was used to determine the fold difference in gene expression between the two conditions.

### Microarray Data Accession

The microarray raw intensity data were deposited in GenBank Gene Expression Omnibus (GEO) database and assigned the accession number GSE15817.

## Results

**Figure 1:**
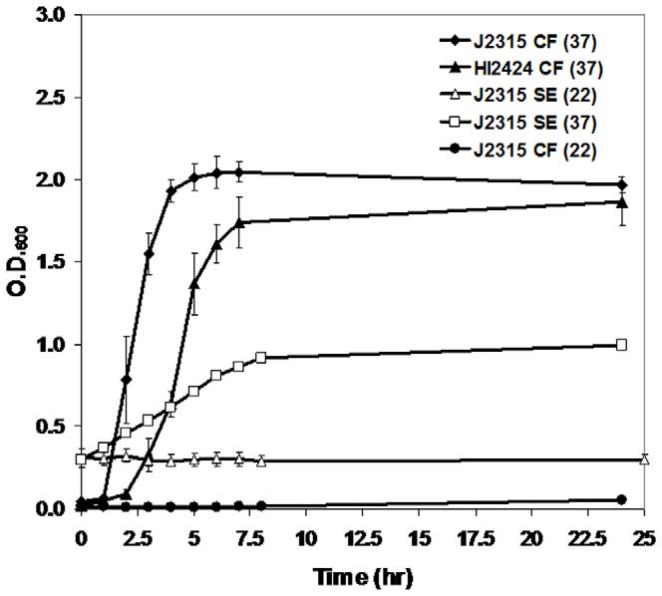
Experimental conditions. Growth, measured by O.D._600_, of strains J2315 and HI2424 under the following conditions: Black diamonds, triangles, circles, and squares represent strain J2315 grown in CF at 37°C, SE at 22°C, CF at 22°C, and SE at 37°C, respectively. Open triangles represent strain HI2424 grown in CF medium at 37°C. Error bars represent a single standard deviation of the data.

Growth of *B. cenocepacia* J2315 and HI2424 under conditions mimicking CF sputum or the soil environment was measured over time to determine the optimal time and cellular density for RNA extraction. Strain J2315 grew significantly faster than HI2424 in CF medium at 37°C (Fig. 1). In contrast, J2315 did not grow well in either medium at 22°C nor did it grow in LB broth at 22°C (Fig. 1), suggesting that J2315 has adapted to the warmer temperature of the human lung. Because J2315 did not grow at a typical soil temperature, it was grown in the soil medium at 37°C. Growth in the more impoverished soil medium was slower than observed for J2315 grown in CF medium, but at least a single doubling was observed which was sufficient for gene expression under the soil conditions.

Competitive hybridizations were performed with cDNA prepared from mid-logarithmic cells in four replicate arrays. Following a global analysis of the pooled data, three gene lists were generated: (i) genes induced in the J2315 versus HI2424 under CF conditions, (ii) genes induced in J2315 CF versus SE conditions, (iii) genes induced in J2315 under CF conditions from both comparisons. The latter should reveal more recent adaptations in J2315 to CF sputum and are discussed separately to avoid redundancy.

### Genes Induced in J2315 under CF Versus SE Conditions

**Figure 2:**
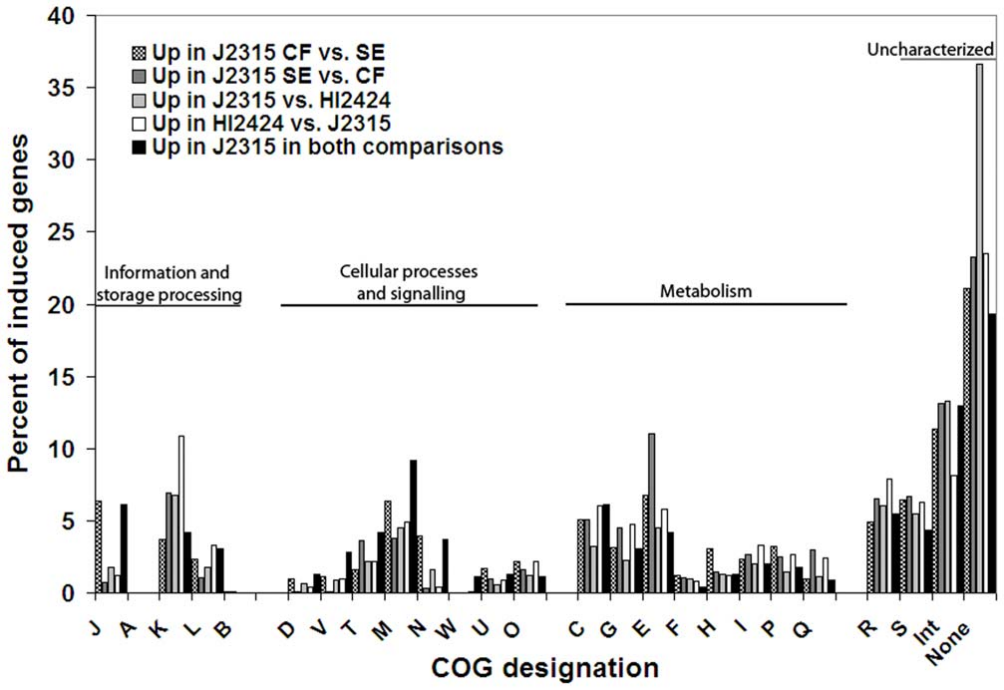
Classification of induced genes by COG functional categories. The percentages of each gene list that encodes proteins belonging to a given COG category are listed for each COG (Table S7 contains key). Checkered bars indicate those genes induced in J2315 under CF conditions in both comparisons (i.e. CF vs. SE conditions, J2315 vs. HI2424 under CF conditions). All raw data are provided in supplementary Tables S2, S3, S4, S5, S6.

Our comparisons indicate that genes encoding proteins involved in translation, and intracellular trafficking show stronger expression under CF conditions (Fig. 2). Genes showing increased expression under CF conditions include those encoding a putative hemolysin, iron transport and ornibactin synthesis, chaperones, type III secretion, and chemotaxis genes (Table S1). Many more genes were found to be differentially expressed and these are provided in Table S1.

**Figure 3:**
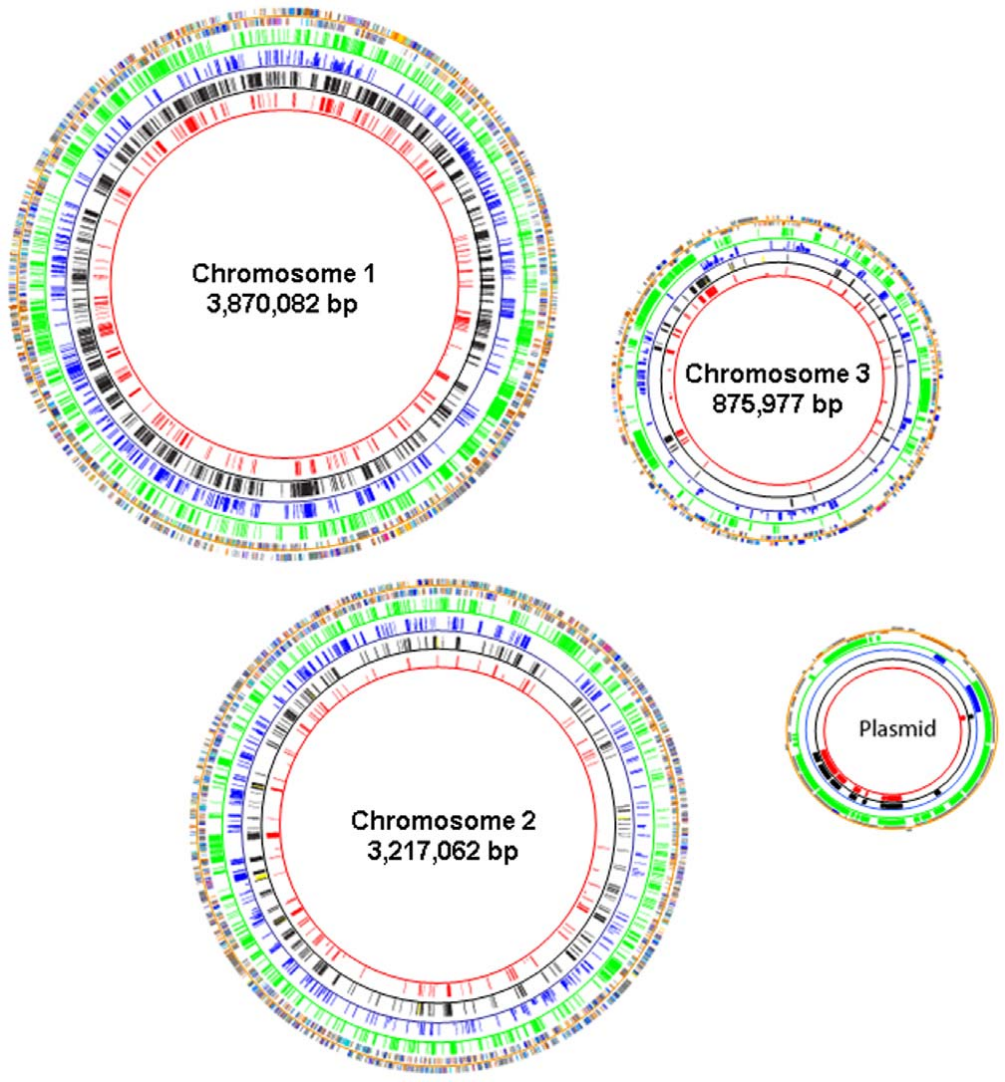
Chromosomal location of induced genes depicted for the three chromosomes and the megaplasmid of J2315. The outermost ring indicates the coding regions colored according to COG functional category. The rings denote (inwards): i) all predicted open reading frames in *B. dolosa* colored by their COG classification (Table S7), ii) the genes induced in J2315 in the comparison with HI2424 under CF conditions (green), iii) the J2315 homologs of the HI2424 genes induced under CF conditions, iv) genes induced in J2315 under CF conditions compared to SE conditions (black), v) the chromosomal location of genes induced in J2315 under CF conditions in both microarray comparisons (red). Genes induced under conditions are further described and classified in Supplementary Tables S1, S2, S3, S4.

Proteins encoding uncharacterized proteins or those involved in signal transduction, carbohydrate and amino acid metabolism are induced under SE conditions (Fig. 3, Table S2). These include proteins involved in putrescine transport, polysaccharide biosynthesis, coenzyme PQQ system, iron transport, nitrogen metabolism, sigma factors, the twin arginine transport (Tat) secretion pathway and many with uncharacterized functions (Table S2).

### Genes Induced in J2315 Versus HI2424 under CF Conditions

Genes that are induced under CF conditions in J2315 may represent genes with changes in regulation or those acquired since the strains diverged. In total, 1833 and 1334 probes, corresponding to 1490 and 1223 annotated genes, were uniquely induced in J2315 and HI2424, respectively. Genes encoding proteins involved in transcription or uncharacterized functions were more induced in J2315 and those involved in inorganic ion metabolism and secondary metabolite metabolism were more strongly induced in HI2424 though the functional profiles of induced genes was quite similar between the strains suggesting that these two strains may utilize different genes to achieve the same tasks (Fig. 2). A large number of the uncharacterized genes induced in J2315 are associated with intergenic regions (Fig. 3). The latter may indicate that there are many either non-coding RNAs or unannotated genes in the J2315 genome that encode mRNAs.

**Figure 4:**
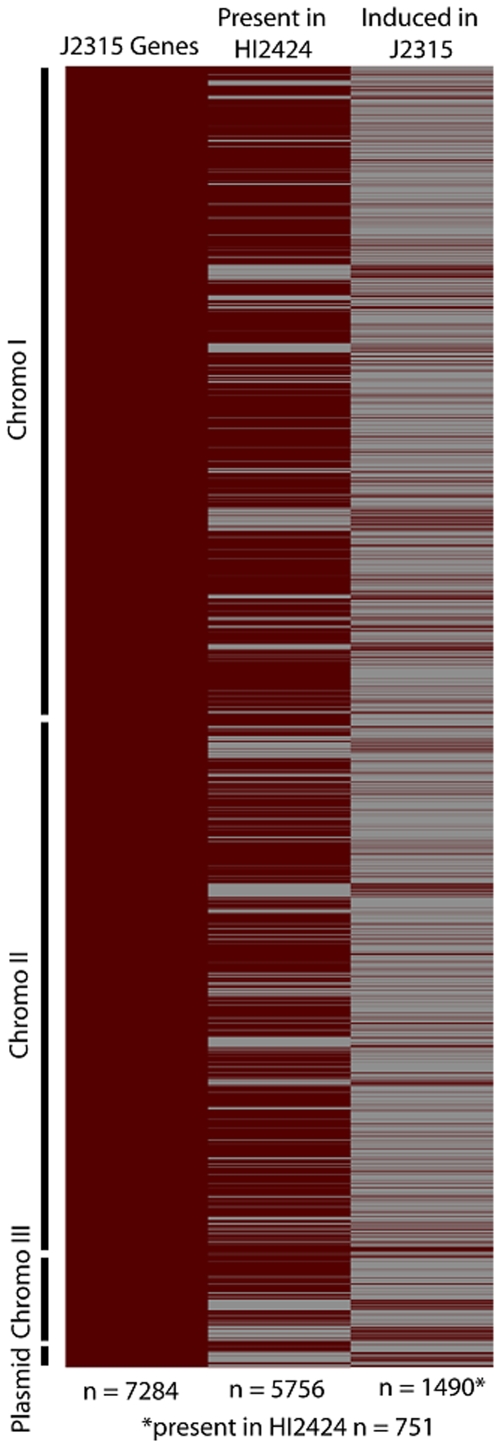
Distribution of J2315 genes, HI2424 homologous genes, and genes induced in J2315 compared to HI2424. Heat map analysis reveals gene clusters induced in J2315 compared to HI2424 under CF conditions that are shared (shown in the middle lane) or unique to J2315. Open reading frames in HI2424 bearing at least 70% identity over 70% of the length were considered homologous to J2315 genes. Red color indicates present/induced and gray indicates absent/not induced.

An examination of the 1490 protein-encoding genes induced over 2-fold in J2315 revealed that 751 of them were present in the HI2424 genome suggesting that the regulation of these “core” genes had changed since the two strains diverged (Fig. 4). The other 739 genes were not found in the HI2424 genome and these genes were typically found in small clusters, i.e, genomic islands, on all three chromosomes (Fig. 4).These 739 genes may represent recent acquisitions by J2315 since over 50% of these genes (430) were annotated as hypothetical proteins (Table S3). Also among these genes were 21 genes corresponding to phage structural genes and more than 30 transposon- and integrase-associated genes, features consistent with genes frequently transferred horizontally among bacteria.

We further investigated the genes uniquely induced in J2315 compared to HI2424 revealed known virulence factors, including the *cciI/R* and *cepI/R* quorum sensing systems, the BCESM gene, genes encoding fimbriae, the AidA adhesin, and the ZmpA metalloprotease (Table S4). Finally, several members of the general secretory (Sec) pathway were highly induced, which is consistent with previous observations from an epidemic strain from the PHDC lineage, AU1054, under this condition [29].

Genes induced in HI2424 under CF conditions included those encoding iron metabolism proteins, chemotaxis proteins, and the ZmpB protease (Table S4). Interestingly, genes encoding proteins in the phenylacetic acid pathway, which were recently described to be important for *B. cenocepacia* virulence in *C. elegans* [43], were also uniquely induced in HI2424.

### Genes Induced in J2315 under CF Conditions Versus SE Conditions and Versus HI2424

From the microarray data sets, 458 genes were induced in both comparisons in J2315 under CF conditions (Table S5). These include genes reported to be important for virulence in previous studies including those encoding biosynthesis of flagellar proteins, capsular polysaccharide, the cable pili, as well as chemotaxis conjugative genes and a host of less well-characterized proteins.

We examined the conservation the 458 genes induced from both comparisons in all sequenced members of the Bcc, a closely related *B. xenovorans* strain, LB400, and in the distantly related Gram-negative CF pathogen, *Pseudomonas aeruginosa*. As expected, genes involved in basal cellular functions such as energy production, translation, amino acid and coenzyme metabolism were conserved in most *Burkholderia* species (Table S5). Additionally, we observed that genes encoding proteins involved in flagella biosynthesis, chemotaxis, or environmental resistance (e.g. multidrug efflux) were also highly conserved in *Burkholderia*. In contrast, intergenic regions and genes encoding proteins involved in transcription (specifically transcription factors) and those annotated as hypothetical were not well conserved. Many of theses genes were unique to J2315. Genes found solely in J2315 or other *B. cenocepacia* strains included proteins involved in capsule biosynthesis (found primarily in *B. cenocepacia* strains only), cable pilus biosynthesis and conjugative processes (unique to *B. cenocepacia* J2315).

As indicated above, two different protein export mechanisms are induced in the two conditions of the study, the Sec system and Tat systems. In both mechanisms, protein export from the cytoplasm occurs when translated proteins containing sequence-specific signal sequences are delivered to the secretion machinery located in the cytoplasmic membrane. To determine whether the induced proteins could be potentially exported by each system, we identified differentially regulated genes predicted to contain signal peptides for either the Sec system (for genes induced under CF conditions) or Tat system (for genes induced under SE conditions). In the J2315 versus HI2424 comparison, 259 (14%) of J2315 induced genes and 255 (19%) of HI2424 genes contain Sec signal sequences (Table S5). Similarly, 242 (17%) of the J2315 induced genes from the CF versus SE comparison contained predicted Sec signal sequences. Many of these genes were annotated to be involved in efflux, transport, iron uptake, signal transduction via methyl-accepting chemotaxis proteins, flagella, pilus, and fimbriae biosynthesis, or have an unknown function. In contrast, only 66 (6%) of the genes up-regulated in J2315 under SE conditions contained a predicted Tat signal sequence (Table S5). Fourteen (21%) of these genes encode proteins of general function prediction only and an additional 8 genes encode proteins of unknown functions. These findings underscore how little we know about the targets of the Tat export pathway. Other than uncharacterized proteins, genes encoding putative short chain dehydrogenases and periplasmic binding proteins were also among the most prevalent functions for proteins with predicted Tat signal sequences. In general, the predicted Sec and Tat signal peptide-containing genes were proportionately distributed among the chromosomes indicating that changes in regulation of secreted proteins has not occurred in a chromosome-dependent manner.

In addition to predicting the presence of signal peptides, we also analyzed the 384 genes encoding proteins for putative protein localization. While many of the proteins (151) could not be accurately predicted, 118, 89, or 9 putative proteins were predicted to lie in the cytoplasm, cytoplasmic membrane, or periplasm respectively (Table S5). Of the remaining proteins, 11 were predicted to lie in the outer membrane and only 6 were predicted to be extracellular.

### Global Distribution of Differentially Expressed Genes

We examined the chromosomal location of differentially expressed genes and found a significantly biased distribution (based on χ^2^ test) compared to the number of genes present on each chromosome (Fig. 3, Table S8). In the J2315 CF versus SE comparison, the number of genes expressed under CF conditions was significantly greater than expected on chromosome 1 and lower than expected for the other replicons (p-values<1e^14^). Conversely, the number of genes induced under SE conditions is significantly greater than expected for chromosome 2 (p-value<0.01). These results suggest that chromosome 2 may provide some functions necessary for soil survival. In the J2315 versus HI2424 comparison, differentially regulated genes were significantly greater than expected for chromosome 3 (p-values<0.001) (Fig. 3, Table S8) which suggests that many of the gene content and regulatory changes since the strains diverged have occurred on chromosome 3.

### Verification of Microarray Data

**Figure 5:**
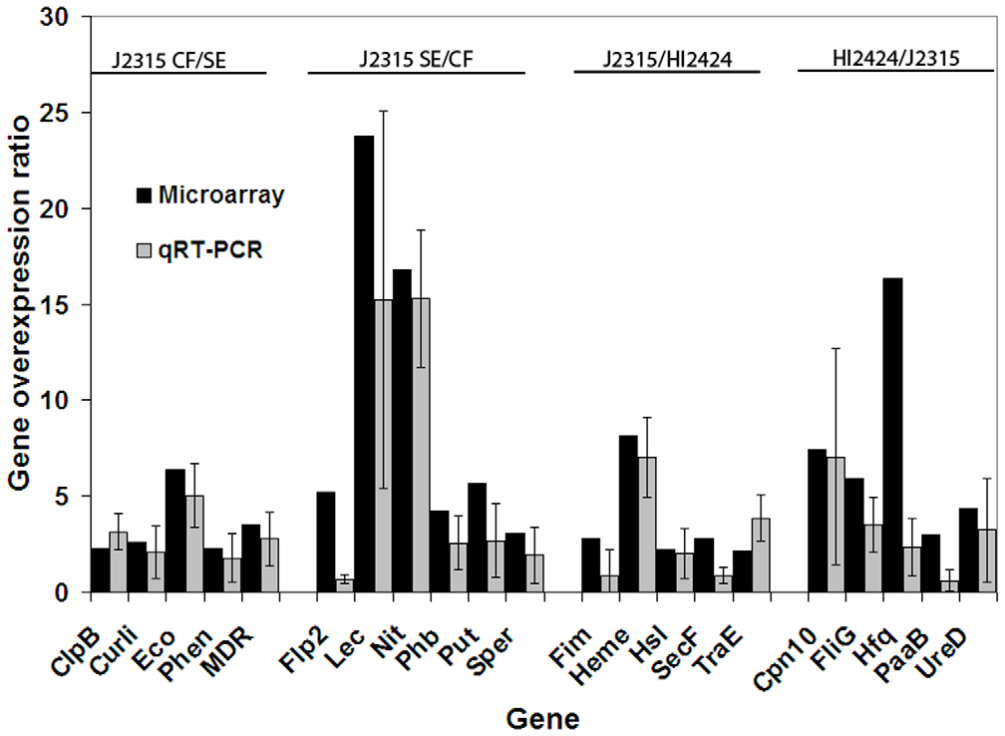
Quantitative real-time PCR verification of microarray data. Twenty-two genes were tested for gene expression ratios and plotted as ratios (either J2315 CF/SE or J2315/HI2424). Error bars correspond to a single standard deviation of the data.

To verify the overexpression ratios observed in the microarray data, qRT-PCR was performed on 21 unlinked chromosomal genes. The genes were chosen based on their putative functions and overexpression patterns in J2315 under CF conditions and include, ClpB protease-associated ATPase, curli production protein, ecotin biosynthesis protein, a multidrug resistance transport protein, phenazine biosynthesis protein; type-1 fimbrial protein, exported heme utilization protein, N-acylhomoserine lactone synthase, general secretory pathway protein F, TraE conjugative transfer protein. Additionally, genes induced in HI2424 (CheA signal transduction histidine kinase, chaperonin Cpn10, flagellar motor switch protein, host factor Hfq, phenylacetate degradation enoyl-CoA hydratase, and urease accessory protein D) or in J2315 under SE conditions (a flp pilus subunit, a lectin, nitrite reductase, polyhydroxybutyrate depolymerase, putrescine permease, spermidine synthase) were also analyzed. Overexpression ratios were statistically consistent with microarray ratios for the 13 of the 20 genes tested (Fig. 5). In the remaining 7 genes, the overall trend of gene expression was similar for both microarray and qRT-PCR ratios but the extent of induction differed up to 5-fold. These results indicate that the microarray data reflects the transcript ratios for the majority of genes.

### Comparison to Previous Transcriptomic Studies

**Figure 6:**
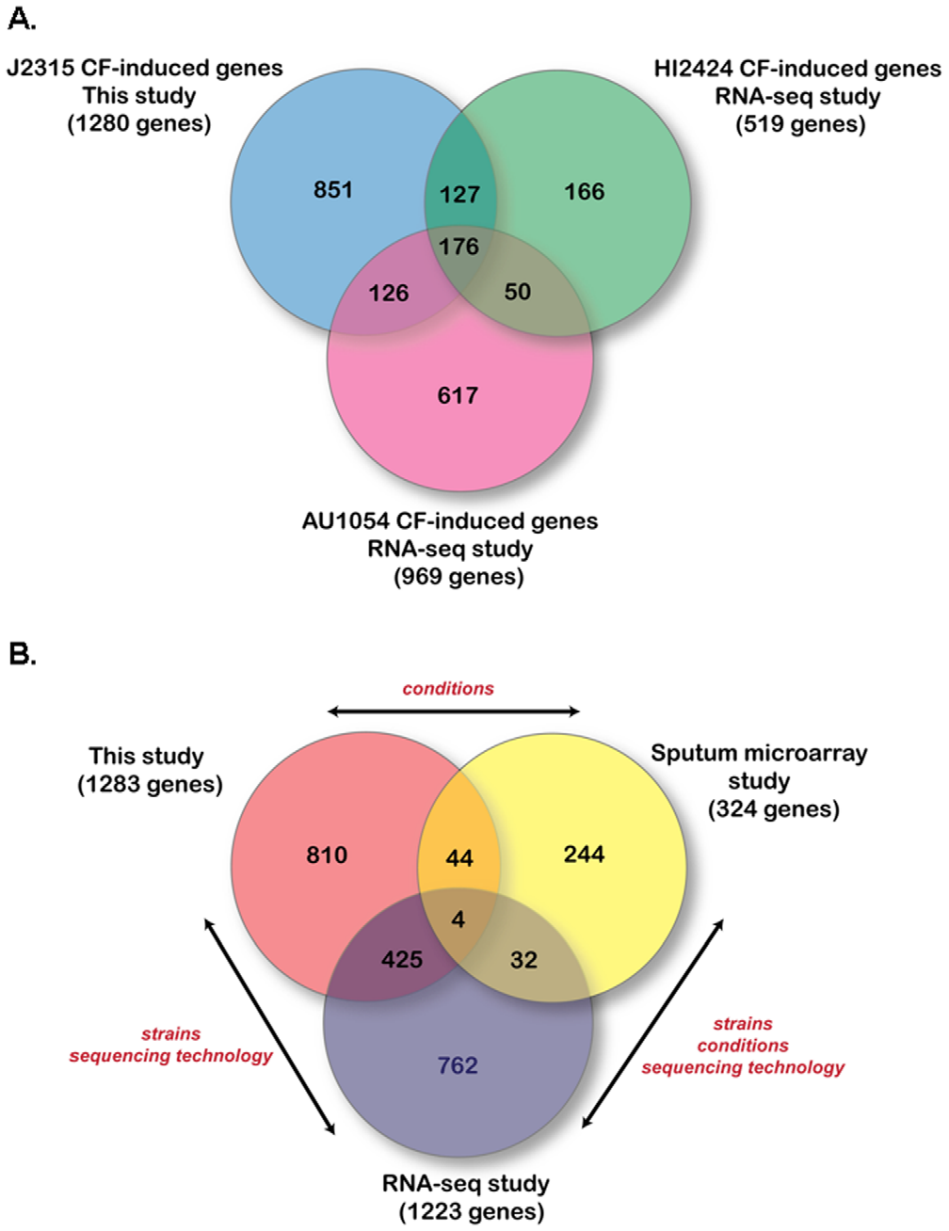
Comparison of microarray data to previous studies. (A) Genes showing at least a 2-fold ratio under CF-like conditions for all three *B. cenocepacia* strains used in this and a previous RNA-seq study [29] compared to minimal growth conditions (i.e. SE conditions at either 22 or 37°C). (B) Genes induced in *B. cenocepacia* strains under CF-like conditions in RNA-seq study [29] and microarray study using different media [29], were collected and compared to the microarray data from this study. The experimental differences between the studies are shown in red text. Note that intergenic regions and genes not found in J2315 were removed from analysis.

A previous study examined the transcriptomic response via Illumina sequencing of cDNA (RNA-seq) from two closely related *B. cenocepacia* strains, AU1054 (an epidemic strain from CF patients) and HI2424, under the same conditions as this study [29]. Because the statistical criteria used for identification of CF-induced genes differed in the previous study, we re-analyzed the RNA-seq data using the multiple testing correction used in the present study and identified 1222 genes that were up-regulated in HI2424 and AU1054 under CF conditions compared to SE conditions. We identified 126 genes induced in the two clinical strains (AU1054 and J2315) and not in the environmental isolate (HI2424) (Fig. 6A, Table S9) which may represent virulence factor candidates. These 126 genes encode proteins with a variety of functions in cellular metabolism including a number of proteins involved in chemotaxis, flagella biosynthesis, and peptidoglycan biosynthesis, suggesting the importance of motility in these clinical isolates. In parallel, we compared these two data sets to previously published microarray-based transcriptomic data from J2315 grown in a dilute CF sputum medium compared to a minimal medium [30]. Only four genes were induced in all three studies (Fig. 6B); these encode ATP synthase gamma subunit, RNA polymerase beta subunit, nucleoside diphosphate kinase, and a putative sulfate transporter (Table S3); the former three are all involved in basic cellular metabolism during growth. The small number of genes commonly induced in these studies could be due to (i) different strains, (ii) different platforms for data analysis, or (iii) different conditions for growth (Fig. 6B). Surprisingly, the latter seems to be more important as the RNA-seq data and the microarray data presented in this study show a greater number of conserved genes expressed compared to the two previous microarray studies and further suggests that RNA-seq and microarray data are quite consistent.

## Discussion

Identifying new Bcc therapeutic targets is of high importance for the cystic fibrosis community. Because of the large number of sequenced Bcc genomes, comparative genomics can help identify potential vaccine targets as has been done with several human pathogens (reviewed in [44]). However, such sequence-based approaches cannot predict those genes that will be expressed in the lung. Our broad transcriptomic approach builds on previous studies and provides a more refined list of potential therapeutic targets for *B. cenocepacia*, one of the most common Bcc CF pathogens. While antimicrobial targets are harder to identify due to the requirement of being essential for growth, potential vaccine targets are easier to identify. Characteristics of good vaccine targets include: (i) expression during infection, (ii) localization to the outer membrane or extracellular milieu, (iii) specificity to many members of the target group (i.e. gene conservation within the group only) and not against beneficial bacteria, and (iv) the ability to trigger a sufficient immunologic response in the host to kill the bacteria. In this study, we identified 17 potential vaccine targets of the 458 candidate genes that are induced in J2315 under infection-like conditions and that are predicted to be localized in the outer membrane or the extracellular milieu. Of these, 8 genes (BCAL0894, BCAL1881, BCAL2082, BCAL2083, BCAL2834, BCAL3279, BCAM1419, BCAM1744) are conserved in all Bcc members but do not have significant similarities to proteins in *P. aeruginosa* PAO1 (Table S6). While it remains speculative whether or not these genes have a role in virulence, their homology to known proteins reveals some interesting candidates. BCAL0894 encodes a homolog of the organic solvent tolerance protein OstA which has been shown to be involved in glutaraldehyde resistance and membrane permeability in *Helicobacter pylori*, a gastroenteric pathogen, although it is not essential for growth in this organism [45]. OstA is also involved in lipopolysaccharide insertion into the outer membrane. While this protein has not been shown to elicit an immunogenic response in humans, it has been included in patented strains of vesicle vaccine-producing *Neisseria meningitis* as this protein promotes antigen production [46]. BCAL1881 encodes a pyrroloquinoline quinone-containing lipoprotein homologous to YfgL found in a variety of bacterial pathogens. *E. coli* YfgL is a lipoprotein involved in outer membrane protein assembly and contributes to epithelial cell invasion *in vitro* [46]. The *Proteus mirabilis* YfgL homolog is immunogenic in a mouse model [47]; thus, the protein encoded by BCAL1881 may play a role in Bcc virulence and may be immunogenic. BCAL2082 encodes a putative chaperone protein Skp precursor. Skp is thought to escort proteins secreted via the Sec pathway, which is known to export toxins and other virulence factors in a broad array of pathogens, to outer membrane porins [48] although localization of this protein is somewhat controversial. Additionally, in pathogenic *E. coli*, Skp is important for survival in an *in vivo* mouse model [49]. BCAL2082 is found in the same operon with another gene on this list, BCAL2083, which encodes a protein, YaeT, which is involved in outer membrane protein assembly. YaeT, an essential protein in *E. coli, Neisseria meningitis*, and *P. aeruginosa* [50], [51], [52], is orthologous to the protective surface antigen D15 which was originally identified in *Haemophilus influenzae*. D15 has been shown to be highly immunogenic in humans, mice, guinea pigs, and rabbits [53], [54] and is currently being studied for inclusion in a vaccine against non-typeable *H. influenzae* [55]. The ortholog of this protein in *B. cenocepacia* HI2424 was shown to be induced under CF-like conditions compared to soil conditions in the RNA-seq study [29]. Because these two genes are found in the same operon, perhaps BCAL2082 is required for BCAL2083 surface presentation. BCAL2834 encodes an acylhydrolase with similarities to phospholipases in other pathogens. It bears some similarity to McaP from *Moraxella catarrhalis*, which is involved in adherence to human epithelial cells [56]. McaP has also been considered as a potential vaccine target [56] although its immunogenic potential in animal models or humans is not clear. The remaining three proteins, BCAL3279, BCAM1419, and BCAM1744, have unknown virulence and immunogenic potential. BCAL3279 encodes a putative membrane protein with unknown function. It is similar to conserved hypothetical proteins in several β- and γ-Proteobacteria including *Ralstonia*, *Vibrio*, and enteric species. BCAM1419 encodes the outer membrane component of a RND-type drug efflux system which can efflux a broad array of antimicrobials and chemicals in several pathogenic bacteria (reviewed in [57]). BCAM1744 encodes a putative serine metallopeptidase similar to extracellular subtilisins. Subtilisin from *Mycobacterium tuberculosis* has been shown to be induced in macrophages [58].

The distribution of CF induced genes was inversely proportional to the number of genes present on the three chromosomes suggesting that the three chromosomes play a different role during adaptation (possibly due to selective pressures) to these environments. The significantly larger number of induced genes in the smaller chromosomes is consistent with previous studies of additional *B. cenocepacia* strains comparing these two media [29] and suggest that the adaptations of the strains since their divergence from each other occurred preferentially in the smaller replicons and that the three replicons of *B. cenocepacia* may play disparate roles in different environments. Bacteria harboring multiple replicons inside and outside the *Burkholderia* genus have also been shown to have a distributional bias in their conserved or induced genes. *B. xenovorans*, a well-known pollutant-degrading environmental *Burkholderia* species, shows the greatest gene conservation on the largest of its three chromosomes [59]. Another example of a distributional bias in the presence of conserved genes is *Agrobacterium* species in which conservation of gene content and order is more conserved on the larger of the two chromosomes [60]. An example of distributional bias of genes induced under a particular condition is *Vibrio cholerae* which, when grown in an *in vivo* mouse intestinal model, shows a greater number of induced genes on the smaller of two replicons compared to growth on rich laboratory medium *in vitro* [61]. The origin and function of multiple replicons in bacteria is just beginning to be studied; however, studying these organisms may give insight into the origin of multiple chromosomes in higher organisms.

In summary, we have used the transcriptomic response of *B. cenocepacia* strains to identify genes potentially involved in virulence or genes that are putative candidates for vaccine or antimicrobial therapies. Interestingly, although these two strains belong to the same species (and share 96.5% ANI), we saw the expression of thousands of genes to be over 2-fold different, indicating strong ecological specialization/adaptation of the two strains. Members of the Bcc are notoriously difficult for physicians to treat due to their extensive antimicrobial resistance. Thus new strategies must be identified to combat and prevent forthcoming infections. Future studies will focus on elucidating the role of these proteins during infection in *in vitro* and *in vivo* models.

## Supporting Information

File S1 Searchable Spreadsheet of the data. Excel spreadsheet that allows easy, searchable access of the differentially regulated genes for all comparisons to the reader. This needs to be maintained as an Excel file, not a PDF.
(1.09 MB XLS)

Table S1 Genes induced in J2315 under CF conditions compared to SE condition.
(2.90 MB DOC)

Table S2 Probes showing at least a 2-fold increase in J2315 under SE conditions versus CF conditions. List of genes induced in the clinical isolate J2315 under soil-like conditions compared to CF-like conditions.
(2.07 MB DOC)

Table S3 Probes showing at least a 2-fold greater pixel intensity in J2315 versus HI2424 under CF conditions. List of genes induced in the clinical isolate J2315 compared to the soil isolate HI2424 under CF-like conditions.
(3.36 MB DOC)

Table S4 Probes showing at least a 2-fold increase in HI2424 versus J2315 under CF conditions. List of genes induced in the soil isolate HI2424 compared to the clinical isolate J2315 under CF-like conditions.
(2.66 MB DOC)

Table S5 Conservation of genes/regions overexpressed in J2315 under CF conditions in both microarray comparisons. List of 458 genes uniquely induced in the clinical epidemic isolate J2315 under CF-like conditions compared to both itself under soil-like conditions and to the soil isolate HI2424 under CF-like conditions.
(1.15 MB DOC)

Table S6 Primers used for quantitative real-time PCR in this study. Primer sequences for each gene used for quantitative real-time PCR in this study.
(0.10 MB DOC)

Table S7 COG functional classifications and colors for Figures 2 and 3. Color indications for each COG functional classification shown in Figures 2 and 3.
(0.05 MB DOC)

Table S8 Chi-squared test for differentially regulation based on chromosomal location. Statistical calculations for determining the significance of the number of genes differentially regulated by chromosomal location.
(0.07 MB DOC)

Table S9 Genes induced in clinical isolates J2315 and AU1054 and not in the soil isolate HI2424. List of 126 genes induced in the clinical epidemic isolates J2315 and AU1054 and not in the soil isolate HI2424.
(0.13 MB DOC)
