# Supplementary material for: Identification of Potential Therapeutic Targets for Burkholderia cenocepacia by Comparative Transcriptomics
Source: PLoS One. 2010 Jan 15;5(1):e8724. doi: 10.1371/journal.pone.0008724 (PMC2806911; doi:10.1371/journal.pone.0008724)
Supplement: Table S1 — Genes induced in J2315 under CF conditions compared to SE condition. (2.90 MB DOC) [file pone.0008724.s002.doc]

Table S1. Probes showing greater than 2-fold expression for J2315 under CF conditions versus SE conditions.

| **Probe ID** | **J2315 CF/SE expression ratio** | **p-value** | **J2315 Gene** | **Annotation*** | **HI2424 homolog** |
| --- | --- | --- | --- | --- | --- |
|  |  |  |  | ***Translation, ribosomal structure and biogenesis (J)*** |  |
| BCAL0115 | 5.62 | 9E-05 | BCAL0115 | 30S ribosomal protein S21 1 | Bcen2424_0242 |
| BCAL0219 | 3.615 | 6E-04 | BCAL0219 | elongation factor Tu | No homolog |
| BCAL0222 | 4.747 | 6E-11 | BCAL0222 | 50S ribosomal protein L11 | Bcen2424_0336 |
| BCAL0223 | 2.964 | 1E-05 | BCAL0223 | 50S ribosomal protein L1 | Bcen2424_0337 |
| BCAL0224 | 14.54 | 1E-04 | BCAL0224 | 50S ribosomal protein L10 | Bcen2424_0338 |
| BCAL0225 | 11.46 | 2E-05 | BCAL0225 | 50S ribosomal protein L7/L12 | Bcen2424_0339 |
| BCAL0229 | 8.02 | 1E-04 | BCAL0229 | 30S ribosomal protein S12 | Bcen2424_0343 |
| BCAL0230 | 4.887 | 2E-08 | BCAL0230 | 30S ribosomal protein S7 | Bcen2424_0344 |
| BCAL0231 | 5.709 | 9E-07 | BCAL0231 | elongation factor G | Bcen2424_0345 |
| BCAL0232 | 3.457 | 3E-04 | BCAL0232 | elongation factor Tu (EF-Tu) | Bcen2424_0346 |
| BCAL0233 | 5.365 | 3E-05 | BCAL0233 | 30s ribosomal protein S10 | Bcen2424_0347 |
| BCAL0234 | 5.867 | 1E-06 | BCAL0234 | 50S ribosomal protein L3 | Bcen2424_0348 |
| BCAL0235 | 18.19 | 2E-07 | BCAL0235a | 50S ribosomal protein L4 | Bcen2424_0349 |
| BCAL0236 | 15.62 | 2E-06 | BCAL0236 | 50S ribosomal protein L23 | Bcen2424_0350 |
| BCAL0237 | 16.99 | 3E-05 | BCAL0237 | 50S ribosomal protein L2 | Bcen2424_0351 |
| BCAL0238 | 21.34 | 2E-27 | BCAL0238 | 30S ribosomal protein S19 | Bcen2424_0352 |
| BCAL0240 | 14.1 | 1E-05 | BCAL0240 | 30S ribosomal protein S3 | Bcen2424_0354 |
| BCAL0241 | 8.874 | 4E-05 | BCAL0241 | 50S ribosomal protein L16 | Bcen2424_0355 |
| BCAL0242 | 11.42 | 3E-07 | BCAL0242 | 50S ribosomal protein L29 | Bcen2424_0356 |
| BCAL0244 | 3.42 | 3E-06 | BCAL0244 | 50S ribosomal protein L14 | Bcen2424_0358 |
| BCAL0245 | 4.716 | 9E-04 | BCAL0245 | 50S ribosomal protein L24 | Bcen2424_0359 |
| BCAL0246 | 7.349 | 1E-04 | BCAL0246 | 50S ribosomal protein L5 | No homolog |
| BCAL0247 | 7.399 | 5E-05 | BCAL0247 | 30S ribosomal protein S14 | Bcen2424_0361 |
| BCAL0248 | 6.953 | 2E-04 | BCAL0248 | 30S ribosomal protein S8 | Bcen2424_0362 |
| BCAL0249 | 4.247 | 4E-05 | BCAL0249 | 50S ribosomal protein L6 | Bcen2424_0363 |
| BCAL0250 | 6.056 | 7E-09 | BCAL0250 | 50S ribosomal protein L18 | Bcen2424_0364 |
| BCAL0251 | 4.362 | 9E-04 | BCAL0251 | 30S ribosomal protein S5 | Bcen2424_0365 |
| BCAL0252 | 3.743 | 2E-06 | BCAL0252 | 50S ribosomal protein L30 | Bcen2424_0366 |
| BCAL0253 | 3.186 | 8E-07 | BCAL0253 | 50S ribosomal protein L15 | Bcen2424_0367 |
| BCAL0255 | 4.943 | 6E-04 | BCAL0255 | translation initiation factor IF-1 1 | Bcen2424_0369 |
| BCAL0256 | 3.173 | 2E-04 | BCAL0256 | 50S ribosomal protein L36 | Bcen2424_0370 |
| BCAL0257 | 2.817 | 4E-11 | BCAL0257 | 30S ribosomal protein S13 | Bcen2424_0371 |
| BCAL0258 | 3.324 | 2E-03 | BCAL0258 | 30S ribosomal protein S11 | Bcen2424_0372 |
| BCAL0259 | 4.444 | 1E-20 | BCAL0259 | 30S ribosomal protein S4 | Bcen2424_0373 |
| BCAL0261 | 3.358 | 1E-04 | BCAL0261 | 50S ribosomal protein L17 | Bcen2424_0375 |
| BCAL0292 | 3.487 | 4E-04 | BCAL0292 | 2',5' RNA ligase family protein | Bcen2424_0405 |
| BCAL0361 | 2.962 | 8E-04 | BCAL0361 | putative methyltransferase | No homolog |
| BCAL0374 | 2.859 | 1E-04 | BCAL0374 | peptide chain release factor 1 | Bcen2424_0510 |
| BCAL0424 | 3.286 | 6E-04 | BCAL0424 | ribonuclease P protein component | Bcen2424_3165 |
| BCAL0469 | 2.232 | 3E-03 | BCAL0469 | ribosomal RNA small subunit methyltransferase B* | Bcen2424_3123 |
| BCAL0485 | 2.306 | 1E-04 | BCAL0485 | glutamyl-tRNA amidotransferase subunit B | No homolog |
| BCAL0798 | 2.622 | 3E-11 | BCAL0798 | peptidyl-tRNA hydrolase | Bcen2424_2804 |
| BCAL0799 | 3.779 | 7E-07 | BCAL0799 | ribosomal L25p family protein | Bcen2424_2803 |
| BCAL0880 | 4.33 | 2E-04 | BCAL0880 | aspartyl-tRNA synthetase | Bcen2424_2725 |
| BCAL0897 | 2.757 | 2E-09 | BCAL0897 | dimethyladenosine transferase | Bcen2424_2708 |
| BCAL1483 | 2.386 | 3E-03 | BCAL1483 | 50S ribosomal protein L35* | Bcen2424_1477 |
| BCAL1484 | 3.012 | 2E-05 | BCAL1484 | 50S ribosomal protein L20 | Bcen2424_1478 |
| BCAL1485 | 2.348 | 7E-04 | BCAL1485 | phenylalanyl-tRNA synthetase alpha chain | Bcen2424_1479 |
| BCAL1507 | 2.066 | 2E-03 | BCAL1507 | translation initiation factor IF-2 | Bcen2424_1500 |
| BCAL1508 | 2.743 | 1E-05 | BCAL1508 | putative ribosome-binding factor | No homolog |
| BCAL1889 | 2.678 | 4E-06 | BCAL1889 | putative 23S rRNA (uracil-5-)-methyltransferase | Bcen2424_1817 |
| BCAL1905 | 3.148 | 3E-04 | BCAL1905 | 50S ribosomal protein L31 type B | Bcen2424_1833 |
| BCAL1933 | 2.639 | 1E-03 | BCAL1933 | L-arabinose formyltransferase | Bcen2424_1861 |
| BCAL1942 | 2.043 | 1E-03 | BCAL1942 | 50S ribosomal protein L9 | Bcen2424_1871 |
| BCAL1943 | 2.021 | 6E-04 | BCAL1943 | 30S ribosomal protein S18 | Bcen2424_1872 |
| BCAL2076 | 5.299 | 1E-04 | BCAL2076 | putative RNA methylase protein | No homolog |
| BCAL2088 | 2.949 | 1E-03 | BCAL2088 | ribosome recycling factor | Bcen2424_2016 |
| BCAL2090 | 3.928 | 1E-14 | BCAL2090 | elongation factor TS | Bcen2424_2018 |
| BCAL2091 | 4.342 | 3E-14 | BCAL2091 | 30S ribosomal protein S2 | Bcen2424_2019 |
| BCAL2095 | 2.248 | 1E-03 | BCAL2095 | putative deformylase | Bcen2424_2023 |
| BCAL2104 | 2.03 | 2E-03 | BCAL2104 | putative DNA methylase | Bcen2424_2033 |
| BCAL2126 | 2.057 | 1E-03 | BCAL2126 | glutamyl-tRNA synthetase | Bcen2424_2056 |
| BCAL2190 | 2.929 | 5E-12 | BCAL2190 | lysyl-tRNA synthetase | Bcen2424_2118 |
| BCAL2348 | 3.382 | 4E-19 | BCAL2348 | polyribonucleotide nucleotidyltransferase | Bcen2424_2253 |
| BCAL2349 | 2.868 | 1E-03 | BCAL2349 | 30S ribosomal protein S15 | Bcen2424_2254 |
| BCAL2395 | 2.427 | 1E-17 | BCAL2395 | ribonuclease G | Bcen2424_2301 |
| BCAL2447 | 3.001 | 2E-04 | BCAL2447 | endoribonuclease L-PSP family protein | Bcen2424_2352 |
| BCAL2714 | 3.245 | 7E-04 | BCAL2714 | 50S ribosomal protein L28 | Bcen2424_2502 |
| BCAL2715 | 3.798 | 8E-05 | BCAL2715 | 50S ribosomal protein L33 | Bcen2424_2503 |
| BCAL2765 | 3.73 | 6E-05 | BCAL2765 | 30S ribosomal protein S20* | Bcen2424_2551 |
| BCAL2925 | 2.989 | 3E-12 | BCAL2925 | 50S ribosomal protein L19 | Bcen2424_1072 |
| BCAL2926 | 2.641 | 6E-04 | BCAL2926 | tRNA(guanine-N(1)-)-methyltransferase | Bcen2424_1071 |
| BCAL2927 | 3.334 | 2E-09 | BCAL2927 | 16S rRNA processing protein | Bcen2424_1070 |
| BCAL2928 | 4.142 | 1E-06 | BCAL2928 | 30S ribosomal protein S16 | Bcen2424_1069 |
| BCAL3257 | 3.243 | 2E-06 | BCAL3257 | putative tRNA delta(2)-isopentenylpyrophosphate transferase | No homolog |
| BCAL3304 | 2.394 | 1E-09 | BCAL3304 | putative queuine tRNA-ribosyltransferase | Bcen2424_0722 |
| BCAL3334 | 2.459 | 2E-07 | BCAL3334 | putative nitrogen regulation-related protein | Bcen2424_0693 |
| BCAL3343 | 3.716 | 1E-04 | BCAL3343 | D-tyrosyl-tRNA | Bcen2424_0684 |
| BCAL3344 | 2.446 | 8E-04 | BCAL3344 | tyrosyl-tRNA synthetase | Bcen2424_0683 |
| BCAL3347 | 5.657 | 6E-05 | BCAL3347 | 30S ribosomal protein S9 | Bcen2424_0680 |
| BCAL3348 | 3.79 | 3E-04 | BCAL3348 | 50S ribosomal protein L13 | Bcen2424_0679 |
| BCAL3373 | 2.374 | 6E-03 | BCAL3373 | leucyl-tRNA synthetase | Bcen2424_0654 |
| BCAL3377 | 2.226 | 4E-04 | BCAL3377 | putative outer membrane protein* | Bcen2424_0650 |
| BCAL3422 | 3.969 | 1E-05 | BCAL3422 | ribosomal protein L11 methyltransferase | Bcen2424_0603 |
| BCAL3441 | 3.234 | 1E-14 | BCAL3441 | 50S ribosomal protein L27 | Bcen2424_0582 |
| BCAL3442 | 5.133 | 3E-03 | BCAL3442 | 50S ribosomal protein L21 | Bcen2424_0581 |
| BCAM1023 | 3.593 | 3E-04 | BCAM1023 | putative selenocysteine-specific elongation factor | Bcen2424_3989 |
| BCAM1618 | 10.93 | 4E-04 | BCAM1618 | translation initiation factor IF-1 2 | Bcen2424_4463 |
| BCAM1839 | 2.21 | 1E-04 | BCAM1839 | putative endoribonuclease | No homolog |
| BCAM1840 | 3.621 | 3E-10 | BCAM1840 | putative acetyltransferase | No homolog |
| BCAS0245 | 2.13 | 3E-02 | BCAS0245 | 30S ribosomal protein S21 3 | No homolog |
| BCAS0468 | 2.376 | 2E-06 | BCAS0468 | putative acetyltransferase-GNAT family | Bcen2424_6553 |
| IG1_3766524 | 3.548 | 6E-04 | BCAL3442 | 50S ribosomal protein L21 | Bcen2424_0581 |
|  |  |  |  | ***Transcription (K)*** |  |
| BCAL0144 | 5.31 | 4E-04 | BCAL0144 | RNA polymerase sigma factor for flagellar regulon FliA | Bcen2424_0271 |
| BCAL0210 | 2.801 | 9E-06 | BCAL0210 | TetR family regulatory protein | No homolog |
| BCAL0221 | 8.715 | 2E-04 | BCAL0221 | transcription antitermination protein NusG | No homolog |
| BCAL0226 | 2.057 | 1E-04 | BCAL0226 | DNA-directed RNA polymerase beta chain | Bcen2424_0340 |
| BCAL0260 | 6.978 | 2E-07 | BCAL0260 | DNA-directed RNA polymerase alpha chain | Bcen2424_0374 |
| BCAL0309 | 2.927 | 5E-05 | BCAL0309 | BolA-like protein | Bcen2424_0422 |
| BCAL0494 | 2.365 | 4E-04 | BCAL0494 | TetR family regulatory protein | Bcen2424_3098 |
| BCAL0562 | 15.72 | 2E-03 | BCAL0562 | negative regulator of flagellin synthesis (anti-sigma-28 factor) | Bcen2424_3027 |
| BCAL0625 | 2.491 | 3E-02 | BCAL0625 | LysR family regulatory protein | Bcen2424_2967 |
| BCAL1058 | 7.62 | 4E-05 | BCAL1058 | AraC family regulatory protein | No homolog |
| BCAL1180 | 3.39 | 2E-05 | BCAL1180 | LysR family regulatory protein | No homolog |
| BCAL1182 | 5.019 | 3E-04 | BCAL1182 | TetR family regulatory protein | No homolog |
| BCAL1506 | 3.244 | 1E-04 | BCAL1506 | N utilization substance protein A | Bcen2424_1499 |
| BCAL1513 | 2.226 | 7E-03 | BCAL1513 | MarR family regulatory protein | Bcen2424_1506 |
| BCAL1595 | 3.112 | 1E-04 | BCAL1595 | putative DNA-binding phage protein | No homolog |
| BCAL1688 | 16.64 | 1E-04 | BCAL1688 | putative RNA polymerase sigma factor | Bcen2424_1632 |
| BCAL1870 | 5.649 | 2E-07 | BCAL1870 | putative transcription accessory protein | Bcen2424_1798 |
| BCAL1901 | 2.429 | 1E-03 | BCAL1901 | transcription termination factor rho | Bcen2424_1829 |
| BCAL2446 | 4.171 | 6E-05 | BCAL2446 | putative aminotransferase | Bcen2424_2351 |
| BCAL2488 | 3.844 | 8E-05 | BCAL2488 | LysR family regulatory protein | No homolog |
| BCAL2540 | 2.34 | 8E-05 | BCAL2540 | LysR family regulatory protein | No homolog |
| BCAL2577 | 2.253 | 9E-05 | BCAL2577 | DJ-1/PfpI family protein | No homolog |
| BCAL2579 | 2.347 | 2E-04 | BCAL2579 | LysR family regulatory protein | No homolog |
| BCAL2586 | 2.013 | 3E-03 | BCAL2586 | AraC family regulatory protein | No homolog |
| BCAL2732 | 2.006 | 2E-02 | BCAL2732 | cold shock-like protein | Bcen2424_2520 |
| BCAL2800 | 2.051 | 6E-05 | BCAL2800 | putative transcriptional regulator | Bcen2424_2587 |
| BCAL3011 | 2.065 | 8E-05 | BCAL3011 | DNA-directed RNA polymerase omega chain | Bcen2424_1000 |
| BCAL3169 | 2.297 | 2E-05 | BCAL3169 | LysR family regulatory protein | Bcen2424_0836 |
| BCAL3190 | 2.092 | 4E-04 | BCAL3190 | IclR family regulatory protein | Bcen2424_0814 |
| BCAL3196 | 3.045 | 6E-04 | BCAL3196 | putative ATP-binding regulatory protein | Bcen2424_0807 |
| BCAL3335 | 2.302 | 2E-04 | BCAL3335 | DNA-binding protein | Bcen2424_0692 |
| BCAM0009 | 2.033 | 5E-15 | BCAM0009 | putative DNA-binding protein* | Bcen2424_5700 |
| BCAM0197 | 2.333 | 4E-05 | BCAM0197 | LysR family regulatory protein | No homolog |
| BCAM0430 | 2.054 | 2E-05 | BCAM0430 | putative transcriptional regulator | No homolog |
| BCAM0518 | 2.18 | 8E-03 | BCAM0518 | LysR family regulatory protein | Bcen2424_3495 |
| BCAM0677 | 2.612 | 5E-04 | BCAM0677 | AraC family regulatory protein | Bcen2424_3641 |
| BCAM1153 | 2.23 | 8E-04 | BCAM1153 | LysR family regulatory protein | No homolog |
| BCAM1466 | 2.995 | 1E-08 | BCAM1466 | IclR family regulatory protein | No homolog |
| BCAM1928 | 3.378 | 2E-03 | BCAM1928 | putative transcription elongation factor | Bcen2424_4734 |
| BCAM2137 | 2.321 | 5E-08 | BCAM2137 | LysR family regulatory protein | No homolog |
| BCAM2158 | 2.126 | 8E-03 | BCAM2158 | putative DNA-binding protein | Bcen2424_4922 |
| BCAM2394 | 2.145 | 3E-03 | BCAM2394 | GntR family regulatory protein | No homolog |
| BCAM2434 | 2.595 | 2E-03 | BCAM2434 | TetR family regulatory protein | Bcen2424_5241 |
| BCAM2538 | 2.569 | 8E-05 | BCAM2538 | LysR family regulatory protein | Bcen2424_5348 |
| BCAM2548 | 2.606 | 2E-09 | BCAM2548 | TetR family regulatory protein | No homolog |
| BCAM2773 | 2.856 | 2E-04 | BCAM2773 | putative DNA-binding protein | No homolog |
| BCAM2774 | 2.607 | 8E-04 | BCAM2774 | GntR family regulatory protein | Bcen2424_5616 |
| BCAM2794 | 2.559 | 1E-06 | BCAM2794 | MarR family regulatory protein | No homolog |
| BCAM2812 | 2.462 | 2E-03 | BCAM2812 | LysR family regulatory protein | Bcen2424_5665 |
| BCAS0062 | 2.125 | 2E-05 | BCAS0062 | LysR family regulatory protein | Bcen2424_6146 |
| BCAS0258 | 2.389 | 8E-03 | BCAS0258 | GntR family regulatory protein | No homolog |
| BCAS0712 | 2.32 | 7E-04 | BCAS0712 | AnsC family regulatory protein | No homolog |
| BCAS0715 | 2.651 | 9E-04 | BCAS0715 | LysR family regulatory protein | No homolog |
| BCAS0717 | 10.12 | 1E-04 | BCAS0717 | hypothetical protein | No homolog |
|  |  |  |  | ***Replication, recombination, and repair (L)*** |  |
| BCAL0079 | 3.461 | 2E-05 | BCAL0079 | ATP-dependent DNA helicase Rep | Bcen2424_0150 |
| BCAL0178 | 2.88 | 1E-04 | BCAL0178 | putative DNA methyltransferase | No homolog |
| BCAL0421 | 3.146 | 1E-04 | BCAL0421 | DNA gyrase subunit B | Bcen2424_0003 |
| BCAL0825 | 2.75 | 4E-04 | BCAL0825 | excinuclease ABC subunit A | Bcen2424_2779 |
| BCAL0881 | 2.358 | 1E-02 | BCAL0881 | putative dATP pyrophosphohydrolase | Bcen2424_2724 |
| BCAL0933 | 5.148 | 3E-04 | BCAL0933 | putative ATP-dependent RNA helicase 1 | Bcen2424_2670 |
| BCAL1412 | 8.439 | 8E-06 | BCAL1412 | NUDIX hydrolase | Bcen2424_1404 |
| BCAL1519 | 2.417 | 5E-03 | BCAL1519 | putative transposase | Bcen2424_1512 |
| BCAL1585 | 3.014 | 8E-05 | BCAL1585 | histone-like DNA-binding phage protein | No homolog |
| BCAL2060 | 3.797 | 6E-23 | BCAL2060 | putative exonuclease | No homolog |
| BCAL2077 | 2.756 | 3E-04 | BCAL2077 | ribonuclease HII | No homolog |
| BCAL2096 | 2.249 | 3E-11 | BCAL2096 | DNA ligase | Bcen2424_2024 |
| BCAL2117 | 2.108 | 3E-02 | BCAL2117 | putative ATP-dependent RNA helicase* | Bcen2424_2047 |
| BCAL2149 | 2.414 | 6E-04 | BCAL2149 | HhH-GPD superfamily base excision DNA repair protein* | Bcen2424_2077 |
| BCAL2184 | 2.191 | 3E-04 | BCAL2184 | putative TatD related DNase | Bcen2424_2112 |
| BCAL2188 | 4.12 | 2E-04 | BCAL2188 | putative single-stranded-DNA-specific exonuclease | Bcen2424_2116 |
| BCAL2220 | 3.059 | 2E-11 | BCAL2220 | endonuclease/exonuclease/phosphatase family protein | No homolog |
| BCAL2230 | 2.291 | 2E-03 | BCAL2230 | putative ATP-dependent helicase* | Bcen2424_2155 |
| BCAL2329 | 2.142 | 1E-10 | BCAL2329 | NUDIX hydrolase | Bcen2424_2234 |
| BCAL2440 | 4.461 | 9E-04 | BCAL2440 | hypothetical protein | Bcen2424_2345 |
| BCAL2454 | 4.683 | 1E-10 | BCAL2454 | topoisomerase IV subunit A | Bcen2424_2359 |
| BCAL2455 | 2.952 | 1E-03 | BCAL2455 | topoisomerase IV subunit B | Bcen2424_2360 |
| BCAL2675 | 5.008 | 7E-06 | BCAL2675 | putative DNA polymerase III chi subunit | Bcen2424_2465 |
| BCAL2758 | 2.316 | 2E-07 | BCAL2758 | putative exodeoxyribonuclease VII large subunit | Bcen2424_2544 |
| BCAL2943 | 2.378 | 2E-17 | BCAL2943 | hypothetical protein* | Bcen2424_1054 |
| BCAL2957 | 3.091 | 1E-04 | BCAL2957 | DNA Gyrase, subunit A | No homolog |
| BCAL3252 | 2.034 | 3E-03 | BCAL3252 | putative transposase | No homolog |
| BCAL3317 | 2.266 | 1E-04 | BCAL3317 | hypothetical protein | Bcen2424_0709 |
| BCAL3338 | 4.377 | 4E-10 | BCAL3338 | holliday junction DNA helicase | Bcen2424_0689 |
| BCAL3494 | 4.814 | 3E-04 | BCAL3494 | type III restriction-modification system methylase | Bcen2424_0028 |
| BCAL3530 | 2.304 | 1E-03 | BCAL3530 | DNA-binding protein HU-alpha | Bcen2424_0065 |
| BCAM0173 | 2.465 | 2E-12 | BCAM0173 | ATP-independent RNA helicase | No homolog |
| BCAM1558 | 2.777 | 8E-04 | BCAM1558 | putative recombination-associated protein | Bcen2424_4426 |
| pBCA057 | 2.884 | 1E-03 | pBCA057 | putative conjugative transfer protein | No homolog |
|  |  |  |  | ***Chromatin structure and dynamics (B)*** |  |
| BCAL2940 | 4.683 | 4E-06 | BCAL2940 | putative histone deacetylase-family protein | Bcen2424_1057 |
|  |  |  |  | ***Cell cycle control, cell division, chromosome partitioning (D)*** |  |
| BCAL0143 | 5.563 | 6E-04 | BCAL0143 | putative flagellar biosynthesis protein | Bcen2424_0270 |
| BCAL0478 | 2.731 | 7E-04 | BCAL0478 | rod shape-determining protein | Bcen2424_3114 |
| BCAL0678 | 2.399 | 3E-13 | BCAL0678 | hypothetical protein* | Bcen2424_2920 |
| BCAL2147 | 2.98 | 2E-04 | BCAL2147 | tRNA(Ile)-lysidine synthase | Bcen2424_2075 |
| BCAL2178 | 2.328 | 1E-04 | BCAL2178 | septum formation initiator | Bcen2424_2106 |
| BCAL2417 | 2.14 | 2E-04 | BCAL2417 | putative DNA translocase* | No homolog |
| BCAL3025 | 4.43 | 4E-04 | BCAL3025 | cell division topological specificity factor | Bcen2424_0985 |
| BCAL3026 | 3.657 | 8E-07 | BCAL3026 | septum site-determining protein | Bcen2424_0984 |
| BCAL3027 | 3.404 | 3E-08 | BCAL3027 | probable septum site-determining protein | Bcen2424_0983 |
| BCAL3457 | 6.71 | 8E-04 | BCAL3457 | cell division protein FtsZ | Bcen2424_0564 |
| BCAL3458 | 10.86 | 2E-06 | BCAL3458 | cell division protein FtsA | No homolog |
| BCAL3463 | 7.813 | 2E-05 | BCAL3463 | cell division protein FtsW | Bcen2424_0558 |
| BCAL3469 | 9.469 | 2E-03 | BCAL3469 | cell division protein FtsL* | Bcen2424_0552 |
| BCAL3511 | 4.386 | 2E-04 | BCAL3510a | hypothetical protein | No homolog |
|  |  |  |  | ***Defense mechanisms (V)*** |  |
| BCAL0307 | 2.411 | 5E-03 | BCAL0307 | ABC transporter ATP-binding protein | Bcen2424_0420 |
| BCAL1285 | 2.15 | 3E-03 | BCAL1285 | MATE family transporter protein* | Bcen2424_1314 |
| BCAL1511 | 3.519 | 1E-10 | BCAL1511 | putative multidrug resistance transport protein | Bcen2424_1504 |
| BCAL1675 | 9.252 | 2E-04 | BCAL1675 | multidrug efflux system transporter protein AmrB | Bcen2424_1623 |
| BCAL1812 | 3.255 | 7E-04 | BCAL1812 | RND family efflux system transporter protein | Bcen2424_1740 |
| BCAL1907 | 3.736 | 2E-05 | BCAL1907 | putative multidrug resistance protein | No homolog |
| BCAL2185 | 2.129 | 3E-04 | BCAL2185 | lipoprotein releasing system ATP-binding protein | Bcen2424_2113 |
| BCAL2408 | 2.74 | 1E-04 | BCAL2408 | lipid A export ATP-binding/permease protein MsbA | No homolog |
| BCAL2821 | 7.029 | 3E-04 | BCAL2821 | RND family efflux system transporter protein | Bcen2424_2608 |
| BCAL3493 | 5.769 | 2E-05 | BCAL3493 | type III restriction system endonuclease | Bcen2424_0027 |
| BCAM1421 | 3.332 | 3E-04 | BCAM1421 | RND family efflux system transporter protein | No homolog |
| BCAM2140 | 2.343 | 2E-04 | BCAM2140 | transporter system transport protein* | No homolog |
| BCAM2141 | 2.982 | 4E-05 | BCAM2141 | ABC transporter ATP-binding membrane protein | No homolog |
| BCAM2350 | 2.982 | 3E-06 | BCAM2350 | ABC transporter ATP-binding protein | Bcen2424_5107 |
| BCAS0081 | 2.328 | 2E-06 | BCAS0081 | ABC transporter ATP-binding membrane protein | Bcen2424_6140 |
| BCAS0716 | 6.78 | 1E-03 | BCAS0716 | putative restriction endonuclease | No homolog |
|  |  |  |  | ***Signal transduction mechanisms (T)*** |  |
| BCAL0128 | 9.973 | 3E-03 | BCAL0128 | chemotaxis two-component response regulator CheY | Bcen2424_0255 |
| BCAL0135 | 6.63 | 3E-03 | BCAL0135 | chemotaxis protein CheY | Bcen2424_0262 |
| BCAL1069 | 2.071 | 2E-02 | BCAL1069 | putative cyclic-di-GMP signaling protein | Bcen2424_1189 |
| BCAL1275 | 2.22 | 6E-06 | BCAL1275 | phosphate regulon two-component regulatory system, response regulator protein | Bcen2424_1305 |
| BCAL1480 | 2.474 | 1E-07 | BCAL1480 | GTP pyrophosphokinase | Bcen2424_1473 |
| BCAL2268 | 4.518 | 4E-04 | BCAL2268 | putative stress protein | No homolog |
| BCAL3010 | 2.154 | 3E-07 | BCAL3010 | guanosine-3',5'-bis(diphosphate) 3'-pyrophosphohydrolase* | Bcen2424_1001 |
| BCAM0110 | 2.686 | 1E-07 | BCAM0110 | two-component regulatory system, sensor kinase protein | No homolog |
| BCAM0111 | 2.053 | 3E-04 | BCAM0111 | two-component regulatory system, response regulator protein | Bcen2424_5811 |
| BCAM0221 | 3.391 | 9E-05 | BCAM0221 | two-component regulatory system, response regulator protein | No homolog |
| BCAM0227 | 3.017 | 4E-04 | BCAM0227 | hybrid two-component system kinase-response regulator protein | No homolog |
| BCAM0379 | 4.209 | 5E-05 | BCAM0379 | hybrid two-component system kinase-response regulator protein* | Bcen2424_3368 |
| BCAM0381 | 3.498 | 1E-06 | BCAM0381 | two-component regulatory system, response regulator protein | Bcen2424_3370 |
| BCAM1382 | 2.142 | 7E-03 | BCAM1382 | two-component regulatory system, response regulator protein | No homolog |
| BCAM1417 | 2.313 | 8E-03 | BCAM1417 | two-component regulatory system, sensor kinase protein | No homolog |
| BCAM1418 | 5.762 | 2E-09 | BCAM1418 | two-component regulatory system, response regulator protein | Bcen2424_4290 |
| BCAM1494 | 2.098 | 5E-05 | BCAM1494 | two-component regulatory system, sensor kinase protein | Bcen2424_4366 |
| BCAM1870 | 6.673 | 2E-03 | BCAM1870 | N-acylhomoserine lactone synthase CepI | Bcen2424_4726 |
| BCAM2175 | 2.338 | 1E-11 | BCAM2175 | two-component regulatory system, response regulator protein | Bcen2424_4939 |
| BCAM2836 | 3.549 | 4E-03 | BCAM2836 | putative diguanylate cyclase | No homolog |
| BCAS0707 | 3.028 | 8E-08 | BCAS0707 | two-component regulatory system, response regulator protein | No homolog |
| BCAS0708 | 4.389 | 3E-04 | BCAS0708 | two-component regulatory system, sensor kinase protein | No homolog |
| BCAS0709 | 2.759 | 3E-03 | BCAS0709 | two-component regulatory system, response regulator protein | No homolog |
| pBCA055 | 7.054 | 2E-03 | pBCA055 | hypothetical protein | No homolog |
|  |  |  |  | ***Cell wall/membrane/envelope biogenesis (M)*** |  |
| BCAL0110 | 10.57 | 4E-06 | BCAL0110 | putative aminotransferase | No homolog |
| BCAL0159 | 2.255 | 4E-03 | BCAL0159 | putative RlpA-like lipoprotein* | Bcen2424_0286 |
| BCAL0274 | 3.793 | 2E-03 | BCAL0274 | penicillin-binding protein 1A | Bcen2424_0387 |
| BCAL0287 | 2.223 | 3E-18 | BCAL0287 | putative outer membrane protein* | Bcen2424_0400 |
| BCAL0304 | 2.142 | 4E-19 | BCAL0304 | VacJ-like lipoprotein* | Bcen2424_0417 |
| BCAL0310 | 4.205 | 5E-05 | BCAL0310 | UDP-N-acetylglucosamine 1-carboxyvinyltransferase | Bcen2424_0423 |
| BCAL0349 | 5.983 | 3E-04 | BCAL0349 | putative outer membrane protein* | Bcen2424_0483, Bcen2424_0480 |
| BCAL0479 | 4.324 | 3E-04 | BCAL0479 | penicillin-binding protein | No homolog |
| BCAL0480 | 2.032 | 4E-05 | BCAL0480 | putative rod shape-determining protein | Bcen2424_3112 |
| BCAL0481 | 2.448 | 3E-04 | BCAL0481 | putative rod shape-determining protein | Bcen2424_3111 |
| BCAL0508 | 3.95 | 5E-05 | BCAL0508 | putative lipid A biosynthesis acyltransferase | Bcen2424_3082 |
| BCAL0575 | 2.96 | 1E-02 | BCAL0575 | YcgR family protein | Bcen2424_3016 |
| BCAL0587 | 2.585 | 8E-07 | BCAL0587 | putative lipoprotein* | Bcen2424_3005 |
| BCAL0691 | 4.038 | 1E-03 | BCAL0691 | putative cytidylyltransferase | Bcen2424_2907 |
| BCAL0704 | 2.854 | 2E-06 | BCAL0704 | D-alanyl-D-alanine carboxypeptidase (penicillin-binding protein precursor)* | Bcen2424_2893 |
| BCAL0818 | 2.098 | 1E-03 | BCAL0818 | putative arabinose 5-phosphate isomerase | Bcen2424_2786 |
| BCAL0894 | 4.147 | 1E-04 | BCAL0894 | hypothetical protein* | No homolog |
| BCAL0960 | 3.24 | 2E-03 | BCAL0960 | O-antigen polymerase family protein* | No homolog |
| BCAL1258 | 2.699 | 6E-05 | BCAL1258 | putative exported transglycosylase protein* | No homolog |
| BCAL1395 | 2.365 | 2E-11 | BCAL1395 | putative cellulose synthase catalytic subunit (UDP-forming) | Bcen2424_1385 |
| BCAL1674 | 2.888 | 9E-03 | BCAL1674 | multidrug efflux system AmrA protein* | No homolog |
| BCAL1676 | 3.35 | 4E-03 | BCAL1676 | multidrug efflux system outer membrane protein* | Bcen2424_1624 |
| BCAL1906 | 3.241 | 2E-04 | BCAL1906 | hypothetical protein | Bcen2424_1834 |
| BCAL1929 | 8.55 | 6E-05 | BCAL1929 | putative undecaprenyl phosphate-alpha-4-amino-4-deoxy-l-arabinose arabinosy transferase* | Bcen2424_1857 |
| BCAL1931 | 2.056 | 9E-03 | BCAL1931 | putative UDP-4-amino-4-deoxy-l-arabinose--oxoglutarate aminotransferas | Bcen2424_1859 |
| BCAL1932 | 2.277 | 4E-06 | BCAL1932 | putative undecaprenyl-phosphate 4-deoxy-4-formamido-l-arabinose transferase | Bcen2424_1860 |
| BCAL2024 | 2.942 | 2E-03 | BCAL2024 | large-conductance mechanosensitive channel protein | No homolog |
| BCAL2078 | 4.742 | 3E-04 | BCAL2078 | putative lipid-A-disaccharide synthase | Bcen2424_2006 |
| BCAL2082 | 4.144 | 9E-05 | BCAL2082 | chaperone protein Skp precursor* | Bcen2424_2010 |
| BCAL2083 | 3.28 | 1E-04 | BCAL2083 | Outer membrane protein assembly factor YaeT* | Bcen2424_2011 |
| BCAL2084 | 2.192 | 3E-05 | BCAL2084 | protease EcfE | Bcen2424_2012 |
| BCAL2166 | 2.954 | 6E-04 | BCAL2166 | putative lipoprotein* | Bcen2424_2094 |
| BCAL2186 | 2.014 | 1E-04 | BCAL2186 | putative lipoprotein releasing system transmembrane protein | Bcen2424_2114 |
| BCAL2291 | 11.05 | 2E-05 | BCAL2291 | TonB family protein | Bcen2424_2198 |
| BCAL2403 | 2.657 | 1E-17 | BCAL2403 | putative LPS core biosynthesis protein | No homolog |
| BCAL2404 | 3.056 | 4E-05 | BCAL2404 | putative glycosyltransferase | No homolog |
| BCAL2405 | 4.623 | 5E-07 | BCAL2405 | hypothetical protein | No homolog |
| BCAL2406 | 2.872 | 6E-05 | BCAL2406 | putative glycosyltransferase | No homolog |
| BCAL2407 | 2.053 | 2E-04 | BCAL2407 | putative glycosyltransferase | No homolog |
| BCAL2482 | 2.26 | 4E-04 | BCAL2482 | putative outer membrane protein* | No homolog |
| BCAL2648 | 3.258 | 4E-03 | BCAL2648 | putative outer membrane protein | Bcen2424_2439 |
| BCAL2670 | 2.999 | 2E-04 | BCAL2670 | prolipoprotein diacylglyceryl transferase | Bcen2424_2460 |
| BCAL2761 | 2.519 | 3E-03 | BCAL2761 | putative 3-deoxy-manno-octulosonate cytidylyltransferase | Bcen2424_2547 |
| BCAL2768 | 2.558 | 2E-06 | BCAL2768 | putative UDP-N-acetylenolpyruvoylglucosamine reductase | Bcen2424_2554 |
| BCAL2820 | 6.082 | 1E-04 | BCAL2820 | efflux system outer membrane protein* | Bcen2424_2607 |
| BCAL2822 | 4.783 | 2E-04 | BCAL2822 | efflux system transport protein* | Bcen2424_2609 |
| BCAL2941 | 2.924 | 1E-05 | BCAL2941 | putative exported transglycosylase* | Bcen2424_1056 |
| BCAL2944 | 2.362 | 2E-04 | BCAL2944 | ADP-l-glycero-D-manno-heptose-6-epimerase | Bcen2424_1053 |
| BCAL2945 | 3.1 | 2E-14 | BCAL2945 | D-beta-D-heptose 7-phosphate kinase | No homolog |
| BCAL2946 | 3.075 | 4E-07 | BCAL2946 | putative UDP-glucose dehydrogenase | Bcen2424_1051 |
| BCAL2958 | 2.032 | 1E-03 | BCAL2958 | putative ompA family protein* | Bcen2424_1039 |
| BCAL3057 | 3.072 | 7E-03 | BCAL3057 | putative lipoprotein | Bcen2424_0953 |
| BCAL3110 | 2.405 | 2E-03 | BCAL3110 | putative 3-deoxy-D-manno-octulosonic acid transferase | No homolog |
| BCAL3118 | 2.489 | 6E-03 | BCAL3118 | UDP-N-acetylglucosamine-1-P transferase* | Bcen2424_0888 |
| BCAL3124 | 3.97 | 5E-04 | BCAL3124 | glycosyltransferase | No homolog |
| BCAL3128 | 3.338 | 2E-03 | BCAL3128 | glycosyltransferase | No homolog |
| BCAL3129 | 3.912 | 3E-05 | BCAL3129 | nucleotide sugar aminotransferase | No homolog |
| BCAL3132 | 3.173 | 1E-04 | BCAL3132 | dTDP-4-keto-L-rhamnose reductase | No homolog |
| BCAL3133 | 2.093 | 2E-03 | BCAL3133 | dTDP-4-keto-6-deoxy-D-glucose 3,5-epimerase | Bcen2424_0873 |
| BCAL3134 | 2.592 | 2E-03 | BCAL3134 | glucose-1-phosphate thymidylyltransferase | Bcen2424_0872 |
| BCAL3202 | 2.476 | 1E-03 | BCAL3202 | possible TolA-related transport transmembrane protein | Bcen2424_0801 |
| BCAL3219 | 2.022 | 3E-03 | BCAL3219 | UDP-3-O-[3-hydroxymyristoyl] N-acetylglucosamine deacetylase | No homolog |
| BCAL3239 | 2.949 | 4E-04 | BCAL3239 | glucosyltransferase | No homolog |
| BCAL3242 | 4.101 | 6E-04 | BCAL3242 | putative capsule polysaccharide export protein, ABC transporter membrane protein* | No homolog |
| BCAL3243 | 2.943 | 2E-08 | BCAL3243 | putative capsular polysaccharide biosynthesis/export protein* | No homolog |
| BCAL3244 | 2.246 | 1E-06 | BCAL3244 | glycosyltransferase | No homolog |
| BCAL3245 | 9.186 | 2E-04 | BCAL3245 | capsule polysaccharide export protein | No homolog |
| BCAL3246 | 5.549 | 8E-04 | BCAL3246 | putative GDP-mannose pyrophosphorylase | No homolog |
| BCAL3247 | 12.35 | 1E-22 | BCAL3247 | mechanosensitive ion channel protein | Bcen2424_0768 |
| BCAL3308 | 2.33 | 3E-04 | BCAL3308 | putative peptidase | Bcen2424_0718 |
| BCAL3372 | 2.56 | 1E-03 | BCAL3372 | putative lipoprotein* | Bcen2424_0655 |
| BCAL3455 | 2.835 | 6E-11 | BCAL3455 | UDP-3-O-[3-hydroxymyristoyl] N-acetylglucosamine deacetylase | Bcen2424_0566 |
| BCAL3459 | 5.623 | 3E-04 | BCAL3459 | cell division protein FtsQ* | Bcen2424_0562 |
| BCAL3460 | 3.97 | 5E-05 | BCAL3460 | D-alanine--D-alanine ligase B | Bcen2424_0561 |
| BCAL3461 | 6.349 | 2E-04 | BCAL3461 | UDP-N-acetylmuramate--alanine ligase | Bcen2424_0560 |
| BCAL3462 | 6.766 | 1E-05 | BCAL3462 | UDP-N-acetylglucosamine--N-acetylmuramyl-(penta peptide) pyrophosphoryl-undecaprenol N-acetylglucosamine transferase MurG* | No homolog |
| BCAL3464 | 9.813 | 6E-05 | BCAL3464 | UDP-N-acetylmuramoylalanine--D-glutamate ligase | No homolog |
| BCAL3465 | 10.88 | 6E-04 | BCAL3465 | phospho-N-acetylmuramoyl-pentapeptide-transfera se | Bcen2424_0556 |
| BCAL3467 | 8.355 | 2E-05 | BCAL3467 | UDP-N-acetylmuramoylalanyl-D-glutamate--2,6-dia minopimelate ligase | Bcen2424_0554 |
| BCAL3468 | 7.708 | 4E-04 | BCAL3468 | peptidoglycan synthetase FtsI* | No homolog |
| BCAL3470 | 5.539 | 8E-04 | BCAL3470 | S-adenosyl-methyltransferase MraW | Bcen2424_0551 |
| BCAM0678 | 2.465 | 2E-16 | BCAM0678 | putative acetyltransferase-GNAT family | No homolog |
| BCAM1419 | 3.251 | 3E-09 | BCAM1419 | efflux system outer membrane protein* | Bcen2424_4291 |
| BCAM1420 | 4.458 | 2E-03 | BCAM1420 | efflux system transport protein | No homolog |
| BCAM1472 | 2.109 | 7E-05 | BCAM1472 | putative glycosyltransferase | No homolog |
| BCAM1543 | 3.111 | 2E-07 | BCAM1543 | putative glycosyltransferase* | No homolog |
| BCAM1754 | 2.714 | 3E-04 | BCAM1754 | putative mechanosensitive ion channel | Bcen2424_4577 |
| BCAM1931 | 6.987 | 2E-02 | BCAM1931 | putative porin* | Bcen2424_4735 |
| BCAM2088 | 2.41 | 7E-03 | BCAM2088 | putative exported cell wall amidase* | Bcen2424_4895 |
| BCAM2142 | 3.046 | 5E-06 | BCAM2142 | transport system outer membrane protein* | No homolog |
| BCAM2253 | 2.232 | 3E-03 | BCAM2253 | RHS-family protein | No homolog |
| BCAM2829 | 2.343 | 1E-07 | BCAM2829 | putative VacJ family lipoprotein* | Bcen2424_5677 |
| BCAS0130 | 5.104 | 2E-04 | BCAS0130 | putative ABC transporter substrate-binding protein* | Bcen2424_6100 |
|  |  |  |  | ***Cell motility (N)*** |  |
| BCAL0113 | 27.58 | 1E-03 | BCAL0113 | B-type flagellar hook-associated protein 2 (HAP2) | Bcen2424_0240 |
| BCAL0114 | 57.28 | 2E-03 | BCAL0114 | flagellin (type II) | No homolog |
| BCAL0126 | 8.657 | 3E-03 | BCAL0126 | chemotaxis protein MotA | Bcen2424_0253 |
| BCAL0127 | 9.614 | 2E-03 | BCAL0127 | chemotaxis protein MotB | Bcen2424_0254 |
| BCAL0129 | 12.56 | 2E-03 | BCAL0129 | chemotaxis two-component sensor kinase CheA | Bcen2424_0256 |
| BCAL0130 | 8.297 | 4E-03 | BCAL0130 | chemotaxis protein CheW | Bcen2424_0257 |
| BCAL0131 | 4.293 | 6E-03 | BCAL0131 | methyl-accepting chemotaxis protein | No homolog |
| BCAL0132 | 9.717 | 2E-03 | BCAL0132 | chemotaxis protein methyltransferase | No homolog |
| BCAL0133 | 5.814 | 2E-03 | BCAL0133 | putative chemoreceptor glutamine deamidase cheD | No homolog |
| BCAL0134 | 7.503 | 4E-03 | BCAL0134 | chemotaxis response regulator protein-glutamate methylesterase 1 | Bcen2424_0261 |
| BCAL0136 | 9.931 | 3E-03 | BCAL0136 | chemotaxis protein CheZ | Bcen2424_0263 |
| BCAL0140 | 15.29 | 7E-04 | BCAL0140 | flagellar biosynthetic protein FlhB | Bcen2424_0267 |
| BCAL0142 | 5.501 | 5E-06 | BCAL0142 | putative flagellar biosynthesis protein | Bcen2424_0269 |
| BCAL0520 | 6.415 | 2E-04 | BCAL0520 | putative flagellar hook-length control protein FliK | Bcen2424_3069 |
| BCAL0521 | 7.711 | 1E-03 | BCAL0521 | flagellar FliJ protein | Bcen2424_3068 |
| BCAL0522 | 9.514 | 2E-04 | BCAL0522 | flagellum-specific ATP synthase FliI* | Bcen2424_3067 |
| BCAL0523 | 7.678 | 4E-04 | BCAL0523 | flagellar assembly protein FliH | Bcen2424_3066 |
| BCAL0524 | 6.549 | 3E-04 | BCAL0524 | flagellar motor switch protein FliG | Bcen2424_3065 |
| BCAL0525 | 5.801 | 4E-05 | BCAL0525 | flagellar M-ring protein FliF | Bcen2424_3064 |
| BCAL0526 | 3.538 | 2E-03 | BCAL0526 | flagellar hook-basal body complex protein FliE* | Bcen2424_3063 |
| BCAL0527 | 8.086 | 3E-03 | BCAL0527 | flagellar protein FliS | No homolog |
| BCAL0561 | 6.415 | 4E-03 | BCAL0561 | flagella synthesis protein FlgN | Bcen2424_3028 |
| BCAL0564 | 8.186 | 9E-04 | BCAL0564 | flagellar basal-body rod protein FlgB (putative proximal rod protein) | Bcen2424_3025 |
| BCAL0565 | 8.55 | 1E-03 | BCAL0565 | flagellar basal-body rod protein FlgC (putative proximal rod protein)* | Bcen2424_3024 |
| BCAL0566 | 12.26 | 6E-04 | BCAL0566 | basal-body rod modification protein FlgD | Bcen2424_3023 |
| BCAL0567 | 13.69 | 3E-04 | BCAL0567 | flagellar hook protein 1 FlgE1* | Bcen2424_3022 |
| BCAL0568 | 13.33 | 6E-04 | BCAL0568 | flagellar basal-body rod protein FlgF (putative proximal rod protein) | Bcen2424_3021 |
| BCAL0569 | 13.61 | 7E-04 | BCAL0569 | flagellar basal-body rod protein FlgG (distal rod protein)* | Bcen2424_3020 |
| BCAL0570 | 13.48 | 4E-04 | BCAL0570 | flagellar L-ring protein precursor (basal body L-ring protein)* | Bcen2424_3019 |
| BCAL0571 | 6.561 | 8E-04 | BCAL0571 | flagellar P-ring protein precursor (basal body P-ring protein)* | Bcen2424_3018 |
| BCAL0572 | 5.557 | 1E-03 | BCAL0572 | peptidoglycan hydrolase FlgJ (muramidase FlgJ)* | No homolog |
| BCAL0576 | 10.85 | 4E-03 | BCAL0576 | flagellar hook-associated protein 1 (HAP1)* | Bcen2424_3015 |
| BCAL0577 | 16.07 | 2E-03 | BCAL0577 | flagellar hook-associated protein 3 (HAP3) | Bcen2424_3014 |
| BCAL0762 | 3.557 | 8E-03 | BCAL0762 | putative methyl-accepting chemotaxis protein* | Bcen2424_2834 |
| BCAL1662 | 2.198 | 2E-03 | BCAL1662 | putative methyl-accepting chemotaxis protein* | Bcen2424_1611 |
| BCAL1677 | 21.27 | 3E-05 | BCAL1677 | putative type-1 fimbrial protein* | Bcen2424_1626 |
| BCAL1679 | 3.444 | 1E-04 | BCAL1679 | putative fimbrial chaperone* | Bcen2424_1628 |
| BCAL3445 | 2.073 | 2E-03 | BCAL3445 | putative type IV pilus assembly protein | Bcen2424_0578 |
| BCAL3447 | 2.621 | 2E-04 | BCAL3447 | type IV prepilin leader peptide type M1 | Bcen2424_0576 |
| BCAL3501 | 3.323 | 5E-05 | BCAL3501 | flagellar biosynthetic protein FliR* | Bcen2424_0035 |
| BCAL3502 | 2.567 | 8E-06 | BCAL3502 | flagellar biosynthetic protein FliQ | Bcen2424_0036 |
| BCAL3503 | 2.137 | 2E-03 | BCAL3503 | flagellar biosynthetic protein FliP precursor* | Bcen2424_0037 |
| BCAL3505 | 4.334 | 1E-03 | BCAL3505 | flagellar motor switch protein FliN* | Bcen2424_0039 |
| BCAL3506 | 4.085 | 2E-04 | BCAL3506 | flagellar motor switch protein FliM | Bcen2424_0040 |
| BCAL3507 | 2.819 | 8E-04 | BCAL3507 | flagellar FliL protein* | No homolog |
| BCAL3521 | 3.073 | 3E-03 | BCAL3521 | type II secretion system protein I* | Bcen2424_0056 |
| BCAL3522 | 3.037 | 1E-04 | BCAL3522 | type II secretion system protein H | Bcen2424_0057 |
| BCAM1424 | 2.129 | 1E-02 | BCAM1424 | methyl-accepting chemotaxis protein* | Bcen2424_4296 |
| BCAM1503 | 2.776 | 3E-02 | BCAM1503 | putative methyl-accepting chemotaxis protein* | Bcen2424_4375 |
| BCAM1804 | 9.933 | 9E-03 | BCAM1804 | methyl-accepting chemotaxis protein* | Bcen2424_4665 |
| BCAM2048 | 2.5 | 8E-03 | BCAM2048 | type III secretion ssytem protein | Bcen2424_4847 |
| BCAM2055 | 2.839 | 3E-03 | BCAM2055 | type III secretion system protein* | Bcen2424_4854 |
| BCAM2564 | 4.554 | 1E-02 | BCAM2564 | putative aerotaxis receptor | Bcen2424_5364 |
| BCAM2689 | 2.216 | 8E-03 | BCAM2689 | putative methyl-accepting chemotaxis protein | Bcen2424_5500 |
| BCAS0104 | 3.791 | 9E-05 | BCAS0104 | A-type flagellar hook-associated protein 2 (HAP2)* | No homolog |
| BCAS0632 | 3.446 | 3E-06 | BCAS0632 | hybrid two-component system kinase-response regulator protein | No homolog |
| IG1_155066 | 9.16 | 2E-03 | BCAL0132 | chemotaxis protein methyltransferase | Bcen2424_0259 |
|  |  |  |  | ***Intracellular trafficking, secretion, and vesicular transport (U)*** |  |
| BCAL0220 | 4.551 | 3E-04 | BCAL0220 | preprotein translocase SecE subunit | Bcen2424_0334 |
| BCAL0254 | 4.057 | 2E-03 | BCAL0254 | preprotein translocase SecY subunit | Bcen2424_0368 |
| BCAL0426 | 4.568 | 4E-04 | BCAL0426 | hypothetical protein* | Bcen2424_3163 |
| BCAL0742 | 2.415 | 4E-05 | BCAL0742 | protein-export protein SecB | Bcen2424_2854 |
| BCAL2292 | 14.23 | 3E-04 | BCAL2292 | putative bipolymer transport protein | Bcen2424_2199 |
| BCAL2293 | 14.54 | 6E-05 | BCAL2293 | putative biopolymer transport protein | Bcen2424_2200 |
| BCAL2345 | 2.031 | 4E-05 | BCAL2345 | protein-export membrane protein SecG | Bcen2424_2250 |
| BCAL2475 | 3.248 | 2E-04 | BCAL2475 | hypothetical protein* | No homolog |
| BCAL3200 | 2.909 | 1E-03 | BCAL3200 | putative TolQ transport transmembrane protein | Bcen2424_0803 |
| BCAL3201 | 2.314 | 1E-03 | BCAL3201 | putative TolR-related protein | Bcen2424_0802 |
| BCAL3203 | 2.29 | 9E-03 | BCAL3203 | putative periplasmic TolB protein* | Bcen2424_0800 |
| BCAL3305 | 3.848 | 2E-04 | BCAL3305 | preprotein translocase subunit | Bcen2424_0721 |
| BCAL3306 | 8.049 | 7E-04 | BCAL3306 | putative protein-export membrane protein SecD* | Bcen2424_0720 |
| BCAL3307 | 5.3 | 4E-04 | BCAL3307 | putative protein-export membrane protein SecF | Bcen2424_0719 |
| BCAL3374 | 2.181 | 2E-04 | BCAL3374 | putative transport-related membrane protein | Bcen2424_0653 |
| BCAL3375 | 2.666 | 1E-03 | BCAL3375 | putative transport-related membrane protein | Bcen2424_0652 |
| BCAL3435 | 2.129 | 8E-04 | BCAL3435 | MarC family integral membrane protein | Bcen2424_0588 |
| BCAL3516 | 2.147 | 4E-04 | BCAL3516 | type II secretion system protein M | Bcen2424_0052 |
| BCAL3519 | 2.017 | 1E-02 | BCAL3519 | type II secretion system protein K | Bcen2424_0054 |
| BCAL3520 | 2.073 | 2E-03 | BCAL3520 | type II secretion system protein J | Bcen2424_0055 |
| BCAM1709 | 4.679 | 9E-04 | BCAM1709 | MarC family protein | No homolog |
| BCAM2041 | 3.674 | 2E-04 | BCAM2041 | type III secretion system protein | Bcen2424_4840 |
| BCAM2051 | 2.131 | 5E-03 | BCAM2051 | type III secretion system protein | Bcen2424_4850 |
| pBCA041 | 2.26 | 5E-04 | pBCA041 | putative TraC conjugative transfer protein | No homolog |
| pBCA059 | 2.565 | 2E-05 | pBCA059 | putative TraD conjugative transfer protein | No homolog |
|  |  |  |  | ***Posttranslational modification, protein turnover, chaperones (O)*** |  |
| BCAL0111 | 11.34 | 1E-04 | BCAL0111 | putative TPR repeat protein | No homolog |
| BCAL0331 | 2.106 | 2E-06 | BCAL0331 | putative stringent starvation protein A | Bcen2424_0444 |
| BCAL0347 | 2.291 | 3E-12 | BCAL0347 | putative type VI secretion system protein TssH | Bcen2424_0473 |
| BCAL0367 | 3.552 | 8E-04 | BCAL0367 | putative chaperone protein | No homolog |
| BCAL0677 | 2.949 | 1E-07 | BCAL0677 | thiol:disulfide interchange protein* | Bcen2424_2921 |
| BCAL0752 | 3.372 | 2E-06 | BCAL0752 | putative cytochrome c oxidase assembly protein | Bcen2424_2844 |
| BCAL0758 | 3.077 | 2E-06 | BCAL0758 | putative cytochrome oxidase assembly protein* | No homolog |
| BCAL0759 | 2.548 | 2E-03 | BCAL0759 | UbiA prenyltransferase family protein | Bcen2424_2837 |
| BCAL0792 | 2.103 | 7E-04 | BCAL0792 | putative maleylacetoacetate isomerase | Bcen2424_2810 |
| BCAL0849 | 18.15 | 4E-25 | BCAL0849 | metallo peptidase, subfamily M48B* | Bcen2424_2755 |
| BCAL0895 | 3.545 | 7E-05 | BCAL0895 | putative peptidyl-prolyl cis-trans isomerase* | Bcen2424_2710 |
| BCAL1733 | 2.477 | 9E-12 | BCAL1733 | putative glutathione S-transferase | Bcen2424_1682 |
| BCAL1876 | 7.339 | 2E-04 | BCAL1876 | protein HflC* | Bcen2424_1804 |
| BCAL1877 | 2.58 | 2E-17 | BCAL1877 | protein HflK | Bcen2424_1805 |
| BCAL1936 | 2.604 | 3E-06 | BCAL1936 | AhpC/TSA family protein | Bcen2424_1864 |
| BCAL1985 | 2.398 | 6E-05 | BCAL1985 | putative exported isomerase* | Bcen2424_1912 |
| BCAL1993 | 7.284 | 6E-06 | BCAL1993 | putative peptidyl-prolyl cis-trans isomerase | Bcen2424_1920 |
| BCAL1997 | 2.505 | 5E-18 | BCAL1997 | trigger factor | Bcen2424_1924 |
| BCAL2073 | 2.221 | 1E-06 | BCAL2073 | hypothetical protein | No homolog |
| BCAL2153 | 2.635 | 7E-04 | BCAL2153 | peptidyl-prolyl cis-trans isomerase B | Bcen2424_2081 |
| BCAL2321 | 2.338 | 4E-03 | BCAL2321 | putative glutathione S-transferase | Bcen2424_2226 |
| BCAL2323 | 2.183 | 5E-04 | BCAL2323 | putative glutathione S-transferase | Bcen2424_2228 |
| BCAL2442 | 3.414 | 4E-03 | BCAL2442 | chaperone protein HtpG | Bcen2424_2347 |
| BCAL2558 | 2.442 | 1E-15 | BCAL2558 | putative pyridine nucleotide-disulphide oxidoreductase | No homolog |
| BCAL2987 | 4.83 | 1E-07 | BCAL2987 | disulfide bond formation protein B | Bcen2424_1021 |
| BCAL3146 | 3.619 | 1E-02 | BCAL3146 | 60 kDa chaperonin 1 | Bcen2424_0860 |
| BCAL3147 | 3.956 | 7E-03 | BCAL3147 | 10 kDa chaperonin 1 | Bcen2424_0859 |
| BCAL3424 | 2.128 | 5E-06 | BCAL3424 | thiol peroxidase | Bcen2424_0601 |
| BCAL3456 | 5.964 | 3E-04 | BCAL3456 | putative thioredoxin reductase | Bcen2424_0565 |
| BCAM0913 | 2.256 | 1E-02 | BCAM0913 | O-sialoglycoprotein endopeptidase | Bcen2424_3881 |
| BCAM1744 | 13.32 | 3E-03 | BCAM1744 | serine peptidase, family S9* | No homolog |
| pBCA043 | 2.004 | 3E-04 | pBCA043 | thiol:disulfide interchange protein DsbC precursor* | No homolog |
|  |  |  |  | ***Energy production and conversion (C)*** |  |
| BCAL0029 | 2.858 | 1E-03 | BCAL0029 | putative ATP synthase protein I AtpI | Bcen2424_0099 |
| BCAL0030 | 22.03 | 1E-04 | BCAL0030 | ATP synthase A chain | Bcen2424_0100 |
| BCAL0031 | 15.44 | 2E-04 | BCAL0031 | ATP synthase C chain | Bcen2424_0101 |
| BCAL0032 | 15.04 | 9E-28 | BCAL0032 | ATP synthase B chain | Bcen2424_0102 |
| BCAL0033 | 20.74 | 3E-05 | BCAL0033 | ATP synthase delta chain | Bcen2424_0103 |
| BCAL0034 | 17.52 | 1E-06 | BCAL0034 | ATP synthase alpha chain | Bcen2424_0104 |
| BCAL0035 | 17.12 | 5E-29 | BCAL0035 | ATP synthase gamma chain | Bcen2424_0105 |
| BCAL0036 | 16.81 | 2E-06 | BCAL0036 | ATP synthase beta chain | Bcen2424_0106 |
| BCAL0037 | 18.59 | 9E-06 | BCAL0037 | ATP synthase epsilon chain | Bcen2424_0107 |
| BCAL0164 | 2.027 | 9E-05 | BCAL0164 | putative cytochrome c-551 precursor* | Bcen2424_0293 |
| BCAL0206 | 2.087 | 5E-03 | BCAL0206 | putative pyruvate ferredoxin/flavodoxin oxidoreductase | Bcen2424_0319 |
| BCAL0286 | 2.255 | 5E-06 | BCAL0286 | glycerophosphoryl diester phosphodiesterase | Bcen2424_0399 |
| BCAL0329 | 2.178 | 6E-05 | BCAL0329 | cytochrome b | Bcen2424_0442 |
| BCAL0366 | 4.2 | 8E-06 | BCAL0366 | nitroreductase family protein | Bcen2424_0501 |
| BCAL0408 | 2.552 | 3E-03 | BCAL0408 | putative phenylacetic acid degradation oxidoreductase | Bcen2424_0543 |
| BCAL0650 | 3.244 | 2E-03 | BCAL0650 | putative pyruvate-flavodoxin oxidoreductase | Bcen2424_2940 |
| BCAL0722 | 8.118 | 1E-03 | BCAL0722 | C4-dicarboxylate transport protein | No homolog |
| BCAL0743 | 2.687 | 1E-05 | BCAL0743 | putative glycerol-3-phosphate dehydrogenase (NAD(P)+) | No homolog |
| BCAL0749 | 3.218 | 2E-06 | BCAL0749 | putative cytochrome c oxidase* | Bcen2424_2847 |
| BCAL0750 | 3.705 | 6E-05 | BCAL0750 | cytochrome c oxidase polypeptide I | Bcen2424_2846 |
| BCAL0754 | 7.625 | 6E-05 | BCAL0754 | putative cytochrome c oxidase subunit III | Bcen2424_2842 |
| BCAL0850 | 14.06 | 1E-06 | BCAL0850 | glycolate permease* | No homolog |
| BCAL0851 | 127.3 | 2E-06 | BCAL0851 | putative iron-sulphur cluster containing protein | No homolog |
| BCAL0853 | 2.941 | 2E-05 | BCAL0853 | hypothetical protein | No homolog |
| BCAL0934 | 4.301 | 2E-04 | BCAL0934 | putative periplasmic cytochrome c containing protein* | Bcen2424_2669 |
| BCAL0935 | 4.44 | 2E-04 | BCAL0935 | putative periplasmic cytochrome c protein* | Bcen2424_2668 |
| BCAL0957 | 3.087 | 3E-04 | BCAL0957 | succinyl-CoA ligase alpha-chain | Bcen2424_2648 |
| BCAL1062 | 7.288 | 4E-05 | BCAL1062 | succinylglutamic semialdehyde dehydrogenase | Bcen2424_1183 |
| BCAL1515 | 6.201 | 2E-04 | BCAL1515 | 2-oxoglutarate dehydrogenase E1 component | No homolog |
| BCAL1516 | 7.801 | 1E-04 | BCAL1516 | dihydrolipoamide succinyltransferase component of 2-oxoglutarate dehydrogenase complex | Bcen2424_1509 |
| BCAL1517 | 9.523 | 7E-04 | BCAL1517 | dihydrolipoamide dehydrogenase | Bcen2424_1510 |
| BCAL1831 | 2.061 | 2E-02 | BCAL1831 | putative betaine aldehyde dehydrogenase | Bcen2424_1758 |
| BCAL2287 | 3.824 | 3E-04 | BCAL2287 | putative fumarate hydratase | Bcen2424_2194 |
| BCAL2298 | 4.047 | 2E-05 | BCAL2298 | hypothetical protein | Bcen2424_2206 |
| BCAL2331 | 3.675 | 2E-03 | BCAL2331 | NADH dehydrogenase I chain N* | No homolog |
| BCAL2332 | 3.157 | 2E-05 | BCAL2332 | NADH dehydrogenase I chain M | Bcen2424_2237 |
| BCAL2333 | 4.064 | 2E-04 | BCAL2333 | NADH-ubiquinone oxidoreductase I chain L | Bcen2424_2238 |
| BCAL2334 | 3.81 | 2E-04 | BCAL2334 | NADH-ubiquinone oxidoreductase I chain K | Bcen2424_2239 |
| BCAL2335 | 3.6 | 4E-03 | BCAL2335 | NADH dehydrogenase I chain J | Bcen2424_2240 |
| BCAL2336 | 2.947 | 1E-09 | BCAL2336 | putative NADH dehydrogenase I chain I | Bcen2424_2241 |
| BCAL2337 | 3.2 | 2E-06 | BCAL2337 | NADH dehydrogenase I chain H | Bcen2424_2242 |
| BCAL2338 | 3.041 | 2E-05 | BCAL2338 | putative NADH dehydrogenase I chain G | Bcen2424_2243 |
| BCAL2339 | 2.406 | 3E-04 | BCAL2339 | NADH dehydrogenase I chain F | Bcen2424_2244 |
| BCAL2340 | 2.946 | 4E-06 | BCAL2340 | putative NADH dehydrogenase I chain E | Bcen2424_2245 |
| BCAL2341 | 2.341 | 1E-03 | BCAL2341 | NADH dehydrogenase I chain D | Bcen2424_2246 |
| BCAL2342 | 2.546 | 6E-04 | BCAL2342 | NADH dehydrogenase I chain C | No homolog |
| BCAL2343 | 2.629 | 9E-04 | BCAL2343 | NADH dehydrogenase I chain B | Bcen2424_2248 |
| BCAL2344 | 3.532 | 9E-04 | BCAL2344 | NADH dehydrogenase I chain A | Bcen2424_2249 |
| BCAL2428 | 3.355 | 2E-03 | BCAL2428 | putative cytochrome C precursor-related protein* | Bcen2424_2330 |
| BCAL2429 | 2.398 | 7E-04 | BCAL2429 | putative cytochrome C precursor-related protein* | Bcen2424_2331 |
| BCAL2458 | 12.12 | 2E-05 | BCAL2458 | rubredoxin | Bcen2424_2363 |
| BCAL2485 | 2.927 | 2E-05 | BCAL2485 | putative iron-sulphur cluster binding electron transport protein | No homolog |
| BCAL2486 | 3.805 | 5E-18 | BCAL2486 | putative iron-sulphur oxidoreductase | No homolog |
| BCAL2487 | 3.414 | 2E-07 | BCAL2487 | putative D-lactate dehydrogenase | No homolog |
| BCAL2817 | 2.183 | 1E-04 | BCAL2817 | S-(hydroxymethyl)glutathione dehydrogenase | Bcen2424_2604 |
| BCAL2908 | 5.222 | 3E-07 | BCAL2908 | fumarate hydratase class II | Bcen2424_1090 |
| BCAL3287 | 2.546 | 2E-06 | BCAL3287 | putative FAD-binding oxidase | Bcen2424_0735 |
| BCAL3290 | 5.106 | 4E-08 | BCAL3290 | putative glycolate oxidase iron-sulfur subunit | Bcen2424_0732 |
| BCAL3312 | 4.879 | 3E-05 | BCAL3312 | putative cytochrome b-561 membrane protein* | Bcen2424_0714 |
| BCAL3325 | 2.292 | 6E-04 | BCAL3325 | NAD(P) transhydrogenase subunit beta | Bcen2424_0702 |
| BCAL3326 | 2.934 | 2E-05 | BCAL3326 | NAD(P) transhydrogenase subunit alpha | Bcen2424_0701 |
| BCAL3395 | 2.358 | 7E-03 | BCAL3395 | NADP-dependent malic enzyme | Bcen2424_0631 |
| BCAM0961 | 2.107 | 7E-03 | BCAM0961 | aconitate hydratase | Bcen2424_3928 |
| BCAM0967 | 3.035 | 4E-04 | BCAM0967 | putative succinate dehydrogenase cytochrome b556 subunit | No homolog |
| BCAM0968 | 3.8 | 7E-05 | BCAM0968 | putative succinate dehydrogenase hydrophobic membrane anchor protein | Bcen2424_3935 |
| BCAM0969 | 3.487 | 3E-04 | BCAM0969 | succinate dehydrogenase flavoprotein subunit | Bcen2424_3936 |
| BCAM0970 | 3.686 | 3E-04 | BCAM0970 | succinate dehydrogenase iron-sulfur protein | Bcen2424_3937 |
| BCAM0972 | 3.101 | 2E-03 | BCAM0972 | citrate synthase | Bcen2424_3939 |
| BCAM1250 | 2.432 | 8E-03 | BCAM1250 | probable acetyl-CoA hydrolase/transferase* | Bcen2424_4126 |
| BCAM1756 | 3.024 | 8E-04 | BCAM1756 | putative molybdopterin oxidoreductase | Bcen2424_4579 |
| BCAM1833 | 8.188 | 2E-03 | BCAM1833 | aconitate hydratase | Bcen2424_4695 |
| BCAM1954 | 3.481 | 1E-03 | BCAM1954 | sodium:dicarboxylate symporter* | No homolog |
| BCAM2277 | 2.355 | 2E-06 | BCAM2277 | putative FMN-dependent dehydrogenase | Bcen2424_5034 |
| BCAS0259 | 2.363 | 4E-05 | BCAS0259 | putative sodium:dicarboxylate symporter family protein* | No homolog |
|  |  |  |  | ***Carbohydrate transport and metabolism (G)*** |  |
| BCAL0282 | 3.593 | 4E-04 | BCAL0282 | putative ABC transporter extracellular solute-binding protein* | Bcen2424_0395 |
| BCAL0284 | 5.432 | 4E-14 | BCAL0284 | putative ABC transporter permease | No homolog |
| BCAL0285 | 2.414 | 2E-04 | BCAL0285 | ABC transporter ATP-binding protein | No homolog |
| BCAL0308 | 5.379 | 1E-04 | BCAL0308 | ABC-2 type transporter, membrane protein | Bcen2424_0421 |
| BCAL0782 | 2.039 | 1E-09 | BCAL0782 | putative chitobiase* | Bcen2424_2820 |
| BCAL0856 | 2.13 | 1E-05 | BCAL0856 | putative aldolase | Bcen2424_2751 |
| BCAL1035 | 2.588 | 2E-03 | BCAL1035 | putative trehalose-phosphatase | Bcen2424_1157 |
| BCAL1181 | 2.242 | 6E-03 | BCAL1181 | putative glycerate kinase | No homolog |
| BCAL1252 | 8.088 | 1E-04 | BCAL1252 | putative proline/betaine transporter | Bcen2424_1281 |
| BCAL1259 | 2.157 | 1E-03 | BCAL1259 | Major Facilitator Superfamily protein | No homolog |
| BCAL1510 | 3.211 | 4E-05 | BCAL1510 | putative multidrug resistance transporter protein | Bcen2424_1503 |
| BCAL1838 | 4.543 | 1E-06 | BCAL1838 | UDP glycosyltransferase | Bcen2424_1765 |
| BCAL1935 | 3.516 | 8E-04 | BCAL1935 | polysaccharide deacetylase | No homolog |
| BCAL1990 | 4.391 | 6E-19 | BCAL1990 | glucose-6-phosphate isomerase | Bcen2424_1917 |
| BCAL2179 | 2.376 | 8E-11 | BCAL2179 | enolase | Bcen2424_2107 |
| BCAL2346 | 2.789 | 2E-03 | BCAL2346 | triosephosphate isomerase | Bcen2424_2251 |
| BCAL2399 | 2.674 | 2E-04 | BCAL2399 | Major Facilitator Superfamily protein* | Bcen2424_2304 |
| BCAL2470 | 3.997 | 1E-04 | BCAL2470 | Major Facilitator Superfamily protein | No homolog |
| BCAL2625 | 2.174 | 2E-03 | BCAL2625 | Major Facilitator Superfamily protein | Bcen2424_2420 |
| BCAL2661 | 2.402 | 1E-13 | BCAL2661 | phosphoglycerate mutase family | Bcen2424_2451 |
| BCAL2799 | 3.102 | 1E-05 | BCAL2799 | putative carbohydrate kinase | Bcen2424_2586 |
| BCAL2840 | 3.639 | 2E-06 | BCAL2840 | putative pyruvate kinase II protein | Bcen2424_2627 |
| BCAL2841 | 2.833 | 1E-05 | BCAL2841 | phosphoglycerate kinase | No homolog |
| BCAL2947 | 4.496 | 3E-05 | BCAL2947 | hypothetical protein | No homolog |
| BCAL3020 | 5.242 | 2E-03 | BCAL3020 | Major Facilitator Superfamily protein* | Bcen2424_0991 |
| BCAL3038 | 5.202 | 3E-05 | BCAL3038 | ABC transporter ATP-binding component | Bcen2424_0971 |
| BCAL3039 | 5.468 | 2E-04 | BCAL3039 | ABC transporter, membrane permease* | Bcen2424_0970 |
| BCAL3040 | 5.767 | 2E-04 | BCAL3040 | ABC transporter, membrane permease | Bcen2424_0969 |
| BCAL3130 | 4.195 | 7E-04 | BCAL3130 | ABC transporter ATP-binding protein | No homolog |
| BCAL3131 | 2.935 | 3E-04 | BCAL3131 | putative ABC transporter, membrane permease | No homolog |
| BCAL3160 | 3.523 | 4E-07 | BCAL3160 | Major Facilitator Superfamily protein | Bcen2424_0845 |
| BCAL3240 | 2.725 | 1E-03 | BCAL3240 | putative capsular polysaccharide transporter ATP-binding protein | No homolog |
| BCAL3241 | 2.501 | 5E-05 | BCAL3241 | putative capsular polysaccharide export protein, ABC transporter membrane protein | No homolog |
| BCAL3342 | 3.325 | 2E-04 | BCAL3342 | putative phosphoglycerate mutase | Bcen2424_0685 |
| BCAL3365 | 2.482 | 5E-05 | BCAL3365 | putative gluconate permease | Bcen2424_0662 |
| BCAL3388 | 2.303 | 4E-04 | BCAL3388 | glyceraldehyde 3-phosphate dehydrogenase 1 | Bcen2424_0639 |
| BCAL3425 | 2.995 | 8E-05 | BCAL3425 | putative sugar kinase | Bcen2424_0600 |
| BCAM1431 | 2.108 | 4E-17 | BCAM1431 | Major Facilitator Superfamily protein | Bcen2424_4304 |
| BCAM1741 | 2.548 | 8E-04 | BCAM1741 | Major Facilitator Superfamily protein* | Bcen2424_4560 |
| BCAM1760 | 3.243 | 2E-04 | BCAM1760 | putative multidrug resistance transporter protein | Bcen2424_4583 |
| BCAM2397 | 3.191 | 4E-04 | BCAM2397 | putative L-fuculose phosphate aldolase | Bcen2424_5157 |
| BCAM2464 | 2.567 | 1E-04 | BCAM2464 | Major Facilitator Superfamily protein | No homolog |
| BCAM2491 | 2.117 | 4E-08 | BCAM2491 | Major Facilitator Superfamily protein | No homolog |
| BCAM2665 | 3.503 | 1E-03 | BCAM2665 | Major Facilitator Superfamily protein | No homolog |
| BCAM2716 | 2.719 | 2E-04 | BCAM2716 | putative citrate transporter* | Bcen2424_5530 |
| BCAS0706 | 6.009 | 3E-04 | BCAS0706 | Major Facilitator Superfamily protein | No homolog |
|  |  |  |  | ***Amino acid transport and metabolism (E)*** |  |
| BCAL0021 | 2.253 | 2E-05 | BCAL0021 | putative branched-chain amino acid ABC transporter permease | Bcen2424_0090 |
| BCAL0022 | 2.903 | 7E-03 | BCAL0022 | putative branched-chain amino acid ABC transporter ATP-binding membrane protein* | Bcen2424_0091 |
| BCAL0023 | 2.583 | 1E-04 | BCAL0023 | putative branched-chain amino acid ABC transporter ATP-binding protein | No homolog |
| BCAL0039 | 2.176 | 5E-08 | BCAL0039 | periplasmic cyclohexadienyl dehydratase* | Bcen2424_0110 |
| BCAL0071 | 2.939 | 1E-09 | BCAL0071 | L-serine dehydratase I | Bcen2424_0142 |
| BCAL0073 | 2.634 | 6E-05 | BCAL0073 | glycine dehydrogenase (decarboxylating) | Bcen2424_0144 |
| BCAL0074 | 2.932 | 2E-03 | BCAL0074 | glycine cleavage system H protein | Bcen2424_0145 |
| BCAL0147 | 10.31 | 7E-05 | BCAL0147 | 5,10-methylenetetrahydrofolate reductase | Bcen2424_0274 |
| BCAL0207 | 38.99 | 1E-05 | BCAL0207 | 4-hydroxyphenylpyruvic acid dioxygenase | Bcen2424_0321 |
| BCAL0280 | 2.041 | 5E-04 | BCAL0280 | 3-dehydroquinate synthase | Bcen2424_0393 |
| BCAL0291 | 3.657 | 2E-03 | BCAL0291 | sodium:amino acid symporter family protein | Bcen2424_0404 |
| BCAL0297 | 2.514 | 2E-03 | BCAL0297 | putative thiamine biosynthesis oxidoreductase ThiO | Bcen2424_0410 |
| BCAL0311 | 3.523 | 2E-16 | BCAL0311 | ATP phosphoribosyltransferase | Bcen2424_0424 |
| BCAL0313 | 2.778 | 2E-04 | BCAL0313 | histidinol-phosphate aminotransferase | Bcen2424_0426 |
| BCAL0316 | 2.041 | 2E-04 | BCAL0316 | imidazole glycerol phosphate synthase subunit HisH | Bcen2424_0429 |
| BCAL0358 | 6.296 | 2E-04 | BCAL0358 | metallo peptidase, family M1* | Bcen2424_0492 |
| BCAL0369 | 4.529 | 1E-05 | BCAL0369 | putative amino acid permease | Bcen2424_0504 |
| BCAL0377 | 2.512 | 3E-04 | BCAL0377 | metallo peptidase, subfamily M24B | Bcen2424_0514 |
| BCAL0493 | 3.718 | 4E-07 | BCAL0493 | putative homoserine O-acetyltransferase | Bcen2424_3099 |
| BCAL0496 | 3.04 | 8E-04 | BCAL0496 | putative acetylglutamate kinase | Bcen2424_3096 |
| BCAL0533 | 2.092 | 1E-02 | BCAL0533 | putative amino-acid transporter transmembrane protein | Bcen2424_3056 |
| BCAL0631 | 2.179 | 4E-06 | BCAL0631 | putative hydroxymethylglutaryl-CoA lyase | Bcen2424_2959 |
| BCAL0644 | 2.397 | 1E-03 | BCAL0644 | dihydrodipicolinate synthetase family protein | Bcen2424_2946 |
| BCAL0681 | 2.525 | 3E-06 | BCAL0681 | putative 5-methyltetrahydrofolate--homocysteine methyltransferase | No homolog |
| BCAL0821 | 2.621 | 8E-04 | BCAL0821 | LysE type translocator* | Bcen2424_2783 |
| BCAL0869 | 2.349 | 1E-06 | BCAL0869 | threonine dehydratase biosynthetic | No homolog |
| BCAL0902 | 2.493 | 2E-06 | BCAL0902 | putative hydrolase/phosphatase protein | Bcen2424_2703 |
| BCAL1055 | 4.895 | 2E-03 | BCAL1055 | histidine transport system permease protein | Bcen2424_1175 |
| BCAL1056 | 3.615 | 3E-03 | BCAL1056 | histidine transport system permease protein | Bcen2424_1176 |
| BCAL1057 | 8.683 | 2E-04 | BCAL1057 | histidine ABC transporter ATP-binding protein | Bcen2424_1177 |
| BCAL1059 | 2.98 | 2E-05 | BCAL1059 | succinylornithine transaminase | Bcen2424_1180 |
| BCAL1060 | 3.502 | 1E-04 | BCAL1060 | putative arginine N-succinyltransferase, alpha chain | Bcen2424_1181 |
| BCAL1061 | 3.776 | 8E-07 | BCAL1061 | putative arginine N-succinyltransferase, beta chain | Bcen2424_1182 |
| BCAL1063 | 5.278 | 1E-05 | BCAL1063 | succinylarginine dihydrolase | Bcen2424_1184 |
| BCAL1064 | 8.729 | 2E-05 | BCAL1064 | putative succinylglutamate desuccinylase | Bcen2424_1185 |
| BCAL1065 | 10.74 | 3E-08 | BCAL1065 | periplasmic solute-binding protein* | No homolog |
| BCAL1090 | 4.412 | 3E-04 | BCAL1090 | ABC transporter ATP-binding protein | Bcen2424_1208 |
| BCAL1094 | 2.627 | 4E-06 | BCAL1094 | putative amino acid transport system, membrane protein* | Bcen2424_1212 |
| BCAL1260 | 5.186 | 2E-05 | BCAL1260 | carbamoyl-phosphate synthase small chain | Bcen2424_1290 |
| BCAL1261 | 4.115 | 9E-04 | BCAL1261 | putative transport-related membrane protein | Bcen2424_1291 |
| BCAL1262 | 5.369 | 2E-05 | BCAL1262 | carbamoyl-phosphate synthase large chain | Bcen2424_1292 |
| BCAL1421 | 2.063 | 2E-04 | BCAL1421 | putative branched amino acid transport system, membrane protein | Bcen2424_1414 |
| BCAL1822 | 3.889 | 1E-03 | BCAL1822 | putrescine transport system permease protein | Bcen2424_1749 |
| BCAL1823 | 2.508 | 8E-04 | BCAL1823 | putrescine ABC transporter ATP-binding protein | No homolog |
| BCAL1839 | 4.297 | 1E-03 | BCAL1839 | putative asparagine synthase | No homolog |
| BCAL1874 | 2.558 | 6E-04 | BCAL1874 | putative ATP phosphoribosyltransferase | Bcen2424_1802 |
| BCAL1925 | 4.451 | 5E-04 | BCAL1925 | threonine synthase | Bcen2424_1853 |
| BCAL1926 | 3.652 | 1E-04 | BCAL1926 | homoserine dehydrogenase | No homolog |
| BCAL1988 | 2.56 | 1E-04 | BCAL1988 | putative D-amino acid dehydrogenase small subunit | No homolog |
| BCAL2146 | 2.968 | 7E-05 | BCAL2146 | aspartokinase | Bcen2424_2074 |
| BCAL2317 | 5.671 | 9E-07 | BCAL2317 | family M14 unassigned peptidase | Bcen2424_2222 |
| BCAL2357 | 2.199 | 6E-04 | BCAL2357 | ketol-acid reductoisomerase | Bcen2424_2263 |
| BCAL2358 | 2.098 | 1E-03 | BCAL2358 | acetolactate synthase isozyme III small subunit | Bcen2424_2264 |
| BCAL2437 | 3.801 | 2E-04 | BCAL2437 | hypothetical protein | Bcen2424_2339 |
| BCAL2641 | 2.119 | 2E-10 | BCAL2641 | putative ornithine decarboxylase | Bcen2424_2432 |
| BCAL2659 | 2.614 | 1E-03 | BCAL2659 | putative cobalamin biosynthesis aminotransferase protein | Bcen2424_2449 |
| BCAL2729 | 2.177 | 6E-03 | BCAL2729 | putative amino acid permease | Bcen2424_2517 |
| BCAL2844 | 2.115 | 1E-03 | BCAL2844 | putative branched-chain amino acid aminotransferase IlvE | Bcen2424_2631 |
| BCAL2933 | 6.762 | 1E-04 | BCAL2933 | D-amino acid dehydrogenase small subunit* | Bcen2424_1064 |
| BCAL2942 | 2.797 | 8E-06 | BCAL2942 | cysteine synthase | Bcen2424_1055 |
| BCAL2952 | 2.493 | 1E-03 | BCAL2952 | 3-phosphoshikimate 1-carboxyvinyltransferase | Bcen2424_1045 |
| BCAL2954 | 2.069 | 3E-04 | BCAL2954 | P-protein (bifunctional includes: chorismate mutase and prephenate dehydratase) | Bcen2424_1043 |
| BCAL3056 | 4.384 | 1E-04 | BCAL3056 | putative aminotransferase | No homolog |
| BCAL3197 | 2.202 | 2E-05 | BCAL3197 | serine hydroxymethyltransferase | Bcen2424_0806 |
| BCAL3263 | 2.611 | 3E-05 | BCAL3263 | hypothetical protein | Bcen2424_0759 |
| BCAL3330 | 6.049 | 3E-05 | BCAL3330 | putative glutamate synthase | No homolog |
| BCAL3356 | 3.021 | 7E-03 | BCAL3356 | glutamate/aspartate transport system permease protein | Bcen2424_0671 |
| BCAL3358 | 2.036 | 8E-03 | BCAL3358 | periplasmic glutamate/aspartate-binding protein* | Bcen2424_0669 |
| BCAL3359 | 7.676 | 1E-03 | BCAL3359 | putative glutamate dehydrogenase | Bcen2424_0668 |
| BCAL3376 | 3.992 | 3E-23 | BCAL3376 | dihydrodipicolinate reductase | Bcen2424_0651 |
| BCAL3419 | 2.074 | 4E-03 | BCAL3419 | 3-dehydroquinate dehydratase | Bcen2424_0606 |
| BCAL3452 | 2.431 | 3E-03 | BCAL3452 | arginine biosynthesis bifunctional protein ArgJ | Bcen2424_0570 |
| BCAM0187 | 2.963 | 2E-03 | BCAM0187 | putative 2-isopropylmalate synthase | Bcen2424_3179 |
| BCAM0368 | 3.023 | 1E-03 | BCAM0368 | putative branched-chain amino acid transport protein | Bcen2424_3356 |
| BCAM0547 | 2.303 | 1E-03 | BCAM0547 | putative LysE type translocator | No homolog |
| BCAM0721 | 8.481 | 2E-10 | BCAM0721 | O-acetylhomoserine (thiol)-lyase | Bcen2424_3688 |
| BCAM1111 | 3.385 | 8E-18 | BCAM1111 | ornithine decarboxylase | No homolog |
| BCAM1112 | 3.974 | 1E-06 | BCAM1112 | biodegradative arginine decarboxylase | No homolog |
| BCAM1113 | 3.554 | 4E-11 | BCAM1113 | putrescine transport protein | No homolog |
| BCAM1243 | 2.917 | 1E-03 | BCAM1243 | putative aminotransferase | No homolog |
| BCAM1262 | 2.952 | 1E-04 | BCAM1262 | dihydroxyacid dehydratase | No homolog |
| BCAM1416 | 6.853 | 3E-04 | BCAM1416 | LysE-family transportery* | Bcen2424_4281 |
| BCAM1798 | 2.398 | 8E-05 | BCAM1798 | putative oxidoreductase | Bcen2424_4659 |
| BCAM2000 | 3.416 | 1E-04 | BCAM2000 | hypothetical protein | Bcen2424_4800 |
| BCAM2082 | 4.435 | 3E-05 | BCAM2082 | putative S-adenosylmethionine decarboxylase proenzyme | Bcen2424_4889 |
| BCAM2176 | 2.799 | 3E-07 | BCAM2176 | putative thermolabile glutaminase | Bcen2424_4940 |
| BCAM2234 | 2.057 | 1E-02 | BCAM2234 | putative pyochelin biosynthetic protein PchB | Bcen2424_5002 |
| BCAM2247 | 3.282 | 5E-04 | BCAM2247 | putative amino acid ABC transporter ATP-binding protein | No homolog |
| BCAM2248 | 2.666 | 1E-05 | BCAM2248 | putative amino acid ABC transporter ATP-binding protein* | No homolog |
| BCAM2249 | 4.622 | 6E-05 | BCAM2249 | putative amino acid transport system permease component of ABC transporter protein | Bcen2424_5017 |
| BCAM2250 | 3.573 | 6E-04 | BCAM2250 | putative amino acid transport system permease component of ABC transporter protein | No homolog |
| BCAM2251 | 2.7 | 7E-04 | BCAM2251 | putative amino acid solute binding component of ABC transporter* | Bcen2424_5019 |
| BCAM2479 | 3.538 | 5E-04 | BCAM2479 | putative transporter-LysE family* | Bcen2424_5281 |
| BCAM2771 | 2.159 | 3E-03 | BCAM2771 | putative dihydrodipicolinate synthetase | Bcen2424_5613 |
| BCAS0060 | 2.696 | 9E-05 | BCAS0060 | extracellular amino acid-binding protein* | No homolog |
| BCAS0128 | 3.708 | 7E-05 | BCAS0128 | ABC transporter ATP-binding protein | Bcen2424_6102 |
| BCAS0129 | 5.17 | 2E-06 | BCAS0129 | putative binding-protein-dependent transport system component | Bcen2424_6101 |
| BCAS0131 | 2.689 | 3E-03 | BCAS0131 | putative binding-protein-dependent transport system component* | No homolog |
|  |  |  |  | ***Nucleotide transport and metabolism (F)*** |  |
| BCAL0004 | 2.735 | 1E-03 | BCAL0004 | cysteine peptidase, family C26 | Bcen2424_0071 |
| BCAL0204 | 2.873 | 4E-04 | BCAL0204 | orotate phosphoribosyltransferase | Bcen2424_0317 |
| BCAL0281 | 2.453 | 2E-06 | BCAL0281 | putative phosphohydrolase | Bcen2424_0394 |
| BCAL0299 | 3.32 | 5E-04 | BCAL0299 | thiazole biosynthesis protein ThiG | Bcen2424_0412 |
| BCAL0800 | 4.592 | 1E-04 | BCAL0800 | ribose-phosphate pyrophosphokinase | Bcen2424_2802 |
| BCAL0820 | 2.675 | 1E-04 | BCAL0820 | putative adenine phosphoribosyltransferase | Bcen2424_2784 |
| BCAL0823 | 5.436 | 2E-05 | BCAL0823 | putative formyltetrahydrofolate deformylase | Bcen2424_2781 |
| BCAL1450 | 2.873 | 1E-03 | BCAL1450 | putative nucleosidase | Bcen2424_1443 |
| BCAL1701 | 16.01 | 7E-04 | BCAL1701 | ornibactin synthetase F | No homolog |
| BCAL1887 | 2.445 | 3E-07 | BCAL1887 | nucleoside diphosphate kinase | Bcen2424_1815 |
| BCAL2089 | 3.678 | 7E-08 | BCAL2089 | uridylate kinase | Bcen2424_2017 |
| BCAL2181 | 2.017 | 9E-16 | BCAL2181 | CTP synthase | Bcen2424_2109 |
| BCAL3139 | 2.127 | 3E-04 | BCAL3139 | aspartate carbamoyltransferase | Bcen2424_0867 |
| BCAM1099 | 2.559 | 2E-06 | BCAM1099 | putative permease | Bcen2424_4002 |
| BCAM2556 | 2.177 | 8E-03 | BCAM2556 | putative purine nucleoside permease* | Bcen2424_5356 |
| BCAM2830 | 3.157 | 4E-04 | BCAM2830 | hypothetical protein | Bcen2424_5678 |
| BCAS0337 | 2.587 | 3E-03 | BCAS0337 | putative lipoprotein* | Bcen2424_6758 |
|  |  |  |  | ***Coenzyme transport and metabolism (H)*** |  |
| BCAL0145 | 7.516 | 3E-04 | BCAL0145 | adenosylhomocysteinase | Bcen2424_0272 |
| BCAL0298 | 2.549 | 2E-04 | BCAL0298 | thiamine biosynthesis protein ThiS | Bcen2424_0411 |
| BCAL0300 | 6.848 | 1E-03 | BCAL0300 | thiamine-phosphate pyrophosphorylase | Bcen2424_0413 |
| BCAL0404 | 10.08 | 1E-04 | BCAL0404 | phenylacetate-coenzyme A ligase | Bcen2424_0539 |
| BCAL0439 | 2.122 | 1E-07 | BCAL0439 | glutamate--cysteine ligase | Bcen2424_3149 |
| BCAL0747 | 3.398 | 1E-03 | BCAL0747 | putative methyltransferase | Bcen2424_2849 |
| BCAL0896 | 3.644 | 4E-04 | BCAL0896 | 4-hydroxythreonine-4-phosphate dehydrogenase | Bcen2424_2709 |
| BCAL0915 | 2.111 | 1E-02 | BCAL0915 | putative 3,4-dihydroxy-2-butanone 4-phosphate synthase | No homolog |
| BCAL1104 | 8.827 | 6E-05 | BCAL1104 | thiamine biosynthesis protein | Bcen2424_1223 |
| BCAL1229 | 2.519 | 2E-07 | BCAL1229 | putative lipoprotein* | Bcen2424_1258 |
| BCAL1705 | 5.043 | 7E-04 | BCAL1705 | cobyrinic acid A,C-diamide synthase | Bcen2424_1651 |
| BCAL1706 | 5.761 | 1E-05 | BCAL1706 | cobalamin adenosyltransferase | Bcen2424_1652 |
| BCAL1708 | 2.142 | 2E-04 | BCAL1708 | uroporphyrin-III C-methyltransferase | No homolog |
| BCAL1711 | 5.608 | 6E-05 | BCAL1711 | putative cobalamin biosynthesis-related protein | No homolog |
| BCAL1712 | 4.8 | 2E-06 | BCAL1712 | putative magnesium chelatase protein | Bcen2424_1658 |
| BCAL1723 | 2.678 | 3E-07 | BCAL1723 | precorrin-3b C17-methyltransferase | Bcen2424_1671 |
| BCAL1724 | 2.782 | 4E-05 | BCAL1724 | precorrin-2 C20-methyltransferase | Bcen2424_1672 |
| BCAL1725 | 3.722 | 3E-04 | BCAL1725 | precorrin-8X methylmutase | Bcen2424_1673 |
| BCAL1730 | 2.681 | 1E-04 | BCAL1730 | precorrin-4 C11-methyltransferase | Bcen2424_1678 |
| BCAL1840 | 3.384 | 1E-21 | BCAL1840 | hypothetical protein | Bcen2424_1767 |
| BCAL1859 | 3.157 | 4E-05 | BCAL1859 | hypothetical protein* | No homolog |
| BCAL2390 | 2.971 | 6E-05 | BCAL2390 | coproporphyrinogen III oxidase, aerobic | No homolog |
| BCAL2391 | 4.366 | 9E-04 | BCAL2391 | putative nicotinate-nucleotide adenylyltransferase | Bcen2424_2297 |
| BCAL2441 | 2.856 | 2E-03 | BCAL2441 | chorismate lyase | Bcen2424_2346 |
| BCAL2651 | 3.341 | 2E-03 | BCAL2651 | pantoate--beta-alanine ligase | Bcen2424_2442 |
| BCAL2652 | 2.633 | 9E-07 | BCAL2652 | putative aspartate 1-decarboxylase | Bcen2424_2443 |
| BCAL2656 | 2.027 | 9E-03 | BCAL2656 | putative cobyric acid synthase protein | No homolog |
| BCAL2662 | 2.472 | 5E-04 | BCAL2662 | putative cobalamin [5'-phosphate] synthase | Bcen2424_2452 |
| BCAL2663 | 2.955 | 3E-04 | BCAL2663 | putative nicotinate-nucleotide--dimethylbenzimidazole phosphoribosyltransferase | Bcen2424_2453 |
| BCAL2700 | 2.191 | 5E-11 | BCAL2700 | putative oxidoreductase | Bcen2424_2490 |
| BCAL2717 | 2.492 | 3E-04 | BCAL2717 | putative nicotinate-nucleotide pyrophosphorylase | No homolog |
| BCAL2915 | 2.018 | 1E-06 | BCAL2915 | dihydrofolate reductase | Bcen2424_1082 |
| BCAL3016 | 3.014 | 1E-04 | BCAL3016 | putative coproporphyrinogen III oxidase family protein | No homolog |
| BCAL3054 | 2.245 | 1E-05 | BCAL3054 | 6,7-dimethyl-8-ribityllumazine synthase | Bcen2424_0956 |
| BCAL3267 | 2.384 | 6E-06 | BCAL3267 | putative 3-methyl-2-oxobutanoate hydroxymethyltransferase | Bcen2424_0755 |
| BCAL3448 | 2.88 | 1E-04 | BCAL3448 | dephospho-CoA kinase | Bcen2424_0575 |
| BCAM0707 | 2.354 | 2E-04 | BCAM0707 | putative nicotinamide mononucleotide transporter | No homolog |
| BCAM0829 | 2.075 | 1E-03 | BCAM0829 | putative riboflavin synthase alpha chain | No homolog |
| BCAM0882 | 2.462 | 5E-03 | BCAM0882 | hypothetical protein | No homolog |
| BCAM1593 | 3.071 | 5E-04 | BCAM1593 | vitamin B12 transporter BtuB precursor* | Bcen2424_4452 |
| BCAM1711 | 8.85 | 6E-04 | BCAM1711 | phenylacetate-coenzyme A ligase | Bcen2424_4529 |
| BCAM2080 | 2.085 | 7E-03 | BCAM2080 | putative bifunctional NMN adenylyltransferase/NUDIX hydrolase | Bcen2424_4887 |
| BCAM2565 | 2.171 | 1E-04 | BCAM2565 | putative methyltransferase | Bcen2424_5365 |
| BCAM2832 | 2.107 | 2E-03 | BCAM2832 | putative flavin containing amine oxidase | No homolog |
|  |  |  |  | ***Lipid transport and metabolism (I)*** |  |
| BCAL0406 | 4.238 | 3E-03 | BCAL0406 | probable enoyl-CoA hydratase PaaG | Bcen2424_0541 |
| BCAL0407 | 2.131 | 2E-03 | BCAL0407 | beta-ketoadipyl CoA thiolase | Bcen2424_0542 |
| BCAL0882 | 3.198 | 7E-17 | BCAL0882 | putative phospholipase D | Bcen2424_2723 |
| BCAL0901 | 2.411 | 6E-05 | BCAL0901 | putative acyltransferase* | Bcen2424_2704 |
| BCAL1384 | 2.747 | 3E-04 | BCAL1384 | putative acyltransferase | Bcen2424_1373 |
| BCAL1386 | 2.323 | 1E-04 | BCAL1386 | putative hydrolase | Bcen2424_1375 |
| BCAL1466 | 2.948 | 1E-03 | BCAL1466 | Major Facilitator Superfamily protein | Bcen2424_1459 |
| BCAL1473 | 3.11 | 9E-04 | BCAL1473 | succinyl-CoA:3-ketoacid-coenzyme A transferase subunit B | No homolog |
| BCAL1636 | 2.985 | 9E-04 | BCAL1636 | putative exported endonuclease* | Bcen2424_1585 |
| BCAL1720 | 2.039 | 1E-03 | BCAL1720 | putative acyl-CoA dehydrogenase | Bcen2424_1668 |
| BCAL1977 | 2.009 | 4E-04 | BCAL1977 | putative thiolase | Bcen2424_1904 |
| BCAL2007 | 2.528 | 2E-04 | BCAL2007 | squalene/phytoene synthase family protein | Bcen2424_1934 |
| BCAL2080 | 3.836 | 7E-06 | BCAL2080 | (3R)-hydroxymyristoyl-[acyl carrier protein] dehydratase | Bcen2424_2008 |
| BCAL2086 | 3.257 | 7E-04 | BCAL2086 | putative phosphatidate cytidylyltransferase membrane protein* | Bcen2424_2014 |
| BCAL2087 | 3.032 | 1E-04 | BCAL2087 | undecaprenyl pyrophosphate synthetase | Bcen2424_2015 |
| BCAL2148 | 2.8 | 3E-05 | BCAL2148 | acetyl-coenzyme A carboxylase carboxyl transferase subunit alpha | Bcen2424_2076 |
| BCAL2325 | 2.178 | 2E-03 | BCAL2325 | hypothetical protein | Bcen2424_2230 |
| BCAL2355 | 6.337 | 1E-12 | BCAL2355 | putative phosphatidyltransferase | Bcen2424_2261 |
| BCAL2356 | 3.235 | 5E-04 | BCAL2356 | putative decarboxylase | Bcen2424_2262 |
| BCAL2834 | 4.771 | 2E-05 | BCAL2834 | putative acylhydrolase* | Bcen2424_2621 |
| BCAL3420 | 2.074 | 1E-03 | BCAL3420 | biotin carboxyl carrier protein of acetyl-CoA carboxylase | No homolog |
| BCAL3421 | 2.685 | 7E-03 | BCAL3421 | biotin carboxylase | Bcen2424_0604 |
| BCAM0149 | 2.687 | 9E-04 | BCAM0149 | putative phospholipase D protein | No homolog |
| BCAM1423 | 2.1 | 8E-04 | BCAM1423 | putative AMP-binding enzyme | Bcen2424_4295 |
| BCAM1459 | 2.392 | 2E-06 | BCAM1459 | short-chain fatty acid transporter | No homolog |
| BCAM1710 | 5.162 | 1E-03 | BCAM1710 | putative enoyl-CoA hydratase/isomerase | Bcen2424_4528 |
| BCAM2395 | 2.912 | 8E-04 | BCAM2395 | putative dehydrogenase/oxidoreductase protein | No homolog |
| BCAM2430 | 9.292 | 9E-04 | BCAM2430 | putative biotin carboxylase | Bcen2424_5237 |
| BCAM2431 | 4.702 | 1E-03 | BCAM2431 | putative enoyl coenzyme A hydratase-like protein | Bcen2424_5238 |
| BCAM2432 | 6.443 | 1E-03 | BCAM2432 | putative biotin-dependent carboxyl transferase | Bcen2424_5239 |
| BCAM2433 | 9.231 | 7E-04 | BCAM2433 | putative acyl-CoA dehydrogenase | Bcen2424_5240 |
| BCAM2831 | 3.341 | 1E-14 | BCAM2831 | squalene--hopene cyclase | Bcen2424_5679 |
| BCAS0167 | 3.716 | 5E-09 | BCAS0167 | squalene-hopene cyclase | Bcen2424_6062 |
| BCAS0504 | 3.053 | 3E-05 | BCAS0504 | putative phage transmembrane acetyltransferase | No homolog |
|  |  |  |  | ***Inorganic ion transport and metabolism (P)*** |  |
| BCAL0028 | 2.264 | 5E-04 | BCAL0028 | putative citrate transporter protein | Bcen2424_0097 |
| BCAL0606 | 2.478 | 2E-03 | BCAL0606 | putative transport related, membrane protein | No homolog |
| BCAL0645 | 3.541 | 8E-04 | BCAL0645 | sulfate-binding protein precursor* | Bcen2424_2945 |
| BCAL0763 | 3.938 | 2E-03 | BCAL0763 | hypothetical protein* | Bcen2424_2833 |
| BCAL0908 | 2.08 | 1E-04 | BCAL0908 | ChaC-like protein | Bcen2424_2696 |
| BCAL1091 | 28.5 | 3E-05 | BCAL1091 | ABC transporter membrane protein | Bcen2424_1209 |
| BCAL1092 | 20.72 | 8E-04 | BCAL1092 | ABC transporter extracellular solute-binding protein* | Bcen2424_1210 |
| BCAL1270 | 2.991 | 5E-13 | BCAL1270 | phosphate transport system, substrate-binding exported periplasmic protein* | Bcen2424_1300 |
| BCAL1271 | 2.614 | 3E-03 | BCAL1271 | phosphate transport system permease protein | Bcen2424_1301 |
| BCAL1272 | 2.074 | 5E-03 | BCAL1272 | phosphate transport system permease protein* | Bcen2424_1302 |
| BCAL1345 | 2.549 | 3E-04 | BCAL1345 | putative TonB-dependent siderophore receptor* | No homolog |
| BCAL1346 | 2.014 | 3E-04 | BCAL1346 | putative Fe uptake system ATP-binding protein | Bcen2424_1348 |
| BCAL1691 | 9.754 | 4E-04 | BCAL1691 | putative iron transport-related ATP-binding protein | Bcen2424_1635 |
| BCAL1692 | 7.75 | 9E-04 | BCAL1692 | putative iron transport-related membrane protein* | Bcen2424_1636 |
| BCAL1694 | 10.55 | 7E-04 | BCAL1694 | putative iron transport-related exported protein | Bcen2424_1638 |
| BCAL1700 | 33.93 | 2E-04 | BCAL1700 | ornibactin receptor precursor* | Bcen2424_1644 |
| BCAL1726 | 3.684 | 1E-05 | BCAL1726 | putative oxidoreductase | Bcen2424_1674 |
| BCAL1871 | 3.455 | 2E-05 | BCAL1871 | kup system potassium uptake protein | Bcen2424_1799 |
| BCAL1939 | 2.875 | 2E-13 | BCAL1939 | putative integral membrane transport protein* | Bcen2424_1868 |
| BCAL1940 | 2.17 | 3E-04 | BCAL1940 | hypothetical protein | Bcen2424_1869 |
| BCAL2271 | 2.102 | 5E-06 | BCAL2271 | putative toxic anion resistance protein | No homolog |
| BCAL2281 | 3.395 | 2E-03 | BCAL2281 | putative ferrichrome receptor* | Bcen2424_2186 |
| BCAL2290 | 7.932 | 2E-04 | BCAL2290 | putative bacterioferritin ferredoxin protein | Bcen2424_2197 |
| BCAL2297 | 3.056 | 2E-02 | BCAL2297 | hypothetical protein* | Bcen2424_2204 |
| BCAL2299 | 7.073 | 2E-06 | BCAL2299 | putative permease protein | Bcen2424_2207 |
| BCAL2301 | 10.41 | 2E-04 | BCAL2301 | hypothetical protein* | Bcen2424_2209 |
| BCAL2352 | 3.665 | 8E-04 | BCAL2352 | putative carbonic anhydrase* | Bcen2424_2257 |
| BCAL2353 | 4.679 | 1E-03 | BCAL2353 | putative sulfate transporter | No homolog |
| BCAL2660 | 2.17 | 2E-02 | BCAL2660 | putaive vitamin B12 transport protein* | Bcen2424_2450 |
| BCAL2664 | 2.764 | 8E-05 | BCAL2664 | ABC transporter ATP-binding protein | Bcen2424_2454 |
| BCAL2936 | 2.695 | 5E-04 | BCAL2936 | hypothetical protein* | Bcen2424_1061 |
| BCAL2937 | 3.262 | 6E-04 | BCAL2937 | ABC transporter membrane permease | Bcen2424_1060 |
| BCAL2938 | 2.492 | 4E-03 | BCAL2938 | ABC transporter ATP-binding protein | Bcen2424_1059 |
| BCAL3097 | 2.287 | 2E-15 | BCAL3097 | hypothetical protein | Bcen2424_0911 |
| BCAL3444 | 2.056 | 1E-11 | BCAL3444 | hypothetical protein | Bcen2424_0579 |
| BCAM0836 | 2.034 | 1E-02 | BCAM0836 | putative manganese transport protein, NRAMP family | Bcen2424_3802 |
| BCAM0890 | 3.511 | 3E-04 | BCAM0890 | ABC transporter ATP-binding protein | Bcen2424_3858 |
| BCAM0891 | 3.63 | 8E-05 | BCAM0891 | putative molybdenum transport system permease | No homolog |
| BCAM0892 | 4.359 | 6E-05 | BCAM0892 | putative molybdate-binding periplasmic protein precursor* | No homolog |
| BCAM1187 | 7.714 | 2E-03 | BCAM1187 | TonB-dependent siderophore receptor* | No homolog |
| BCAM1359 | 5.094 | 5E-04 | BCAM1359 | putative efflux pump/antiporter | Bcen2424_4226 |
| BCAM1426 | 2.91 | 1E-03 | BCAM1426 | putative potassium channel protein | Bcen2424_4298 |
| BCAM1713 | 2.611 | 7E-03 | BCAM1713 | putative ionic antiporter* | Bcen2424_4531 |
| BCAM1958 | 2.23 | 4E-03 | BCAM1958 | ABC transporter, permease protein | Bcen2424_4757 |
| BCAM2007 | 4.825 | 7E-04 | BCAM2007 | TonB-dependent siderophore receptor* | Bcen2424_4808 |
| BCAM2507 | 3.912 | 1E-03 | BCAM2507 | Major Facilitator Superfamily protein | Bcen2424_5310 |
| BCAS0360 | 5.599 | 4E-04 | BCAS0360 | TonB-dependent receptor* | Bcen2424_6729 |
|  |  |  |  | ***Secondary metabolites biosynthesis, transport, and catabolism (Q)*** |  |
| BCAL0302 | 2.753 | 4E-07 | BCAL0302 | hypothetical protein | Bcen2424_0415 |
| BCAL0405 | 4.657 | 1E-04 | BCAL0405 | phenylacetic acid degradation protein PaaI | Bcen2424_0540 |
| BCAL1690 | 15.89 | 7E-05 | BCAL1690 | putative dioxygenase | Bcen2424_1634 |
| BCAL1695 | 4.79 | 3E-03 | BCAL1695 | ornibactin biosynthesis ABC transport protein | Bcen2424_1639 |
| BCAL1696 | 25.65 | 7E-04 | BCAL1696 | ornibactin biosynthesis non-ribosomal peptide synthase | Bcen2424_1640 |
| BCAL1697 | 7.963 | 4E-03 | BCAL1697 | ornibactin biosynthesis non-ribosomal peptide synthase | Bcen2424_1641 |
| BCAL1699 | 20.3 | 1E-04 | BCAL1699 | putative L-ornithine 5-monooxygenase | No homolog |
| BCAL2624 | 3.628 | 5E-03 | BCAL2624 | putative aromatic hydrocarbon catabolic dehydrogenase | Bcen2424_2419 |
| BCAL3159 | 2.634 | 6E-04 | BCAL3159 | hypothetical protein | Bcen2424_0846 |
| BCAL3183 | 2.746 | 1E-04 | BCAL3183 | putative hydrolase | No homolog |
| BCAL3229 | 2.995 | 9E-05 | BCAL3229 | hypothetical protein | No homolog |
| BCAM0195 | 2.64 | 8E-04 | BCAM0195 | putative non-ribosomal peptide synthetase | Bcen2424_3187 |
| BCAM1826 | 2.397 | 7E-08 | BCAM1826 | putative N-hydroxyarylamine O-acetyltransferase | Bcen2424_4687 |
| IG1_2194741 | 2.424 | 2E-04 | BCAL1991 | ABC transporter ATP-binding protein | No homolog |
|  |  |  |  | ***General function prediction only (R)*** |  |
| BCAL0109 | 2.222 | 3E-06 | BCAL0109 | putative TPR repeat protein | No homolog |
| BCAL0163 | 2.019 | 2E-03 | BCAL0163 | putative phospholipid-binding lipoprotein | No homolog |
| BCAL0380 | 3.339 | 1E-03 | BCAL0380 | ABC transporter ATP-binding subunit | No homolog |
| BCAL0490 | 3.39 | 4E-05 | BCAL0490 | peptidase, family M48* | Bcen2424_3102 |
| BCAL0495 | 3.839 | 4E-19 | BCAL0495 | haloacid dehalogenase-like hydrolase | Bcen2424_3097 |
| BCAL0676 | 3.259 | 1E-03 | BCAL0676 | putative short chain dehydrogenase | Bcen2424_2922 |
| BCAL0746 | 2.13 | 2E-12 | BCAL0746 | hypothetical protein | No homolog |
| BCAL0760 | 4.114 | 2E-05 | BCAL0760 | probable lipoprotein* | Bcen2424_2836 |
| BCAL0765 | 4.134 | 4E-04 | BCAL0765 | hypothetical protein* | Bcen2424_2831 |
| BCAL0766 | 5.226 | 2E-03 | BCAL0766 | putative branched-chain amino acid transport system permease protein | Bcen2424_2830 |
| BCAL0767 | 2.276 | 8E-04 | BCAL0767 | ABC transporter ATP-binding protein | Bcen2424_2829 |
| BCAL0771 | 2.438 | 3E-06 | BCAL0771 | non-heme chloroperoxidase | No homolog |
| BCAL0814 | 6.787 | 9E-08 | BCAL0814 | ABC transporter ATP-binding protein* | Bcen2424_2790 |
| BCAL0817 | 2.849 | 6E-04 | BCAL0817 | putative 3-deoxy-D-manno-octulosonate 8-phosphate phosphatase | Bcen2424_2787 |
| BCAL0876 | 2.238 | 3E-04 | BCAL0876 | putative ubiquinone biosynthesis protein | Bcen2424_2729 |
| BCAL0900 | 3.496 | 5E-06 | BCAL0900 | hypothetical protein | Bcen2424_2705 |
| BCAL0909 | 2.579 | 1E-04 | BCAL0909 | hypothetical protein | Bcen2424_2695 |
| BCAL0916 | 2.34 | 4E-04 | BCAL0916 | putative hydrolase protein | No homolog |
| BCAL1034 | 2.829 | 3E-04 | BCAL1034 | SCO1/SenC family protein | Bcen2424_1156 |
| BCAL1211 | 2.726 | 1E-03 | BCAL1211 | hypothetical protein | Bcen2424_1242 |
| BCAL1383 | 2.7 | 5E-04 | BCAL1383 | cytidylyltransferase family protein | Bcen2424_1372 |
| BCAL1518 | 9.963 | 2E-08 | BCAL1518 | AFG1-like ATPase | Bcen2424_1511 |
| BCAL1693 | 3.324 | 9E-03 | BCAL1693 | putative iron transport-related membrane protein* | Bcen2424_1637 |
| BCAL1703 | 6.277 | 1E-03 | BCAL1703 | metallo peptidase, subfamily M20D | Bcen2424_1649 |
| BCAL1710 | 9.575 | 9E-06 | BCAL1710 | putative cobalamin biosynthesis-related protein | Bcen2424_1656 |
| BCAL1722 | 2.232 | 1E-03 | BCAL1722 | putative exported chitinase* | Bcen2424_1670 |
| BCAL1841 | 3.299 | 3E-06 | BCAL1841 | hypothetical protein | Bcen2424_1768 |
| BCAL1843 | 2.042 | 4E-03 | BCAL1843 | ABC transporter ATP-binding protein | Bcen2424_1770 |
| BCAL1846 | 2.832 | 1E-16 | BCAL1846 | ABC transporter ATP-binding protein | No homolog |
| BCAL1878 | 2.387 | 1E-05 | BCAL1878 | putative GTP-binding protein | Bcen2424_1806 |
| BCAL1880 | 2.427 | 1E-04 | BCAL1880 | GTP-binding protein EngA | Bcen2424_1808 |
| BCAL1886 | 4.122 | 8E-21 | BCAL1886 | radical SAM superfamily protein | Bcen2424_1814 |
| BCAL1958 | 2.293 | 3E-04 | BCAL1958 | hypothetical protein | Bcen2424_1887 |
| BCAL2058 | 2.27 | 3E-06 | BCAL2058 | acetyltransferase (GNAT) family protein | No homolog |
| BCAL2165 | 2.864 | 1E-04 | BCAL2165 | metallo-beta-lactamase superfamily protein | Bcen2424_2093 |
| BCAL2170 | 2.019 | 4E-03 | BCAL2170 | metallo peptidase, subfamily M50B | Bcen2424_2098 |
| BCAL2368 | 2.098 | 4E-14 | BCAL2368 | putative decarboxylase | Bcen2424_2274 |
| BCAL2448 | 2.304 | 1E-07 | BCAL2448 | putative phenazine biosynthesis-like protein | Bcen2424_2353 |
| BCAL2456 | 2.748 | 2E-05 | BCAL2456 | ABC transporter ATP-binding protein | No homolog |
| BCAL2466 | 6.366 | 1E-06 | BCAL2466 | ecotin precursor | Bcen2424_2387 |
| BCAL2578 | 2.041 | 1E-03 | BCAL2578 | putative hydrolase | No homolog |
| BCAL2639 | 2.987 | 5E-04 | BCAL2639 | putative hydrolase | Bcen2424_2430 |
| BCAL2677 | 7.659 | 2E-06 | BCAL2677 | putative permease protein | Bcen2424_2467 |
| BCAL2678 | 4.739 | 5E-15 | BCAL2678 | putative permease protein | Bcen2424_2468 |
| BCAL2764 | 4.209 | 5E-06 | BCAL2764 | MviN-like protein | No homolog |
| BCAL2816 | 3.528 | 3E-03 | BCAL2816 | S-formylglutathione hydrolase | Bcen2424_2603 |
| BCAL3199 | 2.188 | 4E-16 | BCAL3199 | putative thioesterase | No homolog |
| BCAL3234 | 2.925 | 9E-04 | BCAL3234 | glycosyltransferase | No homolog |
| BCAL3315 | 2.514 | 4E-04 | BCAL3315 | mammalian cell entry related membrane protein | Bcen2424_0711 |
| BCAL3451 | 2.87 | 1E-04 | BCAL3451 | hypothetical protein | Bcen2424_0571 |
| BCAM0150 | 2.735 | 1E-03 | BCAM0150 | putative lipoprotein* | No homolog |
| BCAM0152 | 5.067 | 5E-06 | BCAM0152 | putative lipoprotein | No homolog |
| BCAM0883 | 3.125 | 4E-05 | BCAM0883 | putative ion transporter | Bcen2424_3851 |
| BCAM0938 | 2.209 | 5E-05 | BCAM0938 | acetyltransferase (GNAT) family protein | No homolog |
| BCAM0962 | 3.915 | 1E-04 | BCAM0962 | 2-methylcitrate dehydratase | Bcen2424_3929 |
| BCAM2008 | 2.535 | 1E-02 | BCAM2008 | hypothetical protein | Bcen2424_4809 |
| BCAM2086 | 3.555 | 4E-04 | BCAM2086 | putative spermidine synthase | No homolog |
| BCAM2492 | 2.159 | 4E-03 | BCAM2492 | hypothetical protein | Bcen2424_5294 |
| BCAM2536 | 5.039 | 2E-06 | BCAM2536 | putative alpha-beta hydrolase | Bcen2424_5346 |
| BCAM2566 | 2.082 | 9E-04 | BCAM2566 | putative GCN5-related N-acetyltransferase | No homolog |
| BCAM2744 | 2.518 | 2E-16 | BCAM2744 | haemolysin-III related protein | Bcen2424_5560 |
| BCAM2828 | 3.275 | 5E-04 | BCAM2828 | hypothetical protein* | Bcen2424_5676 |
| BCAS0109 | 2.058 | 1E-03 | BCAS0109 | succinylglutamate desuccinylase/aspartoacylase family protein | No homolog |
| BCAS0118 | 2.34 | 1E-02 | BCAS0118 | putative H-NS family DNA-binding protein | Bcen2424_6107 |
| BCAS0152 | 2.51 | 1E-02 | BCAS0152 | putative hydrolase | Bcen2424_6081 |
| BCAS0507 | 2.214 | 4E-12 | BCAS0507 | putative phage baseplate assembly protein gpJ | No homolog |
| BCAS0517 | 2.2 | 2E-03 | BCAS0517 | putative phage tail tube protein | No homolog |
| BCAS0518 | 3.241 | 5E-03 | BCAS0518 | putative phage tail sheath protein | No homolog |
| BCAS0722 | 2.495 | 9E-04 | BCAS0722 | putative patatin-like phospholipase | No homolog |
| IG1_2726212 | 2.363 | 7E-03 | BCAL2459 | putative O-methyltransferase | Bcen2424_2380 |
| IG2_990284 | 2.327 | 5E-04 | BCAM0903 | lysine decarboxylase family protein | Bcen2424_3871 |
| pBCA051 | 2.216 | 1E-03 | pBCA051 | LamB/YcsF family protein | No homolog |
|  |  |  |  | ***Function unknown (S)*** |  |
| BCAL0117 | 2.244 | 6E-03 | BCAL0117 | hypothetical protein* | Bcen2424_0244 |
| BCAL0146 | 6.8 | 1E-04 | BCAL0146 | hypothetical protein | Bcen2424_0273 |
| BCAL0179 | 4.248 | 1E-04 | BCAL0179 | hypothetical protein | No homolog |
| BCAL0338 | 3.103 | 6E-04 | BCAL0338 | putative type VI secretion system protein TssK | Bcen2424_0464 |
| BCAL0339 | 3.002 | 2E-03 | BCAL0339 | putative type VI secretion system protein TssJ* | Bcen2424_0465 |
| BCAL0342 | 2.427 | 1E-04 | BCAL0342 | putative type VI secretion system protein TssC | Bcen2424_0468 |
| BCAL0343 | 4.033 | 1E-03 | BCAL0343 | putative type VI secretion system protein TssD | Bcen2424_0469 |
| BCAL0344 | 2.643 | 3E-07 | BCAL0344 | putative type VI secretion system protein TssE | Bcen2424_0470 |
| BCAL0345 | 3.737 | 2E-07 | BCAL0345 | putative type VI secretion system protein TssF | Bcen2424_0471 |
| BCAL0350 | 5.911 | 1E-04 | BCAL0350 | hypothetical protein | Bcen2424_0484 |
| BCAL0351 | 5.779 | 9E-06 | BCAL0351 | putative type VI secretion system protein TssM | Bcen2424_0485 |
| BCAL0360 | 2.208 | 7E-04 | BCAL0360 | hypothetical protein | No homolog |
| BCAL0425 | 4.033 | 1E-04 | BCAL0425 | hypothetical protein* | Bcen2424_3164 |
| BCAL0531 | 6.461 | 4E-04 | BCAL0531 | hypothetical protein | Bcen2424_3058 |
| BCAL0630 | 2.735 | 1E-04 | BCAL0630 | hypothetical protein | Bcen2424_2960 |
| BCAL0761 | 3.819 | 2E-24 | BCAL0761 | hypothetical protein | Bcen2424_2835 |
| BCAL0815 | 4.616 | 8E-06 | BCAL0815 | OstA-like protein | Bcen2424_2789 |
| BCAL0816 | 2.84 | 7E-04 | BCAL0816 | hypothetical protein* | Bcen2424_2788 |
| BCAL0868 | 2.821 | 1E-05 | BCAL0868 | putative GTP cyclohydrolase I | Bcen2424_2737 |
| BCAL0874 | 3.456 | 3E-04 | BCAL0874 | hypothetical protein* | Bcen2424_2731 |
| BCAL0879 | 3.112 | 4E-09 | BCAL0879 | hypothetical protein | No homolog |
| BCAL1032 | 2.248 | 4E-05 | BCAL1032 | hypothetical protein | Bcen2424_1154 |
| BCAL1172 | 2.408 | 3E-03 | BCAL1172 | hypothetical protein | No homolog |
| BCAL1177 | 2.65 | 7E-07 | BCAL1177 | putative fusaric acid resistance transporter protein | No homolog |
| BCAL1286 | 2.548 | 4E-05 | BCAL1286 | hypothetical protein | Bcen2424_1315 |
| BCAL1455 | 2.128 | 1E-04 | BCAL1455 | putative fusaric acid resistance transporter protein* | Bcen2424_1448 |
| BCAL1505 | 3.628 | 2E-05 | BCAL1505 | hypothetical protein | Bcen2424_1498 |
| BCAL1520 | 2.116 | 9E-03 | BCAL1520 | putative lipoprotein* | Bcen2424_1513 |
| BCAL1615 | 2.248 | 2E-12 | BCAL1615 | hypothetical protein | Bcen2424_1558 |
| BCAL1689 | 27.28 | 2E-03 | BCAL1689 | MbtH-like protein | Bcen2424_1633 |
| BCAL1875 | 8.832 | 3E-04 | BCAL1875 | hypothetical protein | Bcen2424_1803 |
| BCAL1881 | 2.511 | 8E-04 | BCAL1881 | putative lipoprotein* | Bcen2424_1809 |
| BCAL1882 | 2.602 | 2E-03 | BCAL1882 | hypothetical protein | Bcen2424_1810 |
| BCAL1904 | 2.032 | 3E-04 | BCAL1904 | hypothetical protein | Bcen2424_1832 |
| BCAL1956 | 5.556 | 6E-04 | BCAL1956 | putative lipoprotein* | Bcen2424_1885 |
| BCAL2075 | 3.819 | 2E-08 | BCAL2075 | putative ATP-binding protein | Bcen2424_2003 |
| BCAL2154 | 2.388 | 4E-04 | BCAL2154 | putative UDP-2,3-diacylglucosamine hydrolase (lipid A biosynthesis related protein) | Bcen2424_2082 |
| BCAL2163 | 5.485 | 1E-03 | BCAL2163 | Cupin superfamily protein* | Bcen2424_2091 |
| BCAL2269 | 10.43 | 1E-04 | BCAL2269 | hypothetical protein | No homolog |
| BCAL2283 | 2.188 | 4E-03 | BCAL2283 | hypothetical protein | Bcen2424_2190 |
| BCAL2286 | 2.372 | 3E-03 | BCAL2286 | hypothetical protein | No homolog |
| BCAL2318 | 3.523 | 1E-06 | BCAL2318 | hypothetical protein | Bcen2424_2223 |
| BCAL2451 | 2.269 | 1E-02 | BCAL2451 | hypothetical protein | No homolog |
| BCAL2475a | 2.392 | 6E-04 | BCAL2475a | hypothetical protein | No homolog |
| BCAL2493 | 2.005 | 6E-03 | BCAL2493 | hypothetical protein | No homolog |
| BCAL2495 | 2.465 | 4E-04 | BCAL2495 | hypothetical protein | No homolog |
| BCAL2647 | 3.027 | 5E-06 | BCAL2647 | hypothetical protein | Bcen2424_2438 |
| BCAL2650 | 2.496 | 2E-04 | BCAL2650 | putative chromosome condensation and segregation protein | Bcen2424_2441 |
| BCAL2654 | 2.113 | 6E-04 | BCAL2654 | hypothetical protein | Bcen2424_2445 |
| BCAL2679 | 3.848 | 8E-05 | BCAL2679 | putative cobaltochelatase | Bcen2424_2469 |
| BCAL2842 | 2.12 | 8E-04 | BCAL2842 | putative branched-chain amino acid transport protein | Bcen2424_2629 |
| BCAL2906 | 3.701 | 2E-07 | BCAL2906 | hypothetical protein | Bcen2424_1092 |
| BCAL2948 | 2.448 | 4E-04 | BCAL2948 | hypothetical protein* | Bcen2424_1049 |
| BCAL3037 | 2.712 | 9E-04 | BCAL3037 | hypothetical protein | Bcen2424_0972 |
| BCAL3205 | 2.167 | 6E-04 | BCAL3205 | hypothetical protein* | Bcen2424_0798 |
| BCAL3212 | 2.321 | 5E-04 | BCAL3212 | hypothetical protein* | Bcen2424_0790 |
| BCAL3279 | 3.008 | 5E-07 | BCAL3279 | hypothetical protein | Bcen2424_0741 |
| BCAL3286 | 2.368 | 3E-04 | BCAL3286 | cobalamin adenosyltransferase protein | Bcen2424_0736 |
| BCAL3310 | 7.708 | 3E-07 | BCAL3310 | hypothetical protein* | Bcen2424_0716 |
| BCAL3311 | 4.252 | 2E-06 | BCAL3311 | hypothetical protein* | Bcen2424_0715 |
| BCAL3471 | 7.35 | 4E-04 | BCAL3471 | protein mraZ | Bcen2424_0550 |
| BCAM0043 | 3.325 | 3E-04 | BCAM0043 | hypothetical protein | Bcen2424_5731 |
| BCAM0367 | 3.909 | 1E-05 | BCAM0367 | putative branched-chain amino acid transport protein | Bcen2424_3355 |
| BCAM0374 | 4.031 | 6E-05 | BCAM0374 | hypothetical protein | Bcen2424_3362 |
| BCAM0380 | 4.797 | 2E-18 | BCAM0380 | hypothetical protein* | Bcen2424_3369 |
| BCAM0397 | 3.448 | 6E-08 | BCAM0397 | hypothetical protein | Bcen2424_3383 |
| BCAM0513 | 2.837 | 2E-18 | BCAM0513 | hypothetical protein | Bcen2424_3478 |
| BCAM0971 | 3.685 | 5E-04 | BCAM0971 | hypothetical protein | Bcen2424_3938 |
| BCAM1027 | 2.293 | 6E-04 | BCAM1027 | hypothetical protein | No homolog |
| BCAM1166 | 3.188 | 9E-05 | BCAM1166 | hypothetical protein | Bcen2424_4060 |
| BCAM1242A | 2.283 | 3E-05 | BCAM1242A | hypothetical protein* | No homolog |
| BCAM1322 | 3.834 | 2E-03 | BCAM1322 | hypothetical protein | Bcen2424_4192 |
| BCAM1425 | 3.12 | 5E-05 | BCAM1425 | hypothetical protein | Bcen2424_4297 |
| BCAM1788 | 2.267 | 1E-05 | BCAM1788 | hypothetical protein | No homolog |
| BCAM1865 | 2.415 | 3E-08 | BCAM1865 | putative multidrug efflux system transporter protein | Bcen2424_4721 |
| BCAM1933 | 2.89 | 2E-04 | BCAM1933 | putative cyclase* | Bcen2424_4739 |
| BCAM2084 | 3.057 | 8E-06 | BCAM2084 | hypothetical protein | Bcen2424_4891 |
| BCAM2085 | 4.498 | 6E-05 | BCAM2085 | hypothetical protein* | Bcen2424_4892 |
| BCAM2087 | 3.436 | 4E-04 | BCAM2087 | putative lipoprotein* | No homolog |
| BCAM2089 | 10.56 | 3E-07 | BCAM2089 | hypothetical protein* | No homolog |
| BCAM2146 | 2.544 | 4E-04 | BCAM2146 | NnrU family protein | Bcen2424_4908 |
| BCAM2253A | 2.227 | 2E-04 | BCAM2253A | hypothetical protein | Bcen2424_5024 |
| BCAM2254 | 3.053 | 1E-04 | BCAM2254 | hypothetical protein | Bcen2424_5025 |
| BCAM2351 | 3.505 | 1E-03 | BCAM2351 | putative transmembrane component of ABC transporter | No homolog |
| BCAM2396 | 2.573 | 2E-03 | BCAM2396 | hypothetical protein | Bcen2424_5156 |
| BCAM2406 | 2.335 | 8E-04 | BCAM2406 | hypothetical protein | Bcen2424_5168 |
| BCAM2650 | 5.281 | 3E-05 | BCAM2650 | putative short-chain dehydrogenase/reductase | Bcen2424_5454 |
| BCAM2767 | 2.19 | 4E-05 | BCAM2767 | hypothetical protein | Bcen2424_5610 |
| BCAM2770 | 2.216 | 5E-04 | BCAM2770 | putative sulfur-binding protein | No homolog |
| BCAS0521 | 3.291 | 5E-05 | BCAS0521 | hypothetical protein | No homolog |
| BCAS0694 | 2.659 | 4E-04 | BCAS0694 | putative carboxymuconolactone decarboxylase family protein | No homolog |
| BCAS0723 | 2.026 | 5E-11 | BCAS0723 | hypothetical protein | No homolog |
| BCAS0745 | 2.6 | 8E-09 | BCAS0745 | hypothetical protein | No homolog |
| BCAS0755 | 2.08 | 1E-03 | BCAS0755 | hypothetical protein* | Bcen2424_6215 |
|  |  |  |  | ***Intergenic regions*** |  |
| IG1_1078680 | 5.011 | 7E-05 | Intergenic region, chromosome 1 | No gene annotation | No homolog |
| IG1_1078958 | 4.08 | 9E-04 | Intergenic region, chromosome 1 | No gene annotation | No homolog |
| IG1_1082987 | 8.32 | 2E-04 | Intergenic region, chromosome 1 | No gene annotation | No homolog |
| IG1_1089442 | 4.956 | 5E-04 | Intergenic region, chromosome 1 | No gene annotation | No homolog |
| IG1_1092335 | 5.321 | 3E-04 | Intergenic region, chromosome 1 | No gene annotation | No homolog |
| IG1_1120751 | 2.91 | 3E-05 | Intergenic region, chromosome 1 | No gene annotation | No homolog |
| IG1_1154931 | 10.69 | 5E-05 | Intergenic region, chromosome 1 | No gene annotation | Bcen2424_1186 |
| IG1_1164190 | 2.655 | 5E-06 | Intergenic region, chromosome 1 | No gene annotation | No homolog |
| IG1_127050 | 2.437 | 4E-04 | Intergenic region, chromosome 1 | No gene annotation | No homolog |
| IG1_129233 | 5.75 | 5E-04 | Intergenic region, chromosome 1 | No gene annotation | No homolog |
| IG1_137022 | 8.247 | 6E-04 | Intergenic region, chromosome 1 | No gene annotation | No homolog |
| IG1_1375706 | 2.133 | 7E-04 | Intergenic region, chromosome 1 | No gene annotation | No homolog |
| IG1_1381576 | 2.51 | 2E-04 | Intergenic region, chromosome 1 | No gene annotation | No homolog |
| IG1_140777 | 2.396 | 5E-03 | Intergenic region, chromosome 1 | No gene annotation | No homolog |
| IG1_147730 | 7.666 | 8E-04 | Intergenic region, chromosome 1 | No gene annotation | No homolog |
| IG1_1507827 | 2.935 | 4E-04 | Intergenic region, chromosome 1 | No gene annotation | No homolog |
| IG1_1556876 | 3.272 | 1E-03 | Intergenic region, chromosome 1 | No gene annotation | No homolog |
| IG1_157905 | 7.518 | 4E-03 | Intergenic region, chromosome 1 | No gene annotation | No homolog |
| IG1_1676264 | 6.282 | 4E-04 | Intergenic region, chromosome 1 | No gene annotation | No homolog |
| IG1_1679445 | 7.77 | 2E-04 | Intergenic region, chromosome 1 | No gene annotation | No homolog |
| IG1_168873 | 8.756 | 1E-04 | Intergenic region, chromosome 1 | No gene annotation | No homolog |
| IG1_1758902 | 4.456 | 6E-04 | Intergenic region, chromosome 1 | No gene annotation | No homolog |
| IG1_1780440 | 4.719 | 3E-03 | Intergenic region, chromosome 1 | No gene annotation | No homolog |
| IG1_1780908 | 2.541 | 1E-03 | Intergenic region, chromosome 1 | No gene annotation | No homolog |
| IG1_1847123 | 33.4 | 6E-05 | Intergenic region, chromosome 1 | No gene annotation | No homolog |
| IG1_1884990 | 4.893 | 5E-10 | Intergenic region, chromosome 1 | No gene annotation | No homolog |
| IG1_1891301 | 4.914 | 1E-06 | Intergenic region, chromosome 1 | No gene annotation | No homolog |
| IG1_2003331 | 2.956 | 9E-08 | Intergenic region, chromosome 1 | No gene annotation | No homolog |
| IG1_2026883 | 2.325 | 6E-03 | Intergenic region, chromosome 1 | No gene annotation | No homolog |
| IG1_2102819 | 5.487 | 2E-05 | Intergenic region, chromosome 1 | No gene annotation | No homolog |
| IG1_2193007 | 4.753 | 6E-04 | Intergenic region, chromosome 1 | No gene annotation | No homolog |
| IG1_22639 | 2.279 | 2E-03 | Intergenic region, chromosome 1 | No gene annotation | No homolog |
| IG1_2266807 | 2.834 | 9E-04 | Intergenic region, chromosome 1 | No gene annotation | Bcen2424_1982 |
| IG1_2319017 | 2.218 | 4E-04 | Intergenic region, chromosome 1 | No gene annotation | No homolog |
| IG1_2323195 | 2.83 | 6E-04 | Intergenic region, chromosome 1 | No gene annotation | No homolog |
| IG1_2378489 | 2.513 | 1E-04 | Intergenic region, chromosome 1 | No gene annotation | No homolog |
| IG1_2382935 | 2.211 | 5E-03 | Intergenic region, chromosome 1 | No gene annotation | No homolog |
| IG1_2384865 | 2.458 | 2E-03 | Intergenic region, chromosome 1 | No gene annotation | No homolog |
| IG1_2389580 | 3.049 | 1E-04 | Intergenic region, chromosome 1 | No gene annotation | No homolog |
| IG1_2445185 | 3.263 | 1E-03 | Intergenic region, chromosome 1 | No gene annotation | No homolog |
| IG1_247826 | 5.405 | 3E-04 | Intergenic region, chromosome 1 | No gene annotation | No homolog |
| IG1_248082 | 2.537 | 6E-04 | Intergenic region, chromosome 1 | No gene annotation | No homolog |
| IG1_2543049 | 13.55 | 2E-04 | Intergenic region, chromosome 1 | No gene annotation | No homolog |
| IG1_257623 | 6.301 | 3E-05 | Intergenic region, chromosome 1 | No gene annotation | No homolog |
| IG1_2617451 | 3.252 | 2E-04 | Intergenic region, chromosome 1 | No gene annotation | No homolog |
| IG1_2645082 | 2.532 | 7E-04 | Intergenic region, chromosome 1 | No gene annotation | No homolog |
| IG1_2646467 | 2.504 | 7E-03 | Intergenic region, chromosome 1 | No gene annotation | No homolog |
| IG1_2647509 | 2.43 | 1E-03 | Intergenic region, chromosome 1 | No gene annotation | No homolog |
| IG1_2651825 | 5.024 | 9E-05 | Intergenic region, chromosome 1 | No gene annotation | No homolog |
| IG1_267619 | 2.901 | 3E-05 | Intergenic region, chromosome 1 | No gene annotation | No homolog |
| IG1_269746 | 3.523 | 2E-06 | Intergenic region, chromosome 1 | No gene annotation | No homolog |
| IG1_2725989 | 5.892 | 9E-04 | Intergenic region, chromosome 1 | No gene annotation | No homolog |
| IG1_2737984 | 3.361 | 2E-04 | Intergenic region, chromosome 1 | No gene annotation | No homolog |
| IG1_274455 | 4.1 | 9E-06 | Intergenic region, chromosome 1 | No gene annotation | No homolog |
| IG1_2758916 | 2.29 | 1E-03 | Intergenic region, chromosome 1 | No gene annotation | No homolog |
| IG1_279976 | 3.582 | 6E-05 | Intergenic region, chromosome 1 | No gene annotation | No homolog |
| IG1_286959 | 3.052 | 1E-07 | Intergenic region, chromosome 1 | No gene annotation | No homolog |
| IG1_288839 | 5.765 | 1E-04 | Intergenic region, chromosome 1 | No gene annotation | No homolog |
| IG1_2921887 | 4.703 | 3E-05 | Intergenic region, chromosome 1 | No gene annotation | No homolog |
| IG1_2978998 | 4.272 | 1E-04 | Intergenic region, chromosome 1 | No gene annotation | No homolog |
| IG1_3008003 | 4.58 | 3E-03 | Intergenic region, chromosome 1 | No gene annotation | No homolog |
| IG1_3056112 | 2.598 | 3E-04 | Intergenic region, chromosome 1 | No gene annotation | No homolog |
| IG1_309252 | 2.046 | 6E-04 | Intergenic region, chromosome 1 | No gene annotation | No homolog |
| IG1_310502 | 2.593 | 3E-04 | Intergenic region, chromosome 1 | No gene annotation | No homolog |
| IG1_3111851 | 2.066 | 8E-05 | Intergenic region, chromosome 1 | No gene annotation | No homolog |
| IG1_3114330 | 2.386 | 4E-05 | Intergenic region, chromosome 1 | No gene annotation | No homolog |
| IG1_3139352 | 3.018 | 2E-03 | Intergenic region, chromosome 1 | No gene annotation | No homolog |
| IG1_3180610 | 8.409 | 9E-04 | Intergenic region, chromosome 1 | No gene annotation | No homolog |
| IG1_3188456 | 2.25 | 4E-04 | Intergenic region, chromosome 1 | No gene annotation | Bcen2424_1088 |
| IG1_3199779 | 2.654 | 2E-03 | Intergenic region, chromosome 1 | No gene annotation | No homolog |
| IG1_32536 | 2.566 | 4E-03 | Intergenic region, chromosome 1 | No gene annotation | Bcen2424_0098 |
| IG1_3308172 | 3.282 | 2E-04 | Intergenic region, chromosome 1 | No gene annotation | No homolog |
| IG1_3308709 | 2.782 | 2E-08 | Intergenic region, chromosome 1 | No gene annotation | No homolog |
| IG1_3338559 | 2.146 | 6E-04 | Intergenic region, chromosome 1 | No gene annotation | No homolog |
| IG1_3342027 | 2.366 | 7E-04 | Intergenic region, chromosome 1 | No gene annotation | No homolog |
| IG1_3345275 | 2.477 | 2E-04 | Intergenic region, chromosome 1 | No gene annotation | No homolog |
| IG1_3348411 | 2.61 | 1E-03 | Intergenic region, chromosome 1 | No gene annotation | No homolog |
| IG1_3368693 | 3.106 | 2E-04 | Intergenic region, chromosome 1 | No gene annotation | No homolog |
| IG1_339954 | 2.505 | 1E-15 | Intergenic region, chromosome 1 | No gene annotation | No homolog |
| IG1_3449993 | 2.198 | 5E-04 | Intergenic region, chromosome 1 | No gene annotation | No homolog |
| IG1_3498894 | 2.223 | 2E-03 | Intergenic region, chromosome 1 | No gene annotation | No homolog |
| IG1_3537906 | 4.787 | 2E-07 | Intergenic region, chromosome 1 | No gene annotation | No homolog |
| IG1_3541037 | 3.329 | 5E-19 | Intergenic region, chromosome 1 | No gene annotation | No homolog |
| IG1_3554535 | 8.694 | 5E-05 | Intergenic region, chromosome 1 | No gene annotation | No homolog |
| IG1_3556424 | 3.655 | 4E-04 | Intergenic region, chromosome 1 | No gene annotation | No homolog |
| IG1_3610794 | 2.008 | 3E-03 | Intergenic region, chromosome 1 | No gene annotation | No homolog |
| IG1_3621670 | 10.56 | 1E-07 | Intergenic region, chromosome 1 | No gene annotation | No homolog |
| IG1_3634213 | 3.018 | 3E-03 | Intergenic region, chromosome 1 | No gene annotation | No homolog |
| IG1_3657187 | 2.667 | 2E-08 | Intergenic region, chromosome 1 | No gene annotation | No homolog |
| IG1_3664357 | 3.423 | 6E-04 | Intergenic region, chromosome 1 | No gene annotation | No homolog |
| IG1_368927 | 3.404 | 4E-04 | Intergenic region, chromosome 1 | No gene annotation | No homolog |
| IG1_3750798 | 2.525 | 1E-03 | Intergenic region, chromosome 1 | No gene annotation | No homolog |
| IG1_37663 | 20.08 | 3E-06 | Intergenic region, chromosome 1 | No gene annotation | No homolog |
| IG1_3776769 | 2.113 | 1E-02 | Intergenic region, chromosome 1 | No gene annotation | No homolog |
| IG1_3822505 | 4.046 | 1E-03 | Intergenic region, chromosome 1 | No gene annotation | No homolog |
| IG1_397645 | 2.499 | 3E-04 | Intergenic region, chromosome 1 | No gene annotation | No homolog |
| IG1_443956 | 2.493 | 1E-03 | Intergenic region, chromosome 1 | No gene annotation | No homolog |
| IG1_446055 | 6.918 | 2E-04 | Intergenic region, chromosome 1 | No gene annotation | No homolog |
| IG1_449463 | 5.307 | 2E-03 | Intergenic region, chromosome 1 | No gene annotation | No homolog |
| IG1_453676 | 3.486 | 1E-04 | Intergenic region, chromosome 1 | No gene annotation | No homolog |
| IG1_463086 | 3.464 | 1E-03 | Intergenic region, chromosome 1 | No gene annotation | No homolog |
| IG1_469909 | 4.82 | 1E-03 | Intergenic region, chromosome 1 | No gene annotation | No homolog |
| IG1_523220 | 2.45 | 2E-03 | Intergenic region, chromosome 1 | No gene annotation | No homolog |
| IG1_52439 | 2.859 | 4E-04 | Intergenic region, chromosome 1 | No gene annotation | No homolog |
| IG1_579967 | 2.465 | 1E-04 | Intergenic region, chromosome 1 | No gene annotation | No homolog |
| IG1_619308 | 9.72 | 5E-04 | Intergenic region, chromosome 1 | No gene annotation | No homolog |
| IG1_631117 | 6.69 | 2E-03 | Intergenic region, chromosome 1 | No gene annotation | No homolog |
| IG1_769618 | 2.102 | 3E-05 | Intergenic region, chromosome 1 | No gene annotation | No homolog |
| IG1_809559 | 3.208 | 3E-04 | Intergenic region, chromosome 1 | No gene annotation | No homolog |
| IG1_816647 | 2.817 | 3E-06 | Intergenic region, chromosome 1 | No gene annotation | No homolog |
| IG1_825611 | 3.971 | 2E-03 | Intergenic region, chromosome 1 | No gene annotation | No homolog |
| IG1_868117 | 2.402 | 2E-05 | Intergenic region, chromosome 1 | No gene annotation | No homolog |
| IG1_890632 | 2.61 | 1E-03 | Intergenic region, chromosome 1 | No gene annotation | No homolog |
| IG1_892194 | 3.575 | 4E-04 | Intergenic region, chromosome 1 | No gene annotation | No homolog |
| IG1_919859 | 2.583 | 2E-02 | Intergenic region, chromosome 1 | No gene annotation | No homolog |
| IG1_950309 | 4.051 | 3E-03 | Intergenic region, chromosome 1 | No gene annotation | No homolog |
| IG1_969583 | 2.548 | 8E-05 | Intergenic region, chromosome 1 | No gene annotation | No homolog |
| IG1_974168 | 4.01 | 2E-03 | Intergenic region, chromosome 1 | No gene annotation | No homolog |
| IG1_99054 | 2.325 | 6E-04 | Intergenic region, chromosome 1 | No gene annotation | No homolog |
| IG2_1076340 | 4.431 | 8E-11 | Intergenic region, chromosome 2 | No gene annotation | No homolog |
| IG2_1175538 | 2.319 | 2E-17 | Intergenic region, chromosome 2 | No gene annotation | No homolog |
| IG2_1199962 | 3.716 | 1E-05 | Intergenic region, chromosome 2 | No gene annotation | No homolog |
| IG2_1452240 | 2.271 | 4E-04 | Intergenic region, chromosome 2 | No gene annotation | No homolog |
| IG2_1537005 | 2.562 | 9E-05 | Intergenic region, chromosome 2 | No gene annotation | No homolog |
| IG2_1656016 | 7.413 | 1E-02 | Intergenic region, chromosome 2 | No gene annotation | No homolog |
| IG2_175839 | 4.205 | 1E-04 | Intergenic region, chromosome 2 | No gene annotation | No homolog |
| IG2_1953120 | 5.774 | 9E-03 | Intergenic region, chromosome 2 | No gene annotation | No homolog |
| IG2_1959827 | 3.926 | 2E-11 | Intergenic region, chromosome 2 | No gene annotation | No homolog |
| IG2_1975919 | 2.211 | 6E-07 | Intergenic region, chromosome 2 | No gene annotation | No homolog |
| IG2_2029035 | 2.251 | 4E-04 | Intergenic region, chromosome 2 | No gene annotation | No homolog |
| IG2_2085682 | 3.314 | 2E-07 | Intergenic region, chromosome 2 | No gene annotation | No homolog |
| IG2_2087974 | 7.006 | 8E-03 | Intergenic region, chromosome 2 | No gene annotation | No homolog |
| IG2_2089610 | 23.42 | 8E-05 | Intergenic region, chromosome 2 | No gene annotation | No homolog |
| IG2_219668 | 2.3 | 3E-04 | Intergenic region, chromosome 2 | No gene annotation | No homolog |
| IG2_2227257 | 11.35 | 7E-04 | Intergenic region, chromosome 2 | No gene annotation | No homolog |
| IG2_2263493 | 2.776 | 4E-03 | Intergenic region, chromosome 2 | No gene annotation | No homolog |
| IG2_2317170 | 2.256 | 2E-07 | Intergenic region, chromosome 2 | No gene annotation | No homolog |
| IG2_2323998 | 2.638 | 1E-03 | Intergenic region, chromosome 2 | No gene annotation | No homolog |
| IG2_2376896 | 2.691 | 5E-04 | Intergenic region, chromosome 2 | No gene annotation | No homolog |
| IG2_2644462 | 5.283 | 8E-05 | Intergenic region, chromosome 2 | No gene annotation | No homolog |
| IG2_2685375 | 2.661 | 1E-04 | Intergenic region, chromosome 2 | No gene annotation | No homolog |
| IG2_2737778 | 4.637 | 2E-04 | Intergenic region, chromosome 2 | No gene annotation | No homolog |
| IG2_2758124 | 2.018 | 1E-02 | Intergenic region, chromosome 2 | No gene annotation | No homolog |
| IG2_2844994 | 2.126 | 2E-03 | Intergenic region, chromosome 2 | No gene annotation | Bcen2424_5310 |
| IG2_2875859 | 2.276 | 5E-07 | Intergenic region, chromosome 2 | No gene annotation | No homolog |
| IG2_2876949 | 2.033 | 7E-04 | Intergenic region, chromosome 2 | No gene annotation | No homolog |
| IG2_2914215 | 2.952 | 2E-05 | Intergenic region, chromosome 2 | No gene annotation | No homolog |
| IG2_2917147 | 2.199 | 4E-04 | Intergenic region, chromosome 2 | No gene annotation | No homolog |
| IG2_3074615 | 2.614 | 2E-03 | Intergenic region, chromosome 2 | No gene annotation | No homolog |
| IG2_3180828 | 2.025 | 2E-03 | Intergenic region, chromosome 2 | No gene annotation | No homolog |
| IG2_529992 | 3.813 | 9E-04 | Intergenic region, chromosome 2 | No gene annotation | No homolog |
| IG2_55282 | 2.36 | 7E-03 | Intergenic region, chromosome 2 | No gene annotation | No homolog |
| IG2_568212 | 3.003 | 2E-04 | Intergenic region, chromosome 2 | No gene annotation | No homolog |
| IG3_11785 | 2.56 | 1E-02 | Intergenic region, chromosome 3 | No gene annotation | No homolog |
| IG3_202414 | 4.448 | 7E-03 | Intergenic region, chromosome 3 | No gene annotation | No homolog |
| IG3_316621 | 2.065 | 3E-03 | Intergenic region, chromosome 3 | No gene annotation | No homolog |
| IG3_72297 | 2.982 | 3E-03 | Intergenic region, chromosome 3 | No gene annotation | No homolog |
| IG3_769736 | 6.278 | 8E-05 | Intergenic region, chromosome 3 | No gene annotation | No homolog |
| IG3_779592 | 2.834 | 3E-03 | Intergenic region, chromosome 3 | No gene annotation | No homolog |
| IG3_781759 | 2.684 | 6E-06 | Intergenic region, chromosome 3 | No gene annotation | No homolog |
| IG3_784806 | 5.022 | 1E-04 | Intergenic region, chromosome 3 | No gene annotation | No homolog |
|  |  |  |  | ***No COG classification*** |  |
| AU1054_G2921 | 7.425 | 3E-03 | No homolog | No gene annotation | Bcen2424_6444 |
| AU1054_G6087 | 2.247 | 3E-08 | No homolog | No gene annotation | No homolog |
| AU1054_G994 | 2.52 | 2E-03 | No homolog | No gene annotation | Bcen2424_0460 |
| BCAL0001 | 2.149 | 2E-03 | BCAL0001 | sodium/hydrogen exchanger family protein | Bcen2424_0068 |
| BCAL0072 | 3.332 | 2E-04 | BCAL0072 | hypothetical protein* | Bcen2424_0143 |
| BCAL0112 | 27.66 | 2E-03 | BCAL0112 | hypothetical protein | Bcen2424_0239 |
| BCAL0137 | 6.289 | 4E-03 | No homolog | No gene annotation | No homolog |
| BCAL0138_J_0 | 2.3 | 5E-04 | BCAL0138 | No gene annotation* | Bcen2424_0265 |
| BCAL0138_J_1 | 3.948 | 7E-16 | BCAL0138 | No gene annotation* | No homolog |
| BCAL0169 | 2.032 | 7E-03 | BCAL0169 | hypothetical protein | No homolog |
| BCAL0206A | 2.699 | 6E-04 | BCAL0206A | putative outer membrane protein* | Bcen2424_0320 |
| BCAL0239 | 14.71 | 5E-07 | Multiple hits | No gene annotation | Bcen2424_0353 |
| BCAL0243 | 12.78 | 2E-24 | Multiple hits | No gene annotation | Bcen2424_0357 |
| BCAL0352 | 2.593 | 1E-04 | BCAL0352 | metallo peptidase, subfamily M15C* | Bcen2424_0486 |
| BCAL0414_J_0 | 5.438 | 2E-04 | No homolog | No gene annotation | No homolog |
| BCAL0419 | 2.203 | 2E-03 | BCAL0419 | hypothetical protein | No homolog |
| BCAL0491 | 4.639 | 5E-03 | BCAL0491 | Major Facilitator Superfamily protein | Bcen2424_3101 |
| BCAL0510 | 2.069 | 9E-05 | BCAL0510 | hypothetical protein* | Bcen2424_3080 |
| BCAL0528 | 8.484 | 2E-03 | BCAL0528 | hypothetical protein | Bcen2424_3061 |
| BCAL0751 | 4.77 | 4E-10 | BCAL0751 | hypothetical protein | Bcen2424_2845 |
| BCAL0753 | 3.868 | 1E-12 | BCAL0753 | hypothetical protein | Bcen2424_2843 |
| BCAL0822 | 3.474 | 5E-05 | BCAL0822 | NUDIX hydrolase | Bcen2424_2782 |
| BCAL0877 | 2.036 | 4E-03 | BCAL0877 | putative methyltransferase | Bcen2424_2728 |
| BCAL0898 | 2.984 | 2E-03 | BCAL0898 | hypothetical protein | Bcen2424_2707 |
| BCAL0955 | 2.039 | 1E-02 | BCAL0955 | hypothetical protein | Bcen2424_2650 |
| BCAL0961 | 4.635 | 7E-04 | BCAL0961 | putative lipoprotein* | No homolog |
| BCAL0989 | 4.609 | 5E-11 | Multiple hits | No gene annotation | Bcen2424_1118 |
| BCAL0990 | 4.054 | 2E-04 | Multiple hits | No gene annotation | Bcen2424_1119 |
| BCAL0994 | 5.376 | 8E-06 | Multiple hits | No gene annotation | Bcen2424_1123 |
| BCAL1002 | 2.583 | 9E-12 | Multiple hits | No gene annotation | Bcen2424_1131 |
| BCAL1004 | 4.199 | 1E-04 | Multiple hits | No gene annotation | Bcen2424_1133 |
| BCAL1009 | 2.265 | 1E-03 | Multiple hits | No gene annotation | No homolog |
| BCAL1012 | 3.664 | 3E-05 | Multiple hits | No gene annotation | Bcen2424_1141 |
| BCAL1019 | 2.518 | 6E-05 | Multiple hits | No gene annotation | Bcen2424_1144 |
| BCAL1041 | 3.572 | 5E-04 | BCAL1041 | hypothetical protein | Bcen2424_1161 |
| BCAL1054 | 2.134 | 7E-17 | BCAL1054 | putative lipoprotein | Bcen2424_1174 |
| BCAL1082 | 2.415 | 2E-03 | BCAL1082 | No gene annotation | Bcen2424_1199 |
| BCAL1189 | 3.471 | 3E-07 | BCAL1189 | hypothetical protein* | No homolog |
| BCAL1301 | 3.531 | 4E-04 | BCAL1301 | hypothetical protein | No homolog |
| BCAL1302_J_0 | 6.256 | 3E-05 | BCAL1302 | No gene annotation | No homolog |
| BCAL1353 | 4.59 | 1E-06 | BCAL1353 | hypothetical protein | No homolog |
| BCAL1363 | 2.404 | 5E-12 | BCAL1363 | hypothetical protein | No homolog |
| BCAL1366 | 2.381 | 2E-05 | BCAL1366 | hypothetical protein | No homolog |
| BCAL1411 | 7.035 | 7E-05 | BCAL1411 | hypothetical protein* | Bcen2424_1403 |
| BCAL1454 | 2.438 | 1E-03 | BCAL1454 | hypothetical protein | Bcen2424_1447 |
| BCAL1560 | 2.017 | 2E-04 | BCAL1560 | hypothetical protein | No homolog |
| BCAL1597 | 2.536 | 8E-04 | BCAL1597 | hypothetical protein* | No homolog |
| BCAL1618 | 2.231 | 2E-06 | BCAL1618 | hypothetical protein | Bcen2424_1561 |
| BCAL1626 | 2.325 | 4E-03 | BCAL1626 | hypothetical protein | No homolog |
| BCAL1680 | 2.185 | 2E-05 | BCAL1680 | putative type-1 fimbrial protein* | No homolog |
| BCAL1681 | 2.112 | 1E-03 | No homolog | No gene annotation | No homolog |
| BCAL1698 | 18.12 | 5E-04 | BCAL1698 | ornibactin biosynthesis protein | No homolog |
| BCAL1702 | 13.32 | 9E-04 | BCAL1702 | putative ornibactin biosynthesis protein | Bcen2424_1646 |
| BCAL1704 | 2.008 | 2E-03 | BCAL1704 | hypothetical protein | No homolog |
| BCAL1707 | 2.828 | 1E-03 | BCAL1707 | putative cobalamin biosynthesis related protein | Bcen2424_1653 |
| BCAL1714 | 2.355 | 1E-06 | No homolog | No gene annotation | No homolog |
| BCAL1758 | 2.626 | 4E-04 | BCAL1758 | hypothetical protein* | No homolog |
| BCAL1811 | 2.116 | 1E-02 | No homolog | No gene annotation | No homolog |
| BCAL1842 | 4.471 | 8E-06 | BCAL1842 | hypothetical protein | No homolog |
| BCAL1930 | 6.614 | 8E-10 | BCAL1930 | SMR family transporter protein | Bcen2424_1858 |
| BCAL2081 | 2.848 | 3E-20 | BCAL2081 | UDP-3-O-[3-hydroxymyristoyl] glucosamine N-acyltransferase | Bcen2424_2009 |
| BCAL2111 | 3.408 | 2E-04 | BCAL2111 | Major Facilitator Superfamily protein* | Bcen2424_2040 |
| BCAL2152 | 2.738 | 4E-04 | No homolog | No gene annotation | Bcen2424_2080 |
| BCAL2164 | 2.664 | 6E-04 | No homolog | No gene annotation | Bcen2424_2092 |
| BCAL2189 | 2.319 | 3E-04 | No homolog | No gene annotation | Bcen2424_2117 |
| BCAL2218 | 2.619 | 2E-18 | Multiple hits | No gene annotation | No homolog |
| BCAL2270 | 6.485 | 1E-05 | BCAL2270 | hypothetical protein | No homolog |
| BCAL2273 | 2.034 | 4E-04 | Multiple hits | No gene annotation | No homolog |
| BCAL2275 | 2.558 | 2E-04 | Multiple hits | No gene annotation | No homolog |
| BCAL2276 | 3.274 | 8E-16 | BCAL2276 | hypothetical protein* | No homolog |
| BCAL2300 | 9.555 | 1E-04 | BCAL2300 | hypothetical protein* | Bcen2424_2208 |
| BCAL2330 | 3.476 | 2E-03 | BCAL2330 | hypothetical protein | No homolog |
| BCAL2436 | 2.533 | 2E-03 | BCAL2436 | hypothetical protein | No homolog |
| BCAL2445 | 3.507 | 2E-04 | BCAL2445 | hypothetical protein | Bcen2424_2350 |
| BCAL2457 | 5.24 | 2E-04 | BCAL2457 | hypothetical protein* | Bcen2424_2362 |
| BCAL2476 | 3.261 | 7E-03 | BCAL2476 | hypothetical protein | No homolog |
| BCAL2479 | 2.117 | 1E-02 | Multiple hits | No gene annotation | No homolog |
| BCAL2480b | 3.599 | 7E-05 | No homolog | No gene annotation | No homolog |
| BCAL2491 | 2.447 | 5E-07 | BCAL2491 | hypothetical protein* | No homolog |
| BCAL2492 | 2.426 | 2E-03 | BCAL2492 | putative transport permease protein | No homolog |
| BCAL2494 | 2.588 | 6E-04 | BCAL2494 | hypothetical protein* | No homolog |
| BCAL2499 | 4.566 | 3E-06 | BCAL2499 | hypothetical protein | No homolog |
| BCAL2500 | 4.336 | 2E-04 | BCAL2500 | hypothetical protein | No homolog |
| BCAL2501 | 2.61 | 7E-05 | BCAL2501 | hypothetical protein | No homolog |
| BCAL2505 | 2.152 | 6E-03 | BCAL2505 | hypothetical protein | No homolog |
| BCAL2523A | 2.481 | 7E-05 | BCAL2523A | hypothetical protein | No homolog |
| BCAL2557 | 3.209 | 3E-12 | BCAL2557 | hypothetical protein | No homolog |
| BCAL2568 | 2.002 | 2E-04 | BCAL2568 | No gene annotation | No homolog |
| BCAL2581_J_1 | 2.368 | 4E-04 | Multiple hits | No gene annotation | No homolog |
| BCAL2598 | 4.308 | 1E-12 | No homolog | No gene annotation | No homolog |
| BCAL2599 | 2.258 | 6E-16 | BCAL2599 | hypothetical protein | No homolog |
| BCAL2600 | 3.563 | 1E-08 | BCAL2600 | integrase | No homolog |
| BCAL2601_J_0 | 3.666 | 2E-04 | BCAL2601 | No gene annotation | No homolog |
| BCAL2601_J_1 | 3.107 | 9E-05 | BCAL2601 | No gene annotation | Bcen2424_2397 |
| BCAL2778 | 2.278 | 9E-05 | BCAL2778 | putative permease protein | Bcen2424_2564 |
| BCAL2846 | 2.659 | 7E-06 | Multiple hits | No gene annotation | Bcen2424_1150 |
| BCAL2860 | 2.972 | 4E-04 | Multiple hits | No gene annotation | Bcen2424_1139 |
| BCAL2867 | 3.778 | 8E-05 | Multiple hits | No gene annotation | Bcen2424_1132 |
| BCAL2872 | 4.824 | 4E-13 | Multiple hits | No gene annotation | Bcen2424_1127 |
| BCAL2873 | 5.408 | 4E-04 | Multiple hits | No gene annotation | Bcen2424_1126 |
| BCAL2875 | 6.036 | 4E-04 | Multiple hits | No gene annotation | Bcen2424_1124 |
| BCAL2877 | 4.358 | 8E-05 | Multiple hits | No gene annotation | Bcen2424_1122 |
| BCAL2878 | 3.215 | 2E-10 | Multiple hits | No gene annotation | Bcen2424_1121 |
| BCAL2879 | 6.495 | 3E-04 | Multiple hits | No gene annotation | Bcen2424_1120 |
| BCAL2882 | 2.041 | 9E-04 | Multiple hits | No gene annotation | No homolog |
| BCAL2883 | 2.347 | 4E-05 | Multiple hits | No gene annotation | Bcen2424_1116 |
| BCAL2888 | 2.684 | 7E-04 | Multiple hits | No gene annotation | No homolog |
| BCAL2896 | 4.011 | 1E-04 | Multiple hits | No gene annotation | Bcen2424_1102 |
| BCAL2897 | 2.041 | 1E-03 | Multiple hits | No gene annotation | Bcen2424_1101 |
| BCAL2904 | 8.904 | 4E-03 | BCAL2904 | hypothetical protein | Bcen2424_1094 |
| BCAL2930 | 2.431 | 2E-07 | BCAL2930 | hypothetical protein* | Bcen2424_1067 |
| BCAL2956 | 2.417 | 1E-03 | BCAL2956 | hypothetical protein* | Bcen2424_1041 |
| BCAL3017 | 3.171 | 7E-10 | BCAL3017 | hypothetical protein* | No homolog |
| BCAL3048 | 2.162 | 4E-06 | BCAL3048 | hypothetical protein | Bcen2424_0962 |
| BCAL3076 | 2.633 | 5E-04 | BCAL3076 | putative integrase | No homolog |
| BCAL3077 | 2.008 | 4E-04 | BCAL3077 | hypothetical protein | No homolog |
| BCAL3083 | 2.583 | 2E-03 | BCAL3083 | hypothetical protein | No homolog |
| BCAL3111 | 3.523 | 2E-04 | BCAL3111 | hypothetical protein | Bcen2424_0895 |
| BCAL3125 | 3.521 | 2E-04 | BCAL3125 | No gene annotation | No homolog |
| BCAL3195a | 2.993 | 1E-03 | BCAL3195a | No gene annotation | Bcen2424_0808 |
| BCAL3217 | 2.396 | 2E-20 | BCAL3217 | putative acetyltransferase protein | No homolog |
| BCAL3228 | 2.698 | 4E-03 | BCAL3228 | hypothetical protein | No homolog |
| BCAL3231 | 4.346 | 3E-04 | BCAL3231 | hypothetical protein | No homolog |
| BCAL3232 | 3.834 | 1E-03 | BCAL3232 | hypothetical protein | No homolog |
| BCAL3233 | 3.495 | 4E-12 | BCAL3233 | glcosyltransferase | No homolog |
| BCAL3248_J_0 | 2.6 | 3E-05 | No homolog | No gene annotation | No homolog |
| BCAL3248_J_1 | 3.132 | 8E-04 | No homolog | No gene annotation | No homolog |
| BCAL3249_J_0 | 2.212 | 4E-03 | BCAL3249 | No gene annotation | No homolog |
| BCAL3271 | 2.767 | 8E-03 | BCAL3271 | thioredoxin | No homolog |
| BCAL3293 | 2.552 | 2E-15 | Multiple hits | No gene annotation | No homolog |
| BCAL3354_J_0 | 3.005 | 6E-04 | BCAL3354 | No gene annotation | Bcen2424_0672 |
| BCAL3354_J_1 | 2.062 | 3E-03 | BCAL3354 | No gene annotation | Bcen2424_0672 |
| BCAL3480 | 3.007 | 4E-05 | BCAL3480 | hypothetical protein | Bcen2424_0014 |
| BCAL3481 | 3.598 | 5E-04 | BCAL3481 | hypothetical protein | Bcen2424_0015 |
| BCAL3482 | 2.859 | 7E-04 | BCAL3482 | hypothetical protein | Bcen2424_0016 |
| BCAL3483 | 4.17 | 3E-03 | BCAL3483 | hypothetical protein | Bcen2424_0017 |
| BCAL3485 | 2.067 | 8E-04 | BCAL3485 | hypothetical protein | No homolog |
| BCAL3517_J_1 | 2.031 | 5E-03 | BCAL3517 | No gene annotation | Bcen2424_0053 |
| BCALr00219 | 6.157 | 3E-04 | BCALr00219 | tRNA-Trp | Bcen2424_R0014 |
| BCALr0080 | 2.295 | 3E-03 | BCALr0080 | tRNA-Arg | Bcen2424_R0005 |
| BCALr0217b | 6.15 | 4E-03 | No homolog | No gene annotation | No homolog |
| BCALr0218b | 2.286 | 3E-03 | No homolog | No gene annotation | No homolog |
| BCALr0218c | 2.953 | 7E-03 | BCALr0218c | tRNA-Thr | Bcen2424_R0013 |
| BCALr0332 | 12.99 | 2E-03 | BCALr0332 | tRNA-Thr | Bcen2424_R0015 |
| BCALr0409c | 8.693 | 3E-03 | Multiple hits | No gene annotation | Bcen2424_R0084, Bcen2424_R0079, Bcen2424_R0063, Bcen2424_R0018, Bcen2424_R0009 |
| BCALr0457 | 10.04 | 3E-02 | BCALr0457 | tRNA-Lys | Bcen2424_R0073 |
| BCALr0914 | 2.198 | 5E-04 | BCALr0914 | misc_RNA | Bcen2424_2689 |
| BCALr0970a | 8.424 | 1E-02 | Multiple hits | No gene annotation | Bcen2424_R0032, Bcen2424_R0031 |
| BCALr1104 | 5.261 | 8E-04 | BCALr1104 | misc_RNA | No homolog |
| BCALr1551a | 3.092 | 3E-02 | Multiple hits | No gene annotation | Bcen2424_R0043, Bcen2424_R0042, Bcen2424_R0041 |
| BCALr1614 | 2.939 | 1E-02 | Multiple hits | No gene annotation | Bcen2424_R0068, Bcen2424_R0044 |
| BCALr1709 | 2.338 | 1E-16 | BCALr1709 | misc_RNA | No homolog |
| BCALr1993b | 5.748 | 2E-02 | No homolog | No gene annotation | No homolog |
| BCALr2006 | 4.323 | 7E-03 | BCALr2006 | tRNA-Leu | Bcen2424_R0049 |
| BCALr2125c | 7.719 | 3E-03 | Multiple hits | No gene annotation | Bcen2424_R0054, Bcen2424_R0052, Bcen2424_R0050, Bcen2424_R0047 |
| BCALr2125e | 10.35 | 4E-03 | Multiple hits | No gene annotation | Bcen2424_R0054, Bcen2424_R0052, Bcen2424_R0050, Bcen2424_R0047 |
| BCALr2125f | 7.712 | 2E-02 | Multiple hits | No gene annotation | Bcen2424_R0055, Bcen2424_R0053, Bcen2424_R0051 |
| BCALr2125g | 2.253 | 1E-02 | BCALr2125g | tRNA-Ala | Bcen2424_R0056 |
| BCALr2145 | 6.603 | 9E-04 | BCALr2145 | tRNA-Ser | Bcen2424_R0057 |
| BCALr2219 | 2.847 | 1E-02 | BCALr2219 | tRNA-Met | Bcen2424_R0058 |
| BCALr2236 | 51.53 | 2E-03 | BCALr2236 | tRNA-Val | Bcen2424_R0059 |
| BCALr2344 | 2.608 | 8E-03 | Multiple hits | No gene annotation | Bcen2424_R0076, Bcen2424_R0066 |
| BCALr2687 | 4.82 | 5E-03 | BCALr2687 | tRNA-Leu | Bcen2424_R0069 |
| BCALr2852d | 3.063 | 1E-02 | Multiple hits | No gene annotation | Bcen2424_R0035, Bcen2424_R0034, Bcen2424_R0033 |
| BCALr3016 | 7.119 | 2E-04 | BCALr3016 | tRNA-Ser | Bcen2424_R0026 |
| BCALr3205 | 35.55 | 4E-03 | BCALr3205 | tRNA-Lys | Bcen2424_R0023 |
| BCALr3443 | 6.732 | 5E-03 | BCALr3443 | tRNA-Pro | Bcen2424_R0021 |
| BCAM0044 | 4.146 | 4E-05 | BCAM0044 | hypothetical protein | Bcen2424_5732 |
| BCAM0045 | 3.358 | 8E-03 | BCAM0045 | putative lipoprotein* | Bcen2424_5733 |
| BCAM0046 | 3.534 | 1E-03 | BCAM0046 | hypothetical protein | No homolog |
| BCAM0129 | 3.381 | 4E-04 | BCAM0129 | TetR family regulatory protein | Bcen2424_5829 |
| BCAM0144 | 2.732 | 3E-12 | BCAM0144 | DnaJ domain protein | Bcen2424_5846 |
| BCAM0151 | 2.056 | 2E-04 | BCAM0151 | No gene annotation | No homolog |
| BCAM0163 | 4.142 | 2E-03 | BCAM0163 | putative microcin immunity protein | No homolog |
| BCAM0174 | 3.654 | 8E-06 | BCAM0174 | hypothetical protein | Bcen2424_3167 |
| BCAM0192 | 6.578 | 1E-03 | BCAM0192 | hypothetical protein | Bcen2424_3184 |
| BCAM0193 | 5.785 | 2E-03 | BCAM0193 | hypothetical protein | Bcen2424_3185 |
| BCAM0194 | 9.07 | 2E-03 | BCAM0194 | hypothetical protein | Bcen2424_3186 |
| BCAM0196 | 6.788 | 1E-03 | BCAM0196 | hypothetical protein | Bcen2424_3188 |
| BCAM0224 | 2.165 | 5E-03 | BCAM0224 | putative haemagglutinin-related autotransporter protein* | No homolog |
| BCAM0225 | 2.194 | 3E-05 | BCAM0225 | No gene annotation* | No homolog |
| BCAM0373 | 2.575 | 3E-03 | BCAM0373 | hypothetical protein* | Bcen2424_3361 |
| BCAM0398 | 2.981 | 4E-07 | BCAM0398 | hypothetical protein | No homolog |
| BCAM0412a | 2.063 | 7E-04 | No homolog | No gene annotation | No homolog |
| BCAM0429 | 2.366 | 8E-04 | BCAM0429 | hypothetical protein | Bcen2424_3396 |
| BCAM0432 | 2.806 | 6E-04 | BCAM0432 | hypothetical protein* | Bcen2424_3399 |
| BCAM0476 | 3.794 | 2E-04 | BCAM0476 | hypothetical protein | No homolog |
| BCAM0493 | 2.631 | 4E-03 | BCAM0493 | hypothetical protein* | No homolog |
| BCAM0510 | 3.159 | 7E-04 | BCAM0510 | hypothetical protein | No homolog |
| BCAM0516 | 2.844 | 7E-19 | BCAM0516 | putative exported glyoxalase | No homolog |
| BCAM0517 | 2.03 | 1E-04 | BCAM0517 | hypothetical protein* | Bcen2424_3494 |
| BCAM0522 | 2.054 | 2E-03 | Multiple hits | No gene annotation | No homolog |
| BCAM0529A | 2.052 | 8E-04 | BCAM0529A | hypothetical protein* | No homolog |
| BCAM0688 | 2.523 | 8E-03 | BCAM0688 | hypothetical protein* | No homolog |
| BCAM0689 | 2.5 | 8E-04 | BCAM0689 | hypothetical protein | Bcen2424_3654 |
| BCAM0706 | 5.898 | 9E-05 | BCAM0706 | No gene annotation* | Bcen2424_3674 |
| BCAM0710 | 3.263 | 6E-03 | BCAM0710 | metallo peptidase, family M35 | No homolog |
| BCAM0731 | 2.7 | 5E-04 | No homolog | No gene annotation | No homolog |
| BCAM0745 | 2.818 | 7E-04 | BCAM0745 | putative shikimate kinase | No homolog |
| BCAM0750 | 2.472 | 2E-04 | BCAM0750 | hypothetical protein | Bcen2424_3716 |
| BCAM0786 | 2.549 | 2E-04 | BCAM0786 | hypothetical protein | No homolog |
| BCAM0787 | 2.121 | 2E-03 | BCAM0788 | No gene annotation | No homolog |
| BCAM0788 | 2.409 | 5E-03 | BCAM0788 | No gene annotation | No homolog |
| BCAM0898 | 2.166 | 4E-03 | BCAM0898 | hypothetical protein | Bcen2424_3866 |
| BCAM0946a | 2.157 | 1E-06 | BCAM0946a | hypothetical protein* | Bcen2424_3913 |
| BCAM0963 | 4.515 | 1E-04 | BCAM0963 | hypothetical protein* | Bcen2424_3930 |
| BCAM1012 | 2.885 | 3E-03 | BCAM1012 | putative histone-like protein* | No homolog |
| BCAM1026 | 2.148 | 5E-04 | BCAM1026 | putative phage DNA-binding protein | No homolog |
| BCAM1030 | 2.143 | 4E-03 | BCAM1030 | hypothetical protein | No homolog |
| BCAM1053C | 3.466 | 4E-04 | BCAM1053C | hypothetical protein | No homolog |
| BCAM1081 | 4.132 | 1E-03 | BCAM1081 | hypothetical protein | No homolog |
| BCAM1082A | 3.228 | 3E-04 | BCAM1082A | putative exported phage protein* | No homolog |
| BCAM1149 | 2.245 | 2E-03 | BCAM1149 | putative lipoprotein* | No homolog |
| BCAM1242 | 2.69 | 7E-05 | BCAM1242 | hypothetical protein* | Bcen2424_4117 |
| BCAM1247 | 2.108 | 1E-02 | BCAM1247 | hypothetical protein* | No homolog |
| BCAM1316a | 2.113 | 2E-02 | No homolog | No gene annotation | No homolog |
| BCAM1316b | 2.54 | 4E-08 | No homolog | No gene annotation | No homolog |
| BCAM1489 | 2.498 | 1E-16 | No homolog | No gene annotation | Bcen2424_4361 |
| BCAM1498_J_0 | 2.959 | 6E-05 | BCAM1498 | No gene annotation | Bcen2424_4371 |
| BCAM1498_J_1 | 4.356 | 6E-04 | BCAM1498 | No gene annotation | Bcen2424_4371 |
| BCAM1518 | 2.163 | 5E-04 | BCAM1518 | hypothetical protein | Bcen2424_4392 |
| BCAM1667 | 2.17 | 2E-10 | BCAM1667 | hypothetical protein | No homolog |
| BCAM1675 | 2.232 | 3E-06 | BCAM1675 | hypothetical protein | Bcen2424_4496 |
| BCAM1745 | 18.66 | 2E-04 | No homolog | No gene annotation | No homolog |
| BCAM1746 | 2.047 | 4E-15 | BCAM1746 | putative DNA-binding protein | Bcen2424_4566 |
| BCAM1749 | 2.385 | 4E-04 | BCAM1749 | hypothetical protein | No homolog |
| BCAM1761 | 2.027 | 2E-05 | BCAM1761 | putative lipoprotein* | Bcen2424_4584 |
| BCAM1811 | 6.276 | 6E-03 | BCAM1811 | hypothetical protein | No homolog |
| BCAM1869 | 3.757 | 4E-05 | BCAM1869 | hypothetical protein | Bcen2424_4725 |
| BCAM1871 | 5.983 | 4E-04 | BCAM1871 | hypothetical protein | No homolog |
| BCAM1874 | 2.023 | 8E-03 | No homolog | No gene annotation | Bcen2424_4731 |
| BCAM1876 | 6.518 | 5E-03 | BCAM1876 | hypothetical protein* | No homolog |
| BCAM1881 | 4.308 | 3E-05 | BCAM1881 | hypothetical protein | No homolog |
| BCAM1882 | 2.47 | 2E-03 | BCAM1882 | hypothetical protein | No homolog |
| BCAM1884 | 7.957 | 2E-03 | BCAM1884 | putative DNA-binding phage protein | No homolog |
| BCAM1919 | 2.331 | 2E-02 | BCAM1919 | hypothetical protein | No homolog |
| BCAM1921 | 3.065 | 8E-04 | BCAM1921 | putative phage membrane protein | No homolog |
| BCAM1922 | 2.229 | 2E-05 | BCAM1922 | putative DNA-binding phage protein | No homolog |
| BCAM1923 | 2.736 | 3E-06 | BCAM1923 | No gene annotation | No homolog |
| BCAM1925 | 5.067 | 3E-04 | Multiple hits | No gene annotation | No homolog |
| BCAM2005 | 2.364 | 1E-07 | BCAM2005 | hypothetical protein | Bcen2424_4805 |
| BCAM2010 | 6.722 | 2E-03 | BCAM2010 | hypothetical protein | Bcen2424_4811 |
| BCAM2042 | 2.234 | 6E-03 | BCAM2042 | type III secretion system protein* | Bcen2424_4841 |
| BCAM2043_J_1 | 2.205 | 3E-04 | BCAM2043 | No gene annotation | No homolog |
| BCAM2050 | 2.862 | 2E-03 | BCAM2050 | type III secretion system protein | Bcen2424_4849 |
| BCAM2053 | 2.518 | 1E-02 | BCAM2053 | hypothetical protein | Bcen2424_4852 |
| BCAM2083 | 5.374 | 2E-05 | BCAM2083 | hypothetical protein | Bcen2424_4890 |
| BCAM2090 | 2.801 | 1E-05 | BCAM2090 | hypothetical protein | No homolog |
| BCAM2143 | 3.227 | 2E-05 | BCAM2143 | cable pilus associated adhesin protein | No homolog |
| BCAM2157 | 2.042 | 4E-11 | BCAM2157 | putative acetyltransferase | Bcen2424_4921 |
| BCAM2177 | 2.48 | 2E-12 | BCAM2177 | hypothetical protein* | No homolog |
| BCAM2204 | 3.815 | 5E-04 | BCAM2204 | hypothetical protein | Bcen2424_4971 |
| BCAM2252_J_1 | 4.546 | 5E-07 | BCAM2252 | No gene annotation | No homolog |
| BCAM2358A | 2.033 | 1E-03 | BCAM2358A | hypothetical protein | Bcen2424_5116 |
| BCAM2359 | 13.1 | 7E-04 | BCAM2359 | hypothetical protein | Bcen2424_5117 |
| BCAM2359A | 4.21 | 2E-03 | BCAM2359A | putative DNA-binding protein | No homolog |
| BCAM2377 | 2.773 | 2E-02 | BCAM2377 | hypothetical protein* | Bcen2424_5137 |
| BCAM2385 | 2.013 | 3E-02 | BCAM2385 | rifampin ADP-ribosyl transferase | No homolog |
| BCAM2417 | 2.295 | 1E-06 | BCAM2417 | hypothetical protein | No homolog |
| BCAM2420 | 2.061 | 6E-04 | BCAM2420 | hypothetical protein | Bcen2424_5225 |
| BCAM2421 | 2.185 | 4E-04 | BCAM2421 | hypothetical protein | Bcen2424_5226 |
| BCAM2422 | 2.052 | 1E-09 | BCAM2422 | hypothetical protein* | No homolog |
| BCAM2427 | 2.072 | 5E-04 | BCAM2427 | hypothetical protein | Bcen2424_5233 |
| BCAM2428 | 2.786 | 2E-11 | BCAM2428 | hypothetical protein | Bcen2424_5234 |
| BCAM2457 | 2.48 | 6E-04 | No homolog | No gene annotation | No homolog |
| BCAM2475 | 2.773 | 5E-06 | BCAM2475 | hypothetical protein* | Bcen2424_5277 |
| BCAM2486 | 4.122 | 3E-03 | BCAM2486 | hypothetical protein* | Bcen2424_5288 |
| BCAM2487 | 8.21 | 1E-04 | BCAM2487 | hypothetical protein | No homolog |
| BCAM2709 | 7.278 | 2E-05 | BCAM2709 | hypothetical protein | Bcen2424_5524 |
| BCAM2755 | 2.145 | 2E-08 | BCAM2755 | hypothetical protein* | Bcen2424_5606 |
| BCAM2759 | 2.472 | 4E-04 | BCAM2759 | putative minor pilin and initiator* | No homolog |
| BCAM2761 | 14.96 | 2E-04 | BCAM2761 | giant cable pilus* | No homolog |
| BCAM2762 | 11.36 | 4E-04 | BCAM2762 | giant cable pilus chaperone protein* | No homolog |
| BCAM2813 | 4.211 | 2E-13 | BCAM2813 | hypothetical protein | Bcen2424_5666 |
| BCAM2837_J_0 | 4.481 | 3E-03 | BCAM2837 | No gene annotation | Bcen2424_5685 |
| BCAM2837_J_1 | 2.6 | 2E-02 | BCAM2837 | No gene annotation | Bcen2424_5686 |
| BCAMr0918 | 3.931 | 2E-03 | BCAMr0918 | tRNA-Met | Bcen2424_R0075 |
| BCAS0063 | 2.411 | 6E-04 | BCAS0063 | hypothetical protein* | No homolog |
| BCAS0064 | 6.698 | 2E-04 | BCAS0064 | hypothetical protein* | No homolog |
| BCAS0174 | 2.18 | 2E-06 | BCAS0174 | hypothetical protein | No homolog |
| BCAS0282 | 2.492 | 5E-03 | BCAS0282 | hypothetical protein | No homolog |
| BCAS0514 | 2.198 | 1E-03 | BCAS0514 | hypothetical protein | No homolog |
| BCAS0515 | 2.482 | 2E-04 | BCAS0515 | putative Lambda G-pre-tape measure frameshift protein | No homolog |
| BCAS0516 | 3.818 | 5E-14 | BCAS0516 | hypothetical protein | No homolog |
| BCAS0519 | 3.716 | 2E-03 | BCAS0519 | hypothetical protein | No homolog |
| BCAS0520 | 3.305 | 4E-08 | BCAS0520 | hypothetical protein | No homolog |
| BCAS0523 | 3.715 | 4E-03 | BCAS0523 | hypothetical protein | No homolog |
| BCAS0524 | 2.566 | 4E-03 | BCAS0524 | hypothetical protein | No homolog |
| BCAS0636 | 4.429 | 3E-02 | BCAS0636 | hypothetical protein | No homolog |
| BCAS0661A | 2.028 | 5E-03 | BCAS0661A | hypothetical protein | No homolog |
| BCAS0661B | 2.848 | 5E-03 | No homolog | No gene annotation | No homolog |
| BCAS0661C | 2.328 | 1E-02 | BCAS0661C | hypothetical protein | No homolog |
| BCAS0674 | 2.148 | 2E-07 | BCAS0674 | hypothetical protein | No homolog |
| BCAS0677 | 2.597 | 1E-02 | BCAS0677 | hypothetical protein | No homolog |
| BCAS0721 | 3.296 | 6E-05 | BCAS0721 | hypothetical protein | No homolog |
| IG1_1235730 | 6.321 | 6E-04 | Multiple hits | No gene annotation | No homolog |
| IG1_1277099 | 3.562 | 5E-05 | BCAL1169 | No gene annotation | No homolog |
| IG1_3414831 | 5.856 | 4E-05 | BCAL3125 | No gene annotation | No homolog |
| IG1_3565903 | 2.411 | 7E-04 | BCAL3258 | tetracycline repressor protein | Bcen2424_0763 |
| IG2_1046147 | 2.152 | 4E-04 | BCAM0945 | hypothetical protein | Bcen2424_3911 |
| IG2_1375301 | 4.902 | 8E-04 | BCAM1251 | No gene annotation | Bcen2424_4127 |
| pBCA012 | 2.162 | 2E-04 | pBCA012 | hypothetical protein | No homolog |
| pBCA029 | 3.023 | 2E-03 | pBCA029 | hypothetical protein | No homolog |
| pBCA030 | 2.341 | 1E-07 | pBCA030 | putative conjugative transfer protein* | No homolog |
| pBCA042 | 2.552 | 2E-04 | pBCA042 | hypothetical protein* | No homolog |
| pBCA047 | 2.58 | 7E-04 | pBCA047 | hypothetical protein | No homolog |
| pBCA048 | 3.273 | 3E-04 | Multiple hits | No gene annotation | No homolog |
| pBCA050 | 2.512 | 2E-03 | pBCA050 | hypothetical protein | No homolog |
| pBCA052 | 2.124 | 2E-03 | pBCA052 | hypothetical protein* | No homolog |
| pBCA056 | 2.255 | 2E-03 | pBCA056 | hypothetical protein | No homolog |
| pBCA058 | 2.461 | 5E-03 | pBCA058 | No gene annotation* | No homolog |

^*^ contains predicted Sec general secretory pathway signal sequence predicted by SignalP software
