# Supplementary material for: Identification of Potential Therapeutic Targets for Burkholderia cenocepacia by Comparative Transcriptomics
Source: PLoS One. 2010 Jan 15;5(1):e8724. doi: 10.1371/journal.pone.0008724 (PMC2806911; doi:10.1371/journal.pone.0008724)
Supplement: Table S2 — Probes showing at least a 2-fold increase in J2315 under SE conditions versus CF conditions. List of genes induced in the clinical isolate J2315 under soil-like conditions compared to CF-like conditions. (2.07 MB DOC) [file pone.0008724.s003.doc]

Table S2. Probes showing at least a 2-fold increase in J2315 under SE conditions versus CF conditions

| **Probe ID** | **J2315 CF/SE expression ratio** | **p-value** | **J2315 Gene** | **Annotation** | **HI2424 homolog** |
| --- | --- | --- | --- | --- | --- |
|  |  |  |  | ***Translation ribosomal structure and biogenesis (J)*** |  |
| BCAL0812 | 0.19 | 5E-08 | BCAL0812 | sigma-54 modulation protein | Bcen2424_2792 |
| BCAL2092 | 0.42 | 7E-13 | BCAL2092 | methionine aminopeptidase | Bcen2424_2020 |
| BCAL2739 | 0.23 | 3E-06 | BCAL2739 | elongation factor G | Bcen2424_2528 |
| BCAM1355 | 0.49 | 7E-04 | BCAM1355 | putative phosphotransferase | No homolog |
| BCAM1544 | 0.42 | 8E-04 | BCAM1544 | putative amidase | No homolog |
| BCAM2131 | 0.25 | 1E-05 | BCAM2131 | 2-aminomuconate deaminase | No homolog |
| BCAM2719 | 0.34 | 1E-04 | BCAM2719 | acetyltransferase (GNAT) family protein | Bcen2424_5533 |
| BCAS0273 | 0.16 | 2E-10 | BCAS0273 | urea amidolyase, allophanate hydrolase subunit | No homolog |
|  |  |  |  | ***Transcription (K)*** |  |
| BCAL0054 | 0.29 | 1E-12 | BCAL0054 | MerR family regulatory protein | Bcen2424_0123 |
| BCAL0364 | 0.40 | 5E-04 | BCAL0364 | TetR family regulatory protein | Bcen2424_0499 |
| BCAL0602 | 0.13 | 8E-10 | BCAL0602 | MerR family regulatory protein | Bcen2424_2991 |
| BCAL0620 | 0.25 | 4E-10 | BCAL0620 | LacI family regulatory protein | Bcen2424_2972 |
| BCAL0813 | 0.33 | 2E-03 | BCAL0813 | putative RNA polymerase sigma-54 factor | Bcen2424_2791 |
| BCAL1150 | 0.34 | 1E-04 | BCAL1150 | AraC family regulatory protein | No homolog |
| BCAL1417 | 0.42 | 1E-08 | BCAL1417 | LysR family regulatory protein | Bcen2424_1410 |
| BCAL1551 | 0.17 | 1E-09 | BCAL1551 | ROK family regulatory protein | Bcen2424_1544 |
| BCAL1557 | 0.20 | 8E-11 | BCAL1557 | GntR family regulatory protein | Bcen2424_1550 |
| BCAL1761 | 0.46 | 5E-05 | BCAL1761 | MarR family regulatory protein | Bcen2424_1698 |
| BCAL1776 | 0.46 | 2E-03 | BCAL1776 | AraC family regulatory protein | No homolog |
| BCAL1802 | 0.45 | 6E-03 | BCAL1802 | LacI family regulatory protein | Bcen2424_1729 |
| BCAL1916 | 0.49 | 4E-03 | BCAL1916 | MerR family regulatory protein | Bcen2424_1844 |
| BCAL1946 | 0.27 | 7E-08 | BCAL1946 | hypothetical protein | Bcen2424_1875 |
| BCAL1947 | 0.22 | 4E-11 | BCAL1947 | putative sigma factor | No homolog |
| BCAL2022 | 0.46 | 3E-06 | BCAL2022 | PspA/IM30 family protein | Bcen2424_1949 |
| BCAL2043 | 0.07 | 2E-08 | BCAL2043 | GntR family regulatory protein | Bcen2424_1966 |
| BCAL2294 | 0.39 | 3E-04 | BCAL2294 | LysR family regulatory protein | Bcen2424_2201 |
| BCAL2309 | 0.34 | 6E-06 | BCAL2309 | putative copper-related MerR family regulatory protein | No homolog |
| BCAL2527 | 0.46 | 2E-04 | BCAL2526a | MerR family regulatory protein | No homolog |
| BCAL2529 | 0.29 | 1E-05 | BCAL2529 | LysR family regulatory protein | No homolog |
| BCAL2537 | 0.38 | 2E-07 | BCAL2537 | LysR family regulatory protein | No homolog |
| BCAL2561 | 0.38 | 2E-08 | BCAL2561 | LysR family regulatory protein | No homolog |
| BCAL2620 | 0.29 | 2E-05 | BCAL2620 | AraC family regulatory protein | Bcen2424_2415 |
| BCAL2825 | 0.46 | 1E-06 | BCAL2825 | AraC family regulatory protein | Bcen2424_2612 |
| BCAL3009 | 0.23 | 3E-11 | BCAL3009 | transcription elongation factor | Bcen2424_1002 |
| BCAL3151 | 0.17 | 1E-04 | BCAL3151 | hypothetical protein | Bcen2424_0855 |
| BCAL3152 | 0.25 | 3E-03 | BCAL3152 | putative RNA polymerase sigma factor | Bcen2424_0854 |
| BCAL3176 | 0.40 | 9E-07 | BCAL3176 | AraC family regulatory protein | No homolog |
| BCAL3320 | 0.33 | 6E-03 | BCAL3320 | ArsR family regulatory protein | No homolog |
| BCAL3382 | 0.43 | 8E-03 | BCAL3382 | GntR family regulatory protein | No homolog |
| BCAL3409 | 0.23 | 4E-11 | BCAL3409 | IclR family regulatory protein | Bcen2424_0617 |
| BCAL3478 | 0.32 | 2E-04 | BCAL3478 | putative RNA polymerase sigma factor | Bcen2424_0011 |
| BCAL3479 | 0.30 | 2E-04 | BCAL3479 | putative transmembrane regulator | No homolog |
| BCAM0001 | 0.43 | 3E-03 | BCAM0001 | putative sigma factor | Bcen2424_5690 |
| BCAM0038 | 0.48 | 1E-06 | BCAM0038 | AraC family regulatory protein | Bcen2424_5729 |
| BCAM0259 | 0.19 | 3E-11 | BCAM0259 | putative repressor protein | No homolog |
| BCAM0489 | 0.45 | 3E-03 | BCAM0489 | MerR family regulatory protein | No homolog |
| BCAM0525 | 0.25 | 2E-09 | BCAM0525 | putative aminotransferase protein | Bcen2424_3498 |
| BCAM0575 | 0.36 | 8E-10 | BCAM0575 | LysR family regulatory protein | Bcen2424_3549 |
| BCAM0626 | 0.49 | 2E-05 | BCAM0626 | putative DNA-binding protein | Bcen2424_3598 |
| BCAM0635 | 0.37 | 4E-05 | BCAM0635 | AnsC family regulatory protein | No homolog |
| BCAM0673 | 0.28 | 6E-11 | BCAM0673 | IclR family regulatory protein | Bcen2424_3637 |
| BCAM0700 | 0.34 | 8E-10 | BCAM0700 | TetR family regulatory protein | Bcen2424_3666 |
| BCAM0794 | 0.26 | 5E-09 | BCAM0794 | LysR family regulatory protein | No homolog |
| BCAM0795 | 0.39 | 1E-07 | BCAM0795 | MarR family regulatory protein | No homolog |
| BCAM0809 | 0.25 | 4E-04 | BCAM0809 | AraC family regulatory protein | No homolog |
| BCAM0815 | 0.47 | 1E-02 | BCAM0815 | putative DNA binding protein | Bcen2424_3780 |
| BCAM0866 | 0.22 | 9E-12 | BCAM0866 | MarR family regulatory protein | Bcen2424_3831 |
| BCAM0868 | 0.41 | 3E-12 | BCAM0868 | AraC family regulatory protein | Bcen2424_3835 |
| BCAM0958 | 0.32 | 4E-12 | BCAM0958 | ArsR family regulatory protein | Bcen2424_3925 |
| BCAM1169 | 0.41 | 5E-05 | BCAM1169 | IclR family regulatory protein | No homolog |
| BCAM1206 | 0.28 | 5E-06 | BCAM1206 | LysR family regulatory protein | Bcen2424_4080 |
| BCAM1232 | 0.04 | 7E-07 | BCAM1232 | LysR family regulatory protein | Bcen2424_4107 |
| BCAM1259 | 0.42 | 7E-03 | BCAM1259 | RNA polymerase sigma factor | Bcen2424_4132 |
| BCAM1290 | 0.39 | 2E-12 | BCAM1290 | RpiR-family transcriptional regulator | No homolog |
| BCAM1402 | 0.45 | 6E-12 | BCAM1402 | LysR family regulatory protein | Bcen2424_4267 |
| BCAM1448 | 0.33 | 2E-07 | BCAM1448 | LuxR superfamily regulatory protein | Bcen2424_4320 |
| BCAM1476 | 0.44 | 6E-08 | BCAM1476 | AnsC family regulatory protein | Bcen2424_4348 |
| BCAM1536 | 0.28 | 3E-05 | BCAM1536 | TetR family regulatory protein | Bcen2424_4403 |
| BCAM1546 | 0.40 | 5E-09 | BCAM1546 | hypothetical protein | No homolog |
| BCAM1661 | 0.19 | 1E-10 | BCAM1661 | RNA polymerase sigma factor | Bcen2424_4479 |
| BCAM1728 | 0.34 | 7E-12 | BCAM1728 | LysR family regulatory protein | Bcen2424_4546 |
| BCAM1768 | 0.02 | 4E-07 | BCAM1768 | hypothetical protein | No homolog |
| BCAM1796 | 0.49 | 3E-12 | BCAM1796 | LysR family regulatory protein | No homolog |
| BCAM1932 | 0.29 | 1E-12 | BCAM1932 | HxlR-family transcriptional regulator | No homolog |
| BCAM1966 | 0.24 | 4E-13 | BCAM1966 | ArsR family regulatory protein | Bcen2424_4763 |
| BCAM1975 | 0.05 | 2E-07 | BCAM1975 | AraC family regulatory protein | Bcen2424_4777 |
| BCAM2095 | 0.10 | 1E-07 | BCAM2095 | putative DNA-binding protein | Bcen2424_4902 |
| BCAM2117 | 0.41 | 3E-02 | BCAM2117 | LysR family regulatory protein | No homolog |
| BCAM2150 | 0.38 | 2E-12 | BCAM2150 | AraC family regulatory protein | Bcen2424_4913 |
| BCAM2327 | 0.01 | 4E-07 | BCAM2327 | AraC family regulatory protein | Bcen2424_5086 |
| BCAM2329 | 0.04 | 2E-07 | BCAM2329 | GntR family regulatory protein | No homolog |
| BCAM2435 | 0.49 | 2E-12 | BCAM2435 | MarR family regulatory protein | Bcen2424_5242 |
| BCAM2655 | 0.48 | 3E-13 | BCAM2655 | ArsR family regulatory protein | Bcen2424_5459 |
| BCAM2802 | 0.40 | 8E-12 | BCAM2802 | LysR family regulatory protein | No homolog |
| BCAS0024 | 0.40 | 3E-04 | BCAS0024 | GntR family regulatory protein | No homolog |
| BCAS0115 | 0.09 | 1E-07 | BCAS0115 | GntR family regulatory protein* | Bcen2424_6110 |
| BCAS0225 | 0.38 | 5E-04 | BCAS0225 | LysR family regulatory protein | Bcen2424_5999 |
| BCAS0556 | 0.03 | 3E-07 | BCAS0556 | AraC family regulatory protein | Bcen2424_6349 |
| BCAS0581 | 0.46 | 1E-02 | BCAS0581 | putative transcriptional regulatory protein | No homolog |
|  |  |  |  | ***Replication recombination and repair (L)*** |  |
| BCAL0056 | 0.48 | 2E-02 | BCAL0056 | AraC family regulatory protein | Bcen2424_0126 |
| BCAL0288 | 0.19 | 1E-11 | BCAL0288 | hypothetical protein | Bcen2424_0401 |
| BCAL0438 | 0.31 | 3E-09 | BCAL0438 | putative DNA-3-methyladenine glycosylase II | Bcen2424_3151 |
| BCAL1500 | 0.01 | 6E-07 | BCAL1500 | hypothetical protein | Bcen2424_1493 |
| BCAL2713 | 0.48 | 3E-05 | BCAL2713 | putative DNA repair protein | Bcen2424_2501 |
| BCAL2924 | 0.35 | 2E-11 | BCAL2924 | NUDIX hydrolase | Bcen2424_1073 |
| BCAM0771 | 0.03 | 3E-07 | BCAM0771 | putative transposase | No homolog |
| BCAM0917 | 0.27 | 8E-08 | BCAM0917 | putative DNA primase | Bcen2424_3885 |
| BCAM1352 | 0.42 | 1E-04 | BCAM1352 | putative phosphoesterase | Bcen2424_4219 |
| BCAM1690 | 0.08 | 5E-08 | BCAM1690 | uracil DNA glycosylase superfamily protein | Bcen2424_4512 |
| IG1_2495977 | 0.19 | 9E-10 | BCAL2252 | putative DNA photolyase | No homolog |
| IG2_1099336 | 0.49 | 5E-07 | BCAM0992 | putative DNA methylase | Bcen2424_3959 |
|  |  |  |  | ***Chromatin structure and dynamics (B)*** |  |
| BCAL1225 | 0.24 | 8E-11 | BCAL1225 | putative aminohydrolase | No homolog |
|  |  |  |  | ***Cell cycle control cell division chromosome partitioning (D)*** |  |
| BCAM0006 | 0.50 | 2E-04 | BCAM0006 | hypothetical protein | Bcen2424_5697 |
|  |  |  |  | ***Defense mechanisms (V)*** |  |
| BCAM2334 | 0.07 | 1E-07 | BCAM2334 | efflux system transport protein | Bcen2424_5091 |
|  |  |  |  | ***Signal transduction mechanisms (T)*** |  |
| BCAL0008 | 0.27 | 4E-07 | BCAL0008 | two-component regulatory system, response regulator protein | Bcen2424_0075 |
| BCAL0502 | 0.32 | 6E-03 | BCAL0502 | DksA/TraR C4-type zinc finger family protein | Bcen2424_3090 |
| BCAL0652 | 0.04 | 2E-07 | BCAL0652 | putative diguanylate phosphodiesterase | Bcen2424_2938 |
| BCAL1280 | 0.43 | 2E-11 | BCAL1280 | putative hydrolase | Bcen2424_1310 |
| BCAL1318 | 0.06 | 7E-08 | BCAL1318 | putative nitrate regulatory protein | Bcen2424_6121 |
| BCAL1648 | 0.02 | 3E-07 | BCAL1648 | putative stress-related protein | Bcen2424_1597 |
| BCAL1663 | 0.06 | 8E-08 | BCAL1663 | PrkA family serine protein kinase | Bcen2424_1613 |
| BCAL1975 | 0.47 | 3E-09 | BCAL1975 | putative diguanylate cyclase | Bcen2424_1903 |
| BCAL2119 | 0.30 | 1E-11 | BCAL2119 | universal stress protein family protein | Bcen2424_2049 |
| BCAL2210 | 0.44 | 3E-03 | BCAL2210 | two-component regulatory system, sensor kinase protein | Bcen2424_2138 |
| BCAL2222 | 0.03 | 4E-07 | BCAL2222 | nitrogen regulation protein NR(I) | Bcen2424_2147 |
| BCAL2223 | 0.07 | 4E-07 | BCAL2223 | putative nitrogen regulation protein NR(II) | Bcen2424_2148 |
| BCAL2605 | 0.41 | 4E-07 | BCAL2605 | two-component regulatory system, sensor kinase protein | Bcen2424_2400 |
| BCAL2749 | 0.05 | 2E-07 | BCAL2749 | putative diguanylate phosphodiesterase | Bcen2424_2537 |
| BCAL3188 | 0.46 | 5E-10 | BCAL3188 | putative diguanylate phosphodiesterase | Bcen2424_0816 |
| BCAM0028 | 0.27 | 2E-04 | BCAM0028 | putative FHA-domain protein | Bcen2424_5719 |
| BCAM0288 | 0.49 | 5E-03 | BCAM0288 | two-component regulatory system, response regulator protein | No homolog |
| BCAM0289 | 0.42 | 2E-04 | BCAM0289 | two-component regulatory system, sensor kinase protein | No homolog |
| BCAM0580 | 0.40 | 2E-04 | BCAM0580 | putative cyclic-di-GMP signaling protein | Bcen2424_3554 |
| BCAM0622 | 0.20 | 2E-11 | BCAM0622 | two-component regulatory system, sensor kinase protein | Bcen2424_3594 |
| BCAM0623 | 0.19 | 3E-11 | BCAM0623 | two-component regulatory system, response regulator protein | Bcen2424_3595 |
| BCAM0639 | 0.23 | 6E-12 | BCAM0639 | LuxR superfamily regulatory protein | Bcen2424_3610 |
| BCAM0645 | 0.43 | 1E-07 | BCAM0645 | two-component regulatory system, sensor kinase protein | Bcen2424_3616 |
| BCAM0748 | 0.49 | 4E-05 | BCAM0748 | putative diguanylate cyclase | Bcen2424_3714 |
| BCAM0820 | 0.47 | 6E-03 | BCAM0820 | hybrid two-component system kinase-response regulator protein | Bcen2424_3785 |
| BCAM0857 | 0.01 | 1E-06 | BCAM0857 | low molecular weight protein-tyrosine-phosphatase | Bcen2424_3822 |
| BCAM0871 | 0.05 | 3E-07 | BCAM0871 | putative transcriptional regulator | Bcen2424_3839 |
| BCAM1342 | 0.39 | 4E-06 | BCAM1342 | putative sigma-54 interacting transcriptional regulator | Bcen2424_4209 |
| BCAM1348 | 0.26 | 7E-08 | BCAM1348 | putative cyclic nucleotide-binding protein | Bcen2424_4215 |
| BCAM1351 | 0.22 | 1E-08 | BCAM1351 | putative regulatory protein | Bcen2424_4218 |
| BCAM1483 | 0.09 | 9E-08 | BCAM1483 | cyclic nucleotide-binding transcriptional regulator | Bcen2424_4355 |
| BCAM1484 | 0.12 | 7E-09 | BCAM1484 | two-component regulatory system, response regulator protein | Bcen2424_4356 |
| BCAM1495 | 0.30 | 6E-11 | BCAM1495 | putative universal stress protein | Bcen2424_4367 |
| BCAM1504 | 0.12 | 2E-09 | BCAM1504 | putative sigma-54 interacting transcriptional regulator | No homolog |
| BCAM1688 | 0.01 | 7E-07 | BCAM1688 | two-component regulatory system, response regulator protein | No homolog |
| BCAM1723 | 0.41 | 2E-11 | BCAM1723 | hypothetical protein | No homolog |
| BCAM1802 | 0.32 | 2E-04 | BCAM1802 | putative regulatory protein | Bcen2424_4663 |
| BCAM1926 | 0.28 | 3E-12 | BCAM1926 | hypothetical protein | No homolog |
| BCAM2039 | 0.12 | 3E-08 | BCAM2039 | putative transcriptional regulator | Bcen2424_4838 |
| BCAM2058 | 0.22 | 3E-11 | BCAM2058 | LuxR superfamily regulatory protein | Bcen2424_4857 |
| BCAM2059 | 0.20 | 4E-11 | BCAM2059 | LuxR superfamily regulatory protein | Bcen2424_4858 |
| BCAM2756 | 0.43 | 1E-02 | BCAM2756 | two-component regulatory system, response regulator protein | No homolog |
|  |  |  |  | ***Cell wall/membrane/envelope biogenesis (M)*** |  |
| BCAL0123 | 0.29 | 2E-12 | BCAL0123 | putative glycosyltransferase | Bcen2424_0250 |
| BCAL0584 | 0.40 | 4E-06 | BCAL0584 | putative outer membrane porin protein* | Bcen2424_3008 |
| BCAL0624 | 0.15 | 3E-06 | BCAL0624 | putative outer membrane porin protein precursor* | No homolog |
| BCAL0940 | 0.06 | 2E-08 | BCAL0940 | putative transglycosylase | Bcen2424_2662 |
| BCAL1147 | 0.21 | 2E-11 | BCAL1147 | glycosyltransferase | No homolog |
| BCAL1368 | 0.20 | 1E-10 | BCAL1368 | putative porin | Bcen2424_1357 |
| BCAL1957 | 0.44 | 7E-06 | BCAL1957 | putative sugar transferase | Bcen2424_1886 |
| BCAL2289 | 0.26 | 4E-12 | BCAL2289 | putative glutamate racemase | Bcen2424_2196 |
| BCAL2615 | 0.07 | 3E-08 | BCAL2615 | putative exported outer membrane porin protein | Bcen2424_2410 |
| BCAL3019 | 0.15 | 1E-09 | BCAL3019 | putative lipoprotein | Bcen2424_0992 |
| BCAL3116 | 0.40 | 7E-04 | BCAL3116 | glycosyltransferase | No homolog |
| BCAL3120 | 0.43 | 8E-06 | BCAL3120 | UDP-N-acetylglucosamine-1-P transferase | No homolog |
| BCAL3149 | 0.31 | 2E-12 | BCAL3149 | hypothetical protein | No homolog |
| BCAL3473 | 0.07 | 2E-07 | BCAL3473 | putative outer membrane porin | Bcen2424_0548 |
| BCAL3508 | 0.06 | 8E-08 | BCAL3508 | LrgB family protein | Bcen2424_0042 |
| BCAM0071 | 0.24 | 5E-09 | BCAM0071 | puatative mandelate racemase/muconate lactonizing enzyme | No homolog |
| BCAM0805 | 0.32 | 5E-12 | BCAM0805 | muconate cycloisomerase I 1 | Bcen2424_3770 |
| BCAM0854 | 0.01 | 5E-07 | BCAM0854 | bifunctional exopolysaccharide biosynthesis protein (phosphomannose isomerase and GDP-D-mannose pyrophosphorylase) | Bcen2424_3819 |
| BCAM0855 | 0.01 | 2E-06 | BCAM0855 | UDP-glucose dehydrogenase | Bcen2424_3821 |
| BCAM0858 | 0.01 | 5E-07 | BCAM0858 | putative polysaccharide biosynthesis/export lipoprotein | Bcen2424_3823 |
| BCAM0859 | 0.01 | 6E-07 | BCAM0859 | tyrosine-protein kinase | Bcen2424_3824 |
| BCAM0861 | 0.01 | 6E-07 | BCAM0861 | putative glycosyltransferase | Bcen2424_3826 |
| BCAM0863 | 0.02 | 1E-06 | BCAM0863 | putative glycosyltransferase | Bcen2424_3828 |
| BCAM0864 | 0.01 | 5E-07 | BCAM0864 | glycosyltransferase | Bcen2424_3829 |
| BCAM1003 | 0.01 | 3E-06 | BCAM1003 | putative epimerase* | Bcen2424_3969 |
| BCAM1004 | 0.01 | 5E-07 | BCAM1004 | GCP-mannose 4,6-dehydratase | Bcen2424_3970 |
| BCAM1008 | 0.01 | 4E-07 | BCAM1008 | glycosyltransferase | Bcen2424_3974 |
| BCAM1010 | 0.02 | 4E-07 | BCAM1010 | putative UTP-glucose-1-phosphate uridylyltransferase | Bcen2424_3977 |
| BCAM1015 | 0.08 | 7E-09 | BCAM1015 | putative porin | Bcen2424_3981 |
| BCAM1128 | 0.35 | 2E-10 | BCAM1128 | putative glycosyl transferase family protein | Bcen2424_4024 |
| BCAM1350 | 0.46 | 2E-08 | BCAM1350 | putative sugar transferase | Bcen2424_4217 |
| BCAM1398 | 0.06 | 7E-08 | BCAM1398 | putative porin | No homolog |
| BCAM1514 | 0.33 | 2E-05 | BCAM1514 | putative outer membrane protein* | No homolog |
| BCAM1550 | 0.19 | 4E-11 | BCAM1550 | putative peptidoglycan-associated lipoprotein | Bcen2424_4417 |
| BCAM1769 | 0.04 | 2E-07 | BCAM1769 | putative D-alanyl-D-alanine dipeptidase | Bcen2424_4590 |
| BCAM1787 | 0.18 | 2E-10 | BCAM1787 | putative porin | Bcen2424_4607 |
| BCAM1808 | 0.44 | 5E-11 | BCAM1808 | putative acetyltransferase | Bcen2424_4669 |
| BCAM1855 | 0.49 | 2E-08 | BCAM1855 | putative porin | Bcen2424_4712 |
| BCAM2284 | 0.25 | 7E-08 | BCAM2284 | putative mrandelate racemase/muconate lactonizing enzyme | Bcen2424_5041 |
| BCAM2335 | 0.17 | 5E-10 | BCAM2335 | outer membrane efflux protein* | No homolog |
| BCAS0121 | 0.31 | 7E-04 | BCAS0121 | putative porin protein | No homolog |
| BCAS0220 | 0.47 | 4E-07 | BCAS0220 | putative permease | No homolog |
| BCAS0256 | 0.21 | 7E-12 | BCAS0256 | putative porin protein | No homolog |
| BCAS0471 | 0.40 | 1E-04 | BCAS0471 | outer membrane efflux protein | No homolog |
|  |  |  |  | ***Cell motility (N)*** |  |
| BCAL1452 | 0.30 | 2E-06 | BCAL1452 | putative methyl-accepting chemotaxis protein | Bcen2424_1445 |
| BCAL1532 | 0.37 | 4E-12 | BCAL1532 | flp type pilus assembly protein | Bcen2424_1525 |
| BCAM0825 | 0.49 | 1E-03 | BCAM0825 | putative chemotaxis-related two component response regulator/histidine kinase | No homolog |
| BCAM1572 | 0.42 | 4E-04 | BCAM1572 | methyl-accepting chemotaxis protein | Bcen2424_4440 |
|  |  |  |  | ***Intracellular trafficking secretion and vesicular transport (U)*** |  |
| BCAL1525 | 0.19 | 8E-09 | BCAL1525 | flp type pilus subunit | No homolog |
| BCAL1526 | 0.39 | 7E-12 | BCAL1526 | putative flp type pilus assembly protein | No homolog |
| BCAL1527 | 0.30 | 1E-12 | BCAL1527 | flp type pilus assembly protein | No homolog |
| BCAL1529 | 0.33 | 1E-12 | BCAL1529 | flp pilus type assembly-related protein | Bcen2424_1522 |
| BCAL1530 | 0.39 | 2E-04 | BCAL1530 | flp pilus type assembly protein | No homolog |
| BCAL1531 | 0.45 | 4E-12 | BCAL1531 | flp type pilus assembly protein | Bcen2424_1524 |
| BCAL1533 | 0.36 | 1E-11 | BCAL1533 | putative lipoprotein* | Bcen2424_1526 |
| BCAL1787 | 0.31 | 1E-06 | BCAL1787 | putative iron-uptake protein | Bcen2424_1714 |
| BCAL1788 | 0.27 | 9E-12 | BCAL1788 | putative iron-transport protein | No homolog |
| BCAL1789 | 0.36 | 1E-07 | BCAL1789 | putative iron-transport protein* | No homolog |
| BCAM0561 | 0.36 | 8E-07 | BCAM0561 | transport protein | Bcen2424_3535 |
|  |  |  |  | ***Posttranslational modification protein turnover chaperones (O)*** |  |
| BCAL0372 | 0.38 | 1E-04 | BCAL0372 | putative glutaredoxin | Bcen2424_0508 |
| BCAL0540 | 0.04 | 3E-07 | BCAL0540 | putative ATPase | Bcen2424_3049 |
| BCAL0647 | 0.46 | 1E-04 | BCAL0647 | putative glutathione S-transferase | Bcen2424_2943 |
| BCAL1234 | 0.42 | 2E-02 | BCAL1234 | putative heat shock protein | Bcen2424_1263 |
| BCAL1525a | 0.37 | 2E-12 | BCAL1525a | putative flp type pilus leader peptidase | Bcen2424_1518 |
| BCAL1612 | 0.21 | 3E-09 | BCAL1612 | putative arginine-tRNA-protein transferase | Bcen2424_1555 |
| BCAL2730 | 0.27 | 9E-08 | BCAL2730 | putative ATP-dependent Clp protease ATP-binding subunit | Bcen2424_2518 |
| BCAL3103 | 0.11 | 6E-07 | BCAL3103 | UreD-family accessory protein | Bcen2424_0903 |
| BCAL3107 | 0.02 | 7E-07 | BCAL3107 | urease accessory protein | No homolog |
| BCAL3108 | 0.17 | 1E-10 | BCAL3108 | urease accessory protein | Bcen2424_0898 |
| BCAL3109 | 0.02 | 6E-07 | BCAL3109 | urease accessory protein | Bcen2424_0897 |
| BCAL3171 | 0.28 | 6E-07 | BCAL3171 | hypothetical protein | Bcen2424_0834 |
| BCAL3331 | 0.25 | 2E-12 | BCAL3331 | putative glutathione S-transferase | Bcen2424_0696 |
| BCAM0727 | 0.42 | 8E-10 | BCAM0727 | hypothetical protein | Bcen2424_3693 |
| BCAM0775 | 0.01 | 1E-06 | BCAM0775 | glutathione-S-tranferase family protein | Bcen2424_3732 |
| BCAM0896 | 0.31 | 1E-11 | BCAM0896 | putative organic hydroperoxide resistance protein | Bcen2424_3864 |
| BCAM2339 | 0.04 | 4E-07 | BCAM2339 | putative methyltransferase | Bcen2424_5096 |
| BCAM2401 | 0.32 | 2E-04 | BCAM2401 | putative aspartyl/asparaginyl beta-hydroxylase | Bcen2424_5163 |
| BCAM2640 | 0.32 | 3E-12 | BCAM2640 | putative methyltransferase | No homolog |
|  |  |  |  | ***Energy production and conversion (C)*** |  |
| BCAL0064 | 0.08 | 5E-08 | BCAL0064 | acetaldehyde dehydrogenase | Bcen2424_0134 |
| BCAL0330 | 0.48 | 2E-04 | BCAL0330 | cytochrome c1 precursor | Bcen2424_0443 |
| BCAL0441 | 0.39 | 8E-11 | BCAL0441 | putative L-lactate permease | Bcen2424_3147 |
| BCAL0518 | 0.49 | 1E-09 | BCAL0518 | putative coniferyl aldehyde dehydrogenase | Bcen2424_3072 |
| BCAL0539 | 0.40 | 9E-04 | BCAL0539 | putative nitroreductase/p-nitrobenzoate reductase | Bcen2424_3050 |
| BCAL0603 | 0.14 | 5E-08 | BCAL0603 | gamma-glutamyl-gamma-aminobutyraldehyde dehydrogenase | Bcen2424_2990 |
| BCAL1049 | 0.32 | 1E-12 | BCAL1049 | luciferase-like monooxygenase | Bcen2424_1169 |
| BCAL1106 | 0.18 | 8E-11 | BCAL1106 | cytochrome b561 family protein | No homolog |
| BCAL1212 | 0.47 | 4E-04 | BCAL1212 | 2-oxoisovalerate dehydrogenase alpha subunit | Bcen2424_1243 |
| BCAL1803 | 0.07 | 2E-07 | BCAL1803 | 2-ketogluconate reductase | Bcen2424_1731 |
| BCAL2118 | 0.49 | 2E-05 | BCAL2118 | isocitrate lyase | Bcen2424_2048 |
| BCAL2142 | 0.47 | 2E-02 | BCAL2142 | cytochrome o ubiquinol oxidase subunit III | Bcen2424_2069 |
| BCAL2143 | 0.26 | 4E-03 | BCAL2143 | ubiquinol oxidase polypeptide I | Bcen2424_2070 |
| BCAL2144 | 0.33 | 2E-03 | BCAL2144 | ubiquinol oxidase polypeptide II precursor | Bcen2424_2071 |
| BCAL2145 | 0.03 | 7E-07 | BCAL2145 | NADH-ubiquinone oxidoreductase subunit | Bcen2424_2072 |
| BCAL2197 | 0.49 | 9E-04 | BCAL2197 | putative iron-sulfur cluster scaffold protein | Bcen2424_2125 |
| BCAL2305 | 0.20 | 7E-11 | BCAL2305 | putative potassium channel subunit | Bcen2424_2213 |
| BCAL2623 | 0.44 | 2E-02 | BCAL2623 | putative aldehyde dehydrogenase | Bcen2424_2418 |
| BCAL2745 | 0.49 | 1E-02 | BCAL2745 | putative CoA transferase family protein | No homolog |
| BCAL2976 | 0.44 | 1E-03 | BCAL2976 | NAD-dependent formate dehydrogenase gamma subunit | Bcen2424_1033 |
| BCAL2977 | 0.39 | 7E-04 | BCAL2977 | NAD-dependent formate dehydrogenase beta subunit | Bcen2424_1032 |
| BCAL3381 | 0.17 | 2E-09 | BCAL3381 | aerobic C4-dicarboxylate transport protein | Bcen2424_0646 |
| BCAL3476 | 0.35 | 2E-04 | BCAL3476 | putative type-b cytochrome | No homolog |
| BCAM0498 | 0.03 | 9E-07 | BCAM0498 | putative aldehyde dehydrogenase | Bcen2424_3462 |
| BCAM0557 | 0.33 | 4E-07 | BCAM0557 | putative monooxygenase | Bcen2424_3531 |
| BCAM0850 | 0.06 | 5E-08 | BCAM0850 | putative rubyerythrin | Bcen2424_3815 |
| BCAM0851 | 0.04 | 6E-04 | BCAM0851 | hypothetical protein | Bcen2424_3816 |
| BCAM0905 | 0.34 | 9E-08 | BCAM0905 | putative NADH dehydrogenase | No homolog |
| BCAM1017_J_1 | 0.20 | 6E-10 | BCAM1017 | formate dehydrogenase, major subunit* | Bcen2424_3984 |
| BCAM1019 | 0.07 | 4E-07 | BCAM1019 | formate dehydrogenase, iron-sulfur subunit | Bcen2424_3985 |
| BCAM1020 | 0.50 | 8E-05 | BCAM1020 | formate dehydrogenase, cytochrome b556 (FDN) subunit | Bcen2424_3986 |
| BCAM1140 | 0.05 | 1E-06 | BCAM1140 | putative aldehyde oxidase/xanthine dehydrogenase | No homolog |
| BCAM1147 | 0.07 | 1E-07 | BCAM1147 | isoquinoline 1-oxidoreductase alpha subunit | Bcen2424_4043 |
| BCAM1148 | 0.18 | 2E-10 | BCAM1148 | putative cytochrome c | Bcen2424_4044 |
| BCAM1174 | 0.37 | 3E-04 | BCAM1174 | putative molybdopterin oxidoreductase | No homolog |
| BCAM1358 | 0.35 | 4E-05 | BCAM1358 | gluconate 2-dehydrogenase cytochrome c subunit* | Bcen2424_4225 |
| BCAM1408 | 0.25 | 3E-10 | BCAM1408 | xenobiotic reductase | Bcen2424_4273 |
| BCAM1442 | 0.32 | 6E-06 | BCAM1442 | putative methylmalonate-semialdehyde dehydrogenase | Bcen2424_4314 |
| BCAM1539 | 0.28 | 5E-03 | BCAM1539 | putative dehydrogenase, iron-sulfur cluster subunit | Multiple hits |
| BCAM1581 | 0.35 | 5E-09 | BCAM1581 | phosphoenolpyruvate carboxykinase (GTP) | Bcen2424_4445 |
| BCAM1685 | 0.01 | 5E-06 | BCAM1685 | putative nitrite reductase (NAD(P)H) | No homolog |
| BCAM1734 | 0.28 | 6E-12 | BCAM1734 | putative cytochrome C | Bcen2424_4552 |
| BCAM1967 | 0.22 | 1E-11 | BCAM1967 | putative xenobiotic reductase | Bcen2424_4764 |
| BCAM2132 | 0.20 | 6E-05 | BCAM2132 | 2-aminomuconate 6-semialdehyde dehydrogenase | No homolog |
| BCAM2212 | 0.43 | 1E-05 | BCAM2212 | 2Fe-2S iron-sulfur | Bcen2424_4980 |
| BCAM2318 | 0.50 | 2E-02 | BCAM2318 | putative ferredoxin oxidoreductase protein | Bcen2424_5077 |
| BCAM2321 | 0.31 | 2E-02 | BCAM2321 | putative electron transfer flavoprotein alpha subunit | Bcen2424_5080 |
| BCAM2322 | 0.35 | 4E-03 | BCAM2322 | putative iron-sulfur cluster membrane protein | Bcen2424_5081 |
| BCAM2323 | 0.23 | 6E-03 | BCAM2323 | putative N-methylproline demethylase | No homolog |
| BCAM2468 | 0.38 | 5E-05 | BCAM2468 | putative aldehyde dehydrogenase family protein | No homolog |
| BCAM2701 | 0.38 | 5E-12 | BCAM2701 | aconitate hydratase 1 | Bcen2424_5515 |
| BCAS0021 | 0.41 | 1E-05 | BCAS0021 | putative CoA-transferase | No homolog |
| BCAS0201 | 0.39 | 5E-03 | BCAS0201 | putative FAD dependent oxidoreductase | Bcen2424_6023 |
| BCAS0241 | 0.21 | 5E-06 | BCAS0241 | putative sodium:dicarboxylate symporter family protein | Bcen2424_5970 |
| BCAS0277 | 0.39 | 7E-05 | BCAS0277 | putative aldehyde dehydrogenase | Bcen2424_5904 |
| BCAS0319 | 0.07 | 2E-07 | BCAS0319 | putative oxidoreductase* | No homolog |
| BCAS0320 | 0.07 | 5E-08 | BCAS0320 | isoquinoline 1-oxidoreductase alpha subunit | Bcen2424_6768 |
| BCAS0396 | 0.35 | 6E-05 | BCAS0396 | putative dehydrogenase | No homolog |
| IG2_936658 | 0.06 | 6E-08 | BCAM0851 | hypothetical protein | Bcen2424_3816 |
|  |  |  |  | ***Carbohydrate transport and metabolism (G)*** |  |
| BCAL0121 | 0.32 | 5E-05 | BCAL0121 | aquaporin Z* | Bcen2424_0248 |
| BCAL0476 | 0.45 | 6E-04 | BCAL0476 | HpcH/HpaI aldolase/citrate lyase family protein | Bcen2424_3116 |
| BCAL0543 | 0.11 | 3E-09 | BCAL0543 | Major Facilitator Superfamily protein | Bcen2424_3046 |
| BCAL0618 | 0.11 | 2E-09 | BCAL0618 | PfkB family carbohydrate kinase | No homolog |
| BCAL0619 | 0.08 | 2E-08 | BCAL0619 | putative N-acylglucosamine 2-epimerase | Bcen2424_2973 |
| BCAL0735 | 0.46 | 6E-07 | BCAL0735 | phosphocarrier protein HPr | Bcen2424_2861 |
| BCAL0950 | 0.29 | 4E-08 | BCAL0950 | Major Facilitator Superfamily protein | Bcen2424_2655 |
| BCAL1148 | 0.31 | 7E-07 | BCAL1148 | putative polysaccharide deacetylase | No homolog |
| BCAL1289 | 0.32 | 3E-08 | BCAL1289 | aldose 1-epimerase | Bcen2424_1318 |
| BCAL1431 | 0.49 | 7E-05 | BCAL1431 | putative ribose ABC transport system, substrate-binding exported protein | Bcen2424_1424 |
| BCAL1451 | 0.39 | 2E-09 | BCAL1451 | putative fosmidomycin resistance protein* | Bcen2424_1444 |
| BCAL1543 | 0.32 | 2E-08 | BCAL1543 | Major Facilitator Superfamily protein | No homolog |
| BCAL1548 | 0.27 | 1E-03 | BCAL1548 | putative sugar ABC transport system, lipoprotein | Bcen2424_1541 |
| BCAL1550 | 0.19 | 4E-08 | BCAL1550 | putative sugar ABC transporter ATP-binding protein | Bcen2424_1543 |
| BCAL1640 | 0.39 | 5E-04 | BCAL1640 | Major Facilitator Superfamily protein | Bcen2424_1589 |
| BCAL1657 | 0.49 | 8E-03 | BCAL1657 | putative ribose transport system, substrate-binding protein* | Bcen2424_1606 |
| BCAL1804 | 0.09 | 1E-08 | BCAL1804 | Major Facilitator Superfamily protein | Bcen2424_1732 |
| BCAL1805 | 0.02 | 4E-07 | BCAL1805 | putative sugar kinase | Bcen2424_1733 |
| BCAL1806 | 0.02 | 4E-07 | BCAL1806 | hypothetical protein | Bcen2424_1734 |
| BCAL2040 | 0.15 | 3E-09 | BCAL2040 | polysaccharide deacetylase | Bcen2424_1963 |
| BCAL2251 | 0.21 | 3E-09 | BCAL2251 | Major Facilitator Superfamily protein | Bcen2424_2177 |
| BCAL2419 | 0.33 | 2E-12 | BCAL2419 | glycosyl hydrolases family protein | Bcen2424_2323 |
| BCAL2472 | 0.33 | 7E-05 | BCAL2472 | alpha,alpha-trehalose-phosphate synthase (UDP-forming) | Bcen2424_2393 |
| BCAL2545 | 0.42 | 2E-11 | BCAL2545 | Major Facilitator Superfamily protein | No homolog |
| BCAL2801 | 0.41 | 4E-04 | BCAL2801 | Major Facilitator Superfamily protein | Bcen2424_2588 |
| BCAL3042 | 0.46 | 1E-07 | BCAL3042 | glucose-6-phosphate 1-dehydrogenase | Bcen2424_0967 |
| BCAL3070 | 0.42 | 2E-03 | BCAL3070 | Major Facilitator Superfamily protein | No homolog |
| BCAL3113 | 0.17 | 5E-11 | BCAL3113 | phosphomannomutase | Bcen2424_0893 |
| BCAL3181 | 0.37 | 2E-04 | BCAL3181 | Major Facilitator Superfamily protein | Bcen2424_0823 |
| BCAL3309 | 0.37 | 4E-09 | BCAL3309 | Major Facilitator Superfamily protein | Bcen2424_0717 |
| BCAL3366 | 0.40 | 7E-12 | BCAL3366 | KHG/KDPG aldolase | Bcen2424_0661 |
| BCAL3408 | 0.45 | 4E-05 | BCAL3408 | putative galactonokinase | Bcen2424_0618 |
| BCAM0087 | 0.07 | 5E-08 | BCAM0087 | putative lipoprotein | Bcen2424_5786 |
| BCAM0363 | 0.27 | 7E-10 | BCAM0363 | putative lipoprotein | No homolog |
| BCAM0364 | 0.27 | 8E-11 | BCAM0364 | putative lipoprotein | Bcen2424_3350 |
| BCAM0577 | 0.04 | 2E-07 | BCAM0577 | Major Facilitator Superfamily protein | No homolog |
| BCAM0783 | 0.01 | 4E-07 | BCAM0783 | Major Facilitator Superfamily protein | Bcen2424_3742 |
| BCAM0881 | 0.19 | 4E-09 | BCAM0881 | putative alpha amylase-family protein | Bcen2424_3849 |
| BCAM1138 | 0.05 | 1E-07 | BCAM1138 | Major Facilitator Superfamily protein | Bcen2424_4034 |
| BCAM1326 | 0.04 | 1E-06 | BCAM1326 | putative beta-glucosidase | Bcen2424_4195 |
| BCAM1521 | 0.16 | 6E-10 | BCAM1521 | shikimate transporter* | Bcen2424_4394 |
| BCAM1973 | 0.35 | 4E-04 | BCAM1973 | putative ABC transporter, substrate-binding protein | Bcen2424_4775 |
| BCAM2113 | 0.18 | 1E-03 | BCAM2113 | Major Facilitator Superfamily protein | No homolog |
| BCAM2260 | 0.48 | 7E-08 | BCAM2260 | Major Facilitator Superfamily protein | No homolog |
| BCAM2337 | 0.08 | 6E-07 | BCAM2337 | putative multidrug resistance transporter protein | Bcen2424_5094 |
| BCAM2338 | 0.03 | 7E-07 | BCAM2338 | putative glycosyltransferase | Bcen2424_5095 |
| BCAM2500 | 0.09 | 4E-08 | BCAM2500 | putative glucarate transporter | No homolog |
| BCAM2545 | 0.21 | 9E-06 | BCAM2545 | Major Facilitator Superfamily protein | No homolog |
| BCAM2703 | 0.30 | 1E-03 | BCAM2703 | probable methylisocitrate lyase | No homolog |
| BCAS0011 | 0.41 | 3E-10 | BCAS0011 | hypothetical protein | Bcen2424_6184 |
| BCAS0122 | 0.13 | 1E-07 | BCAS0122 | putative transporter protein | No homolog |
| BCAS0148 | 0.28 | 2E-03 | BCAS0148 | hypothetical protein | No homolog |
| BCAS0254 | 0.31 | 3E-12 | BCAS0254 | Major Facilitator Superfamily protein | No homolog |
|  |  |  |  | ***Amino acid transport and metabolism (E)*** |  |
| BCAL0010 | 0.01 | 7E-07 | BCAL0010 | phenylalanine-4-hydroxylase | Bcen2424_0078 |
| BCAL0015 | 0.27 | 9E-12 | BCAL0015 | putative branched-chain amino acid ABC transporter ATP-binding protein | Bcen2424_0083 |
| BCAL0016 | 0.41 | 6E-11 | BCAL0016 | putative branched-chain amino acid ABC transporter ATP-binding protein | No homolog |
| BCAL0058 | 0.07 | 1E-07 | BCAL0058 | putative ethanolamine permease | Bcen2424_0128 |
| BCAL0059 | 0.21 | 3E-11 | BCAL0059 | ethanolamine ammonia-lyase heavy chain | Bcen2424_0129 |
| BCAL0060 | 0.34 | 3E-05 | BCAL0060 | ethanolamine ammonia-lyase light chain | Bcen2424_0130 |
| BCAL0151 | 0.02 | 3E-07 | BCAL0151 | extracellular ligand binding protein | Bcen2424_0278 |
| BCAL0289 | 0.21 | 4E-11 | BCAL0289 | glutamate synthase large subunit | Bcen2424_0402 |
| BCAL0290 | 0.27 | 4E-12 | BCAL0290 | glutamate synthase small subunit | Bcen2424_0403 |
| BCAL0544 | 0.26 | 2E-09 | BCAL0544 | putative periplasmic dipeptide transport protein* | Bcen2424_3045 |
| BCAL0592 | 0.42 | 2E-09 | BCAL0592 | putative oxidoreductase | Bcen2424_3002 |
| BCAL0593 | 0.36 | 3E-05 | BCAL0593 | putative oxidoreductase | Bcen2424_3001 |
| BCAL0595 | 0.06 | 1E-07 | BCAL0595 | putrescine transport system permease protein | Bcen2424_2999 |
| BCAL0596 | 0.18 | 4E-09 | BCAL0596 | putrescine ABC transport system, permease protein | No homolog |
| BCAL0597 | 0.09 | 4E-08 | BCAL0597 | putrescine ABC transporter ATP-binding protein | Bcen2424_2997 |
| BCAL0598 | 0.02 | 2E-04 | BCAL0598 | putrescine ABC transport system, binding exported protein* | No homolog |
| BCAL0600 | 0.02 | 1E-04 | BCAL0600 | putative glutamine synthetase | Bcen2424_2994 |
| BCAL0658 | 0.39 | 4E-05 | BCAL0658 | allophanate hydrolase | No homolog |
| BCAL0659 | 0.45 | 5E-06 | BCAL0659 | allophanate hydrolase | No homolog |
| BCAL0729 | 0.01 | 8E-07 | BCAL0729 | nitrogen regulatory protein P-II 1 | Bcen2424_2866 |
| BCAL0791 | 0.45 | 3E-05 | BCAL0791 | glycosyl transferase family protein | Bcen2424_2811 |
| BCAL1045 | 0.13 | 6E-08 | BCAL1045 | periplasmic ligand binding lipoprotein | Bcen2424_1165 |
| BCAL1429 | 0.45 | 5E-04 | BCAL1429 | putative TPP-binding acetolactate synthase | No homolog |
| BCAL1641 | 0.25 | 2E-09 | BCAL1641 | metallo peptidase, subfamily M20A | Bcen2424_1590 |
| BCAL2041 | 0.01 | 3E-04 | BCAL2041 | hydantoin racemase | No homolog |
| BCAL2224 | 0.06 | 7E-08 | BCAL2224 | glutamine synthetase | Bcen2424_2149 |
| BCAL2238 | 0.33 | 1E-12 | BCAL2238 | putative glutamine synthetase | Bcen2424_2163 |
| BCAL2247 | 0.13 | 7E-09 | BCAL2247 | periplasmic glutamate/aspartate-binding protein | No homolog |
| BCAL2672 | 0.33 | 9E-12 | BCAL2672 | putative dihydroxy-acid dehydratase | Bcen2424_2462 |
| BCAL2705 | 0.14 | 5E-10 | BCAL2705 | ABC transporter ATP-binding protein | No homolog |
| BCAL2706 | 0.13 | 7E-09 | BCAL2706 | ABC transporter ATP-binding protein | Bcen2424_2495 |
| BCAL2707 | 0.22 | 6E-06 | BCAL2707 | putative transport system permease protein | Bcen2424_2496 |
| BCAL2708 | 0.10 | 2E-08 | BCAL2708 | putative amino-acid transport permease protein | Bcen2424_2497 |
| BCAL2791 | 0.33 | 3E-11 | BCAL2791 | putative kynureninase | Bcen2424_2578 |
| BCAL3098 | 0.01 | 8E-07 | BCAL3098 | hypothetical protein* | Bcen2424_0910 |
| BCAL3099 | 0.12 | 1E-08 | BCAL3099 | putative branched-chain amino acid transport system, permease component | Bcen2424_0909 |
| BCAL3100 | 0.01 | 5E-07 | BCAL3100 | putative branched-chain amino acid transport system, permease component | Bcen2424_0908 |
| BCAL3102 | 0.01 | 1E-06 | BCAL3102 | ABC transporter ATP-binding protein | No homolog |
| BCAL3104 | 0.01 | 6E-07 | BCAL3104 | urease gamma subunit | Bcen2424_0902 |
| BCAL3105 | 0.02 | 4E-07 | BCAL3105 | urease beta subunit | Bcen2424_0901 |
| BCAL3106 | 0.02 | 4E-07 | BCAL3106 | urease alpha subunit | Bcen2424_0900 |
| BCAL3164 | 0.34 | 4E-03 | BCAL3164 | serine peptidase, family S10* | Bcen2424_0841 |
| BCAL3282 | 0.29 | 6E-04 | BCAL3282 | putative phospho-2-dehydro-3-deoxyheptonate aldolase | Bcen2424_0738 |
| BCAL3367 | 0.28 | 2E-06 | BCAL3367 | phosphogluconate dehydratase | Bcen2424_0660 |
| BCAL3390 | 0.33 | 1E-12 | BCAL3390 | putative spermidine synthase | No homolog |
| BCAL3411 | 0.17 | 3E-09 | BCAL3411 | serine peptidase, family S10 | Bcen2424_0615 |
| BCAM0016 | 0.39 | 2E-12 | BCAM0016 | tartrate dehydrogenase | Bcen2424_5707 |
| BCAM0072 | 0.34 | 2E-04 | BCAM0072 | putative thiamine pyrophosphate enzyme | No homolog |
| BCAM0410 | 0.41 | 4E-04 | BCAM0410 | putative ABC-type glycine betaine transport protein | No homolog |
| BCAM0452 | 0.21 | 4E-07 | BCAM0452 | serine peptidase, family S10* | Bcen2424_3420 |
| BCAM0459 | 0.28 | 4E-03 | BCAM0459 | cysteine desulfurase | Bcen2424_3426 |
| BCAM0508 | 0.21 | 1E-10 | BCAM0508 | putative periplasmic binding protein* | Bcen2424_3474 |
| BCAM0509 | 0.45 | 1E-05 | BCAM0509 | putative FAD dependent oxidoreductase | Bcen2424_3475 |
| BCAM0633 | 0.02 | 5E-07 | BCAM0633 | hypothetical protein | Bcen2424_3604 |
| BCAM0672 | 0.39 | 3E-11 | BCAM0672 | ABC transporter ATP-binding protein | No homolog |
| BCAM0760 | 0.39 | 7E-04 | BCAM0760 | histidine transport system permease | Bcen2424_3725 |
| BCAM0762 | 0.45 | 2E-08 | BCAM0762 | histidine ABC transporter ATP-binding protein | Bcen2424_3727 |
| BCAM0874 | 0.05 | 1E-07 | BCAM0874 | periplasmic solute-binding protein | Bcen2424_3842 |
| BCAM0952 | 0.25 | 3E-11 | BCAM0952 | ABC transporter ATP-binding protein | Bcen2424_3918 |
| BCAM0953 | 0.30 | 5E-12 | BCAM0953 | extracellular solute-binding protein* | Bcen2424_3919 |
| BCAM0954 | 0.29 | 3E-07 | BCAM0954 | binding-protein-dependent transport system protein | Bcen2424_3920 |
| BCAM1142 | 0.34 | 5E-07 | BCAM1142 | hypothetical protein | Bcen2424_4039 |
| BCAM1208 | 0.24 | 1E-06 | BCAM1208 | periplasmic glutamine-binding protein | Bcen2424_4082 |
| BCAM1209 | 0.34 | 2E-08 | BCAM1209 | glutamine ABC transporter, permease protein | Bcen2424_4083 |
| BCAM1235 | 0.01 | 3E-06 | BCAM1235 | transglutaminase-like protein | Bcen2424_4110 |
| BCAM1238 | 0.25 | 1E-04 | BCAM1238 | transglutaminase-like protein* | Bcen2424_4113 |
| BCAM1266 | 0.33 | 1E-11 | BCAM1266 | putative dihydrodipicolinate synthase | Bcen2424_4139 |
| BCAM1291 | 0.47 | 1E-05 | BCAM1291 | L-asparaginase | Bcen2424_4161 |
| BCAM1353 | 0.16 | 4E-09 | BCAM1353 | alanine dehydrogenase | Bcen2424_4220 |
| BCAM1357 | 0.36 | 6E-12 | BCAM1357 | gluconate 2-dehydrogenase flavoprotein subunit | Bcen2424_4224 |
| BCAM1450 | 0.35 | 1E-09 | BCAM1450 | beta-alanine specific aminopeptidase | No homolog |
| BCAM1485 | 0.36 | 1E-12 | BCAM1485 | putative ornithine cyclodeaminase | Bcen2424_4357 |
| BCAM1488 | 0.49 | 6E-10 | BCAM1488 | putative proline racemase | Bcen2424_4360 |
| BCAM1522 | 0.17 | 5E-10 | BCAM1522 | putative dioxygenase | Bcen2424_4395 |
| BCAM1743 | 0.10 | 1E-08 | BCAM1743 | periplasmic solute-binding protein | Bcen2424_4562 |
| BCAM1770 | 0.10 | 6E-09 | BCAM1770 | putative ABC transporter, substrate-binding protein | No homolog |
| BCAM1771 | 0.17 | 2E-10 | BCAM1771 | putative ABC transporter, permease protein | No homolog |
| BCAM1772 | 0.27 | 3E-05 | BCAM1772 | putative ABC transporter, permease protein | Bcen2424_4593 |
| BCAM1773 | 0.21 | 2E-10 | BCAM1773 | ABC transporter ATP-binding protein | Bcen2424_4594 |
| BCAM1774 | 0.34 | 3E-08 | BCAM1774 | ABC transporter ATP-binding protein | No homolog |
| BCAM1846 | 0.27 | 8E-10 | BCAM1846 | putative thiamine pyrophosphate binding protein | Bcen2424_4703 |
| BCAM1977 | 0.35 | 8E-06 | BCAM1977 | putative amino acid permease | Bcen2424_4779 |
| BCAM2044 | 0.04 | 1E-06 | BCAM2044 | putative asparagine synthetase | Bcen2424_4843 |
| BCAM2094 | 0.11 | 3E-09 | BCAM2094 | putative gamma-glutamylputrescine synthetase | Bcen2424_4901 |
| BCAM2096 | 0.13 | 1E-09 | BCAM2096 | putative gamma-glutamylputrescine oxidoreductase | Bcen2424_4903 |
| BCAM2125 | 0.20 | 3E-06 | BCAM2125 | indole-3-glycerol phosphate synthase | No homolog |
| BCAM2237 | 0.22 | 2E-11 | BCAM2237 | putative 2,2-dialkylglycine decarboxylase | Bcen2424_5005 |
| BCAM2295 | 0.29 | 2E-04 | BCAM2295 | putative solute-binding component of ABC transporter | Bcen2424_5052 |
| BCAM2325 | 0.06 | 2E-06 | BCAM2325 | putative dipeptidase | Bcen2424_5084 |
| BCAM2326 | 0.09 | 1E-04 | BCAM2326 | putative serine hydroxymethyltransferase | No homolog |
| BCAM2333 | 0.45 | 1E-02 | BCAM2333 | putative glutathione-independent formaldehyde dehydrogenase | Bcen2424_5090 |
| BCAM2389 | 0.41 | 3E-05 | BCAM2389 | putative sarcosine oxidase alpha subunit* | Bcen2424_5149 |
| BCAM2390 | 0.29 | 6E-06 | BCAM2390 | putative sarcosine oxidase delta subunit | No homolog |
| BCAM2391 | 0.34 | 7E-12 | BCAM2391 | putative sarcosine oxidase beta subunit | Bcen2424_5151 |
| BCAM2407 | 0.48 | 3E-02 | BCAM2407 | putative glycine betaine/L-proline-binding protein ABC transporter component | No homolog |
| BCAM2409 | 0.24 | 5E-03 | BCAM2409 | putative glycine-betaine binding ABC transporter permease protein | Bcen2424_5171 |
| BCAM2410 | 0.36 | 3E-02 | BCAM2410 | putative glycine betaine/L-proline ABC transporter ATP-binding protein | Bcen2424_5172 |
| BCAM2447 | 0.45 | 6E-11 | BCAM2447 | putative LysE family transporter | Bcen2424_5250 |
| BCAM2477 | 0.45 | 1E-04 | BCAM2477 | serine peptidase, family S10 | Bcen2424_5279 |
| BCAM2499 | 0.45 | 3E-06 | BCAM2499 | putative aminotransferase protein | Bcen2424_5301 |
| BCAM2501 | 0.03 | 2E-07 | BCAM2501 | shikimate 5-dehydrogenase | Bcen2424_5303 |
| BCAM2502 | 0.02 | 3E-04 | BCAM2502 | 3-dehydroquinate dehydratase | No homolog |
| BCAM2618 | 0.04 | 3E-07 | BCAM2618 | putative periplasmic lysine-arginine-ornithine-binding protein | No homolog |
| BCAM2725 | 0.44 | 3E-08 | BCAM2725 | putative oligopeptide ABC transporter ATP-binding protein | Bcen2424_5539 |
| BCAS0110 | 0.03 | 9E-07 | BCAS0110 | periplasmic solute-binding protein | No homolog |
| BCAS0111 | 0.04 | 1E-05 | BCAS0111 | putative binding-protein-dependent transport system component | Bcen2424_6114 |
| BCAS0112 | 0.12 | 2E-09 | BCAS0112 | putative binding-protein-dependent transport system component | No homolog |
| BCAS0113 | 0.05 | 9E-07 | BCAS0113 | ABC transporter ATP-binding protein | Bcen2424_6112 |
| BCAS0114 | 0.08 | 5E-08 | BCAS0114 | putative histidine ammonia-lyase | Bcen2424_6111 |
| BCAS0136 | 0.42 | 5E-05 | BCAS0136 | putative amino acid permease | Bcen2424_6096 |
| BCAS0140 | 0.17 | 3E-10 | BCAS0140 | hypothetical protein* | Bcen2424_6092 |
| BCAS0141 | 0.34 | 6E-10 | BCAS0141 | putative transport system component | Bcen2424_6091 |
| BCAS0142 | 0.31 | 3E-11 | BCAS0142 | putative amino-acid transport system component | Bcen2424_6090 |
| BCAS0143 | 0.23 | 1E-08 | BCAS0143 | ABC transporter ATP-binding protein | Bcen2424_6089 |
| BCAS0144 | 0.20 | 1E-10 | BCAS0144 | ABC transporter ATP-binding protein | Bcen2424_6088 |
| BCAS0145 | 0.16 | 3E-10 | BCAS0145 | metallo peptidase, family M20 unassigned | Bcen2424_6087 |
| BCAS0211 | 0.41 | 1E-03 | BCAS0211 | putative pyridoxal-dependent decarboxylase | Bcen2424_6013 |
| BCAS0240 | 0.19 | 3E-10 | BCAS0240 | periplasmic solute-binding protein | Bcen2424_5971 |
| BCAS0276 | 0.07 | 2E-07 | BCAS0276 | hypothetical protein | Bcen2424_5919 |
| BCAS0388 | 0.19 | 2E-10 | BCAS0388 | putative periplasmic solute-binding protein* | Bcen2424_6679 |
| BCAS0389 | 0.36 | 2E-03 | BCAS0389 | putative binding-protein-dependent transport system component | Bcen2424_6678 |
| BCAS0390 | 0.49 | 2E-04 | BCAS0390 | putative binding-protein-dependent transport system component | No homolog |
| BCAS0395 | 0.45 | 8E-06 | BCAS0395 | putative FAD-dependent oxidoreductase | Bcen2424_6672 |
| BCAS0577 | 0.01 | 5E-07 | BCAS0577 | periplasmic solute-binding protein | Bcen2424_6331 |
| BCAS0578 | 0.45 | 4E-03 | BCAS0578 | L-asparagine permease | Bcen2424_6330 |
| BCAS0734 | 0.12 | 2E-08 | BCAS0734 | putative pyridine nucleotide-disulphide oxidoreductase family protein | No homolog |
| BCAS0735 | 0.06 | 6E-08 | BCAS0735 | metallo peptidase, family M20 unassigned | Bcen2424_6234 |
| BCAS0751 | 0.14 | 4E-09 | BCAS0751 | putative gamma-glutamyltransferase precursor | Bcen2424_6219 |
| IG2_1360226 | 0.23 | 2E-11 | BCAM1238 | transglutaminase-like protein* | No homolog |
| BCAL0437 | 0.39 | 7E-09 | BCAL0437 | O6-methylguanine-DNA methyltransferase | Bcen2424_3152 |
| BCAL2038 | 0.17 | 6E-11 | BCAL2038 | putative allantoicase | No homolog |
|  |  |  |  | ***Nucleotide transport and metabolism (F)*** |  |
| BCAL2042 | 0.02 | 4E-06 | BCAL2042 | putative transport-related membrane protein | No homolog |
| BCAL2836 | 0.49 | 2E-03 | BCAL2836 | phosphoribosylaminoimidazole carboxylase ATPase subunit | Bcen2424_2623 |
| BCAL3172 | 0.18 | 2E-10 | BCAL3172 | putative xanthine dehydrogenase large subunit | Bcen2424_0833 |
| BCAL3379 | 0.36 | 2E-11 | BCAL3379 | putative ureidoglycolate hydrolase | Bcen2424_0648 |
| BCAL3380 | 0.31 | 3E-12 | BCAL3380 | putative allantoicase | Bcen2424_0647 |
| BCAM1457 | 0.50 | 1E-08 | BCAM1457 | cytosine deaminase | Bcen2424_4328 |
| BCAS0728 | 0.36 | 6E-07 | BCAS0728 | cytidine deaminase | No homolog |
| BCAS0731 | 0.05 | 2E-07 | BCAS0731 | D-hydantoinase | Bcen2424_6238 |
| BCAS0732 | 0.08 | 4E-08 | BCAS0732 | putative cytosine/purines, uracil, thiamine, allantoin permease | No homolog |
| BCAS0733 | 0.31 | 5E-08 | BCAS0733 | putative dihydroorotate dehydrogenase family protein | No homolog |
|  |  |  |  | ***Coenzyme transport and metabolism (H)*** |  |
| BCAL0009 | 0.06 | 1E-07 | BCAL0009 | pterin-4-alpha-carbinolamine dehydratase | Bcen2424_0077 |
| BCAL0599 | 0.04 | 7E-07 | BCAL0599 | putative aminotransferase | Bcen2424_2995 |
| BCAL0732 | 0.49 | 1E-08 | BCAL0732 | glutathione synthetase | Bcen2424_2863 |
| BCAL2237 | 0.28 | 2E-10 | BCAL2237 | putative aminotransferase | Bcen2424_2162 |
| BCAL2751 | 0.45 | 1E-05 | BCAL2751 | putative ketopantoate reductase | No homolog |
| BCAL2923 | 0.32 | 4E-12 | BCAL2923 | putative cobalamin biosynthesis membrane protein | No homolog |
| BCAL3094 | 0.43 | 9E-06 | BCAL3094 | oxygen-independent coproporphyrinogen III oxidase | Bcen2424_0914 |
| BCAL3472 | 0.26 | 4E-12 | BCAL3472 | hypothetical protein | Bcen2424_0549 |
| BCAM0055 | 0.49 | 4E-03 | BCAM0055 | GTP cyclohydrolase I | Bcen2424_5743 |
| BCAM0057 | 0.10 | 6E-08 | BCAM0057 | p-hydroxybenzoate hydroxylase | No homolog |
| BCAM1146 | 0.39 | 3E-12 | BCAM1146 | putative flavoprotein monooxygenase | Bcen2424_4042 |
| BCAM1441 | 0.20 | 1E-09 | BCAM1441 | omega-amino-acid-pyruvate aminotransferase | Bcen2424_4313 |
| BCAM1687 | 0.01 | 8E-07 | BCAM1687 | putative uroporphyrin-III C-methyltransferase | Bcen2424_4509 |
| BCAM2362 | 0.15 | 7E-05 | BCAM2362 | coenzyme PQQ synthesis protein C | Bcen2424_5121 |
| BCAS0137 | 0.48 | 2E-04 | BCAS0137 | putative aminotransferase | Bcen2424_6095 |
| BCAS0206 | 0.33 | 8E-05 | BCAS0206 | putative methyltransferase family protein | Bcen2424_6018 |
| BCAS0253 | 0.33 | 5E-13 | BCAS0253 | putative ketopantoate reductase family protein | No homolog |
|  |  |  |  | ***Lipid transport and metabolism (I)*** |  |
| BCAL0046 | 0.47 | 2E-05 | BCAL0046 | putative fatty-acid CoA ligase | Bcen2424_0116 |
| BCAL0047 | 0.48 | 2E-04 | BCAL0047 | putative acyl-CoA dehydrogenase | Bcen2424_0117 |
| BCAL0660 | 0.38 | 2E-09 | BCAL0660 | biotin carboxylase | No homolog |
| BCAL0832 | 0.46 | 3E-03 | BCAL0832 | putative poly-beta-hydroxy-butyrate storage protein | No homolog |
| BCAL1249 | 0.24 | 2E-11 | BCAL1249 | putative PHB depolymerase | Bcen2424_1278 |
| BCAL1862 | 0.24 | 5E-12 | BCAL1862 | acetyl-CoA acetyltransferase | Bcen2424_1790 |
| BCAM0058 | 0.22 | 7E-13 | BCAM0058 | 3-oxoadipate CoA-transferase subunit A | Bcen2424_5746 |
| BCAM0059 | 0.29 | 5E-06 | BCAM0059 | 3-oxoadipate CoA-transferase subunit B | Bcen2424_5747 |
| BCAM0070 | 0.26 | 2E-11 | BCAM0070 | putative hydrolase | No homolog |
| BCAM0388 | 0.47 | 2E-08 | BCAM0388 | putative phospholipid n-methyltransferase | No homolog |
| BCAM0644 | 0.43 | 4E-10 | BCAM0644 | putative acyl-CoA dehydrogenase protein | Bcen2424_3615 |
| BCAM0774 | 0.01 | 6E-05 | BCAM0774 | poly[D-(-)-3-hydroxybutyrate] depolymerase | Bcen2424_3731 |
| BCAM1005 | 0.02 | 2E-06 | BCAM1005 | putative acyltransferase | No homolog |
| BCAM1009 | 0.01 | 4E-07 | BCAM1009 | putative acyltransferase | Bcen2424_3975 |
| BCAM1011 | 0.05 | 3E-06 | BCAM1011 | putative acetyltransferase | Bcen2424_3978 |
| BCAM1241 | 0.27 | 2E-04 | BCAM1241 | putative acyltransferase | Bcen2424_4116 |
| BCAM1595 | 0.38 | 4E-04 | BCAM1595 | putative esterase | No homolog |
| BCAM2067 | 0.18 | 1E-07 | BCAM2067 | putative undecaprenyl pyrophosphate synthetase | No homolog |
| BCAM2114 | 0.09 | 2E-04 | BCAM2114 | putative hydroxylase | No homolog |
| BCAM2119 | 0.43 | 2E-02 | BCAM2119 | carboxylesterase | No homolog |
| BCAM2134 | 0.21 | 1E-04 | BCAM2134 | hypothetical protein | No homolog |
| BCAM2136 | 0.14 | 6E-10 | BCAM2136 | putative crotonobetaine/carnitine-CoA ligase | No homolog |
| BCAM2195 | 0.42 | 9E-12 | BCAM2195 | putative AMP-binding enzyme | Bcen2424_4962 |
| BCAS0208 | 0.38 | 3E-03 | BCAS0208 | putative acyl-CoA dehydrogenase | Bcen2424_6016 |
| BCAS0209 | 0.45 | 3E-03 | BCAS0209 | hypothetical protein | Bcen2424_6015 |
| BCAS0210 | 0.42 | 6E-03 | BCAS0210 | putative AMP-binding enzyme | Bcen2424_6014 |
| BCAS0212 | 0.36 | 2E-03 | BCAS0212 | hypothetical protein | Bcen2424_6012 |
| BCAS0222 | 0.42 | 9E-04 | BCAS0222 | putative AMP-dependent synthetase | Bcen2424_6002 |
| BCAS0272 | 0.05 | 8E-07 | BCAS0272 | urea amidolyase, urea carboxylase subunit | Bcen2424_5923 |
| BCAS0737 | 0.36 | 2E-06 | BCAS0737 | putative acetyl-CoA acetyltransferase | Bcen2424_6232 |
| BCAS0739 | 0.41 | 6E-06 | BCAS0739 | putative acetyl-CoA synthetase | Bcen2424_6230 |
|  |  |  |  | ***Inorganic ion transport and metabolism (P)*** |  |
| BCAL0155 | 0.06 | 5E-08 | BCAL0155 | putative cation efflux protein | Bcen2424_0282 |
| BCAL0604 | 0.42 | 3E-04 | BCAL0604 | putative cheavy metal binding protein | Bcen2424_2989 |
| BCAL0730 | 0.01 | 3E-05 | BCAL0730 | ammonium transporter family protein* | No homolog |
| BCAL1277 | 0.48 | 7E-04 | BCAL1277 | polyphosphate kinase | Bcen2424_1307 |
| BCAL1319 | 0.02 | 1E-05 | BCAL1319 | putative formate/nitrate transporter | Bcen2424_6120 |
| BCAL2225 | 0.44 | 6E-14 | BCAL2225 | hypothetical protein | Bcen2424_2150 |
| BCAL2288 | 0.24 | 1E-05 | BCAL2288 | bacterioferritin | Bcen2424_2195 |
| BCAL2740 | 0.08 | 3E-08 | BCAL2740 | high-affinity nickel transport protein | Bcen2424_2529 |
| BCAL3297 | 0.45 | 2E-02 | BCAL3297 | putative ferritin DPS-family DNA binding protein | Bcen2424_0728 |
| BCAL3299 | 0.43 | 6E-04 | BCAL3299 | peroxidase/catalase KatB | Bcen2424_0726 |
| BCAL3477 | 0.14 | 6E-10 | BCAL3477 | putative catalase* | Bcen2424_0010 |
| BCAM0437 | 0.41 | 2E-08 | BCAM0437 | putative sodium/calcium exchanger protein | Bcen2424_3404 |
| BCAM0558 | 0.43 | 7E-09 | BCAM0558 | hypothetical protein | Bcen2424_3532 |
| BCAM0648 | 0.47 | 6E-08 | BCAM0648 | putative ring-hydroxylating alpha subunit | Bcen2424_3619 |
| BCAM0810 | 0.12 | 1E-07 | BCAM0810 | putative aromatic oxygenase | Bcen2424_3775 |
| BCAM0907 | 0.26 | 2E-12 | BCAM0907 | putative sulfurtransferase | Bcen2424_3875 |
| BCAM1684 | 0.01 | 2E-06 | BCAM1684 | putative nitrite reductase | Bcen2424_4505 |
| BCAM1686 | 0.01 | 8E-07 | BCAM1686 | putative nitrate transporter | Bcen2424_4507 |
| BCAM1689 | 0.01 | 4E-07 | BCAM1689 | putative nitrate transporter component | Bcen2424_4511 |
| BCAM1725 | 0.49 | 7E-03 | BCAM1725 | putative voltage-gated clc-type chloride channel | Bcen2424_4543 |
| BCAM1852 | 0.26 | 1E-10 | BCAM1852 | [2Fe-2S]-binding protein | No homolog |
| BCAM1854 | 0.48 | 1E-06 | BCAM1854 | [2Fe-2S]-binding protein | No homolog |
| BCAM2135 | 0.27 | 1E-05 | BCAM2135 | Major Facilitator Superfamily protein | No homolog |
| BCAM2319 | 0.17 | 2E-02 | BCAM2319 | iron-sulphur Rieske protein | No homolog |
| BCAS0269 | 0.01 | 2E-05 | BCAS0269 | hypothetical protein* | Bcen2424_5927 |
| BCAS0274 | 0.05 | 1E-07 | BCAS0274 | putative binding-protein-dependent transport system component | Bcen2424_5921 |
| BCAS0275 | 0.02 | 9E-06 | BCAS0275 | ABC transporter ATP-binding protein | Bcen2424_5920 |
| BCAS0349 | 0.34 | 5E-06 | BCAS0349 | ABC transporter ATP-binding protein | Bcen2424_6746 |
| IG1_708173 | 0.28 | 8E-08 | BCAL0651 | sodium/hydrogen exchanger family protein | No homolog |
|  |  |  |  | ***Secondary metabolites biosynthesis transport and catabolism (Q)*** |  |
| BCAL0668 | 0.02 | 1E-06 | BCAL0668 | serine peptidase, family S9 unassigned | Bcen2424_2930 |
| BCAL0833 | 0.15 | 7E-10 | BCAL0833 | putative Acetoacetyl-CoA reductase | Bcen2424_2771 |
| BCAL1772 | 0.40 | 7E-03 | BCAL1772 | isochorismatase family protein | No homolog |
| BCAL1912 | 0.45 | 2E-03 | BCAL1912 | putative acetoin(diacetyl) reductase | Bcen2424_1840 |
| BCAL1914 | 0.46 | 1E-02 | BCAL1914 | acetoin catabolism regulatory protein | Bcen2424_1842 |
| BCAL2689 | 0.18 | 3E-09 | BCAL2689 | putative short-chain type dehydrogenase/reductase | No homolog |
| BCAL2811 | 0.28 | 1E-10 | BCAL2811 | sorbitol dehydrogenase | Bcen2424_2598 |
| BCAL2824 | 0.43 | 4E-05 | BCAL2824 | isochorismatase family protein | Bcen2424_2611 |
| BCAL3406 | 0.47 | 1E-03 | BCAL3406 | putative dehydrogenase* | Bcen2424_0620 |
| BCAM0067 | 0.34 | 2E-08 | BCAM0067 | putative short chain dehydrogenase | No homolog |
| BCAM0528 | 0.23 | 3E-03 | BCAM0528 | putative oxidoreductase/short-chain dehydrogenase* | Bcen2424_3502 |
| BCAM0782 | 0.02 | 7E-07 | BCAM0782 | putative prolidase | Bcen2424_3741 |
| BCAM0804 | 0.48 | 5E-04 | BCAM0804 | catechol 1,2-dioxygenase 1 | Bcen2424_3769 |
| BCAM0811 | 0.23 | 4E-03 | BCAM0811 | putative aromatic oxygenase | Bcen2424_3776 |
| BCAM0894 | 0.37 | 2E-12 | BCAM0894 | poly(3-hydroxyalkanoate) depolymerase C precursor | Bcen2424_3862 |
| BCAM1144 | 0.31 | 1E-11 | BCAM1144 | putative Asp/Glu/Hydantoin racemase | Bcen2424_4041 |
| BCAM1156 | 0.20 | 6E-10 | BCAM1156 | putative isochorismatase | Bcen2424_4052 |
| BCAM1299 | 0.27 | 8E-12 | BCAM1299 | protocatechuate 3,4-dioxygenase alpha chain | Bcen2424_4171 |
| BCAM1300 | 0.35 | 2E-03 | BCAM1300 | protocatechuate 3,4-dioxygenase beta chain | Bcen2424_4172 |
| BCAM1364 | 0.46 | 8E-09 | BCAM1364 | putative NAD dependent epimerase/dehydratase | Bcen2424_4229 |
| BCAM1411 | 0.29 | 7E-05 | BCAM1411 | putative short-chain dehydrogenase | Bcen2424_4276 |
| BCAM1847 | 0.50 | 6E-10 | BCAM1847 | short chain dehydrogenase | Bcen2424_4704 |
| BCAM1851 | 0.37 | 7E-12 | BCAM1851 | short-chain dehydrogenase | Bcen2424_4708 |
| BCAM2122 | 0.48 | 4E-03 | BCAM2122 | acetaldehyde dehydrogenase | No homolog |
| BCAM2123 | 0.29 | 1E-03 | BCAM2123 | putative 4-oxalocrotonate decarboxylase | No homolog |
| BCAM2124 | 0.36 | 1E-03 | BCAM2124 | 2-keto-4-pentenoate hydratase | No homolog |
| BCAM2127 | 0.24 | 3E-03 | BCAM2127 | putative dienelactone hydrolase family protein | No homolog |
| BCAM2153 | 0.17 | 5E-10 | BCAM2153 | putative cytochrome P450 oxidoreductase | No homolog |
| BCAM2211 | 0.16 | 1E-09 | BCAM2211 | putative sigma-54 activated regulatory protein | No homolog |
| BCAM2283 | 0.31 | 4E-08 | BCAM2283 | putative short chain dehydrogenase* | Bcen2424_5040 |
| BCAM2365 | 0.04 | 3E-07 | BCAM2365 | putative signal-transduction and transcriptional regulator Fis and NtrC family protein* | Bcen2424_5123 |
| BCAM2690 | 0.31 | 1E-09 | BCAM2690 | putative thioesterase | Bcen2424_5501 |
| BCAS0265 | 0.47 | 4E-08 | BCAS0265 | subfamily S9C non-peptidase homologue | No homolog |
| BCAS0317 | 0.48 | 3E-04 | BCAS0317 | dienelactone hydrolase family protein | Bcen2424_6771 |
| BCAS0738 | 0.35 | 3E-04 | BCAS0738 | putative short-chain dehydrogenase family protein* | Bcen2424_6231 |
|  |  |  |  | ***General function prediction only (R)*** |  |
| BCAL0154 | 0.42 | 6E-03 | BCAL0154 | histone-like nucleoid-structuring (H-NS) protein | Bcen2424_0281 |
| BCAL0202 | 0.11 | 3E-09 | BCAL0202 | putative flavoprotein | Bcen2424_0315 |
| BCAL0269 | 0.41 | 3E-04 | BCAL0269 | putative oxidoreductase* | Bcen2424_0383 |
| BCAL0477 | 0.36 | 3E-05 | BCAL0477 | hypothetical protein* | No homolog |
| BCAL0541 | 0.08 | 3E-08 | BCAL0541 | putative FAD dependent oxidoreductase | Bcen2424_3048 |
| BCAL0601 | 0.09 | 7E-08 | BCAL0601 | putative gamma-glutamyl-gamma-aminobutyrate hydrolase | Bcen2424_2993 |
| BCAL0622 | 0.41 | 5E-13 | BCAL0622 | putative sodium bile acid symporter family protein | Bcen2424_2970 |
| BCAL0627 | 0.41 | 1E-03 | BCAL0627 | putative hydrolase | Bcen2424_2964 |
| BCAL0662 | 0.38 | 5E-05 | BCAL0662 | LamB/YcsF family protein | No homolog |
| BCAL0808 | 0.31 | 4E-11 | BCAL0808 | P-loop ATPase protein family protein | Bcen2424_2795 |
| BCAL0863 | 0.45 | 3E-10 | BCAL0863 | cysteine peptidase, family C56 | Bcen2424_2743 |
| BCAL1088 | 0.46 | 1E-03 | BCAL1088 | hypothetical protein | Bcen2424_1206 |
| BCAL1248 | 0.22 | 2E-11 | BCAL1248 | putative phosphoribosyl transferase protein | Bcen2424_1277 |
| BCAL1257 | 0.31 | 6E-12 | BCAL1257 | putative gultathione hydrolase | No homolog |
| BCAL1281 | 0.26 | 2E-04 | BCAL1281 | hypothetical protein | Bcen2424_1311 |
| BCAL1538 | 0.38 | 1E-11 | BCAL1538 | Hfq protein | Bcen2424_1531 |
| BCAL1549 | 0.22 | 2E-11 | BCAL1549 | putative sugar ABC transport system, membrane protein | Bcen2424_1542 |
| BCAL1818 | 0.21 | 3E-09 | BCAL1818 | metallo-beta-lactamase superfamily protein | Bcen2424_1745 |
| BCAL1820 | 0.38 | 1E-04 | BCAL1820 | hypothetical protein | Bcen2424_1747 |
| BCAL2030 | 0.36 | 1E-05 | BCAL2030 | putative purine catabolism-related protein | Bcen2424_1957 |
| BCAL2182 | 0.46 | 6E-06 | BCAL2182 | hypothetical protein | Bcen2424_2110 |
| BCAL2239 | 0.19 | 6E-10 | BCAL2239 | putative gamma-glutamyl-gamma-aminobutyrate hydrolase | No homolog |
| BCAL2306 | 0.33 | 2E-12 | BCAL2306 | hypothetical protein | Bcen2424_2214 |
| BCAL2528 | 0.38 | 2E-05 | BCAL2528 | putative hydrolase | No homolog |
| BCAL2536a | 0.22 | 1E-12 | BCAL2536A | putative hydrolase | No homolog |
| BCAL2690 | 0.10 | 3E-08 | BCAL2690 | short chain dehydrogenase | No homolog |
| BCAL2691 | 0.43 | 2E-05 | BCAL2691 | putative hydrolase | Bcen2424_2481 |
| BCAL2790 | 0.24 | 1E-10 | BCAL2790 | putative cyclase | Bcen2424_2577 |
| BCAL2978 | 0.44 | 1E-02 | BCAL2978 | NAD-dependent formate dehydrogenase alpha subunit* | Bcen2424_1031 |
| BCAL3101 | 0.01 | 1E-06 | BCAL3101 | ABC transporter ATP-binding protein | Bcen2424_0907 |
| BCAL3352 | 0.26 | 7E-06 | BCAL3352 | family C44 non-peptidase homologue | Bcen2424_0674 |
| BCAL3509 | 0.08 | 7E-09 | BCAL3509 | LrgA family protein | Bcen2424_0043 |
| BCAM0047 | 0.02 | 5E-07 | BCAM0047 | putative transporter-LysE family | Bcen2424_5735 |
| BCAM0061 | 0.36 | 1E-04 | BCAM0061 | putative 3-oxoadipate enol-lactonase I | Bcen2424_5749 |
| BCAM0638 | 0.15 | 4E-10 | BCAM0638 | 4-hydroxyphenylacetate 3-monooxygenase, reductase component | Bcen2424_3609 |
| BCAM0860 | 0.01 | 6E-07 | BCAM0860 | glycosyltransferase | No homolog |
| BCAM0870 | 0.31 | 6E-09 | BCAM0870 | hypothetical protein | Bcen2424_3837 |
| BCAM0889 | 0.45 | 2E-11 | BCAM0889 | putative molybdenum transport protein | Bcen2424_3857 |
| BCAM1007 | 0.03 | 4E-06 | BCAM1007 | putative polysaccharide biosynthesis protein | Bcen2424_3973 |
| BCAM1051 | 0.22 | 1E-11 | BCAM1051 | putative phage death-on-curing protein | No homolog |
| BCAM1176 | 0.42 | 7E-08 | BCAM1176 | putative dimethyl sulfoxide reductase subunit | No homolog |
| BCAM1407 | 0.23 | 8E-12 | BCAM1407 | DJ-1/PfpI family protein | Bcen2424_4272 |
| BCAM1506 | 0.16 | 4E-10 | BCAM1506 | putative phospholipid-binding lipoprotein | No homolog |
| BCAM1567 | 0.50 | 4E-07 | BCAM1567 | putative hydrolase | Bcen2424_4435 |
| BCAM1605 | 0.43 | 2E-04 | BCAM1605 | putative phenylacetic acid degradation protein | No homolog |
| BCAM1843 | 0.46 | 1E-10 | BCAM1843 | putative dioxygenase | Bcen2424_4700 |
| BCAM1845 | 0.50 | 5E-05 | BCAM1845 | putative dehydrogenase | Bcen2424_4702 |
| BCAM2115 | 0.07 | 1E-07 | BCAM2115 | putative 4-hydroxyphenylacetate 3-monooxygenase, reductase component* | No homolog |
| BCAM2128 | 0.20 | 4E-06 | BCAM2128 | putative short chain dehydrogenase | No homolog |
| BCAM2129 | 0.26 | 3E-04 | BCAM2129 | 2-amino-3-carboxymuconate 6-semialdehyde decarboxylase | No homolog |
| BCAM2133 | 0.17 | 2E-04 | BCAM2133 | hypothetical protein | No homolog |
| BCAM2208 | 0.14 | 1E-09 | BCAM2208 | putative permease protein | Bcen2424_4975 |
| BCAM2238 | 0.44 | 3E-09 | BCAM2238 | putative short-chain dehydrogenase/reductase | No homolog |
| BCAM2324 | 0.38 | 1E-02 | BCAM2324 | hypothetical protein | Bcen2424_5083 |
| BCAM2336 | 0.04 | 7E-07 | BCAM2336 | putative sugar transferase | Bcen2424_5093 |
| BCAM2340 | 0.02 | 4E-07 | BCAM2340 | putative (R)-3-hydroxydecanoyl-ACP:CoA transacylase | Bcen2424_5097 |
| BCAM2358 | 0.31 | 3E-04 | BCAM2358 | putative esterase | No homolog |
| BCAM2363 | 0.16 | 3E-05 | BCAM2363 | coenzyme PQQ synthesis protein B | No homolog |
| BCAM2366 | 0.35 | 4E-03 | BCAM2366 | putative proline iminopeptidase | Bcen2424_5124 |
| BCAM2415 | 0.42 | 3E-12 | BCAM2415 | putative NADPH-dependent FMN reductase* | No homolog |
| BCAM2574 | 0.40 | 3E-03 | BCAM2574 | metallo peptidase, subfamily M20D | Bcen2424_5374 |
| BCAM2619 | 0.24 | 9E-12 | BCAM2619 | succinylglutamate desuccinylase/aspartoacylase family protein | Bcen2424_5425 |
| BCAM2654 | 0.09 | 4E-09 | BCAM2654 | putative acetyltransferase | Bcen2424_5458 |
| BCAM2678 | 0.45 | 2E-11 | BCAM2678 | hypothetical protein | Bcen2424_5490 |
| BCAM2684 | 0.31 | 4E-12 | BCAM2684 | putative acetyltransferase | Bcen2424_5495 |
| BCAS0090 | 0.14 | 6E-10 | BCAS0090 | flavoprotein | Bcen2424_6131 |
| BCAS0091 | 0.39 | 9E-10 | BCAS0091 | putative pirin family protein | Bcen2424_6130 |
| BCAS0190 | 0.32 | 3E-03 | BCAS0190 | putative H-NS family DNA-binding protein | Bcen2424_6035 |
| BCAS0226 | 0.35 | 6E-08 | BCAS0226 | putative hydrolase | No homolog |
| BCAS0267a | 0.02 | 4E-07 | BCAS0267a | putative calcineurin-like phosphoesterase family protein | No homolog |
| BCAS0385 | 0.37 | 5E-03 | BCAS0385 | putative acetyltransferase, GNAT family | Bcen2424_6683 |
| BCAS0386 | 0.13 | 2E-08 | BCAS0386 | putative haloacid dehalogenase-like hydrolase | Bcen2424_6682 |
| BCAS0391 | 0.49 | 6E-07 | BCAS0391 | ABC transporter ATP-binding protein | Bcen2424_6676 |
| BCAS0394 | 0.28 | 3E-05 | BCAS0394 | hypothetical protein | No homolog |
| IG1_1237542 | 0.45 | 7E-05 | BCAL1138 | hypothetical protein | No homolog |
| pBCA093 | 0.34 | 5E-09 | pBCA093 | PIN domain protein | No homolog |
|  |  |  |  | ***Function unknown (S)*** |  |
| BCAL0728 | 0.50 | 4E-03 | BCAL0728 | hypothetical protein | Bcen2424_2867 |
| BCAL1108 | 0.22 | 3E-11 | BCAL1108 | putative DNA-binding protein | Bcen2424_1226 |
| BCAL1109 | 0.28 | 2E-09 | BCAL1109 | phage derived protein, Gp49-like | Bcen2424_1227 |
| BCAL1401 | 0.25 | 2E-05 | BCAL1401 | hypothetical protein | No homolog |
| BCAL1464 | 0.23 | 5E-05 | BCAL1464 | hypothetical protein | No homolog |
| BCAL1664 | 0.10 | 5E-09 | BCAL1664 | hypothetical protein | Bcen2424_1614 |
| BCAL1665 | 0.07 | 3E-07 | BCAL1665 | SpoVR like protein | No homolog |
| BCAL1683 | 0.42 | 2E-04 | BCAL1683 | hypothetical protein | No homolog |
| BCAL1791 | 0.06 | 8E-08 | BCAL1791 | hypothetical protein | Bcen2424_1719 |
| BCAL1792 | 0.13 | 3E-08 | BCAL1792 | hypothetical protein | Bcen2424_1720 |
| BCAL1794 | 0.26 | 1E-05 | BCAL1794 | hypothetical protein | No homolog |
| BCAL1808 | 0.21 | 5E-11 | BCAL1808 | hypothetical protein | Bcen2424_1736 |
| BCAL1819 | 0.16 | 2E-09 | BCAL1819 | hypothetical protein | Bcen2424_1746 |
| BCAL1913 | 0.15 | 1E-02 | BCAL1913 | putative acetoin catabolism protein | No homolog |
| BCAL2039 | 0.21 | 6E-12 | BCAL2039 | putative uricase | Bcen2424_1962 |
| BCAL2191 | 0.37 | 1E-03 | BCAL2191 | hypothetical protein | No homolog |
| BCAL2196 | 0.44 | 7E-03 | BCAL2196 | HesB family protein | Bcen2424_2124 |
| BCAL2315 | 0.41 | 7E-13 | BCAL2315 | hypothetical protein | No homolog |
| BCAL2731 | 0.23 | 4E-11 | BCAL2731 | ATP-dependent Clp protease adaptor protein ClpS | Bcen2424_2519 |
| BCAL2752 | 0.27 | 7E-05 | BCAL2752 | hypothetical protein | No homolog |
| BCAL2826a | 0.24 | 4E-11 | BCAL2826a | hypothetical protein | Bcen2424_2613 |
| BCAL2827 | 0.39 | 3E-12 | BCAL2827 | hypothetical protein | No homolog |
| BCAL3062 | 0.50 | 3E-08 | BCAL3062 | hypothetical protein | No homolog |
| BCAL3153 | 0.19 | 7E-05 | BCAL3153 | putative lipoprotein | Bcen2424_0853 |
| BCAL3214 | 0.17 | 8E-10 | BCAL3214 | carboxymuconolactone decarboxylase family protein | Bcen2424_0788 |
| BCAL3319 | 0.25 | 3E-04 | BCAL3319 | hypothetical protein | No homolog |
| BCAL3346 | 0.37 | 3E-03 | BCAL3346 | putative HesB-like protein | Bcen2424_0681 |
| BCAL3488 | 0.33 | 3E-10 | BCAL3488 | hypothetical protein | Bcen2424_0022 |
| BCAL3489 | 0.32 | 4E-05 | BCAL3489 | hypothetical protein | Bcen2424_0023 |
| BCAM0062 | 0.37 | 1E-04 | BCAM0062 | 4-carboxymuconolactone decarboxylase | Bcen2424_5750 |
| BCAM0066 | 0.37 | 1E-11 | BCAM0066 | putative lipoprotein | No homolog |
| BCAM0073 | 0.40 | 3E-04 | BCAM0073 | hypothetical protein | No homolog |
| BCAM0271 | 0.48 | 9E-08 | BCAM0271 | hypothetical protein | Bcen2424_3304 |
| BCAM0504 | 0.42 | 3E-04 | BCAM0504 | CsbD-like protein | Bcen2424_3468 |
| BCAM0576 | 0.05 | 2E-07 | BCAM0576 | hypothetical protein* | Bcen2424_3550 |
| BCAM0627 | 0.48 | 9E-06 | BCAM0627 | hypothetical protein | Bcen2424_3599 |
| BCAM0726 | 0.44 | 2E-09 | BCAM0726 | hypothetical protein | Bcen2424_3692 |
| BCAM0770 | 0.01 | 6E-07 | BCAM0770 | hypothetical protein | Bcen2424_3728 |
| BCAM0796 | 0.36 | 9E-12 | BCAM0796 | conserned hypothetical protein | Bcen2424_3759 |
| BCAM0797 | 0.33 | 1E-12 | BCAM0797 | hypothetical protein | No homolog |
| BCAM0843 | 0.21 | 3E-09 | BCAM0843 | putative lipoprotein* | Bcen2424_3808 |
| BCAM0844 | 0.18 | 9E-11 | BCAM0844 | hypothetical protein | No homolog |
| BCAM0888 | 0.39 | 4E-04 | BCAM0888 | hypothetical protein | Bcen2424_3856 |
| BCAM0959 | 0.35 | 4E-04 | BCAM0959 | hypothetical protein | No homolog |
| BCAM0960 | 0.23 | 2E-11 | BCAM0960 | hypothetical protein | No homolog |
| BCAM1014 | 0.40 | 4E-05 | BCAM1014 | putative 3-demethylubiquinone-9 3-methyltransferase | Bcen2424_3980 |
| BCAM1016 | 0.39 | 8E-05 | BCAM1016 | putative ribonuclease* | Bcen2424_3982 |
| BCAM1119 | 0.29 | 2E-03 | BCAM1119 | hypothetical protein | No homolog |
| BCAM1233 | 0.01 | 8E-06 | BCAM1233 | hypothetical protein | Bcen2424_4108 |
| BCAM1234 | 0.01 | 7E-04 | BCAM1234 | hypothetical protein | Bcen2424_4109 |
| BCAM1236 | 0.02 | 2E-05 | BCAM1236 | transglutaminase-like protein | Bcen2424_4111 |
| BCAM1237 | 0.27 | 3E-12 | BCAM1237 | hypothetical protein | Bcen2424_4112 |
| BCAM1261 | 0.24 | 8E-12 | BCAM1261 | hypothetical protein | Bcen2424_4134 |
| BCAM1443 | 0.03 | 8E-07 | BCAM1443 | hypothetical protein | Bcen2424_4315 |
| BCAM1479 | 0.35 | 2E-12 | BCAM1479 | hypothetical protein | Bcen2424_4351 |
| BCAM1496 | 0.32 | 3E-11 | BCAM1496 | hypothetical protein | Bcen2424_4368 |
| BCAM1508 | 0.29 | 1E-10 | BCAM1508 | hypothetical protein* | No homolog |
| BCAM1538 | 0.39 | 7E-06 | BCAM1538 | putative dehydrogenase, monooxygenase subunit | Bcen2424_4405 |
| BCAM1547 | 0.27 | 3E-11 | BCAM1547 | glyoxalase/bleomycin resistance protein/dioxygenase superfamily protein | Bcen2424_4414 |
| BCAM1602 | 0.41 | 6E-03 | BCAM1602 | hypothetical protein | Bcen2424_4458 |
| BCAM1818 | 0.47 | 2E-03 | BCAM1818 | hypothetical protein | Bcen2424_4678 |
| BCAM1850 | 0.13 | 7E-09 | BCAM1850 | hypothetical protein | Bcen2424_4707 |
| BCAM1962A | 0.47 | 1E-03 | BCAM1962A | hypothetical protein | No homolog |
| BCAM2069 | 0.39 | 4E-06 | BCAM2069 | hypothetical protein | Bcen2424_4868 |
| BCAM2154 | 0.09 | 1E-08 | BCAM2154 | hypothetical protein | Bcen2424_4917 |
| BCAM2169 | 0.44 | 5E-04 | BCAM2169 | putative outer membrane autotransporter | No homolog |
| BCAM2213 | 0.31 | 2E-12 | BCAM2213 | hypothetical protein | Bcen2424_4981 |
| BCAM2294 | 0.24 | 2E-09 | BCAM2294 | hypothetical protein | Bcen2424_5051 |
| BCAM2546 | 0.32 | 4E-04 | BCAM2546 | putative MlrC family protein | No homolog |
| BCAM2595 | 0.47 | 3E-09 | BCAM2595 | putative carboxymuconolactone dehydrogenase family protein | Bcen2424_5402 |
| BCAM2700 | 0.42 | 1E-03 | BCAM2700 | hypothetical protein | Bcen2424_5514 |
| BCAS0125 | 0.38 | 6E-06 | BCAS0125 | hypothetical protein | No homolog |
| BCAS0146 | 0.45 | 2E-04 | BCAS0146 | putative MlrC family protein | Bcen2424_6086 |
| BCAS0270 | 0.01 | 7E-06 | BCAS0270 | hypothetical protein | Bcen2424_5925 |
| BCAS0271 | 0.02 | 6E-06 | BCAS0271 | hypothetical protein | Bcen2424_5924 |
| BCAS0757 | 0.42 | 2E-05 | BCAS0757 | hypothetical protein | Bcen2424_6213 |
| pBCA008 | 0.29 | 8E-06 | pBCA008 | hypothetical protein | Bcen2424_6868 |
| pBCA094 | 0.41 | 1E-04 | pBCA094 | hypothetical protein | No homolog |
|  |  |  |  | ***Intergenic regions*** |  |
| IG1_1211174 | 0.34 | 1E-04 | Intergenic region, chromosome 1 | No gene annotation | No homolog |
| IG1_1326468 | 0.13 | 9E-10 | Intergenic region, chromosome 1 | No gene annotation | No homolog |
| IG1_1337938 | 0.49 | 2E-05 | Intergenic region, chromosome 1 | No gene annotation | No homolog |
| IG1_1366016 | 0.48 | 1E-03 | Intergenic region, chromosome 1 | No gene annotation | No homolog |
| IG1_1439332 | 0.13 | 1E-06 | Intergenic region, chromosome 1 | No gene annotation | No homolog |
| IG1_1478353 | 0.07 | 2E-08 | Intergenic region, chromosome 1 | No gene annotation | No homolog |
| IG1_1527001 | 0.16 | 2E-09 | Intergenic region, chromosome 1 | No gene annotation | No homolog |
| IG1_1557854 | 0.46 | 5E-07 | Intergenic region, chromosome 1 | No gene annotation | No homolog |
| IG1_1580939 | 0.37 | 2E-09 | Intergenic region, chromosome 1 | No gene annotation | No homolog |
| IG1_1605376 | 0.21 | 2E-04 | Intergenic region, chromosome 1 | No gene annotation | No homolog |
| IG1_1650963 | 0.30 | 6E-05 | Intergenic region, chromosome 1 | No gene annotation | No homolog |
| IG1_1684126 | 0.19 | 7E-07 | Intergenic region, chromosome 1 | No gene annotation | No homolog |
| IG1_1690680 | 0.35 | 2E-12 | Intergenic region, chromosome 1 | No gene annotation | No homolog |
| IG1_1699963 | 0.38 | 1E-12 | Intergenic region, chromosome 1 | No gene annotation | No homolog |
| IG1_1703192 | 0.35 | 2E-02 | Intergenic region, chromosome 1 | No gene annotation | No homolog |
| IG1_1766538 | 0.18 | 2E-10 | Intergenic region, chromosome 1 | No gene annotation | No homolog |
| IG1_1807854 | 0.32 | 4E-04 | Intergenic region, chromosome 1 | No gene annotation | No homolog |
| IG1_1818831 | 0.08 | 2E-08 | Intergenic region, chromosome 1 | No gene annotation | No homolog |
| IG1_1994827 | 0.34 | 1E-06 | Intergenic region, chromosome 1 | No gene annotation | No homolog |
| IG1_2085013 | 0.46 | 1E-06 | Intergenic region, chromosome 1 | No gene annotation | No homolog |
| IG1_2152204 | 0.26 | 2E-04 | Intergenic region, chromosome 1 | No gene annotation | No homolog |
| IG1_2153880 | 0.34 | 2E-08 | Intergenic region, chromosome 1 | No gene annotation | No homolog |
| IG1_2166257 | 0.34 | 2E-03 | Intergenic region, chromosome 1 | No gene annotation | No homolog |
| IG1_237943 | 0.48 | 1E-07 | Intergenic region, chromosome 1 | No gene annotation | No homolog |
| IG1_2391355 | 0.47 | 3E-09 | Intergenic region, chromosome 1 | No gene annotation | No homolog |
| IG1_2431502 | 0.22 | 8E-05 | Intergenic region, chromosome 1 | No gene annotation | No homolog |
| IG1_2567189 | 0.46 | 2E-06 | Intergenic region, chromosome 1 | No gene annotation | No homolog |
| IG1_2570370 | 0.45 | 2E-05 | Intergenic region, chromosome 1 | No gene annotation | No homolog |
| IG1_2607722 | 0.34 | 3E-10 | Intergenic region, chromosome 1 | No gene annotation | No homolog |
| IG1_2616144 | 0.37 | 7E-13 | Intergenic region, chromosome 1 | No gene annotation | No homolog |
| IG1_2681109 | 0.44 | 3E-05 | Intergenic region, chromosome 1 | No gene annotation | No homolog |
| IG1_2684447 | 0.34 | 5E-05 | Intergenic region, chromosome 1 | No gene annotation | No homolog |
| IG1_2701408 | 0.49 | 7E-03 | Intergenic region, chromosome 1 | No gene annotation | No homolog |
| IG1_2905285 | 0.49 | 4E-03 | Intergenic region, chromosome 1 | No gene annotation | No homolog |
| IG1_2941513 | 0.34 | 5E-10 | Intergenic region, chromosome 1 | No gene annotation | No homolog |
| IG1_2971768 | 0.45 | 4E-07 | Intergenic region, chromosome 1 | No gene annotation | No homolog |
| IG1_3000663 | 0.28 | 6E-10 | Intergenic region, chromosome 1 | No gene annotation | No homolog |
| IG1_3051717 | 0.38 | 6E-05 | Intergenic region, chromosome 1 | No gene annotation | No homolog |
| IG1_3089813 | 0.01 | 3E-04 | Intergenic region, chromosome 1 | No gene annotation | No homolog |
| IG1_3095414 | 0.38 | 4E-08 | Intergenic region, chromosome 1 | No gene annotation | No homolog |
| IG1_315662 | 0.21 | 2E-12 | Intergenic region, chromosome 1 | No gene annotation | No homolog |
| IG1_3169576 | 0.48 | 3E-12 | Intergenic region, chromosome 1 | No gene annotation | No homolog |
| IG1_3171459 | 0.37 | 6E-04 | Intergenic region, chromosome 1 | No gene annotation | No homolog |
| IG1_322475 | 0.27 | 8E-12 | Intergenic region, chromosome 1 | No gene annotation | No homolog |
| IG1_3242996 | 0.11 | 3E-08 | Intergenic region, chromosome 1 | No gene annotation | No homolog |
| IG1_3245322 | 0.49 | 1E-02 | Intergenic region, chromosome 1 | No gene annotation | No homolog |
| IG1_3246787 | 0.34 | 5E-03 | Intergenic region, chromosome 1 | No gene annotation | No homolog |
| IG1_3295009 | 0.17 | 4E-07 | Intergenic region, chromosome 1 | No gene annotation | No homolog |
| IG1_3297972 | 0.10 | 3E-08 | Intergenic region, chromosome 1 | No gene annotation | No homolog |
| IG1_3390104 | 0.01 | 4E-07 | Intergenic region, chromosome 1 | No gene annotation | No homolog |
| IG1_3393882 | 0.01 | 2E-06 | Intergenic region, chromosome 1 | No gene annotation | No homolog |
| IG1_3453557 | 0.10 | 2E-08 | Intergenic region, chromosome 1 | No gene annotation | No homolog |
| IG1_3490179 | 0.39 | 2E-07 | Intergenic region, chromosome 1 | No gene annotation | No homolog |
| IG1_3503400 | 0.33 | 2E-05 | Intergenic region, chromosome 1 | No gene annotation | No homolog |
| IG1_3596870 | 0.41 | 5E-05 | Intergenic region, chromosome 1 | No gene annotation | No homolog |
| IG1_3598341 | 0.49 | 3E-02 | Intergenic region, chromosome 1 | No gene annotation | No homolog |
| IG1_3667388 | 0.27 | 3E-07 | Intergenic region, chromosome 1 | No gene annotation | No homolog |
| IG1_418869 | 0.42 | 6E-03 | Intergenic region, chromosome 1 | No gene annotation | No homolog |
| IG1_486377 | 0.43 | 1E-05 | Intergenic region, chromosome 1 | No gene annotation | No homolog |
| IG1_572528 | 0.42 | 3E-08 | Intergenic region, chromosome 1 | No gene annotation | No homolog |
| IG1_593118 | 0.03 | 2E-07 | Intergenic region, chromosome 1 | No gene annotation | No homolog |
| IG1_594404 | 0.31 | 3E-10 | Intergenic region, chromosome 1 | No gene annotation | No homolog |
| IG1_598325 | 0.38 | 5E-11 | Intergenic region, chromosome 1 | No gene annotation | No homolog |
| IG1_657823 | 0.19 | 5E-11 | Intergenic region, chromosome 1 | No gene annotation | No homolog |
| IG1_659402 | 0.28 | 2E-06 | Intergenic region, chromosome 1 | No gene annotation | No homolog |
| IG1_677590 | 0.09 | 2E-08 | Intergenic region, chromosome 1 | No gene annotation | No homolog |
| IG1_697051 | 0.36 | 6E-03 | Intergenic region, chromosome 1 | No gene annotation | No homolog |
| IG1_711528 | 0.01 | 4E-07 | Intergenic region, chromosome 1 | No gene annotation | No homolog |
| IG1_72814 | 0.09 | 4E-08 | Intergenic region, chromosome 1 | No gene annotation | No homolog |
| IG1_754777 | 0.45 | 2E-13 | Intergenic region, chromosome 1 | No gene annotation | No homolog |
| IG1_77800 | 0.04 | 4E-07 | Intergenic region, chromosome 1 | No gene annotation | No homolog |
| IG1_986723 | 0.50 | 2E-11 | Intergenic region, chromosome 1 | No gene annotation | No homolog |
| IG2_1055462 | 0.21 | 2E-10 | Intergenic region, chromosome 2 | No gene annotation | No homolog |
| IG2_1060502 | 0.40 | 1E-09 | Intergenic region, chromosome 2 | No gene annotation | No homolog |
| IG2_1092309 | 0.14 | 1E-08 | Intergenic region, chromosome 2 | No gene annotation | No homolog |
| IG2_1120955 | 0.02 | 3E-07 | Intergenic region, chromosome 2 | No gene annotation | No homolog |
| IG2_1123992 | 0.15 | 4E-08 | Intergenic region, chromosome 2 | No gene annotation | No homolog |
| IG2_1136610 | 0.36 | 4E-04 | Intergenic region, chromosome 2 | No gene annotation | No homolog |
| IG2_1231039 | 0.24 | 8E-04 | Intergenic region, chromosome 2 | No gene annotation | No homolog |
| IG2_1336095 | 0.05 | 2E-07 | Intergenic region, chromosome 2 | No gene annotation | No homolog |
| IG2_1351912 | 0.02 | 6E-07 | Intergenic region, chromosome 2 | No gene annotation | No homolog |
| IG2_1353910 | 0.05 | 1E-07 | Intergenic region, chromosome 2 | No gene annotation | No homolog |
| IG2_1357417 | 0.03 | 3E-07 | Intergenic region, chromosome 2 | No gene annotation | No homolog |
| IG2_1361275 | 0.25 | 3E-05 | Intergenic region, chromosome 2 | No gene annotation | No homolog |
| IG2_1377787 | 0.47 | 6E-11 | Intergenic region, chromosome 2 | No gene annotation | No homolog |
| IG2_1395299 | 0.39 | 1E-04 | Intergenic region, chromosome 2 | No gene annotation | No homolog |
| IG2_1486238 | 0.42 | 7E-04 | Intergenic region, chromosome 2 | No gene annotation | No homolog |
| IG2_1491140 | 0.22 | 8E-08 | Intergenic region, chromosome 2 | No gene annotation | No homolog |
| IG2_1502006 | 0.41 | 3E-04 | Intergenic region, chromosome 2 | No gene annotation | No homolog |
| IG2_1503934 | 0.34 | 8E-13 | Intergenic region, chromosome 2 | No gene annotation | No homolog |
| IG2_1518908 | 0.38 | 5E-06 | Intergenic region, chromosome 2 | No gene annotation | No homolog |
| IG2_1565086 | 0.29 | 9E-04 | Intergenic region, chromosome 2 | No gene annotation | No homolog |
| IG2_1660910 | 0.24 | 1E-11 | Intergenic region, chromosome 2 | No gene annotation | No homolog |
| IG2_1706950 | 0.43 | 1E-03 | Intergenic region, chromosome 2 | No gene annotation | No homolog |
| IG2_1713089 | 0.39 | 2E-07 | Intergenic region, chromosome 2 | No gene annotation | No homolog |
| IG2_1719237 | 0.31 | 1E-09 | Intergenic region, chromosome 2 | No gene annotation | No homolog |
| IG2_1729992 | 0.45 | 2E-03 | Intergenic region, chromosome 2 | No gene annotation | No homolog |
| IG2_1745287 | 0.07 | 6E-07 | Intergenic region, chromosome 2 | No gene annotation | No homolog |
| IG2_1882664 | 0.01 | 5E-07 | Intergenic region, chromosome 2 | No gene annotation | No homolog |
| IG2_1884439 | 0.05 | 1E-07 | Intergenic region, chromosome 2 | No gene annotation | No homolog |
| IG2_1885142 | 0.15 | 2E-09 | Intergenic region, chromosome 2 | No gene annotation | No homolog |
| IG2_1985968 | 0.04 | 3E-07 | Intergenic region, chromosome 2 | No gene annotation | No homolog |
| IG2_2018202 | 0.25 | 6E-12 | Intergenic region, chromosome 2 | No gene annotation | No homolog |
| IG2_2259633 | 0.39 | 2E-03 | Intergenic region, chromosome 2 | No gene annotation | No homolog |
| IG2_2274945 | 0.13 | 4E-09 | Intergenic region, chromosome 2 | No gene annotation | No homolog |
| IG2_2301878 | 0.19 | 3E-06 | Intergenic region, chromosome 2 | No gene annotation | No homolog |
| IG2_2304176 | 0.29 | 2E-05 | Intergenic region, chromosome 2 | No gene annotation | No homolog |
| IG2_2378068 | 0.50 | 8E-08 | Intergenic region, chromosome 2 | No gene annotation | No homolog |
| IG2_2466870 | 0.22 | 3E-11 | Intergenic region, chromosome 2 | No gene annotation | No homolog |
| IG2_2473824 | 0.42 | 2E-04 | Intergenic region, chromosome 2 | No gene annotation | No homolog |
| IG2_2586922 | 0.27 | 9E-03 | Intergenic region, chromosome 2 | No gene annotation | No homolog |
| IG2_2614723 | 0.03 | 3E-07 | Intergenic region, chromosome 2 | No gene annotation | No homolog |
| IG2_2617890 | 0.06 | 2E-07 | Intergenic region, chromosome 2 | No gene annotation | No homolog |
| IG2_2698655 | 0.33 | 6E-06 | Intergenic region, chromosome 2 | No gene annotation | No homolog |
| IG2_2789309 | 0.37 | 1E-06 | Intergenic region, chromosome 2 | No gene annotation | No homolog |
| IG2_2841811 | 0.05 | 2E-07 | Intergenic region, chromosome 2 | No gene annotation | No homolog |
| IG2_3004307 | 0.42 | 4E-13 | Intergenic region, chromosome 2 | No gene annotation | No homolog |
| IG2_3023656 | 0.34 | 1E-03 | Intergenic region, chromosome 2 | No gene annotation | No homolog |
| IG2_3061820 | 0.38 | 6E-12 | Intergenic region, chromosome 2 | No gene annotation | No homolog |
| IG2_321816 | 0.43 | 1E-03 | Intergenic region, chromosome 2 | No gene annotation | No homolog |
| IG2_412993 | 0.34 | 2E-04 | Intergenic region, chromosome 2 | No gene annotation | No homolog |
| IG2_418529 | 0.42 | 7E-06 | Intergenic region, chromosome 2 | No gene annotation | No homolog |
| IG2_551370 | 0.09 | 5E-09 | Intergenic region, chromosome 2 | No gene annotation | No homolog |
| IG2_58076 | 0.09 | 2E-08 | Intergenic region, chromosome 2 | No gene annotation | No homolog |
| IG2_585146 | 0.30 | 2E-02 | Intergenic region, chromosome 2 | No gene annotation | No homolog |
| IG2_639578 | 0.21 | 4E-10 | Intergenic region, chromosome 2 | No gene annotation | No homolog |
| IG2_643345 | 0.17 | 6E-10 | Intergenic region, chromosome 2 | No gene annotation | No homolog |
| IG2_64643 | 0.41 | 8E-05 | Intergenic region, chromosome 2 | No gene annotation | No homolog |
| IG2_690307 | 0.44 | 2E-11 | Intergenic region, chromosome 2 | No gene annotation | No homolog |
| IG2_737876 | 0.43 | 5E-05 | Intergenic region, chromosome 2 | No gene annotation | Bcen2424_3636 |
| IG2_878857 | 0.27 | 2E-04 | Intergenic region, chromosome 2 | No gene annotation | No homolog |
| IG2_943874 | 0.01 | 6E-07 | Intergenic region, chromosome 2 | No gene annotation | No homolog |
| IG2_949283 | 0.01 | 7E-06 | Intergenic region, chromosome 2 | No gene annotation | No homolog |
| IG2_961669 | 0.28 | 7E-12 | Intergenic region, chromosome 2 | No gene annotation | Bcen2424_3838 |
| IG2_963604 | 0.30 | 3E-10 | Intergenic region, chromosome 2 | No gene annotation | No homolog |
| IG2_977477 | 0.40 | 5E-12 | Intergenic region, chromosome 2 | No gene annotation | No homolog |
| IG2_984755 | 0.23 | 1E-02 | Intergenic region, chromosome 2 | No gene annotation | No homolog |
| IG2_996197 | 0.29 | 6E-04 | Intergenic region, chromosome 2 | No gene annotation | No homolog |
| IG3_121896 | 0.03 | 5E-07 | Intergenic region, chromosome 3 | No gene annotation | Bcen2424_6115 |
| IG3_127820 | 0.22 | 3E-11 | Intergenic region, chromosome 3 | No gene annotation | No homolog |
| IG3_154338 | 0.12 | 6E-10 | Intergenic region, chromosome 3 | No gene annotation | No homolog |
| IG3_156712 | 0.22 | 2E-10 | Intergenic region, chromosome 3 | No gene annotation | Bcen2424_6091 |
| IG3_209555 | 0.29 | 8E-06 | Intergenic region, chromosome 3 | No gene annotation | No homolog |
| IG3_26003 | 0.21 | 8E-06 | Intergenic region, chromosome 3 | No gene annotation | No homolog |
| IG3_301419 | 0.04 | 6E-04 | Intergenic region, chromosome 3 | No gene annotation | No homolog |
| IG3_306996 | 0.02 | 1E-04 | Intergenic region, chromosome 3 | No gene annotation | No homolog |
| IG3_347461 | 0.32 | 8E-11 | Intergenic region, chromosome 3 | No gene annotation | No homolog |
| IG3_354019 | 0.14 | 1E-09 | Intergenic region, chromosome 3 | No gene annotation | No homolog |
| IG3_452850 | 0.48 | 2E-03 | Intergenic region, chromosome 3 | No gene annotation | No homolog |
| IG3_453171 | 0.39 | 4E-09 | Intergenic region, chromosome 3 | No gene annotation | No homolog |
| IG3_480246 | 0.39 | 5E-03 | Intergenic region, chromosome 3 | No gene annotation | No homolog |
| IG3_613426 | 0.13 | 3E-06 | Intergenic region, chromosome 3 | No gene annotation | No homolog |
| IG3_710629 | 0.48 | 3E-04 | Intergenic region, chromosome 3 | No gene annotation | No homolog |
|  |  |  |  |  |  |
| AU1054_G0993 | 0.44 | 7E-03 | No homolog | No gene annotation | No homolog |
| AU1054_G2526 | 0.49 | 1E-07 | No homolog | No gene annotation | No homolog |
| AU1054_G2816 | 0.38 | 3E-04 | No homolog | No gene annotation | No homolog |
| AU1054_G3696 | 0.07 | 2E-06 | No homolog | No gene annotation | No homolog |
| AU1054_G6153 | 0.38 | 2E-11 | No homolog | No gene annotation | No homolog |
| BCAL0057 | 0.12 | 8E-09 | No homolog | No gene annotation | No homolog |
| BCAL0063 | 0.09 | 3E-08 | BCAL0063 | putative FMN flavoprotein | Bcen2424_0133 |
| BCAL0065 | 0.12 | 3E-06 | BCAL0065 | hypothetical protein | Bcen2424_0135 |
| BCAL0066 | 0.01 | 1E-06 | No homolog | No gene annotation | No homolog |
| BCAL0193 | 0.45 | 1E-02 | BCAL0193 | hypothetical protein* | Bcen2424_0305 |
| BCAL0200 | 0.48 | 2E-12 | BCAL0200 | putative lipoprotein | No homolog |
| BCAL0431 | 0.22 | 3E-11 | BCAL0431 | hypothetical protein | Bcen2424_3159 |
| BCAL0432 | 0.22 | 1E-05 | BCAL0432 | hypothetical protein | Bcen2424_3158 |
| BCAL0434 | 0.28 | 2E-10 | BCAL0434 | hypothetical protein | Bcen2424_3155 |
| BCAL0440 | 0.41 | 5E-07 | BCAL0440 | hypothetical protein | Bcen2424_3148 |
| BCAL0514 | 0.05 | 3E-07 | BCAL0514 | hypothetical protein | Bcen2424_3076 |
| BCAL0585 | 0.14 | 6E-09 | BCAL0585 | hypothetical protein | Bcen2424_3007 |
| BCAL0594 | 0.15 | 8E-07 | BCAL0594 | hypothetical protein | Bcen2424_3000 |
| BCAL0617 | 0.10 | 2E-08 | BCAL0617 | hypothetical protein | Bcen2424_2975 |
| BCAL0623 | 0.19 | 3E-10 | BCAL0623 | hypothetical protein* | No homolog |
| BCAL0653 | 0.07 | 1E-07 | BCAL0653 | hypothetical protein | Bcen2424_2937 |
| BCAL0654 | 0.47 | 6E-11 | BCAL0654 | hypothetical protein | No homolog |
| BCAL0731 | 0.42 | 5E-10 | BCAL0731 | glutamate--cysteine ligase | Bcen2424_2864 |
| BCAL0831 | 0.08 | 2E-07 | BCAL0831 | putative storage protein | No homolog |
| BCAL0834 | 0.15 | 6E-10 | BCAL0834 | hypothetical protein | Bcen2424_2770 |
| BCAL0858 | 0.41 | 1E-03 | BCAL0858 | hypothetical protein | No homolog |
| BCAL0860 | 0.35 | 2E-12 | BCAL0860 | putative protease | Bcen2424_2746 |
| BCAL0889 | 0.49 | 6E-06 | BCAL0889 | hypothetical protein | Bcen2424_2716 |
| BCAL0927 | 0.16 | 4E-09 | BCAL0927 | hypothetical protein | Bcen2424_2676 |
| BCAL0928 | 0.02 | 4E-07 | BCAL0928 | hypothetical protein | Bcen2424_2675 |
| BCAL0964 | 0.44 | 2E-07 | BCAL0964 | hypothetical protein | No homolog |
| BCAL1023 | 0.49 | 2E-03 | Multiple hits | No gene annotation | Bcen2424_1147 |
| BCAL1085 | 0.38 | 1E-04 | BCAL1085 | hypothetical protein | No homolog |
| BCAL1102 | 0.37 | 5E-12 | BCAL1102 | putative lipoprotein | Bcen2424_1221 |
| BCAL1105 | 0.22 | 3E-04 | BCAL1105 | hypothetical protein | No homolog |
| BCAL1130 | 0.35 | 2E-06 | BCAL1130 | hypothetical protein | No homolog |
| BCAL1131 | 0.33 | 1E-05 | BCAL1131 | hypothetical protein | No homolog |
| BCAL1132 | 0.38 | 3E-03 | No homolog | No gene annotation | No homolog |
| BCAL1238 | 0.40 | 5E-04 | BCAL1238 | putative malonate transport-related system membrane protein | Bcen2424_1267 |
| BCAL1282 | 0.03 | 4E-07 | BCAL1282 | hypothetical protein | Bcen2424_1312 |
| BCAL1291 | 0.47 | 3E-02 | BCAL1291 | hypothetical protein | No homolog |
| BCAL1292 | 0.48 | 3E-03 | BCAL1292 | hypothetical protein | No homolog |
| BCAL1320 | 0.05 | 1E-04 | BCAL1320 | hypothetical protein | No homolog |
| BCAL1413A | 0.20 | 3E-10 | BCAL1413A | putative lipoprotein | Bcen2424_1406 |
| BCAL1418 | 0.28 | 1E-03 | BCAL1418 | Major Facilitator Superfamily protein | Bcen2424_1411 |
| BCAL1458 | 0.24 | 1E-09 | BCAL1458 | hypothetical protein | Bcen2424_1451 |
| BCAL1492 | 0.35 | 3E-08 | BCAL1492 | hypothetical protein | Bcen2424_1485 |
| BCAL1498 | 0.04 | 1E-07 | BCAL1498 | hypothetical protein | Bcen2424_1491 |
| BCAL1499 | 0.02 | 5E-06 | BCAL1499 | hypothetical protein | Bcen2424_1492 |
| BCAL1523 | 0.48 | 2E-09 | BCAL1523 | putative lipoprotein | Bcen2424_1515 |
| BCAL1524 | 0.17 | 3E-10 | BCAL1524 | putative lipoprotein | No homolog |
| BCAL1534 | 0.31 | 1E-11 | BCAL1534 | hypothetical protein | Bcen2424_1527 |
| BCAL1539 | 0.35 | 3E-06 | BCAL1539 | hypothetical protein* | Bcen2424_1532 |
| BCAL1623 | 0.28 | 1E-12 | No homolog | No gene annotation | No homolog |
| BCAL1624 | 0.22 | 1E-08 | BCAL1624 | hypothetical protein | Bcen2424_1567 |
| BCAL1763 | 0.40 | 2E-03 | BCAL1763 | hypothetical protein | Bcen2424_1700 |
| BCAL1795 | 0.25 | 6E-07 | BCAL1795 | putative ATP-ase | Bcen2424_1722 |
| BCAL1832 | 0.37 | 2E-10 | BCAL1832 | hypothetical protein | Bcen2424_1759 |
| BCAL1844 | 0.45 | 8E-03 | BCAL1844 | hypothetical protein | Bcen2424_1771 |
| BCAL1868 | 0.41 | 1E-04 | BCAL1868 | hypothetical protein | Bcen2424_1796 |
| BCAL1954 | 0.32 | 4E-05 | BCAL1954 | hypothetical protein | No homolog |
| BCAL2025 | 0.07 | 6E-08 | BCAL2025 | hypothetical protein | Bcen2424_1952 |
| BCAL2027 | 0.02 | 2E-06 | No homolog | No gene annotation | No homolog |
| BCAL2066 | 0.41 | 2E-06 | BCAL2066 | No gene annotation | No homolog |
| BCAL2125 | 0.22 | 3E-09 | BCAL2125 | putative acetyltransferase | Bcen2424_2055 |
| BCAL2206 | 0.14 | 2E-05 | BCAL2206 | phasin-like protein | Bcen2424_2134 |
| BCAL2265 | 0.45 | 6E-06 | BCAL2265 | hypothetical protein | Bcen2424_2184 |
| BCAL2296 | 0.34 | 3E-11 | BCAL2296 | hypothetical protein | No homolog |
| BCAL2361 | 0.29 | 2E-12 | BCAL2361 | hypothetical protein | Bcen2424_2267 |
| BCAL2375 | 0.38 | 4E-05 | BCAL2375 | hypothetical protein | No homolog |
| BCAL2467 | 0.39 | 2E-04 | BCAL2467 | putative lipoprotein | Bcen2424_2388 |
| BCAL2468 | 0.43 | 4E-04 | BCAL2468 | hypothetical protein | Bcen2424_2389 |
| BCAL2698 | 0.50 | 1E-12 | BCAL2698 | hypothetical protein | No homolog |
| BCAL2709 | 0.06 | 5E-08 | BCAL2709 | hypothetical protein | No homolog |
| BCAL2859 | 0.26 | 9E-12 | Multiple hits | No gene annotation | Bcen2424_1140 |
| BCAL2962 | 0.23 | 8E-11 | BCAL2962 | hypothetical protein | No homolog |
| BCAL2962a | 0.45 | 5E-05 | BCAL2962a | hypothetical protein | No homolog |
| BCAL2964 | 0.25 | 6E-12 | BCAL2964 | putative phage-related DNA-binding protein | No homolog |
| BCAL2970 | 0.45 | 2E-02 | BCAL2970 | hypothetical protein | No homolog |
| BCAL2971 | 0.50 | 5E-05 | BCAL2971 | hypothetical protein | No homolog |
| BCAL2979 | 0.30 | 4E-03 | BCAL2979 | NAD-dependent formate dehydrogenase delta subunit | Bcen2424_1030 |
| BCAL2998 | 0.19 | 1E-06 | BCAL2998 | transglycosylase associated protein | No homolog |
| BCAL3003 | 0.08 | 1E-08 | BCAL3003 | hypothetical protein | Bcen2424_1008 |
| BCAL3007 | 0.09 | 4E-07 | BCAL3007 | hypothetical protein* | Bcen2424_1004 |
| BCAL3018 | 0.46 | 8E-04 | BCAL3018 | hypothetical protein | Bcen2424_0993 |
| BCAL3059A | 0.44 | 8E-13 | BCAL3059A | hypothetical protein | No homolog |
| BCAL3148 | 0.17 | 3E-11 | BCAL3148 | hypothetical protein | Bcen2424_0858 |
| BCAL3150 | 0.19 | 2E-11 | BCAL3150 | hypothetical protein* | Bcen2424_0856 |
| BCAL3207 | 0.41 | 2E-03 | No homolog | No gene annotation | No homolog |
| BCAL3410 | 0.32 | 6E-08 | BCAL3410 | hypothetical protein | Bcen2424_0616 |
| BCAL3490 | 0.46 | 4E-04 | BCAL3490 | hypothetical protein | Bcen2424_0024 |
| BCALr1899 | 0.41 | 3E-02 | BCALr1899 | misc_RNA | Bcen2424_R0046 |
| BCALr2281 | 0.33 | 2E-02 | BCALr2281 | tRNA-Ser | Bcen2424_R0060 |
| BCALr3029 | 0.45 | 2E-02 | BCALr3029 | tRNA-Ser | Bcen2424_R0025 |
| BCAM0022 | 0.41 | 4E-03 | Multiple hits | No gene annotation | Multiple hits |
| BCAM0025 | 0.18 | 5E-11 | No homolog | No gene annotation | No homolog |
| BCAM0050a | 0.19 | 1E-10 | No homolog | No gene annotation | No homolog |
| BCAM0052 | 0.07 | 8E-08 | BCAM0052 | hypothetical protein | Bcen2424_5740 |
| BCAM0064 | 0.11 | 1E-08 | BCAM0064 | hypothetical protein | Bcen2424_5752 |
| BCAM0069 | 0.22 | 2E-11 | BCAM0069 | hypothetical protein | No homolog |
| BCAM0074 | 0.45 | 2E-04 | BCAM0074 | hypothetical protein | No homolog |
| BCAM0088 | 0.42 | 6E-07 | BCAM0088 | putative peptidyl-prolyl cis-trans isomerase | Bcen2424_5787 |
| BCAM0159 | 0.18 | 3E-10 | BCAM0159 | conserved ypothetical protein | No homolog |
| BCAM0160 | 0.26 | 9E-10 | BCAM0160 | hypothetical protein* | Bcen2424_5854 |
| BCAM0184 | 0.05 | 1E-07 | BCAM0184 | lectin | Bcen2424_3176 |
| BCAM0185 | 0.04 | 1E-07 | BCAM0185 | lectin | Bcen2424_3177 |
| BCAM0186 | 0.04 | 1E-07 | BCAM0186 | lectin | Bcen2424_3178 |
| BCAM0257 | 0.45 | 1E-04 | BCAM0257 | putative transcriptional regulator | No homolog |
| BCAM0258 | 0.34 | 5E-04 | BCAM0258 | repressor protein | No homolog |
| BCAM0301 | 0.37 | 2E-03 | BCAM0301 | hypothetical protein | No homolog |
| BCAM0411 | 0.46 | 2E-03 | No homolog | No gene annotation | No homolog |
| BCAM0444 | 0.19 | 3E-11 | BCAM0444 | hypothetical protein | No homolog |
| BCAM0453 | 0.30 | 3E-06 | BCAM0453 | hypothetical protein | No homolog |
| BCAM0494 | 0.44 | 8E-06 | BCAM0494 | hypothetical protein | No homolog |
| BCAM0495 | 0.32 | 7E-05 | BCAM0495 | putative DNA-binding protein | No homolog |
| BCAM0525A | 0.49 | 7E-04 | BCAM0525A | hypothetical protein | No homolog |
| BCAM0551 | 0.35 | 2E-06 | No homolog | No gene annotation | No homolog |
| BCAM0582 | 0.50 | 2E-04 | No homolog | No gene annotation | No homolog |
| BCAM0624 | 0.48 | 2E-04 | BCAM0624 | hypothetical protein | No homolog |
| BCAM0634 | 0.04 | 6E-05 | BCAM0634 | hypothetical protein | Bcen2424_3605 |
| BCAM0694 | 0.33 | 3E-07 | BCAM0694 | hypothetical protein | Bcen2424_3660 |
| BCAM0695 | 0.29 | 8E-04 | BCAM0695 | putative lipoprotein | No homolog |
| BCAM0701 | 0.44 | 1E-06 | BCAM0701 | putative lipoprotein* | No homolog |
| BCAM0753 | 0.18 | 3E-10 | BCAM0753 | hypothetical protein | Bcen2424_3719 |
| BCAM0772 | 0.09 | 6E-08 | No homolog | No gene annotation | No homolog |
| BCAM0773 | 0.20 | 8E-10 | BCAM0773 | hypothetical protein | No homolog |
| BCAM0776 | 0.05 | 5E-06 | No homolog | No gene annotation | No homolog |
| BCAM0852 | 0.21 | 5E-10 | BCAM0852 | hypothetical protein | Bcen2424_3817 |
| BCAM0853 | 0.02 | 2E-07 | BCAM0853 | hypothetical protein | Bcen2424_3818 |
| BCAM0856_J_0 | 0.02 | 4E-07 | No homolog | No gene annotation | Bcen2424_3820 |
| BCAM0856_J_1 | 0.01 | 1E-06 | BCAM0856 | No gene annotation | Bcen2424_3820 |
| BCAM0862 | 0.04 | 1E-07 | BCAM0862 | putative polymerase | No homolog |
| BCAM0875 | 0.30 | 1E-04 | BCAM0875 | hypothetical protein | Bcen2424_3843 |
| BCAM0877 | 0.13 | 1E-09 | BCAM0877 | putative serine/threonine protein phosphatase | Bcen2424_3845 |
| BCAM0887 | 0.39 | 7E-10 | BCAM0887 | putative tautomerase | Bcen2424_3855 |
| BCAM0895 | 0.32 | 3E-03 | BCAM0895 | hypothetical protein | No homolog |
| BCAM0943 | 0.28 | 2E-11 | BCAM0943 | hypothetical protein | No homolog |
| BCAM0945 | 0.50 | 1E-03 | BCAM0945 | hypothetical protein | Bcen2424_3911 |
| BCAM0976 | 0.31 | 2E-04 | No homolog | No gene annotation | No homolog |
| BCAM0979 | 0.23 | 2E-10 | BCAM0979 | putative glutathione-S-transferase | Bcen2424_3946 |
| BCAM1002 | 0.42 | 6E-05 | Multiple hits | No gene annotation | Multiple hits |
| BCAM1006 | 0.04 | 2E-07 | BCAM1006 | hypothetical protein | Bcen2424_3972 |
| BCAM1052 | 0.17 | 3E-10 | BCAM1052 | hypothetical protein | No homolog |
| BCAM1053 | 0.33 | 2E-04 | Multiple hits | No gene annotation | No homolog |
| BCAM1097 | 0.07 | 2E-08 | BCAM1097 | hypothetical protein | No homolog |
| BCAM1136 | 0.05 | 2E-07 | BCAM1136 | hypothetical protein | Bcen2424_4032 |
| BCAM1164 | 0.09 | 1E-08 | BCAM1164 | hypothetical protein | Bcen2424_4058 |
| BCAM1203 | 0.30 | 9E-13 | No homolog | No gene annotation | No homolog |
| BCAM1215 | 0.45 | 6E-10 | BCAM1215 | hypothetical protein | Bcen2424_4089 |
| BCAM1239 | 0.14 | 2E-09 | No homolog | No gene annotation | No homolog |
| BCAM1260 | 0.26 | 2E-12 | BCAM1260 | putative lipoprotein | No homolog |
| BCAM1305 | 0.27 | 4E-12 | BCAM1305 | putative transcriptional regulator | Bcen2424_4177 |
| BCAM1356 | 0.29 | 2E-10 | BCAM1356 | putative gluconate 2-dehydrogenase subunit 3 | Bcen2424_4223 |
| BCAM1363 | 0.17 | 2E-09 | BCAM1363 | hypothetical protein | Bcen2424_4228 |
| BCAM1403 | 0.36 | 1E-04 | No homolog | No gene annotation | No homolog |
| BCAM1451 | 0.49 | 9E-03 | BCAM1451 | hypothetical protein | Bcen2424_4323 |
| BCAM1480 | 0.48 | 1E-06 | BCAM1480 | hypothetical protein | Bcen2424_4352 |
| BCAM1481 | 0.23 | 1E-11 | BCAM1481 | hypothetical protein | Bcen2424_4353 |
| BCAM1482 | 0.30 | 1E-07 | BCAM1482 | hypothetical protein | No homolog |
| BCAM1491 | 0.09 | 3E-08 | BCAM1491 | hypothetical protein | Bcen2424_4363 |
| BCAM1501 | 0.10 | 4E-07 | BCAM1501 | hypothetical protein* | Bcen2424_4373 |
| BCAM1502 | 0.04 | 5E-07 | BCAM1502 | hypothetical protein | Bcen2424_4374 |
| BCAM1507 | 0.21 | 6E-10 | BCAM1507 | hypothetical protein | Bcen2424_4379 |
| BCAM1509 | 0.39 | 5E-05 | BCAM1509 | putative PRC-barrel domain protein | Bcen2424_4381 |
| BCAM1511 | 0.24 | 8E-12 | BCAM1511 | hypothetical protein | Bcen2424_4384 |
| BCAM1513 | 0.46 | 5E-05 | BCAM1513 | hypothetical protein | Bcen2424_4386 |
| BCAM1526 | 0.11 | 3E-07 | No homolog | No gene annotation | No homolog |
| BCAM1537 | 0.28 | 4E-12 | No homolog | No gene annotation | No homolog |
| BCAM1543a | 0.36 | 3E-12 | BCAM1543C | No gene annotation | No homolog |
| BCAM1548 | 0.42 | 8E-09 | BCAM1548 | hypothetical protein | Bcen2424_4415 |
| BCAM1555 | 0.32 | 2E-12 | BCAM1555 | hypothetical protein | Bcen2424_4424 |
| BCAM1569 | 0.10 | 2E-07 | BCAM1569 | putative BNR/Asp-box protein* | Bcen2424_4437 |
| BCAM1573 | 0.22 | 9E-12 | No homolog | No gene annotation | No homolog |
| BCAM1594 | 0.31 | 2E-03 | BCAM1594 | hypothetical protein | No homolog |
| BCAM1598 | 0.20 | 8E-08 | BCAM1598 | putative lipoprotein | No homolog |
| BCAM1662 | 0.45 | 1E-08 | BCAM1662 | hypothetical protein | Bcen2424_4481 |
| BCAM1668 | 0.26 | 5E-12 | BCAM1668 | hypothetical protein | Bcen2424_4488 |
| BCAM1669 | 0.22 | 2E-07 | BCAM1669 | hypothetical protein | No homolog |
| BCAM1739 | 0.45 | 4E-04 | No homolog | No gene annotation | No homolog |
| BCAM1742 | 0.30 | 2E-04 | No homolog | No gene annotation | No homolog |
| BCAM1794 | 0.28 | 1E-05 | BCAM1794 | No gene annotation* | Bcen2424_4613 |
| BCAM1794 | 0.38 | 4E-09 | BCAM1794 | No gene annotation* | Bcen2424_4613 |
| BCAM1799 | 0.33 | 2E-12 | BCAM1799 | hypothetical protein | No homolog |
| BCAM1830 | 0.34 | 1E-12 | BCAM1830 | hypothetical protein | No homolog |
| BCAM1853 | 0.25 | 3E-11 | BCAM1853 | hypothetical protein | Bcen2424_4710 |
| BCAM1858 | 0.17 | 1E-09 | BCAM1858 | hypothetical protein* | Bcen2424_4715 |
| BCAM1873 | 0.50 | 6E-04 | BCAM1873 | glyoxalase/bleomycin resistance/dioxygenase superfamily protein | Bcen2424_4729 |
| BCAM1875 | 0.38 | 2E-05 | BCAM1875 | hypothetical protein | No homolog |
| BCAM1878 | 0.44 | 6E-03 | BCAM1878 | putative phage repressor protein | No homolog |
| BCAM1927 | 0.35 | 3E-03 | BCAM1927 | hypothetical protein | Bcen2424_4733 |
| BCAM2005A | 0.17 | 1E-09 | BCAM2005A | putative entericidin | Bcen2424_4806 |
| BCAM2068 | 0.28 | 2E-04 | BCAM2068 | hypothetical protein | No homolog |
| BCAM2073 | 0.36 | 4E-05 | BCAM2073 | hypothetical protein* | Bcen2424_4880 |
| BCAM2081 | 0.16 | 6E-08 | BCAM2081 | hypothetical protein | No homolog |
| BCAM2116 | 0.12 | 3E-04 | BCAM2116 | hypothetical protein | No homolog |
| BCAM2130 | 0.15 | 7E-10 | BCAM2130 | 3-hydroxyanthranilate 3,4-dioxygenase | No homolog |
| BCAM2152 | 0.23 | 8E-06 | BCAM2152 | hypothetical protein | Bcen2424_4915 |
| BCAM2206 | 0.24 | 2E-11 | BCAM2206 | hypothetical protein | Bcen2424_4972 |
| BCAM2207 | 0.03 | 1E-06 | BCAM2207 | hypothetical protein | Bcen2424_4973 |
| BCAM2209 | 0.04 | 2E-07 | BCAM2209 | hypothetical protein | Bcen2424_4977 |
| BCAM2210 | 0.14 | 1E-08 | BCAM2210 | hypothetical protein | Bcen2424_4978 |
| BCAM2217 | 0.04 | 3E-07 | No homolog | No gene annotation | No homolog |
| BCAM2289 | 0.39 | 3E-03 | BCAM2289 | hypothetical protein | No homolog |
| BCAM2330 | 0.11 | 2E-08 | BCAM2330 | hypothetical protein | Bcen2424_5089 |
| BCAM2353 | 0.47 | 3E-02 | BCAM2353 | hypothetical protein | Bcen2424_5110 |
| BCAM2361a | 0.28 | 2E-05 | BCAM2361A | coenzyme PQQ synthesis protein D | Bcen2424_5120 |
| BCAM2364 | 0.09 | 3E-08 | BCAM2364 | coenzyme PQQ synthesis protein A | No homolog |
| BCAM2400a | 0.01 | 1E-06 | BCAM2400a | hypothetical protein | Bcen2424_5161 |
| BCAM2400b | 0.01 | 1E-06 | No homolog | No gene annotation | No homolog |
| BCAM2402 | 0.30 | 3E-05 | BCAM2402 | hypothetical protein | Bcen2424_5164 |
| BCAM2403 | 0.28 | 8E-07 | BCAM2403 | hypothetical protein | No homolog |
| BCAM2413 | 0.50 | 8E-10 | BCAM2413 | putative GNAT family N-acetyltransferase | Bcen2424_5175 |
| BCAM2444 | 0.07 | 5E-08 | BCAM2444 | hypothetical protein | No homolog |
| BCAM2466 | 0.42 | 4E-04 | Multiple hits | No gene annotation | Multiple hits |
| BCAM2510 | 0.47 | 5E-06 | BCAM2510 | hypothetical protein | No homolog |
| BCAM2609 | 0.25 | 1E-12 | BCAM2609 | hypothetical protein* | Bcen2424_5415 |
| BCAM2621_J_0 | 0.26 | 4E-09 | BCAM2621 | No gene annotation | Bcen2424_5427 |
| BCAM2621_J_1 | 0.26 | 3E-09 | BCAM2621 | No gene annotation | Bcen2424_5427 |
| BCAM2623 | 0.40 | 4E-03 | BCAM2623 | hypothetical protein | Bcen2424_5428 |
| BCAM2653 | 0.23 | 1E-06 | BCAM2653 | hypothetical protein | No homolog |
| BCAM2657 | 0.10 | 2E-08 | No homolog | No gene annotation | No homolog |
| BCAM2666 | 0.12 | 1E-06 | BCAM2666 | putative permease protein | Bcen2424_5470 |
| BCAM2670 | 0.38 | 2E-05 | BCAM2670 | hypothetical protein | Bcen2424_5474 |
| BCAM2679 | 0.01 | 2E-06 | BCAM2679 | hypothetical protein | No homolog |
| BCAM2685 | 0.20 | 5E-10 | BCAM2685 | hypothetical protein | Bcen2424_5496 |
| BCAM2717 | 0.28 | 5E-12 | No homolog | No gene annotation | No homolog |
| BCAM2752 | 0.42 | 2E-04 | No homolog | No gene annotation | No homolog |
| BCAM2804 | 0.37 | 6E-04 | No homolog | No gene annotation | No homolog |
| BCAM2834 | 0.19 | 1E-10 | No homolog | No gene annotation | No homolog |
| BCAMr0727 | 0.11 | 2E-06 | BCAMr0727 | tRNA-OTHER | Bcen2424_R0074 |
| BCAS0018 | 0.22 | 1E-06 | No homolog | No gene annotation | No homolog |
| BCAS0020 | 0.40 | 2E-07 | No homolog | No gene annotation | No homolog |
| BCAS0126 | 0.23 | 3E-03 | No homolog | No gene annotation | No homolog |
| BCAS0160 | 0.49 | 3E-10 | BCAS0160 | hypothetical protein | Bcen2424_6069 |
| BCAS0177 | 0.46 | 4E-04 | BCAS0177 | hypothetical protein | No homolog |
| BCAS0193 | 0.48 | 8E-03 | No homolog | No gene annotation | Bcen2424_6032 |
| BCAS0194 | 0.34 | 6E-04 | BCAS0194 | hypothetical protein | No homolog |
| BCAS0205 | 0.42 | 4E-03 | BCAS0205 | TauD/TfdA taurine catabolism dioxygenase family protein | Bcen2424_6019 |
| BCAS0213 | 0.47 | 1E-02 | BCAS0213 | hypothetical protein | Bcen2424_6011 |
| BCAS0214 | 0.47 | 4E-03 | BCAS0214 | hypothetical protein | Bcen2424_6010 |
| BCAS0215 | 0.26 | 3E-04 | BCAS0215 | hypothetical protein | Bcen2424_6009 |
| BCAS0216 | 0.46 | 2E-02 | BCAS0216 | putative acyl carrier protein | Bcen2424_6008 |
| BCAS0217 | 0.49 | 1E-02 | BCAS0217 | hypothetical protein | No homolog |
| BCAS0219 | 0.38 | 1E-04 | BCAS0219 | hypothetical protein | Bcen2424_6005 |
| BCAS0238 | 0.13 | 3E-09 | BCAS0238 | hypothetical protein | Bcen2424_5981 |
| BCAS0257 | 0.39 | 1E-05 | BCAS0257 | putative acetyltransferase | No homolog |
| BCAS0406 | 0.36 | 8E-06 | BCAS0406 | hypothetical protein* | No homolog |
| BCAS0468a | 0.41 | 2E-04 | BCAS0468a | hypothetical protein | No homolog |
| BCAS0469 | 0.48 | 1E-05 | BCAS0469 | hypothetical protein | No homolog |
| BCAS0476 | 0.45 | 8E-07 | BCAS0476 | hypothetical protein | Bcen2424_6545 |
| BCAS0537 | 0.50 | 3E-06 | BCAS0537 | hypothetical protein | No homolog |
| BCAS0539 | 0.46 | 9E-03 | BCAS0539 | cro/cI repressor transcription regulator | No homolog |
| BCAS0572 | 0.04 | 1E-06 | BCAS0572 | hypothetical protein* | No homolog |
| BCAS0573 | 0.04 | 1E-07 | BCAS0573 | hypothetical protein | No homolog |
| BCAS0624 | 0.30 | 7E-05 | BCAS0624 | hypothetical protein | No homolog |
| BCAS0633 | 0.17 | 2E-06 | No homolog | No gene annotation | No homolog |
| BCAS0646_J_0 | 0.24 | 2E-11 | BCAS0646 | No gene annotation | No homolog |
| BCAS0649 | 0.44 | 4E-04 | BCAS0649 | hypothetical protein | No homolog |
| BCAS0669 | 0.02 | 4E-07 | BCAS0669 | hypothetical protein | No homolog |
| BCAS0679 | 0.47 | 1E-12 | Multiple hits | No gene annotation | No homolog |
| BCAS0683 | 0.07 | 6E-08 | BCAS0683 | No gene annotation | No homolog |
| BCAS0684_J_0 | 0.40 | 4E-03 | No homolog | No gene annotation | No homolog |
| BCAS0686 | 0.36 | 7E-04 | BCAS0686 | hypothetical protein | No homolog |
| BCAS0750 | 0.30 | 5E-04 | BCAS0750 | hypothetical protein | Bcen2424_6220 |
| HI2424_G6260 | 0.43 | 1E-11 | No homolog | No gene annotation | No homolog |
| IG2_1140182 | 0.48 | 1E-03 | BCAM1053a | putative phage DNA-binding protein | No homolog |
| IG3_284778 | 0.20 | 8E-11 | BCAS0257 | putative acetyltransferase | No homolog |
| pBCA003 | 0.46 | 2E-03 | pBCA003 | hypothetical protein | Bcen2424_6873 |
| pBCA007 | 0.41 | 2E-05 | Multiple hits | No gene annotation | No homolog |
| pBCA091 | 0.47 | 2E-13 | pBCA091 | hypothetical protein | No homolog |
| pBCA092 | 0.46 | 6E-13 | pBCA092 | hypothetical protein | No homolog |

^*^ contains predicted Tat signal peptide based on TatP predictions
