# Supplementary material for: Identification of Potential Therapeutic Targets for Burkholderia cenocepacia by Comparative Transcriptomics
Source: PLoS One. 2010 Jan 15;5(1):e8724. doi: 10.1371/journal.pone.0008724 (PMC2806911; doi:10.1371/journal.pone.0008724)
Supplement: Table S3 — Probes showing at least a 2-fold greater pixel intensity in J2315 versus HI2424 under CF conditions. List of genes induced in the clinical isolate J2315 compared to the soil isolate HI2424 under CF-like conditions. (3.36 MB DOC) [file pone.0008724.s004.doc]

Table S3. Probes showing at least a 2-fold greater pixel intensity in J2315 versus HI2424 under CF conditions

| **Probe ID** | **J2315/**  **HI2424 pixel intensity ratio** | **p-value** | **J2315 Gene** | **Annotation** | **HI2424 homolog** |
| --- | --- | --- | --- | --- | --- |
|  |  |  |  | ***Translation, ribosomal structure and biogenesis (J)*** |  |
| BCAL0209 | 2919 | 2E-02 | BCAL0209 | acetyltransferase (GNAT) family protein | Bcen2424_0323 |
| BCAL0225 | 2272 | 3E-02 | BCAL0225 | 50S ribosomal protein L7/L12 | Bcen2424_0339 |
| BCAL0250 | 3613 | 8E-02 | BCAL0250 | 50S ribosomal protein L18 | Bcen2424_0364 |
| BCAL0424 | 2066 | 2E-02 | BCAL0424 | ribonuclease P protein component | Bcen2424_3165 |
| BCAL0484 | 2489 | 2E-02 | BCAL0484 | glutamyl-tRNA amidotransferase subunit A | Bcen2424_3108 |
| BCAL0485 | 264 | 3E-02 | BCAL0485 | glutamyl-tRNA amidotransferase subunit B | No homolog |
| BCAL0745 | 2622 | 5E-02 | BCAL0745 | SpoU rRNA methylase family protein | Bcen2424_2851 |
| BCAL0799 | 385 | 2E-02 | BCAL0799 | ribosomal L25p family protein | Bcen2424_2803 |
| BCAL0867 | 2801 | 4E-02 | BCAL0867 | YjgF family protein | No homolog |
| BCAL1099 | 2612 | 1E-02 | BCAL1099 | putative release factor | Bcen2424_1217 |
| BCAL1448 | 2832 | 8E-02 | BCAL1448 | putative valyl-tRNA synthetase | Bcen2424_1441 |
| IG1_1598102 | 2599 | 2E-02 | BCAL1448 | putative valyl-tRNA synthetase | Bcen2424_1441 |
| BCAL1509 | 375 | 2E-01 | BCAL1509 | tRNA pseudouridine synthase B | No homolog |
| BCAL1865 | 2033 | 4E-02 | BCAL1865 | ribosomal large subunit pseudouridine synthase D | Bcen2424_1793 |
| BCAL1973 | 1834 | 1E-02 | BCAL1973 | endoribonuclease L-PSP family protein | No homolog |
| BCAL2059 | 3205 | 9E-02 | BCAL2059 | putative 2'-5' RNA ligase | No homolog |
| BCAL2076 | 2696 | 1E-00 | BCAL2076 | putative RNA methylase protein | No homolog |
| BCAL2104 | 3619 | 4E-02 | BCAL2104 | putative DNA methylase | Bcen2424_2033 |
| BCAL2739 | 3932 | 4E-02 | BCAL2739 | elongation factor G | Bcen2424_2528 |
| BCAL2926 | 2369 | 1E-02 | BCAL2926 | tRNA(guanine-N(1)-)-methyltransferase | Bcen2424_1071 |
| BCAL3257 | 486 | 4E-02 | BCAL3257 | putative tRNA delta(2)-isopentenylpyrophosphate transferase | No homolog |
| BCAL3343 | 6117 | 1E-02 | BCAL3343 | D-tyrosyl-tRNA | Bcen2424_0684 |
| IG1_3766524 | 48 | 1E-02 | BCAL3442 | 50S ribosomal protein L21 | Bcen2424_0581 |
| BCAM0265 | 2023 | 1E-02 | BCAM0265 | putative amidase | No homolog |
| BCAM1828 | 2464 | 3E-02 | BCAM1828 | putative amidase | Bcen2424_4689 |
| BCAM1839 | 9006 | 1E-02 | BCAM1839 | putative endoribonuclease | No homolog |
| BCAM2074 | 3515 | 1E-02 | BCAM2074 | putative acetyltransferase | Bcen2424_4881 |
| BCAM2131 | 3374 | 2E-02 | BCAM2131 | 2-aminomuconate deaminase | No homolog |
| BCAM2448 | 4062 | 2E-02 | BCAM2448 | putative GNAT family N-acetyltransferase | Bcen2424_5251 |
| BCAM2782 | 2024 | 2E-02 | BCAM2782 | putative acetyltransferase protein | No homolog |
| BCAS0245 | 3746 | 1E-02 | BCAS0245 | 30S ribosomal protein S21 3 | No homolog |
| BCAS0468 | 7842 | 6E-02 | BCAS0468 | putative acetyltransferase-GNAT family | Bcen2424_6553 |
|  |  |  |  | ***Transcription (K)*** |  |
| BCAL0053 | 5877 | 2E-02 | BCAL0053 | PadR family regulatory protein* | Bcen2424_0122 |
| BCAL0221 | 2093 | 5E-01 | BCAL0221 | transcription antitermination protein NusG | No homolog |
| BCAL0226 | 2046 | 3E-02 | BCAL0226 | DNA-directed RNA polymerase beta chain | Bcen2424_0340 |
| BCAL0260 | 218 | 3E-02 | BCAL0260 | DNA-directed RNA polymerase alpha chain | Bcen2424_0374 |
| BCAL0562 | 3972 | 5E-02 | BCAL0562 | negative regulator of flagellin synthesis (anti-sigma-28 factor) | Bcen2424_3027 |
| BCAL0625 | 235 | 9E-01 | BCAL0625 | LysR family regulatory protein | Bcen2424_2967 |
| BCAL0663 | 2196 | 3E-02 | BCAL0663 | LysR family regulatory protein | No homolog |
| BCAL0772 | 3411 | 1E-02 | BCAL0772 | AraC family regulatory protein | No homolog |
| BCAL0776 | 3904 | 8E-02 | BCAL0776 | LysR family regulatory protein | No homolog |
| BCAL1044 | 2322 | 2E-01 | BCAL1044 | GntR family regulatory protein | Bcen2424_1164 |
| BCAL1146 | 3989 | 3E-02 | BCAL1146 | AraC family regulatory protein | No homolog |
| BCAL1150 | 2247 | 9E-02 | BCAL1150 | AraC family regulatory protein | No homolog |
| BCAL1162 | 4009 | 1E-02 | BCAL1162 | TetR family regulatory protein | No homolog |
| BCAL1180 | 749 | 7E-02 | BCAL1180 | LysR family regulatory protein | No homolog |
| BCAL1182 | 2288 | 5E-02 | BCAL1182 | TetR family regulatory protein | No homolog |
| BCAL1217 | 3038 | 4E-02 | BCAL1217 | LysR family regulatory protein | No homolog |
| BCAL1343 | 2143 | 2E-02 | BCAL1343 | LysR family regulatory protein | No homolog |
| BCAL1477 | 2195 | 6E-02 | BCAL1477 | LysR family regulatory protein | Bcen2424_1470 |
| BCAL1503 | 2257 | 2E-02 | BCAL1503 | putative transcriptional regulator protein | Bcen2424_1496 |
| BCAL1595 | 2945 | 6E-02 | BCAL1595 | putative DNA-binding phage protein | No homolog |
| BCAL1656 | 3858 | 5E-02 | BCAL1656 | aliphatic sulfonate utilization regulatory protein SsuR | No homolog |
| BCAL2036 | 2018 | 8E-02 | BCAL2036 | LysR family regulatory protein | No homolog |
| BCAL2309 | 2541 | 5E-02 | BCAL2309 | putative copper-related MerR family regulatory protein | No homolog |
| BCAL2488 | 1269 | 4E-02 | BCAL2488 | LysR family regulatory protein | No homolog |
| BCAL2490 | 9075 | 9E-02 | BCAL2490 | LysR family regulatory protein | No homolog |
| BCAL2518 | 1371 | 6E-02 | BCAL2518 | putative DNA-binding protein | No homolog |
| BCAL2523 | 8583 | 3E-02 | BCAL2523 | LysR family regulatory protein | No homolog |
| BCAL2524 | 7729 | 8E-02 | BCAL2524 | AraC family regulatory protein | No homolog |
| BCAL2527 | 4057 | 5E-02 | BCAL2526a | MerR family regulatory protein | No homolog |
| BCAL2529 | 6033 | 3E-02 | BCAL2529 | LysR family regulatory protein | No homolog |
| IG1_2797564 | 6309 | 8E-02 | BCAL2529 | LysR family regulatory protein | No homolog |
| BCAL2537 | 3566 | 2E-02 | BCAL2537 | LysR family regulatory protein | No homolog |
| BCAL2540 | 1483 | 6E-02 | BCAL2540 | LysR family regulatory protein | No homolog |
| BCAL2544 | 1541 | 8E-22 | BCAL2544 | TetR family regulatory protein | No homolog |
| BCAL2559 | 3373 | 3E-02 | BCAL2559 | putative transcriptional regulator | No homolog |
| BCAL2561 | 2433 | 2E-02 | BCAL2561 | LysR family regulatory protein | No homolog |
| BCAL2577 | 105 | 2E-02 | BCAL2577 | DJ-1/PfpI family protein | No homolog |
| BCAL2579 | 7737 | 3E-02 | BCAL2579 | LysR family regulatory protein | No homolog |
| BCAL2586 | 837 | 9E-02 | BCAL2586 | AraC family regulatory protein | No homolog |
| BCAL2686 | 3346 | 4E-02 | BCAL2686 | cys regulon transcriptional activator CysB | Bcen2424_2476 |
| BCAL2688 | 2844 | 3E-02 | BCAL2688 | LysR family regulatory protein | No homolog |
| BCAL3009 | 2395 | 1E-02 | BCAL3009 | transcription elongation factor | Bcen2424_1002 |
| BCAL3055 | 3374 | 8E-02 | BCAL3055 | probable N utilization substance protein B | Bcen2424_0955 |
| BCAL3076A | 1029 | 4E-02 | BCAL3076A | putative DNA-binding protein | No homolog |
| BCAL3088 | 2147 | 8E-02 | BCAL3088 | LysR family regulatory protein | No homolog |
| BCAL3151 | 4331 | 1E-02 | BCAL3151 | hypothetical protein | Bcen2424_0855 |
| BCAL3152 | 2108 | 1E-02 | BCAL3152 | putative RNA polymerase sigma factor | Bcen2424_0854 |
| BCAL3213 | 4366 | 1E-02 | BCAL3213 | AraC family regulatory protein | Bcen2424_0789 |
| BCAL3275 | 2061 | 7E-02 | BCAL3275 | putative heat-inducible transcription repressor | No homolog |
| BCAL3320 | 1249 | 3E-01 | BCAL3320 | ArsR family regulatory protein | No homolog |
| BCAL3479 | 2987 | 2E-02 | BCAL3479 | putative transmembrane regulator | No homolog |
| BCAM0001 | 8136 | 1E-02 | BCAM0001 | putative sigma factor | Bcen2424_5690 |
| BCAM0004 | 2968 | 2E-02 | BCAM0004 | putative partitioning protein ParB | Bcen2424_5694 |
| BCAM0014 | 2803 | 2E-02 | BCAM0014 | TetR family regulatory protein | No homolog |
| BCAM0040 | 3385 | 1E-02 | BCAM0040 | AraC family regulatory protein | No homolog |
| BCAM0076 | 2348 | 3E-02 | BCAM0076 | TetR family regulatory protein | Bcen2424_5773 |
| BCAM0085 | 2311 | 5E-02 | BCAM0085 | TetR family regulatory protein | No homolog |
| BCAM0156 | 4509 | 2E-02 | BCAM0156 | transcriptional regulator KdgR | No homolog |
| BCAM0161 | 4515 | 8E-02 | BCAM0161 | AraC family regulatory protein | No homolog |
| BCAM0176 | 9478 | 2E-02 | BCAM0176 | AnsC family regulatory protein | No homolog |
| BCAM0233 | 201 | 6E-02 | BCAM0233 | ArsR family regulatory protein | No homolog |
| BCAM0240 | 1396 | 2E-02 | BCAM0240 | N-acylhomoserine lactone dependent regulatory protein | No homolog |
| BCAM0256 | 2311 | 1E-02 | BCAM0256 | epidemic strain marker regulator | No homolog |
| BCAM0259 | 1096 | 5E-02 | BCAM0259 | putative repressor protein | No homolog |
| BCAM0342 | 3266 | 2E-02 | BCAM0342 | LysR family regulatory protein | No homolog |
| BCAM0412 | 4818 | 4E-02 | BCAM0412 | DeoR family regulatory protein | No homolog |
| BCAM0464 | 3404 | 6E-02 | BCAM0464 | LysR family regulatory protein | No homolog |
| BCAM0489 | 2082 | 1E-02 | BCAM0489 | MerR family regulatory protein | No homolog |
| BCAM0605 | 252 | 6E-02 | BCAM0605 | AnsC family regulatory protein | Bcen2424_3578 |
| BCAM0615 | 3182 | 5E-02 | BCAM0615 | TetR family regulatory protein* | No homolog |
| BCAM0764 | 2928 | 2E-02 | BCAM0764 | LacI family regulatory protein | No homolog |
| BCAM0780 | 1191 | 2E-02 | BCAM0780 | putative helicase | No homolog |
| BCAM0809 | 6212 | 1E-01 | BCAM0809 | AraC family regulatory protein | No homolog |
| BCAM0868 | 5998 | 5E-02 | BCAM0868 | AraC family regulatory protein | Bcen2424_3835 |
| BCAM1177 | 3461 | 1E-02 | BCAM1177 | IclR family regulatory protein | No homolog |
| BCAM1189 | 3621 | 4E-02 | BCAM1189 | LysR family regulatory protein | Bcen2424_4063 |
| IG2_1311396 | 2228 | 4E-02 | BCAM1201 | LysR family regulatory protein | Bcen2424_4075 |
| BCAM1254 | 2038 | 6E-02 | BCAM1254 | MarR family regulatory protein | Bcen2424_4128 |
| BCAM1276 | 2231 | 7E-02 | BCAM1276 | ArsR family regulatory protein | No homolog |
| BCAM1280 | 2081 | 2E-02 | BCAM1280 | putative GntR-family transcriptional regulator/aminotransferase | No homolog |
| BCAM1415 | 9545 | 1E-02 | BCAM1415 | AraC family regulatory protein | Bcen2424_4280 |
| BCAM1453 | 2667 | 2E-02 | BCAM1453 | LysR family regulatory protein | Bcen2424_4325 |
| BCAM1546 | 1468 | 2E-01 | BCAM1546 | hypothetical protein | No homolog |
| BCAM1650 | 4388 | 6E-02 | BCAM1650 | LysR family regulatory protein | No homolog |
| BCAM1681 | 2342 | 1E-02 | BCAM1681 | LysR family regulatory protein* | No homolog |
| BCAM1750 | 3087 | 1E-02 | BCAM1750 | MarR family regulatory protein | No homolog |
| BCAM1768 | 3357 | 1E-02 | BCAM1768 | hypothetical protein | No homolog |
| BCAM1796 | 5182 | 6E-01 | BCAM1796 | LysR family regulatory protein | No homolog |
| BCAM1810 | 1424 | 9E-02 | BCAM1810 | putative cold shock protein | Bcen2424_5888 |
| BCAM1879 | 8269 | 4E-02 | BCAM1879 | hypothetical protein | No homolog |
| BCAM1992 | 2038 | 3E-02 | BCAM1992 | LysR family regulatory protein | No homolog |
| BCAM2105 | 2241 | 8E-01 | BCAM2105 | MerR family regulatory protein | No homolog |
| BCAM2117 | 2952 | 9E-02 | BCAM2117 | LysR family regulatory protein | No homolog |
| BCAM2118 | 5971 | 3E-02 | BCAM2118 | LysR family regulatory protein | No homolog |
| BCAM2137 | 884 | 8E-02 | BCAM2137 | LysR family regulatory protein | No homolog |
| BCAM2231 | 2266 | 7E-02 | BCAM2231 | transcriptional regulator PchR | No homolog |
| BCAM2265 | 2634 | 1E-02 | BCAM2265 | LysR family regulatory protein | No homolog |
| BCAM2332 | 2942 | 1E-02 | BCAM2332 | LysR family regulatory protein | No homolog |
| BCAM2393 | 2039 | 9E-02 | BCAM2393 | AraC family regulatory protein | No homolog |
| BCAM2548 | 9864 | 2E-02 | BCAM2548 | TetR family regulatory protein | No homolog |
| BCAM2575 | 3124 | 2E-02 | BCAM2575 | LysR family regulatory protein | No homolog |
| BCAM2592 | 1605 | 7E-02 | BCAM2592 | IclR family regulatory protein | No homolog |
| BCAM2816 | 3008 | 9E-01 | BCAM2816 | GntR family regulatory protein | No homolog |
| BCAS0005 | 218 | 1E-02 | BCAS0005 | AraC family regulatory protein | No homolog |
| BCAS0036 | 2927 | 2E-02 | BCAS0036 | LysR family regulatory protein | No homolog |
| BCAS0067 | 1749 | 6E-02 | BCAS0067 | AraC family regulatory protein | No homolog |
| BCAS0107 | 2458 | 4E-02 | BCAS0107 | LysR family regulatory protein | No homolog |
| BCAS0123 | 1537 | 6E-02 | BCAS0123 | putative transcriptional regulator | No homolog |
| BCAS0169 | 2809 | 6E-02 | BCAS0169 | LysR family regulatory protein | No homolog |
| BCAS0225 | 1531 | 2E-02 | BCAS0225 | LysR family regulatory protein | Bcen2424_5999 |
| BCAS0252 | 6405 | 2E-02 | BCAS0252 | DJ-1/PfpI family protein | Bcen2424_5950 |
| BCAS0258 | 4707 | 3E-02 | BCAS0258 | GntR family regulatory protein | No homolog |
| BCAS0283 | 2413 | 2E-16 | BCAS0283 | LysR family regulatory protein | No homolog |
| BCAS0373 | 3012 | 2E-02 | BCAS0373 | LacI family regulatory protein | Bcen2424_6695 |
| BCAS0376 | 4128 | 9E-02 | BCAS0376 | GntR family regulatory protein | No homolog |
| BCAS0467 | 2614 | 2E-02 | BCAS0467 | DeoR family regulatory protein | No homolog |
| BCAS0503 | 4539 | 5E-02 | BCAS0503 | LysR family regulatory protein | No homolog |
| BCAS0543 | 4936 | 7E-01 | BCAS0543 | putative phage transcriptional regulator | No homolog |
| BCAS0688 | 276 | 1E-01 | BCAS0688 | TetR family regulatory protein | No homolog |
| BCAS0697 | 7604 | 4E-02 | BCAS0697 | LysR family regulatory protein | No homolog |
| BCAS0710 | 2924 | 3E-01 | BCAS0710 | LysR family regulatory protein | No homolog |
| BCAS0715 | 9667 | 2E-01 | BCAS0715 | LysR family regulatory protein | No homolog |
| BCAS0717 | 2193 | 9E-02 | BCAS0717 | hypothetical protein | No homolog |
| pBCA035 | 2781 | 5E-02 | pBCA035 | GntR family regulatory protein | No homolog |
|  |  |  |  | ***Replication, recombination, and repair (L)*** |  |
| BCAL0165 | 3581 | 6E-02 | BCAL0165 | putative plasmid replication-associated protein | No homolog |
| BCAL0178 | 1141 | 2E-02 | BCAL0178 | putative DNA methyltransferase | No homolog |
| BCAL0462 | 2446 | 7E-02 | BCAL0462 | putative DNA topoisomerase III | Bcen2424_3130 |
| BCAL0828 | 245 | 4E-02 | BCAL0828 | putative G/U mismatch-specific DNA glycosylase | Bcen2424_2776 |
| BCAL0953 | 2413 | 7E-02 | BCAL0953 | putative recombinase A | No homolog |
| BCAL1118 | 3515 | 5E-02 | BCAL1118 | putative integrase | No homolog |
| BCAL1204 | 6125 | 2E-02 | BCAL1204 | putative helicase | No homolog |
| BCAL1315 | 2158 | 6E-02 | BCAL1315 | hypothetical protein | No homolog |
| BCAL1585 | 6678 | 8E-01 | BCAL1585 | histone-like DNA-binding phage protein | No homolog |
| BCAL1755 | 2603 | 3E-02 | BCAL1755 | hypothetical protein | No homolog |
| BCAL1891 | 2261 | 1E-02 | BCAL1891 | hypothetical protein | No homolog |
| BCAL2077 | 2275 | 2E-02 | BCAL2077 | ribonuclease HII | No homolog |
| BCAL2096 | 2347 | 3E-02 | BCAL2096 | DNA ligase | Bcen2424_2024 |
| BCAL2114 | 4805 | 1E-02 | BCAL2114 | uracil DNA glycosylase superfamily protein | Bcen2424_2043 |
| BCAL2252 | 2611 | 4E-00 | BCAL2252 | putative DNA photolyase | No homolog |
| IG1_2495977 | 4895 | 1E-01 | BCAL2252 | putative DNA photolyase | No homolog |
| BCAL2563 | 3855 | 2E-02 | BCAL2563 | putative IstB-like insertion sequence protein | No homolog |
| BCAL2564 | 2553 | 2E-02 | BCAL2564 | putative transposase for insertion sequence IS408 | No homolog |
| BCAL2758 | 2125 | 4E-01 | BCAL2758 | putative exodeoxyribonuclease VII large subunit | Bcen2424_2544 |
| BCAL2943 | 2001 | 2E-01 | BCAL2943 | hypothetical protein* | Bcen2424_1054 |
| BCAL2961 | 2472 | 2E-02 | BCAL2961 | putative integrase | No homolog |
| BCAL3141 | 25 | 3E-02 | BCAL3141 | hypothetical protein | Bcen2424_0865 |
| BCAL3252 | 7308 | 5E-02 | BCAL3252 | putative transposase | No homolog |
| BCAL3254 | 4558 | 7E-02 | BCAL3254 | putative transposase | No homolog |
| BCAM0007 | 3074 | 5E-02 | BCAM0007 | putative phage integrase | Bcen2424_5698 |
| BCAM0771 | 7548 | 9E-02 | BCAM0771 | putative transposase | No homolog |
| BCAM1024 | 6496 | 9E-02 | BCAM1024 | putative phage integrase | No homolog |
| IG2_1380867 | 2178 | 4E-02 | BCAM1258 | putative DNA-binding protein | No homolog |
| BCAS0551 | 1473 | 3E-02 | BCAS0551 | HU DNA-binding protein | No homolog |
| BCAS0652 | 7164 | 9E-02 | BCAS0652 | putative transposase | No homolog |
| BCAS0660 | 3417 | 2E-02 | BCAS0660 | putative transposase | No homolog |
| pBCA057 | 1413 | 7E-02 | pBCA057 | putative conjugative transfer protein | No homolog |
| pBCA090 | 7946 | 4E-02 | pBCA090 | putative integrase | No homolog |
|  |  |  |  | ***Defense mechanisms (V)*** |  |
| BCAL0418 | 6584 | 1E-03 | BCAL0418 | type I restriction enzyme specificity protein | No homolog |
| BCAL0420 | 5589 | 2E-02 | BCAL0420 | type I restriction component of type I restriction-modification system | No homolog |
| BCAL1176 | 6625 | 4E-02 | BCAL1176 | putative fusaric acid resistance transport protein* | No homolog |
| BCAL1675 | 4609 | 2E-02 | BCAL1675 | multidrug efflux system transporter protein AmrB | Bcen2424_1623 |
| BCAL2408 | 1128 | 5E-02 | BCAL2408 | lipid A export ATP-binding/permease protein MsbA | No homolog |
| BCAL3091 | 2028 | 2E-02 | BCAL3091 | putative undecaprenol kinase | No homolog |
| BCAM1421 | 1656 | 2E-02 | BCAM1421 | RND family efflux system transporter protein | No homolog |
| BCAM1520 | 5417 | 4E-02 | BCAM1520 | putative restriction endonuclease | No homolog |
| BCAM1779 | 3902 | 3E-02 | BCAM1779 | putative beta-lactamase, class A* | Bcen2424_4599 |
| BCAM2140 | 3997 | 5E-02 | BCAM2140 | transporter system transport protein* | No homolog |
| BCAM2141 | 5551 | 4E-02 | BCAM2141 | ABC transporter ATP-binding membrane protein | No homolog |
| BCAM2634a | 2277 | 3E-01 | BCAM2634a | putative chloramphenicol resistance-related protein | No homolog |
| BCAS0081 | 202 | 2E-02 | BCAS0081 | ABC transporter ATP-binding membrane protein | Bcen2424_6140 |
| BCAS0203 | 5964 | 5E-02 | BCAS0203 | ABC transporter protein | Bcen2424_6021 |
| BCAS0204 | 8933 | 7E-02 | BCAS0204 | ABC transporter ATP-binding protein* | Bcen2424_6020 |
| BCAS0716 | 813 | 2E-02 | BCAS0716 | putative restriction endonuclease | No homolog |
|  |  |  |  | ***Signal transduction mechanisms (T)*** |  |
| BCAL0128 | 3744 | 3E-01 | BCAL0128 | chemotaxis two-component response regulator CheY | Bcen2424_0255 |
| BCAL0135 | 2175 | 3E-01 | BCAL0135 | chemotaxis protein CheY | Bcen2424_0262 |
| BCAL0376 | 222 | 3E-02 | BCAL0376 | two-component regulatory system, response regulator protein | Bcen2424_0512 |
| BCAL0534 | 3371 | 1E-02 | BCAL0534 | two-component regulatory system, response regulator protein | Bcen2424_3055 |
| BCAL0535 | 2156 | 2E-02 | BCAL0535 | sensor kinase protein | Bcen2424_3054 |
| BCAL1381 | 3507 | 3E-01 | BCAL1381 | LuxR superfamily regulatory protein | No homolog |
| BCAL1635 | 4866 | 4E-02 | BCAL1635 | putative cyclic-di-GMP signaling protein | No homolog |
| BCAL1684 | 2015 | 1E-02 | BCAL1684 | two-component regulatory system, response regulator protein | No homolog |
| BCAL2200 | 2818 | 1E-02 | BCAL2200 | low molecular weight phosphotyrosine protein phosphatase | Bcen2424_2128 |
| BCAM0050 | 5776 | 6E-02 | BCAM0050 | universal stress-related protein | Bcen2424_5738 |
| BCAM0110 | 2797 | 8E-02 | BCAM0110 | two-component regulatory system, sensor kinase protein | No homolog |
| BCAM0221 | 9238 | 9E-02 | BCAM0221 | two-component regulatory system, response regulator protein | No homolog |
| BCAM0227 | 7369 | 4E-02 | BCAM0227 | hybrid two-component system kinase-response regulator protein | No homolog |
| BCAM0234 | 2192 | 3E-02 | BCAM0234 | putative arsenate reductase | No homolog |
| BCAM0239a | 2232 | 1E-02 | BCAM0239a | N-acylhomoserine lactone synthase | No homolog |
| BCAM0287 | 3169 | 1E-02 | BCAM0287 | CRP family regulatory protein | No homolog |
| BCAM0288 | 6237 | 2E-02 | BCAM0288 | two-component regulatory system, response regulator protein | No homolog |
| BCAM0289 | 7447 | 2E-01 | BCAM0289 | two-component regulatory system, sensor kinase protein | No homolog |
| BCAM0628 | 2854 | 9E-02 | BCAM0628 | putative low molecular weight phosphotyrosine protein phosphatase* | No homolog |
| BCAM0820 | 2053 | 5E-02 | BCAM0820 | hybrid two-component system kinase-response regulator protein | Bcen2424_3785 |
| BCAM0884 | 1387 | 6E-02 | BCAM0884 | two-component regulatory system, sensor kinase protein | No homolog |
| BCAM1310 | 3742 | 2E-02 | BCAM1310 | putative transcription antitermination regulator* | No homolog |
| BCAM1417 | 9065 | 4E-02 | BCAM1417 | two-component regulatory system, sensor kinase protein | No homolog |
| BCAM1418 | 4135 | 9E-02 | BCAM1418 | two-component regulatory system, response regulator protein | Bcen2424_4290 |
| BCAM1500 | 4046 | 1E-02 | BCAM1500 | putative universal stress protein | No homolog |
| BCAM1545 | 5846 | 2E-02 | BCAM1545 | LuxR superfamily regulatory protein | No homolog |
| BCAM1802 | 2319 | 8E-02 | BCAM1802 | putative regulatory protein | Bcen2424_4663 |
| BCAM1934 | 4015 | 6E-02 | BCAM1934 | LuxR superfamily regulatory protein* | No homolog |
| BCAM2418 | 5447 | 9E-02 | BCAM2418 | putative haemagglutinin-related autotransporter protein* | No homolog |
| BCAM2426 | 4744 | 4E-02 | BCAM2426 | putative diguanylate phosphodiesterase | Bcen2424_5231 |
| BCAM2756 | 2106 | 3E-02 | BCAM2756 | two-component regulatory system, response regulator protein | No homolog |
| BCAM2757 | 6213 | 7E-02 | BCAM2757 | two-component regulatory system, sensor kinase protein* | No homolog |
| BCAM2836 | 2723 | 9E-02 | BCAM2836 | putative diguanylate cyclase | No homolog |
| BCAS0070 | 4276 | 1E-02 | BCAS0070 | two-component regulatory system, response regulator protein | No homolog |
| BCAS0234 | 2508 | 7E-01 | BCAS0234 | hybrid two-component system kinase-response regulator protein* | Bcen2424_5987 |
| BCAS0235 | 4214 | 2E-02 | BCAS0235 | two-component regulatory system, response regulator protein | No homolog |
| BCAS0707 | 4982 | 9E-17 | BCAS0707 | two-component regulatory system, response regulator protein | No homolog |
| BCAS0708 | 1185 | 5E-02 | BCAS0708 | two-component regulatory system, sensor kinase protein | No homolog |
| BCAS0709 | 1832 | 2E-01 | BCAS0709 | two-component regulatory system, response regulator protein | No homolog |
| pBCA055 | 7567 | 7E-02 | pBCA055 | hypothetical protein | No homolog |
|  |  |  |  | ***Cell wall/membrane/envelope biogenesis (M)*** |  |
| BCAL0025 | 2007 | 2E-02 | BCAL0025 | methyltransferase GidB | No homolog |
| BCAL0067 | 3068 | 2E-02 | BCAL0067 | hypothetical protein | No homolog |
| BCAL0110 | 1694 | 5E-02 | BCAL0110 | putative aminotransferase | No homolog |
| BCAL0479 | 2566 | 9E-02 | BCAL0479 | penicillin-binding protein | No homolog |
| BCAL0508 | 2383 | 1E-03 | BCAL0508 | putative lipid A biosynthesis acyltransferase | Bcen2424_3082 |
| BCAL0894 | 2884 | 2E-02 | BCAL0894 | hypothetical protein* | No homolog |
| BCAL1050 | 2942 | 2E-01 | BCAL1050 | putative glycosyltransferase | Bcen2424_1170 |
| BCAL1147 | 3119 | 5E-02 | BCAL1147 | glycosyltransferase | No homolog |
| BCAL1149 | 2795 | 4E-01 | BCAL1149 | hypothetical protein | No homolog |
| BCAL1178 | 2801 | 6E-02 | BCAL1178 | putative fusaric acid resistance outer membrane efflux protein* | No homolog |
| BCAL1258 | 2226 | 1E-03 | BCAL1258 | putative exported transglycosylase protein* | No homolog |
| BCAL1368 | 3551 | 4E-02 | BCAL1368 | putative porin* | Bcen2424_1357 |
| BCAL1395 | 2834 | 4E-02 | BCAL1395 | putative cellulose synthase catalytic subunit (UDP-forming) | Bcen2424_1385 |
| BCAL1512 | 4342 | 8E-02 | BCAL1512 | outer membrane efflux protein* | Bcen2424_1505 |
| BCAL1671 | 3166 | 3E-02 | BCAL1671 | metallo peptidase, subfamily M23B | No homolog |
| BCAL1674 | 2609 | 3E-02 | BCAL1674 | multidrug efflux system AmrA protein* | No homolog |
| BCAL1676 | 3483 | 2E-02 | BCAL1676 | multidrug efflux system outer membrane protein* | Bcen2424_1624 |
| BCAL2078 | 4038 | 1E-01 | BCAL2078 | putative lipid-A-disaccharide synthase | Bcen2424_2006 |
| BCAL2082 | 2179 | 4E-02 | BCAL2082 | chaperone protein Skp precursor* | Bcen2424_2010 |
| BCAL2083 | 3562 | 6E-02 | BCAL2083 | Outer membrane protein assembly factor YaeT* | Bcen2424_2011 |
| BCAL2166 | 2004 | 4E-02 | BCAL2166 | putative lipoprotein* | Bcen2424_2094 |
| BCAL2370 | 5973 | 4E-02 | BCAL2370 | hypothetical protein* | No homolog |
| BCAL2402 | 309 | 5E-02 | BCAL2402 | putative LPS biosynthesis-related protein | No homolog |
| BCAL2403 | 1806 | 3E-02 | BCAL2403 | putative LPS core biosynthesis protein | No homolog |
| BCAL2404 | 8343 | 3E-02 | BCAL2404 | putative glycosyltransferase | No homolog |
| BCAL2405 | 1636 | 1E-02 | BCAL2405 | hypothetical protein | No homolog |
| BCAL2406 | 6167 | 5E-02 | BCAL2406 | putative glycosyltransferase | No homolog |
| BCAL2407 | 2181 | 2E-02 | BCAL2407 | putative glycosyltransferase | No homolog |
| BCAL2482 | 2238 | 7E-02 | BCAL2482 | putative outer membrane protein* | No homolog |
| BCAL2944 | 2846 | 3E-02 | BCAL2944 | ADP-l-glycero-D-manno-heptose-6-epimerase | Bcen2424_1053 |
| BCAL2946 | 2858 | 1E-02 | BCAL2946 | putative UDP-glucose dehydrogenase | Bcen2424_1051 |
| BCAL3073 | 2382 | 9E-02 | BCAL3073 | hypothetical protein | Bcen2424_0933 |
| BCAL3110 | 2567 | 8E-02 | BCAL3110 | putative 3-deoxy-D-manno-octulosonic acid transferase | No homolog |
| BCAL3116 | 2922 | 1E-01 | BCAL3116 | glycosyltransferase | No homolog |
| BCAL3119 | 3106 | 5E-02 | BCAL3119 | nucleotide sugar epimerase/dehydratase* | No homolog |
| BCAL3120 | 2206 | 1E-02 | BCAL3120 | UDP-N-acetylglucosamine-1-P transferase | No homolog |
| BCAL3121 | 208 | 3E-02 | BCAL3121 | putative nucleotide sugar epimerase/dehydratase | No homolog |
| BCAL3123 | 1333 | 6E-02 | BCAL3123 | acetyltransferase | No homolog |
| BCAL3124 | 908 | 5E-02 | BCAL3124 | glycosyltransferase | No homolog |
| BCAL3128 | 6542 | 1E-03 | BCAL3128 | glycosyltransferase | No homolog |
| BCAL3129 | 2386 | 2E-02 | BCAL3129 | nucleotide sugar aminotransferase | No homolog |
| BCAL3132 | 1761 | 3E-02 | BCAL3132 | dTDP-4-keto-L-rhamnose reductase | No homolog |
| BCAL3133 | 8599 | 1E-02 | BCAL3133 | dTDP-4-keto-6-deoxy-D-glucose 3,5-epimerase | Bcen2424_0873 |
| BCAL3134 | 2955 | 2E-02 | BCAL3134 | glucose-1-phosphate thymidylyltransferase | Bcen2424_0872 |
| BCAL3149 | 5983 | 4E-02 | BCAL3149 | hypothetical protein | No homolog |
| BCAL3219 | 3322 | 5E-02 | BCAL3219 | UDP-3-O-[3-hydroxymyristoyl] N-acetylglucosamine deacetylase | No homolog |
| BCAL3221 | 2095 | 9E-01 | BCAL3221 | putative capsular polysaccharide biosynthesis sulfatase membrane protein | No homolog |
| BCAL3239 | 2274 | 2E-02 | BCAL3239 | glucosyltransferase | No homolog |
| BCAL3242 | 2392 | 3E-02 | BCAL3242 | putative capsule polysaccharide export protein, ABC transporter membrane protein* | No homolog |
| BCAL3243 | 2721 | 2E-02 | BCAL3243 | putative capsular polysaccharide biosynthesis/export protein* | No homolog |
| BCAL3244 | 1042 | 9E-02 | BCAL3244 | glycosyltransferase | No homolog |
| BCAL3245 | 3521 | 3E-02 | BCAL3245 | capsule polysaccharide export protein | No homolog |
| BCAL3246 | 1016 | 1E-02 | BCAL3246 | putative GDP-mannose pyrophosphorylase | No homolog |
| BCAL3247 | 3452 | 7E-02 | BCAL3247 | mechanosensitive ion channel protein | Bcen2424_0768 |
| BCAL3460 | 22 | 5E-02 | BCAL3460 | D-alanine--D-alanine ligase B | Bcen2424_0561 |
| BCAM0071 | 125 | 4E-02 | BCAM0071 | puatative mandelate racemase/muconate lactonizing enzyme | No homolog |
| BCAM0169 | 2851 | 1E-02 | BCAM0169 | NmrA-like family protein | No homolog |
| BCAM0407 | 4046 | 3E-02 | BCAM0407 | putative porin* | No homolog |
| BCAM0854 | 2759 | 1E-02 | BCAM0854 | bifunctional exopolysaccharide biosynthesis protein (phosphomannose isomerase and GDP-D-mannose pyrophosphorylase) | Bcen2424_3819 |
| BCAM0855 | 2301 | 8E-02 | BCAM0855 | UDP-glucose dehydrogenase | Bcen2424_3821 |
| BCAM0859 | 2313 | 1E-02 | BCAM0859 | tyrosine-protein kinase | Bcen2424_3824 |
| BCAM0861 | 2068 | 5E-02 | BCAM0861 | putative glycosyltransferase | Bcen2424_3826 |
| BCAM1335 | 3345 | 3E-02 | BCAM1335 | glycosyltransferase | No homolog |
| BCAM1337 | 2264 | 1E-02 | BCAM1337 | glycosyltransferase | No homolog |
| BCAM1398 | 4545 | 5E-02 | BCAM1398 | putative porin* | No homolog |
| BCAM1419 | 1241 | 5E-02 | BCAM1419 | efflux system outer membrane protein* | Bcen2424_4291 |
| BCAM1420 | 3617 | 4E-02 | BCAM1420 | efflux system transport protein | No homolog |
| BCAM1472 | 474 | 8E-01 | BCAM1472 | putative glycosyltransferase | No homolog |
| BCAM1514 | 3087 | 5E-02 | BCAM1514 | putative outer membrane protein* | No homolog |
| BCAM1543 | 4457 | 7E-02 | BCAM1543 | putative glycosyltransferase* | No homolog |
| BCAM1576 | 2912 | 1E-02 | BCAM1576 | phosphoesterase family protein* | Bcen2424_4444 |
| BCAM1795 | 3997 | 3E-02 | BCAM1795 | NAD-dependent epimerase/dehydratase | No homolog |
| BCAM2112 | 5051 | 8E-02 | BCAM2112 | NAD dependent epimerase/dehydratase family protein | No homolog |
| BCAM2142 | 7772 | 5E-02 | BCAM2142 | transport system outer membrane protein* | No homolog |
| BCAM2253 | 2901 | 5E-02 | BCAM2253 | RHS-family protein | No homolog |
| BCAS0105 | 6436 | 6E-02 | BCAS0105 | hypothetical protein* | No homolog |
| BCAS0121 | 2757 | 1E-02 | BCAS0121 | putative porin protein* | No homolog |
| BCAS0220 | 2071 | 5E-02 | BCAS0220 | putative permease* | No homolog |
| BCAS0663 | 3576 | 4E-02 | BCAS0663 | RHS-family protein | No homolog |
| BCAS0692 | 2648 | 9E-01 | BCAS0692 | putative LrgB family membrane protein | No homolog |
| BCAS0704 | 2573 | 2E-02 | BCAS0704 | putative short-chain dehydrogenase/oxidoreductase | No homolog |
| pBCA049 | 2414 | 6E-02 | pBCA049 | putative transglycosylase protein* | No homolog |
| pBCA086 | 933 | 2E-02 | pBCA086 | putative UDP-N-acetylenolpyruvoylglucosamine reductase | No homolog |
|  |  |  |  | ***Cell motility (N)*** |  |
| BCAL0113 | 2143 | 2E-02 | BCAL0113 | B-type flagellar hook-associated protein 2 (HAP2) | Bcen2424_0240 |
| BCAL0114 | 1739 | 3E-02 | BCAL0114 | flagellin (type II) | No homolog |
| BCAL0126 | 3428 | 2E-02 | BCAL0126 | chemotaxis protein MotA | Bcen2424_0253 |
| BCAL0127 | 4436 | 6E-02 | BCAL0127 | chemotaxis protein MotB | Bcen2424_0254 |
| BCAL0129 | 5292 | 1E-02 | BCAL0129 | chemotaxis two-component sensor kinase CheA | Bcen2424_0256 |
| BCAL0131 | 4795 | 6E-02 | BCAL0131 | methyl-accepting chemotaxis protein | No homolog |
| BCAL0132 | 423 | 2E-02 | BCAL0132 | chemotaxis protein methyltransferase | No homolog |
| BCAL0140 | 5256 | 2E-01 | BCAL0140 | flagellar biosynthetic protein FlhB | Bcen2424_0267 |
| BCAL0521 | 4148 | 1E-02 | BCAL0521 | flagellar FliJ protein | Bcen2424_3068 |
| BCAL0522 | 4208 | 1E-02 | BCAL0522 | flagellum-specific ATP synthase FliI* | Bcen2424_3067 |
| BCAL0523 | 4101 | 3E-02 | BCAL0523 | flagellar assembly protein FliH | Bcen2424_3066 |
| BCAL0524 | 5423 | 6E-01 | BCAL0524 | flagellar motor switch protein FliG | Bcen2424_3065 |
| BCAL0525 | 2824 | 3E-01 | BCAL0525 | flagellar M-ring protein FliF | Bcen2424_3064 |
| BCAL0526 | 5222 | 5E-02 | BCAL0526 | flagellar hook-basal body complex protein FliE* | Bcen2424_3063 |
| BCAL0571 | 3853 | 3E-02 | BCAL0571 | flagellar P-ring protein precursor (basal body P-ring protein)* | Bcen2424_3018 |
| BCAL2636 | 1675 | 7E-02 | BCAL2636 | putative fimbriae chaperone* | No homolog |
| BCAL2637 | 2631 | 2E-02 | BCAL2637 | putative fimbriae usher protein* | No homolog |
| BCAL3505 | 7783 | 3E-02 | BCAL3505 | flagellar motor switch protein FliN* | Bcen2424_0039 |
| BCAL3506 | 3038 | 6E-02 | BCAL3506 | flagellar motor switch protein FliM | Bcen2424_0040 |
| BCAL3507 | 2287 | 1E-02 | BCAL3507 | flagellar FliL protein* | No homolog |
| BCAL3525 | 2823 | 5E-02 | BCAL3525 | type II secretion system protein F | Bcen2424_0060 |
| BCAM0821 | 4206 | 7E-02 | BCAM0821 | putative methyl-accepting chemotaxis protein | No homolog |
| BCAM0987 | 4637 | 2E-02 | BCAM0987 | flagellar hook protein 2 FlgE2 | Bcen2424_3954 |
| BCAM1503 | 3805 | 2E-02 | BCAM1503 | putative methyl-accepting chemotaxis protein* | Bcen2424_4375 |
| BCAM1804 | 3531 | 3E-02 | BCAM1804 | methyl-accepting chemotaxis protein* | Bcen2424_4665 |
| BCAM2760 | 2396 | 5E-02 | BCAM2760 | putative outer membrane usher* | No homolog |
| BCAS0104 | 2953 | 6E-01 | BCAS0104 | A-type flagellar hook-associated protein 2 (HAP2)* | No homolog |
| BCAS0631 | 444 | 1E-02 | BCAS0631 | putative CheB family methylesterase | No homolog |
| BCAS0632 | 7538 | 1E-02 | BCAS0632 | hybrid two-component system kinase-response regulator protein | No homolog |
|  |  |  |  | ***Intracellular trafficking, secretion, and vesicular transport (U)*** |  |
| BCAL0173 | 2483 | 9E-02 | BCAL0173 | putative plasmid conjugal transfer protein* | No homolog |
| BCAL0905 | 3456 | 1E-01 | BCAL0905 | hypothetical protein | No homolog |
| BCAL1522 | 8144 | 1E-02 | BCAL1522 | putative exported heme utilisation related protein* | Bcen2424_1514 |
| BCAL1526 | 1783 | 4E-02 | BCAL1526 | putative flp type pilus assembly protein | No homolog |
| BCAL2345 | 2187 | 1E-02 | BCAL2345 | protein-export membrane protein SecG | Bcen2424_2250 |
| BCAL2475 | 9182 | 2E-02 | BCAL2475 | hypothetical protein* | No homolog |
| BCAL3435 | 4539 | 2E-02 | BCAL3435 | MarC family integral membrane protein | Bcen2424_0588 |
| BCAS0547 | 4255 | 8E-02 | BCAS0547 | putative DNA-binding phage protein | No homolog |
| pBCA020 | 4378 | 3E-02 | pBCA020 | putative TraG conjugative transfer protein | No homolog |
| pBCA041 | 3353 | 9E-02 | pBCA041 | putative TraC conjugative transfer protein | No homolog |
| pBCA059 | 169 | 4E-02 | pBCA059 | putative TraD conjugative transfer protein | No homolog |
|  |  |  |  | ***Posttranslational modification, protein turnover, chaperones (O)*** |  |
| BCAL0111 | 1109 | 1E-02 | BCAL0111 | putative TPR repeat protein | No homolog |
| BCAL0268 | 2772 | 7E-02 | BCAL0268 | putative cytochrome c biogenesis protein | Bcen2424_0382 |
| BCAL0347 | 2019 | 2E-02 | BCAL0347 | putative type VI secretion system protein TssH | Bcen2424_0473 |
| BCAL0540 | 2131 | 9E-02 | BCAL0540 | putative ATPase | Bcen2424_3049 |
| BCAL0557 | 2687 | 1E-02 | BCAL0557 | putative glutathione S-transferase | Bcen2424_3032 |
| BCAL1250 | 2357 | 4E-02 | BCAL1250 | putative glutathione S-transferase | No homolog |
| BCAL1653 | 318 | 3E-02 | BCAL1653 | sulfate transport system permease protein | Bcen2424_1602 |
| BCAL2013 | 2354 | 1E-01 | BCAL2013 | AhpC/TSA family protein | Bcen2424_1940 |
| BCAL2162 | 4326 | 6E-01 | BCAL2162 | putative FkbP-type peptidyl-prolyl cis-trans isomerase | Bcen2424_2090 |
| BCAL2321 | 1105 | 4E-02 | BCAL2321 | putative glutathione S-transferase | Bcen2424_2226 |
| BCAL2323 | 2138 | 8E-02 | BCAL2323 | putative glutathione S-transferase | Bcen2424_2228 |
| BCAL2416 | 2855 | 4E-02 | BCAL2416 | putative lipoprotein* | Bcen2424_2320 |
| BCAL2539 | 3487 | 6E-02 | BCAL2539 | putative glutathione-S-transferase | No homolog |
| BCAL3269 | 3619 | 1E-02 | BCAL3269 | putative DnaJ chaperone protein | Bcen2424_0753 |
| BCAL3418 | 2109 | 1E-02 | BCAL3418 | putative thioredoxin protein* | No homolog |
| BCAM0278 | 4511 | 6E-01 | BCAM0278 | putative heat shock protein | No homolog |
| BCAM0524 | 2475 | 3E-02 | BCAM0524 | AAA family ATPase protein* | No homolog |
| BCAM1021 | 288 | 1E-02 | BCAM1021 | protein FdhE homologue | Bcen2424_3987 |
| BCAM1744 | 4481 | 1E-02 | BCAM1744 | serine peptidase, family S9* | No homolog |
| BCAM2753 | 2556 | 3E-02 | BCAM2753 | putative organic hydroperoxide resistance protein | No homolog |
| pBCA025 | 4464 | 3E-02 | pBCA025 | putative TraF conjugative transfer protein* | No homolog |
| pBCA043 | 29 | 2E-02 | pBCA043 | thiol:disulfide interchange protein DsbC precursor* | No homolog |
|  |  |  |  | ***Energy production and conversion (C)*** |  |
| BCAL0029 | 4549 | 4E-02 | BCAL0029 | putative ATP synthase protein I AtpI | Bcen2424_0099 |
| BCAL0034 | 2553 | 5E-02 | BCAL0034 | ATP synthase alpha chain | Bcen2424_0104 |
| BCAL0037 | 2854 | 3E-00 | BCAL0037 | ATP synthase epsilon chain | Bcen2424_0107 |
| BCAL0052 | 2239 | 1E-02 | BCAL0052 | putative oxidoreductase | Bcen2424_0121 |
| BCAL0164 | 2751 | 2E-02 | BCAL0164 | putative cytochrome c-551 precursor* | Bcen2424_0293 |
| BCAL0408 | 2762 | 1E-02 | BCAL0408 | putative phenylacetic acid degradation oxidoreductase | Bcen2424_0543 |
| BCAL0586 | 206 | 1E-02 | BCAL0586 | glutathione reductase | Bcen2424_3006 |
| BCAL0743 | 3177 | 2E-02 | BCAL0743 | putative glycerol-3-phosphate dehydrogenase (NAD(P)+) | No homolog |
| BCAL0754 | 2374 | 9E-02 | BCAL0754 | putative cytochrome c oxidase subunit III | Bcen2424_2842 |
| BCAL0775 | 202 | 4E-02 | BCAL0775 | putative molybdopterin oxidoreductase/DMSO reductase protein | No homolog |
| BCAL0850 | 1688 | 6E-02 | BCAL0850 | glycolate permease* | No homolog |
| BCAL0851 | 1935 | 1E-02 | BCAL0851 | putative iron-sulphur cluster containing protein | No homolog |
| BCAL0853 | 2104 | 4E-02 | BCAL0853 | hypothetical protein | No homolog |
| BCAL1051 | 5631 | 2E-02 | BCAL1051 | radical SAM superfamily protein | Bcen2424_1171 |
| BCAL1074 | 4966 | 6E-01 | BCAL1074 | isoquinoline 1-oxidoreductase alpha subunit | No homolog |
| BCAL1106 | 2425 | 3E-02 | BCAL1106 | cytochrome b561 family protein | No homolog |
| BCAL1153 | 2088 | 5E-02 | BCAL1153 | putative salicylaldehyde dehydrogenase* | No homolog |
| BCAL1183 | 8021 | 7E-02 | BCAL1183 | aldehyde dehydrogenase family protein | No homolog |
| BCAL1340 | 2356 | 6E-02 | BCAL1340 | putative maleylacetate reductase | No homolog |
| BCAL1517 | 2157 | 3E-02 | BCAL1517 | dihydrolipoamide dehydrogenase | Bcen2424_1510 |
| BCAL1769 | 2352 | 1E-02 | BCAL1769 | putative L(+)-mandelate dehydrogenase | No homolog |
| BCAL2305 | 3177 | 1E-02 | BCAL2305 | putative potassium channel subunit | Bcen2424_2213 |
| BCAL2331 | 2347 | 3E-02 | BCAL2331 | NADH dehydrogenase I chain N* | No homolog |
| BCAL2332 | 2335 | 2E-02 | BCAL2332 | NADH dehydrogenase I chain M | Bcen2424_2237 |
| BCAL2334 | 2908 | 1E-02 | BCAL2334 | NADH-ubiquinone oxidoreductase I chain K | Bcen2424_2239 |
| BCAL2335 | 358 | 2E-02 | BCAL2335 | NADH dehydrogenase I chain J | Bcen2424_2240 |
| BCAL2347 | 2081 | 2E-02 | BCAL2347 | putative oxidoreductase | Bcen2424_2252 |
| BCAL2485 | 1124 | 1E-02 | BCAL2485 | putative iron-sulphur cluster binding electron transport protein | No homolog |
| BCAL2486 | 9333 | 1E-03 | BCAL2486 | putative iron-sulphur oxidoreductase | No homolog |
| BCAL2487 | 5913 | 2E-02 | BCAL2487 | putative D-lactate dehydrogenase | No homolog |
| BCAL2587 | 2266 | 3E-02 | BCAL2587 | putative alcohol dehydrogenase* | No homolog |
| BCAL2745 | 2441 | 6E-02 | BCAL2745 | putative CoA transferase family protein | No homolog |
| BCAL2908 | 3116 | 7E-02 | BCAL2908 | fumarate hydratase class II | Bcen2424_1090 |
| BCAL2935 | 2267 | 4E-02 | BCAL2935 | electron transfer flavoprotein beta-subunit | Bcen2424_1062 |
| BCAL2976 | 3798 | 9E-02 | BCAL2976 | NAD-dependent formate dehydrogenase gamma subunit | Bcen2424_1033 |
| BCAL3312 | 4804 | 2E-02 | BCAL3312 | putative cytochrome b-561 membrane protein* | Bcen2424_0714 |
| BCAL3475 | 2806 | 3E-02 | BCAL3475 | putative molybdopterin-containing oxidoreductase | Bcen2424_0545 |
| BCAM0320 | 2517 | 7E-02 | BCAM0320 | putative cytochrome b561 | No homolog |
| BCAM0905 | 5351 | 4E-02 | BCAM0905 | putative NADH dehydrogenase | No homolog |
| BCAM0961 | 4549 | 5E-02 | BCAM0961 | aconitate hydratase | Bcen2424_3928 |
| BCAM0967 | 373 | 3E-02 | BCAM0967 | putative succinate dehydrogenase cytochrome b556 subunit | No homolog |
| BCAM0969 | 447 | 3E-02 | BCAM0969 | succinate dehydrogenase flavoprotein subunit | Bcen2424_3936 |
| BCAM0972 | 1629 | 5E-02 | BCAM0972 | citrate synthase | Bcen2424_3939 |
| BCAM1250 | 204 | 2E-02 | BCAM1250 | probable acetyl-CoA hydrolase/transferase* | Bcen2424_4126 |
| BCAM1540 | 1159 | 1E-03 | BCAM1540 | putative dehydrogenase, molybdopterin binding subunit* | Bcen2424_5194 |
| BCAM1542 | 9568 | 2E-02 | BCAM1542 | putative aldehyde dehydrogenase | Bcen2424_4409 |
| BCAM1648 | 276 | 5E-02 | BCAM1648 | putative monooxygenase | No homolog |
| BCAM1944 | 8351 | 2E-02 | BCAM1944 | pyruvate dehydrogenase E1 component 2 | No homolog |
| BCAM1954 | 214 | 1E-02 | BCAM1954 | sodium:dicarboxylate symporter* | No homolog |
| BCAM2098 | 1231 | 2E-02 | BCAM2098 | putative isoquinoline 1-oxidoreductase alpha subunit | No homolog |
| BCAM2108 | 2897 | 9E-02 | BCAM2108 | putative GroES binding Zinc alcohol dehydrogenase | No homolog |
| BCAM2132 | 2437 | 5E-02 | BCAM2132 | 2-aminomuconate 6-semialdehyde dehydrogenase | No homolog |
| BCAM2277 | 4292 | 6E-02 | BCAM2277 | putative FMN-dependent dehydrogenase | Bcen2424_5034 |
| BCAM2710 | 6261 | 2E-02 | BCAM2710 | putative acetyl-CoA synthetase | Bcen2424_5525 |
| BCAS0078 | 2595 | 2E-02 | BCAS0078 | putative dehydrogenase, zinc-containing | No homolog |
| BCAS0207 | 2263 | 7E-02 | BCAS0207 | hypothetical protein | Bcen2424_6017 |
| BCAS0396 | 3052 | 4E-02 | BCAS0396 | putative dehydrogenase | No homolog |
| BCAS0500 | 2606 | 1E-02 | BCAS0500 | putative aldo/keto reductase family protein | No homolog |
| BCAS0711 | 2405 | 4E-02 | BCAS0711 | pyruvate dehydrogenase E1 component 3 | No homolog |
|  |  |  |  | ***Carbohydrate transport and metabolism (G)*** |  |
| BCAL0294 | 2666 | 1E-02 | BCAL0294 | Major Facilitator Superfamily protein | Bcen2424_0407 |
| BCAL0782 | 2429 | 2E-02 | BCAL0782 | putative chitobiase* | Bcen2424_2820 |
| BCAL1035 | 2795 | 3E-02 | BCAL1035 | putative trehalose-phosphatase | Bcen2424_1157 |
| BCAL1111 | 255 | 1E-02 | BCAL1111 | Major Facilitator Superfamily protein* | Bcen2424_1229 |
| BCAL1148 | 4938 | 2E-02 | BCAL1148 | putative polysaccharide deacetylase | No homolog |
| BCAL1181 | 6929 | 9E-02 | BCAL1181 | putative glycerate kinase | No homolog |
| BCAL1188 | 8014 | 1E-02 | BCAL1188 | hypothetical protein | No homolog |
| BCAL1252 | 15 | 6E-02 | BCAL1252 | putative proline/betaine transporter | Bcen2424_1281 |
| BCAL1659 | 6417 | 2E-02 | BCAL1659 | putative ribose transport system, permease protein | No homolog |
| BCAL1806 | 3833 | 4E-02 | BCAL1806 | hypothetical protein | Bcen2424_1734 |
| BCAL1855 | 2119 | 2E-02 | BCAL1855 | putative fructokinase-like protein | Bcen2424_1781 |
| BCAL2074 | 2608 | 4E-02 | BCAL2074 | phosphoenolpyruvate synthase | Bcen2424_2002 |
| BCAL2251 | 2452 | 3E-02 | BCAL2251 | Major Facilitator Superfamily protein | Bcen2424_2177 |
| BCAL2418 | 583 | 1E-01 | BCAL2418 | hypothetical protein* | Bcen2424_2322 |
| BCAL2470 | 4507 | 4E-02 | BCAL2470 | Major Facilitator Superfamily protein | No homolog |
| BCAL2476a | 6845 | 1E-02 | BCAL2476c | putative lysozyme | No homolog |
| BCAL2545 | 2189 | 2E-02 | BCAL2545 | Major Facilitator Superfamily protein | No homolog |
| BCAL2947 | 3045 | 2E-02 | BCAL2947 | hypothetical protein | No homolog |
| BCAL3130 | 8908 | 5E-02 | BCAL3130 | ABC transporter ATP-binding protein | No homolog |
| BCAL3240 | 4324 | 4E-02 | BCAL3240 | putative capsular polysaccharide transporter ATP-binding protein | No homolog |
| BCAL3241 | 4092 | 1E-02 | BCAL3241 | putative capsular polysaccharide export protein, ABC transporter membrane protein | No homolog |
| BCAM0068 | 7206 | 4E-02 | BCAM0068 | Major Facilitator Superfamily protein | No homolog |
| BCAM0783 | 3382 | 4E-02 | BCAM0783 | Major Facilitator Superfamily protein | Bcen2424_3742 |
| BCAM1361 | 4893 | 1E-02 | BCAM1361 | sugar-binding periplasmic protein precursor* | No homolog |
| BCAM1741 | 2836 | 2E-02 | BCAM1741 | Major Facilitator Superfamily protein* | Bcen2424_4560 |
| BCAM1760 | 2791 | 3E-02 | BCAM1760 | putative multidrug resistance transporter protein | Bcen2424_4583 |
| BCAM2243 | 2211 | 2E-02 | BCAM2243 | hypothetical protein | Bcen2424_5011 |
| BCAM2464 | 297 | 3E-02 | BCAM2464 | Major Facilitator Superfamily protein | No homolog |
| BCAM2600 | 2822 | 4E-02 | BCAM2600 | putative glucose-6-phosphate 1-dehydrogenase | Bcen2424_5407 |
| BCAM2665 | 313 | 1E-02 | BCAM2665 | Major Facilitator Superfamily protein | No homolog |
| BCAM2825 | 3159 | 2E-02 | BCAM2825 | Major Facilitator Superfamily protein* | Bcen2424_5673 |
| BCAS0035 | 2404 | 8E-02 | BCAS0035 | putative proline/betaine transporter | Bcen2424_6159 |
| BCAS0108 | 2295 | 1E-02 | BCAS0108 | Major Facilitator Superfamily protein | No homolog |
| BCAS0122 | 2154 | 8E-02 | BCAS0122 | putative transporter protein | No homolog |
| BCAS0254 | 377 | 1E-02 | BCAS0254 | Major Facilitator Superfamily protein | No homolog |
| BCAS0325 | 2305 | 3E-02 | BCAS0325 | putative periplasmic solute-binding protein* | Bcen2424_3285 |
| BCAS0690 | 2605 | 3E-01 | BCAS0690 | Major Facilitator Superfamily protein* | No homolog |
| BCAS0699 | 2521 | 2E-01 | BCAS0699 | hypothetical protein* | No homolog |
| BCAS0701 | 235 | 2E-02 | BCAS0701 | hypothetical protein | No homolog |
| BCAS0706 | 537 | 1E-02 | BCAS0706 | Major Facilitator Superfamily protein | No homolog |
| pBCA053 | 3166 | 2E-02 | pBCA053 | putative extracellular solute-binding protein* | No homolog |
|  |  |  |  | ***Cell cycle control, cell division, chromosome partitioning (D)*** |  |
| BCAL0143 | 2584 | 1E-02 | BCAL0143 | putative flagellar biosynthesis protein | Bcen2424_0270 |
| BCAL2147 | 2012 | 2E-02 | BCAL2147 | tRNA(Ile)-lysidine synthase | Bcen2424_2075 |
| BCAL2178 | 2602 | 1E-02 | BCAL2178 | septum formation initiator | Bcen2424_2106 |
| BCAL2394 | 3236 | 2E-02 | BCAL2394 | Maf-like protein | No homolog |
| BCAL2417 | 297 | 9E-02 | BCAL2417 | putative DNA translocase* | No homolog |
| BCAL3063 | 2354 | 1E-03 | BCAL3063 | CrcB-family inner membrane protein | Bcen2424_0946 |
| BCAL3227 | 1103 | 5E-02 | BCAL3227 | hypothetical protein | No homolog |
| BCAL3458 | 2833 | 7E-02 | BCAL3458 | cell division protein FtsA | No homolog |
| BCAL3469 | 5609 | 1E-02 | BCAL3469 | cell division protein FtsL* | Bcen2424_0552 |
| BCAM0006 | 2666 | 3E-02 | BCAM0006 | hypothetical protein | Bcen2424_5697 |
| BCAM1041 | 3075 | 6E-02 | BCAM1041 | putative phage coiled coil domain protein | No homolog |
| BCAM1534 | 3517 | 1E-19 | BCAM1534 | hypothetical protein | No homolog |
|  |  |  |  | ***Amino acid transport and metabolism (E)*** |  |
| BCAL0049 | 3643 | 2E-01 | BCAL0049 | putative aminotransferase | Bcen2424_0119 |
| BCAL0051 | 313 | 1E-02 | BCAL0051 | periplasmic solute-binding protein* | Bcen2424_0120 |
| BCAL0217 | 7872 | 8E-02 | BCAL0217 | putative exported GDSL-like lipase/acylhydrolase* | No homolog |
| BCAL0280 | 2955 | 2E-02 | BCAL0280 | 3-dehydroquinate synthase | Bcen2424_0393 |
| BCAL0291 | 4188 | 2E-02 | BCAL0291 | sodium:amino acid symporter family protein | Bcen2424_0404 |
| BCAL0312 | 2591 | 9E-02 | BCAL0312 | histidinol dehydrogenase | No homolog |
| BCAL0318 | 2113 | 4E-02 | BCAL0318 | imidazole glycerol phosphate synthase subunit HisF | Bcen2424_0431 |
| BCAL0319 | 3104 | 2E-01 | BCAL0319 | phosphoribosyl-AMP cyclohydrolase | Bcen2424_0432 |
| BCAL0377 | 2546 | 7E-02 | BCAL0377 | metallo peptidase, subfamily M24B | Bcen2424_0514 |
| BCAL0385 | 215 | 6E-02 | BCAL0385 | putative amino acid permease | Bcen2424_0522 |
| BCAL0774 | 2389 | 2E-02 | BCAL0774 | putative ornithine cyclodeaminase | No homolog |
| BCAL0797 | 4115 | 2E-02 | BCAL0797 | putative histidinol-phosphate aminotransferase | Bcen2424_2805 |
| BCAL1127 | 1091 | 5E-02 | BCAL1127 | hypothetical protein | No homolog |
| BCAL1185 | 4131 | 1E-02 | BCAL1185 | putative oxidoreductase | No homolog |
| BCAL1218 | 4593 | 2E-01 | BCAL1218 | putative agmatinase | No homolog |
| BCAL1219 | 2161 | 6E-02 | BCAL1219 | putative acetyltransferase | No homolog |
| BCAL1467 | 3225 | 3E-02 | BCAL1467 | chorismate synthase | No homolog |
| BCAL1839 | 2032 | 4E-02 | BCAL1839 | putative asparagine synthase | No homolog |
| BCAL1925 | 236 | 3E-02 | BCAL1925 | threonine synthase | Bcen2424_1853 |
| BCAL1988 | 2613 | 4E-02 | BCAL1988 | putative D-amino acid dehydrogenase small subunit | No homolog |
| BCAL2146 | 2022 | 1E-02 | BCAL2146 | aspartokinase | Bcen2424_2074 |
| BCAL2280 | 2299 | 9E-02 | BCAL2280 | putative homoserine/homoserine lactone efflux protein* | No homolog |
| BCAL2317 | 2669 | 4E-02 | BCAL2317 | family M14 unassigned peptidase | Bcen2424_2222 |
| BCAL2354 | 7678 | 2E-02 | BCAL2354 | 2-isopropylmalate synthase | No homolog |
| BCAL2542 | 227 | 6E-02 | BCAL2542 | 3-isopropylmalate dehydratase large subunit 2 | No homolog |
| BCAL2659 | 251 | 3E-02 | BCAL2659 | putative cobalamin biosynthesis aminotransferase protein | Bcen2424_2449 |
| BCAL2672 | 2411 | 4E-02 | BCAL2672 | putative dihydroxy-acid dehydratase | Bcen2424_2462 |
| BCAL2682 | 2073 | 3E-02 | BCAL2682 | putative sulfate adenylyltransferase subunit 2 1 | Bcen2424_2472 |
| BCAL2683 | 6698 | 2E-26 | BCAL2683 | putative phosphoadenosine phosphosulfate reductase | Bcen2424_2473 |
| BCAL2687 | 4066 | 2E-02 | BCAL2687 | putative lipoprotein* | Bcen2424_2477 |
| BCAL2796 | 2481 | 6E-02 | BCAL2796 | putative benzoylformate decarboxylase | Bcen2424_2583 |
| BCAL2902 | 2697 | 8E-02 | BCAL2902 | putative branched-chain amino acid transport protein | Bcen2424_1096 |
| BCAL2942 | 3572 | 2E-02 | BCAL2942 | cysteine synthase | Bcen2424_1055 |
| BCAL2954 | 2437 | 4E-02 | BCAL2954 | P-protein (bifunctional includes: chorismate mutase and prephenate dehydratase) | Bcen2424_1043 |
| BCAL2993 | 3408 | 4E-02 | BCAL2993 | aminopeptidase N | No homolog |
| BCAL3056 | 2403 | 3E-02 | BCAL3056 | putative aminotransferase | No homolog |
| BCAL3102 | 3073 | 5E-02 | BCAL3102 | ABC transporter ATP-binding protein | No homolog |
| BCAL3197 | 1002 | 6E-02 | BCAL3197 | serine hydroxymethyltransferase | Bcen2424_0806 |
| BCAL3292 | 2789 | 7E-02 | BCAL3292 | putative pyrroline-5-carboxylate reductase | No homolog |
| BCAL3332 | 8418 | 7E-02 | BCAL3332 | aminopeptidase P | Bcen2424_0695 |
| BCAM0016 | 2332 | 1E-01 | BCAM0016 | tartrate dehydrogenase | Bcen2424_5707 |
| BCAM0072 | 2946 | 2E-02 | BCAM0072 | putative thiamine pyrophosphate enzyme | No homolog |
| BCAM0260 | 5841 | 6E-02 | BCAM0260 | putative amino acid transporter* | No homolog |
| BCAM0261 | 2008 | 2E-02 | BCAM0261 | putative branched-chain amino acid transporter | No homolog |
| BCAM0263 | 7009 | 5E-02 | BCAM0263 | ABC transporter ATP-binding protein | No homolog |
| BCAM0299 | 2275 | 2E-02 | BCAM0299 | putative zinc-binding alcoholdehydrogenase | No homolog |
| BCAM0387 | 2538 | 7E-02 | BCAM0387 | pyruvate dehydrogenase (cytochrome) | Bcen2424_3376 |
| BCAM0409 | 6506 | 3E-02 | BCAM0409 | hypothetical protein | No homolog |
| BCAM0410 | 555 | 1E-00 | BCAM0410 | putative ABC-type glycine betaine transport protein* | No homolog |
| BCAM0459 | 29 | 3E-02 | BCAM0459 | cysteine desulfurase | Bcen2424_3426 |
| BCAM0460 | 2129 | 2E-02 | BCAM0460 | putative amino acid ABC transporter ATP-binding protein | No homolog |
| BCAM0512 | 3449 | 4E-02 | BCAM0512 | putative aminotransferase | Bcen2424_3477 |
| BCAM0547 | 713 | 1E-02 | BCAM0547 | putative LysE type translocator | No homolog |
| BCAM0817 | 2707 | 1E-02 | BCAM0817 | putative acetolactate synthase | Bcen2424_3782 |
| BCAM0984 | 1982 | 2E-02 | BCAM0984 | 3-isopropylmalate dehydratase small subunit 1 | Bcen2424_3951 |
| BCAM0985 | 2022 | 1E-02 | BCAM0985 | 3-isopropylmalate dehydrogenase | Bcen2424_3952 |
| IG2_1360226 | 3373 | 5E-01 | BCAM1238 | transglutaminase-like protein | No homolog |
| BCAM1243 | 2991 | 3E-02 | BCAM1243 | putative aminotransferase | No homolog |
| BCAM1262 | 2339 | 3E-02 | BCAM1262 | dihydroxyacid dehydratase | No homolog |
| BCAM1293 | 2007 | 2E-02 | BCAM1293 | ABC transporter, substrate-binding protein* | No homolog |
| BCAM1311 | 1094 | 4E-02 | BCAM1311 | putative aliphatic amidase expression-regulating protein | No homolog |
| BCAM1416 | 1681 | 2E-01 | BCAM1416 | LysE-family transportery* | Bcen2424_4281 |
| BCAM1591 | 5254 | 6E-01 | BCAM1591 | putative pyridoxal-phosphate dependent enzyme | No homolog |
| BCAM1743 | 2171 | 3E-02 | BCAM1743 | periplasmic solute-binding protein* | Bcen2424_4562 |
| BCAM1798 | 2457 | 1E-01 | BCAM1798 | putative oxidoreductase | Bcen2424_4659 |
| BCAM2125 | 2477 | 5E-02 | BCAM2125 | indole-3-glycerol phosphate synthase | No homolog |
| BCAM2275 | 9847 | 2E-02 | BCAM2275 | putative DNA-binding protein | No homolog |
| BCAM2618 | 2191 | 1E-02 | BCAM2618 | putative periplasmic lysine-arginine-ornithine-binding protein* | No homolog |
| BCAM2671 | 4304 | 2E-02 | BCAM2671 | putative low-specificity L-threonine aldolase | Bcen2424_5475 |
| BCAM2747 | 1114 | 5E-02 | BCAM2747 | putative epimerase | No homolog |
| BCAM2776 | 2427 | 2E-02 | BCAM2776 | periplasmic solute-binding protein* | No homolog |
| BCAS0060 | 6786 | 1E-03 | BCAS0060 | extracellular amino acid-binding protein* | No homolog |
| BCAS0095 | 2995 | 6E-02 | BCAS0095 | putative sodium:solute symporter family protein | No homolog |
| BCAS0129 | 4485 | 1E-03 | BCAS0129 | putative binding-protein-dependent transport system component | Bcen2424_6101 |
| BCAS0132 | 3339 | 6E-02 | BCAS0132 | putative amidinohydrolase | No homolog |
| BCAS0140 | 2751 | 7E-02 | BCAS0140 | hypothetical protein* | Bcen2424_6092 |
| BCAS0145 | 3555 | 3E-02 | BCAS0145 | metallo peptidase, family M20 unassigned | Bcen2424_6087 |
| BCAS0211 | 1436 | 2E-02 | BCAS0211 | putative pyridoxal-dependent decarboxylase | Bcen2424_6013 |
| BCAS0375 | 3634 | 1E-02 | BCAS0375 | putative aspartate aminotransferase | No homolog |
| BCAS0409 | 4842 | 3E-02 | BCAS0409 | zinc metalloprotease ZmpA* | Bcen2424_6599 |
| BCAS0456 | 2077 | 5E-02 | BCAS0456 | hypothetical protein | No homolog |
| BCAS0702 | 4198 | 3E-02 | BCAS0702 | putative substrate-binding transporter protein | No homolog |
|  |  |  |  | ***Nucleotide transport and metabolism (F)*** |  |
| BCAL0012 | 2468 | 6E-02 | BCAL0012 | putative adenylate cyclase | Bcen2424_0080 |
| BCAL0437 | 2018 | 6E-02 | BCAL0437 | O6-methylguanine-DNA methyltransferase | Bcen2424_3152 |
| BCAL0871 | 2453 | 4E-02 | BCAL0871 | hypothetical protein | Bcen2424_2734 |
| BCAL1278 | 2842 | 2E-02 | BCAL1278 | putative exopolyphosphatase | No homolog |
| BCAL2038 | 4361 | 4E-02 | BCAL2038 | putative allantoicase | No homolog |
| BCAL2181 | 3023 | 7E-02 | BCAL2181 | CTP synthase | Bcen2424_2109 |
| BCAL2727 | 2888 | 7E-01 | BCAL2727 | deoxyuridine 5'-triphosphate nucleotidohydrolase | Bcen2424_2515 |
| BCAL2762 | 2289 | 4E-02 | BCAL2762 | putative adenylate kinase | Bcen2424_2548 |
| BCAL2989 | 3103 | 5E-02 | BCAL2989 | adenosine deaminase | Bcen2424_1020 |
| BCAL3015 | 2219 | 3E-02 | BCAL3015 | Ham1 family protein | Bcen2424_0996 |
| BCAL3172 | 2906 | 2E-02 | BCAL3172 | putative xanthine dehydrogenase large subunit | Bcen2424_0833 |
| BCAL3261 | 2582 | 1E-02 | BCAL3261 | putative phosphoribosylformylglycinamidine cyclo-ligase | No homolog |
| BCAL3361 | 3309 | 5E-02 | BCAL3361 | adenylosuccinate lyase | Bcen2424_0666 |
| BCAM0402 | 2624 | 2E-02 | BCAM0402 | guanine deaminase | No homolog |
| BCAM1457 | 4827 | 4E-02 | BCAM1457 | cytosine deaminase | Bcen2424_4328 |
| BCAS0337 | 2453 | 1E-02 | BCAS0337 | putative lipoprotein* | Bcen2424_6758 |
| pBCA088 | 3554 | 8E-02 | pBCA088 | amidohydrolase family protein | No homolog |
|  |  |  |  | ***Coenzyme transport and metabolism (H)*** |  |
| BCAL0710 | 277 | 2E-02 | BCAL0710 | lipoic acid synthetase | Bcen2424_2887 |
| BCAL1339 | 2853 | 1E-02 | BCAL1339 | aromatic hydrocarbon catabolic monooxygenase | No homolog |
| BCAL1705 | 5127 | 4E-02 | BCAL1705 | cobyrinic acid A,C-diamide synthase | Bcen2424_1651 |
| BCAL1711 | 4801 | 2E-02 | BCAL1711 | putative cobalamin biosynthesis-related protein | No homolog |
| BCAL1840 | 2052 | 3E-00 | BCAL1840 | hypothetical protein | Bcen2424_1767 |
| BCAL2628 | 2631 | 4E-02 | BCAL2628 | putative porphyrin biosynthesis related protein | No homolog |
| BCAL2632 | 2154 | 6E-02 | BCAL2632 | putative biotin biosyntehsis related protein | Bcen2424_2427 |
| BCAL2662 | 3564 | 3E-02 | BCAL2662 | putative cobalamin [5'-phosphate] synthase | Bcen2424_2452 |
| BCAL2717 | 2642 | 2E-02 | BCAL2717 | putative nicotinate-nucleotide pyrophosphorylase | No homolog |
| BCAL2955 | 2479 | 1E-02 | BCAL2955 | phosphoserine aminotransferase | Bcen2424_1042 |
| BCAL3145 | 3503 | 1E-02 | BCAL3145 | putative kinase | Bcen2424_0861 |
| BCAL3187 | 2761 | 2E-02 | BCAL3187 | putative oxidoreductase | Bcen2424_0817 |
| BCAL3218 | 345 | 2E-02 | BCAL3218 | putative acyl-CoA transferase | No homolog |
| BCAL3296 | 6753 | 1E-02 | BCAL3296 | putative 4-hydroxybenzoate transmembrane octaprenyltransferase | No homolog |
| BCAL3333 | 2927 | 4E-02 | BCAL3333 | putative ubiquinone biosynthesis-related protein | Bcen2424_0694 |
| BCAM0010 | 322 | 7E-01 | BCAM0010 | 2-amino-3-ketobutyrate coenzyme A ligase | Bcen2424_5701 |
| BCAM0077 | 2212 | 5E-02 | BCAM0077 | putative flavoprotein monooxygenase | Bcen2424_5774 |
| BCAM2080 | 2275 | 3E-02 | BCAM2080 | putative bifunctional NMN adenylyltransferase/NUDIX hydrolase | Bcen2424_4887 |
| BCAM2185 | 3101 | 1E-02 | BCAM2185 | putative methyltransferase | No homolog |
| BCAM2784 | 2496 | 6E-01 | BCAM2784 | putative aminotransferase | No homolog |
| BCAS0013 | 2882 | 8E-02 | BCAS0013 | putative molybdenum transport protein | Bcen2424_6181 |
| BCAS0206 | 8078 | 3E-02 | BCAS0206 | putative methyltransferase family protein | Bcen2424_6018 |
| BCAS0253 | 2378 | 6E-02 | BCAS0253 | putative ketopantoate reductase family protein | No homolog |
|  |  |  |  | ***Lipid transport and metabolism (I)*** |  |
| BCAL0038 | 3431 | 3E-02 | BCAL0038 | putative long-chain-fatty-acid--CoA ligase | No homolog |
| BCAL0391 | 2274 | 1E-02 | BCAL0391 | acyl carrier protein phosphodiesterase | Bcen2424_0528 |
| BCAL0882 | 3212 | 4E-02 | BCAL0882 | putative phospholipase D | Bcen2424_2723 |
| BCAL0886 | 2819 | 1E-02 | BCAL0886 | putative 3-ketoacyl-CoA thiolase | Bcen2424_2719 |
| BCAL1235 | 3403 | 3E-02 | BCAL1235 | putative 3-hydroxyacyl-CoA dehydrogenase | Bcen2424_1264 |
| BCAL1287 | 22 | 3E-02 | BCAL1287 | putative acetyl-CoA synthetase | Bcen2424_1316 |
| BCAL1473 | 4564 | 4E-02 | BCAL1473 | succinyl-CoA:3-ketoacid-coenzyme A transferase subunit B | No homolog |
| BCAL2322 | 2148 | 1E-02 | BCAL2322 | putative enoyl-CoA hydratase | Bcen2424_2227 |
| BCAL2326 | 2411 | 6E-02 | BCAL2326 | putative acyl-CoA dehydrogenase family protein | Bcen2424_2231 |
| BCAL2719 | 2025 | 8E-02 | BCAL2719 | putative transmembrane fatty acid desaturase | Bcen2424_2507 |
| BCAL2772 | 2085 | 4E-02 | BCAL2772 | putative AMP-binding enzyme | No homolog |
| BCAL2834 | 2874 | 2E-02 | BCAL2834 | putative acylhydrolase* | Bcen2424_2621 |
| BCAL2909 | 331 | 4E-02 | BCAL2909 | long-chain acyl-CoA thioester hydrolase | Bcen2424_1089 |
| BCAL2939 | 2298 | 2E-02 | BCAL2939 | putative exported monoglyceride lipase* | Bcen2424_1058 |
| BCAM0241 | 3209 | 1E-02 | BCAM0241 | putative 3-oxoacyl-[acyl-carrier-protein] synthase | No homolog |
| BCAM0242 | 2068 | 1E-01 | BCAM0242 | hypothetical protein* | No homolog |
| BCAM0244 | 2047 | 9E-02 | BCAM0244 | putative acyl-CoA dehydrogenase | No homolog |
| BCAM0252 | 4054 | 8E-02 | BCAM0252 | putative 3-oxoacyl-[acyl-carrier-protein] synthase* | No homolog |
| BCAM0253 | 2135 | 4E-02 | BCAM0253 | putative 3-oxoacyl-[acyl-carrier-protein] synthase | No homolog |
| BCAM0341 | 3443 | 4E-01 | BCAM0341 | FMN-dependent NADH-azoreductase | No homolog |
| BCAM1333 | 321 | 1E-02 | BCAM1333 | putative exopolysaccharide acyltransferase | No homolog |
| BCAM1459 | 6496 | 1E-02 | BCAM1459 | short-chain fatty acid transporter | No homolog |
| BCAM1595 | 7112 | 1E-01 | BCAM1595 | putative esterase | No homolog |
| BCAM2114 | 3612 | 1E-03 | BCAM2114 | putative hydroxylase | No homolog |
| BCAM2173 | 3761 | 4E-02 | BCAM2173 | FMN-dependent nadh-azoreductase* | No homolog |
| BCAM2372 | 2208 | 9E-02 | BCAM2372 | putative acetyl-coenzyme A synthetase | Bcen2424_5130 |
| BCAM2568 | 235 | 1E-02 | BCAM2568 | putative beta-ketoadipyl CoA thiolase | Bcen2424_5368 |
| BCAM2793 | 2897 | 4E-02 | BCAM2793 | putative feruloyl-CoA hydratase | Bcen2424_5636 |
| BCAS0175 | 494 | 2E-02 | BCAS0175 | putative hydrolase | No homolog |
| BCAS0208 | 156 | 4E-02 | BCAS0208 | putative acyl-CoA dehydrogenase | Bcen2424_6016 |
| BCAS0209 | 7775 | 1E-02 | BCAS0209 | hypothetical protein | Bcen2424_6015 |
| BCAS0210 | 1439 | 6E-02 | BCAS0210 | putative AMP-binding enzyme | Bcen2424_6014 |
| BCAS0212 | 1263 | 3E-02 | BCAS0212 | hypothetical protein | Bcen2424_6012 |
| BCAS0222 | 4478 | 2E-02 | BCAS0222 | putative AMP-dependent synthetase | Bcen2424_6002 |
| BCAS0223 | 7835 | 4E-02 | BCAS0223 | putative fatty acid desaturase | No homolog |
| BCAS0504 | 1178 | 2E-02 | BCAS0504 | putative phage transmembrane acetyltransferase | No homolog |
| pBCA036 | 2053 | 1E-02 | pBCA036 | hypothetical protein* | No homolog |
|  |  |  |  | ***Inorganic ion transport and metabolism (P)*** |  |
| BCAL0273 | 3809 | 5E-02 | BCAL0273 | protein CyaY | Bcen2424_0386 |
| BCAL0671 | 2074 | 4E-02 | BCAL0671 | putative carbonic anhydrase | Bcen2424_2927 |
| BCAL1270 | 2423 | 4E-02 | BCAL1270 | phosphate transport system, substrate-binding exported periplasmic protein* | Bcen2424_1300 |
| BCAL1271 | 7207 | 4E-02 | BCAL1271 | phosphate transport system permease protein | Bcen2424_1301 |
| BCAL1277 | 2947 | 5E-02 | BCAL1277 | polyphosphate kinase | Bcen2424_1307 |
| BCAL1654 | 3621 | 9E-02 | BCAL1654 | sulfate transport system permease protein | Bcen2424_1603 |
| BCAL1655 | 4623 | 2E-02 | BCAL1655 | sulfate ABC transporter ATP-binding protein | Bcen2424_1604 |
| BCAL2271 | 1396 | 1E-02 | BCAL2271 | putative toxic anion resistance protein | No homolog |
| BCAL2353 | 1851 | 5E-02 | BCAL2353 | putative sulfate transporter | No homolog |
| BCAL2643 | 2099 | 1E-02 | BCAL2643 | superoxide dismutase SodC* | Bcen2424_2434 |
| BCAL2681 | 2222 | 2E-02 | BCAL2681 | putative sulfate adenylyltransferase subunit 1 | Bcen2424_2471 |
| BCAL2685 | 1231 | 1E-03 | BCAL2685 | putative sulfite reductase | Bcen2424_2475 |
| BCAL2814 | 2457 | 1E-02 | BCAL2814 | ABC transporter ATP-binding protein | Bcen2424_2601 |
| BCAL2938 | 2109 | 2E-02 | BCAL2938 | ABC transporter ATP-binding protein | Bcen2424_1059 |
| BCAL2975 | 2486 | 6E-02 | BCAL2975 | possible regulatory protein | Bcen2424_1034 |
| BCAL3023 | 4641 | 2E-02 | BCAL3023 | putative chloride-channel protein | No homolog |
| BCAL3216 | 6085 | 2E-01 | BCAL3216 | adenylyl-sulfate kinase | No homolog |
| BCAL3297 | 1512 | 5E-02 | BCAL3297 | putative ferritin DPS-family DNA binding protein | Bcen2424_0728 |
| BCAL3299 | 4364 | 8E-02 | BCAL3299 | peroxidase/catalase KatB | Bcen2424_0726 |
| BCAM0827 | 3297 | 5E-02 | BCAM0827 | putative voltage gated chloride channel membrane protein | No homolog |
| BCAM0832 | 2279 | 6E-02 | BCAM0832 | putative Dyd-type peroxidase family protein | Bcen2424_3798 |
| BCAM1187 | 136 | 6E-01 | BCAM1187 | TonB-dependent siderophore receptor* | No homolog |
| BCAM1359 | 9113 | 2E-01 | BCAM1359 | putative efflux pump/antiporter | Bcen2424_4226 |
| BCAM1458 | 2902 | 4E-02 | BCAM1458 | putative voltage-gated chloride channel | Bcen2424_4329 |
| BCAM1620 | 2686 | 5E-01 | BCAM1620 | Rieske iron-sulphur domain protein | No homolog |
| BCAM2135 | 2422 | 2E-02 | BCAM2135 | Major Facilitator Superfamily protein | No homolog |
|  |  |  |  |  |  |
| BCAL0149 | 271 | 5E-02 | BCAL0149 | putative dienelactone hydrolase | Bcen2424_0276 |
| BCAL1970 | 2644 | 6E-02 | BCAL1970 | thioesterase superfamily protein | No homolog |
| BCAL2733 | 2095 | 1E-02 | BCAL2733 | putative multicopper oxidase* | Bcen2424_2521 |
| BCAL3183 | 5168 | 4E-00 | BCAL3183 | putative hydrolase | No homolog |
| BCAL3229 | 5323 | 2E-02 | BCAL3229 | hypothetical protein | No homolog |
| BCAM0067 | 3279 | 9E-02 | BCAM0067 | putative short chain dehydrogenase* | No homolog |
| BCAM0528 | 2952 | 2E-02 | BCAM0528 | putative oxidoreductase/short-chain dehydrogenase | Bcen2424_3502 |
| BCAM0568 | 2017 | 8E-02 | BCAM0568 | putative short chain dehydrogenase | Bcen2424_3542 |
| BCAM0804 | 2137 | 5E-01 | BCAM0804 | catechol 1,2-dioxygenase 1 | Bcen2424_3769 |
| BCAM0811 | 2077 | 4E-02 | BCAM0811 | putative aromatic oxygenase | Bcen2424_3776 |
| BCAM0894 | 2613 | 2E-02 | BCAM0894 | poly(3-hydroxyalkanoate) depolymerase C precursor | Bcen2424_3862 |
| BCAM1156 | 3532 | 8E-02 | BCAM1156 | putative isochorismatase | Bcen2424_4052 |
| BCAM1360 | 278 | 2E-02 | BCAM1360 | putative short-chain dehydrogenase/oxidoreductase | No homolog |
| BCAM1411 | 2103 | 7E-02 | BCAM1411 | putative short-chain dehydrogenase | Bcen2424_4276 |
| BCAM2123 | 2053 | 1E-02 | BCAM2123 | putative 4-oxalocrotonate decarboxylase | No homolog |
| BCAM2127 | 6328 | 7E-02 | BCAM2127 | putative dienelactone hydrolase family protein | No homolog |
| BCAM2178 | 2876 | 4E-02 | BCAM2178 | putative isochorismatase | No homolog |
| BCAM2283 | 2398 | 1E-00 | BCAM2283 | putative short chain dehydrogenase | Bcen2424_5040 |
| BCAM2707 | 2167 | 1E-00 | BCAM2707 | putative FAA-hydrolase family protein | Bcen2424_5521 |
| BCAM2754 | 2538 | 9E-02 | BCAM2754 | putative ketoreductase | No homolog |
| BCAM2775 | 218 | 3E-02 | BCAM2775 | putative dioxygenase | Bcen2424_5617 |
|  |  |  |  | ***General function prediction only (R)*** |  |
| BCAL0094 | 378 | 1E-02 | BCAL0094 | phage major tail tube protein | No homolog |
| BCAL0109 | 9669 | 5E-02 | BCAL0109 | putative TPR repeat protein | No homolog |
| BCAL0154 | 7984 | 4E-02 | BCAL0154 | histone-like nucleoid-structuring (H-NS) protein | Bcen2424_0281 |
| BCAL0176 | 7537 | 6E-02 | BCAL0176 | hypothetical protein | No homolog |
| BCAL0202 | 2055 | 1E-02 | BCAL0202 | putative flavoprotein | Bcen2424_0315 |
| BCAL0455 | 2245 | 5E-02 | BCAL0455 | putative hydrolase | No homolog |
| BCAL0673 | 2157 | 4E-02 | BCAL0673 | metallo-beta-lactamase superfamily protein | Bcen2424_2925 |
| BCAL0771 | 933 | 6E-02 | BCAL0771 | non-heme chloroperoxidase | No homolog |
| BCAL1031 | 5209 | 3E-02 | BCAL1031 | aetyltransferase (GNAT) family protein | Bcen2424_1153 |
| IG1_1237542 | 2513 | 5E-02 | BCAL1138 | hypothetical protein | No homolog |
| BCAL1139 | 5346 | 3E-02 | BCAL1139 | putative hydrolase | No homolog |
| BCAL1186 | 8366 | 2E-02 | BCAL1186 | putative oxidoreductase | No homolog |
| BCAL1297 | 169 | 3E-02 | BCAL1297 | hypothetical protein | No homolog |
| BCAL1380 | 2973 | 1E-03 | BCAL1380 | (R)-stereoselective amidase | No homolog |
| BCAL1465 | 2028 | 4E-02 | BCAL1465 | hypothetical protein | Bcen2424_1458 |
| BCAL1478 | 2077 | 2E-02 | BCAL1478 | putative hydrolase | Bcen2424_1471 |
| BCAL1518 | 2333 | 2E-02 | BCAL1518 | AFG1-like ATPase | Bcen2424_1511 |
| BCAL1703 | 2773 | 1E-02 | BCAL1703 | metallo peptidase, subfamily M20D | Bcen2424_1649 |
| BCAL1710 | 3943 | 3E-02 | BCAL1710 | putative cobalamin biosynthesis-related protein | Bcen2424_1656 |
| BCAL1716 | 2854 | 2E-02 | BCAL1716 | hypothetical protein* | No homolog |
| BCAL1759 | 2739 | 1E-02 | BCAL1759 | 2-nitropropane dioxygenase family protein* | No homolog |
| BCAL1846 | 3288 | 7E-02 | BCAL1846 | ABC transporter ATP-binding protein | No homolog |
| BCAL1886 | 3232 | 3E-02 | BCAL1886 | radical SAM superfamily protein | Bcen2424_1814 |
| BCAL1895 | 2145 | 9E-02 | BCAL1895 | putative acid phosphatase | No homolog |
| BCAL1986 | 2222 | 1E-01 | BCAL1986 | putative acetyltransferase | No homolog |
| BCAL2058 | 6867 | 6E-01 | BCAL2058 | acetyltransferase (GNAT) family protein | No homolog |
| BCAL2158 | 3273 | 4E-02 | BCAL2158 | FAD dependent oxidoreductase | Bcen2424_2086 |
| BCAL2165 | 2154 | 3E-02 | BCAL2165 | metallo-beta-lactamase superfamily protein | Bcen2424_2093 |
| BCAL2226 | 2549 | 7E-02 | BCAL2226 | putative molybdopterin-binding protein | Bcen2424_2151 |
| BCAL2413 | 2194 | 6E-02 | BCAL2413 | hypothetical protein* | Bcen2424_2317 |
| BCAL2466 | 2385 | 1E-02 | BCAL2466 | ecotin precursor | Bcen2424_2387 |
| BCAL2528 | 2878 | 5E-02 | BCAL2528 | putative hydrolase | No homolog |
| BCAL2530 | 4579 | 2E-01 | BCAL2530 | hypothetical protein | No homolog |
| BCAL2536a | 7061 | 9E-02 | BCAL2536A | putative hydrolase | No homolog |
| BCAL2541 | 7697 | 7E-02 | BCAL2541 | putative hydrolase | No homolog |
| BCAL2554 | 4654 | 3E-02 | BCAL2554 | hypothetical protein | No homolog |
| BCAL2578 | 9303 | 9E-02 | BCAL2578 | putative hydrolase | No homolog |
| BCAL2584 | 1121 | 1E-02 | BCAL2584 | metallo-beta-lactamase superfamily protein | No homolog |
| BCAL2585 | 7089 | 4E-02 | BCAL2585 | putative nitrilase | No homolog |
| BCAL2690 | 355 | 3E-02 | BCAL2690 | short chain dehydrogenase | No homolog |
| BCAL2788 | 2274 | 4E-02 | BCAL2788 | flavin reductase family protein | No homolog |
| BCAL2978 | 3244 | 5E-01 | BCAL2978 | NAD-dependent formate dehydrogenase alpha subunit | Bcen2424_1031 |
| BCAL3122 | 1638 | 2E-02 | BCAL3122 | glycosyltransferase | No homolog |
| BCAL3198 | 1802 | 8E-02 | BCAL3198 | putative short-chain dehydrogenase | No homolog |
| BCAL3215 | 2685 | 9E-02 | BCAL3215 | cysteine peptidase, family C44 | Bcen2424_0787 |
| BCAL3222 | 3231 | 3E-02 | BCAL3222 | putative capsular polysaccharide biosynthesis dehydrogenase/reductase protein | No homolog |
| BCAL3234 | 9326 | 1E-02 | BCAL3234 | glycosyltransferase | No homolog |
| BCAL3291 | 2575 | 9E-02 | BCAL3291 | hypothetical protein | Bcen2424_0731 |
| BCAM0013 | 257 | 7E-02 | BCAM0013 | putative acetyltransferase-GNAT family | No homolog |
| BCAM0150 | 6522 | 7E-02 | BCAM0150 | putative lipoprotein* | No homolog |
| BCAM0152 | 2745 | 9E-01 | BCAM0152 | putative lipoprotein | No homolog |
| BCAM0236 | 2682 | 4E-02 | BCAM0236 | putative NADPH-dependent FMN reductase | Bcen2424_3197 |
| BCAM0280 | 243 | 1E-02 | BCAM0280 | putative phospholipid-binding protein | No homolog |
| BCAM0286 | 3871 | 2E-00 | BCAM0286 | putative alcohol dehydrogenase | No homolog |
| BCAM0347 | 2656 | 4E-02 | BCAM0347 | putative PIN-domain protein | No homolog |
| IG2_990284 | 3505 | 7E-02 | BCAM0903 | lysine decarboxylase family protein | Bcen2424_3871 |
| BCAM0938 | 4699 | 2E-02 | BCAM0938 | acetyltransferase (GNAT) family protein | No homolog |
| BCAM1051 | 1107 | 4E-02 | BCAM1051 | putative phage death-on-curing protein | No homolog |
| BCAM1255 | 5046 | 4E-02 | BCAM1255 | hypothetical protein | No homolog |
| BCAM1307 | 2066 | 2E-02 | BCAM1307 | putative helicase | Bcen2424_4179 |
| BCAM1435 | 2489 | 5E-02 | BCAM1435 | putative hydrolase | Bcen2424_4307 |
| BCAM1506 | 2603 | 2E-02 | BCAM1506 | putative phospholipid-binding lipoprotein | No homolog |
| BCAM1766 | 5791 | 5E-02 | BCAM1766 | putative regulatory protein | No homolog |
| BCAM1782 | 2103 | 2E-02 | BCAM1782 | hypothetical protein | Bcen2424_4602 |
| BCAM1821 | 2918 | 2E-02 | BCAM1821 | putative tautomerase | No homolog |
| BCAM1918 | 2435 | 1E-02 | BCAM1918 | putative phage lysozyme* | No homolog |
| BCAM1965 | 2955 | 3E-02 | BCAM1965 | putative hydrolase | No homolog |
| BCAM2011 | 5169 | 1E-02 | BCAM2011 | hypothetical protein | No homolog |
| BCAM2099 | 2441 | 8E-01 | BCAM2099 | putative Pfp1 family protein | No homolog |
| BCAM2120 | 3548 | 3E-02 | BCAM2120 | metallo-beta-lactamase superfamily protein | No homolog |
| BCAM2128 | 3764 | 4E-02 | BCAM2128 | putative short chain dehydrogenase | No homolog |
| BCAM2129 | 3954 | 3E-02 | BCAM2129 | 2-amino-3-carboxymuconate 6-semialdehyde decarboxylase | No homolog |
| BCAM2133 | 2237 | 2E-02 | BCAM2133 | hypothetical protein | No homolog |
| BCAM2357 | 6962 | 1E-02 | BCAM2357 | putative alpha/beta hydrolase | Bcen2424_5114 |
| BCAM2358 | 2916 | 2E-02 | BCAM2358 | putative esterase* | No homolog |
| BCAM2492 | 3128 | 6E-02 | BCAM2492 | hypothetical protein | Bcen2424_5294 |
| BCAM2578 | 2025 | 2E-02 | BCAM2578 | putative short chain dehydrogenase | No homolog |
| BCAM2619 | 2331 | 6E-02 | BCAM2619 | succinylglutamate desuccinylase/aspartoacylase family protein | Bcen2424_5425 |
| BCAM2705 | 156 | 3E-02 | BCAM2705 | hypothetical protein | No homolog |
| BCAM2739 | 2644 | 7E-02 | BCAM2739 | MoaA/NifB/PqqE family protein | Bcen2424_5553 |
| BCAS0006 | 3243 | 3E-02 | BCAS0006 | hypothetical protein* | Bcen2424_6189 |
| BCAS0034 | 344 | 6E-01 | BCAS0034 | metallo-beta-lactamase superfamily protein | Bcen2424_6162 |
| BCAS0079 | 2588 | 2E-02 | BCAS0079 | non-heme chloroperoxidase | No homolog |
| BCAS0090 | 2452 | 3E-01 | BCAS0090 | flavoprotein | Bcen2424_6131 |
| BCAS0097 | 3157 | 7E-02 | BCAS0097 | putative cobalamin synthesis protein | No homolog |
| BCAS0109 | 2487 | 3E-02 | BCAS0109 | succinylglutamate desuccinylase/aspartoacylase family protein | No homolog |
| BCAS0152 | 274 | 1E-03 | BCAS0152 | putative hydrolase | Bcen2424_6081 |
| BCAS0172 | 2672 | 2E-02 | BCAS0172 | putative dehydrogenase | No homolog |
| BCAS0173 | 3813 | 8E-02 | BCAS0173 | putative tautomerase | No homolog |
| BCAS0179 | 2962 | 8E-01 | BCAS0179 | metallo-beta-lactamase superfamily protein | No homolog |
| BCAS0226 | 9753 | 5E-01 | BCAS0226 | putative hydrolase | No homolog |
| BCAS0267a | 2481 | 3E-02 | BCAS0267a | putative calcineurin-like phosphoesterase family protein | No homolog |
| BCAS0416 | 2787 | 1E-02 | BCAS0416 | putative oxidoreductase* | Bcen2424_6594 |
| BCAS0506 | 3736 | 2E-02 | BCAS0506 | putative phage tail protein gpI | No homolog |
| BCAS0507 | 2635 | 1E-02 | BCAS0507 | putative phage baseplate assembly protein gpJ | No homolog |
| BCAS0508 | 2228 | 4E-02 | BCAS0508 | putative phage baseplate protein gpW | No homolog |
| BCAS0509 | 2866 | 5E-02 | BCAS0509 | putative phage baseplate assembly protein gpV | No homolog |
| BCAS0512 | 3073 | 1E-00 | BCAS0512 | hypothetical protein | No homolog |
| BCAS0517 | 7605 | 5E-02 | BCAS0517 | putative phage tail tube protein | No homolog |
| BCAS0580 | 71 | 4E-02 | BCAS0580 | hypothetical protein | No homolog |
| BCAS0660A | 3003 | 4E-02 | BCAS0660A | putative H-NS family DNA-binding protein | No homolog |
| BCAS0666 | 7124 | 3E-01 | BCAS0666 | putative ankyrin-repeat exported protein* | No homolog |
| BCAS0689 | 168 | 2E-02 | BCAS0689 | metallo-beta-lactamase superfamily protein | No homolog |
| BCAS0691 | 1075 | 1E-02 | BCAS0691 | putative LrgA family membrane protein | No homolog |
| BCAS0695 | 4138 | 4E-02 | BCAS0695 | putative putative phospholipid-binding exported protein* | No homolog |
| BCAS0703 | 4529 | 4E-02 | BCAS0703 | putative short-chain dehydrogenase | No homolog |
| BCAS0713 | 2572 | 1E-02 | BCAS0713 | putative short-chain oxidoreductase | No homolog |
| BCAS0722 | 1462 | 6E-02 | BCAS0722 | putative patatin-like phospholipase | No homolog |
| pBCA051 | 2023 | 1E-02 | pBCA051 | LamB/YcsF family protein | No homolog |
| pBCA093 | 2876 | 1E-02 | pBCA093 | PIN domain protein | No homolog |
|  |  |  |  | ***Function unknown (S)*** |  |
| BCAL0002 | 6446 | 4E-02 | BCAL0002 | carboxylate-amine ligase YbdK | Bcen2424_0069 |
| BCAL0179 | 7739 | 8E-02 | BCAL0179 | hypothetical protein | No homolog |
| BCAL0270 | 2434 | 7E-02 | BCAL0270 | ferric reductase-like transmembrane component | Bcen2424_0384 |
| BCAL0321 | 4888 | 1E-02 | BCAL0321 | hypothetical protein | No homolog |
| BCAL0337 | 3891 | 3E-02 | BCAL0337 | putative type VI secretion system protein TssL | Bcen2424_0463 |
| BCAL0360 | 3091 | 5E-02 | BCAL0360 | hypothetical protein | No homolog |
| BCAL0641A | 3134 | 9E-02 | BCAL0641A | hypothetical protein | No homolog |
| BCAL0728 | 3025 | 2E-02 | BCAL0728 | hypothetical protein | Bcen2424_2867 |
| BCAL0852 | 1263 | 4E-02 | BCAL0852 | hypothetical protein* | No homolog |
| BCAL1033 | 2002 | 9E-02 | BCAL1033 | hypothetical protein* | Bcen2424_1155 |
| BCAL1072 | 2119 | 8E-02 | BCAL1072 | hypothetical protein | No homolog |
| BCAL1165 | 4241 | 6E-02 | BCAL1165 | hypothetical protein | No homolog |
| BCAL1168 | 1241 | 3E-02 | BCAL1168 | hypothetical protein | No homolog |
| BCAL1172 | 3763 | 4E-02 | BCAL1172 | hypothetical protein | No homolog |
| BCAL1177 | 8514 | 1E-03 | BCAL1177 | putative fusaric acid resistance transporter protein | No homolog |
| BCAL1294 | 1286 | 2E-02 | BCAL1294 | hypothetical protein | No homolog |
| BCAL1295 | 2753 | 9E-02 | BCAL1295 | hypothetical protein | No homolog |
| BCAL1354 | 2278 | 3E-02 | BCAL1354 | hypothetical protein | No homolog |
| BCAL1355 | 1386 | 1E-03 | BCAL1355 | hypothetical protein | No homolog |
| BCAL1359 | 5753 | 1E-02 | BCAL1359 | hypothetical protein | No homolog |
| BCAL1362 | 7895 | 7E-02 | BCAL1362 | hypothetical protein | No homolog |
| BCAL1364 | 1998 | 7E-02 | BCAL1364 | hypothetical protein | No homolog |
| BCAL1401 | 266 | 1E-02 | BCAL1401 | hypothetical protein | No homolog |
| BCAL1455 | 322 | 2E-02 | BCAL1455 | putative fusaric acid resistance transporter protein* | Bcen2424_1448 |
| BCAL1520 | 2353 | 1E-02 | BCAL1520 | putative lipoprotein* | Bcen2424_1513 |
| BCAL1565 | 3835 | 4E-02 | BCAL1565 | hypothetical protein | No homolog |
| BCAL1604 | 334 | 9E-02 | BCAL1604 | hypothetical protein | No homolog |
| BCAL1606 | 4587 | 1E-02 | BCAL1606 | hypothetical protein | No homolog |
| BCAL1760 | 2918 | 2E-01 | BCAL1760 | hypothetical protein | Bcen2424_1697 |
| BCAL1881 | 5076 | 3E-02 | BCAL1881 | putative lipoprotein* | Bcen2424_1809 |
| BCAL1952 | 5812 | 2E-02 | BCAL1952 | hypothetical protein | Bcen2424_1881 |
| BCAL1967 | 4493 | 4E-02 | BCAL1967 | hypothetical protein | Bcen2424_1896 |
| BCAL2269 | 6025 | 9E-02 | BCAL2269 | hypothetical protein | No homolog |
| BCAL2401 | 3225 | 3E-02 | BCAL2401 | hypothetical protein* | No homolog |
| BCAL2493 | 3388 | 1E-02 | BCAL2493 | hypothetical protein | No homolog |
| BCAL2495 | 9809 | 2E-02 | BCAL2495 | hypothetical protein | No homolog |
| BCAL2502 | 2115 | 5E-02 | BCAL2502 | hypothetical protein | No homolog |
| BCAL2634a | 7194 | 1E-02 | BCAL2634a | hypothetical protein* | No homolog |
| BCAL2635 | 1653 | 6E-02 | BCAL2635 | hypothetical protein* | No homolog |
| BCAL2684 | 7155 | 2E-02 | BCAL2684 | hypothetical protein | Bcen2424_2474 |
| BCAL3062 | 3643 | 2E-01 | BCAL3062 | hypothetical protein | No homolog |
| BCAL3089 | 2099 | 2E-02 | BCAL3089 | hypothetical protein | No homolog |
| BCAL3153 | 4451 | 8E-01 | BCAL3153 | putative lipoprotein* | Bcen2424_0853 |
| BCAL3214 | 2008 | 3E-02 | BCAL3214 | carboxymuconolactone decarboxylase family protein | Bcen2424_0788 |
| BCAL3255 | 1059 | 4E-02 | BCAL3255 | putative DedA family protein | No homolog |
| BCAL3260 | 2487 | 3E-02 | BCAL3260 | hypothetical protein | No homolog |
| BCAL3279 | 3173 | 1E-02 | BCAL3279 | hypothetical protein | Bcen2424_0741 |
| BCAL3284 | 2327 | 9E-02 | BCAL3284 | hypothetical protein | No homolog |
| BCAL3311 | 2149 | 1E-02 | BCAL3311 | hypothetical protein* | Bcen2424_0715 |
| BCAL3319 | 8889 | 3E-02 | BCAL3319 | hypothetical protein | No homolog |
| BCAL3340 | 1633 | 6E-02 | BCAL3340 | hypothetical protein | No homolog |
| BCAL3489 | 2323 | 3E-02 | BCAL3489 | hypothetical protein | Bcen2424_0023 |
| BCAM0015 | 2223 | 2E-02 | BCAM0015 | hypothetical protein | Bcen2424_5705 |
| BCAM0066 | 115 | 6E-01 | BCAM0066 | putative lipoprotein | No homolog |
| BCAM0073 | 3084 | 6E-01 | BCAM0073 | hypothetical protein | No homolog |
| BCAM0148 | 4553 | 2E-02 | BCAM0148 | hypothetical protein | No homolog |
| BCAM0268 | 4778 | 9E-02 | BCAM0268 | hypothetical protein | No homolog |
| BCAM0271 | 5425 | 1E-16 | BCAM0271 | hypothetical protein | Bcen2424_3304 |
| BCAM0614 | 2278 | 2E-02 | BCAM0614 | DinB family protein* | No homolog |
| BCAM0846 | 2462 | 1E-02 | BCAM0846 | hypothetical protein | Bcen2424_3811 |
| BCAM0888 | 2593 | 4E-02 | BCAM0888 | hypothetical protein | Bcen2424_3856 |
| BCAM1027 | 1511 | 2E-02 | BCAM1027 | hypothetical protein | No homolog |
| BCAM1032 | 4179 | 6E-02 | BCAM1032 | putative phage DNA-binding protein | No homolog |
| BCAM1063 | 2038 | 2E-02 | BCAM1063 | hypothetical protein | No homolog |
| BCAM1066 | 2402 | 1E-01 | BCAM1066 | hypothetical protein | No homolog |
| BCAM1068 | 209 | 9E-02 | BCAM1068 | putative exported phage protein | No homolog |
| BCAM1193 | 8004 | 4E-01 | BCAM1193 | SpoVT/AbrB family regulatory protein | No homolog |
| BCAM1508 | 2004 | 2E-02 | BCAM1508 | hypothetical protein* | No homolog |
| BCAM1659 | 2057 | 8E-02 | BCAM1659 | hypothetical protein* | Bcen2424_4477 |
| BCAM1818 | 2039 | 2E-02 | BCAM1818 | hypothetical protein | Bcen2424_4678 |
| BCAM1883 | 4738 | 9E-02 | BCAM1883 | hypothetical protein | No homolog |
| BCAM1962A | 329 | 6E-02 | BCAM1962A | hypothetical protein | No homolog |
| BCAM2069 | 3059 | 7E-02 | BCAM2069 | hypothetical protein* | Bcen2424_4868 |
| BCAM2070 | 2415 | 1E-02 | BCAM2070 | hypothetical protein | Bcen2424_4875 |
| IG2_2305723 | 6663 | 1E-01 | BCAM2070 | hypothetical protein | Bcen2424_4875 |
| BCAM2351 | 2385 | 4E-02 | BCAM2351 | putative transmembrane component of ABC transporter | No homolog |
| BCAM2445 | 2836 | 5E-02 | BCAM2445 | hypothetical protein* | No homolog |
| BCAM2490 | 2091 | 8E-02 | BCAM2490 | hypothetical protein | No homolog |
| BCAM2517 | 5033 | 4E-02 | BCAM2517 | hypothetical protein* | No homolog |
| BCAM2682 | 1483 | 8E-01 | BCAM2682 | putative HicB family protein | No homolog |
| BCAM2700 | 3917 | 2E-02 | BCAM2700 | hypothetical protein | Bcen2424_5514 |
| BCAS0093 | 6187 | 6E-01 | BCAS0093 | putative Fic family protein | Bcen2424_6128 |
| BCAS0098 | 8709 | 7E-02 | BCAS0098 | hypothetical protein* | No homolog |
| BCAS0106 | 6235 | 1E-02 | BCAS0106 | hypothetical protein | No homolog |
| BCAS0125 | 1722 | 2E-02 | BCAS0125 | hypothetical protein | No homolog |
| BCAS0146 | 2598 | 2E-02 | BCAS0146 | putative MlrC family protein | Bcen2424_6086 |
| BCAS0170 | 2157 | 1E-02 | BCAS0170 | hypothetical protein | No homolog |
| BCAS0238A | 2254 | 2E-02 | BCAS0238A | hypothetical protein | No homolog |
| BCAS0250 | 2741 | 1E-02 | BCAS0250 | hypothetical protein | No homolog |
| BCAS0513 | 2458 | 3E-01 | BCAS0513 | putative phage tail protein | No homolog |
| BCAS0521 | 8135 | 2E-02 | BCAS0521 | hypothetical protein | No homolog |
| BCAS0527 | 2707 | 1E-02 | BCAS0527 | hypothetical protein | No homolog |
| BCAS0664 | 2375 | 2E-02 | BCAS0664 | hypothetical protein | No homolog |
| BCAS0667 | 2001 | 9E-02 | BCAS0667 | hypothetical protein | No homolog |
| BCAS0668 | 3682 | 6E-02 | BCAS0668 | hypothetical protein | No homolog |
| BCAS0693 | 2775 | 5E-00 | BCAS0693 | putative monooxygenase | No homolog |
| BCAS0694 | 7967 | 6E-02 | BCAS0694 | putative carboxymuconolactone decarboxylase family protein | No homolog |
| BCAS0723 | 4978 | 1E-00 | BCAS0723 | hypothetical protein | No homolog |
| BCAS0747 | 3504 | 5E-02 | BCAS0747 | hypothetical protein | No homolog |
| pBCA022 | 3566 | 2E-02 | pBCA022 | hypothetical protein | No homolog |
|  |  |  |  | ***Intergenic regions*** |  |
| IG1_3125198 | 1815 | 1E-02 | Multiple hits | No gene annotation | No homolog |
| IG2_2129074 | 2344 | 4E-02 | Multiple hits | No gene annotation | No homolog |
| IG1_1031094 | 2395 | 4E-02 | Intergenic region, chromosome 1 | No gene annotation | No homolog |
| IG1_106611 | 6976 | 5E-01 | Intergenic region, chromosome 1 | No gene annotation | No homolog |
| IG1_1078958 | 3001 | 3E-02 | Intergenic region, chromosome 1 | No gene annotation | No homolog |
| IG1_1089442 | 2549 | 3E-02 | Intergenic region, chromosome 1 | No gene annotation | No homolog |
| IG1_1108288 | 4773 | 6E-02 | Intergenic region, chromosome 1 | No gene annotation | No homolog |
| IG1_1120751 | 5918 | 3E-01 | Intergenic region, chromosome 1 | No gene annotation | No homolog |
| IG1_1222745 | 2696 | 2E-02 | Intergenic region, chromosome 1 | No gene annotation | No homolog |
| IG1_1224302 | 104 | 2E-02 | Intergenic region, chromosome 1 | No gene annotation | No homolog |
| IG1_1241336 | 4888 | 2E-02 | Intergenic region, chromosome 1 | No gene annotation | No homolog |
| IG1_1250607 | 5875 | 5E-02 | Intergenic region, chromosome 1 | No gene annotation | No homolog |
| IG1_1269507 | 2328 | 5E-02 | Intergenic region, chromosome 1 | No gene annotation | No homolog |
| IG1_1280464 | 3451 | 4E-02 | Intergenic region, chromosome 1 | No gene annotation | No homolog |
| IG1_1300172 | 839 | 1E-02 | Intergenic region, chromosome 1 | No gene annotation | No homolog |
| IG1_1320299 | 387 | 7E-02 | Intergenic region, chromosome 1 | No gene annotation | No homolog |
| IG1_1360268 | 3167 | 2E-01 | Intergenic region, chromosome 1 | No gene annotation | No homolog |
| IG1_137022 | 2298 | 2E-02 | Intergenic region, chromosome 1 | No gene annotation | No homolog |
| IG1_1381576 | 4028 | 2E-02 | Intergenic region, chromosome 1 | No gene annotation | No homolog |
| IG1_1402903 | 6247 | 8E-02 | Intergenic region, chromosome 1 | No gene annotation | No homolog |
| IG1_1411086 | 1077 | 2E-02 | Intergenic region, chromosome 1 | No gene annotation | No homolog |
| IG1_1413944 | 6238 | 3E-02 | Intergenic region, chromosome 1 | No gene annotation | No homolog |
| IG1_1428495 | 2277 | 6E-02 | Intergenic region, chromosome 1 | No gene annotation | No homolog |
| IG1_1459535 | 2829 | 2E-02 | Intergenic region, chromosome 1 | No gene annotation | No homolog |
| IG1_147730 | 2669 | 6E-02 | Intergenic region, chromosome 1 | No gene annotation | No homolog |
| IG1_1488167 | 7474 | 2E-02 | Intergenic region, chromosome 1 | No gene annotation | No homolog |
| IG1_1495019 | 1303 | 3E-02 | Intergenic region, chromosome 1 | No gene annotation | No homolog |
| IG1_1622885 | 2246 | 2E-02 | Intergenic region, chromosome 1 | No gene annotation | No homolog |
| IG1_1650963 | 2588 | 1E-02 | Intergenic region, chromosome 1 | No gene annotation | No homolog |
| IG1_1653344 | 2798 | 2E-02 | Intergenic region, chromosome 1 | No gene annotation | No homolog |
| IG1_1669072 | 1025 | 9E-02 | Intergenic region, chromosome 1 | No gene annotation | No homolog |
| IG1_1711150 | 2807 | 3E-02 | Intergenic region, chromosome 1 | No gene annotation | No homolog |
| IG1_175527 | 2579 | 5E-02 | Intergenic region, chromosome 1 | No gene annotation | No homolog |
| IG1_1780440 | 4487 | 1E-02 | Intergenic region, chromosome 1 | No gene annotation | No homolog |
| IG1_1780908 | 5653 | 5E-02 | Intergenic region, chromosome 1 | No gene annotation | No homolog |
| IG1_1781945 | 3219 | 8E-02 | Intergenic region, chromosome 1 | No gene annotation | No homolog |
| IG1_1797530 | 2093 | 3E-02 | Intergenic region, chromosome 1 | No gene annotation | No homolog |
| IG1_1803822 | 268 | 7E-01 | Intergenic region, chromosome 1 | No gene annotation | No homolog |
| IG1_181371 | 3993 | 2E-02 | Intergenic region, chromosome 1 | No gene annotation | No homolog |
| IG1_1818831 | 4043 | 2E-02 | Intergenic region, chromosome 1 | No gene annotation | No homolog |
| IG1_1876828 | 9064 | 8E-02 | Intergenic region, chromosome 1 | No gene annotation | No homolog |
| IG1_1891301 | 2026 | 8E-02 | Intergenic region, chromosome 1 | No gene annotation | No homolog |
| IG1_193540 | 2158 | 2E-01 | Intergenic region, chromosome 1 | No gene annotation | No homolog |
| IG1_1942024 | 2199 | 2E-02 | Intergenic region, chromosome 1 | No gene annotation | No homolog |
| IG1_196793 | 3349 | 5E-02 | Intergenic region, chromosome 1 | No gene annotation | No homolog |
| IG1_2049629 | 5279 | 1E-03 | Intergenic region, chromosome 1 | No gene annotation | No homolog |
| IG1_2102819 | 2811 | 3E-02 | Intergenic region, chromosome 1 | No gene annotation | No homolog |
| IG1_2152204 | 2117 | 4E-02 | Intergenic region, chromosome 1 | No gene annotation | No homolog |
| IG1_2166257 | 5983 | 2E-02 | Intergenic region, chromosome 1 | No gene annotation | No homolog |
| IG1_2196190 | 3516 | 5E-02 | Intergenic region, chromosome 1 | No gene annotation | No homolog |
| IG1_2266261 | 4757 | 9E-02 | Intergenic region, chromosome 1 | No gene annotation | No homolog |
| IG1_2266807 | 216 | 3E-02 | Intergenic region, chromosome 1 | No gene annotation | Bcen2424_1982 |
| IG1_2305438 | 2528 | 2E-02 | Intergenic region, chromosome 1 | No gene annotation | No homolog |
| IG1_2384865 | 6274 | 7E-02 | Intergenic region, chromosome 1 | No gene annotation | No homolog |
| IG1_2402666 | 5272 | 2E-02 | Intergenic region, chromosome 1 | No gene annotation | No homolog |
| IG1_2431502 | 2049 | 8E-02 | Intergenic region, chromosome 1 | No gene annotation | No homolog |
| IG1_245729 | 4126 | 2E-02 | Intergenic region, chromosome 1 | No gene annotation | No homolog |
| IG1_247826 | 3025 | 9E-02 | Intergenic region, chromosome 1 | No gene annotation | No homolog |
| IG1_257623 | 1313 | 1E-02 | Intergenic region, chromosome 1 | No gene annotation | No homolog |
| IG1_2686150 | 4859 | 4E-01 | Intergenic region, chromosome 1 | No gene annotation | No homolog |
| IG1_2714718 | 9953 | 2E-02 | Intergenic region, chromosome 1 | No gene annotation | No homolog |
| IG1_2737984 | 5717 | 1E-03 | Intergenic region, chromosome 1 | No gene annotation | No homolog |
| IG1_2758916 | 7393 | 2E-02 | Intergenic region, chromosome 1 | No gene annotation | No homolog |
| IG1_2817503 | 8218 | 7E-02 | Intergenic region, chromosome 1 | No gene annotation | No homolog |
| IG1_2828670 | 3694 | 9E-02 | Intergenic region, chromosome 1 | No gene annotation | No homolog |
| IG1_2839754 | 1084 | 3E-02 | Intergenic region, chromosome 1 | No gene annotation | No homolog |
| IG1_2843061 | 1165 | 7E-02 | Intergenic region, chromosome 1 | No gene annotation | No homolog |
| IG1_288839 | 2113 | 9E-02 | Intergenic region, chromosome 1 | No gene annotation | No homolog |
| IG1_2896142 | 2026 | 3E-02 | Intergenic region, chromosome 1 | No gene annotation | No homolog |
| IG1_2912072 | 7624 | 3E-02 | Intergenic region, chromosome 1 | No gene annotation | No homolog |
| IG1_2921887 | 5283 | 7E-02 | Intergenic region, chromosome 1 | No gene annotation | No homolog |
| IG1_2941513 | 2566 | 3E-02 | Intergenic region, chromosome 1 | No gene annotation | No homolog |
| IG1_2956285 | 2115 | 4E-02 | Intergenic region, chromosome 1 | No gene annotation | No homolog |
| IG1_3129514 | 2136 | 7E-02 | Intergenic region, chromosome 1 | No gene annotation | No homolog |
| IG1_3138657 | 9259 | 1E-02 | Intergenic region, chromosome 1 | No gene annotation | No homolog |
| IG1_3139352 | 572 | 1E-02 | Intergenic region, chromosome 1 | No gene annotation | No homolog |
| IG1_3170819 | 3555 | 2E-00 | Intergenic region, chromosome 1 | No gene annotation | No homolog |
| IG1_3180283 | 225 | 8E-02 | Intergenic region, chromosome 1 | No gene annotation | No homolog |
| IG1_3180461 | 7252 | 2E-02 | Intergenic region, chromosome 1 | No gene annotation | No homolog |
| IG1_3180610 | 2187 | 1E-02 | Intergenic region, chromosome 1 | No gene annotation | No homolog |
| IG1_3241588 | 4626 | 3E-02 | Intergenic region, chromosome 1 | No gene annotation | No homolog |
| IG1_3242996 | 9669 | 3E-02 | Intergenic region, chromosome 1 | No gene annotation | No homolog |
| IG1_3246787 | 8502 | 9E-02 | Intergenic region, chromosome 1 | No gene annotation | No homolog |
| IG1_3255066 | 9136 | 3E-02 | Intergenic region, chromosome 1 | No gene annotation | No homolog |
| IG1_3308709 | 2863 | 7E-02 | Intergenic region, chromosome 1 | No gene annotation | No homolog |
| IG1_3360123 | 3355 | 3E-02 | Intergenic region, chromosome 1 | No gene annotation | No homolog |
| IG1_3368693 | 4586 | 2E-22 | Intergenic region, chromosome 1 | No gene annotation | No homolog |
| IG1_3379970 | 4247 | 1E-02 | Intergenic region, chromosome 1 | No gene annotation | No homolog |
| IG1_3476475 | 5209 | 1E-02 | Intergenic region, chromosome 1 | No gene annotation | No homolog |
| IG1_3492560 | 2704 | 2E-02 | Intergenic region, chromosome 1 | No gene annotation | No homolog |
| IG1_352121 | 8697 | 2E-02 | Intergenic region, chromosome 1 | No gene annotation | No homolog |
| IG1_3535047 | 1896 | 3E-01 | Intergenic region, chromosome 1 | No gene annotation | No homolog |
| IG1_3537906 | 6609 | 1E-02 | Intergenic region, chromosome 1 | No gene annotation | No homolog |
| IG1_3541037 | 2986 | 1E-02 | Intergenic region, chromosome 1 | No gene annotation | No homolog |
| IG1_3543041 | 6716 | 3E-02 | Intergenic region, chromosome 1 | No gene annotation | No homolog |
| IG1_3554535 | 8098 | 1E-02 | Intergenic region, chromosome 1 | No gene annotation | No homolog |
| IG1_3556424 | 1006 | 6E-02 | Intergenic region, chromosome 1 | No gene annotation | No homolog |
| IG1_3598341 | 3617 | 5E-02 | Intergenic region, chromosome 1 | No gene annotation | No homolog |
| IG1_360976 | 218 | 5E-02 | Intergenic region, chromosome 1 | No gene annotation | No homolog |
| IG1_3634213 | 3663 | 1E-02 | Intergenic region, chromosome 1 | No gene annotation | No homolog |
| IG1_3664357 | 3695 | 3E-02 | Intergenic region, chromosome 1 | No gene annotation | No homolog |
| IG1_3745235 | 3553 | 6E-02 | Intergenic region, chromosome 1 | No gene annotation | No homolog |
| IG1_37663 | 4001 | 1E-02 | Intergenic region, chromosome 1 | No gene annotation | No homolog |
| IG1_3770932 | 2238 | 1E-02 | Intergenic region, chromosome 1 | No gene annotation | No homolog |
| IG1_3822505 | 2528 | 3E-02 | Intergenic region, chromosome 1 | No gene annotation | No homolog |
| IG1_392074 | 2203 | 4E-02 | Intergenic region, chromosome 1 | No gene annotation | No homolog |
| IG1_410649 | 6069 | 4E-02 | Intergenic region, chromosome 1 | No gene annotation | No homolog |
| IG1_446055 | 2584 | 1E-02 | Intergenic region, chromosome 1 | No gene annotation | No homolog |
| IG1_453676 | 6626 | 3E-02 | Intergenic region, chromosome 1 | No gene annotation | No homolog |
| IG1_471918 | 4945 | 2E-02 | Intergenic region, chromosome 1 | No gene annotation | No homolog |
| IG1_525485 | 2478 | 2E-02 | Intergenic region, chromosome 1 | No gene annotation | No homolog |
| IG1_563415 | 2895 | 3E-02 | Intergenic region, chromosome 1 | No gene annotation | No homolog |
| IG1_582769 | 225 | 1E-02 | Intergenic region, chromosome 1 | No gene annotation | No homolog |
| IG1_59230 | 1127 | 2E-02 | Intergenic region, chromosome 1 | No gene annotation | No homolog |
| IG1_598325 | 677 | 8E-02 | Intergenic region, chromosome 1 | No gene annotation | No homolog |
| IG1_631117 | 3566 | 1E-02 | Intergenic region, chromosome 1 | No gene annotation | No homolog |
| IG1_701310 | 2765 | 2E-01 | Intergenic region, chromosome 1 | No gene annotation | No homolog |
| IG1_830187 | 2311 | 1E-02 | Intergenic region, chromosome 1 | No gene annotation | No homolog |
| IG1_845294 | 5674 | 2E-02 | Intergenic region, chromosome 1 | No gene annotation | No homolog |
| IG1_846330 | 2308 | 4E-02 | Intergenic region, chromosome 1 | No gene annotation | No homolog |
| IG1_851142 | 8122 | 3E-02 | Intergenic region, chromosome 1 | No gene annotation | No homolog |
| IG1_890632 | 4964 | 1E-18 | Intergenic region, chromosome 1 | No gene annotation | No homolog |
| IG1_898886 | 3393 | 3E-27 | Intergenic region, chromosome 1 | No gene annotation | No homolog |
| IG1_953783 | 2802 | 2E-02 | Intergenic region, chromosome 1 | No gene annotation | No homolog |
| IG1_963484 | 1694 | 2E-01 | Intergenic region, chromosome 1 | No gene annotation | No homolog |
| IG2 | 3212 | 1E-01 | Intergenic region, chromosome 2 | No gene annotation | No homolog |
| IG2_1011813 | 3076 | 3E-02 | Intergenic region, chromosome 2 | No gene annotation | No homolog |
| IG2_1060502 | 2042 | 1E-02 | Intergenic region, chromosome 2 | No gene annotation | No homolog |
| IG2_1092309 | 3131 | 4E-02 | Intergenic region, chromosome 2 | No gene annotation | No homolog |
| IG2_1107856 | 1187 | 2E-02 | Intergenic region, chromosome 2 | No gene annotation | No homolog |
| IG2_1120955 | 4654 | 6E-02 | Intergenic region, chromosome 2 | No gene annotation | No homolog |
| IG2_1135565 | 1086 | 3E-20 | Intergenic region, chromosome 2 | No gene annotation | No homolog |
| IG2_1156093 | 2003 | 4E-02 | Intergenic region, chromosome 2 | No gene annotation | No homolog |
| IG2_1191364 | 2991 | 5E-02 | Intergenic region, chromosome 2 | No gene annotation | No homolog |
| IG2_1207816 | 205 | 2E-02 | Intergenic region, chromosome 2 | No gene annotation | No homolog |
| IG2_1271157 | 211 | 3E-02 | Intergenic region, chromosome 2 | No gene annotation | No homolog |
| IG2_1327122 | 446 | 6E-02 | Intergenic region, chromosome 2 | No gene annotation | No homolog |
| IG2_1423308 | 4108 | 1E-02 | Intergenic region, chromosome 2 | No gene annotation | No homolog |
| IG2_1452240 | 4005 | 3E-02 | Intergenic region, chromosome 2 | No gene annotation | No homolog |
| IG2_1467745 | 2782 | 8E-02 | Intergenic region, chromosome 2 | No gene annotation | No homolog |
| IG2_1474118 | 2379 | 3E-02 | Intergenic region, chromosome 2 | No gene annotation | No homolog |
| IG2_1537005 | 2717 | 4E-01 | Intergenic region, chromosome 2 | No gene annotation | No homolog |
| IG2_1619082 | 1237 | 5E-02 | Intergenic region, chromosome 2 | No gene annotation | No homolog |
| IG2_1622450 | 442 | 2E-02 | Intergenic region, chromosome 2 | No gene annotation | No homolog |
| IG2_16302 | 6952 | 7E-02 | Intergenic region, chromosome 2 | No gene annotation | No homolog |
| IG2_1656016 | 302 | 3E-02 | Intergenic region, chromosome 2 | No gene annotation | No homolog |
| IG2_1719237 | 8073 | 9E-02 | Intergenic region, chromosome 2 | No gene annotation | No homolog |
| IG2_1754913 | 3162 | 5E-02 | Intergenic region, chromosome 2 | No gene annotation | No homolog |
| IG2_175839 | 1294 | 2E-02 | Intergenic region, chromosome 2 | No gene annotation | No homolog |
| IG2_1775692 | 5537 | 7E-02 | Intergenic region, chromosome 2 | No gene annotation | No homolog |
| IG2_1899056 | 2765 | 2E-01 | Intergenic region, chromosome 2 | No gene annotation | No homolog |
| IG2_1926503 | 4774 | 3E-02 | Intergenic region, chromosome 2 | No gene annotation | No homolog |
| IG2_2029035 | 3901 | 3E-02 | Intergenic region, chromosome 2 | No gene annotation | No homolog |
| IG2_2039105 | 348 | 5E-02 | Intergenic region, chromosome 2 | No gene annotation | No homolog |
| IG2_205028 | 2776 | 5E-02 | Intergenic region, chromosome 2 | No gene annotation | No homolog |
| IG2_2055265 | 4479 | 8E-02 | Intergenic region, chromosome 2 | No gene annotation | No homolog |
| IG2_2089610 | 3926 | 1E-02 | Intergenic region, chromosome 2 | No gene annotation | No homolog |
| IG2_2119934 | 1127 | 3E-02 | Intergenic region, chromosome 2 | No gene annotation | No homolog |
| IG2_2146009 | 3908 | 5E-02 | Intergenic region, chromosome 2 | No gene annotation | No homolog |
| IG2_2201625 | 2975 | 3E-02 | Intergenic region, chromosome 2 | No gene annotation | No homolog |
| IG2_2241699 | 208 | 7E-01 | Intergenic region, chromosome 2 | No gene annotation | Bcen2424_4834 |
| IG2_2263493 | 4553 | 2E-02 | Intergenic region, chromosome 2 | No gene annotation | No homolog |
| IG2_2304176 | 3288 | 8E-02 | Intergenic region, chromosome 2 | No gene annotation | No homolog |
| IG2_2328812 | 532 | 2E-01 | Intergenic region, chromosome 2 | No gene annotation | No homolog |
| IG2_2336808 | 2591 | 4E-01 | Intergenic region, chromosome 2 | No gene annotation | No homolog |
| IG2_2342118 | 3323 | 7E-02 | Intergenic region, chromosome 2 | No gene annotation | No homolog |
| IG2_2344521 | 2333 | 5E-02 | Intergenic region, chromosome 2 | No gene annotation | No homolog |
| IG2_2355274 | 3718 | 7E-02 | Intergenic region, chromosome 2 | No gene annotation | No homolog |
| IG2_2434561 | 4668 | 2E-02 | Intergenic region, chromosome 2 | No gene annotation | No homolog |
| IG2_2435392 | 1361 | 6E-02 | Intergenic region, chromosome 2 | No gene annotation | No homolog |
| IG2_2436246 | 2737 | 6E-01 | Intergenic region, chromosome 2 | No gene annotation | No homolog |
| IG2_2510614 | 5749 | 3E-02 | Intergenic region, chromosome 2 | No gene annotation | No homolog |
| IG2_2557825 | 5916 | 4E-02 | Intergenic region, chromosome 2 | No gene annotation | No homolog |
| IG2_2569405 | 2731 | 5E-02 | Intergenic region, chromosome 2 | No gene annotation | No homolog |
| IG2_2570553 | 5918 | 2E-02 | Intergenic region, chromosome 2 | No gene annotation | No homolog |
| IG2_2586922 | 1067 | 2E-02 | Intergenic region, chromosome 2 | No gene annotation | No homolog |
| IG2_2644462 | 2069 | 3E-02 | Intergenic region, chromosome 2 | No gene annotation | No homolog |
| IG2_2694843 | 2229 | 4E-02 | Intergenic region, chromosome 2 | No gene annotation | No homolog |
| IG2_2737778 | 1135 | 5E-02 | Intergenic region, chromosome 2 | No gene annotation | No homolog |
| IG2_2875859 | 4074 | 1E-02 | Intergenic region, chromosome 2 | No gene annotation | No homolog |
| IG2_2878493 | 2927 | 1E-02 | Intergenic region, chromosome 2 | No gene annotation | No homolog |
| IG2_2914215 | 1557 | 3E-02 | Intergenic region, chromosome 2 | No gene annotation | No homolog |
| IG2_293080 | 2141 | 3E-02 | Intergenic region, chromosome 2 | No gene annotation | No homolog |
| IG2_295645 | 245 | 9E-02 | Intergenic region, chromosome 2 | No gene annotation | No homolog |
| IG2_2980616 | 2241 | 2E-02 | Intergenic region, chromosome 2 | No gene annotation | No homolog |
| IG2_2984457 | 3909 | 8E-02 | Intergenic region, chromosome 2 | No gene annotation | No homolog |
| IG2_3010692 | 9625 | 4E-02 | Intergenic region, chromosome 2 | No gene annotation | No homolog |
| IG2_3023656 | 8443 | 6E-02 | Intergenic region, chromosome 2 | No gene annotation | No homolog |
| IG2_305463 | 5493 | 2E-02 | Intergenic region, chromosome 2 | No gene annotation | No homolog |
| IG2_3097968 | 2749 | 6E-02 | Intergenic region, chromosome 2 | No gene annotation | No homolog |
| IG2_326356 | 3571 | 6E-02 | Intergenic region, chromosome 2 | No gene annotation | No homolog |
| IG2_327487 | 5666 | 6E-02 | Intergenic region, chromosome 2 | No gene annotation | No homolog |
| IG2_396034 | 3258 | 2E-02 | Intergenic region, chromosome 2 | No gene annotation | No homolog |
| IG2_408267 | 2373 | 6E-02 | Intergenic region, chromosome 2 | No gene annotation | No homolog |
| IG2_439284 | 3497 | 7E-02 | Intergenic region, chromosome 2 | No gene annotation | No homolog |
| IG2_464303 | 2965 | 3E-02 | Intergenic region, chromosome 2 | No gene annotation | No homolog |
| IG2_519183 | 4749 | 2E-02 | Intergenic region, chromosome 2 | No gene annotation | No homolog |
| IG2_529992 | 9173 | 4E-02 | Intergenic region, chromosome 2 | No gene annotation | No homolog |
| IG2_551370 | 4642 | 3E-02 | Intergenic region, chromosome 2 | No gene annotation | No homolog |
| IG2_55282 | 3209 | 2E-02 | Intergenic region, chromosome 2 | No gene annotation | No homolog |
| IG2_568212 | 6813 | 4E-02 | Intergenic region, chromosome 2 | No gene annotation | No homolog |
| IG2_625427 | 7385 | 9E-01 | Intergenic region, chromosome 2 | No gene annotation | No homolog |
| IG2_643345 | 2721 | 4E-02 | Intergenic region, chromosome 2 | No gene annotation | No homolog |
| IG2_690307 | 2692 | 1E-02 | Intergenic region, chromosome 2 | No gene annotation | No homolog |
| IG2_847551 | 2605 | 2E-02 | Intergenic region, chromosome 2 | No gene annotation | No homolog |
| IG2_902428 | 5246 | 1E-02 | Intergenic region, chromosome 2 | No gene annotation | No homolog |
| IG2_904254 | 2331 | 4E-02 | Intergenic region, chromosome 2 | No gene annotation | No homolog |
| IG2_933755 | 2703 | 9E-02 | Intergenic region, chromosome 2 | No gene annotation | No homolog |
| IG3_106284 | 5295 | 5E-02 | Intergenic region, chromosome 3 | No gene annotation | No homolog |
| IG3_116795 | 1051 | 3E-02 | Intergenic region, chromosome 3 | No gene annotation | No homolog |
| IG3_11785 | 1621 | 6E-02 | Intergenic region, chromosome 3 | No gene annotation | No homolog |
| IG3_202414 | 2074 | 2E-02 | Intergenic region, chromosome 3 | No gene annotation | No homolog |
| IG3_209555 | 3174 | 3E-02 | Intergenic region, chromosome 3 | No gene annotation | No homolog |
| IG3_216945 | 2635 | 2E-01 | Intergenic region, chromosome 3 | No gene annotation | No homolog |
| IG3_242956 | 9206 | 7E-02 | Intergenic region, chromosome 3 | No gene annotation | No homolog |
| IG3_244567 | 1307 | 5E-02 | Intergenic region, chromosome 3 | No gene annotation | No homolog |
| IG3_245540 | 5556 | 4E-02 | Intergenic region, chromosome 3 | No gene annotation | No homolog |
| IG3_264432 | 4611 | 2E-01 | Intergenic region, chromosome 3 | No gene annotation | No homolog |
| IG3_289386 | 6207 | 4E-02 | Intergenic region, chromosome 3 | No gene annotation | No homolog |
| IG3_316621 | 7854 | 3E-02 | Intergenic region, chromosome 3 | No gene annotation | No homolog |
| IG3_480246 | 2871 | 2E-02 | Intergenic region, chromosome 3 | No gene annotation | No homolog |
| IG3_536263 | 2297 | 2E-02 | Intergenic region, chromosome 3 | No gene annotation | No homolog |
| IG3_572966 | 4519 | 1E-02 | Intergenic region, chromosome 3 | No gene annotation | No homolog |
| IG3_584892 | 2586 | 1E-03 | Intergenic region, chromosome 3 | No gene annotation | No homolog |
| IG3_607288 | 914 | 5E-02 | Intergenic region, chromosome 3 | No gene annotation | No homolog |
| IG3_608028 | 9704 | 1E-02 | Intergenic region, chromosome 3 | No gene annotation | No homolog |
| IG3_608364 | 5089 | 2E-01 | Intergenic region, chromosome 3 | No gene annotation | No homolog |
| IG3_688383 | 2401 | 1E-02 | Intergenic region, chromosome 3 | No gene annotation | No homolog |
| IG3_710629 | 119 | 3E-02 | Intergenic region, chromosome 3 | No gene annotation | No homolog |
| IG3_745205 | 4956 | 3E-02 | Intergenic region, chromosome 3 | No gene annotation | No homolog |
| IG3_754644 | 5038 | 7E-02 | Intergenic region, chromosome 3 | No gene annotation | No homolog |
| IG3_757021 | 2932 | 4E-02 | Intergenic region, chromosome 3 | No gene annotation | No homolog |
| IG3_762717 | 2294 | 2E-02 | Intergenic region, chromosome 3 | No gene annotation | No homolog |
| IG3_769736 | 3074 | 7E-02 | Intergenic region, chromosome 3 | No gene annotation | No homolog |
| IG3_781759 | 1115 | 4E-02 | Intergenic region, chromosome 3 | No gene annotation | No homolog |
| IG3_784806 | 2275 | 1E-02 | Intergenic region, chromosome 3 | No gene annotation | No homolog |
| IG3_817239 | 3119 | 1E-02 | Intergenic region, chromosome 3 | No gene annotation | No homolog |
| IG3_858390 | 2807 | 2E-02 | Intergenic region, chromosome 3 | No gene annotation | No homolog |
| IG3_9096 | 1014 | 2E-02 | Intergenic region, chromosome 3 | No gene annotation | No homolog |
|  |  |  |  | ***No assigned COG*** |  |
| BCAL0084 | 48 | 4E-02 | BCAL0084 | putative phage transcriptional activator protein | No homolog |
| BCAL0085 | 2378 | 1E-02 | BCAL0085 | hypothetical protein | No homolog |
| BCAL0086 | 1303 | 4E-02 | BCAL0086 | hypothetical protein | No homolog |
| BCAL0088 | 1451 | 3E-02 | BCAL0088 | hypothetical protein | No homolog |
| BCAL0093 | 2072 | 7E-02 | BCAL0093 | putative phage tail protein | No homolog |
| BCAL0112 | 6718 | 2E-01 | BCAL0112 | hypothetical protein | Bcen2424_0239 |
| BCAL0124 | 2155 | 7E-02 | BCAL0124 | flagellar regulon master regulator subunit FlhD | Bcen2424_0251 |
| BCAL0138_J1 | 3262 | 1E-02 | BCAL0138 | No gene annotation* | No homolog |
| BCAL0169 | 8757 | 3E-02 | BCAL0169 | hypothetical protein | No homolog |
| BCAL0172 | 3617 | 8E-02 | BCAL0172 | putative plasmid conjugal transfer protein | No homolog |
| BCAL0175 | 5952 | 9E-01 | BCAL0175 | hypothetical protein | No homolog |
| BCAL0177 | 2094 | 8E-01 | BCAL0177 | putative plasmid conjugal transfer protein | No homolog |
| BCAL0180 | 2747 | 4E-02 | BCAL0180 | hypothetical protein | No homolog |
| BCAL0182 | 4763 | 4E-02 | BCAL0182 | putative plasmid recombinase | No homolog |
| BCAL0218 | 2015 | 6E-02 | BCAL0218 | hypothetical protein | No homolog |
| BCAL0336 | 9316 | 1E-02 | BCAL0336 | hypothetical protein | No homolog |
| BCAL0352 | 3516 | 3E-01 | BCAL0352 | metallo peptidase, subfamily M15C* | Bcen2424_0486 |
| BCAL0412 | 207 | 2E-02 | BCAL0412 | No gene annotation | No homolog |
| BCAL0419 | 648 | 4E-02 | BCAL0419 | hypothetical protein | No homolog |
| BCAL0440 | 9677 | 2E-02 | BCAL0440 | hypothetical protein* | Bcen2424_3148 |
| BCAL0542 | 3257 | 1E-02 | BCAL0542 | putative iron-sulfur protein* | Bcen2424_3047 |
| BCAL0582 | 2185 | 1E-02 | BCAL0582 | hypothetical protein | No homolog |
| BCAL0656 | 1207 | 4E-02 | BCAL0656 | putative aminotransferase | No homolog |
| BCAL0789 | 2758 | 6E-02 | BCAL0789 | hypothetical protein | Bcen2424_2813 |
| BCAL0822 | 2388 | 1E-03 | BCAL0822 | NUDIX hydrolase | Bcen2424_2782 |
| BCAL0948 | 2148 | 1E-02 | BCAL0948 | putative plasmid-related recombination enzyme | No homolog |
| BCAL0949 | 2474 | 3E-02 | BCAL0949 | putative phage-related integrase | No homolog |
| BCAL1028_J2_1 | 1207 | 1E-02 | BCAL1028 | No gene annotation | No homolog |
| BCAL1082 | 2009 | 8E-02 | BCAL1082 | No gene annotation | Bcen2424_1199 |
| BCAL1119 | 1575 | 2E-02 | BCAL1119 | hypothetical protein | No homolog |
| BCAL1121 | 4996 | 5E-02 | BCAL1121 | hypothetical protein | No homolog |
| BCAL1122 | 4463 | 2E-02 | BCAL1122 | hypothetical protein | No homolog |
| BCAL1124 | 4474 | 2E-02 | BCAL1124 | hypothetical protein | No homolog |
| BCAL1128 | 5244 | 1E-02 | BCAL1128 | putative DNA-binding protein | No homolog |
| BCAL1129 | 2308 | 2E-02 | BCAL1129 | hypothetical protein | No homolog |
| BCAL1130 | 4041 | 6E-02 | BCAL1130 | hypothetical protein | No homolog |
| BCAL1131 | 2646 | 1E-02 | BCAL1131 | hypothetical protein | No homolog |
| BCAL1143 | 2601 | 4E-02 | BCAL1143 | No gene annotation | No homolog |
| BCAL1144 | 2778 | 2E-02 | BCAL1144 | hypothetical protein | No homolog |
| BCAL1145 | 8387 | 5E-01 | BCAL1145 | hypothetical protein | No homolog |
| BCAL1161 | 6136 | 7E-02 | BCAL1161 | hypothetical protein* | No homolog |
| BCAL1166 | 1506 | 4E-02 | BCAL1166 | hypothetical protein* | No homolog |
| BCAL1169 | 1187 | 8E-02 | BCAL1169 | No gene annotation* | No homolog |
| BCAL1184 | 593 | 8E-02 | BCAL1184 | No gene annotation | No homolog |
| BCAL1187 | 1834 | 3E-02 | BCAL1187 | hypothetical protein | No homolog |
| BCAL1192 | 4515 | 1E-02 | BCAL1192 | putative DNA-binding protein | No homolog |
| BCAL1193 | 2736 | 8E-02 | BCAL1193 | hypothetical protein* | No homolog |
| BCAL1200 | 4513 | 5E-02 | BCAL1200 | hypothetical protein | No homolog |
| BCAL2508 | 4816 | 1E-01 | BCAL1200 | hypothetical protein | No homolog |
| BCAL1236 | 2376 | 2E-02 | BCAL1236 | hypothetical protein* | Bcen2424_1265 |
| BCAL1244 | 2631 | 2E-02 | BCAL1244 | putative malonate decarboxylase* | Bcen2424_1273 |
| BCAL1279 | 2223 | 8E-02 | BCAL1279 | hypothetical protein* | No homolog |
| BCAL1291 | 4175 | 1E-02 | BCAL1291 | hypothetical protein | No homolog |
| BCAL1292 | 1916 | 1E-02 | BCAL1292 | hypothetical protein | No homolog |
| BCAL1296 | 2065 | 1E-01 | BCAL1296 | hypothetical protein | No homolog |
| BCAL1298 | 1152 | 2E-02 | BCAL1298 | hypothetical protein* | No homolog |
| BCAL1299 | 1199 | 6E-02 | BCAL1299 | hypothetical protein | No homolog |
| BCAL1300 | 1113 | 2E-02 | BCAL1300 | hypothetical protein | No homolog |
| BCAL1301 | 4064 | 1E-02 | BCAL1301 | hypothetical protein | No homolog |
| BCAL1316 | 7158 | 6E-02 | BCAL1316 | hypothetical protein | No homolog |
| BCAL1317 | 1326 | 2E-02 | BCAL1317 | putative phage integrase | No homolog |
| BCAL1353 | 2186 | 3E-02 | BCAL1353 | hypothetical protein | No homolog |
| BCAL1356 | 3895 | 1E-02 | BCAL1356 | hypothetical protein | No homolog |
| BCAL1357 | 261 | 1E-02 | BCAL1357 | hypothetical protein* | No homolog |
| BCAL1358 | 1591 | 7E-02 | BCAL1358 | hypothetical protein | No homolog |
| BCAL1360 | 1105 | 5E-02 | BCAL1360 | hypothetical protein | No homolog |
| BCAL1363 | 3344 | 7E-02 | BCAL1363 | hypothetical protein | No homolog |
| BCAL1365 | 4465 | 1E-02 | BCAL1365 | putative lipoprotein* | No homolog |
| BCAL1366 | 1524 | 2E-02 | BCAL1366 | hypothetical protein | No homolog |
| BCAL1382 | 2014 | 2E-02 | BCAL1382 | hypothetical protein* | No homolog |
| BCAL1446 | 2176 | 8E-02 | BCAL1446 | putative lipoprotein* | Bcen2424_1439 |
| BCAL1454 | 2523 | 1E-02 | BCAL1454 | hypothetical protein | Bcen2424_1447 |
| BCAL1459 | 2169 | 1E-02 | BCAL1459 | calcineurin-like phosphoesterase | No homolog |
| BCAL1495 | 3545 | 4E-02 | BCAL1495 | hypothetical protein | Bcen2424_1488 |
| BCAL1539 | 2068 | 1E-02 | BCAL1539 | hypothetical protein* | Bcen2424_1532 |
| BCAL1559 | 5795 | 1E-02 | BCAL1559 | hypothetical protein | No homolog |
| BCAL1560 | 5585 | 2E-02 | BCAL1560 | hypothetical protein | No homolog |
| BCAL1569 | 2573 | 2E-02 | BCAL1569 | hypothetical protein | No homolog |
| BCAL1570 | 3498 | 6E-02 | BCAL1570 | hypothetical protein | No homolog |
| BCAL1577 | 2121 | 1E-02 | BCAL1577 | hypothetical protein | No homolog |
| BCAL1583 | 2012 | 7E-02 | BCAL1583 | hypothetical protein | No homolog |
| BCAL1584 | 3074 | 3E-02 | BCAL1584 | hypothetical protein | No homolog |
| BCAL1586 | 2964 | 2E-01 | BCAL1586 | hypothetical protein | No homolog |
| BCAL1588 | 2659 | 7E-02 | BCAL1588 | hypothetical protein | No homolog |
| BCAL1590 | 2048 | 2E-02 | BCAL1590 | hypothetical protein | No homolog |
| BCAL1596 | 8739 | 3E-02 | BCAL1596 | hypothetical protein | No homolog |
| BCAL1598 | 2232 | 1E-03 | BCAL1598 | hypothetical protein* | No homolog |
| BCAL1603 | 2125 | 7E-02 | BCAL1603 | hypothetical protein | No homolog |
| BCAL1605 | 3515 | 6E-02 | BCAL1605 | hypothetical protein | No homolog |
| BCAL1626 | 2803 | 1E-02 | BCAL1626 | hypothetical protein | No homolog |
| BCAL1628 | 5437 | 6E-02 | BCAL1628 | hypothetical protein | No homolog |
| BCAL1629 | 2355 | 6E-02 | BCAL1629 | hypothetical protein | No homolog |
| BCAL1630 | 2561 | 4E-02 | BCAL1630 | hypothetical protein | No homolog |
| BCAL1632 | 4094 | 7E-02 | BCAL1632 | putative lipoprotein* | No homolog |
| BCAL1633 | 3933 | 2E-02 | BCAL1633 | No gene annotation | No homolog |
| BCAL1672_J0 | 3068 | 1E-03 | BCAL1672 | No gene annotation | No homolog |
| BCAL1672_J1 | 3096 | 2E-02 | BCAL1672 | No gene annotation | Bcen2424_1621 |
| BCAL1680 | 2821 | 8E-02 | BCAL1680 | putative type-1 fimbrial protein* | No homolog |
| BCAL1816 | 6848 | 3E-02 | BCAL1816 | hypothetical protein* | No homolog |
| BCAL1833 | 7734 | 2E-02 | BCAL1833 | hypothetical protein* | Bcen2424_1760 |
| BCAL1844 | 2091 | 1E-03 | BCAL1844 | hypothetical protein | Bcen2424_1771 |
| BCAL1848 | 2437 | 3E-02 | BCAL1848 | hypothetical protein* | No homolog |
| BCAL1857 | 3734 | 4E-02 | BCAL1857 | hypothetical protein | No homolog |
| BCAL1954 | 2438 | 5E-02 | BCAL1954 | hypothetical protein* | No homolog |
| BCAL1972 | 5298 | 2E-01 | BCAL1972 | hypothetical protein | No homolog |
| BCAL1974 | 3894 | 1E-03 | BCAL1974 | putative lipoprotein* | Bcen2424_1902 |
| BCAL2081 | 2181 | 3E-02 | BCAL2081 | UDP-3-O-[3-hydroxymyristoyl] glucosamine N-acyltransferase | Bcen2424_2009 |
| BCAL2099 | 2015 | 4E-02 | BCAL2099 | hypothetical protein | Bcen2424_2027 |
| BCAL2159 | 1137 | 4E-02 | BCAL2159 | hypothetical protein* | Bcen2424_2087 |
| BCAL2161 | 2303 | 1E-02 | BCAL2161 | hypothetical protein* | Bcen2424_2089 |
| BCAL2206 | 234 | 2E-02 | BCAL2206 | phasin-like protein | Bcen2424_2134 |
| BCAL2270 | 1849 | 3E-02 | BCAL2270 | hypothetical protein | No homolog |
| BCAL2277 | 3337 | 4E-02 | BCAL2277 | hypothetical protein | No homolog |
| BCAL2279 | 3005 | 9E-02 | BCAL2279 | No gene annotation | No homolog |
| BCAL2307 | 3685 | 3E-01 | BCAL2307 | hypothetical protein | No homolog |
| BCAL2439 | 3654 | 9E-02 | BCAL2439 | hypothetical protein | No homolog |
| BCAL2461 | 2634 | 3E-02 | BCAL2461 | hypothetical protein | Bcen2424_2382 |
| BCAL2491 | 1422 | 1E-02 | BCAL2491 | hypothetical protein* | No homolog |
| BCAL2492 | 5317 | 1E-02 | BCAL2492 | putative transport permease protein | No homolog |
| BCAL2500 | 336 | 1E-03 | BCAL2500 | hypothetical protein | No homolog |
| BCAL2501 | 4544 | 3E-02 | BCAL2501 | hypothetical protein | No homolog |
| BCAL2505 | 4477 | 1E-02 | BCAL2505 | hypothetical protein | No homolog |
| BCAL2506 | 7392 | 1E-02 | BCAL2506 | hypothetical protein | No homolog |
| BCAL2507 | 2126 | 2E-02 | BCAL2507 | hypothetical protein | No homolog |
| BCAL2516 | 5747 | 9E-02 | BCAL2516 | hypothetical protein | No homolog |
| BCAL2517 | 9308 | 1E-02 | BCAL2517 | hypothetical protein | No homolog |
| BCAL2520 | 13 | 7E-02 | BCAL2520 | putative lipoprotein* | No homolog |
| BCAL2523A | 1241 | 2E-02 | BCAL2523A | hypothetical protein | No homolog |
| BCAL2526 | 2666 | 1E-02 | BCAL2526 | No gene annotation | No homolog |
| BCAL2531 | 463 | 1E-02 | BCAL2531 | hypothetical protein | No homolog |
| BCAL2548_J0 | 229 | 8E-02 | BCAL2548 | No gene annotation* | No homolog |
| BCAL2552 | 6482 | 6E-02 | BCAL2552 | hypothetical protein | No homolog |
| BCAL2555 | 2185 | 8E-02 | BCAL2555 | hypothetical protein | No homolog |
| BCAL2556 | 2364 | 2E-02 | BCAL2556 | putative helicase | No homolog |
| BCAL2557 | 2133 | 7E-02 | BCAL2557 | hypothetical protein | No homolog |
| BCAL2562 | 2971 | 3E-02 | BCAL2562 | No gene annotation | No homolog |
| BCAL2565 | 2767 | 1E-03 | BCAL2565 | hypothetical protein | No homolog |
| BCAL2567 | 5044 | 8E-01 | BCAL2567 | hypothetical protein | No homolog |
| BCAL2568 | 1706 | 3E-02 | BCAL2568 | No gene annotation | No homolog |
| BCAL2569 | 3815 | 9E-02 | BCAL2569 | hypothetical protein | No homolog |
| BCAL2588 | 3169 | 4E-02 | BCAL2588 | No gene annotation | No homolog |
| BCAL2596 | 3805 | 3E-02 | BCAL2596 | hypothetical protein | No homolog |
| BCAL2597 | 2976 | 2E-02 | BCAL2597 | hypothetical protein | No homolog |
| BCAL2599 | 2906 | 7E-01 | BCAL2599 | hypothetical protein | No homolog |
| BCAL2600 | 139 | 4E-00 | BCAL2600 | integrase | No homolog |
| BCAL2601_J0 | 7011 | 6E-02 | BCAL2601 | No gene annotation | No homolog |
| BCAL2607 | 256 | 6E-02 | BCAL2607 | hypothetical protein* | No homolog |
| BCAL2846 | 2641 | 2E-02 | Multiple hits | No gene annotation | Bcen2424_1150 |
| BCAL2847 | 7163 | 1E-02 | Multiple hits | No gene annotation | No homolog |
| BCAL2848 | 4507 | 1E-02 | Multiple hits | No gene annotation | Bcen2424_1148 |
| BCAL2851 | 2028 | 2E-02 | Multiple hits | No gene annotation | Bcen2424_1145 |
| BCAL2852 | 1079 | 5E-22 | Multiple hits | No gene annotation | No homolog |
| BCAL2859 | 4983 | 2E-02 | Multiple hits | No gene annotation | Bcen2424_1140 |
| BCAL2860 | 3464 | 3E-02 | Multiple hits | No gene annotation | Bcen2424_1139 |
| BCAL2867 | 3002 | 2E-02 | Multiple hits | No gene annotation | Bcen2424_1132 |
| BCAL2872 | 5063 | 1E-01 | Multiple hits | No gene annotation | Bcen2424_1127 |
| BCAL2873 | 3242 | 1E-02 | Multiple hits | No gene annotation | Bcen2424_1126 |
| BCAL2874 | 3896 | 3E-02 | Multiple hits | No gene annotation | Bcen2424_1125 |
| BCAL2875 | 2396 | 5E-02 | Multiple hits | No gene annotation | Bcen2424_1124 |
| BCAL2877 | 2357 | 1E-02 | Multiple hits | No gene annotation | Bcen2424_1122 |
| BCAL2878 | 2451 | 3E-02 | Multiple hits | No gene annotation | Bcen2424_1121 |
| BCAL2883 | 241 | 1E-03 | Multiple hits | No gene annotation | Bcen2424_1116 |
| BCAL2886 | 2185 | 4E-02 | Multiple hits | No gene annotation | Bcen2424_1113 |
| BCAL2892 | 2576 | 5E-02 | Multiple hits | No gene annotation | Bcen2424_1106 |
| BCAL2896 | 3021 | 1E-02 | Multiple hits | No gene annotation | Bcen2424_1102 |
| BCAL2905 | 2305 | 8E-02 | BCAL2905 | hypothetical protein | No homolog |
| BCAL2962 | 2265 | 8E-02 | BCAL2962 | hypothetical protein | No homolog |
| BCAL2963 | 8753 | 2E-02 | BCAL2963 | putative phage-related DNA-binding protein | No homolog |
| BCAL2964 | 5037 | 8E-02 | BCAL2964 | putative phage-related DNA-binding protein | No homolog |
| BCAL2965 | 4391 | 3E-02 | BCAL2965 | hypothetical protein | No homolog |
| BCAL2965a | 4061 | 2E-02 | BCAL2965a | hypothetical protein | No homolog |
| BCAL2965C | 8974 | 3E-02 | BCAL2965C | hypothetical protein | No homolog |
| BCAL2968 | 2002 | 7E-02 | BCAL2968 | hypothetical protein | No homolog |
| BCAL2969 | 1503 | 2E-02 | BCAL2969 | hypothetical protein | No homolog |
| BCAL2969a | 4492 | 8E-02 | BCAL2969a | hypothetical protein | No homolog |
| BCAL2970 | 372 | 7E-02 | BCAL2970 | hypothetical protein | No homolog |
| BCAL2971 | 9475 | 2E-02 | BCAL2971 | hypothetical protein | No homolog |
| BCAL2971a | 5738 | 3E-02 | BCAL2971a | hypothetical protein | No homolog |
| BCAL2972 | 5318 | 3E-02 | BCAL2972 | hypothetical protein | No homolog |
| BCAL2973 | 1309 | 1E-02 | BCAL2973 | hypothetical protein* | Bcen2424_1036 |
| BCAL2974 | 2518 | 2E-02 | BCAL2974 | hypothetical protein | Bcen2424_1035 |
| BCAL2979 | 308 | 8E-02 | BCAL2979 | NAD-dependent formate dehydrogenase delta subunit | Bcen2424_1030 |
| BCAL3017 | 2315 | 5E-02 | BCAL3017 | hypothetical protein* | No homolog |
| BCAL3031 | 2029 | 4E-02 | BCAL3031 | hypothetical protein | No homolog |
| BCAL3075 | 3699 | 3E-02 | BCAL3075 | No gene annotation | No homolog |
| BCAL3076 | 9151 | 1E-02 | BCAL3076 | putative integrase | No homolog |
| BCAL3078_J0 | 4161 | 4E-02 | BCAL3078 | putative lipoprotein* | No homolog |
| BCAL3079 | 6867 | 2E-01 | BCAL3079 | hypothetical protein | No homolog |
| BCAL3080 | 6696 | 6E-02 | BCAL3080 | hypothetical protein | No homolog |
| BCAL3081 | 2927 | 2E-02 | BCAL3081 | hypothetical protein | No homolog |
| BCAL3083 | 2389 | 3E-02 | BCAL3083 | hypothetical protein | No homolog |
| BCAL3125 | 8116 | 2E-02 | BCAL3125 | No gene annotation | No homolog |
| BCAL3148 | 2724 | 5E-02 | BCAL3148 | hypothetical protein | Bcen2424_0858 |
| BCAL3150 | 5443 | 1E-02 | BCAL3150 | hypothetical protein* | Bcen2424_0856 |
| BCAL3154 | 2178 | 5E-02 | BCAL3154 | putative glycine-rich surface protein | No homolog |
| BCAL3217 | 1014 | 4E-01 | BCAL3217 | putative acetyltransferase protein | No homolog |
| BCAL3223_J0 | 722 | 4E-02 | BCAL3223 | No gene annotation* | No homolog |
| BCAL3223_J1 | 2961 | 1E-02 | BCAL3223 | No gene annotation* | No homolog |
| BCAL3228 | 2498 | 3E-02 | BCAL3228 | hypothetical protein | No homolog |
| BCAL3232 | 6545 | 1E-02 | BCAL3232 | hypothetical protein | No homolog |
| BCAL3236 | 4557 | 3E-02 | Multiple hits | No gene annotation | No homolog |
| BCAL3237 | 1358 | 3E-02 | Multiple hits | No gene annotation | No homolog |
| BCAL3249_J1 | 1281 | 4E-01 | BCAL3249 | No gene annotation | No homolog |
| BCAL3250 | 1056 | 1E-03 | Multiple hits | No gene annotation | No homolog |
| BCAL3258 | 7398 | 3E-02 | BCAL3258 | tetracycline repressor protein | Bcen2424_0763 |
| BCAL3259_J0 | 488 | 9E-02 | BCAL3259 | No gene annotation | No homolog |
| BCAL3259_J1 | 6735 | 1E-02 | BCAL3259 | No gene annotation | Bcen2424_0762 |
| BCAL3271 | 2304 | 5E-02 | BCAL3271 | thioredoxin | No homolog |
| BCAL3298 | 5653 | 3E-02 | BCAL3298 | hypothetical protein* | Bcen2424_0727 |
| BCAL3354_J1 | 2101 | 4E-01 | BCAL3354 | No gene annotation | Bcen2424_0672 |
| BCAL3438 | 2131 | 5E-02 | BCAL3438 | putative lipoprotein* | Bcen2424_0585 |
| BCALr0080 | 2263 | 4E-02 | BCALr0080 | tRNA-Arg | Bcen2424_R0005 |
| BCALr0164 | 7282 | 4E-02 | BCALr0164 | tRNA-Ala | Bcen2424_R0006 |
| BCALr0332 | 9336 | 4E-02 | BCALr0332 | tRNA-Thr | Bcen2424_R0015 |
| BCALr0457 | 778 | 3E-02 | BCALr0457 | tRNA-Lys | Bcen2424_R0073 |
| BCALr1117 | 2292 | 5E-02 | BCALr1117 | misc_RNA | Bcen2424_1236 |
| BCALr1279 | 2627 | 5E-00 | BCALr1279 | tRNA-Pro | Bcen2424_R0037 |
| BCALr1551a | 3279 | 6E-02 | Multiple hits | No gene annotation | Multiple hits |
| BCALr2125c | 2158 | 2E-02 | Multiple hits | No gene annotation | Multiple hits |
| BCALr2125e | 2658 | 3E-02 | Multiple hits | No gene annotation | Multiple hits |
| BCALr2125f | 1922 | 3E-02 | Multiple hits | No gene annotation | Multiple hits |
| BCALr2219 | 2124 | 1E-02 | BCALr2219 | tRNA-Met | Bcen2424_R0058 |
| BCALr1614 | 2328 | 1E-02 | Multiple hits | No gene annotation | Multiple hits |
| BCALr2687 | 2411 | 2E-02 | BCALr2687 | tRNA-Leu | Bcen2424_R0069 |
| BCALr2852a | 3154 | 5E-02 | Multiple hits | No gene annotation | Bcen2424_R0036 |
| BCALr2852d | 2666 | 1E-02 | Multiple hits | No gene annotation | Multiple hits |
| BCALr0970a | 1612 | 2E-02 | Multiple hits | No gene annotation | Multiple hits |
| IG1_3319801 | 3223 | 1E-01 | BCALr3029 | tRNA-Ser | Bcen2424_R0025 |
| BCALr3075 | 2985 | 3E-02 | BCALr3075 | tRNA-Gly | Bcen2424_R0024 |
| BCALr3205 | 1349 | 3E-00 | BCALr3205 | tRNA-Lys | Bcen2424_R0023 |
| BCALr3443 | 1459 | 3E-02 | BCALr3443 | tRNA-Pro | Bcen2424_R0021 |
| BCAM0022 | 2022 | 4E-02 | Multiple hits | No gene annotation | Multiple hits |
| BCAM0041 | 482 | 4E-02 | BCAM0041 | putative lipoprotein* | No homolog |
| BCAM0074 | 848 | 1E-02 | BCAM0074 | hypothetical protein | No homolog |
| BCAM0147 | 6687 | 6E-02 | BCAM0147 | hypothetical protein | No homolog |
| BCAM0151 | 8393 | 2E-02 | BCAM0151 | No gene annotation | No homolog |
| BCAM0159 | 3157 | 1E-01 | BCAM0159 | conserved ypothetical protein | No homolog |
| BCAM0202 | 2071 | 5E-02 | BCAM0202 | hypothetical protein* | No homolog |
| BCAM0216 | 6549 | 1E-02 | BCAM0216 | hypothetical protein | No homolog |
| BCAM0217 | 7173 | 5E-02 | BCAM0217 | hypothetical protein | No homolog |
| BCAM0225 | 38 | 9E-02 | BCAM0225 | No gene annotation* | No homolog |
| BCAL3293 | 2475 | 4E-02 | Multiple hits | No gene annotation | No homolog |
| BCAL3295 | 6743 | 5E-02 | Multiple hits | No gene annotation | No homolog |
| BCAM0258 | 2346 | 2E-02 | BCAM0258 | repressor protein | No homolog |
| BCAM0270 | 404 | 2E-02 | BCAM0270 | hypothetical protein | No homolog |
| BCAM0273 | 7192 | 2E-02 | BCAM0273 | No gene annotation | No homolog |
| BCAM0274a | 2034 | 3E-02 | BCAM0274a | No gene annotation | No homolog |
| BCAM0275 | 2508 | 9E-02 | BCAM0275 | hypothetical protein | No homolog |
| BCAM0280A | 5278 | 1E-01 | BCAM0280A | hypothetical protein | No homolog |
| BCAM0282 | 2107 | 7E-02 | BCAM0282 | hypothetical protein | No homolog |
| BCAM0301 | 2411 | 6E-02 | BCAM0301 | hypothetical protein | No homolog |
| BCAM0335_J0 | 2521 | 1E-02 | BCAM0335 | No gene annotation | Bcen2424_3322 |
| BCAM0340 | 2336 | 1E-02 | BCAM0340 | hypothetical protein | No homolog |
| BCAM0345 | 2154 | 8E-02 | BCAM0345 | hypothetical protein* | Bcen2424_3331 |
| BCAM0354 | 1251 | 4E-02 | BCAM0354 | putative transcriptional regulator | No homolog |
| BCAM0384 | 2142 | 5E-02 | BCAM0384 | putative lipoprotein* | Bcen2424_3373 |
| BCAM0398 | 2581 | 8E-01 | BCAM0398 | hypothetical protein | No homolog |
| BCAM0428 | 2336 | 2E-02 | BCAM0428 | hypothetical protein* | No homolog |
| BCAM0454_J0 | 1337 | 1E-02 | BCAM0454 | No gene annotation | No homolog |
| BCAM0457 | 8319 | 1E-02 | BCAM0457 | hypothetical protein* | No homolog |
| BCAM0475 | 6094 | 1E-02 | BCAM0475 | hypothetical protein | Bcen2424_3437 |
| BCAM0476 | 1798 | 3E-02 | BCAM0476 | hypothetical protein | No homolog |
| BCAM0488 | 6597 | 3E-02 | BCAM0488 | hypothetical protein* | No homolog |
| BCAM0497 | 2146 | 1E-02 | BCAM0497 | putative lipoprotein* | No homolog |
| BCAM0510 | 8654 | 2E-02 | BCAM0510 | hypothetical protein | No homolog |
| BCAM0529A | 1955 | 2E-02 | BCAM0529A | hypothetical protein* | No homolog |
| BCAM0546 | 9347 | 5E-02 | BCAM0546 | hypothetical protein | No homolog |
| BCAM0616 | 3995 | 1E-02 | BCAM0616 | hypothetical protein | Bcen2424_3587 |
| BCAM0643 | 2852 | 3E-01 | BCAM0643 | hypothetical protein | Bcen2424_3614 |
| BCAM0694 | 2076 | 8E-02 | BCAM0694 | hypothetical protein | Bcen2424_3660 |
| BCAM0709 | 4545 | 2E-02 | BCAM0709 | hypothetical protein* | No homolog |
| BCAM0723 | 3225 | 1E-02 | BCAM0723 | hypothetical protein* | No homolog |
| BCAM0786 | 1264 | 3E-02 | BCAM0786 | hypothetical protein | No homolog |
| BCAM0788 | 1587 | 9E-02 | BCAM0788 | No gene annotation | No homolog |
| BCAM0800 | 2916 | 6E-02 | BCAM0800 | hypothetical protein | Bcen2424_3765 |
| BCAM0828 | 3621 | 7E-02 | BCAM0828 | hypothetical protein | No homolog |
| BCAM0838 | 3578 | 1E-02 | BCAM0838 | hypothetical protein | No homolog |
| BCAM0853 | 2544 | 3E-02 | BCAM0853 | hypothetical protein | Bcen2424_3818 |
| BCAM0895 | 275 | 4E-01 | BCAM0895 | hypothetical protein | No homolog |
| BCAM0929 | 1158 | 5E-02 | BCAM0929 | hypothetical protein* | No homolog |
| BCAM0942 | 3391 | 6E-02 | BCAM0942 | hypothetical protein* | No homolog |
| BCAM0983A | 3387 | 4E-02 | BCAM0983A | putative entericidin B-like bacteriolytic toxin* | No homolog |
| BCAM0988 | 5526 | 4E-02 | BCAM0988 | hypothetical protein* | No homolog |
| BCAM1026 | 5969 | 1E-02 | BCAM1026 | putative phage DNA-binding protein | No homolog |
| BCAM1028 | 4089 | 4E-02 | BCAM1028 | hypothetical protein | No homolog |
| BCAM1029 | 4647 | 1E-02 | BCAM1029 | hypothetical protein | No homolog |
| BCAM1034 | 7429 | 3E-02 | BCAM1034 | hypothetical protein | No homolog |
| BCAM1035 | 4534 | 3E-02 | BCAM1035 | hypothetical protein | No homolog |
| BCAM1036 | 627 | 1E-02 | BCAM1036 | phage methyltransferase | No homolog |
| BCAM1038 | 8177 | 5E-02 | BCAM1038 | hypothetical protein | No homolog |
| BCAM1039 | 9912 | 8E-01 | BCAM1039 | hypothetical protein | No homolog |
| BCAM1040 | 2835 | 4E-02 | BCAM1040 | hypothetical protein | No homolog |
| BCAM1044 | 5559 | 3E-02 | BCAM1044 | hypothetical protein | No homolog |
| BCAM1045 | 3584 | 5E-02 | BCAM1045 | hypothetical protein | No homolog |
| BCAM1046 | 306 | 2E-02 | BCAM1046 | hypothetical protein | No homolog |
| BCAM1047 | 409 | 3E-02 | BCAM1047 | hypothetical protein | No homolog |
| BCAM1048 | 5885 | 3E-02 | BCAM1048 | hypothetical protein* | No homolog |
| BCAM1049 | 4729 | 3E-01 | BCAM1049 | hypothetical protein | No homolog |
| BCAM1050 | 6471 | 3E-02 | BCAM1050 | hypothetical protein | No homolog |
| BCAM1052 | 4077 | 4E-01 | BCAM1052 | hypothetical protein | No homolog |
| BCAM1053a | 1598 | 1E-02 | BCAM1053a | putative phage DNA-binding protein | No homolog |
| IG2_1140182 | 4486 | 2E-02 | BCAM1053a | putative phage DNA-binding protein | No homolog |
| BCAM1053C | 2616 | 6E-02 | BCAM1053C | hypothetical protein | No homolog |
| BCAM1054 | 2141 | 4E-02 | BCAM1054 | putative phage DNA-binding protein | No homolog |
| BCAM1059 | 2673 | 7E-02 | BCAM1059 | putative phage DNA binding protein | No homolog |
| BCAM1061 | 304 | 3E-02 | BCAM1061 | hypothetical protein | No homolog |
| BCAM1062 | 205 | 1E-02 | BCAM1062 | hypothetical protein | No homolog |
| BCAM1067 | 2465 | 4E-02 | BCAM1067 | hypothetical protein | No homolog |
| BCAM1070 | 172 | 8E-02 | BCAM1070 | hypothetical protein | No homolog |
| BCAM1071 | 6017 | 3E-02 | BCAM1071 | hypothetical protein | No homolog |
| BCAM1072 | 3494 | 1E-02 | BCAM1072 | hypothetical protein | No homolog |
| BCAM1079 | 3182 | 6E-02 | BCAM1079 | hypothetical protein | No homolog |
| BCAM1080 | 874 | 2E-02 | BCAM1080 | hypothetical protein | No homolog |
| BCAM1081 | 194 | 7E-02 | BCAM1081 | hypothetical protein | No homolog |
| BCAM1082 | 2706 | 8E-02 | BCAM1082 | hypothetical protein | No homolog |
| BCAM1082A | 9948 | 1E-02 | BCAM1082A | putative exported phage protein* | No homolog |
| BCAM1084 | 2359 | 1E-02 | BCAM1084 | hypothetical protein | No homolog |
| BCAM1086 | 2122 | 2E-02 | BCAM1086 | hypothetical protein | No homolog |
| BCAM1089 | 2117 | 4E-01 | BCAM1089 | putative exported phage protein* | No homolog |
| BCAM1090 | 2164 | 2E-02 | BCAM1090 | putative phage lipoprotein* | No homolog |
| BCAM1094 | 2032 | 4E-02 | BCAM1094 | hypothetical protein | No homolog |
| BCAM1097 | 4769 | 9E-02 | BCAM1097 | hypothetical protein | No homolog |
| BCAM1102 | 7514 | 5E-02 | BCAM1102 | No gene annotation | No homolog |
| BCAM1163 | 5933 | 4E-02 | BCAM1163 | hypothetical protein | Bcen2424_4057 |
| BCAM1215 | 2957 | 3E-02 | BCAM1215 | hypothetical protein* | Bcen2424_4089 |
| BCAM1242 | 283 | 4E-02 | BCAM1242 | hypothetical protein* | Bcen2424_4117 |
| BCAM1248 | 3554 | 2E-02 | BCAM1248 | putative oxygenase | Bcen2424_4124 |
| BCAM1278 | 2045 | 2E-02 | BCAM1278 | hypothetical protein* | No homolog |
| BCAM1327 | 3239 | 2E-02 | BCAM1327 | hypothetical protein | No homolog |
| BCAM1328 | 3666 | 5E-02 | BCAM1328 | No gene annotation* | Bcen2424_4196 |
| BCAM1347 | 2143 | 5E-02 | BCAM1347 | putative acyl carrier protein | Bcen2424_4214 |
| BCAM1363 | 2741 | 5E-02 | BCAM1363 | hypothetical protein | Bcen2424_4228 |
| BCAM1410 | 3344 | 1E-03 | BCAM1410 | putative lipoprotein* | No homolog |
| BCAM1413a | 1368 | 2E-02 | BCAM1413a | hypothetical protein | Bcen2424_4278 |
| BCAM1414 | 3424 | 2E-01 | BCAM1414 | hypothetical protein | Bcen2424_4279 |
| BCAM1451 | 1569 | 6E-02 | BCAM1451 | hypothetical protein* | Bcen2424_4323 |
| BCAM1464 | 2354 | 9E-02 | BCAM1464 | hypothetical protein | No homolog |
| BCAM1490 | 2513 | 2E-01 | BCAM1490 | hypothetical protein* | No homolog |
| BCAM1491 | 3073 | 6E-01 | BCAM1491 | hypothetical protein* | Bcen2424_4363 |
| BCAM1498_J1 | 2534 | 8E-02 | BCAM1498 | No gene annotation | Bcen2424_4371 |
| IG2_1703077 | 3123 | 8E-02 | BCAM1535 | putative lipoprotein | No homolog |
| BCAM1557 | 5312 | 2E-02 | BCAM1557 | hypothetical protein | No homolog |
| BCAM1594 | 2038 | 1E-02 | BCAM1594 | hypothetical protein | No homolog |
| BCAM1604 | 2756 | 1E-02 | BCAM1604 | hypothetical protein* | Bcen2424_4460 |
| BCAM1617 | 3602 | 3E-02 | BCAM1617 | hypothetical protein | No homolog |
| BCAM1667 | 1377 | 2E-02 | BCAM1667 | hypothetical protein | No homolog |
| BCAM1669 | 4856 | 1E-02 | BCAM1669 | hypothetical protein* | No homolog |
| BCAM1696 | 3626 | 3E-02 | BCAM1696 | putative lipoprotein* | No homolog |
| BCAM1706 | 2267 | 1E-02 | BCAM1706 | hypothetical protein | Bcen2424_4524 |
| BCAM1767 | 4131 | 3E-02 | BCAM1767 | putative regulatory protein | No homolog |
| BCAM1777A | 2909 | 1E-02 | BCAM1777A | hypothetical protein* | Bcen2424_4597 |
| BCAM1799 | 4367 | 4E-02 | BCAM1799 | hypothetical protein | No homolog |
| BCAM1800 | 2651 | 7E-02 | BCAM1800 | hypothetical protein | No homolog |
| BCAM1811 | 2713 | 2E-02 | BCAM1811 | hypothetical protein | No homolog |
| BCAM1834 | 6762 | 2E-01 | BCAM1834 | hypothetical protein | No homolog |
| BCAM1836 | 4521 | 8E-02 | BCAM1836 | hypothetical protein | No homolog |
| BCAM1857 | 7878 | 3E-02 | BCAM1857 | hypothetical protein | No homolog |
| BCAM1875 | 609 | 7E-02 | BCAM1875 | hypothetical protein | No homolog |
| BCAM1876A | 3553 | 7E-02 | BCAM1876A | hypothetical protein | No homolog |
| BCAM1880 | 2775 | 2E-02 | BCAM1880 | hypothetical protein | No homolog |
| BCAM1882 | 8077 | 6E-02 | BCAM1882 | hypothetical protein | No homolog |
| BCAM1884 | 2483 | 9E-02 | BCAM1884 | putative DNA-binding phage protein | No homolog |
| BCAM1907 | 3606 | 8E-02 | BCAM1907 | hypothetical protein | No homolog |
| BCAM1910 | 2178 | 1E-03 | BCAM1910 | hypothetical protein | No homolog |
| BCAM1919 | 4656 | 3E-02 | BCAM1919 | hypothetical protein | No homolog |
| BCAM1920 | 1613 | 1E-03 | BCAM1920 | hypothetical protein | No homolog |
| BCAM1921 | 2467 | 6E-02 | BCAM1921 | putative phage membrane protein | No homolog |
| BCAM1922 | 2354 | 2E-02 | BCAM1922 | putative DNA-binding phage protein | No homolog |
| BCAM1923 | 1997 | 4E-02 | BCAM1923 | No gene annotation | No homolog |
| BCAM1906 | 1624 | 5E-02 | Multiple hits | No gene annotation | No homolog |
| BCAM1925 | 3524 | 1E-02 | Multiple hits | No gene annotation | No homolog |
| BCAM1927 | 2336 | 7E-02 | BCAM1927 | hypothetical protein* | Bcen2424_4733 |
| BCAM1929 | 804 | 5E-02 | Multiple hits | No gene annotation | No homolog |
| BCAM1930 | 4768 | 9E-02 | Multiple hits | No gene annotation | No homolog |
| BCAM1953 | 5233 | 2E-02 | Multiple hits | No gene annotation | No homolog |
| BCAM1980 | 586 | 2E-00 | BCAM1980 | hypothetical protein | No homolog |
| BCAM2006a_J1 | 2378 | 3E-02 | BCAM2006a | No gene annotation | No homolog |
| BCAM2068 | 5096 | 5E-01 | BCAM2068 | hypothetical protein | No homolog |
| BCAM2073 | 2465 | 4E-02 | BCAM2073 | hypothetical protein* | Bcen2424_4880 |
| BCAM2112a | 2966 | 5E-02 | BCAM2112a | No gene annotation | No homolog |
| BCAM2130 | 4071 | 9E-02 | BCAM2130 | 3-hydroxyanthranilate 3,4-dioxygenase | No homolog |
| BCAM2143 | 1364 | 7E-02 | BCAM2143 | cable pilus associated adhesin protein | No homolog |
| BCAM2177 | 7661 | 2E-02 | BCAM2177 | hypothetical protein* | No homolog |
| BCAM2205 | 2334 | 3E-02 | BCAM2205 | hypothetical protein | No homolog |
| BCAM2244 | 2525 | 2E-02 | BCAM2244 | hypothetical protein* | No homolog |
| BCAM2252_J0 | 5868 | 1E-02 | BCAM2252 | No gene annotation | No homolog |
| BCAM2252_J1 | 2121 | 2E-02 | BCAM2252 | No gene annotation | No homolog |
| BCAM2274a | 107 | 9E-02 | BCAM2274a | hypothetical protein | No homolog |
| BCAM2287 | 7102 | 5E-02 | BCAM2287 | hypothetical protein | Bcen2424_5044 |
| BCAM2356 | 8721 | 4E-02 | BCAM2356 | hypothetical protein | Bcen2424_5113 |
| BCAM2359A | 2067 | 1E-02 | BCAM2359A | putative DNA-binding protein | No homolog |
| BCAM2400a | 2656 | 4E-02 | BCAM2400a | hypothetical protein* | Bcen2424_5161 |
| BCAM2402 | 2328 | 1E-01 | BCAM2402 | hypothetical protein | Bcen2424_5164 |
| BCAM2414 | 2616 | 4E-02 | BCAM2414 | hypothetical protein | Bcen2424_5176 |
| BCAM2417 | 3171 | 2E-02 | BCAM2417 | hypothetical protein | No homolog |
| BCAM2422 | 2397 | 2E-01 | BCAM2422 | hypothetical protein* | No homolog |
| BCAM2444 | 4625 | 2E-02 | BCAM2444 | hypothetical protein* | No homolog |
| BCAM2475 | 433 | 3E-02 | BCAM2475 | hypothetical protein* | Bcen2424_5277 |
| BCAM2486 | 3549 | 2E-02 | BCAM2486 | hypothetical protein* | Bcen2424_5288 |
| BCAM2513 | 2077 | 4E-02 | BCAM2513 | hypothetical protein | Bcen2424_5318 |
| BCAM2535 | 3734 | 6E-02 | BCAM2535 | hypothetical protein | No homolog |
| BCAM2547a | 110 | 2E-02 | BCAM2547a | glyoxalase/bleomycin resistance protein/dioxygenase superfamily protein | No homolog |
| BCAM2555 | 3126 | 1E-03 | BCAM2555 | hypothetical protein* | No homolog |
| BCAM2609 | 2492 | 1E-02 | BCAM2609 | hypothetical protein* | Bcen2424_5415 |
| BCAM2615 | 2256 | 1E-02 | BCAM2615 | hypothetical protein | Bcen2424_5421 |
| BCAM2624 | 2112 | 4E-02 | BCAM2624 | hypothetical protein | Bcen2424_5429 |
| BCAM2679 | 2423 | 5E-02 | BCAM2679 | hypothetical protein | No homolog |
| BCAM2685 | 2927 | 1E-02 | BCAM2685 | hypothetical protein | Bcen2424_5496 |
| BCAM2686 | 3945 | 1E-02 | BCAM2686 | hypothetical protein | No homolog |
| BCAM2713 | 4481 | 3E-02 | BCAM2713 | hypothetical protein* | No homolog |
| BCAM2714 | 1045 | 9E-02 | Multiple hits | No gene annotation | No homolog |
| BCAM2759 | 3869 | 8E-01 | BCAM2759 | putative minor pilin and initiator* | No homolog |
| BCAM2762 | 1617 | 3E-02 | BCAM2762 | giant cable pilus chaperone protein* | No homolog |
| BCAM2766 | 2705 | 3E-02 | BCAM2766 | hypothetical protein* | No homolog |
| BCAM2811 | 2062 | 3E-01 | BCAM2811 | hypothetical protein | No homolog |
| BCAMr0727 | 2557 | 6E-02 | BCAMr0727 | tRNA-OTHER | Bcen2424_R0074 |
| BCAS0009 | 6948 | 5E-02 | BCAS0009 | hypothetical protein | No homolog |
| BCAS0012 | 3065 | 2E-02 | BCAS0012 | putative NifQ protein | No homolog |
| BCAM2636 | 1511 | 1E-02 | Multiple hits | No gene annotation | No homolog |
| BCAS0076 | 3043 | 4E-02 | BCAS0076 | hypothetical protein | No homolog |
| BCAS0151 | 3196 | 3E-02 | BCAS0151 | hypothetical protein* | No homolog |
| BCAS0174 | 1051 | 1E-02 | BCAS0174 | hypothetical protein | No homolog |
| BCAS0180 | 2671 | 5E-00 | BCAS0180 | No gene annotation | No homolog |
| BCAS0182 | 2062 | 4E-02 | BCAS0182 | hypothetical protein | No homolog |
| BCAS0202 | 3301 | 3E-02 | BCAS0202 | hypothetical protein | Bcen2424_6022 |
| BCAS0205 | 4073 | 1E-02 | BCAS0205 | TauD/TfdA taurine catabolism dioxygenase family protein* | Bcen2424_6019 |
| BCAS0213 | 3845 | 2E-02 | BCAS0213 | hypothetical protein | Bcen2424_6011 |
| BCAS0214 | 9313 | 1E-02 | BCAS0214 | hypothetical protein | Bcen2424_6010 |
| BCAS0215 | 2819 | 4E-02 | BCAS0215 | hypothetical protein | Bcen2424_6009 |
| BCAS0216 | 6727 | 3E-02 | BCAS0216 | putative acyl carrier protein | Bcen2424_6008 |
| BCAS0217 | 3108 | 3E-02 | BCAS0217 | hypothetical protein | No homolog |
| BCAS0218 | 1472 | 1E-22 | BCAS0218 | hypothetical protein | Bcen2424_6006 |
| BCAS0219 | 4517 | 2E-02 | BCAS0219 | hypothetical protein* | Bcen2424_6005 |
| BCAS0221 | 1673 | 9E-01 | BCAS0221 | No gene annotation | Bcen2424_6003 |
| BCAS0244 | 7742 | 5E-01 | BCAS0244 | hypothetical protein | No homolog |
| BCAS0246 | 3841 | 7E-01 | BCAS0246 | hypothetical protein | Bcen2424_5957 |
| BCAS0247 | 2473 | 1E-02 | BCAS0247 | hypothetical protein | Bcen2424_5956 |
| BCAS0257 | 3532 | 5E-02 | BCAS0257 | putative acetyltransferase | No homolog |
| BCAS0260 | 51 | 7E-02 | BCAS0260 | hypothetical protein | Bcen2424_5941 |
| BCAS0406 | 6982 | 5E-02 | BCAS0406 | hypothetical protein* | No homolog |
| BCAS0407 | 6626 | 1E-02 | BCAS0407 | hypothetical protein | Bcen2424_6621 |
| BCAS0410 | 5594 | 5E-02 | BCAS0410 | hypothetical protein | No homolog |
| BCAS0411 | 2668 | 5E-02 | BCAS0411 | hypothetical protein | No homolog |
| BCAS0412 | 3158 | 1E-02 | BCAS0412 | hypothetical protein | No homolog |
| BCAM2606 | 8422 | 5E-02 | Multiple hits | No gene annotation | Bcen2424_3472 |
| BCAS0068 | 926 | 1E-03 | Multiple hits | No gene annotation | Bcen2424_3472 |
| BCAS0433 | 2113 | 2E-02 | BCAS0433 | putative DNA-binding protein | Bcen2424_6581 |
| BCAS0505a | 7242 | 5E-02 | BCAS0505a | hypothetical protein | No homolog |
| BCAS0511 | 2238 | 7E-02 | BCAS0511 | putative phage tail protein gpX | No homolog |
| BCAS0515 | 8458 | 8E-02 | BCAS0515 | putative Lambda G-pre-tape measure frameshift protein | No homolog |
| BCAS0516 | 1296 | 1E-03 | BCAS0516 | hypothetical protein | No homolog |
| BCAS0519 | 1093 | 7E-01 | BCAS0519 | hypothetical protein | No homolog |
| BCAS0520 | 912 | 3E-02 | BCAS0520 | hypothetical protein | No homolog |
| BCAS0522 | 2224 | 5E-02 | BCAS0522 | hypothetical protein | No homolog |
| BCAS0523 | 2875 | 7E-02 | BCAS0523 | hypothetical protein | No homolog |
| BCAS0524 | 6484 | 3E-02 | BCAS0524 | hypothetical protein | No homolog |
| BCAS0529 | 2329 | 2E-02 | BCAS0529 | hypothetical protein TerS | No homolog |
| BCAS0530 | 3688 | 2E-02 | BCAS0530 | hypothetical protein | No homolog |
| BCAS0531 | 2574 | 3E-02 | BCAS0531 | putative phage membrane protein | No homolog |
| BCAS0532 | 3845 | 8E-01 | Multiple hits | No gene annotation | No homolog |
| BCAS0534 | 1149 | 2E-02 | BCAS0534 | holin | No homolog |
| BCAS0535 | 1357 | 3E-02 | BCAS0535 | hypothetical protein | No homolog |
| BCAS0536 | 6611 | 1E-02 | BCAS0536 | putative phage membrane protein | No homolog |
| BCAS0537 | 1245 | 7E-02 | BCAS0537 | hypothetical protein* | No homolog |
| BCAS0538 | 1668 | 4E-02 | BCAS0538 | putative phage membrane protein | No homolog |
| BCAS0539 | 7242 | 2E-02 | BCAS0539 | cro/cI repressor transcription regulator | No homolog |
| IG3_601100 | 4752 | 2E-02 | BCAS0540a | hypothetical protein | No homolog |
| BCAS0541 | 447 | 1E-02 | Multiple hits | No gene annotation | No homolog |
| BCAS0544 | 2506 | 5E-02 | BCAS0544 | hypothetical protein | No homolog |
| BCAS0545 | 2181 | 6E-02 | BCAS0545 | hypothetical protein | No homolog |
| BCAS0546 | 2578 | 5E-00 | BCAS0546 | Tn552/IS1604 rve transposase | No homolog |
| BCAS0549 | 7193 | 2E-02 | BCAS0549 | hypothetical protein | No homolog |
| BCAS0550 | 1545 | 7E-02 | BCAS0550 | hypothetical protein | No homolog |
| BCAS0561 | 2105 | 3E-02 | BCAS0561 | hypothetical protein | No homolog |
| BCAS0579 | 3401 | 8E-02 | BCAS0579 | hypothetical protein* | Bcen2424_6329 |
| BCAM1776 | 5215 | 3E-02 | Multiple hits | No gene annotation | No homolog |
| BCAS0611 | 3842 | 2E-02 | Multiple hits | No gene annotation | No homolog |
| BCAS0624 | 9767 | 5E-02 | BCAS0624 | hypothetical protein | No homolog |
| BCAS0629 | 3277 | 3E-02 | BCAS0629 | putative lipoprotein* | Bcen2424_6258 |
| BCAS0642_J0 | 5232 | 1E-02 | BCAS0642 | No gene annotation | No homolog |
| BCAS0642_J1 | 5798 | 2E-02 | BCAS0642 | No gene annotation | No homolog |
| BCAS0646_J0 | 4871 | 1E-02 | BCAS0646 | No gene annotation | No homolog |
| BCAS0646A | 3492 | 7E-02 | BCAS0646A | putative DNA-binding protein | No homolog |
| BCAS0648 | 2169 | 6E-02 | BCAS0648 | hypothetical protein | No homolog |
| BCAS0649 | 1216 | 2E-02 | BCAS0649 | hypothetical protein | No homolog |
| BCAM0522 | 1691 | 2E-02 | Multiple hits | No gene annotation | No homolog |
| BCAM0521 | 1518 | 8E-02 | Multiple hits | No gene annotation | No homolog |
| BCAS0650 | 2499 | 2E-02 | Multiple hits | No gene annotation | No homolog |
| BCAS0650a | 7325 | 1E-24 | Multiple hits | No gene annotation | No homolog |
| BCAS0656 | 165 | 6E-02 | Multiple hits | No gene annotation | No homolog |
| BCAS0657 | 3319 | 1E-02 | BCAS0657 | No gene annotation | No homolog |
| BCAS0660C | 2592 | 2E-02 | BCAS0660C | hypothetical protein | No homolog |
| BCAS0661A | 6253 | 2E-02 | BCAS0661A | hypothetical protein | No homolog |
| BCAS0662 | 3169 | 2E-02 | BCAS0662 | hypothetical protein | No homolog |
| BCAS0665 | 8588 | 1E-02 | BCAS0665 | hypothetical protein | No homolog |
| BCAS0669 | 5284 | 4E-02 | BCAS0669 | hypothetical protein | No homolog |
| BCAS0670 | 3944 | 6E-02 | BCAS0670 | hypothetical protein | No homolog |
| BCAS0671 | 1112 | 1E-00 | BCAS0671 | hypothetical protein | No homolog |
| BCAS0672 | 1102 | 3E-02 | BCAS0672 | hypothetical protein | No homolog |
| BCAS0673 | 1301 | 2E-02 | BCAS0673 | hypothetical protein | No homolog |
| BCAS0674 | 7109 | 3E-02 | BCAS0674 | hypothetical protein | No homolog |
| BCAS0675 | 9003 | 2E-02 | BCAS0675 | hypothetical protein | No homolog |
| BCAS0676 | 1379 | 1E-02 | BCAS0676 | hypothetical protein | No homolog |
| IG3_736569 | 2214 | 1E-02 | BCAS0676 | hypothetical protein | No homolog |
| BCAS0677 | 4103 | 1E-02 | BCAS0677 | hypothetical protein | No homolog |
| BCAS0678 | 776 | 6E-02 | BCAS0678 | hypothetical protein | No homolog |
| BCAS0679A | 2434 | 5E-02 | BCAS0679A | conserved hypothetical IS element protein | No homolog |
| BCAS0686 | 1237 | 1E-01 | BCAS0686 | hypothetical protein | No homolog |
| BCAS0687 | 236 | 1E-02 | BCAS0687 | hypothetical protein | No homolog |
| BCAS0720 | 25 | 5E-02 | BCAS0720 | No gene annotation | No homolog |
| BCAS0721 | 9377 | 2E-02 | BCAS0721 | hypothetical protein | No homolog |
| BCAS0742 | 3562 | 2E-02 | BCAS0742 | hypothetical protein | No homolog |
| BCAS0743 | 2033 | 5E-02 | BCAS0743 | putative acetyltransferase-GNAT family* | Bcen2424_6226 |
| BCALr0409c | 2398 | 1E-02 | Multiple hits | No gene annotation | Multiple hits |
| BCALr1480 | 2722 | 7E-02 | Multiple hits | No gene annotation | Multiple hits |
| BCAL0044 | 215 | 3E-02 | Multiple hits | No gene annotation | No homolog |
| BCAL0050 | 1074 | 1E-02 | Multiple hits | No gene annotation | No homolog |
| BCAL0082 | 2096 | 4E-02 | Multiple hits | No gene annotation | No homolog |
| BCAL0239 | 209 | 1E-02 | Multiple hits | No gene annotation | Bcen2424_0353 |
| BCAL0393 | 1659 | 3E-02 | Multiple hits | No gene annotation | No homolog |
| BCAL0410 | 2171 | 5E-01 | Multiple hits | No gene annotation | No homolog |
| BCAL0573_J0 | 2014 | 4E-02 | Multiple hits | No gene annotation | No homolog |
| BCAL0573_J1 | 2905 | 3E-02 | Multiple hits | No gene annotation | No homolog |
| BCAL0591 | 3999 | 2E-02 | Multiple hits | No gene annotation | No homolog |
| BCAL0969 | 3668 | 3E-02 | Multiple hits | No gene annotation | Bcen2424_1097 |
| BCAL0970 | 201 | 7E-02 | Multiple hits | No gene annotation | Bcen2424_1098 |
| BCAL0972 | 3156 | 7E-02 | Multiple hits | No gene annotation | Bcen2424_1100 |
| BCAL0977 | 2811 | 6E-18 | Multiple hits | No gene annotation | Bcen2424_1105 |
| BCAL0981 | 2401 | 1E-02 | Multiple hits | No gene annotation | Bcen2424_1109 |
| BCAL0983 | 2672 | 4E-02 | Multiple hits | No gene annotation | Bcen2424_1112 |
| BCAL0986 | 4049 | 3E-02 | Multiple hits | No gene annotation | Bcen2424_1115 |
| BCAL0989 | 4713 | 3E-02 | Multiple hits | No gene annotation | Bcen2424_1118 |
| BCAL0990 | 3296 | 2E-02 | Multiple hits | No gene annotation | Bcen2424_1119 |
| BCAL0996 | 3927 | 4E-02 | Multiple hits | No gene annotation | Bcen2424_1125 |
| BCAL1001 | 2675 | 3E-02 | Multiple hits | No gene annotation | Bcen2424_1130 |
| BCAL1002 | 7347 | 4E-01 | Multiple hits | No gene annotation | Bcen2424_1131 |
| BCAL1004 | 2211 | 6E-02 | Multiple hits | No gene annotation | Bcen2424_1133 |
| BCAL1006 | 2179 | 1E-02 | Multiple hits | No gene annotation | Bcen2424_1135 |
| BCAL1008 | 3852 | 1E-02 | Multiple hits | No gene annotation | No homolog |
| BCAL1014_J0 | 5044 | 1E-01 | Multiple hits | No gene annotation | No homolog |
| BCAL1019 | 49 | 1E-02 | Multiple hits | No gene annotation | Bcen2424_1144 |
| BCAL1021 | 3025 | 8E-02 | Multiple hits | No gene annotation | No homolog |
| BCAL1025 | 1597 | 3E-02 | Multiple hits | No gene annotation | No homolog |
| BCAL1028_J0_1 | 1426 | 8E-02 | Multiple hits | No gene annotation | Bcen2424_3472 |
| BCAL1028_J1 | 109 | 8E-01 | Multiple hits | No gene annotation | No homolog |
| BCAL1066 | 771 | 2E-02 | Multiple hits | No gene annotation | No homolog |
| BCAL1067 | 3055 | 1E-02 | Multiple hits | No gene annotation | No homolog |
| BCAL1141 | 3123 | 8E-02 | Multiple hits | No gene annotation | No homolog |
| BCAL1142 | 6468 | 1E-02 | Multiple hits | No gene annotation | No homolog |
| BCAL1163 | 473 | 1E-03 | Multiple hits | No gene annotation | No homolog |
| BCAL1164 | 3476 | 8E-02 | Multiple hits | No gene annotation | No homolog |
| BCAL1174 | 8684 | 5E-02 | Multiple hits | No gene annotation | No homolog |
| BCAL2218 | 2603 | 6E-01 | Multiple hits | No gene annotation | No homolog |
| BCAL2273 | 4785 | 7E-02 | Multiple hits | No gene annotation | No homolog |
| BCAL2275 | 4108 | 3E-02 | Multiple hits | No gene annotation | No homolog |
| BCAL2479 | 2011 | 2E-02 | Multiple hits | No gene annotation | No homolog |
| BCAL2497_J0_1 | 2479 | 2E-02 | Multiple hits | No gene annotation | No homolog |
| BCAL2497_J1 | 8608 | 3E-02 | Multiple hits | No gene annotation | No homolog |
| BCAL2581_J0 | 5017 | 1E-01 | Multiple hits | No gene annotation | No homolog |
| BCAL2581_J1 | 1221 | 5E-02 | Multiple hits | No gene annotation | No homolog |
| BCAL2583 | 3714 | 1E-02 | Multiple hits | No gene annotation | No homolog |
| BCAL2755 | 4128 | 5E-02 | Multiple hits | No gene annotation | No homolog |
| AU1054_G5650 | 2958 | 1E-02 | No homolog | No gene annotation | No homolog |
| BCAL0137 | 4566 | 4E-02 | No homolog | No gene annotation | No homolog |
| BCAL0181 | 2613 | 7E-02 | No homolog | No gene annotation | No homolog |
| BCAL0335 | 3637 | 7E-02 | No homolog | No gene annotation | No homolog |
| BCAL0359 | 3618 | 2E-02 | No homolog | No gene annotation | No homolog |
| BCAL0414_J1 | 604 | 3E-02 | No homolog | No gene annotation | No homolog |
| BCAL1132 | 2989 | 6E-02 | No homolog | No gene annotation | No homolog |
| BCAL1136 | 5531 | 3E-01 | No homolog | No gene annotation | No homolog |
| BCAL1137 | 324 | 5E-00 | No homolog | No gene annotation | No homolog |
| BCAL1138 | 3246 | 1E-02 | No homolog | No gene annotation | No homolog |
| BCAL1607 | 3976 | 4E-02 | No homolog | No gene annotation | No homolog |
| BCAL1681 | 4311 | 2E-01 | No homolog | No gene annotation | No homolog |
| BCAL1714 | 2416 | 7E-02 | No homolog | No gene annotation | No homolog |
| BCAL2480 | 2046 | 2E-02 | No homolog | No gene annotation | No homolog |
| BCAL2480b | 1202 | 5E-02 | No homolog | No gene annotation | No homolog |
| BCAL2533 | 2634 | 4E-02 | No homolog | No gene annotation | No homolog |
| BCAL2546a | 3804 | 9E-02 | No homolog | No gene annotation |  |
| BCAL2547a | 2804 | 2E-01 | No homolog | No gene annotation |  |
| BCAL2598 | 1056 | 1E-02 | No homolog | No gene annotation | No homolog |
| BCAL2962b | 2383 | 2E-02 | No homolog | No gene annotation | No homolog |
| BCAL2965b | 8487 | 3E-02 | No homolog | No gene annotation | No homolog |
| BCAL2966a | 4854 | 1E-02 | No homolog | No gene annotation | No homolog |
| BCAL3022 | 5753 | 3E-02 | No homolog | No gene annotation | No homolog |
| BCAL3078_J1 | 4022 | 3E-02 | No homolog | No gene annotation | No homolog |
| BCAL3248_J1 | 2482 | 5E-02 | No homolog | No gene annotation | No homolog |
| BCALr2231b | 1936 | 3E-02 | No homolog | No gene annotation | No homolog |
| BCAM0065A | 334 | 2E-02 | No homolog | No gene annotation | No homolog |
| BCAM0245_J0 | 7916 | 1E-02 | No homolog | No gene annotation | No homolog |
| BCAM0245_J1 | 2814 | 7E-02 | No homolog | No gene annotation | No homolog |
| BCAM0277 | 2264 | 1E-02 | No homolog | No gene annotation | No homolog |
| BCAM0412a | 164 | 9E-02 | No homolog | No gene annotation | No homolog |
| BCAM0458 | 2589 | 5E-01 | No homolog | No gene annotation | No homolog |
| BCAM0520 | 2475 | 4E-02 | No homolog | No gene annotation | No homolog |
| BCAM0856_J0 | 7881 | 1E-01 | No homolog | No gene annotation | Bcen2424_3820 |
| BCAM0928 | 861 | 3E-02 | No homolog | No gene annotation | No homolog |
| BCAM1203 | 3721 | 3E-02 | No homolog | No gene annotation | No homolog |
| BCAM1281 | 234 | 1E-02 | No homolog | No gene annotation | No homolog |
| BCAM1316b | 4272 | 2E-02 | No homolog | No gene annotation | No homolog |
| BCAM1837 | 1166 | 2E-02 | No homolog | No gene annotation | No homolog |
| BCAM1838 | 7816 | 7E-02 | No homolog | No gene annotation | No homolog |
| BCAM1874 | 2394 | 3E-02 | No homolog | No gene annotation | Bcen2424_4731 |
| BCAM2400b | 4015 | 4E-02 | No homolog | No gene annotation | No homolog |
| BCAM2457 | 1429 | 8E-01 | No homolog | No gene annotation | No homolog |
| BCAM2544 | 4129 | 2E-02 | No homolog | No gene annotation | No homolog |
| BCAS0050 | 2098 | 6E-02 | No homolog | No gene annotation | No homolog |
| BCAS0069 | 3885 | 2E-02 | No homolog | No gene annotation | No homolog |
| BCAS0080 | 2042 | 3E-02 | No homolog | No gene annotation | No homolog |
| BCAS0193 | 2049 | 2E-02 | No homolog | No gene annotation | Bcen2424_6032 |
| BCAS0200 | 2588 | 8E-02 | No homolog | No gene annotation | No homolog |
| BCAS0224 | 2293 | 1E-02 | No homolog | No gene annotation | No homolog |
| BCAS0255 | 2126 | 6E-01 | No homolog | No gene annotation | No homolog |
| BCAS0499 | 22 | 6E-02 | No homolog | No gene annotation | No homolog |
| BCAS0542 | 2231 | 1E-02 | No homolog | No gene annotation | No homolog |
| BCAS0651 | 21 | 7E-02 | No homolog | No gene annotation | No homolog |
| BCAS0655 | 5753 | 4E-02 | No homolog | No gene annotation | No homolog |
| BCAS0661B | 2625 | 1E-01 | No homolog | No gene annotation | No homolog |
| BCAS0681 | 2388 | 4E-02 | No homolog | No gene annotation | No homolog |
| BCAS0684_J0 | 297 | 1E-01 | No homolog | No gene annotation | No homolog |
| BCAS0749 | 1552 | 5E-02 | No homolog | No gene annotation | No homolog |
| BCAL1135 (BCAL1132) | 2678 | 3E-02 | Multiple genes | No gene annotation |  |
| BCAL2536 (BCAL2535a) | 1078 | 8E-02 | Multiple genes | No gene annotation |  |
| BCAL2594 (BCAL2591) | 2214 | 1E-01 | Multiple genes | No gene annotation |  |
| BCALr0217d (23 rRNA) | 3065 | 1E-02 | Multiple genes | No gene annotation |  |
| BCAM0787 (BCAM0788) | 1528 | 1E-02 | Multiple genes | No gene annotation |  |
| BCAM1905 (BCAM1906) | 1746 | 4E-02 | Multiple genes | No gene annotation |  |
| BCAM1917 (BCAM1914) | 2675 | 5E-02 | Multiple genes | No gene annotation |  |
| BCAM1924 (multiple matches) | 146 | 3E-02 | Multiple genes | No gene annotation |  |
| BCAM2104 (BCAM2101) | 4997 | 3E-02 | Multiple genes | No gene annotation |  |
| BCAS0683 (BCAS0681) | 3395 | 8E-02 | Multiple genes | No gene annotation |  |
| BCASr0743a (several 5S rRNA) | 1385 | 3E-02 | Multiple genes | No gene annotation |  |
| IG1_1277099 | 264 | 3E-02 | BCAL1169 | No gene annotation* | No homolog |
| IG1_1303825 | 252 | 2E-02 | BCAL1203 | hypothetical protein | No homolog |
| IG1_1392663 | 212 | 9E-02 | BCAL1279 | hypothetical protein* | No homolog |
| IG1_3414831 | 1095 | 1E-02 | BCAL3125 | No gene annotation | No homolog |
| IG2_1375301 | 1049 | 1E-01 | BCAM1251 | No gene annotation | Bcen2424_4127 |
| pBCA011_J1 | 3696 | 7E-02 | pBCA011 | No gene annotation* | No homolog |
| pBCA012 | 2947 | 9E-02 | pBCA012 | hypothetical protein | No homolog |
| pBCA013 | 487 | 2E-02 | pBCA013 | hypothetical protein* | No homolog |
| pBCA014 | 2232 | 2E-02 | pBCA014 | hypothetical protein | No homolog |
| pBCA015 | 2736 | 7E-02 | pBCA015 | hypothetical protein | No homolog |
| pBCA016 | 3226 | 7E-02 | pBCA016 | hypothetical protein | No homolog |
| pBCA017 | 4401 | 2E-02 | pBCA017 | hypothetical protein | No homolog |
| pBCA018 | 1034 | 1E-03 | pBCA018 | hypothetical protein | No homolog |
| pBCA019 | 379 | 2E-02 | pBCA019 | hypothetical protein | No homolog |
| pBCA021 | 3502 | 8E-02 | pBCA021 | putative TraH conjugative transfer protein* | No homolog |
| pBCA023 | 2854 | 2E-02 | pBCA023 | hypothetical protein | No homolog |
| pBCA024 | 4553 | 2E-02 | pBCA024 | hypothetical protein | No homolog |
| pBCA026 | 4305 | 5E-02 | pBCA026 | hypothetical protein* | No homolog |
| pBCA027 | 578 | 2E-01 | pBCA027 | putative conjugative transfer protein* | No homolog |
| pBCA028 | 6617 | 2E-02 | pBCA028 | hypothetical protein* | No homolog |
| pBCA031 | 2273 | 6E-02 | pBCA031 | putative TraU conjugative transfer protein precursor* | No homolog |
| pBCA032 | 2453 | 5E-02 | pBCA032 | putative TraW conjugative transfer protein precursor* | No homolog |
| pBCA037a | 308 | 3E-02 | pBCA037a | hypothetical protein* | No homolog |
| pBCA038 | 3039 | 5E-02 | pBCA038 | hypothetical protein | No homolog |
| pBCA042 | 5802 | 2E-02 | pBCA042 | hypothetical protein* | No homolog |
| pBCA045 | 313 | 1E-02 | pBCA045 | hypothetical protein* | No homolog |
| pBCA046 | 2117 | 1E-02 | pBCA046 | putative TraE conjugative transfer protein | No homolog |
| pBCA050 | 9105 | 3E-02 | pBCA050 | hypothetical protein | No homolog |
| pBCA052 | 7517 | 4E-02 | pBCA052 | hypothetical protein* | No homolog |
| pBCA054 | 1293 | 3E-01 | pBCA054 | LuxR family regulatory protein | No homolog |
| pBCA056 | 9262 | 2E-02 | pBCA056 | hypothetical protein | No homolog |
| pBCA058 | 2144 | 1E-16 | pBCA058 | No gene annotation* | No homolog |
| pBCA060 | 1488 | 4E-02 | pBCA060 | hypothetical protein | No homolog |
| pBCA062 | 7918 | 8E-02 | pBCA062 | hypothetical protein | No homolog |
| pBCA064 | 2881 | 4E-02 | pBCA064 | hypothetical protein* | No homolog |
| pBCA066 | 2302 | 4E-02 | pBCA066 | hypothetical protein | No homolog |
| pBCA078 | 5956 | 8E-01 | pBCA078 | hypothetical protein | No homolog |
| pBCA084 | 9721 | 1E-02 | Multiple hits | No gene annotation | No homolog |
| pBCA085 | 3515 | 3E-02 | pBCA085 | No gene annotation | No homolog |
| pBCA087 | 4992 | 1E-02 | pBCA087 | NUDIX hydrolase family protein | No homolog |
| pBCA089 | 1462 | 5E-01 | pBCA089 | conseved hypothetical protein | No homolog |
| pBCA089a | 9044 | 2E-02 | pBCA089a | No gene annotation | No homolog |
| pBCA091 | 2115 | 2E-02 | pBCA091 | hypothetical protein | No homolog |
| pBCA048 | 8356 | 1E-02 | Multiple hits | No gene annotation | No homolog |

^*^ contains predicted Sec general secretory pathway signal sequence predicted by SignalP software
