# Supplementary material for: Identification of Potential Therapeutic Targets for Burkholderia cenocepacia by Comparative Transcriptomics
Source: PLoS One. 2010 Jan 15;5(1):e8724. doi: 10.1371/journal.pone.0008724 (PMC2806911; doi:10.1371/journal.pone.0008724)
Supplement: Table S4 — Probes showing at least a 2-fold increase in HI2424 versus J2315 under CF conditions. List of genes induced in the soil isolate HI2424 compared to the clinical isolate J2315 under CF-like conditions. (2.66 MB DOC) [file pone.0008724.s005.doc]

Table S4. Probes showing at least a 2-fold increase in HI2424 versus J2315 under CF conditions.

| **Probe ID** | **J2315/**  **HI2424 pixel intensity ratio** | **p-value** | **HI2424 Gene* (Bcen2424_)** | **Annotation** | **J2315 homolog*** |
| --- | --- | --- | --- | --- | --- |
|  |  |  |  | ***Translation, ribosomal structure and biogenesis (J)*** |  |
| HI2424_G6382 | 0.02 | 2E-05 | 0190 | hypothetical protein | No homolog |
| HI2424_G7009 | 0.07 | 1E-07 | 3444 | hypothetical protein | No homolog |
| AU1054_G2861 | 0.09 | 5E-06 | 6416 | tryptophanyl-tRNA synthetase | No homolog |
| BCAM2179 | 0.18 | 2E-08 | 4948 | ribonuclease T2 | BCAM2179 |
| BCAL1702 | 0.24 | 9E-04 | 1646 | hypothetical protein | BCAL1702 |
| BCAS0236 | 0.25 | 7E-05 | 5983 | YadA C-terminal domain protein | Multiple hits |
| AU1054_G2900 | 0.27 | 2E-11 | 6374 | Endoribonuclease L-PSP | No homolog |
| BCAL2715 | 0.31 | 3E-12 | 2503 | ribosomal protein L33 | BCAL2715 |
| BCAL0465 | 0.33 | 6E-12 | 3127 | peptide deformylase | BCAL0465 |
| BCAL0483 | 0.34 | 1E-10 | 3109 | glutamyl-tRNA(Gln) amidotransferase, C subunit | BCAL0483 |
| BCALr3349 | 0.40 | 2E-03 | R0022 | RNaseP RNA | BCALr3349 |
| BCAM2261 | 0.40 | 2E-03 | No homolog | No gene annotation | BCAM2261 |
| BCAL2189 | 0.43 | 7E-04 | 2117 | peptide chain release factor 2 | No homolog |
| BCAL2697 | 0.43 | 1E-07 | 2487 | Pseudouridine synthase, Rsu | BCAL2697 |
| BCAL2411 | 0.47 | 8E-13 | No homolog | No gene annotation | BCAL2411 |
| BCAL0373 | 0.48 | 5E-03 | 0509 | modification methylase, HemK family | BCAL0373 |
|  |  |  |  | ***Transcription (K)*** |  |
| AU1054_G2137 | 0.02 | 3E-05 | 6308 | transcriptional regulator, LacI family | No homolog |
| AU1054_G1164 | 0.02 | 1E-06 | 5481 | hypothetical protein | No homolog |
| AU1054_G551 | 0.03 | 4E-05 | 6600 | GntR domain protein | No homolog |
| AU1054_G6522 | 0.03 | 1E-05 | 5221 | putative transcriptional regulator, AsnC family | No homolog |
| HI2424_G4111 | 0.03 | 2E-06 | 6893 | putative transcriptional regulator | No homolog |
| AU1054_G2904 | 0.03 | 1E-06 | 6370 | transcriptional regulator, AraC family | No homolog |
| HI2424_G6265 | 0.03 | 3E-04 | 0156 | transcriptional regulator, GntR family | No homolog |
| HI2424_G6245 | 0.03 | 8E-07 | 0178 | transcriptional regulator, TetR family | No homolog |
| AU1054_G6280 | 0.03 | 4E-07 | 5208 | transcriptional regulator, AraC family | No homolog |
| HI2424_G4058 | 0.03 | 1E-04 | 6861 | transcriptional regulator, TetR family | No homolog |
| AU1054_G152 | 0.04 | 9E-07 | 5979 | transcriptional regulator, TetR family | No homolog |
| AU1054_G5227 | 0.04 | 6E-06 | 4397 | transcriptional regulator, XRE family | No homolog |
| AU1054_G2814 | 0.04 | 5E-06 | 6469 | transcriptional regulator, LysR family | No homolog |
| HI2424_G4082 | 0.04 | 2E-06 | 6891 | hypothetical protein | No homolog |
| AU1054_G2889 | 0.05 | 3E-04 | 6385 | transcriptional regulator, AraC family | No homolog |
| AU1054_G4959 | 0.05 | 9E-06 | 6042 | transcriptional regulator, TetR family | No homolog |
| HI2424_G4104 | 0.05 | 4E-07 | 6844 | transcriptional regulator, TetR family | No homolog |
| AU1054_G5718 | 0.06 | 1E-05 | 5379 | transcriptional regulator, LysR family | No homolog |
| AU1054_G2798 | 0.06 | 1E-06 | 2753 | hypothetical protein | No homolog |
| HI2424_G6269 | 0.06 | 2E-07 | 0152 | phage transcriptional regulator, AlpA | No homolog |
| AU1054_G1269 | 0.06 | 5E-07 | 4909 | TfoX, N-terminal domain protein | No homolog |
| AU1054_G5912 | 0.06 | 1E-07 | 5890 | transcriptional regulator, TetR family | No homolog |
| AU1054_G2883 | 0.07 | 3E-07 | 6391 | TfoX, N-terminal domain protein | No homolog |
| AU1054_G811 | 0.07 | 8E-06 | 1650 | putative transcriptional regulators, CopG/Arc/MetJ family | No homolog |
| AU1054_G2901 | 0.08 | 4E-06 | 6373 | transcriptional regulator, TrmB | No homolog |
| AU1054_G2442 | 0.08 | 8E-08 | 3219 | transcriptional regulator, AraC family | No homolog |
| AU1054_G1934 | 0.08 | 2E-07 | 6246 | transcriptional regulator, LysR family | No homolog |
| AU1054_G5975 | 0.08 | 2E-07 | 5930 | GCN5-related N-acetyltransferase | No homolog |
| AU1054_G2879 | 0.08 | 5E-04 | 6395 | transcriptional regulator, GntR family | No homolog |
| HI2424_G4074 | 0.09 | 2E-08 | 6881 | transcriptional regulator, XRE family | No homolog |
| AU1054_G1920 | 0.09 | 6E-07 | 6263 | transcriptional regulator, LysR family | No homolog |
| AU1054_G1125 | 0.09 | 9E-08 | 5440 | transcriptional regulator, LysR family | No homolog |
| AU1054_G5710 | 0.11 | 2E-06 | 5388 | transcriptional regulator, LysR family | No homolog |
| AU1054_G2905 | 0.11 | 9E-08 | 6369 | transcriptional regulator, LysR family | No homolog |
| AU1054_G2122 | 0.12 | 3E-06 | 6323 | transcriptional regulator, AraC family | No homolog |
| AU1054_G425 | 0.13 | 1E-08 | 3996 | transcriptional regulator, HxlR family | No homolog |
| AU1054_G2898 | 0.14 | 9E-09 | 6376 | transcriptional regulator, AraC family | No homolog |
| AU1054_G2512 | 0.14 | 3E-07 | 4183 | transcriptional regulator, HxlR family | No homolog |
| AU1054_G2420 | 0.14 | 2E-09 | 3251 | transcriptional regulator, AraC family | No homolog |
| AU1054_G1958 | 0.15 | 1E-09 | 6298 | transcriptional regulator, DeoR family | No homolog |
| AU1054_G2437 | 0.15 | 5E-10 | 3226 | transcriptional regulator, LysR family | No homolog |
| AU1054_G3612 | 0.15 | 5E-06 | 4648 | transcriptional regulator, LysR family | No homolog |
| AU1054_G2376 | 0.16 | 5E-10 | 0224 | hypothetical protein | No homolog |
| BCAM2616 | 0.16 | 3E-10 | 5422 | transcriptional regulator, AraC family | BCAM2616 |
| AU1054_G1450 | 0.16 | 8E-07 | 6516 | helix-turn-helix, Fis-type | No homolog |
| AU1054_G2141 | 0.16 | 5E-10 | 6303 | hypothetical protein | No homolog |
| AU1054_G5529 | 0.16 | 8E-10 | 6631 | transcriptional regulator, HxlR family | No homolog |
| BCAM0815 | 0.17 | 3E-09 | 3780 | putative transcriptional regulator | BCAM0815 |
| AU1054_G2452 | 0.17 | 2E-09 | 3208 | transcriptional regulator, LysR family | No homolog |
| AU1054_G2895 | 0.17 | 1E-08 | 6379 | transcriptional regulator, LysR family | No homolog |
| BCAM0599 | 0.18 | 1E-09 | 3572 | transcriptional regulator, BadM/Rrf2 family | BCAM0599 |
| BCAM0189 | 0.18 | 4E-04 | 3181 | transcriptional regulator, AraC family | BCAM0189 |
| AU1054_G1321 | 0.18 | 1E-09 | 4768 | transcriptional regulator, LysR family | No homolog |
| AU1054_G2427 | 0.18 | 2E-07 | 3244 | transcriptional regulator, AraC family | No homolog |
| AU1054_G5537 | 0.19 | 4E-09 | 6639 | transcriptional regulator, GntR family | No homolog |
| AU1054_G2848 | 0.19 | 2E-09 | 6430 | transcriptional regulator, GntR family | No homolog |
| AU1054_G5528 | 0.19 | 1E-07 | 6630 | transcriptional regulator, LysR family | No homolog |
| BCAL2614 | 0.20 | 6E-08 | 2409 | transcriptional regulator, LysR family | BCAL2614 |
| AU1054_G5602 | 0.20 | 2E-06 | 6712 | transcriptional regulator, AraC family | No homolog |
| AU1054_G3527 | 0.20 | 4E-10 | 4559 | GCN5-related N-acetyltransferase | No homolog |
| AU1054_G1447 | 0.21 | 1E-10 | 6519 | transcriptional regulator, AraC family | No homolog |
| AU1054_G6529 | 0.21 | 2E-08 | 5189 | transcriptional regulator, LysR family | No homolog |
| AU1054_G4915 | 0.21 | 4E-06 | 5995 | transcriptional regulator, LysR family | No homolog |
| BCAM0038 | 0.23 | 4E-09 | 5729 | transcriptional regulator, AraC family | BCAM0038 |
| AU1054_G5712 | 0.24 | 8E-11 | 5385 | transcriptional regulator, LysR family | No homolog |
| BCAM1484 | 0.25 | 2E-03 | 4356 | response regulator receiver protein | BCAM1484 |
| BCAM2065 | 0.25 | 2E-05 | 4865 | transcriptional regulator, TetR family | BCAM2065 |
| AU1054_G3731 | 0.26 | 1E-10 | 0922 | hypothetical protein | No homolog |
| BCAM1858 | 0.26 | 5E-04 | 4715 | hypothetical protein | BCAM1858 |
| AU1054_G1304 | 0.26 | 1E-06 | 4750 | transcriptional regulator, TetR family | No homolog |
| BCAL2314 | 0.27 | 1E-10 | 2219 | two component transcriptional regulator, LuxR family | BCAL2314 |
| AU1054_G5070 | 0.27 | 1E-08 | 6160 | transcriptional regulator, GntR family | No homolog |
| AU1054_G6477 | 0.27 | 2E-10 | 5197 | transcriptional regulator, LysR family | No homolog |
| BCAS0382 | 0.27 | 3E-04 | 6686 | transcriptional regulator, AsnC family | BCAS0382 |
| BCAM1218 | 0.27 | 1E-11 | 4094 | transcriptional regulator, LysR family | BCAM1218 |
| AU1054_G5322 | 0.27 | 6E-06 | 4288 | transcriptional regulator, AraC family | No homolog |
| BCAM1807 | 0.27 | 7E-11 | 4668 | transcriptional regulator, LysR family | BCAM1807 |
| BCAM1752A | 0.28 | 8E-12 | 4575 | transcriptional regulator, LysR family | BCAM1752A |
| BCAL1403 | 0.28 | 3E-05 | No homolog | No gene annotation | BCAL1403 |
| BCAM1259 | 0.29 | 5E-11 | 4132 | sigma factor 70 SigJ | BCAM1259 |
| BCAM2777 | 0.29 | 1E-05 | 5621 | transcriptional regulator, LysR family | BCAM2777 |
| AU1054_G428 | 0.29 | 7E-08 | 3993 | transcriptional regulator, LuxR family | No homolog |
| AU1054_G4893 | 0.30 | 5E-05 | 3834 | GCN5-related N-acetyltransferase | No homolog |
| BCAM2708 | 0.30 | 8E-11 | 5523 | transcriptional regulator, IclR family | BCAM2708 |
| BCAM2246 | 0.31 | 1E-05 | 5014 | transcriptional regulator, LysR family | BCAM2246 |
| BCAL2671 | 0.32 | 1E-09 | 2461 | transcriptional regulator, LysR family | BCAL2671 |
| BCAM2306 | 0.33 | 2E-10 | 5064 | transcriptional regulator, AraC family | BCAM2306 |
| BCAL0685 | 0.33 | 2E-04 | 2913 | transcriptional regulator, IclR family | BCAL0685 |
| AU1054_G5554 | 0.34 | 3E-08 | 6661 | transcriptional regulator, LysR family | No homolog |
| BCAL0638 | 0.34 | 2E-05 | 2952 | transcriptional regulator, LysR family | BCAL0638 |
| BCAL1851 | 0.34 | 2E-04 | 1777 | transcriptional regulator, LysR family | BCAL1851 |
| BCAL0602 | 0.34 | 3E-06 | 2991 | transcriptional regulator, XRE family with cupin sensor | BCAL0602 |
| BCAM0481 | 0.35 | 1E-08 | 3448 | transcriptional regulator, AraC family | BCAM0481 |
| AU1054_G312 | 0.36 | 1E-10 | 5974 | transcriptional regulator, TetR family | No homolog |
| AU1054_G5600 | 0.36 | 1E-11 | 6710 | GntR domain protein | No homolog |
| BCAL3409 | 0.36 | 1E-03 | 0617 | transcriptional regulator, IclR family | BCAL3409 |
| BCAM2327 | 0.37 | 2E-12 | 5086 | transcriptional regulator, AraC family | BCAM2327 |
| AU1054_G6153 | 0.37 | 9E-12 | 3422 | transcriptional regulator, AraC family | No homolog |
| BCAM0133 | 0.37 | 1E-05 | No homolog | No gene annotation | BCAM0133 |
| BCAL0865 | 0.37 | 1E-11 | 2741 | transcriptional regulator, RpiR family | BCAL0865 |
| BCAS0062 | 0.38 | 4E-11 | 6146 | transcriptional regulator, LysR family | BCAS0062 |
| BCAM1722 | 0.39 | 4E-03 | No homolog | No gene annotation | BCAM1722 |
| BCAM2617 | 0.39 | 2E-05 | 5423 | transcriptional regulator, LysR family | BCAM2617 |
| AU1054_G3608 | 0.40 | 8E-09 | 4644 | transcriptional regulator, AsnC family | No homolog |
| BCAM2394 | 0.40 | 6E-04 | No homolog | No gene annotation | BCAM2394 |
| BCAS0770 | 0.40 | 3E-04 | No homolog | No gene annotation | BCAS0770 |
| BCAS0736 | 0.41 | 8E-04 | 6233 | transcriptional regulator, TetR family | BCAS0736 |
| BCAS0361 | 0.41 | 3E-02 | No homolog | No gene annotation | BCAS0361 |
| BCAL0787 | 0.41 | 1E-02 | 2815 | sigma factor 70 SigM | BCAL0787 |
| BCAM1380 | 0.41 | 1E-06 | No homolog | No gene annotation | BCAM1380 |
| BCAL1227 | 0.42 | 5E-12 | 1256 | transcriptional regulator, LysR family | BCAL1227 |
| BCAL1946 | 0.42 | 3E-04 | 1875 | putative transmembrane anti-sigma factor | BCAL1946 |
| BCAL1335 | 0.42 | 1E-12 | 1343 | transcriptional regulator, PadR-like family | BCAL1335 |
| BCAM1724 | 0.42 | 9E-05 | No homolog | No gene annotation | BCAM1724 |
| BCAL0941 | 0.43 | 6E-06 | 2661 | transcriptional regulator, LysR family | BCAL0941 |
| BCAM2802 | 0.43 | 9E-12 | No homolog | No gene annotation | BCAM2802 |
| BCAL2803 | 0.43 | 1E-12 | 2590 | transcriptional regulator, LysR family | BCAL2803 |
| BCAM2774 | 0.43 | 1E-04 | 5616 | transcriptional regulator, histidine utilization repressor, GntR family | BCAM2774 |
| BCAL2125 | 0.44 | 1E-05 | 2055 | GCN5-related N-acetyltransferase | BCAL2125 |
| BCAM2162 | 0.44 | 2E-04 | 4926 | transcriptional regulator, MarR family | BCAM2162 |
| BCAM2569 | 0.44 | 1E-03 | 5369 | transcriptional regulator, IclR family | BCAM2569 |
| BCAM2824 | 0.46 | 6E-12 | 5672 | two component transcriptional regulator, LuxR family | BCAM2824 |
| BCAM0134 | 0.46 | 1E-05 | 5834 | transcriptional regulator, LysR family | BCAM0134 |
| BCAM0575 | 0.47 | 8E-06 | 3549 | transcriptional regulator, LysR family | BCAM0575 |
| BCAM1862 | 0.47 | 4E-12 | 4718 | transcriptional regulator, LysR family | BCAM1862 |
| BCAM2805 | 0.47 | 2E-08 | 5655 | transcriptional regulator, LacI family | BCAM2805 |
| BCAM0702 | 0.47 | 6E-06 | 3669 | transcriptional regulator, LysR family | BCAM0702 |
| BCAM2654 | 0.47 | 3E-03 | 5458 | GCN5-related N-acetyltransferase | BCAM2654 |
| BCAM0358 | 0.48 | 2E-03 | 3342 | putative transcriptional regulator, ArsR family | BCAM0358 |
| BCAM0673 | 0.48 | 1E-03 | 3637 | transcriptional regulator, IclR family | BCAM0673 |
| BCAM0935 | 0.48 | 8E-10 | 3902 | transcriptional regulator, TetR family | BCAM0935 |
| BCAL1553 | 0.48 | 5E-08 | 1546 | ribonuclease R | BCAL1553 |
| BCAL1376 | 0.48 | 1E-03 | 1366 | transcriptional regulator, LysR family | BCAL1376 |
| BCAM2655 | 0.48 | 3E-04 | 5459 | transcriptional regulator, ArsR family | BCAM2655 |
| BCAM0403 | 0.48 | 1E-03 | 3390 | GCN5-related N-acetyltransferase | BCAM0403 |
| BCAL3510 | 0.49 | 1E-05 | 0044 | transcriptional regulator, LysR family | BCAL3510 |
| BCAL2800 | 0.49 | 1E-05 | 2587 | transcriptional regulator, DeoR family | BCAL2800 |
| BCAL3368 | 0.49 | 1E-06 | 0659 | transcriptional regulator, RpiR family | BCAL3368 |
| BCAS0323 | 0.49 | 8E-11 | 3283 | transcriptional regulator, LacI family | BCAS0323 |
| AU1054_G4972 | 0.49 | 1E-07 | 6056 | transcriptional regulator, LysR family | No homolog |
| BCAL0641 | 0.50 | 2E-12 | 2949 | transcriptional regulator, LysR family | BCAL0641 |
| BCAM0958 | 0.50 | 1E-03 | 3925 | transcriptional regulator, ArsR family | BCAM0958 |
| BCAM0977 | 0.50 | 1E-09 | 3944 | transcriptional regulator, AsnC family | BCAM0977 |
| HI2424_G6719 | > 0.01 | 1E-06 | 3481 | GCN5-related N-acetyltransferase | No homolog |
| HI2424_G195 | > 0.01 | 5E-05 | 6941 | putative transcriptional regulator | No homolog |
|  |  |  |  | ***Replication, recombination, and repair (L)*** |  |
| AU1054_G6083 | 0.02 | 1E-06 | 5567 | phage integrase family protein | No homolog |
| AU1054_G5206 | 0.02 | 5E-07 | 4422 | transposase, IS605 OrfB | No homolog |
| AU1054_G2863 | 0.03 | 8E-06 | 6413 | serine/threonine protein kinase | No homolog |
| AU1054_G1870 | 0.04 | 4E-05 | 0446 | phage integrase family protein | No homolog |
| HI2424_G4055 | 0.04 | 3E-06 | 6857 | transposase IS66 | No homolog |
| HI2424_G4061 | 0.05 | 2E-06 | 6864 | phage integrase family protein | No homolog |
| AU1054_G6524 | 0.06 | 4E-04 | 5218 | Integrase, catalytic region | No homolog |
| HI2424_G4083 | 0.06 | 7E-06 | 6892 | phage integrase family protein | No homolog |
| HI2424_G177 | 0.06 | 4E-07 | 6945 | TrwC protein | No homolog |
| AU1054_G4607 | 0.08 | 2E-07 | 1326 | phage integrase family protein | No homolog |
| HI2424_G6755 | 0.11 | 2E-08 | 3442 | Integrase, catalytic region | No homolog |
| AU1054_G3438 | 0.11 | 1E-07 | 2364 | phage integrase family protein | No homolog |
| HI2424_G6227 | 0.13 | 6E-10 | 0206 | ATP-dependent endonuclease of the OLD family-like protein | No homolog |
| AU1054_G2371 | 0.13 | 2E-05 | 0212 | Integrase, catalytic region | No homolog |
| AU1054_G4611 | 0.15 | 4E-10 | 1320 | phage terminase, small subunit, putative, P27 family | No homolog |
| HI2424_G6251 | 0.16 | 2E-09 | 0172 | transposase and inactivated derivatives-like protein | No homolog |
| HI2424_G4042 | 0.16 | 2E-09 | 6840 | transposase IS66 | No homolog |
| AU1054_G2432 | 0.17 | 2E-08 | 3234 | transposase IS3/IS911 family protein | No homolog |
| HI2424_G4065 | 0.18 | 4E-06 | 6869 | hypothetical protein | No homolog |
| HI2424_G4105 | 0.18 | 3E-10 | 6835 | phage integrase family protein | No homolog |
| AU1054_G439 | 0.19 | 1E-10 | 3999 | hypothetical protein | No homolog |
| HI2424_G6144 | 0.21 | 6E-11 | 6945 | TrwC protein | No homolog |
| HI2424_G4041 | 0.22 | 3E-04 | 6839 | IS66 Orf2 family protein | No homolog |
| AU1054_G2460 | 0.24 | 5E-04 | 3258 | Integrase, catalytic region | No homolog |
| AU1054_G540 | 0.26 | 9E-05 | 6605 | phage integrase family protein | No homolog |
| HI2424_G6226 | 0.29 | 2E-07 | 0207 | hypothetical protein | No homolog |
| AU1054_G3733 | 0.33 | 9E-09 | 0926 | phage integrase family protein | No homolog |
| HI2424_G4057 | 0.33 | 4E-10 | 6859 | transposase IS3/IS911 family protein | No homolog |
| BCAL0161 | 0.37 | 2E-03 | No homolog | No gene annotation | BCAL0161 |
| AU1054_G539 | 0.37 | 2E-10 | 6606 | transposase Tn3 family protein | No homolog |
| AU1054_G3045 | 0.37 | 1E-04 | 1569 | phage-related integrase | No homolog |
| BCAM2814 | 0.37 | 1E-11 | No homolog | No gene annotation | BCAM2814 |
| BCAL1899 | 0.39 | 3E-12 | 1827 | DNA polymerase III, subunits gamma and tau | BCAL1899 |
| pBCA006 | 0.39 | 2E-04 | 6829 | hypothetical protein | pBCA006 |
| HI2424_G4107 | 0.40 | 3E-08 | 6838 | transposase IS3/IS911 family protein | No homolog |
| BCAL1254 | 0.40 | 6E-12 | 1284 | DNA polymerase III, epsilon subunit | BCAL1254 |
| AU1054_G2399 | 0.41 | 1E-05 | 3279 | transposase, IS21 family | No homolog |
| BCAL2455 | 0.42 | 1E-03 | 2360 | DNA topoisomerase (ATP-hydrolyzing) | BCAL2455 |
| HI2424_G6229 | 0.46 | 4E-09 | 0204 | GP47 | No homolog |
| BCAL2621 | 0.46 | 1E-06 | 2416 | Excinuclease ABC, C subunit domain protein | BCAL2621 |
| BCAL0422 | 0.46 | 6E-03 | 0002 | DNA polymerase III, beta subunit | BCAL0422 |
| BCAL2675 | 0.48 | 2E-03 | 2465 | DNA polymerase III chi subunit, HolC | BCAL2675 |
| BCAM1690 | 0.49 | 3E-06 | 4512 | Uracil-DNA glycosylase superfamily | BCAM1690 |
| BCAL3530 | 0.50 | 2E-02 | 0065 | histone family protein DNA-binding protein | BCAL3530 |
|  |  |  |  | ***Cell cycle control, cell division, chromosome partitioning (D)*** |  |
| AU1054_G2294 | 0.06 | 2E-07 | 2642 | glycosyl hydrolase, BNR repeat-containing protein | No homolog |
| AU1054_G4213 | 0.13 | 7E-09 | 2948 | prevent-host-death family protein | No homolog |
| AU1054_G3637 | 0.16 | 1E-08 | 5502 | plasmid stabilization system | No homolog |
| BCAL0830 | 0.32 | 4E-11 | No homolog | No gene annotation | BCAL0830 |
| BCAL0678 | 0.37 | 1E-05 | 2920 | Sporulation domain protein | BCAL0678 |
|  |  |  |  | **Defense mechanisms (V)** |  |
| BCAM2226 | 0.24 | 4E-03 | 4995 | ABC transporter related | BCAM2226 |
| BCAL2044 | 0.28 | 2E-11 | 1967 | Muramoyltetrapeptide carboxypeptidase | BCAL2044 |
| AU1054_G1436 | 0.28 | 4E-09 | 6531 | acriflavin resistance protein | No homolog |
| BCAM0511 | 0.29 | 1E-02 | No homolog | No gene annotation | BCAM0511 |
| BCAL2185 | 0.37 | 7E-12 | 2113 | lipoprotein releasing system, ATP-binding protein | BCAL2185 |
| BCAM1946 | 0.38 | 2E-05 | 4745 | transporter, hydrophobe/amphiphile efflux-1 (HAE1) family | BCAM1946 |
| BCAL3430 | 0.40 | 5E-09 | 0593 | N-acetylmuramyl-L-alanine amidase, negative regulator of AmpC, AmpD | BCAL3430 |
| BCAM1864 | 0.45 | 2E-06 | 4720 | secretion protein HlyD family protein | BCAM1864 |
| BCAL3514 | 0.47 | 2E-12 | 0050 | RND efflux system, outer membrane lipoprotein, NodT family | BCAL3514 |
| BCAL1555 | 0.47 | 2E-03 | No homolog | No gene annotation | BCAL1555 |
| BCAL2907 | 0.48 | 2E-04 | 1091 | MATE efflux family protein | BCAL2907 |
| BCAL1647 | 0.49 | 2E-11 | 1596 | nodulation ABC transporter NodI | BCAL1647 |
| AU1054_G2374 | > 0.01 | 1E-06 | 0217 | N-6 DNA methylase | No homolog |
|  |  |  |  | ***Signal transduction mechanisms (T)*** |  |
| AU1054_G6293 | 0.04 | 5E-07 | 5187 | multi-sensor signal transduction histidine kinase | No homolog |
| AU1054_G4673 | 0.05 | 3E-07 | 1321 | RelA/SpoT domain protein | No homolog |
| HI2424_G6757 | 0.07 | 6E-06 | 3439 | two component transcriptional regulator, winged helix family | No homolog |
| HI2424_G7005 | 0.08 | 2E-07 | 3497 | hypothetical protein | No homolog |
| BCAL0809 | 0.14 | 8E-09 | 2794 | HPr kinase | BCAL0809 |
| AU1054_G5589 | 0.15 | 3E-08 | 6699 | methyl-accepting chemotaxis sensory transducer | No homolog |
| AU1054_G3478 | 0.16 | 2E-10 | 2373 | hypothetical protein | No homolog |
| AU1054_G4230 | 0.16 | 2E-05 | 3198 | histidine kinase | No homolog |
| AU1054_G6294 | 0.18 | 1E-09 | 5186 | two component transcriptional regulator, LuxR family | No homolog |
| AU1054_G1672 | 0.18 | 7E-11 | 1901 | diguanylate cyclase | No homolog |
| AU1054_G2828 | 0.19 | 7E-05 | 6454 | multi-sensor hybrid histidine kinase | No homolog |
| HI2424_G6267 | 0.20 | 2E-10 | 0154 | methyl-accepting chemotaxis sensory transducer | No homolog |
| BCAM2533 | 0.26 | 1E-09 | 5341 | periplasmic sensor signal transduction histidine kinase | BCAM2533 |
| BCAM1483 | 0.27 | 2E-03 | 4355 | transcriptional regulator, Crp/Fnr family | BCAM1483 |
| BCAL1975 | 0.37 | 3E-04 | 1903 | diguanylate cyclase | BCAL1975 |
| BCAL0430 | 0.37 | 6E-05 | 3160 | diguanylate cyclase | BCAL0430 |
| BCAM0091 | 0.38 | 2E-07 | No homolog | No gene annotation | BCAM0091 |
| BCAS0263 | 0.39 | 9E-05 | 5939 | response regulator receiver modulated metal dependent phosphohydrolase | BCAS0263 |
| BCAM0748 | 0.42 | 5E-09 | 3714 | diguanylate cyclase | BCAM0748 |
| BCAL0499 | 0.42 | 1E-06 | 3093 | response regulator receiver protein | BCAL0499 |
| AU1054_G2886 | 0.46 | 5E-03 | 6388 | UspA domain protein | No homolog |
| AU1054_G3589 | 0.47 | 4E-03 | 4624 | periplasmic sensor signal transduction histidine kinase | No homolog |
| BCAL2223 | 0.47 | 1E-02 | 2148 | signal transduction histidine kinase, nitrogen specific, NtrB | BCAL2223 |
| BCAM0049 | 0.48 | 4E-04 | 5737 | transcriptional regulator, Crp/Fnr family | BCAM0049 |
| BCAL0952 | 0.48 | 1E-12 | 2653 | two component transcriptional regulator, winged helix family | BCAL0952 |
| AU1054_G2816 | 0.49 | 7E-11 | 6466 | response regulator receiver protein | No homolog |
| BCAS0010 | 0.49 | 9E-06 | No homolog | No gene annotation | BCAS0010 |
| BCAL2380 | 0.50 | 4E-06 | 2285 | osmosensitive K+ channel signal transduction histidine kinase | BCAL2380 |
| AU1054_G2830 | > 0.01 | 1E-06 | 6451 | two component, sigma54 specific, transcriptional regulator, Fis family | No homolog |
|  |  |  |  | ***Cell wall/membrane/envelope biogenesis (M)*** |  |
| AU1054_G3941 | 0.02 | 5E-07 | 0778 | lipopolysaccharide biosynthesis | No homolog |
| AU1054_G6252 | 0.02 | 1E-06 | 0772 | glycosyl transferase, family 2 | No homolog |
| AU1054_G6091 | 0.03 | 5E-07 | 0875 | rhamnosyltransferase | No homolog |
| AU1054_G6088 | 0.04 | 2E-06 | 0878 | glycosyl transferase, family 2 | No homolog |
| AU1054_G6254 | 0.04 | 8E-07 | 0769 | Capsule polysaccharide biosynthesis | No homolog |
| AU1054_G993 | 0.05 | 3E-07 | 0460 | YD repeat protein | No homolog |
| AU1054_G3697 | 0.07 | 1E-07 | 0882 | methyltransferase FkbM family | No homolog |
| AU1054_G3386 | 0.08 | 6E-08 | 2310 | O-antigen polymerase | No homolog |
| AU1054_G1455 | 0.08 | 2E-05 | 6510 | Methyltransferase type 12 | No homolog |
| BCAL0967 | 0.08 | 1E-07 | 2633 | lipopolysaccharide heptosyltransferase II | BCAL0967 |
| HI2424_G6145 | 0.09 | 3E-04 | 6789 | Lytic transglycosylase, catalytic | No homolog |
| AU1054_G590 | 0.10 | 2E-08 | 4812 | peptidase M15D, vanX D-ala-D-ala dipeptidase | No homolog |
| AU1054_G2327 | 0.12 | 1E-04 | 0235 | hypothetical protein | No homolog |
| AU1054_G994 | 0.13 | 9E-09 | 0460 | YD repeat protein | No homolog |
| AU1054_G541 | 0.15 | 6E-08 | 6604 | hypothetical protein | No homolog |
| AU1054_G3385 | 0.19 | 4E-09 | 2309 | glycosyl transferase, family 2 | No homolog |
| BCAL0575 | 0.19 | 2E-10 | 3016 | YcgR family protein | BCAL0575 |
| BCAM1015 | 0.19 | 4E-10 | 3981 | porin, Gram-negative type | BCAM1015 |
| BCAL2615 | 0.20 | 5E-05 | 2410 | outer membrane protein (porin)-like protein | BCAL2615 |
| AU1054_G1613 | 0.23 | 5E-08 | 4027 | hypothetical protein | No homolog |
| BCAM1738 | 0.23 | 1E-09 | No homolog | No gene annotation | BCAM1738 |
| BCAL2291 | 0.23 | 3E-02 | 2198 | TonB family protein | BCAL2291 |
| HI2424_G6758 | 0.24 | 3E-06 | 3438 | MltA-interacting MipA family protein | No homolog |
| BCAS0066 | 0.26 | 5E-09 | 6141 | porin, Gram-negative type | BCAS0066 |
| BCAL2021 | 0.27 | 2E-11 | 1948 | penicillin-binding protein, 1A family | BCAL2021 |
| AU1054_G3999 | 0.28 | 1E-03 | 0781 | sulfatase | No homolog |
| AU1054_G3593 | 0.28 | 1E-10 | 4628 | porin, Gram-negative type | No homolog |
| BCAM1945 | 0.30 | 6E-11 | 4744 | RND efflux system, outer membrane lipoprotein, NodT family | BCAM1945 |
| BCAL0403 | 0.31 | 1E-06 | 0538 | MltA domain protein | BCAL0403 |
| BCAL1938 | 0.32 | 3E-03 | No homolog | No gene annotation | BCAL1938 |
| BCAL2699 | 0.33 | 3E-12 | 2489 | NAD-dependent epimerase/dehydratase | BCAL2699 |
| BCAM2584 | 0.33 | 3E-03 | No homolog | No gene annotation | BCAM2584 |
| BCAS0605 | 0.34 | 2E-11 | 6279 | NmrA family protein | BCAS0605 |
| BCAM2621_J_1 | 0.34 | 3E-13 | 5427 | porin, Gram-negative type | BCAM2621 |
| BCAL3473 | 0.34 | 1E-05 | 0548 | porin, Gram-negative type | BCAL3473 |
| BCAL1829 | 0.35 | 2E-09 | 1756 | OmpW family protein | BCAL1829 |
| BCAM2551 | 0.35 | 4E-04 | 5352 | efflux transporter, RND family, MFP subunit | BCAM2551 |
| HI2424_G6242 | 0.35 | 2E-08 | 0181 | outer membrane protein (porin) | No homolog |
| BCAM2621_J_0 | 0.35 | 2E-11 | 5427 | porin, Gram-negative type | BCAM2621 |
| BCAM2381 | 0.37 | 3E-02 | 5141 | Substrate-binding region of ABC-type glycine betaine transport system | BCAM2381 |
| BCAL0480 | 0.37 | 1E-10 | 3112 | Rod shape-determining protein MreD | BCAL0480 |
| BCAM2284 | 0.37 | 1E-02 | 5041 | Mandelate racemase/muconate lactonizing enzyme, C-terminal domain protein | BCAM2284 |
| BCAM0179 | 0.37 | 2E-05 | 3171 | MscS Mechanosensitive ion channel | BCAM0179 |
| BCAM1489 | 0.38 | 1E-07 | 4361 | Peptidoglycan glycosyltransferase | No homolog |
| BCAL1813 | 0.38 | 5E-12 | 1741 | RND efflux system, outer membrane lipoprotein, NodT family | BCAL1813 |
| BCAL0624 | 0.38 | 8E-06 | No homolog | No gene annotation | BCAL0624 |
| AU1054_G2827 | 0.39 | 7E-08 | 6455 | NAD-dependent epimerase/dehydratase | No homolog |
| BCAM0944 | 0.40 | 3E-10 | 3910 | peptidase M23B | BCAM0944 |
| BCAM2088 | 0.41 | 1E-03 | 4895 | cell wall hydrolase/autolysin | BCAM2088 |
| BCAM2632 | 0.42 | 3E-08 | No homolog | No gene annotation | BCAM2632 |
| BCAL0960 | 0.43 | 5E-03 | No homolog | No gene annotation | BCAL0960 |
| BCAL2759 | 0.43 | 2E-05 | 2545 | tetraacyldisaccharide 4'-kinase | BCAL2759 |
| BCAL1932 | 0.44 | 7E-13 | 1860 | glycosyl transferase, family 2 | BCAL1932 |
| BCAM1204 | 0.44 | 1E-06 | 4078 | alanine racemase | BCAM1204 |
| AU1054_G4992 | 0.44 | 2E-07 | 6076 | NAD-dependent epimerase/dehydratase | No homolog |
| BCAL3508 | 0.45 | 1E-03 | 0042 | LrgB family protein | BCAL3508 |
| BCAM1003 | 0.46 | 4E-03 | 3969 | NAD-dependent epimerase/dehydratase | BCAM1003 |
| BCAM2311 | 0.48 | 3E-09 | 5070 | porin, Gram-negative type | BCAM2311 |
| BCAL0818 | 0.48 | 8E-04 | 2786 | KpsF/GutQ family protein | BCAL0818 |
| AU1054_G1433 | 0.49 | 5E-08 | 6534 | RND efflux system, outer membrane lipoprotein, NodT family | No homolog |
| AU1054_G6253 | > 0.01 | 1E-06 | 0771 | polysaccharide export protein | No homolog |
| AU1054_G6251 | > 0.01 | 1E-06 | 0774 | glycosyl transferase, group 1 | No homolog |
| AU1054_G6092 | > 0.01 | 4E-07 | 0874 | dTDP-4-dehydrorhamnose reductase | No homolog |
| AU1054_G3696 | > 0.01 | 9E-06 | 0881 | NAD-dependent epimerase/dehydratase | No homolog |
| AU1054_G6250 | > 0.01 | 1E-06 | 0775 | glycosyl transferase, group 1 | No homolog |
|  |  |  |  | ***Cell motility (N)*** |  |
| AU1054_G6081 | 0.19 | 4E-08 | 5569 | PRC-barrel domain protein | No homolog |
| AU1054_G4426 | 0.19 | 5E-08 | 5862 | OmpA/MotB domain protein | No homolog |
| BCAL0959 | 0.23 | 3E-04 | 2646 | hypothetical protein | BCAL0959 |
| BCAL1532 | 0.40 | 3E-11 | 1525 | type II secretion system protein | BCAL1532 |
| BCAL2046 | 0.47 | 1E-02 | 1969 | hypothetical protein | BCAL2046 |
|  |  |  |  | ***Extracellular structures (W)*** |  |
| AU1054_G6275 | 0.05 | 2E-06 | 5223 | Haemagluttinin domain protein | No homolog |
|  |  |  |  | ***Intracellular trafficking, secretion, and vesicular transport (U)*** |  |
| BCAL1525 | 0.20 | 1E-06 | No homolog | No gene annotation | BCAL1525 |
| AU1054_G3841 | 0.23 | 8E-11 | 0053 | general secretion pathway protein L | No homolog |
| BCAL1528 | 0.24 | 2E-05 | 1521 | type II and III secretion system protein | BCAL1528 |
| BCAL1525a | 0.32 | 1E-04 | 1518 | peptidase A24A, prepilin type IV | BCAL1525a |
| BCAM1709 | 0.32 | 5E-03 | No homolog | No gene annotation | BCAM1709 |
| AU1054_G2843 | 0.33 | 6E-09 | 6435 | multiple antibiotic resistance (MarC)-related proteins | No homolog |
| AU1054_G789 | 0.38 | 8E-05 | 1661 | Tetratricopeptide TPR_2 repeat protein | No homolog |
| BCAL1527 | 0.42 | 2E-04 | No homolog | No gene annotation | BCAL1527 |
| BCAL3517_J_0 | 0.43 | 1E-04 | 0053 | general secretion pathway protein L | BCAL3517 |
| BCAM0332 | 0.48 | 2E-03 | 3319 | Conjugal transfer protein TrbG/VirB9/CagX | BCAM0332 |
| HI2424_G6153 | 0.49 | 8E-11 | 6801 | hypothetical protein | No homolog |
| BCAL3516 | 0.50 | 1E-04 | 0052 | General secretion pathway M protein | BCAL3516 |
|  |  |  |  | ***Posttranslational modification, protein turnover, chaperones (O)*** |  |
| AU1054_G5548 | 0.03 | 7E-04 | 6655 | heat shock protein Hsp20 | No homolog |
| AU1054_G2865 | 0.05 | 2E-07 | 6411 | ATPase AAA-2 domain protein | No homolog |
| BCAL1234 | 0.12 | 3E-08 | 1263 | heat shock protein Hsp20 | BCAL1234 |
| BCAM0515 | 0.16 | 2E-09 | No homolog | No gene annotation | BCAM0515 |
| BCAS0638 | 0.20 | 3E-04 | 6250 | chaperonin Cpn10 | BCAS0638 |
| BCAL0849 | 0.21 | 2E-09 | 2755 | peptidase M48, Ste24p | BCAL0849 |
| BCAL3107 | 0.22 | 3E-03 | No homolog | No gene annotation | BCAL3107 |
| BCAL2442 | 0.23 | 1E-10 | 2347 | heat shock protein Hsp90 | BCAL2442 |
| BCAL0501 | 0.23 | 4E-04 | 3091 | 20S proteasome, A and B subunits | BCAL0501 |
| BCAL1919 | 0.26 | 2E-06 | 1847 | ATPase AAA-2 domain protein | BCAL1919 |
| BCAM0548 | 0.26 | 2E-02 | No homolog | No gene annotation | BCAM0548 |
| BCAM0896 | 0.26 | 7E-11 | 3864 | OsmC family protein | BCAM0896 |
| BCAS0724 | 0.26 | 5E-11 | 6245 | methionine-R-sulfoxide reductase | BCAS0724 |
| BCAL1399 | 0.29 | 4E-11 | 1390 | OsmC family protein | BCAL1399 |
| AU1054_G2855 | 0.31 | 9E-08 | 6423 | ATPase AAA-2 domain protein | No homolog |
| AU1054_G6064 | 0.32 | 2E-08 | 5594 | band 7 protein | No homolog |
| BCAL0500 | 0.34 | 4E-03 | No homolog | No gene annotation | BCAL0500 |
| BCAL2071 | 0.35 | 6E-12 | 1999 | SsrA-binding protein | BCAL2071 |
| BCAL3270 | 0.36 | 1E-04 | 0752 | chaperone protein DnaK | BCAL3270 |
| BCAM0833 | 0.39 | 2E-03 | 3799 | OsmC family protein | BCAM0833 |
| BCAM2339 | 0.45 | 1E-08 | 5096 | Isoprenylcysteine carboxyl methyltransferase | BCAM2339 |
| BCAL2153 | 0.45 | 5E-03 | 2081 | peptidyl-prolyl cis-trans isomerase, cyclophilin type | BCAL2153 |
| BCAL3424 | 0.47 | 3E-02 | 0601 | Redoxin domain protein | BCAL3424 |
| BCAM2718 | 0.47 | 1E-12 | No homolog | No gene annotation | BCAM2718 |
| BCAS0085 | 0.48 | 1E-10 | 6136 | OsmC family protein | BCAS0085 |
| BCAM0355 | 0.48 | 2E-04 | 3339 | ABC transporter related | BCAM0355 |
| BCAM1941 | 0.49 | 6E-10 | 4742 | Glutathione S-transferase, N-terminal domain | BCAM1941 |
| BCAL2730 | 0.49 | 2E-02 | 2518 | ATP-dependent Clp protease, ATP-binding subunit clpA | BCAL2730 |
| AU1054_G5547 | > 0.01 | 1E-06 | 6654 | heat shock protein Hsp20 | No homolog |
|  |  |  |  | ***Energy production and conversion (C)*** |  |
| AU1054_G6009 | 0.03 | 2E-05 | 5654 | transcriptional regulator, TetR family | No homolog |
| BCAM2674 | 0.04 | 3E-07 | 5478 | cytochrome bd ubiquinol oxidase, subunit I | BCAM2674 |
| HI2424_G6754 | 0.06 | 2E-07 | 3443 | transposase IS3/IS911 family protein | No homolog |
| BCAS0153 | 0.07 | 2E-07 | 6080 | Sel1 domain protein repeat-containing protein | BCAS0153 |
| AU1054_G2923 | 0.08 | 2E-07 | 6396 | No gene annotation | No homolog |
| AU1054_G2451 | 0.08 | 6E-07 | 3209 | Citrate synthase | No homolog |
| AU1054_G1631 | 0.09 | 7E-08 | 4067 | putative virulence-associated protein | No homolog |
| AU1054_G5887 | 0.11 | 8E-08 | 5918 | regulatory protein, LysR | No homolog |
| AU1054_G524 | 0.11 | 1E-06 | 4945 | Thioesterase | No homolog |
| AU1054_G4090 | 0.12 | 3E-06 | 2144 | hypothetical protein | No homolog |
| AU1054_G3610 | 0.12 | 1E-04 | 4646 | aldo/keto reductase | No homolog |
| HI2424_G6250 | 0.14 | 7E-07 | 0173 | transposase, IS4 family protein | No homolog |
| AU1054_G3613 | 0.14 | 8E-05 | 4649 | (R)-2-hydroxyacid dehydrogenase | No homolog |
| BCAM2675 | 0.15 | 1E-04 | 5479 | cytochrome d ubiquinol oxidase, subunit II | BCAM2675 |
| AU1054_G6289 | 0.16 | 3E-09 | 5196 | cyclic nucleotide-regulated FAD-dependent pyridine nucleotide-disulphide oxidoreductase | No homolog |
| AU1054_G1233 | 0.16 | 4E-04 | 4872 | Phosphate acetyltransferase | No homolog |
| AU1054_G63 | 0.18 | 3E-08 | 0008 | putative insertion element protein | No homolog |
| BCAL0925 | 0.19 | 4E-10 | 2678 | glycerol kinase | BCAL0925 |
| AU1054_G3245 | 0.19 | 1E-09 | 2978 | short-chain dehydrogenase/reductase SDR | No homolog |
| AU1054_G64 | 0.21 | 8E-10 | 0007 | transposase IS3/IS911 family protein | No homolog |
| AU1054_G2916 | 0.21 | 2E-10 | 6396 | No gene annotation | No homolog |
| AU1054_G137 | 0.24 | 7E-03 | 6353 | NADH:flavin oxidoreductase/NADH oxidase | No homolog |
| BCAL2141 | 0.24 | 1E-10 | 2068 | cytochrome C oxidase subunit IV | BCAL2141 |
| AU1054_G2459 | 0.24 | 1E-11 | 3259 | TniB family protein | No homolog |
| pBCA011_J_0 | 0.25 | 9E-09 | 6865 | YadA C-terminal domain protein | pBCA011 |
| BCAL0926 | 0.25 | 4E-07 | 2677 | FAD dependent oxidoreductase | BCAL0926 |
| BCAL0677 | 0.27 | 2E-04 | 2921 | DSBA oxidoreductase | BCAL0677 |
| HI2424_G6151 | 0.27 | 1E-03 | 6799 | protein-disulfide isomerase | No homolog |
| BCAL0042 | 0.28 | 1E-04 | No homolog | No gene annotation | BCAL0042 |
| BCAM2594 | 0.31 | 6E-11 | 5401 | Alcohol dehydrogenase, zinc-binding domain protein | BCAM2594 |
| AU1054_G2426 | 0.32 | 2E-05 | 3245 | 2-oxo-acid dehydrogenase E1 subunit, homodimeric type | No homolog |
| BCAL2142 | 0.32 | 2E-05 | 2069 | cytochrome c oxidase, subunit III | BCAL2142 |
| AU1054_G6298 | 0.32 | 3E-12 | 5180 | transcriptional regulator, XRE family | No homolog |
| AU1054_G1232 | 0.33 | 1E-03 | 4871 | acetate kinase | No homolog |
| HI2424_G6165 | 0.34 | 4E-06 | 6819 | Zeta toxin family protein | No homolog |
| AU1054_G5605 | 0.35 | 3E-10 | 6716 | cytochrome B561 | No homolog |
| BCAM0129 | 0.36 | 1E-03 | 5829 | transcriptional regulator, TetR family | BCAM0129 |
| BCAL1718 | 0.36 | 4E-03 | 1666 | L-carnitine dehydratase/bile acid-inducible protein F | BCAL1718 |
| AU1054_G132 | 0.37 | 7E-08 | 6358 | cytochrome c, class I | No homolog |
| AU1054_G1234 | 0.37 | 1E-02 | 4873 | ATP synthase F1, gamma subunit | No homolog |
| BCAM2093 | 0.38 | 2E-10 | 4900 | monooxygenase, FAD-binding | BCAM2093 |
| AU1054_G2867 | 0.38 | 5E-12 | 6408 | cytochrome d ubiquinol oxidase, subunit II | No homolog |
| AU1054_G5715 | 0.38 | 1E-08 | 5382 | (2Fe-2S)-binding domain protein | No homolog |
| HI2424_G4044 | 0.39 | 3E-10 | 6842 | putative insertion sequence transposase protein | No homolog |
| BCAL2143 | 0.39 | 2E-09 | 2070 | Cytochrome-c oxidase | BCAL2143 |
| BCAL1209 | 0.40 | 1E-05 | 1240 | NADH:flavin oxidoreductase/NADH oxidase | BCAL1209 |
| BCAL0935 | 0.40 | 1E-03 | 2668 | cytochrome c, class I | BCAL0935 |
| BCAM0042 | 0.41 | 3E-03 | 5730 | aldo/keto reductase | BCAM0042 |
| AU1054_G3609 | 0.41 | 1E-06 | 4645 | Alcohol dehydrogenase, zinc-binding domain protein | No homolog |
| AU1054_G1892 | 0.41 | 7E-11 | 6293 | iron-containing alcohol dehydrogenase | No homolog |
| AU1054_G6070 | 0.41 | 6E-06 | 5587 | transposase, IS204/IS1001/IS1096/IS1165 family protein | No homolog |
| AU1054_G1448 | 0.42 | 3E-11 | 6518 | Benzaldehyde dehydrogenase (NAD(+)) | No homolog |
| BCAL2144 | 0.42 | 3E-12 | 2071 | ubiquinol oxidase, subunit II | BCAL2144 |
| BCAL1911 | 0.42 | 1E-12 | 1839 | Pyruvate dehydrogenase (acetyl-transferring) | BCAL1911 |
| AU1054_G134 | 0.42 | 6E-10 | 6356 | aldehyde oxidase and xanthine dehydrogenase, molybdopterin binding | No homolog |
| BCAM2792 | 0.42 | 3E-04 | No homolog | No gene annotation | BCAM2792 |
| AU1054_G2448 | 0.42 | 3E-03 | 3213 | D-isomer specific 2-hydroxyacid dehydrogenase, NAD-binding | No homolog |
| BCAM1606 | 0.42 | 3E-03 | No homolog | No gene annotation | BCAM1606 |
| AU1054_G4974 | 0.42 | 1E-06 | 6058 | aldo/keto reductase | No homolog |
| BCAL3061 | 0.42 | 3E-13 | 0948 | protein of unknown function YGGT | BCAL3061 |
| HI2424_G6252 | 0.43 | 2E-04 | 0171 | NAD-dependent aldehyde dehydrogenases-like protein | No homolog |
| BCAL2429 | 0.43 | 3E-07 | 2331 | cytochrome c, class I | BCAL2429 |
| BCAS0159 | 0.44 | 1E-12 | 6070 | aldo/keto reductase | BCAS0159 |
| AU1054_G133 | 0.44 | 2E-11 | 6357 | (2Fe-2S)-binding domain protein | No homolog |
| BCAL1049 | 0.45 | 2E-08 | 1169 | luciferase family protein | BCAL1049 |
| BCAM0596 | 0.45 | 2E-12 | No homolog | No gene annotation | BCAM0596 |
| BCAL0725 | 0.45 | 2E-07 | 2870 | Redoxin domain protein | BCAL0725 |
| BCAM2733 | 0.45 | 1E-06 | 5548 | acylphosphatase | BCAM2733 |
| HI2424_G6169 | 0.45 | 2E-04 | 6824 | YadA C-terminal domain protein | No homolog |
| AU1054_G5716 | 0.46 | 2E-10 | 5381 | aldehyde oxidase and xanthine dehydrogenase, a/b hammerhead | No homolog |
| BCAL3285 | 0.46 | 3E-02 | 0737 | globin | BCAL3285 |
| BCAM0684 | 0.47 | 5E-04 | 3648 | FAD-dependent pyridine nucleotide-disulphide oxidoreductase | BCAM0684 |
| BCAL2746 | 0.47 | 8E-04 | 2534 | Citrate synthase | BCAL2746 |
| BCAL2023 | 0.47 | 5E-04 | 1950 | Tetratricopeptide TPR_2 repeat protein | BCAL2023 |
| BCAM1565 | 0.48 | 3E-10 | No homolog | No gene annotation | BCAM1565 |
| AU1054_G3595 | 0.48 | 2E-07 | 4631 | Formamidase | No homolog |
| BCAL0356 | 0.48 | 2E-09 | 0490 | Alcohol dehydrogenase, zinc-binding domain protein | BCAL0356 |
| AU1054_G1449 | 0.49 | 5E-09 | 6517 | Alcohol dehydrogenase, zinc-binding domain protein | No homolog |
| BCAS0609 | 0.49 | 1E-04 | 6275 | Electron-transferring-flavoprotein dehydrogenase | BCAS0609 |
| AU1054_G6509 | > 0.01 | 2E-05 | 3337 | transcriptional regulator, XRE family | No homolog |
| AU1054_G6498 | > 0.01 | 7E-06 | 5565 | transcriptional regulator, XRE family | No homolog |
|  |  |  |  | ***Carbohydrate transport and metabolism (G)*** |  |
| AU1054_G6518 | 0.03 | 4E-06 | 0777 | ABC-2 type transporter | No homolog |
| AU1054_G1453 | 0.05 | 2E-06 | 6513 | major facilitator superfamily MFS_1 | No homolog |
| AU1054_G2903 | 0.11 | 3E-05 | 6371 | Aldose 1-epimerase | No homolog |
| BCAL0924 | 0.14 | 2E-09 | 2679 | MIP family channel proteins | BCAL0924 |
| AU1054_G4347 | 0.16 | 6E-08 | 5772 | major facilitator superfamily MFS_1 | No homolog |
| AU1054_G5597 | 0.20 | 2E-07 | 6707 | periplasmic binding protein/LacI transcriptional regulator | No homolog |
| BCAS0324 | 0.20 | 6E-10 | 3284 | ABC transporter related | BCAS0324 |
| BCAL3040 | 0.21 | 1E-04 | 0969 | binding-protein-dependent transport systems inner membrane component | BCAL3040 |
| AU1054_G2129 | 0.23 | 5E-08 | 6316 | glycoside hydrolase, family 3 domain protein | No homolog |
| BCAM0138 | 0.23 | 4E-10 | 5840 | Phosphoglycerate mutase | BCAM0138 |
| BCAS0326 | 0.23 | 5E-04 | 3286 | binding-protein-dependent transport systems inner membrane component | BCAS0326 |
| AU1054_G3614 | 0.24 | 1E-08 | 4650 | major facilitator superfamily MFS_1 | No homolog |
| AU1054_G1918 | 0.25 | 8E-03 | 6265 | major facilitator superfamily MFS_1 | No homolog |
| BCAL0736 | 0.25 | 1E-10 | 2860 | phosphoenolpyruvate-protein phosphotransferase | BCAL0736 |
| HI2424_G6243 | 0.26 | 1E-10 | 0180 | major facilitator superfamily MFS_1 | No homolog |
| BCAL1101 | 0.27 | 1E-08 | 1220 | protein of unknown function DUF6, transmembrane | BCAL1101 |
| BCAL0778 | 0.27 | 9E-04 | 2824 | N-acetylglucosamine-6-phosphate deacetylase | BCAL0778 |
| BCAM2064 | 0.28 | 5E-03 | 4864 | Alpha,alpha-trehalase | BCAM2064 |
| AU1054_G2435 | 0.29 | 3E-07 | 3228 | major facilitator superfamily MFS_1 | No homolog |
| BCAL3041 | 0.29 | 1E-03 | 0968 | extracellular solute-binding protein, family 1 | BCAL3041 |
| BCAL0950 | 0.30 | 9E-12 | 2655 | putative haloacid permease, Deh4p | BCAL0950 |
| BCAL1548 | 0.30 | 3E-03 | 1541 | periplasmic binding protein/LacI transcriptional regulator | BCAL1548 |
| AU1054_G5526 | 0.31 | 6E-07 | 6628 | major facilitator superfamily MFS_1 | No homolog |
| BCAL2419 | 0.31 | 3E-04 | 2323 | glycoside hydrolase 15-related | BCAL2419 |
| BCAM1288 | 0.31 | 8E-08 | 4158 | glycoside hydrolase, family 31 | BCAM1288 |
| BCAL3405 | 0.33 | 9E-04 | 0621 | periplasmic binding protein/LacI transcriptional regulator | BCAL3405 |
| BCAS0328 | 0.33 | 5E-11 | No homolog | No gene annotation | BCAS0328 |
| BCAM2507 | 0.33 | 3E-04 | 5310 | major facilitator superfamily MFS_1 | BCAM2507 |
| AU1054_G1538 | 0.34 | 1E-03 | 3659 | drug resistance transporter, Bcr/CflA subfamily | No homolog |
| BCAL3403 | 0.35 | 2E-04 | 0623 | inner-membrane translocator | BCAL3403 |
| AU1054_G5593 | 0.35 | 2E-09 | 6703 | Mannitol dehydrogenase, C-terminal domain | No homolog |
| BCAL3039 | 0.36 | 9E-03 | 0970 | binding-protein-dependent transport systems inner membrane component | BCAL3039 |
| BCAL0810_J_0 | 0.36 | 6E-05 | 2793 | putative PTS IIA-like nitrogen-regulatory protein PtsN | BCAL0810 |
| AU1054_G2492 | 0.37 | 2E-08 | 4202 | hypothetical protein | No homolog |
| AU1054_G5615 | 0.38 | 7E-11 | 6726 | glycosyl hydrolase, BNR repeat-containing protein | No homolog |
| HI2424_G6259 | 0.38 | 1E-03 | 0162 | major facilitator superfamily MFS_1 | No homolog |
| BCAL2841 | 0.40 | 3E-11 | No homolog | No gene annotation | BCAL2841 |
| BCAM2349A | 0.41 | 9E-11 | 5106 | Cupin 2, conserved barrel domain protein | BCAM2349A |
| BCAM1761 | 0.41 | 3E-04 | 4584 | hypothetical protein | BCAM1761 |
| BCAM0094 | 0.41 | 4E-03 | 5794 | xylulokinase | BCAM0094 |
| BCAL1434 | 0.42 | 2E-03 | 1427 | Xylose isomerase domain protein TIM barrel | BCAL1434 |
| BCAS0595 | 0.42 | 1E-07 | No homolog | No gene annotation | BCAS0595 |
| AU1054_G4943 | 0.42 | 4E-07 | 6025 | 3-carboxymuconate cyclase-like protein | No homolog |
| AU1054_G5598 | 0.42 | 2E-11 | 6708 | inner-membrane translocator | No homolog |
| BCAS0594 | 0.42 | 1E-05 | 6290 | ribokinase | BCAS0594 |
| AU1054_G2138 | 0.43 | 3E-09 | 6306 | ABC transporter related | No homolog |
| BCAL2525 | 0.43 | 4E-06 | No homolog | No gene annotation | BCAL2525 |
| AU1054_G2213 | 0.43 | 4E-08 | 6306 | ABC transporter related | No homolog |
| BCAM1460 | 0.44 | 1E-03 | 4332 | major facilitator superfamily MFS_1 | BCAM1460 |
| BCAL3276 | 0.45 | 2E-03 | 0744 | NAD(+) kinase | BCAL3276 |
| BCAL1550 | 0.45 | 7E-03 | 1543 | ABC transporter related | BCAL1550 |
| BCAM1444 | 0.46 | 9E-12 | 4316 | protein of unknown function DUF6, transmembrane | BCAM1444 |
| AU1054_G5968 | 0.46 | 1E-10 | 5383 | major facilitator superfamily MFS_1 | No homolog |
| AU1054_G6077 | 0.47 | 3E-07 | 5573 | transketolase | No homolog |
| BCAL2625 | 0.47 | 2E-02 | 2420 | major facilitator superfamily MFS_1 | BCAL2625 |
| BCAL3389 | 0.47 | 1E-03 | 0638 | transketolase | BCAL3389 |
| BCAL2262 | 0.48 | 9E-12 | 2181 | major facilitator superfamily MFS_1 | BCAL2262 |
| AU1054_G2140 | 0.49 | 8E-06 | 6304 | binding-protein-dependent transport systems inner membrane component | No homolog |
| BCAL3181 | 0.50 | 1E-03 | 0823 | major facilitator superfamily MFS_1 | BCAL3181 |
| BCAL1556 | 0.50 | 6E-03 | 1549 | ribose 5-phosphate isomerase | BCAL1556 |
| AU1054_G6090 | > 0.01 | 5E-05 | 0876 | ABC-2 type transporter | No homolog |
| AU1054_G6089 | > 0.01 | 1E-06 | 0877 | ABC transporter related | No homolog |
| AU1054_G3694 | > 0.01 | 8E-06 | 0879 | mannose-1-phosphate guanylyltransferase/mannose-6-phosphate isomerase | No homolog |
|  |  |  |  | ***Amino acid transport and metabolism (E)*** |  |
| HI2424_G6711 | 0.03 | 6E-06 | 3492 | Extracellular ligand-binding receptor | No homolog |
| BCAM2771 | 0.03 | 2E-07 | 5613 | Dihydrodipicolinate synthase | BCAM2771 |
| AU1054_G5780 | 0.07 | 3E-07 | 5315 | Extracellular ligand-binding receptor | No homolog |
| AU1054_G5511 | 0.10 | 8E-07 | 6610 | ABC transporter related | No homolog |
| BCAM2307 | 0.10 | 4E-06 | 5066 | zinc metalloprotease ZmpB | BCAM2307 |
| AU1054_G2917 | 0.11 | 1E-07 | 6410 | Glyoxalase/bleomycin resistance protein/dioxygenase | No homolog |
| BCAL2708 | 0.11 | 1E-08 | 2497 | inner-membrane translocator | BCAL2708 |
| AU1054_G3607 | 0.14 | 1E-04 | 4643 | branched-chain amino acid aminotransferase | No homolog |
| BCAM0193 | 0.15 | 4E-03 | 3185 | Cupin 2, conserved barrel domain protein | BCAM0193 |
| AU1054_G5509 | 0.17 | 2E-06 | 6609 | acetylornithine deacetylase or succinyl-diaminopimelate desuccinylase | No homolog |
| AU1054_G5516 | 0.18 | 2E-07 | 6616 | FAD dependent oxidoreductase | No homolog |
| BCAS0372 | 0.18 | 2E-10 | 6696 | Dihydroxy-acid dehydratase | BCAS0372 |
| AU1054_G5782 | 0.20 | 2E-06 | 5314 | aminotransferase, class IV | No homolog |
| BCAL2707 | 0.22 | 2E-11 | 2496 | inner-membrane translocator | BCAL2707 |
| BCAL2844 | 0.23 | 2E-10 | 2631 | branched-chain amino acid aminotransferase | BCAL2844 |
| BCAS0111 | 0.23 | 2E-03 | 6114 | polar amino acid ABC transporter, inner membrane subunit | BCAS0111 |
| AU1054_G5539 | 0.23 | 2E-10 | 6641 | FAD dependent oxidoreductase | No homolog |
| BCAL2706 | 0.24 | 6E-11 | 2495 | ABC transporter related | BCAL2706 |
| HI2424_G6262 | 0.24 | 4E-11 | 0159 | Glyoxalase/bleomycin resistance protein/dioxygenase | No homolog |
| BCAM2512 | 0.26 | 3E-07 | 5317 | dihydrodipicolinate synthetase | BCAM2512 |
| AU1054_G5899 | 0.26 | 9E-09 | 5905 | Cysteine synthase | No homolog |
| AU1054_G2464 | 0.27 | 1E-10 | 3210 | Glyoxalase/bleomycin resistance protein/dioxygenase | No homolog |
| BCAS0128 | 0.27 | 2E-10 | 6102 | glycine betaine/L-proline ABC transporter, ATPase subunit | BCAS0128 |
| BCAL0075 | 0.28 | 9E-06 | 0146 | glycine cleavage system T protein | BCAL0075 |
| BCAM2502 | 0.28 | 2E-09 | No homolog | No gene annotation | BCAM2502 |
| AU1054_G5781 | 0.28 | 6E-09 | 5315 | Extracellular ligand-binding receptor | No homolog |
| BCAS0751 | 0.31 | 4E-06 | 6219 | Gamma-glutamyltransferase | BCAS0751 |
| BCAL0864 | 0.31 | 1E-10 | 2742 | amino acid aldolase or racemase-like protein | BCAL0864 |
| AU1054_G2136 | 0.32 | 1E-07 | 6309 | glucose-methanol-choline oxidoreductase | No homolog |
| BCAL3104 | 0.32 | 2E-08 | 0902 | urease, gamma subunit | BCAL3104 |
| AU1054_G2894 | 0.32 | 3E-04 | 6380 | Lysine exporter protein (LYSE/YGGA) | No homolog |
| BCAM2519 | 0.32 | 6E-04 | 5326 | Lysine exporter protein (LYSE/YGGA) | BCAM2519 |
| BCAM2726 | 0.33 | 3E-05 | 5540 | binding-protein-dependent transport systems inner membrane component | BCAM2726 |
| BCAS0110 | 0.34 | 7E-03 | No homolog | No gene annotation | BCAS0110 |
| AU1054_G2609 | 0.34 | 1E-11 | 1985 | Glyoxalase/bleomycin resistance protein/dioxygenase | No homolog |
| AU1054_G2881 | 0.34 | 5E-09 | 6393 | Extracellular ligand-binding receptor | No homolog |
| IG3_121896 | 0.35 | 4E-03 | 6115 | extracellular solute-binding protein, family 3 | Intergenic region, chromosome 3 |
| BCAM2706 | 0.36 | 3E-12 | No homolog | No gene annotation | BCAM2706 |
| BCAL2155 | 0.36 | 8E-08 | 2083 | serine O-acetyltransferase | BCAL2155 |
| BCAM2501 | 0.36 | 1E-04 | 5303 | Shikimate dehydrogenase substrate binding, N-terminal domain protein | BCAM2501 |
| AU1054_G5527 | 0.37 | 8E-08 | 6629 | Dihydroxy-acid dehydratase | No homolog |
| BCAL2100 | 0.38 | 7E-12 | 2029 | aminotransferase, class I and II | BCAL2100 |
| BCAL2357 | 0.39 | 1E-03 | 2263 | ketol-acid reductoisomerase | BCAL2357 |
| AU1054_G4350 | 0.39 | 2E-07 | 5769 | lysine 2,3-aminomutase YodO family protein | No homolog |
| BCAM1370 | 0.40 | 4E-04 | 4235 | 5-carboxymethyl-2-hydroxymuconate isomerase | BCAM1370 |
| BCAL2705 | 0.40 | 3E-06 | No homolog | No gene annotation | BCAL2705 |
| BCAL0015 | 0.43 | 3E-02 | 0083 | ABC transporter related | BCAL0015 |
| BCAS0136 | 0.43 | 5E-04 | 6096 | amino acid permease-associated region | BCAS0136 |
| AU1054_G2841 | 0.43 | 2E-10 | 6437 | Alcohol dehydrogenase GroES domain protein | No homolog |
| BCAM2382 | 0.43 | 7E-06 | 5142 | binding-protein-dependent transport systems inner membrane component | BCAM2382 |
| BCAM1819 | 0.43 | 2E-02 | 4679 | Urea transporter | BCAM1819 |
| BCAS0112 | 0.44 | 1E-03 | No homolog | No gene annotation | BCAS0112 |
| AU1054_G5544 | 0.44 | 2E-09 | 6646 | ABC transporter related | No homolog |
| BCAL0039 | 0.44 | 1E-02 | 0110 | Prephenate dehydratase | BCAL0039 |
| BCAL0544 | 0.44 | 1E-03 | 3045 | extracellular solute-binding protein, family 5 | BCAL0544 |
| AU1054_G3540 | 0.44 | 1E-10 | 4574 | putative threonine efflux protein-like protein | No homolog |
| BCAL2437 | 0.44 | 6E-03 | 2339 | spermidine synthase-like protein | BCAL2437 |
| BCAL1094 | 0.45 | 3E-12 | 1212 | amino acid permease-associated region | BCAL1094 |
| BCAL3367 | 0.45 | 7E-03 | 0660 | 6-phosphogluconate dehydratase | BCAL3367 |
| BCAM2295 | 0.45 | 2E-04 | 5052 | Substrate-binding region of ABC-type glycine betaine transport system | BCAM2295 |
| AU1054_G5665 | 0.46 | 2E-11 | 6617 | dihydrodipicolinate synthetase | No homolog |
| BCAL3362 | 0.46 | 1E-06 | 0665 | FAD dependent oxidoreductase | BCAL3362 |
| BCAL3411 | 0.47 | 5E-03 | 0615 | peptidase S10, serine carboxypeptidase | BCAL3411 |
| BCAL0681 | 0.47 | 3E-11 | No homolog | No gene annotation | BCAL0681 |
| BCAM2149 | 0.48 | 5E-07 | 4911 | peptidase M20 | BCAM2149 |
| BCAS0114 | 0.48 | 1E-02 | 6111 | phenylalanine/histidine ammonia-lyase | BCAS0114 |
| BCAS0113 | 0.48 | 9E-03 | 6112 | ABC transporter related | BCAS0113 |
| BCAL2244 | 0.49 | 3E-05 | 2170 | urocanate hydratase | BCAL2244 |
| BCAM2316 | 0.49 | 3E-02 | No homolog | No gene annotation | BCAM2316 |
| BCAL0073 | 0.49 | 1E-03 | 0144 | glycine dehydrogenase | BCAL0073 |
| BCAL2266 | 0.49 | 8E-12 | 2185 | HAD-superfamily subfamily IB hydrolase, TIGR01490 | BCAL2266 |
| BCAM1111 | 0.49 | 3E-02 | No homolog | No gene annotation | BCAM1111 |
| AU1054_G426 | 0.49 | 1E-06 | 3995 | amino acid permease-associated region | No homolog |
| BCAM1134 | 0.50 | 2E-05 | 4029 | Asp/Glu racemase | BCAM1134 |
| BCAL2019 | 0.50 | 1E-03 | 1946 | Pyridoxal-5'-phosphate-dependent enzyme, beta subunit | BCAL2019 |
| AU1054_G2378 | > 0.01 | 5E-05 | 0229 | protein of unknown function DUF955 | No homolog |
| HI2424_G6384 | > 0.01 | 9E-07 | 0188 | protein of unknown function DUF955 | No homolog |
|  |  |  |  | ***Nucleotide transport and metabolism (F)*** |  |
| AU1054_G4904 | 0.05 | 2E-07 | 3737 | response regulator receiver protein | No homolog |
| BCAL1701 | 0.15 | 8E-07 | No homolog | No gene annotation | BCAL1701 |
| BCAL1220 | 0.23 | 7E-11 | No homolog | No gene annotation | BCAL1220 |
| IG2_1187397 | 0.35 | 1E-11 | 4002 | uracil-xanthine permease | BCAM1099 |
| BCAL3494 | 0.35 | 8E-05 | 0028 | Site-specific DNA-methyltransferase (adenine-specific) | BCAL3494 |
| BCAL2387 | 0.37 | 3E-07 | 2293 | uracil phosphoribosyltransferase | BCAL2387 |
| BCAL2045 | 0.43 | 2E-05 | No homolog | No gene annotation | BCAL2045 |
| BCAS0730 | 0.47 | 4E-04 | 6239 | Na+ dependent nucleoside transporter domain protein | BCAS0730 |
| AU1054_G5517 | 0.47 | 2E-07 | 6618 | dihydropyrimidinase | No homolog |
| BCAL2836 | 0.50 | 4E-03 | 2623 | phosphoribosylaminoimidazole carboxylase, ATPase subunit | BCAL2836 |
| AU1054_G5776 | > 0.01 | 6E-05 | 5319 | hypothetical protein | No homolog |
|  |  |  |  | ***Coenzyme transport and metabolism (H)*** |  |
| AU1054_G5891 | 0.03 | 3E-07 | 5913 | aminotransferase class-III | No homolog |
| AU1054_G2872 | 0.24 | 7E-05 | 6403 | molybdopterin biosynthesis MoaE | No homolog |
| BCAL2441 | 0.26 | 2E-04 | 2346 | Chorismate lyase | BCAL2441 |
| BCAL0665 | 0.28 | 5E-08 | 2933 | dethiobiotin synthase | BCAL0665 |
| BCAL2391 | 0.32 | 6E-06 | 2297 | nicotinate (nicotinamide) nucleotide adenylyltransferase | BCAL2391 |
| BCAL0614 | 0.35 | 3E-12 | No homolog | No gene annotation | BCAL0614 |
| BCAL2781 | 0.36 | 5E-12 | 2567 | UBA/THIF-type NAD/FAD binding protein | BCAL2781 |
| AU1054_G2422 | 0.37 | 7E-11 | 3249 | 3,4-dihydroxy-2-butanone 4-phosphate synthase | No homolog |
| BCAL2656 | 0.37 | 6E-04 | No homolog | No gene annotation | BCAL2656 |
| BCAL0298 | 0.40 | 3E-03 | 0411 | thiamine biosynthesis protein ThiS | BCAL0298 |
| BCAL1708 | 0.40 | 3E-09 | No homolog | No gene annotation | BCAL1708 |
| BCAL0962 | 0.42 | 7E-11 | 2638 | molybdenum cofactor biosynthesis protein C | BCAL0962 |
| BCAL1859 | 0.43 | 5E-11 | No homolog | No gene annotation | BCAL1859 |
| BCAL2629 | 0.45 | 2E-03 | No homolog | No gene annotation | BCAL2629 |
| BCAL0667 | 0.47 | 5E-06 | 2931 | adenosylmethionine-8-amino-7-oxononanoate aminotransferase | BCAL0667 |
| HI2424_G6720 | > 0.01 | 2E-06 | 3480 | MoeA domain protein, domain I and II | No homolog |
|  |  |  |  | ***Lipid transport and metabolism (I)*** |  |
| AU1054_G2440 | 0.06 | 1E-05 | 3221 | Alpha/beta hydrolase fold-3 domain protein | No homolog |
| AU1054_G1888 | 0.08 | 7E-08 | 6299 | short-chain dehydrogenase/reductase SDR | No homolog |
| AU1054_G6475 | 0.11 | 6E-03 | 3328 | hypothetical protein | No homolog |
| HI2424_G4060 | 0.11 | 6E-09 | 6863 | short-chain dehydrogenase/reductase SDR | No homolog |
| HI2424_G6247 | 0.12 | 7E-06 | 0176 | Enoyl-CoA hydratase/isomerase | No homolog |
| AU1054_G1452 | 0.13 | 1E-04 | 6514 | short-chain dehydrogenase/reductase SDR | No homolog |
| HI2424_G7007 | 0.13 | 2E-09 | 3485 | acyl-CoA dehydrogenase domain protein | No homolog |
| AU1054_G5889 | 0.15 | 3E-08 | 5916 | short-chain dehydrogenase/reductase SDR | No homolog |
| HI2424_G6241 | 0.20 | 4E-10 | 0182 | Carboxylesterase, type B | No homolog |
| AU1054_G424 | 0.23 | 4E-07 | 3998 | short-chain dehydrogenase/reductase SDR | No homolog |
| BCAL1636 | 0.24 | 5E-06 | 1585 | phosphatidylserine/phosphatidylglycerophosphate/cardiolipin synthases like enzyme | BCAL1636 |
| AU1054_G3439 | 0.26 | 7E-09 | 2365 | hypothetical protein | No homolog |
| AU1054_G1231 | 0.26 | 6E-03 | 4870 | PHB de-polymerase domain protein | No homolog |
| AU1054_G2415 | 0.28 | 1E-09 | 3256 | AMP-dependent synthetase and ligase | No homolog |
| BCAM0392 | 0.28 | 8E-05 | 3378 | acyltransferase 3 | BCAM0392 |
| HI2424_G6164 | 0.29 | 2E-03 | 6817 | putative endonuclease | No homolog |
| BCAM2395 | 0.29 | 1E-05 | No homolog | No gene annotation | BCAM2395 |
| HI2424_G6249 | 0.30 | 1E-08 | 0174 | short-chain dehydrogenase/reductase SDR | No homolog |
| BCAL2016 | 0.30 | 1E-12 | 1943 | 2-C-methyl-D-erythritol 4-phosphate cytidylyltransferase | BCAL2016 |
| BCAS0616 | 0.30 | 1E-07 | 6272 | AMP-dependent synthetase and ligase | BCAS0616 |
| BCAM2552 | 0.31 | 2E-06 | 5353 | Alpha/beta hydrolase fold-3 domain protein | BCAM2552 |
| BCAL1980 | 0.32 | 3E-03 | No homolog | No gene annotation | BCAL1980 |
| AU1054_G2416 | 0.32 | 7E-11 | 3255 | Enoyl-CoA hydratase/isomerase | No homolog |
| BCAS0607 | 0.34 | 3E-04 | 6277 | 3-hydroxybutyryl-CoA dehydrogenase | BCAS0607 |
| BCAL0845 | 0.34 | 7E-06 | 2759 | hypothetical protein | BCAL0845 |
| BCAM1640 | 0.35 | 1E-03 | 4467 | acyltransferase 3 | BCAM1640 |
| BCAM1710 | 0.36 | 3E-02 | 4528 | Enoyl-CoA hydratase/isomerase | BCAM1710 |
| BCAL2007 | 0.38 | 5E-03 | 1934 | Squalene/phytoene synthase | BCAL2007 |
| BCAM0054 | 0.38 | 2E-04 | 5742 | short-chain dehydrogenase/reductase SDR | BCAM0054 |
| AU1054_G4349 | 0.38 | 2E-03 | 5770 | protein of unknown function DUF201 | No homolog |
| AU1054_G5711 | 0.39 | 1E-08 | 5387 | short-chain dehydrogenase/reductase SDR | No homolog |
| BCAM1462 | 0.39 | 2E-03 | 4334 | short-chain dehydrogenase/reductase SDR | BCAM1462 |
| BCAS0737 | 0.40 | 9E-03 | 6232 | acetyl-CoA acetyltransferases | BCAS0737 |
| BCAL1384 | 0.40 | 4E-08 | 1373 | phospholipid/glycerol acyltransferase | BCAL1384 |
| BCAL2015 | 0.40 | 2E-07 | 1942 | 2C-methyl-D-erythritol 2,4-cyclodiphosphate synthase | BCAL2015 |
| BCAS0493_J_0 | 0.41 | 3E-05 | 6527 | short-chain dehydrogenase/reductase SDR | BCAS0493 |
| BCAL0838 | 0.41 | 1E-05 | 2766 | beta-ketoacyl synthase | BCAL0838 |
| BCAS0086 | 0.44 | 3E-05 | 6135 | Alpha/beta hydrolase fold-3 domain protein | BCAS0086 |
| AU1054_G5553 | 0.44 | 4E-10 | 6660 | short-chain dehydrogenase/reductase SDR | No homolog |
| AU1054_G4914 | 0.48 | 1E-02 | 5994 | (Acyl-carrier-protein) phosphodiesterase | No homolog |
| AU1054_G2419 | 0.49 | 4E-04 | 3252 | Enoyl-CoA hydratase/isomerase | No homolog |
| AU1054_G127 | 0.50 | 2E-05 | 4656 | 6-phosphogluconate dehydrogenase, NAD-binding | No homolog |
| AU1054_G2858 | > 0.01 | 8E-07 | 6419 | phospholipase D/Transphosphatidylase | No homolog |
|  |  |  |  | ***Inorganic ion transport and metabolism (P)*** |  |
| BCAM2626 | 0.04 | 8E-03 | 5431 | TonB-dependent hemoglobin/transferrin/lactoferrin family receptor | BCAM2626 |
| BCAM2630 | 0.05 | 4E-03 | 5435 | ABC transporter related | BCAM2630 |
| BCAM2224 | 0.06 | 4E-03 | 4993 | Fe(III)-pyochelin outer membrane receptor precursor | BCAM2224 |
| BCAM2627 | 0.06 | 1E-02 | 5432 | Hemin-degrading family protein | BCAM2627 |
| AU1054_G960 | 0.10 | 5E-08 | 4671 | aliphatic sulfonates family ABC transporter, periplsmic ligand-binding protein | No homolog |
| BCAM2629 | 0.10 | 1E-02 | 5434 | transport system permease protein | BCAM2629 |
| BCAM2628 | 0.10 | 5E-03 | 5433 | periplasmic binding protein | BCAM2628 |
| BCAL2297 | 0.11 | 1E-02 | 2204 | hypothetical protein | BCAL2297 |
| HI2424_G6716 | 0.15 | 9E-09 | 3484 | sodium/hydrogen exchanger | No homolog |
| AU1054_G1479 | 0.19 | 5E-06 | 6481 | Catalase | No homolog |
| AU1054_G6076 | 0.24 | 6E-05 | 5575 | sodium/hydrogen exchanger | No homolog |
| BCAM2439 | 0.28 | 1E-02 | No homolog | No gene annotation | BCAM2439 |
| AU1054_G3743 | 0.31 | 2E-10 | 0939 | Chromate transporter | No homolog |
| BCAL1692 | 0.31 | 8E-03 | 1636 | putative iron transport-related membrane protein, OrbD | BCAL1692 |
| BCAL0028 | 0.32 | 9E-04 | 0097 | Citrate transporter | BCAL0028 |
| AU1054_G4228 | 0.32 | 1E-11 | 3200 | Carbonate dehydratase | No homolog |
| BCAM2367 | 0.35 | 6E-03 | 5125 | TonB-dependent receptor | BCAM2367 |
| BCAL0604 | 0.38 | 1E-05 | 2989 | Heavy metal transport/detoxification protein | BCAL0604 |
| AU1054_G3744 | 0.38 | 5E-06 | 0940 | Chromate transporter | No homolog |
| BCAS0421_J_0 | 0.38 | 3E-11 | 6589 | ABC transporter related | BCAS0421 |
| BCAL2051 | 0.40 | 2E-10 | 1974 | nitrate ABC transporter, periplasmic nitrate-binding protein, putative | BCAL2051 |
| BCAL0711 | 0.40 | 2E-08 | 2886 | taurine ABC transporter, periplasmic binding protein | BCAL0711 |
| BCAM1521 | 0.40 | 2E-04 | 4394 | metabolite/H+ symporter, major facilitator superfamily (MFS) | BCAM1521 |
| AU1054_G1051 | 0.42 | 5E-09 | 6589 | ABC transporter related | No homolog |
| BCAM2313 | 0.43 | 5E-09 | 5072 | sulfatase | BCAM2313 |
| BCAM0400 | 0.44 | 8E-03 | 3388 | Carbonate dehydratase | BCAM0400 |
| BCAL2603 | 0.44 | 7E-04 | 2398 | binding-protein-dependent transport systems inner membrane component | BCAL2603 |
| BCAL1371 | 0.45 | 2E-04 | 1361 | TonB-dependent siderophore receptor | BCAL1371 |
| BCAM1385 | 0.47 | 4E-07 | 4250 | aliphatic sulfonates family ABC transporter, periplsmic ligand-binding protein | BCAM1385 |
| BCAL1726 | 0.47 | 3E-11 | 1674 | precorrin-3B synthase | BCAL1726 |
| BCAL0713 | 0.47 | 1E-02 | 2884 | binding-protein-dependent transport systems inner membrane component | BCAL0713 |
| BCAS0457 | 0.47 | 1E-03 | 6556 | alkylphosphonate utilization operon protein PhnA | BCAS0457 |
| BCAL2573 | 0.48 | 6E-10 | No homolog | No gene annotation | BCAL2573 |
| BCAM0569 | 0.48 | 5E-04 | 3543 | sulfatase | BCAM0569 |
| AU1054_G2852 | > 0.01 | 5E-07 | 6426 | Dyp-type peroxidase family | No homolog |
|  |  |  |  | ***Secondary metabolites biosynthesis, transport, and catabolism (Q)*** |  |
| AU1054_G149 | 0.04 | 2E-03 | 5976 | fumarylacetoacetase | No homolog |
| AU1054_G3445 | 0.04 | 3E-06 | 2379 | Phytanoyl-CoA dioxygenase | No homolog |
| HI2424_G6713 | 0.07 | 3E-08 | 3488 | Thioesterase | No homolog |
| BCAM2232 | 0.09 | 1E-02 | 5000 | AMP-dependent synthetase and ligase | BCAM2232 |
| BCAL1697 | 0.09 | 1E-06 | 1641 | ornibactin biosynthesis non-ribosomal peptide, OrbJ | BCAL1697 |
| AU1054_G5979 | 0.11 | 8E-08 | 5898 | Methyltransferase type 11 | No homolog |
| BCAM2233 | 0.12 | 2E-02 | 5001 | Thioesterase | BCAM2233 |
| HI2424_G4053 | 0.12 | 4E-06 | 6855 | DsbA oxidoreductase | No homolog |
| BCAM2230 | 0.14 | 2E-02 | 4998 | amino acid adenylation domain | BCAM2230 |
| BCAL1700 | 0.16 | 7E-05 | 1644 | ornibactin receptor precursor, OrbA | BCAL1700 |
| HI2424_G6261 | 0.17 | 1E-07 | 0160 | 5-oxopent-3-ene-1,2,5-tricarboxylate decarboxylase | No homolog |
| AU1054_G5275 | 0.19 | 9E-06 | 4343 | Methyltransferase type 11 | No homolog |
| BCAM0195 | 0.19 | 5E-03 | 3187 | amino acid adenylation domain | BCAM0195 |
| BCAL0405 | 0.21 | 8E-03 | 0540 | phenylacetic acid degradation protein PaaI | BCAL0405 |
| AU1054_G542 | 0.21 | 2E-10 | 6603 | amidohydrolase | No homolog |
| BCAL0214 | 0.22 | 2E-02 | 0328 | phenylacetate-CoA oxygenase, PaaI subunit | BCAL0214 |
| BCAL0213 | 0.23 | 2E-02 | 0327 | phenylacetate-CoA oxygenase, PaaJ subunit | BCAL0213 |
| BCAM0499 | 0.24 | 1E-05 | 3465 | TonB-dependent siderophore receptor | BCAM0499 |
| HI2424_G6248 | 0.25 | 2E-07 | 0175 | AMP-dependent synthetase and ligase | No homolog |
| BCAL0686 | 0.25 | 3E-11 | 2912 | fumarylacetoacetate (FAA) hydrolase | BCAL0686 |
| AU1054_G2611 | 0.26 | 6E-11 | 1987 | phenazine biosynthesis protein PhzF family | No homolog |
| BCAL1696 | 0.27 | 4E-02 | 1640 | ornibactin biosynthesis non-ribosomal peptide, OrbI | BCAL1696 |
| AU1054_G2902 | 0.30 | 5E-04 | 6372 | phenazine biosynthesis protein PhzF family | No homolog |
| BCAM1751 | 0.31 | 5E-12 | 4572 | isochorismatase hydrolase | BCAM1751 |
| BCAL0409 | 0.35 | 3E-04 | 0544 | phenylacetic acid degradation enoyl-CoA hydratase PaaF | BCAL0409 |
| AU1054_G4206 | 0.36 | 7E-07 | 2901 | protein of unknown function DUF140 | No homolog |
| BCAM1144 | 0.36 | 3E-09 | 4041 | Asp/Glu racemase | BCAM1144 |
| BCAM1731 | 0.37 | 4E-12 | 4549 | isochorismatase hydrolase | BCAM1731 |
| BCAM1582 | 0.40 | 1E-03 | No homolog | No gene annotation | BCAM1582 |
| AU1054_G5525 | 0.40 | 7E-12 | 6627 | Taurine catabolism dioxygenase TauD/TfdA | No homolog |
| BCAL0668 | 0.41 | 7E-06 | 2930 | dienelactone hydrolase and related enzymes-like protein | BCAL0668 |
| BCAM1366 | 0.43 | 9E-04 | 4231 | 4-hydroxyphenylacetate degradation bifunctional isomerase/decarboxylase, HpaG1 subunit | BCAM1366 |
| BCAM2227 | 0.43 | 2E-02 | 4996 | thiazolinyl imide reductase | BCAM2227 |
| BCAL2242 | 0.45 | 2E-10 | 2168 | imidazolonepropionase | BCAL2242 |
| BCAL0303 | 0.46 | 5E-12 | No homolog | No gene annotation | BCAL0303 |
| HI2424_G6714 | > 0.01 | 6E-07 | 3487 | amino acid adenylation domain | No homolog |
| HI2424_G7006 | > 0.01 | 2E-06 | 3489 | hypothetical protein | No homolog |
|  |  |  |  | ***General function prediction only (R)*** |  |
| HI2424_G6736 | 0.02 | 2E-06 | 3463 | Ankyrin | No homolog |
| AU1054_G2318 | 0.03 | 1E-06 | 0220 | helicase domain protein | No homolog |
| AU1054_G6277 | 0.03 | 1E-04 | 5213 | KAP P-loop domain protein | No homolog |
| AU1054_G2857 | 0.03 | 1E-06 | 6420 | Sel1 domain protein repeat-containing protein | No homolog |
| AU1054_G2293 | 0.04 | 4E-07 | 2641 | glycosyl hydrolase, BNR repeat-containing protein | No homolog |
| AU1054_G5775 | 0.05 | 3E-06 | 5320 | PilT protein domain protein | No homolog |
| AU1054_G6278 | 0.05 | 1E-06 | 5211 | hypothetical protein | No homolog |
| AU1054_G5286 | 0.05 | 1E-04 | 4331 | HipA domain protein | No homolog |
| AU1054_G1176 | 0.07 | 2E-07 | 4092 | PilT protein domain protein | No homolog |
| BCAS0371 | 0.09 | 2E-08 | 6697 | hypothetical protein | BCAS0371 |
| AU1054_G6281 | 0.09 | 2E-08 | 5207 | alpha/beta hydrolase fold | No homolog |
| AU1054_G148 | 0.10 | 3E-08 | 5975 | cyclase family protein | No homolog |
| AU1054_G1454 | 0.10 | 3E-08 | 6512 | HAD-superfamily hydrolase, subfamily IA, variant 3 | No homolog |
| BCAM2308 | 0.12 | 9E-04 | 5067 | leucyl aminopeptidase | BCAM2308 |
| AU1054_G6528 | 0.12 | 1E-05 | 5191 | transport-associated | No homolog |
| HI2424_G6244 | 0.13 | 2E-06 | 0179 | amidohydrolase 2 | No homolog |
| BCAM2522 | 0.13 | 6E-09 | 5329 | carbonic anhydrases/acetyltransferases isoleucine patch superfamily-like protein | BCAM2522 |
| BCAL1034 | 0.14 | 5E-09 | 1156 | electron transport protein SCO1/SenC | BCAL1034 |
| AU1054_G5913 | 0.15 | 5E-09 | 5889 | alpha/beta hydrolase fold | No homolog |
| AU1054_G1323 | 0.16 | 3E-09 | 4770 | dienelactone hydrolase-like protein | No homolog |
| BCAM0165 | 0.16 | 8E-03 | 5856 | hypothetical protein | BCAM0165 |
| AU1054_G5215 | 0.17 | 7E-10 | 4410 | Patatin | No homolog |
| AU1054_G4973 | 0.18 | 2E-09 | 6057 | flavodoxin/nitric oxide synthase | No homolog |
| AU1054_G4810 | 0.20 | 9E-10 | 3761 | hypothetical protein | No homolog |
| BCAL1921 | 0.22 | 7E-03 | 1849 | globin | BCAL1921 |
| BCAL1383 | 0.22 | 3E-11 | 1372 | phosphatidate cytidylyltransferase | BCAL1383 |
| AU1054_G508 | 0.23 | 2E-06 | 4941 | short-chain dehydrogenase/reductase SDR | No homolog |
| BCAM2163 | 0.23 | 8E-11 | 4927 | Antibiotic biosynthesis monooxygenase | BCAM2163 |
| BCAM2184 | 0.24 | 4E-10 | 4952 | metal dependent phosphohydrolase | BCAM2184 |
| AU1054_G1933 | 0.24 | 1E-04 | 6247 | short-chain dehydrogenase/reductase SDR | No homolog |
| AU1054_G2386 | 0.25 | 1E-09 | 3294 | hypothetical protein | No homolog |
| HI2424_G4048 | 0.26 | 8E-07 | 6847 | short-chain dehydrogenase/reductase SDR | No homolog |
| BCAL2816 | 0.26 | 1E-09 | 2603 | Carboxylesterase | BCAL2816 |
| AU1054_G1996 | 0.27 | 2E-08 | 3948 | alpha/beta hydrolase fold | No homolog |
| AU1054_G5614 | 0.27 | 9E-08 | 6725 | Glyoxalase/bleomycin resistance protein/dioxygenase | No homolog |
| AU1054_G2513 | 0.28 | 6E-04 | 4182 | alpha/beta hydrolase fold | No homolog |
| AU1054_G1426 | 0.28 | 3E-11 | 3084 | plasmid maintenance system killer | No homolog |
| BCAS0022 | 0.29 | 3E-11 | No homolog | No gene annotation | BCAS0022 |
| BCAL0537 | 0.29 | 2E-07 | 3052 | Endonuclease/exonuclease/phosphatase | BCAL0537 |
| AU1054_G2908 | 0.29 | 2E-11 | 6366 | transcriptional regulator, winged helix family | No homolog |
| BCAL1818 | 0.30 | 1E-06 | 1745 | beta-lactamase domain protein | BCAL1818 |
| BCAM2536 | 0.30 | 4E-06 | 5346 | alpha/beta hydrolase fold | BCAM2536 |
| AU1054_G441 | 0.31 | 4E-07 | 6351 | Amidohydrolase 3 | No homolog |
| BCAL0095 | 0.32 | 3E-11 | No homolog | No gene annotation | BCAL0095 |
| BCAM2516 | 0.32 | 1E-11 | 5323 | peptidase M50 | BCAM2516 |
| HI2424_G481 | 0.32 | 3E-08 | 6925 | protein of unknown function UPF0005 | No homolog |
| BCAL0160 | 0.33 | 3E-12 | No homolog | No gene annotation | BCAL0160 |
| AU1054_G2455 | 0.35 | 3E-10 | 3224 | Pirin domain protein domain protein | No homolog |
| BCAL3093 | 0.35 | 3E-03 | 0915 | protein of unknown function UPF0153 | BCAL3093 |
| AU1054_G2526 | 0.35 | 9E-11 | 4168 | alpha/beta hydrolase fold | No homolog |
| AU1054_G1398 | 0.36 | 2E-04 | 3083 | putative plasmid maintenance system antidote protein, XRE family | No homolog |
| AU1054_G2997 | 0.36 | 1E-12 | 1551 | oxidoreductase domain protein | No homolog |
| BCAM1815 | 0.37 | 5E-11 | 4675 | short-chain dehydrogenase/reductase SDR | BCAM1815 |
| BCAL1820 | 0.37 | 2E-07 | 1747 | protein of unknown function DUF81 | BCAL1820 |
| BCAL1386 | 0.38 | 4E-04 | 1375 | alpha/beta hydrolase fold | BCAL1386 |
| BCAM2340 | 0.38 | 1E-04 | 5097 | alpha/beta hydrolase | BCAM2340 |
| BCAM2598 | 0.38 | 2E-07 | No homolog | No gene annotation | BCAM2598 |
| BCAL2764 | 0.39 | 2E-03 | No homolog | No gene annotation | BCAL2764 |
| AU1054_G5595 | 0.39 | 1E-06 | 6705 | amidohydrolase 2 | No homolog |
| AU1054_G1457 | 0.39 | 3E-05 | 6508 | Rieske (2Fe-2S) domain protein | No homolog |
| BCAL0963 | 0.40 | 9E-12 | No homolog | No gene annotation | BCAL0963 |
| AU1054_G5612 | 0.40 | 8E-12 | 6723 | alpha/beta hydrolase fold | No homolog |
| BCAL0808 | 0.40 | 3E-03 | 2795 | Uncharacterised P-loop ATPase protein UPF0042 | BCAL0808 |
| BCAM0092 | 0.41 | 5E-11 | 5791 | esterase/lipase-like protein | BCAM0092 |
| BCAL0622 | 0.41 | 1E-12 | 2970 | Bile acid:sodium symporter | BCAL0622 |
| AU1054_G5515 | 0.42 | 4E-03 | 6615 | BFD domain protein (2Fe-2S)-binding domain protein | No homolog |
| BCAM0534 | 0.42 | 2E-03 | No homolog | No gene annotation | BCAM0534 |
| BCAL1558_J_1 | 0.43 | 2E-12 | 1551 | oxidoreductase domain protein | No homolog |
| BCAS0397 | 0.43 | 2E-04 | No homolog | No gene annotation | BCAS0397 |
| AU1054_G2825 | 0.43 | 2E-05 | 6457 | transport-associated | No homolog |
| BCAL1741 | 0.43 | 2E-07 | No homolog | No gene annotation | BCAL1741 |
| AU1054_G3734 | 0.43 | 4E-10 | 0927 | phage/plasmid primase, P4 family | No homolog |
| IG1_1042213 | 0.43 | 8E-12 | 2651 | regulatory protein RecX | BCAL0954 |
| AU1054_G2837 | 0.43 | 3E-08 | 6443 | short-chain dehydrogenase/reductase SDR | No homolog |
| AU1054_G1445 | 0.44 | 2E-11 | 6521 | transcriptional regulator, winged helix family | No homolog |
| BCAL1742 | 0.44 | 6E-03 | 1684 | extracellular solute-binding protein, family 1 | BCAL1742 |
| AU1054_G2449 | 0.44 | 1E-06 | 3212 | short-chain dehydrogenase/reductase SDR | No homolog |
| BCAM2494 | 0.44 | 5E-11 | 5296 | phosphonoacetate hydrolase | BCAM2494 |
| BCAL0817 | 0.44 | 5E-09 | 2787 | 3-deoxy-D-manno-octulosonate 8-phosphate phosphatase, YrbI family | BCAL0817 |
| AU1054_G2441 | 0.44 | 8E-04 | 3220 | protein of unknown function DUF1486 | No homolog |
| BCAM1960 | 0.44 | 4E-12 | 4759 | metallophosphoesterase | BCAM1960 |
| AU1054_G2897 | 0.45 | 8E-11 | 6377 | short-chain dehydrogenase/reductase SDR | No homolog |
| AU1054_G2839 | 0.45 | 1E-11 | 6440 | transport-associated | No homolog |
| AU1054_G5721 | 0.45 | 2E-07 | 5376 | alpha/beta hydrolase fold | No homolog |
| BCAL3090 | 0.45 | 1E-02 | 0918 | tRNA (guanine-N(7)-)-methyltransferase | BCAL3090 |
| BCAL1398 | 0.46 | 3E-12 | 1389 | Pirin domain protein domain protein | BCAL1398 |
| BCAL1866 | 0.46 | 3E-05 | 1794 | DNA uptake lipoprotein-like protein | BCAL1866 |
| AU1054_G3604 | 0.46 | 1E-04 | 4640 | HAD-superfamily hydrolase, subfamily IA, variant 3 | No homolog |
| BCAL2319 | 0.47 | 2E-13 | 2224 | Protein of unknown function DUF132 | BCAL2319 |
| BCAM1817 | 0.47 | 4E-10 | 4677 | alpha/beta hydrolase fold | BCAM1817 |
| AU1054_G5531 | 0.47 | 4E-04 | 6633 | amidohydrolase 2 | No homolog |
| BCAL1878 | 0.49 | 1E-04 | 1806 | small GTP-binding protein | BCAL1878 |
| BCAM1394 | 0.49 | 2E-06 | 4259 | cysteine dioxygenase type I | BCAM1394 |
| AU1054_G1272 | 0.49 | 1E-11 | 4912 | 2-nitropropane dioxygenase, NPD | No homolog |
| BCAS0082 | 0.49 | 7E-07 | 6139 | hypothetical protein | BCAS0082 |
| BCAL2129 | 0.49 | 2E-04 | 2059 | ABC transporter related | BCAL2129 |
| BCAS0601 | 0.50 | 7E-07 | 6283 | protein of unknown function DUF815 | BCAS0601 |
| BCAM2605 | 0.50 | 1E-03 | 5412 | 2,5-didehydrogluconate reductase | BCAM2605 |
| AU1054_G3698 | > 0.01 | 2E-03 | 0884 | glycosyl transferase, family 2 | No homolog |
| AU1054_G3044 | > 0.01 | 1E-05 | 1572 | Curculin domain protein (mannose-binding) lectin | No homolog |
| AU1054_G1165 | > 0.01 | 1E-06 | 5482 | HipA domain protein | No homolog |
| AU1054_G2896 | > 0.01 | 5E-05 | 6378 | alpha/beta hydrolase fold | No homolog |
|  |  |  |  | ***Function unknown (S)*** |  |
| AU1054_G4283 | 0.02 | 8E-06 | 5839 | hypothetical protein | No homolog |
| HI2424_G4090 | 0.02 | 6E-07 | 6904 | Rhs element Vgr protein | No homolog |
| AU1054_G3382 | 0.03 | 9E-06 | 2306 | integral membrane protein-like protein | No homolog |
| AU1054_G2853 | 0.03 | 1E-05 | 6425 | Linocin_M18 bacteriocin protein | No homolog |
| AU1054_G801 | 0.04 | 2E-07 | 1648 | hypothetical protein | No homolog |
| HI2424_G4059 | 0.05 | 7E-07 | 6862 | alkylhydroperoxidase like protein, AhpD family | No homolog |
| AU1054_G5877 | 0.06 | 6E-04 | 5933 | protein of unknown function DUF925 | No homolog |
| AU1054_G2612 | 0.06 | 8E-06 | 1988 | DinB family protein | No homolog |
| AU1054_G509 | 0.09 | 2E-08 | 4943 | Cupin 2, conserved barrel domain protein | No homolog |
| AU1054_G2850 | 0.11 | 5E-06 | 6428 | membrane protein | No homolog |
| AU1054_G2851 | 0.13 | 4E-08 | 6427 | phosphate-starvation-inducible E | No homolog |
| BCAL1689 | 0.16 | 4E-03 | 1633 | MbtH domain protein | BCAL1689 |
| BCAM2595 | 0.16 | 1E-09 | 5402 | uncharacterized peroxidase-related enzyme | BCAM2595 |
| BCAL2668 | 0.16 | 1E-10 | 2458 | protein of unknown function DUF55 | BCAL2668 |
| BCAM2253A | 0.17 | 6E-10 | 5024 | hypothetical protein | BCAM2253A |
| BCAM2254 | 0.18 | 9E-10 | 5025 | Rhs element Vgr protein | BCAM2254 |
| BCAL2243 | 0.19 | 3E-10 | 2169 | protein of unknown function DUF886 | BCAL2243 |
| BCAL0968 | 0.20 | 5E-10 | 2632 | hypothetical protein | BCAL0968 |
| BCAM2154 | 0.21 | 1E-10 | 4917 | hypothetical protein | BCAM2154 |
| AU1054_G2842 | 0.22 | 2E-09 | 6436 | hypothetical protein | No homolog |
| BCAM2222 | 0.22 | 2E-02 | 4991 | PepSY-associated TM helix domain protein | BCAM2222 |
| BCAL1634 | 0.25 | 5E-06 | 1583 | Glyoxalase/bleomycin resistance protein/dioxygenase | BCAL1634 |
| BCAL2229 | 0.26 | 2E-11 | 2154 | hypothetical protein | BCAL2229 |
| BCAM1867 | 0.26 | 2E-04 | 4723 | MgtC/SapB transporter | BCAM1867 |
| BCAL1819 | 0.27 | 1E-10 | 1746 | protein of unknown function DUF442 | BCAL1819 |
| AU1054_G2813 | 0.27 | 7E-11 | 6470 | PRC-barrel domain protein | No homolog |
| BCAM2159 | 0.28 | 1E-09 | 4923 | conserved hypothetical protein 156 | BCAM2159 |
| BCAL2991 | 0.29 | 3E-05 | 1017 | protein of unknown function UPF0016 | BCAL2991 |
| AU1054_G5524 | 0.29 | 3E-05 | 6626 | protein of unknown function DUF521 | No homolog |
| BCAM2085 | 0.29 | 2E-08 | 4892 | protein of unknown function DUF350 | BCAM2085 |
| BCAL2738 | 0.29 | 1E-11 | 2527 | protein of unknown function DUF192 | BCAL2738 |
| AU1054_G2564 | 0.30 | 5E-11 | 4134 | SH3, type 3 domain protein | No homolog |
| AU1054_G2808 | 0.30 | 1E-09 | 6476 | protein of unknown function DUF892 | No homolog |
| BCAM1461 | 0.30 | 2E-03 | No homolog | No gene annotation | BCAM1461 |
| BCAL2393 | 0.31 | 3E-09 | 2299 | protein of unknown function DUF163 | BCAL2393 |
| BCAM2745 | 0.31 | 4E-08 | No homolog | No gene annotation | BCAM2745 |
| BCAL3487 | 0.32 | 6E-03 | 0020 | alkylhydroperoxidase like protein, AhpD family | BCAL3487 |
| BCAL1981 | 0.32 | 3E-10 | 1908 | protein of unknown function UPF0061 | BCAL1981 |
| AU1054_G802 | 0.32 | 1E-10 | 1647 | hypothetical protein | No homolog |
| AU1054_G5378 | 0.33 | 1E-05 | 4414 | Glyoxalase/bleomycin resistance protein/dioxygenase | No homolog |
| BCAM1108 | 0.33 | 6E-03 | 4005 | Antibiotic biosynthesis monooxygenase | BCAM1108 |
| BCAL2703 | 0.33 | 5E-05 | No homolog | No gene annotation | BCAL2703 |
| AU1054_G2457 | 0.35 | 1E-06 | 3292 | hypothetical protein | No homolog |
| BCAL2123 | 0.36 | 2E-05 | 2053 | protein of unknown function UPF0131 | BCAL2123 |
| AU1054_G2907 | 0.36 | 1E-09 | 6367 | DoxX family protein | No homolog |
| BCAL0395 | 0.36 | 4E-06 | 0530 | adenylate cyclase | BCAL0395 |
| BCAL2930 | 0.37 | 8E-04 | 1067 | hypothetical protein | BCAL2930 |
| BCAL2392 | 0.37 | 2E-06 | 2298 | iojap-like protein | BCAL2392 |
| BCAL0425 | 0.37 | 3E-03 | 3164 | protein of unknown function DUF37 | BCAL0425 |
| BCAL2364 | 0.38 | 4E-10 | 2270 | metallophosphoesterase | BCAL2364 |
| BCAM1166 | 0.38 | 2E-11 | 4060 | protein of unknown function DUF979 | BCAM1166 |
| AU1054_G6078 | 0.38 | 2E-10 | 5572 | hypothetical protein | No homolog |
| BCAM2656 | 0.39 | 5E-05 | 5460 | Activator of Hsp90 ATPase 1 family protein | BCAM2656 |
| BCAM2170 | 0.40 | 2E-11 | 4935 | protein of unknown function DUF218 | BCAM2170 |
| BCAM2309 | 0.41 | 2E-02 | 5068 | protein of unknown function DUF74 | BCAM2309 |
| BCAS0604 | 0.41 | 2E-03 | 6280 | integral membrane protein | BCAS0604 |
| BCAL0939 | 0.42 | 4E-03 | 2663 | glutathione-dependent formaldehyde-activating, GFA | BCAL0939 |
| BCAL2191 | 0.42 | 4E-03 | No homolog | No gene annotation | BCAL2191 |
| BCAM1321 | 0.42 | 9E-03 | 4191 | protein of unknown function DUF344 | BCAM1321 |
| BCAL0588 | 0.42 | 2E-03 | 3004 | hypothetical protein | BCAL0588 |
| BCAM1205 | 0.43 | 2E-03 | 4079 | membrane protein-like protein | BCAM1205 |
| BCAL1535 | 0.43 | 8E-03 | 1528 | membrane protein | BCAL1535 |
| BCAM2216 | 0.43 | 3E-07 | 4984 | protein of unknown function DUF461 | BCAM2216 |
| BCAL2906 | 0.45 | 8E-10 | 1092 | hypothetical protein | BCAL2906 |
| BCAM1479 | 0.45 | 2E-05 | 4351 | hypothetical protein | BCAM1479 |
| AU1054_G5898 | 0.46 | 5E-11 | 5906 | membrane-associated proteins in eicosanoid and glutathione metabolism (MAPEG) | No homolog |
| BCAL1032 | 0.47 | 2E-03 | 1154 | membrane protein | BCAL1032 |
| AU1054_G5523 | 0.47 | 4E-04 | 6625 | protein of unknown function DUF126 | No homolog |
| BCAM0362 | 0.47 | 3E-11 | No homolog | No gene annotation | BCAM0362 |
| BCAL2669 | 0.47 | 7E-03 | 2459 | protein of unknown function DUF541 | BCAL2669 |
| BCAL0815 | 0.48 | 2E-11 | 2789 | OstA family protein | BCAL0815 |
| BCAL0669 | 0.48 | 3E-12 | 2929 | hypothetical protein | BCAL0669 |
| BCAM0796 | 0.48 | 2E-03 | 3759 | protein of unknown function DUF521 | BCAM0796 |
| BCAL0816 | 0.48 | 2E-11 | 2788 | protein of unknown function DUF1239 | BCAL0816 |
| BCAL3212 | 0.48 | 9E-04 | 0790 | YiaAB two helix domain protein | BCAL3212 |
| BCAM1547 | 0.48 | 3E-02 | 4414 | Glyoxalase/bleomycin resistance protein/dioxygenase | BCAM1547 |
| BCAL2763 | 0.49 | 3E-02 | 2549 | hypothetical protein | BCAL2763 |
| AU1054_G3833 | > 0.01 | 2E-04 | 0045 | PilT protein domain protein | No homolog |
| AU1054_G995 | > 0.01 | 7E-06 | 0459 | Rhs element Vgr protein | No homolog |
| AU1054_G979 | > 0.01 | 6E-07 | 0475 | Rhs element Vgr protein | No homolog |
| HI2424_G6715 | > 0.01 | 5E-07 | 3486 | YbaK/prolyl-tRNA synthetase associated region | No homolog |
| AU1054_G4822 | > 0.01 | 6E-07 | 3746 | Rhs element Vgr protein | No homolog |
| AU1054_G2859 | > 0.01 | 3E-06 | 6418 | Rhs element Vgr protein | No homolog |
|  |  |  |  | ***Intergenic regions*** |  |
| IG1_2653432 | 0.04 | 9E-07 | Present? | No gene annotation | No homolog |
| IG1_2117018 | 0.09 | 4E-04 | Present? | No gene annotation | No homolog |
| IG2_3060513 | 0.10 | 2E-07 | Present? | No gene annotation | No homolog |
| IG1_3627057 | 0.14 | 9E-10 | Present? | No gene annotation | No homolog |
| IG1_1014163 | 0.14 | 5E-08 | Present? | No gene annotation | No homolog |
| IG2_450151 | 0.15 | 3E-08 | Present? | No gene annotation | No homolog |
| IG1_3393882 | 0.16 | 3E-08 | Present? | No gene annotation | No homolog |
| IG2_2590456 | 0.16 | 1E-02 | Present? | No gene annotation | No homolog |
| IG1_1028911 | 0.17 | 3E-09 | Present? | No gene annotation | No homolog |
| IG1_677590 | 0.18 | 7E-10 | Present? | No gene annotation | No homolog |
| IG2_433416 | 0.18 | 1E-08 | Present? | No gene annotation | No homolog |
| IG1_1684126 | 0.18 | 1E-08 | Present? | No gene annotation | No homolog |
| IG1_899543 | 0.18 | 3E-09 | Present? | No gene annotation | No homolog |
| IG2_2899303 | 0.20 | 4E-08 | Present? | No gene annotation | No homolog |
| IG2_2952067 | 0.20 | 3E-09 | Present? | No gene annotation | No homolog |
| IG1_2622531 | 0.21 | 1E-10 | Present? | No gene annotation | No homolog |
| IG1_2681109 | 0.22 | 1E-10 | Present? | No gene annotation | No homolog |
| IG2_1931541 | 0.23 | 2E-04 | Present? | No gene annotation | No homolog |
| IG2_2274945 | 0.24 | 6E-10 | Present? | No gene annotation | No homolog |
| IG2_219668 | 0.24 | 4E-03 | Present? | No gene annotation | No homolog |
| IG2_3137937 | 0.24 | 7E-09 | Present? | No gene annotation | No homolog |
| IG1_516060 | 0.25 | 4E-06 | Present? | No gene annotation | No homolog |
| IG1_1043076 | 0.25 | 2E-10 | Present? | No gene annotation | No homolog |
| IG3_523151 | 0.26 | 7E-11 | Present? | No gene annotation | No homolog |
| IG2_2876949 | 0.26 | 1E-10 | Present? | No gene annotation | No homolog |
| IG1_3016273 | 0.26 | 2E-02 | Present? | No gene annotation | No homolog |
| IG1_3477390 | 0.27 | 1E-05 | Present? | No gene annotation | No homolog |
| IG2_1975919 | 0.27 | 2E-08 | Present? | No gene annotation | No homolog |
| IG1_2570370 | 0.28 | 4E-09 | Present? | No gene annotation | No homolog |
| IG2_2323998 | 0.28 | 4E-09 | Present? | No gene annotation | No homolog |
| IG1_185981 | 0.28 | 1E-06 | Present? | No gene annotation | No homolog |
| IG1_926490 | 0.28 | 6E-08 | Present? | No gene annotation | No homolog |
| IG1_422927 | 0.29 | 3E-12 | Present? | No gene annotation | No homolog |
| IG1_161120 | 0.29 | 6E-04 | Present? | No gene annotation | No homolog |
| IG2_444113 | 0.30 | 8E-03 | Present? | No gene annotation | No homolog |
| IG1_2874215 | 0.30 | 2E-05 | Present? | No gene annotation | No homolog |
| IG2_2803864 | 0.30 | 6E-06 | Present? | No gene annotation | No homolog |
| IG2_2589126 | 0.30 | 3E-02 | Present? | No gene annotation | No homolog |
| IG2_1896347 | 0.31 | 2E-09 | Present? | No gene annotation | No homolog |
| IG3_654635 | 0.31 | 1E-05 | Present? | No gene annotation | No homolog |
| IG1_2712162 | 0.32 | 7E-12 | Present? | No gene annotation | No homolog |
| IG1_2936760 | 0.32 | 2E-03 | Present? | No gene annotation | No homolog |
| IG2_1565086 | 0.32 | 3E-03 | Present? | No gene annotation | No homolog |
| IG2_2970010 | 0.33 | 5E-12 | Present? | No gene annotation | No homolog |
| IG1_3507377 | 0.33 | 4E-06 | Present? | No gene annotation | No homolog |
| IG2_2085682 | 0.33 | 3E-05 | Present? | No gene annotation | No homolog |
| IG1_2538832 | 0.33 | 1E-05 | Present? | No gene annotation | No homolog |
| IG1_2420168 | 0.33 | 2E-03 | Present? | No gene annotation | No homolog |
| IG2_2440784 | 0.34 | 2E-03 | Present? | No gene annotation | No homolog |
| IG3_798183 | 0.35 | 3E-03 | Present? | No gene annotation | No homolog |
| IG2_214633 | 0.36 | 3E-03 | Present? | No gene annotation | No homolog |
| IG1_500384 | 0.36 | 2E-09 | Present? | No gene annotation | No homolog |
| IG1_1337938 | 0.36 | 8E-04 | Present? | No gene annotation | No homolog |
| IG1_368927 | 0.36 | 2E-07 | Present? | No gene annotation | No homolog |
| IG1_625858 | 0.36 | 2E-05 | Present? | No gene annotation | No homolog |
| IG2_36215 | 0.36 | 2E-03 | Present? | No gene annotation | No homolog |
| IG1_659402 | 0.37 | 2E-04 | Present? | No gene annotation | No homolog |
| IG2_1946387 | 0.37 | 2E-11 | Present? | No gene annotation | No homolog |
| IG1_1928097 | 0.38 | 3E-04 | Present? | No gene annotation | No homolog |
| IG1_640011 | 0.38 | 4E-03 | Present? | No gene annotation | No homolog |
| IG1_435852 | 0.38 | 5E-04 | Present? | No gene annotation | No homolog |
| IG1_2235333 | 0.39 | 2E-11 | Present? | No gene annotation | No homolog |
| IG1_2434003 | 0.39 | 1E-10 | Present? | No gene annotation | No homolog |
| IG2_2917147 | 0.40 | 9E-06 | Present? | No gene annotation | No homolog |
| IG1_3199779 | 0.40 | 1E-05 | Present? | No gene annotation | No homolog |
| IG1_2003331 | 0.40 | 3E-07 | Present? | No gene annotation | No homolog |
| IG2_2841811 | 0.40 | 6E-07 | Present? | No gene annotation | No homolog |
| IG1_568652 | 0.41 | 4E-06 | Present? | No gene annotation | Intergenic region, chromosome 1 |
| IG2_2240134 | 0.41 | 2E-03 | Present? | No gene annotation | No homolog |
| IG2_3096444 | 0.41 | 1E-11 | Present? | No gene annotation | No homolog |
| IG1_2130505 | 0.41 | 2E-11 | Present? | No gene annotation | No homolog |
| IG1_2379897 | 0.41 | 1E-04 | Present? | No gene annotation | No homolog |
| IG1_2684447 | 0.42 | 6E-03 | Present? | No gene annotation | No homolog |
| IG2_58076 | 0.42 | 8E-10 | Present? | No gene annotation | No homolog |
| IG1_516822 | 0.42 | 4E-10 | Present? | No gene annotation | No homolog |
| IG1_3297972 | 0.42 | 2E-03 | Present? | No gene annotation | No homolog |
| IG1_3867113 | 0.43 | 2E-06 | Present? | No gene annotation | No homolog |
| IG2_2008060 | 0.43 | 3E-11 | Present? | No gene annotation | No homolog |
| IG1_3025580 | 0.43 | 8E-04 | Present? | No gene annotation | No homolog |
| IG1_2017343 | 0.43 | 1E-11 | Present? | No gene annotation | No homolog |
| IG2_2317170 | 0.44 | 2E-10 | Present? | No gene annotation | No homolog |
| IG1_2283993 | 0.44 | 2E-03 | Present? | No gene annotation | No homolog |
| IG1_2676354 | 0.44 | 2E-12 | Present? | No gene annotation | No homolog |
| IG1_2616144 | 0.44 | 2E-12 | Present? | No gene annotation | No homolog |
| IG1_2644135 | 0.45 | 2E-03 | Present? | No gene annotation | No homolog |
| IG2_2536473 | 0.45 | 6E-04 | Present? | No gene annotation | No homolog |
| IG2_2676155 | 0.45 | 2E-04 | Present? | No gene annotation | No homolog |
| IG1_2087559 | 0.45 | 8E-06 | Present? | No gene annotation | No homolog |
| IG1_786937 | 0.45 | 7E-03 | Present? | No gene annotation | No homolog |
| IG1_2389580 | 0.46 | 4E-04 | Present? | No gene annotation | No homolog |
| IG1_2657870 | 0.46 | 4E-07 | Present? | No gene annotation | Intergenic region, chromosome 1 |
| IG1_1161731 | 0.46 | 2E-05 | Present? | No gene annotation | No homolog |
| IG1_2965090 | 0.46 | 2E-02 | Present? | No gene annotation | No homolog |
| IG1_335692 | 0.46 | 2E-11 | Present? | No gene annotation | No homolog |
| IG1_3503400 | 0.47 | 1E-03 | Present? | No gene annotation | No homolog |
| IG1_561836 | 0.47 | 1E-02 | Present? | No gene annotation | No homolog |
| IG1_752786 | 0.47 | 3E-04 | Present? | No gene annotation | No homolog |
| IG1_3281141 | 0.48 | 2E-02 | Present? | No gene annotation | No homolog |
| IG1_953334 | 0.48 | 9E-13 | Present? | No gene annotation | No homolog |
| IG2_1377787 | 0.48 | 1E-02 | Present? | No gene annotation | No homolog |
| IG1_2947629 | 0.49 | 2E-07 | Present? | No gene annotation | No homolog |
| IG2_2437623 | 0.49 | 2E-02 | Present? | No gene annotation | No homolog |
| IG1_253372 | 0.49 | 3E-03 | Present? | No gene annotation | No homolog |
| IG1_800614 | 0.49 | 2E-05 | Present? | No gene annotation | No homolog |
| IG1_3823329 | 0.49 | 8E-03 | Present? | No gene annotation | No homolog |
| IG2_145734 | 0.49 | 4E-03 | Present? | No gene annotation | No homolog |
| IG1_2671514 | 0.50 | 1E-04 | Present? | No gene annotation | No homolog |
| IG2_2018202 | 0.50 | 3E-09 | Present? | No gene annotation | No homolog |
|  |  |  |  | ***No assigned COG*** |  |
| AU1054_G2375 | 0.02 | 8E-04 | 0223 | hypothetical protein | No homolog |
| BCAS0154 | 0.02 | 5E-07 | 6079 | hypothetical protein | BCAS0154 |
| HI2424_G6710 | 0.02 | 8E-05 | No homolog | No gene annotation | No homolog |
| AU1054_G2379 | 0.02 | 1E-05 | 0230 | hypothetical protein | No homolog |
| AU1054_G978 | 0.02 | 6E-07 | 0476 | hypothetical protein | No homolog |
| AU1054_G2320 | 0.03 | 1E-06 | 0222 | hypothetical protein | No homolog |
| AU1054_G6263 | 0.03 | 1E-04 | Multiple hits | No gene annotation | No homolog |
| AU1054_G6084 | 0.03 | 7E-05 | 5566 | hypothetical protein | No homolog |
| AU1054_G3476 | 0.03 | 5E-04 | 2344 | hypothetical protein | No homolog |
| AU1054_G6186 | 0.03 | 1E-06 | Multiple hits | No gene annotation | No homolog |
| AU1054_G975 | 0.03 | 4E-07 | 0479 | hypothetical protein | No homolog |
| HI2424_G4054 | 0.03 | 7E-05 | 6856 | plasmid pRiA4b ORF-3 family protein | No homolog |
| AU1054_G5129 | 0.03 | 3E-04 | No homolog | No gene annotation | No homolog |
| AU1054_G6241 | 0.03 | 5E-07 | Multiple hits | No gene annotation | No homolog |
| AU1054_G291 | 0.03 | 3E-05 | Multiple hits | No gene annotation | No homolog |
| AU1054_G1884 | 0.03 | 4E-07 | 0453 | hypothetical protein | No homolog |
| HI2424_G6381 | 0.04 | 4E-07 | 0191 | hypothetical protein | No homolog |
| HI2424_G6189 | 0.04 | 2E-06 | No homolog | No gene annotation | No homolog |
| AU1054_G3940 | 0.04 | 3E-07 | Multiple hits | No gene annotation | No homolog |
| HI2424_G6235 | 0.04 | 7E-07 | 0193 | hypothetical protein | No homolog |
| AU1054_G1093 | 0.04 | 3E-07 | 0634 | hypothetical protein | No homolog |
| AU1054_G5856 | 0.04 | 9E-07 | Multiple hits | No gene annotation | No homolog |
| HI2424_G4076 | 0.04 | 2E-07 | 6884 | hypothetical protein | No homolog |
| AU1054_G4023 | 0.04 | 2E-06 | 2073 | hypothetical protein | No homolog |
| AU1054_G6041 | 0.04 | 3E-07 | 5620 | hypothetical protein | No homolog |
| AU1054_G6087 | 0.04 | 9E-07 | No homolog | No gene annotation | No homolog |
| AU1054_G4902 | 0.04 | 1E-04 | No homolog | No gene annotation | No homolog |
| HI2424_G6380 | 0.05 | 5E-07 | 0192 | hypothetical protein | No homolog |
| AU1054_G2319 | 0.05 | 4E-07 | 0221 | hypothetical protein | No homolog |
| HI2424_G4112 | 0.05 | 6E-07 | 6894 | hypothetical protein | No homolog |
| HI2424_G6991 | 0.05 | 5E-06 | 3491 | hypothetical protein | No homolog |
| AU1054_G6189 | 0.05 | 2E-06 | 3384 | hypothetical protein | No homolog |
| AU1054_G3789 | 0.05 | 1E-07 | 0904 | hypothetical protein | No homolog |
| AU1054_G2292 | 0.05 | 2E-07 | 2640 | hypothetical protein | No homolog |
| AU1054_G1560 | 0.05 | 5E-07 | No homolog | No gene annotation | No homolog |
| AU1054_G2385 | 0.05 | 2E-04 | Multiple hits | No gene annotation | No homolog |
| AU1054_G3134 | 0.05 | 6E-07 | Multiple hits | No gene annotation | No homolog |
| AU1054_G972 | 0.05 | 7E-07 | 0482 | hypothetical protein | No homolog |
| AU1054_G1874 | 0.05 | 1E-06 | Multiple hits | No gene annotation | No homolog |
| HI2424_G4077 | 0.05 | 1E-05 | 6885 | hypothetical protein | No homolog |
| AU1054_G2326 | 0.05 | 7E-03 | 0234 | hypothetical protein | No homolog |
| AU1054_G1546 | 0.05 | 1E-05 | 3668 | hypothetical protein | No homolog |
| AU1054_G3477 | 0.05 | 2E-07 | 2372 | hypothetical protein | No homolog |
| AU1054_G2458 | 0.06 | 2E-06 | 3261 | hypothetical protein | No homolog |
| AU1054_G2849 | 0.06 | 4E-04 | 6429 | hypothetical protein | No homolog |
| AU1054_G1883 | 0.06 | 3E-06 | 0452 | hypothetical protein | No homolog |
| AU1054_G1285 | 0.06 | 3E-07 | No homolog | No gene annotation | No homolog |
| AU1054_G5751 | 0.06 | 1E-07 | 5345 | hypothetical protein | No homolog |
| AU1054_G4132 | 0.06 | 5E-05 | 2188 | hypothetical protein | No homolog |
| AU1054_G6125 | 0.06 | 5E-07 | Multiple hits | No gene annotation | No homolog |
| HI2424_G6377 | 0.06 | 5E-07 | 0208 | hypothetical protein | No homolog |
| AU1054_G6062 | 0.06 | 4E-05 | 5597 | hypothetical protein | No homolog |
| BCAL0510 | 0.07 | 6E-08 | 3080 | hypothetical protein | BCAL0510 |
| AU1054_G1706 | 0.07 | 6E-08 | Multiple hits | No gene annotation | No homolog |
| AU1054_G6469 | 0.07 | 8E-06 | No homolog | No gene annotation | No homolog |
| AU1054_G2406 | 0.07 | 1E-05 | Multiple hits | No gene annotation | No homolog |
| AU1054_G6516 | 0.07 | 2E-04 | No homolog | No gene annotation | No homolog |
| AU1054_G3120 | 0.07 | 1E-07 | Multiple hits | No gene annotation | No homolog |
| AU1054_G324_4 | 0.08 | 7E-05 | No homolog | No gene annotation | No homolog |
| AU1054_G759 | 0.08 | 9E-08 | 0523 | hypothetical protein | No homolog |
| AU1054_G129 | 0.08 | 2E-08 | 4660 | hypothetical protein | No homolog |
| AU1054_G2316 | 0.08 | 1E-07 | Multiple hits | No gene annotation | No homolog |
| AU1054_G3466 | 0.08 | 1E-03 | 2370 | hypothetical protein | No homolog |
| AU1054_G151 | 0.08 | 2E-05 | 5978 | hypothetical protein | No homolog |
| AU1054_G65 | 0.08 | 8E-06 | Multiple hits | No gene annotation | No homolog |
| AU1054_G2373 | 0.09 | 7E-08 | 0215 | hypothetical protein | No homolog |
| AU1054_G4610 | 0.09 | 1E-07 | 1322 | hypothetical protein | No homolog |
| AU1054_G2860 | 0.09 | 1E-07 | 6417 | hypothetical protein | No homolog |
| AU1054_G324_2 | 0.09 | 2E-03 | No homolog | No gene annotation | No homolog |
| AU1054_G5432 | 0.09 | 5E-08 | 1787 | hypothetical protein | No homolog |
| AU1054_G3938 | 0.09 | 3E-06 | Multiple hits | No gene annotation | No homolog |
| AU1054_G4570 | 0.10 | 6E-06 | 1370 | hypothetical protein | No homolog |
| AU1054_G4672 | 0.10 | 2E-05 | 1346 | hypothetical protein | No homolog |
| HI2424_G4086 | 0.10 | 3E-08 | 6899 | hypothetical protein | No homolog |
| AU1054_G2888 | 0.10 | 3E-08 | 6386 | hypothetical protein | No homolog |
| AU1054_G435 | 0.10 | 7E-07 | 3992 | hypothetical protein | No homolog |
| HI2424_G6735 | 0.11 | 3E-08 | 3464 | hypothetical protein | No homolog |
| AU1054_G3797 | 0.11 | 5E-09 | 0931 | hypothetical protein | No homolog |
| AU1054_G2845 | 0.11 | 3E-08 | 6433 | hypothetical protein | No homolog |
| AU1054_G3638 | 0.11 | 1E-08 | 5503 | hypothetical protein | No homolog |
| AU1054_G4419 | 0.11 | 4E-05 | No homolog | No gene annotation | No homolog |
| AU1054_G1016 | 0.11 | 2E-06 | 6552 | hypothetical protein | No homolog |
| BCAM2228_J_1 | 0.12 | 1E-02 | No homolog | No gene annotation | BCAM2228 |
| AU1054_G182 | 0.12 | 3E-04 | 4842 | hypothetical protein | No homolog |
| AU1054_G5652 | 0.12 | 1E-06 | 6691 | hypothetical protein | No homolog |
| AU1054_G2882 | 0.12 | 9E-09 | 6392 | hypothetical protein | No homolog |
| HI2424_G4084 | 0.12 | 9E-09 | 6896 | hypothetical protein | No homolog |
| BCAL3207 | 0.12 | 3E-08 | No homolog | No gene annotation | No homolog |
| AU1054_G2862 | 0.12 | 2E-06 | 6414 | hypothetical protein | No homolog |
| AU1054_G6492 | 0.12 | 9E-06 | 5596 | hypothetical protein | No homolog |
| BCAS0155 | 0.12 | 1E-08 | 6078 | hypothetical protein | BCAS0155 |
| HI2424_G6232 | 0.13 | 1E-04 | 0197 | hypothetical protein | No homolog |
| AU1054_G2979 | 0.13 | 4E-05 | 1573 | C-5 cytosine-specific DNA methylase | No homolog |
| BCAL1698 | 0.13 | 6E-05 | No homolog | No gene annotation | BCAL1698 |
| AU1054_G5896 | 0.13 | 1E-05 | No homolog | No gene annotation | No homolog |
| AU1054_G2029 | 0.13 | 6E-05 | No homolog | No gene annotation | No homolog |
| AU1054_G6279 | 0.13 | 3E-09 | 5209 | hypothetical protein | No homolog |
| AU1054_G4431 | 0.14 | 4E-09 | No homolog | No gene annotation | No homolog |
| BCAS0682 | 0.14 | 9E-10 | Multiple hits | No gene annotation | Multiple hits |
| AU1054_G6067 | 0.15 | 1E-03 | 5591 | 17 kDa surface antigen | No homolog |
| AU1054_G5382 | 0.15 | 2E-09 | 4499 | hypothetical protein | No homolog |
| AU1054_G3035 | 0.15 | 6E-09 | 1574 | hypothetical protein | No homolog |
| AU1054_G6010 | 0.15 | 3E-07 | 5653 | glycoside hydrolase, family 5 | No homolog |
| AU1054_G436 | 0.15 | 1E-04 | 3997 | hypothetical protein | No homolog |
| AU1054_G2413 | 0.15 | 6E-10 | Multiple hits | No gene annotation | No homolog |
| BCAM2164 | 0.15 | 4E-04 | No homolog | No gene annotation | BCAM2164 |
| AU1054_G2370 | 0.15 | 6E-10 | 0210 | hypothetical protein | No homolog |
| BCAM2223 | 0.16 | 2E-02 | 4992 | hypothetical protein | BCAM2223 |
| BCAM2504 | 0.16 | 8E-11 | 5306 | hypothetical protein | BCAM2504 |
| BCAS0183 | 0.17 | 1E-08 | 6046 | hypothetical protein | BCAS0183 |
| AU1054_G6539 | 0.17 | 1E-05 | No homolog | No gene annotation | No homolog |
| BCAL3205C | 0.17 | 5E-09 | 0796 | hypothetical protein | BCAL3205C |
| AU1054_G4353 | 0.17 | 4E-10 | 5760 | hypothetical protein | No homolog |
| AU1054_G5974 | 0.17 | 3E-10 | No homolog | No gene annotation | No homolog |
| AU1054_G3418 | 0.17 | 2E-10 | 2342 | hypothetical protein | No homolog |
| AU1054_G2844 | 0.17 | 2E-06 | 6434 | hypothetical protein | No homolog |
| AU1054_G5522 | 0.18 | 8E-06 | 6624 | FAD dependent oxidoreductase | No homolog |
| AU1054_G3796 | 0.18 | 7E-10 | 0925 | hypothetical protein | No homolog |
| AU1054_G3735 | 0.18 | 5E-10 | 0929 | hypothetical protein | No homolog |
| AU1054_G4554 | 0.18 | 6E-08 | 1388 | hypothetical protein | No homolog |
| HI2424_G6167 | 0.18 | 3E-10 | 6821 | conserved hypothetical lipoprotein | No homolog |
| BCAL0783 | 0.19 | 6E-10 | 2819 | cyd operon protein YbgT | BCAL0783 |
| AU1054_G6525 | 0.19 | 6E-05 | 5217 | hypothetical protein | No homolog |
| AU1054_G2454 | 0.19 | 1E-10 | Multiple hits | No gene annotation | No homolog |
| AU1054_G209 | 0.19 | 1E-06 | 4842 | hypothetical protein | No homolog |
| AU1054_G2433 | 0.20 | 6E-08 | No homolog | No gene annotation | No homolog |
| BCAM0394 | 0.20 | 5E-07 | No homolog | No gene annotation | BCAM0394 |
| AU1054_G1280 | 0.20 | 1E-07 | 4866 | hypothetical protein | No homolog |
| AU1054_G2409 | 0.20 | 9E-05 | Multiple hits | No gene annotation | No homolog |
| AU1054_G229 | 0.21 | 3E-07 | 0294 | hypothetical protein | No homolog |
| AU1054_G3043 | 0.21 | 3E-10 | 1575 | hypothetical protein | No homolog |
| BCAM0946a | 0.21 | 3E-11 | 3913 | hypothetical protein | BCAM0946a |
| AU1054_G5610 | 0.21 | 6E-08 | 6721 | NIPSNAP family containing protein | No homolog |
| AU1054_G2383 | 0.21 | 8E-11 | No homolog | No gene annotation | No homolog |
| AU1054_G2974 | 0.22 | 3E-07 | 1582 | hypothetical protein | No homolog |
| AU1054_G2430 | 0.23 | 7E-04 | No homolog | No gene annotation | No homolog |
| BCAL0653 | 0.23 | 1E-10 | 2937 | hypothetical protein | BCAL0653 |
| AU1054_G2462 | 0.23 | 3E-09 | No homolog | No gene annotation | No homolog |
| BCAL0683 | 0.23 | 1E-10 | 2915 | hypothetical protein | BCAL0683 |
| AU1054_G5137 | 0.23 | 2E-09 | 6196 | hypothetical protein | No homolog |
| AU1054_G5581 | 0.23 | 7E-09 | 6690 | Ethyl tert-butyl ether degradation EthD | No homolog |
| BCAM0024 | 0.24 | 3E-06 | No homolog | No gene annotation | No homolog |
| HI2424_G6237 | 0.24 | 2E-09 | 0186 | hypothetical protein | No homolog |
| AU1054_G4582 | 0.24 | 3E-11 | No homolog | No gene annotation | No homolog |
| BCAM2781 | 0.25 | 7E-11 | 5625 | 17 kDa surface antigen | BCAM2781 |
| BCAM0192 | 0.25 | 2E-02 | 3184 | hypothetical protein | BCAM0192 |
| AU1054_G1574 | 0.25 | 6E-12 | 4074 | hypothetical protein | No homolog |
| AU1054_G6493 | 0.25 | 3E-09 | 5589 | hypothetical protein | No homolog |
| BCAL2998 | 0.25 | 9E-04 | No homolog | No gene annotation | BCAL2998 |
| BCAM2709 | 0.26 | 2E-06 | 5524 | hypothetical protein | BCAM2709 |
| AU1054_G2819 | 0.26 | 9E-11 | 6463 | hypothetical protein | No homolog |
| BCAS0116 | 0.26 | 1E-08 | 6109 | hypothetical protein | BCAS0116 |
| AU1054_G2931 | 0.26 | 9E-12 | 1629 | fimbrial protein | No homolog |
| AU1054_G2805 | 0.26 | 2E-02 | 6479 | protein of unknown function DUF1328 | No homolog |
| AU1054_G2456 | 0.26 | 1E-04 | 3240 | hypothetical protein | No homolog |
| BCALr1104 | 0.26 | 2E-10 | No homolog | No gene annotation | BCALr1104 |
| AU1054_G2498 | 0.27 | 2E-05 | 4196 | hypothetical protein | No homolog |
| AU1054_G3787 | 0.27 | 4E-12 | 0923 | hypothetical protein | No homolog |
| AU1054_G4432 | 0.27 | 3E-11 | No homolog | No gene annotation | No homolog |
| AU1054_G2321 | 0.27 | 1E-07 | 0226 | hypothetical protein | No homolog |
| AU1054_G3188 | 0.28 | 1E-10 | 1236 | hypothetical protein | No homolog |
| BCAM0432 | 0.28 | 1E-10 | 3399 | hypothetical protein | BCAM0432 |
| AU1054_G6489 | 0.28 | 2E-11 | 5627 | hypothetical protein | No homolog |
| AU1054_G5068 | 0.28 | 3E-04 | 6158 | protein of unknown function DUF1330 | No homolog |
| AU1054_G3799 | 0.28 | 7E-05 | 0981 | hypothetical protein | No homolog |
| HI2424_G4073 | 0.28 | 9E-12 | 6880 | hypothetical protein | No homolog |
| AU1054_G1306 | 0.28 | 2E-11 | 4752 | hypothetical protein | No homolog |
| BCAL2362 | 0.29 | 1E-06 | 2268 | hypothetical protein | BCAL2362 |
| AU1054_G2847 | 0.29 | 6E-03 | No homolog | No gene annotation | No homolog |
| BCAL0966 | 0.29 | 2E-06 | 2634 | hypothetical protein | BCAL0966 |
| HI2424_G6170 | 0.29 | 6E-05 | 6826 | protein of unknown function DUF1173 | No homolog |
| BCAL2463 | 0.29 | 4E-10 | No homolog | No gene annotation | BCAL2463 |
| BCAS0184 | 0.29 | 3E-05 | No homolog | No gene annotation | No homolog |
| AU1054_G6073 | 0.29 | 3E-08 | 5579 | hypothetical protein | No homolog |
| AU1054_G2396 | 0.30 | 9E-08 | 3282 | hypothetical protein | No homolog |
| BCAS0454 | 0.30 | 3E-09 | No homolog | No gene annotation | No homolog |
| BCAL2709 | 0.30 | 7E-12 | No homolog | No gene annotation | BCAL2709 |
| BCAL0193 | 0.31 | 5E-03 | 0305 | hypothetical protein | BCAL0193 |
| BCAM0493 | 0.31 | 1E-07 | No homolog | No gene annotation | BCAM0493 |
| AU1054_G2810 | 0.31 | 2E-08 | 6473 | hypothetical protein | No homolog |
| AU1054_G2325 | 0.31 | 8E-06 | 0233 | hypothetical protein | No homolog |
| BCAM2603 | 0.32 | 6E-08 | 5410 | hypothetical protein | BCAM2603 |
| BCAM0633 | 0.32 | 6E-03 | 3604 | hypothetical protein | BCAM0633 |
| pBCA005 | 0.32 | 7E-10 | Multiple hits | No gene annotation | pBCA005 |
| BCAL0538 | 0.32 | 7E-12 | 3051 | hypothetical protein | BCAL0538 |
| AU1054_G3040 | 0.32 | 1E-05 | 1625 | hypothetical protein | No homolog |
| BCAM2010 | 0.32 | 3E-02 | 4811 | hypothetical protein | BCAM2010 |
| AU1054_G6245 | 0.33 | 7E-12 | No homolog | No gene annotation | No homolog |
| BCAL0942 | 0.33 | 2E-03 | 2660 | hypothetical protein | BCAL0942 |
| AU1054_G2823 | 0.33 | 1E-08 | 6459 | hypothetical protein | No homolog |
| BCAL3515 | 0.33 | 4E-11 | 0051 | hypothetical protein | BCAL3515 |
| BCAM0600 | 0.34 | 8E-08 | No homolog | No gene annotation | No homolog |
| BCAM2228_J_0 | 0.34 | 2E-03 | No homolog | No gene annotation | BCAM2228 |
| BCAM1863 | 0.34 | 9E-04 | 4719 | hypothetical protein | BCAM1863 |
| BCAM2438 | 0.35 | 6E-10 | 5245 | hypothetical protein | BCAM2438 |
| BCAM2050 | 0.35 | 4E-12 | 4849 | hypothetical protein | BCAM2050 |
| BCAM0634 | 0.35 | 6E-03 | 3605 | hypothetical protein | BCAM0634 |
| AU1054_G2806 | 0.35 | 3E-09 | 6478 | hypothetical protein | No homolog |
| BCAS0640 | 0.35 | 1E-03 | 6249 | hypothetical protein | BCAS0640 |
| BCAL2361 | 0.36 | 1E-11 | 2267 | hypothetical protein | BCAL2361 |
| AU1054_G5978 | 0.36 | 3E-02 | 5915 | hypothetical protein | No homolog |
| BCAM0090 | 0.36 | 3E-06 | 5789 | hypothetical protein | BCAM0090 |
| AU1054_G5763 | 0.36 | 3E-03 | 5333 | hypothetical protein | No homolog |
| BCAL2746A | 0.36 | 3E-12 | 2535 | hypothetical protein | BCAL2746A |
| HI2424_G6166 | 0.36 | 3E-04 | 6820 | hypothetical protein | No homolog |
| BCAM2090 | 0.36 | 4E-05 | No homolog | No gene annotation | BCAM2090 |
| AU1054_G4669 | 0.36 | 1E-11 | No homolog | No gene annotation | No homolog |
| BCAM0837 | 0.37 | 2E-12 | No homolog | No gene annotation | BCAM0837 |
| HI2424_G6174 | 0.37 | 7E-05 | 6795 | hypothetical protein | No homolog |
| BCAL0858 | 0.37 | 6E-03 | No homolog | No gene annotation | BCAL0858 |
| BCAL0680 | 0.38 | 4E-03 | 2918 | hypothetical protein | BCAL0680 |
| BCAM2207 | 0.38 | 1E-03 | 4973 | hypothetical protein | BCAM2207 |
| BCAS0063 | 0.38 | 6E-12 | No homolog | No gene annotation | BCAS0063 |
| BCAM0373 | 0.39 | 4E-05 | 3361 | hypothetical protein | BCAM0373 |
| BCAL3095 | 0.39 | 2E-12 | 0913 | hypothetical protein | BCAL3095 |
| BCAM0174 | 0.39 | 4E-08 | 3167 | hypothetical protein | BCAM0174 |
| BCAL0931 | 0.40 | 4E-03 | 2672 | hypothetical protein | BCAL0931 |
| HI2424_G6161 | 0.40 | 9E-09 | 6809 | hypothetical protein | No homolog |
| BCAL1746 | 0.40 | 1E-05 | 1688 | hypothetical protein | BCAL1746 |
| BCAL0786 | 0.40 | 2E-03 | 2816 | hypothetical protein | BCAL0786 |
| BCAM1354 | 0.40 | 8E-12 | No homolog | No gene annotation | BCAM1354 |
| BCAM1058 | 0.41 | 1E-08 | No homolog | No gene annotation | BCAM1058 |
| HI2424_G6386 | 0.41 | 1E-09 | 0169 | hypothetical protein | No homolog |
| BCAL1413A | 0.41 | 7E-03 | 1406 | hypothetical protein | BCAL1413A |
| AU1054_G4416 | 0.41 | 1E-07 | Multiple hits | No gene annotation | No homolog |
| AU1054_G6074 | 0.41 | 1E-02 | 5577 | hypothetical protein | No homolog |
| BCAM1374 | 0.41 | 2E-08 | 4239 | hypothetical protein | BCAM1374 |
| AU1054_G5212 | 0.41 | 9E-05 | 4413 | hypothetical protein | No homolog |
| BCAL0072 | 0.41 | 1E-03 | 0143 | hypothetical protein | BCAL0072 |
| BCAM0542 | 0.42 | 3E-05 | 3516 | hypothetical protein | BCAM0542 |
| AU1054_G1473 | 0.42 | 5E-09 | 6487 | hypothetical protein | No homolog |
| BCAL0836 | 0.43 | 5E-12 | 2768 | 3-hydroxylacyl-(acyl carrier protein) dehydratase-like protein | BCAL0836 |
| BCAL1687 | 0.43 | 5E-03 | 1631 | hypothetical protein | BCAL1687 |
| AU1054_G5604 | 0.43 | 2E-08 | No homolog | No gene annotation | No homolog |
| BCAL1103 | 0.43 | 2E-03 | 1222 | hypothetical protein | BCAL1103 |
| AU1054_G2891 | 0.44 | 4E-05 | 6383 | hypothetical protein | No homolog |
| BCAL0927 | 0.44 | 1E-03 | 2676 | hypothetical protein | BCAL0927 |
| HI2424_G6148 | 0.44 | 2E-06 | 6793 | hypothetical protein | No homolog |
| BCAM0431 | 0.44 | 2E-04 | No homolog | No gene annotation | No homolog |
| BCAL0955 | 0.44 | 2E-02 | 2650 | hypothetical protein | BCAL0955 |
| HI2424_G6175 | 0.45 | 2E-05 | 6812 | hypothetical protein | No homolog |
| BCAM2052 | 0.46 | 2E-03 | 4851 | hypothetical protein | BCAM2052 |
| AU1054_G6149 | 0.46 | 8E-10 | No homolog | No gene annotation | No homolog |
| BCAL3002 | 0.46 | 4E-03 | 1009 | hypothetical protein | BCAL3002 |
| BCAM2830 | 0.47 | 6E-05 | 5678 | hypothetical protein | BCAM2830 |
| BCAS0633 | 0.47 | 3E-03 | No homolog | No gene annotation | No homolog |
| BCAS0645_J_0 | 0.47 | 5E-04 | No homolog | No gene annotation | BCAS0645 |
| BCAL2473 | 0.47 | 1E-12 | 2395 | hypothetical protein | BCAL2473 |
| HI2424_G192 | 0.47 | 4E-02 | 6937 | hypothetical protein | No homolog |
| AU1054_G6002 | 0.47 | 3E-05 | 5661 | hypothetical protein | No homolog |
| BCAMr1592 | 0.47 | 4E-03 | No homolog | No gene annotation | BCAMr1592 |
| BCAL2398 | 0.47 | 4E-04 | No homolog | No gene annotation | BCAL2398 |
| BCAM2467a | 0.47 | 2E-02 | Multiple hits | No gene annotation | No homolog |
| BCAL0434 | 0.48 | 2E-02 | 3155 | hypothetical protein | BCAL0434 |
| BCALr2665 | 0.48 | 1E-02 | No homolog | No gene annotation | BCALr2665 |
| BCAL3007 | 0.48 | 2E-03 | 1004 | hypothetical protein | BCAL3007 |
| BCAL2667 | 0.48 | 5E-12 | 2457 | protein of unknown function DUF710 | BCAL2667 |
| BCAS0636 | 0.48 | 9E-03 | No homolog | No gene annotation | BCAS0636 |
| BCAL0692 | 0.48 | 2E-03 | 2906 | hypothetical protein | BCAL0692 |
| BCAM1591A | 0.48 | 2E-03 | 4451 | hypothetical protein | BCAM1591A |
| BCAM0160 | 0.48 | 1E-02 | 5854 | hypothetical protein | BCAM0160 |
| BCAM2002 | 0.48 | 1E-02 | 4802 | hypothetical protein | BCAM2002 |
| HI2424_G6238 | 0.48 | 3E-03 | 0185 | hypothetical protein | No homolog |
| AU1054_G6494 | 0.48 | 3E-09 | 5584 | 17 kDa surface antigen | No homolog |
| BCAM1660 | 0.48 | 5E-04 | No homolog | No gene annotation | BCAM1660 |
| BCAL0585 | 0.49 | 1E-02 | 3007 | hypothetical protein | BCAL0585 |
| BCAS0016 | 0.49 | 6E-03 | 6178 | protein of unknown function DUF1656 | BCAS0016 |
| HI2424_G6176 | 0.49 | 7E-10 | 6818 | hypothetical protein | No homolog |
| BCAL2025 | 0.49 | 8E-04 | 1952 | hypothetical protein | BCAL2025 |
| BCAL3059A | 0.49 | 1E-02 | No homolog | No gene annotation | BCAL3059A |
| AU1054_G5032 | 0.49 | 1E-02 | 6119 | LamG domain protein jellyroll fold domain protein | No homolog |
| BCAM0371 | 0.49 | 8E-11 | 3359 | protein of unknown function DUF1105 | BCAM0371 |
| BCAM2206 | 0.49 | 2E-11 | 4972 | hypothetical protein | BCAM2206 |
| AU1054_G3587 | 0.49 | 7E-09 | 4621 | hypothetical protein | No homolog |
| AU1054_G2890 | 0.49 | 2E-08 | 6384 | hypothetical protein | No homolog |
| BCAM0532 | 0.49 | 7E-11 | No homolog | No gene annotation | BCAM0532 |
| BCAL2633 | 0.50 | 9E-03 | No homolog | No gene annotation | BCAL2633 |
| BCAM2209 | 0.50 | 4E-02 | 4977 | hypothetical protein | BCAM2209 |
| BCAL0470 | 0.50 | 2E-04 | No homolog | No gene annotation | BCAL0470 |
| BCAM0429 | 0.50 | 4E-03 | 3396 | hypothetical protein | BCAM0429 |
| BCAM2673 | 0.50 | 3E-04 | 5477 | hypothetical protein | BCAM2673 |
| BCAM1196 | 0.50 | 9E-03 | No homolog | No gene annotation | BCAM1196 |
| BCAS0615 | 0.50 | 4E-04 | No homolog | No gene annotation | No homolog |
| HI2424_G6239 | > 0.01 | 4E-07 | 0184 | NmrA family protein | No homolog |
| HI2424_G6234 | > 0.01 | 6E-07 | 0194 | hypothetical protein | No homolog |
| AU1054_G2322 | > 0.01 | 6E-07 | 0227 | hypothetical protein | No homolog |
| AU1054_G1003 | > 0.01 | 2E-04 | 0454 | phage protein | No homolog |
| AU1054_G1002 | > 0.01 | 3E-05 | 0461 | hypothetical protein | No homolog |
| AU1054_G992 | > 0.01 | 3E-06 | 0462 | hypothetical protein | No homolog |
| AU1054_G977 | > 0.01 | 3E-04 | 0477 | hypothetical protein | No homolog |
| AU1054_G976 | > 0.01 | 8E-04 | 0478 | hypothetical protein | No homolog |
| AU1054_G6519 | > 0.01 | 8E-06 | 0773 | hypothetical protein | No homolog |
| AU1054_G197 | > 0.01 | 2E-05 | 1013 | hypothetical protein | No homolog |
| AU1054_G6501 | > 0.01 | 2E-06 | 1681 | hypothetical protein | No homolog |
| HI2424_G6721 | > 0.01 | 1E-06 | 3479 | hypothetical protein | No homolog |
| HI2424_G6712 | > 0.01 | 4E-06 | 3490 | chlorinating enzyme | No homolog |
| AU1054_G4821 | > 0.01 | 3E-04 | 3748 | hypothetical protein | No homolog |
| AU1054_G5214 | > 0.01 | 9E-05 | 4411 | hypothetical protein | No homolog |
| AU1054_G5384 | > 0.01 | 1E-04 | 4470 | putative lipoprotein | No homolog |
| AU1054_G6444 | > 0.01 | 1E-06 | 5022 | hypothetical protein | No homolog |
| AU1054_G5752 | > 0.01 | 6E-07 | 5344 | hypothetical protein | No homolog |
| AU1054_G1121 | > 0.01 | 7E-06 | 5436 | hypothetical protein | No homolog |
| AU1054_G6499 | > 0.01 | 4E-06 | 5564 | hypothetical protein | No homolog |
| HI2424_G4078 | > 0.01 | 2E-06 | 6886 | hypothetical protein | No homolog |
| HI2424_G4079 | > 0.01 | 5E-05 | 6887 | hypothetical protein | No homolog |
| HI2424_G4089 | > 0.01 | 9E-06 | 6903 | hypothetical protein | No homolog |
| HI2424_G4114 | > 0.01 | 3E-04 | 6905 | hypothetical protein | No homolog |
| AU1054_G2400 | > 0.01 | 8E-06 | Multiple hits | No gene annotation | No homolog |
| AU1054_G2651 | > 0.01 | 2E-05 | Multiple hits | No gene annotation | No homolog |
| AU1054_G3131 | > 0.01 | 2E-05 | Multiple hits | No gene annotation | No homolog |
| AU1054_G6341 | > 0.01 | 1E-06 | Multiple hits | No gene annotation | No homolog |
| AU1054_G893 | > 0.01 | 4E-05 | Multiple hits | No gene annotation | No homolog |
| AU1054_G998 | > 0.01 | 1E-04 | Multiple hits | No gene annotation | No homolog |
| AU1054_G5608 | > 0.01 | 1E-04 | No homolog | No gene annotation | No homolog |

* “No homolog” indicates there is not a gene containing at least an 85% identity to the sequence of the probe in that respective genome.
