# Supplementary material for: Identification of Potential Therapeutic Targets for Burkholderia cenocepacia by Comparative Transcriptomics
Source: PLoS One. 2010 Jan 15;5(1):e8724. doi: 10.1371/journal.pone.0008724 (PMC2806911; doi:10.1371/journal.pone.0008724)
Supplement: Table S5 — Conservation of genes/regions overexpressed in J2315 under CF conditions in both microarray comparisons. List of 458 genes uniquely induced in the clinical epidemic isolate J2315 under CF-like conditions compared to both itself under soil-like conditions and to the soil isolate HI2424 under CF-like conditions. (1.15 MB DOC) [file pone.0008724.s006.doc]

Table S5. Conservation (% nucleotide identity) of genes/regions overexpressed in J2315 under CF conditions in both microarray comparisons

|  |  | pSORTb† | ***B. cenocepacia*** | | | | | ***B. lata*** | ***B. ambifaria*** | | ***B. vietnamensis*** | ***B. dolosa*** | ***B. multi-vorans*** | ***B. xeno-vorans*** | ***P. aeru-ginosa*** |
| --- | --- | --- | --- | --- | --- | --- | --- | --- | --- | --- | --- | --- | --- | --- | --- |
| **GENE ID** | **Annotation** |  | **J**  **2315** | **HI**  **2424** | **AU**  **1054** | **PC-184** | **MCO-3** | **ATCC 17776** | **AMMD** | **MC 40-6** | **G4** | **AU**  **1058** | **ATCC17616** | **LB400** | **PA14** |
|  | ***Energy production and conversion (C)*** |  |  |  |  |  |  |  |  |  |  |  |  |  |  |
| BCAL0029 | ATP synthase protein I ATPI | CM | 100 | 98 | 98 | 98 | 98 | 95 | 91 | 92 | 90 | 89 | 91 | 73 |  |
| BCAL0034 | ATP synthase alpha chain | U | 100 | 99 | 99 | 99 | 99 | 97 | 97 | 97 | 97 | 96 | 96 | 91 | 78 |
| BCAL0037 | ATP synthase epsilon chain | C | 100 | 99 | 99 | 99 | 99 | 97 | 96 | 96 | 96 | 95 | 96 | 82 |  |
| BCAL0164 | Cytochrome c-551 precursor* | P | 100 | 95 | 95 | 95 | 95 | 90 | 89 | 90 | 86 | 88 | 84 | - |  |
| BCAL0408 | Phenylacetic acid degradation | C | 100 | 95 | 95 | 95 | 95 | 92 | 90 | 91 | 89 | 87 | 89 | 80 |  |
| BCAL0754 | Cytochrome c oxidase subunit III | CM | 100 | 98 | 98 | 98 | 98 | 97 | 97 | 97 | 95 | 93 | 94 | 85 | 65 |
| BCAL0850 | Glycolate permease* | CM | 100 | - | - | - | - | 93 | 94 | 94 | 93 |  |  | - | 73 |
| BCAL0851 | Iron-sulphur cluster containing | C | 100 | - | - | - | - | 94 | 94 | 94 | 91 | 91 | 91 | - | - |
| BCAL0853 | Conserved hypothetical protein | C | 100 | 57 | 57 | 57 | 57 | 91 | 90 | 90 | 88 | 57 | 59 | - |  |
| BCAL1517 | Dihydrolipoamide dehydrogenase | C | 100 | 97 | 97 | 97 | 97 | 97 | 97 | 97 | 96 | 95 | 96 | 88 | 64 |
| BCAL2331 | NADH dehydrogenase I chain N* | CM | 100 | 98 | 98 | 97 | 97 | 96 | 95 | 95 | 95 | 95 | 92 | 80 |  |
| BCAL2332 | NADH dehydrogenase I chain M | CM | 100 | 98 | 98 | 98 | 98 | 96 | 96 | 96 | 95 | 95 | 95 | 86 |  |
| BCAL2334 | NADH-ubiquinone oxidoreductase I chain K | CM | 100 | 98 | 98 | 97 | 98 | 95 | 96 | 97 | 95 | 97 | 96 | 91 |  |
| BCAL2335 | NADH dehydrogenase I chain J | CM | 100 | 98 | 98 | 98 | 98 | 97 | 96 | 95 | 94 | 94 | 94 | 84 |  |
| BCAL2485 | 4Fe-4S ferredoxins, iron-sulfur cluster binding | C | 100 | 59 | 59 | 59 | 58 | 59 | 59 | - | 99 | 91 | 91 | 73 | - |
| BCAL2486 | Iron-sulphur oxidoreductase | U | 100 | - | - | - | - | - | - | - | 99 | 58 | 83 | 75 | - |
| BCAL2487 | D-Lactate dehydrogenase | U | 100 | - | - | - | - | - | - | - | 98 |  |  | 76 |  |
| BCAL2908 | Fumarate hydratase class II | C | 100 | 97 | 97 | 97 | 97 | 95 | 94 | 94 | 94 | 94 | 94 | 85 | 67 |
| BCAL3271 | Thioredoxin | U | 100 | 93 | 93 | 91 | 91 | 91 | 85 | 86 | 90 | 88 | 88 | 79 | 66 |
| BCAL3312 | Cytochrome b-561 membrane protein* | CM | 100 | 97 | 97 | 96 | 98 | 91 | 92 | 92 | 88 | 90 | 89 | 79 |  |
| BCAM0961 | Aconitate hydratase | C | 100 | 97 | 97 | 97 | 97 | 96 | 96 | 96 | 95 | 94 | 93 | 88 |  |
| BCAM0967 | Succinate dehydrogenase cytochrome b556 | CM | 100 | 97 | 97 | 97 | 97 | 96 | 96 | 96 | 94 | 95 | 95 | 74 |  |
| BCAM0969 | Succinate dehydrogenase flavoprotein subunit | U | 100 | 96 | 96 | 96 | 96 | 96 | 96 | 96 | 94 | 95 | 94 | 87 | 67 |
| BCAM0972 | Citrate synthase | CM | 100 | 98 | 98 | 98 | 98 | 97 | 96 | 96 | 97 | 95 | 95 | 90 | 72 |
| BCAM1250 | Probable acetyl-coA hydrolase/transferase* | U | 100 | 97 | 97 | - | 97 | 95 | 95 | 95 | 94 | - | 88 | 77 | - |
| BCAM1954 | Sodium:Dicarboxylate symporter* | CM | 100 | 95 | 95 | 95 | 95 | 93 | 92 | 91 | 61 | 61 | 60 | 57 | - |
| BCAM2277 | FMN-dependent dehydrogenase | C | 100 | 94 | 94 | 94 | 95 | - | - | - | - | - | 74 | - | - |
| pBCA043 | Thiol:disulfide interchange protein DsbC* | P | 100 | - | - | - | - | - | - | - | 58 |  |  | - |  |
|  | ***Cell cycle control, cell division, chromosome partitioning (D)*** |  |  |  |  |  |  |  |  |  |  |  |  |  |  |
| BCAL0143 | Flagellar biosynthesis protein | U | 100 | 98 | 98 | 97 | 98 | 93 | 91 | 91 | 89 | 90 | 87 | 66 |  |
| BCAL2147 | tRNA(Ile)-Lysidine synthase | U | 100 | 92 | 92 | 92 | 93 | 88 | 86 | 86 | 83 | 89 | 88 | 70 | - |
| BCAL2178 | Septum formation initiator | U | 100 | 96 | 96 | 96 | 96 | 95 | 91 | 92 | 90 | 92 | 92 | 79 |  |
| BCAL2417 | DNA translocase* | CM | 100 | 87 | 87 | 83 | 87 | - | - | - | - | 92 | 89 | - | 58 |
| BCAL3458 | Cell division protein FtsA | C | 100 | 98 | 98 | 98 | 98 | 97 | 96 | 96 | 96 | 95 | 95 | 90 | 72 |
| BCAL3469 | Cell division protein FtsL* | U | 100 | 97 | 97 | 97 | 97 | 94 | 94 | 93 | 90 | 95 | 92 | 83 |  |
|  | ***Amino acid transport and metabolism (E)*** |  |  |  |  |  |  |  |  |  |  |  |  |  |  |
| BCAL0280 | 3-Dehydroquinate synthase | C | 100 | 97 | 97 | 97 | 97 | 94 | 93 | 94 | 91 | 91 | 92 | 82 | 69 |
| BCAL0291 | Sodium:amino acid symporter family protein | CM | 100 | 97 | 97 | 97 | 97 | 92 | 92 | 92 | - |  | 92 | - | 65 |
| BCAL0377 | Metallopeptidase, subfamily M24B | U | 100 | 96 | 96 | 96 | 96 | 94 | 91 | 91 | 91 | 93 | 91 | 77 | - |
| BCAL1839 | Asparagine synthase | U | 100 | 92 | 92 | 92 | 92 | 89 | 92 | - | - |  |  | - |  |
| BCAL1925 | Threonine synthase | C | 100 | 97 | 97 | 96 | 97 | 95 | 96 | 95 | 93 | 94 | 94 | 84 |  |
| BCAL1988 | D-Amino acid dehydrogenase small | C | 100 | 94 | 94 | 94 | 94 | 92 | 90 | 90 | 88 |  | 86 | 71 |  |
| BCAL2146 | Aspartokinase | C | 100 | 98 | 98 | 98 | 98 | 97 | 96 | 96 | 95 | - | - | 86 | - |
| BCAL2317 | Family M14 unassigned peptidase | C | 100 | 96 | 96 | 96 | 96 | 92 | 91 | 91 | 91 | 87 | 88 | 79 | 58 |
| BCAL2659 | Cobalamin biosynthesis aminotransferase | U | 100 | 93 | 93 | 92 | 93 | 90 | 86 | 86 | 85 | 86 | 85 | 73 | 60 |
| BCAL2942 | Cysteine synthase | U | 100 | 95 | 95 | 96 | 95 | 94 | 93 | 93 | 92 | 91 | 93 | 85 | 69 |
| BCAL2954 | P-Protein [bifunctional includes: chorismate mutase/prephenate dehydratase] | C | 100 | 96 | 95 | 96 | 96 | 94 | 93 | 94 | 93 | 92 | 94 | 85 | 62 |
| BCAL3056 | Aminotransferase | U | 100 | 95 | 95 | 95 | 95 | 92 | 92 | 91 | 91 | 83 | 81 | 78 | - |
| BCAL3197 | Serine hydroxymethyltransferase | C | 100 | 97 | 97 | 99 | 96 | 95 | 94 | 94 | 94 | 94 | 94 | 88 | 70 |
| BCAM0547 | LysE type translocator | CM | 100 | - | - | - | - | 87 | 87 | 86 | 82 | - | - | - | - |
| BCAM1243 | Aminotransferase | C | 100 | 93 | 93 | - | 93 | 91 | 90 | 90 | 90 | - | - | 77 | - |
| BCAM1416 | LysE-family transporter* | CM | 100 | 97 | 97 | 97 | 97 | 94 | 87 | 87 | 88 | 92 | 87 | - | 58 |
| BCAM1798 | Oxidoreductase | CM | 100 | 94 | 94 | 94 | 94 | 89 | 90 | 90 | 88 | 85 | 83 | 74 | - |
| BCAS0060 | Extracellular amino acid-binding protein* | P | 100 | 94 | 94 | 96 | 96 | 96 | 95 | 95 | 94 | 93 | 91 | 81 |  |
| BCAS0129 | Binding-protein-dependent transport | CM | 100 | 95 | 95 | 95 | 96 | 93 | - | - | - | 92 | 90 | 83 | 65 |
|  | ***Nucleotide transport and metabolism (F)*** |  |  |  |  |  |  |  |  |  |  |  |  |  |  |
| BCAL2181 | CTP synthase | C | 100 | 98 | 98 | 98 | 98 | 96 | 95 | 96 | 95 | - | 92 | 90 | - |
| BCAS0337 | Lipoprotein * | U | 100 | 92 | 92 | - | 91 | 83 | - | - | - |  |  | - | 73 |
|  | ***Carbohydrate transport and metabolism (G)*** |  |  |  |  |  |  |  |  |  |  |  |  |  |  |
| BCAL0743 | Glycerol-3-phosphate dehydrogenase | C | 100 | 96 | 96 | 96 | 97 | 95 | 93 | 92 | 91 | 92 | 90 | 79 |  |
| BCAL0782 | Chitobiase* | P | 100 | 96 | 96 | 95 | 96 | 92 | 91 | 91 | 88 |  |  | - |  |
| BCAL1035 | Trehalose-phosphatase | C | 100 | 96 | 96 | 96 | 96 | 94 | 91 | 90 | 91 | 90 | 90 | 76 |  |
| BCAL1181 | Glycerate kinase | C | 100 | 62 | 62 | 62 | 62 | 61 | 61 | 61 | 60 | 60 | 61 | 60 |  |
| BCAL1252 | Proline/Betaine transporter | CM | 100 | 97 | 97 | 96 | 96 | 94 | 93 | 94 | 93 | 92 | 93 | 83 |  |
| BCAL2470 | Major Facilitator Superfamily protein | CM | 100 | 97 | 97 | 96 | 96 | 93 | 93 | 93 | 90 | 89 | 90 | 76 | - |
| BCAL2860 | Beta-hexosaminidase 1 | C | 100 | 96 | 96 | 96 | 96 | 93 | 93 | 93 | 91 | 91 | 89 | 77 | 62 |
| BCAL2947 | Membrane protein | C | 100 | 95 | 95 | 95 | 95 | 93 | 90 | 90 | 90 | 90 | 91 | 78 |  |
| BCAM1262 | Dihydroxyacid dehydratase | C | 100 | 98 | 98 | - | 98 | 95 | 95 | 95 | 94 | 93 | 94 | 71 | 78 |
| BCAM1741 | Major Facilitator Superfamily protein* | CM | 100 | 96 | 96 | 97 | 96 | 92 | 92 | 91 | 92 | 93 | 91 | 83 | 63 |
| BCAM1760 | Multidrug resistance transporter | CM | 100 | 97 | 97 | 97 | 97 | 93 | 92 | 92 | 72 | 76 | 71 | 77 | 62 |
| BCAM2464 | Major Facilitator Superfamily protein | CM | 100 | 96 | 96 | 95 | 96 | 95 | 94 | 94 | 93 | - | - | 81 | - |
| BCAM2665 | Major Facilitator Superfamily protein | CM | 100 | 95 | 95 | 67 | 94 | 93 | 90 | 89 | 88 | 93 | 91 | - | - |
| BCAS0706 | Major Facilitator Superfamily protein | CM | 100 | 70 | 70 | 61 | 70 | 59 | 59 | 59 | 89 | 85 | - | 58 | - |
|  | ***Coenzyme transport and metabolism (H)*** |  |  |  |  |  |  |  |  |  |  |  |  |  |  |
| BCAL1705 | Cobyrinic acid A,C-diamide synthase | U | 100 | 92 | 92 | 95 | 95 | 87 | 90 | 90 | 86 | 87 | 85 | 73 | 65 |
| BCAL1711 | Cobalamin biosynthesis-related protein | U | 100 | 96 | 96 | 96 | 96 | 92 | 93 | 93 | 91 | 90 | 90 | 76 | 65 |
| BCAL1840 | Conserved hypothetical protein | C | 100 | 96 | 96 | 96 | 96 | 91 | 90 | - | - |  |  | - |  |
| BCAL2662 | Cobalamin [5'-phosphate] synthase | CM | 100 | 95 | 95 | 95 | 94 | 90 | 89 | 88 | 89 | 89 | 86 | 73 |  |
| BCAL2717 | Nicotinate-Nucleotide | C | 100 | 94 | 94 | 94 | 94 | 92 | 92 | 92 | 91 | 90 | 90 | 78 | 62 |
| BCAM2080 | Bifunctional NMN | U | 100 | 98 | 98 | 98 | 98 | 95 | 95 | 95 | 93 | 90 |  | - |  |
|  | ***Lipid transport and metabolism (I)*** |  |  |  |  |  |  |  |  |  |  |  |  |  |  |
| BCAL0882 | Phospholipase | U | 100 | 96 | 96 | 96 | 96 | 91 | 92 | 92 | 91 | - | - | 72 | - |
| BCAL1019 | CDP-diacylglycerol-glycerol-3-phosphate 3-phosphatidyltransferase 2 | CM | 100 | 96 | 96 | 96 | 96 | 94 | 95 | 95 | 94 | 92 | 93 | 85 | - |
| BCAL1473 | Succinyl-coA:3-ketoacid-coenzyme A transferase | CM | 100 | 96 | 96 | 96 | 96 | 95 | 95 | 95 | 93 | 92 | 92 | 86 | - |
| BCAL2834 | Acylhydrolase* | OM | 100 | 96 | 96 | 96 | 96 | 88 | 91 | 92 | 93 | 92 | 92 | - | - |
| BCAL2875 | Acyl carrier protein (ACP) 1 | C | 100 | 98 | 98 | 98 | 98 | 98 | 96 | 96 | 96 | 79 | 77 | 92 | 60 |
| BCAL2877 | Malonyl coA-acyl carrier protein transacylase 1 | U | 100 | 96 | 96 | 96 | 96 | 94 | 93 | 93 | 92 | 96 | 96 | 84 | 80 |
| BCAL2878 | 3-Oxoacyl-[acyl-carrier-protein] synthase III 1 | CM | 100 | 98 | 97 | 97 | 98 | 96 | 94 | 94 | 93 | - | 90 | 84 | 64 |
| BCAM1459 | Short-chain fatty acid transporter | CM | 100 | - | - | - | - | 93 | 94 | 94 | - | - | - | - | - |
| BCAS0504 | Phage transmembrane acetyltransferase | CM | 100 | - | - | - | - | - | - | - | - | - | 91 | - | - |
|  | ***Intergenic regions*** |  |  |  |  |  |  |  |  |  |  |  |  |  |  |
| IG1_1078958 | Interg_chr1_pos_587_1078958:1079086 | N.A. | 100 | 95 | 95 | 92 | 95 | 95 | 95 | 94 | 88 | 91 | 59 | - | - |
| IG1_1089442 | Interg_chr1_pos_599_1089442:1089630 | N.A. | 100 | - | - | - | - | - | - | - | - | 95 | 93 | - | - |
| IG1_1120751 | Interg_chr1_pos_623_1120751:1120836 | N.A. | 100 | 93 | 93 | 93 | 93 | 83 | - | - | - | - | - | - | - |
| IG1_1277099 | Interg_chr1_pos_697_1277099:1278690 | N.A. | 100 | 94 | 94 | 94 | 94 | 91 | - | - | - | - | 76 | 56 | - |
| IG1_137022 | Interg_chr1_pos_62_137022:139507 | N.A. | 100 | 94 | 94 | 91 | 94 | 90 | 82 | 89 | 86 | - | 97 | - | 68 |
| IG1_1381576 | Interg_chr1_pos_750_1381576:1382014 | N.A. | 100 | 100 | 100 | - | 100 | 96 | 98 | 98 | 95 | 87 | - | - | - |
| IG1_147730 | Interg_chr1_pos_68_147730:147909 | N.A. | 100 | 83 | 83 | 83 | 84 | 73 | - | - | - | - | - | - | - |
| IG1_1780440 | Interg_chr1_pos_964_1780440:1780658 | N.A. | 100 | 96 | 96 | 96 | 96 | 95 | 94 | 95 | 92 | - | 81 | - | - |
| IG1_1780908 | Interg_chr1_pos_965_1780908:1781428 | N.A. | 100 | - | - | - | - | - | - | - | - | - | - | - | - |
| IG1_1891301 | Interg_chr1_pos_1027_1891301:1891368 | N.A. | 100 | 95 | 95 | 94 | 94 | 96 | 96 | 96 | 94 | - | - | - | - |
| IG1_2102819 | Interg_chr1_pos_1104_2102819:2102948 | N.A. | 100 | 84 | 84 | 84 | 84 | 84 | - | - | - | - | 86 | - | - |
| IG1_2266807 | Interg_chr1_pos_1177_2266807:2267628 | N.A. | 100 | 98 | 98 | - | 98 | 98 | 100 | 100 | 96 | - | 86 | - | - |
| IG1_2384865 | Interg_chr1_pos_1213_2384865:2385669 | N.A. | 100 | 87 | 87 | 87 | 87 | - | - | - | - | 61 | 62 | - | - |
| IG1_247826 | Interg_chr1_pos_123_247826:247889 | N.A. | 100 | 97 | 97 | 95 | 97 | 91 | 89 | 91 | 86 | - | - | 72 | - |
| IG1_257623 | Interg_chr1_pos_137_257623:257931 | N.A. | 100 | - | - | - | - | - | - | - | - | - | 93 | - | - |
| IG1_2737984 | Interg_chr1_pos_1344_2737984:2741642 | N.A. | 100 | - | - | 84 | - | - | - | - | - | 89 | 89 | - | - |
| IG1_2758916 | Interg_chr1_pos_1352_2758916:2758984 | N.A. | 100 | 88 | 88 | 88 | 87 | - | - | - | - | - | - | - | - |
| IG1_288839 | Interg_chr1_pos_174_288839:288976 | N.A. | 100 | 100 | 99 | 100 | 100 | 97 | 99 | 100 | 99 | - | - | - | - |
| IG1_2921887 | Interg_chr1_pos_1436_2921887:2922156 | N.A. | 100 | 86 | 86 | 85 | 86 | 85 | - | - | - | 93 | 93 | - | - |
| IG1_3139352 | Interg_chr1_pos_1527_3139352:3139618 | N.A. | 100 | - | - | 93 | 91 | 89 | - | - | - | - | - | - | - |
| IG1_3180610 | Interg_chr1_pos_1539_3180610:3180736 | N.A. | 100 | - | - | - | - | - | - | - | 100 | 82 | 73 | - | - |
| IG1_3308709 | Interg_chr1_pos_1618_3308709:3308938 | N.A. | 100 | 97 | 97 | 97 | 97 | 94 | 92 | 92 | 92 | - | - | - | - |
| IG1_3368693 | Interg_chr1_pos_1648_3368693:3368966 | N.A. | 100 | - | - | - | - | - | - | - | - | - | - | - | - |
| IG1_3414831 | Interg_chr1_pos_1677_3414831:3418039 | N.A. | 100 | 97 | 97 | 97 | 97 | 95 | 93 | 93 | 93 | - | 95 | - | - |
| IG1_3537906 | Interg_chr1_pos_1722_3537906:3539109 | N.A. | 100 | - | - | - | - | - | - | - | - | - | - | - | - |
| IG1_3541037 | Interg_chr1_pos_1724_3541037:3542321 | N.A. | 100 | - | - | - | - | - | - | - | - | - | - | - | - |
| IG1_3554535 | Interg_chr1_pos_1729_3554535:3554995 | N.A. | 100 | - | - | - | - | - | - | - | - | - | - | - | - |
| IG1_3556424 | Interg_chr1_pos_1730_3556424:3556621 | N.A. | 100 | 96 | 96 | 96 | 96 | 89 | 87 | 86 | 85 | - | - | - | - |
| IG1_3634213 | Interg_chr1_pos_1767_3634213:3634690 | N.A. | 100 | 88 | 88 | - | 88 | 81 | 79 | - | - | - | - | - | - |
| IG1_3664357 | Interg_chr1_pos_1786_3664357:3666206 | N.A. | 100 | - | - | - | - | - | - | - | - | - | - | - | - |
| IG1_37663 | Interg_chr1_pos_28_37663:37730 | N.A. | 100 | 90 | 90 | 90 | 90 | - | - | - | - | 92 | 91 | - | - |
| IG1_3766524 | Interg_chr1_pos_1817_3766524:3769935 | N.A. | 100 | - | - | - | - | - | - | - | - | 91 | 91 | - | - |
| IG1_3822505 | Interg_chr1_pos_1830_3822505:3822812 | N.A. | 100 | - | - | - | 80 | - | - | - | - | 90 | 91 | - | - |
| IG1_446055 | Interg_chr1_pos_278_446055:446118 | N.A. | 100 | 87 | 87 | 87 | 89 | - | - | - | - | 71 | - | - | - |
| IG1_453676 | Interg_chr1_pos_286_453676:454606 | N.A. | 100 | 84 | 84 | 83 | 84 | 76 | 69 | 71 | - | - | 95 | - | - |
| IG1_631117 | Interg_chr1_pos_374_631117:631394 | N.A. | 100 | 84 | 84 | 84 | 82 | 84 | - | - | - | - | - | - | - |
| IG1_890632 | Interg_chr1_pos_496_890632:890706 | N.A. | 100 | - | - | - | - | - | - | - | - | - | - | - | - |
| IG2_1375301 | Interg_chr2_pos_713_1375301:1376820 | N.A. | 100 | 89 | 89 | 88 | 89 | - | - | - | - | - | - | - | - |
| IG2_1452240 | Interg_chr2_pos_749_1452240:1452464 | N.A. | 100 | 92 | 92 | 91 | 90 | 83 | 81 | 80 | 85 | - | 92 | - | - |
| IG2_1537005 | Interg_chr2_pos_790_1537005:1537080 | N.A. | 100 | 96 | 96 | 95 | 97 | 91 | - | - | - | - | - | - | - |
| IG2_1656016 | Interg_chr2_pos_843_1656016:1656240 | N.A. | 100 | 96 | 96 | - | 97 | 96 | - | - | 58 | - | - | 83 | - |
| IG2_175839 | Interg_chr2_pos_85_175839:175944 | N.A. | 100 | 92 | 92 | 92 | 92 | 85 | 81 | - | 77 | 79 | 79 | - | - |
| IG2_2029035 | Interg_chr2_pos_1007_2029035:2029415 | N.A. | 100 | - | - | - | - | - | - | - | - | - | - | - | - |
| IG2_2089610 | Interg_chr2_pos_1036_2089610:2090371 | N.A. | 100 | 90 | - | 90 | 91 | 84 | - | - | - | - | - | - | - |
| IG2_2263493 | Interg_chr2_pos_1132_2263493:2263684 | N.A. | 100 | 86 | - | 88 | 86 | - | - | - | - | - | - | - | - |
| IG2_2644462 | Interg_chr2_pos_1252_2644462:2644620 | N.A. | 100 | 87 | 87 | 77 | 81 | 81 | - | - | - | - | - | - | - |
| IG2_2737778 | Interg_chr2_pos_1301_2737778:2741423 | N.A. | 100 | - | - | - | - | - | - | - | - | 79 | - | - | - |
| IG2_2875859 | Interg_chr2_pos_1346_2875859:2876060 | N.A. | 100 | - | - | - | - | 77 | - | - | - | - | - | - | - |
| IG2_2914215 | Interg_chr2_pos_1359_2914215:2914288 | N.A. | 100 | 92 | 92 | 92 | 92 | - | - | - | - | 71 | - | - | - |
| IG2_529992 | Interg_chr2_pos_274_529992:530292 | N.A. | 100 | 93 | 93 | 96 | 93 | 88 | 84 | 83 | 81 | - | - | - | - |
| IG2_55282 | Interg_chr2_pos_21_55282:57880 | N.A. | 100 | - | - | - | 85 | - | 86 | - | 79 | - | - | - | - |
| IG2_568212 | Interg_chr2_pos_292_568212:568307 | N.A. | 100 | 95 | 95 | 95 | 95 | 89 | - | - | - | - | - | - | - |
| IG2_990284 | Interg_chr2_pos_509_990284:991829 | N.A. | 100 | - | - | - | - | - | - | - | - | - | - | - | - |
| IG3_11785 | Interg_chr3_pos_3_11785:12268 | N.A. | 100 | - | - | - | - | - | - | - | 75 | 82 | 81 | - | - |
| IG3_202414 | Interg_chr3_pos_123_202414:203195 | N.A. | 100 | - | - | - | - | - | - | - | - | - | - | - | - |
| IG3_316621 | Interg_chr3_pos_167_316621:316725 | N.A. | 100 | 90 | 90 | 92 | 91 | 87 | 78 | - | - | - | - | 75 | 66 |
| IG3_769736 | Interg_chr3_pos_351_769736:770233 | N.A. | 100 | - | - | - | - | - | - | - | - | - | - | - | - |
| IG3_781759 | Interg_chr3_pos_354_781759:782387 | N.A. | 100 | 76 | 76 | 76 | 76 | 66 | - | - | - | - | - | - | - |
| IG3_784806 | Interg_chr3_pos_355_784806:785366 | N.A. | 100 | - | - | - | - | - | - | - | - | - | - | - | - |
|  | ***Translation, ribosomal structure and biogenesis (J)*** |  |  |  |  |  |  |  |  |  |  |  |  |  |  |
| BCAL0225 | 50S ribosomal protein L7/L12 | U | 100 | 98 | 98 | 98 | 98 | 92 | 95 | 95 | 94 | - | - | 86 | - |
| BCAL0239 | 50S ribosomal protein L22 | U | 100 | 99 | 99 | - | 99 | 99 | 99 | 99 | 99 | 94 | 94 | 95 | 70 |
| BCAL0250 | 50S ribosomal protein L18 | C | 100 | 98 | 98 | 99 | 99 | 98 | 97 | 97 | 98 | 97 | 97 | 90 | 69 |
| BCAL0424 | Ribonuclease P protein component | C | 100 | 98 | 98 | 98 | 98 | 96 | 95 | 96 | 95 | - | - | 75 | - |
| BCAL0485 | Glutamyl-tRNA amidotransferase subunit B | U | 100 | 97 | 97 | 97 | 97 | 96 | 96 | 96 | 96 | 95 | 94 | 85 | 64 |
| BCAL0799 | Ribosomal L25p family protein | U | 100 | 96 | 96 | 96 | 96 | 96 | 95 | 95 | 95 | 94 | 95 | 82 | 69 |
| BCAL0990 | 50S ribosomal protein L32 2 | U | 100 | 100 | 100 | 100 | 100 | 99 | 98 | 100 | 99 | 94 | 94 | 95 | 60 |
| BCAL2076 | RNA methylase protein | C | 100 | 92 | 92 | 92 | 92 | 90 | 88 | 88 | 89 | 98 | 98 | 76 | - |
| BCAL2104 | DNA methylase | C | 100 | 96 | 96 | 96 | 96 | 94 | 91 | 92 | 91 | 92 | 91 | 77 | 64 |
| BCAL2926 | tRNA(Guanine-N(1)-)-Methyltransferase | C | 100 | 97 | 97 | 97 | 97 | 95 | 91 | 91 | 91 | 94 | 96 | 80 | 72 |
| BCAL3257 | tRNA delta(2)-isopentenylpyrophosphate | U | 100 | 92 | 92 | 93 | 93 | 96 | 90 | 90 | 87 | 92 | 92 | 75 | 64 |
| BCAL3343 | D-Tyrosyl-tRNA | U | 100 | 96 | 96 | 94 | 96 | 94 | 93 | 93 | 91 | 88 | 89 | 83 | 66 |
| BCALr0332 | tRNA Thr anticodon TGT, Cove score 81.3 | U | 100 | 100 | 100 | 100 | 100 | 100 | 100 | 100 | 100 | 91 | 91 | 91 | 66 |
| BCALr0409c | tRNA Ala anticodon TGC, Cove score 90.89 | U | 100 | 100 | 100 | - | 100 | 100 | 100 | 100 | 100 | 100 | 100 | 96 | 82 |
| BCALr0457 | tRNA Lys anticodon CTT, Cove score 94.68 | U | 100 | 100 | 100 | 100 | 100 | 100 | 100 | 100 | 100 | - | 100 | 100 | 85 |
| BCALr0970a | tRNA Asn anticodon GTT, Cove score 85.87 | U | 100 | 100 | 100 | 100 | 100 | 100 | 100 | 100 | 100 | 100 | 100 | 100 | 77 |
| BCALr1551a | tRNA Leu anticodon CAG, Cove score 72.78 | U | 100 | 100 | 100 | - | 100 | 100 | 100 | 100 | 100 | 100 | 100 | 98 | 86 |
| BCALr1614 | tRNA Met anticodon CAT, Cove score 86.01 | U | 100 | 100 | 100 | 98 | 100 | 100 | 100 | 100 | 100 | 98 | 98 | 100 | 95 |
| BCALr2125c | tRNA Asp anticodon GTC, Cove score 90.27 | U | 100 | 98 | 98 | 75 | 98 | 98 | 98 | 98 | 96 | 98 | 100 | 96 | 96 |
| BCALr2125e | tRNA Asp anticodon GTC, Cove score 95.34 | U | 100 | 100 | 100 | - | 100 | 100 | 100 | 100 | 100 | 95 | 95 | 98 | 95 |
| BCALr2125f | tRNA Glu anticodon TTC, Cove score 60.1 | U | 100 | 100 | 100 | - | 100 | 100 | 100 | 100 | 100 | 100 | 100 | 100 | 100 |
| BCALr2219 | tRNA Met anticodon CAT, Cove score 86.8 | U | 100 | 100 | 100 | 95 | 100 | 96 | 96 | 96 | 96 | - | 100 | 96 | 86 |
| BCALr2687 | tRNA Leu anticodon CAA, Cove score 75.09 | U | 100 | 100 | 100 | 100 | 100 | 100 | 100 | 100 | 100 | 94 | 96 | 100 | 92 |
| BCALr2852d | tRNA Gly anticodon GCC, Cove score 89.05 | U | 100 | 100 | 100 | 100 | 100 | 100 | 100 | 100 | 100 | 100 | 100 | 98 | 78 |
| BCALr3205 | tRNA Lys anticodon TTT, Cove score 90.9 | U | 100 | 100 | 100 | - | 100 | 100 | 100 | 100 | 73 | 100 | 100 | 73 | 90 |
| BCALr3443 | tRNA Pro anticodon CGG, Cove score 81.43 | U | 100 | 100 | 100 | 100 | 100 | 100 | 100 | 100 | 100 | - | 76 | 100 | - |
| BCAM1839 | Endoribonuclease | U | 100 | - | - | - | - | 90 | - | - | - | 90 | 90 | - | - |
| BCAS0245 | 30S ribosomal protein S21 3 | U | 100 | 91 | 91 | - | 92 | - | 85 | 85 | 78 | 100 | 100 | - | 77 |
| BCAS0468 | Acetyltransferase - GNAT family | U | 100 | 94 | 94 | - | 94 | 87 | 86 | 85 | 86 | - | - | 61 | - |
|  | ***Transcription (K)*** |  |  |  |  |  |  |  |  |  |  |  |  |  |  |
| BCAL0221 | Transcription antitermination protein NusG | C | 100 | 97 | 97 | 97 | 97 | 96 | 96 | 96 | 94 | 67 | - | 87 | - |
| BCAL0226 | DNA-Directed RNA polymerase beta chain | C | 100 | 98 | 98 | 98 | 98 | 97 | 97 | 97 | 97 | 93 | 95 | 90 | 67 |
| BCAL0260 | DNA-Directed RNA polymerase alpha chain | C | 100 | 99 | 99 | 98 | 98 | 97 | 96 | 96 | 96 | 96 | 96 | 88 | 71 |
| BCAL0625 | LysR family regulatory protein | C | 100 | 96 | 96 | 96 | 96 | 94 | 95 | 94 | 92 | 95 | 93 | 76 | 68 |
| BCAL1180 | LysR family regulatory protein | C | 100 | 55 | 55 | 55 | 55 | - | 54 | 54 | - | 92 | 89 | - | - |
| BCAL1182 | Transcriptional regulator | U | 100 | - | - | - | - | - | - | - | - | - | - | - | - |
| BCAL1595 | DNA-binding phage protein | U | 100 | - | - | - | - | - | - | - | - | 62 | 62 | - | 63 |
| BCAL2488 | LysR family regulatory protein | CM | 100 | - | - | - | - | 59 | 60 | 61 | 98 | - | - | 71 | - |
| BCAL2540 | LysR family regulatory protein | C | 100 | 59 | 59 | 60 | 60 | 55 | - | 60 | - | 60 | 60 | 60 | 57 |
| BCAL2577 | Dj-1/PfpI family protein | U | 100 | - | - | - | - | - | - | - | - |  |  | - |  |
| BCAL2579 | LysR family regulatory protein | C | 100 | - | - | - | - | - | - | - | - | 70 | 53 | 69 | - |
| BCAL2586 | AraC family regulatory protein | U | 100 | - | - | - | - | - | - | - | - | - | - | - | - |
| BCAL2872 | RNA polymerase sigma-E factor (sigma-24) 1 | C | 100 | 98 | 98 | 98 | 98 | 98 | 95 | 95 | 94 | - | - | 85 | - |
| BCAM0412a | LysR family regulatory protein | U | 100 | - | - | - | - | 93 | - | - | - | 94 | 92 | 73 | 66 |
| BCAM2137 | LysR family regulatory protein | C | 100 | 61 | - | - | 61 | 60 | 62 | 62 | - | - | - | 60 | - |
| BCAM2548 | TetR family regulatory protein | U | 100 | - | - | - | - | - | - | - | - | - | 60 | - | - |
| BCAS0258 | GntR family regulatory protein | C | 100 | 91 | 91 | - | 92 | 89 | - | 89 | - | - | - | - | - |
| BCAS0715 | LysR family regulatory protein | C | 100 | - | - | - | - | 55 | - | - | - | - | - | - | - |
| BCAS0717 | Hypothetical protein | C | 100 | - | - | - | - | - | - | - | - | - | - | - | - |
|  | ***Replication, recombination, and repair (L)*** |  |  |  |  |  |  |  |  |  |  |  |  |  |  |
| BCAL0178 | DNA methyltransferase | U | 100 | - | - | - | - | - | - | - | - | 88 | 89 | - | - |
| BCAL1585 | Histone-like DNA-binding phage protein | U | 100 | 62 | 62 | 62 | 62 | 62 | 62 | 62 | 63 | - | - | 61 | - |
| BCAL2077 | Ribonuclease HII | C | 100 | 93 | 93 | 94 | 94 | 92 | 88 | 88 | 88 | 89 | 88 | 77 | - |
| BCAL2096 | DNA ligase | C | 100 | 96 | 96 | 97 | 97 | 94 | 93 | 93 | 93 | 88 | 86 | 83 | 63 |
| BCAL2218 | Transposase | C | 100 | - | - | - | - | - | - | - | - | - | - | - | - |
| BCAL2479 | IstB-like ATP binding protein | U | 100 | - | - | - | - | - | - | - | 92 | - | - | - | - |
| BCAL2480b | Transposase | C | 99 | 62 | - | - | - | 72 | 90 | - | 99 | - | - | 62 | - |
| BCAL2758 | Exodeoxyribonuclease VII large subunit | U | 100 | 96 | 96 | - | 95 | 93 | 92 | 92 | 90 | - | - | 79 | - |
| BCAL2943 | Exported protein* | U | 100 | 92 | 92 | 92 | 92 | 83 | 81 | 83 | 80 | 92 | 91 | 74 | - |
| BCAL3252 | Transposase | U | 100 | - | - | - | - | - | - | 88 | - | 69 | 93 | 78 | - |
| BCAL3293 | Transposase | C | 100 | - | - | - | - | - | - | - | - | - | - | - | - |
| BCAM0522 | Integrase | C | 100 | - | - | - | - | - | - | - | 93 | 100 | 100 | - | 90 |
| BCAM1925 | Transposase | C | 99 | 62 | - | - | - | 72 | 90 | - | 99 | - | - | 62 | - |
| pBCA057 | Conjugative transfer protein | C | 100 | 57 | - | - | - | - | - | - | - | - | - | - | - |
|  | ***Cell wall/membrane/envelope biogenesis (M)*** |  |  |  |  |  |  |  |  |  |  |  |  |  |  |
| BCAL0110 | Aminotransferase | C | 100 | 77 | 77 | 77 | 90 | - | - | - | - | - | - | 78 | - |
| BCAL0508 | Lipid A biosynthesis acyltransferase | CM | 100 | 98 | 98 | 98 | 97 | 84 | 87 | 87 | 94 | 89 | 85 | 82 | - |
| BCAL0894 | Exported protein* | OM | 100 | 97 | 97 | 97 | 97 | 95 | 94 | 94 | 92 | 89 | 89 | 78 | - |
| BCAL1258 | Exported transglycosylase protein* | U | 100 | 96 | 96 | 96 | 96 | 94 | 93 | 93 | 91 | 91 | 89 | 82 |  |
| BCAL1395 | Cellulose synthase catalytic subunit | CM | 100 | 95 | 95 | - | 96 | 93 | 91 | 92 | 90 | 90 | 89 | 68 |  |
| BCAL2078 | Lipid-A-disaccharide synthase | U | 100 | 96 | 96 | 95 | 96 | 95 | 93 | 93 | 92 | 91 | 93 | 79 | 69 |
| BCAL2081 | UDP-3-O-[3-hydroxymyristoyl] glucosamine | C | 100 | 96 | 96 | 96 | 96 | 94 | 91 | 89 | 89 | 92 | 92 | 78 | 59 |
| BCAL2082 | Chaperone protein Skp precursor* | OM | 100 | 97 | 97 | 96 | 97 | 95 | 93 | 92 | 93 | 91 | 91 | 81 | 69 |
| BCAL2083 | Outer membrane protein assembly factor YaeT* | OM | 100 | 98 | 98 | 98 | 98 | 96 | 96 | 95 | 96 | 95 | 95 | 85 |  |
| BCAL2166 | Lipoprotein* | U | 100 | 97 | 97 | 97 | 96 | 93 | 92 | 92 | 92 | 91 | 91 | 77 |  |
| BCAL2403 | LPS core biosynthesis protein | C | 100 | 73 | 73 | 73 | 73 | 73 | 71 | 72 | 72 | 72 | 63 | - |  |
| BCAL2404 | Glycosyltransferase | U | 100 | - | - | - | - | - | - | - | - |  |  | 68 |  |
| BCAL2405 | Membrane protein | CM | 100 | - | - | - | - | - | - | - | - |  |  | 67 |  |
| BCAL2406 | Glycosyltransferase | U | 100 | - | - | - | - | - | - | - | - |  |  | 72 |  |
| BCAL2407 | Glycosyltransferase | U | 100 | - | - | - | - | - | - | - | - |  |  | 71 |  |
| BCAL2482 | Outer membrane protein* | OM | 100 | - | - | - | 59 | - | - | - | 98 |  |  | 65 |  |
| BCAL2867 | Gtp-binding protein LepA 1 | C | 100 | 98 | 98 | 97 | 98 | 94 | 96 | 96 | 96 | 85 | 84 | 87 | - |
| BCAL2944 | ADP-L-clycero-D-manno-heptose-6-epimerase | C | 100 | 97 | 97 | 97 | 97 | 94 | 93 | 93 | 93 | 93 | 92 | 86 | - |
| BCAL2946 | UDP-glucose dehydrogenase | U | 100 | 96 | 96 | 96 | 96 | 94 | 91 | 91 | 91 | 92 | 92 | 80 | 69 |
| BCAL3110 | 3-Deoxy-D-manno-octulosonic acid | CM | 100 | 95 | 95 | 94 | 94 | 92 | 90 | 89 | 90 | 91 | 90 | 78 | 64 |
| BCAL3124 | Glycosyltransferase | C | 100 | - | - | - | - | - | - | - | - |  |  | - |  |
| BCAL3128 | Glycosyltransferase | C | 100 | - | - | - | - | - | - | - | - |  |  | - |  |
| BCAL3129 | Nucleotide sugar aminotransferase | U | 100 | - | - | 56 | - | - | - | - | - | 94 | 94 | 59 | 69 |
| BCAL3130 | ABC transporter ATP-binding protein | U | 100 | - | - | - | - | - | - | - | - | 90 | 90 | - | 64 |
| BCAL3132 | DTDP-4-keto-L-rhamnose reductase | U | 100 | 59 | 59 | 59 | 59 | - | - | - | - | 90 | 90 | - | - |
| BCAL3133 | DTDP-4-keto-6-deoxy-D-glucose 3,5-epimerase | U | 100 | 91 | 91 | 90 | 90 | 78 | 79 | 82 | 78 | - | - | 69 | - |
| BCAL3134 | Glucose-1-phosphate thymidylyltransferase | U | 100 | 94 | 94 | 94 | 94 | 83 | 83 | 85 | 84 | 85 | 86 | 83 | 73 |
| BCAL3219 | UDP-3-O-[3-hydroxymyristoyl] N-acetylglucosamine | C | 100 | 90 | 90 | 91 | 90 | 96 | - | - | - | 80 | 83 | - | 68 |
| BCAL3239 | Glucosyltransferase | C | 100 | - | - | - | - | - | - | - | - | - | - | - | - |
| BCAL3240 | Capsular polysaccharide transporter | U | 100 | 65 | 65 | 76 | 62 | 62 | - | - | - |  |  | - |  |
| BCAL3241 | Capsular polysaccharide export protein | CM | 100 | - | - | 72 | - | - | - | - | - |  |  | - |  |
| BCAL3242 | Capsule polysaccharide export protein* | CM | 100 | 57 | 57 | 71 | - | - | - | - | - |  |  | - |  |
| BCAL3243 | Capsular polysaccharide* | OM | 100 | 68 | 68 | 69 | 67 | 70 | - | - | - |  |  | - |  |
| BCAL3244 | Glycosyltransferase | C | 100 | 66 | 66 | 70 | 64 | 75 | - | - | - |  |  | - |  |
| BCAL3245 | Capsule polysaccharide export protein | C | 100 | - | - | - | - | 65 | - | - | - |  |  | - |  |
| BCAL3246 | GDP-Mannose pyrophosphorylase | U | 100 | 70 | 68 | 69 | 83 | 91 | 61 | 61 | 62 | 62 |  | - | 61 |
| BCAL3247 | Mechanosensitive ion channel protein | CM | 100 | 92 | 92 | 92 | 91 | 95 | 92 | 92 | - | 87 | 88 | 75 | - |
| BCAL3460 | D-Alanine-D-alanine ligase B | C | 100 | 97 | 97 | 97 | 97 | 93 | 95 | 95 | 93 | 94 | 95 | 84 | 62 |
| BCAM1472 | Glycosyltransferase | C | 100 | - | - | - | - | - | - | - | - |  |  | - |  |
| BCAM1543 | Glycosyl transferase* | C | 100 | - | - | - | - | - | - | - | - | 62 |  | - |  |
| BCAM2142 | Transport system outer membrane protein* | OM | 100 | - | - | - | - | - | - | - | - | - | 84 | - | 57 |
| BCAM2253 | Rhs-family protein | CM | 100 | 89 | 89 | 89 | 83 | - | - | 81 | - |  |  | - |  |
|  | ***Cell motility and secretion (N)*** |  |  |  |  |  |  |  |  |  |  |  |  |  |  |
| BCAL0113 | B-type flagellar hook-associated protein 2 | E | 100 | 96 | 96 | 96 | 95 | 77 | - | - | 67 |  | 81 | 73 |  |
| BCAL0114 | Flagellin (type II) | E | 100 | 98 | 98 | 92 | 91 | - | - | - | - |  |  | 80 |  |
| BCAL0126 | Chemotaxis protein MotA | CM | 100 | 97 | 97 | 97 | 97 | 97 | 95 | 95 | 93 | 89 | 88 | 87 | 63 |
| BCAL0127 | Chemotaxis protein MotB | CM | 100 | 97 | 97 | 97 | 98 | 93 | 93 | 93 | 90 | 96 | 94 | 79 | 60 |
| BCAL0140 | Flagellar biosynthetic protein FlhB | CM | 100 | 97 | 97 | 97 | 97 | 94 | 93 | 92 | 91 | 92 | 91 | 78 | 60 |
| BCAL0521 | Flagellar FliJ protein | U | 100 | 97 | 97 | 97 | 97 | 92 | 91 | 91 | 90 | 92 | 90 | 71 |  |
| BCAL0522 | Flagellum-specific ATP synthase FliI* | C | 100 | 98 | 98 | 98 | 98 | 94 | 94 | 94 | 91 | 92 | 92 | 83 |  |
| BCAL0523 | Flagellar assembly protein FliH | C | 100 | 97 | 97 | 96 | 96 | 95 | 93 | 92 | 91 | 93 | 90 | 72 |  |
| BCAL0524 | Flagellar motor switch protein FliG | C | 100 | 98 | 98 | 96 | 97 | 96 | 95 | 95 | 94 | 95 | 95 | 88 | 63 |
| BCAL0525 | Flagellar M-ring protein FliF | U | 100 | 98 | 98 | 98 | 98 | 95 | 94 | 94 | 94 | 94 | 91 | 75 |  |
| BCAL0526 | Flagellar hook-basal body complex protein FliE* | U | 100 | 98 | 98 | 98 | 98 | 96 | 95 | 94 | 89 |  | 95 | 76 |  |
| BCAL0562 | Negative regulator of flagellin synthesis, FlgA | U | 100 | 97 | 97 | 96 | 96 | 91 | 89 | 89 | 86 | 85 | 81 | - |  |
| BCAL0571 | Flagellar P-ring protein precursor (basal body)* | P | 100 | 95 | 95 | 95 | 95 | 95 | 93 | 94 | 92 | 93 | 93 | 84 | 66 |
| BCAL3505 | Flagellar motor switch protein FliN* | CM | 100 | 96 | 96 | 96 | 96 | 94 | 90 | 92 | 89 | 90 | 89 | 83 |  |
| BCAL3506 | Flagellar motor switch protein FliM | C | 100 | 98 | 98 | 98 | 98 | 97 | 96 | 96 | 95 | 94 | 93 | 86 | 63 |
| BCAL3507 | Flagellar FliL protein* | CM | 100 | 97 | 97 | 96 | 97 | 96 | 94 | 94 | 91 | 91 | 87 | 71 |  |
| BCAS0104 | A-Type flagellar hook-associated protein 2* | E | 100 | 93 | 93 | 92 | 92 | 83 | - | - | 63 |  | 73 | 67 |  |
|  | ***Posttranslational modification, protein turnover, chaperones (O)*** |  |  |  |  |  |  |  |  |  |  |  |  |  |  |
| BCAL0111 | TPR repeat protein | CM | 100 | 90 | 90 | 79 | 89 | - | - | - | - | - | - | 61 | - |
| BCAL0347 | Protease associated ATPase ClpB | C | 100 | 96 | 96 | 96 | 96 | 95 | 94 | 94 | 93 | - | - | - | - |
| BCAL2321 | Glutathione S-transferase | C | 100 | 94 | 94 | 94 | 94 | 92 | 87 | 87 | 84 | 94 | 94 | 69 | - |
| BCAL2323 | Glutathione S-transferase | U | 100 | 95 | 95 | 95 | 95 | 94 | 92 | 92 | 91 | 86 | 85 | - | - |
| BCAM1744 | Serine peptidase, family S9* | E | 100 | 94 | 94 | 93 | 94 | 90 | 85 | 85 | 82 | 90 | 91 | - | - |
|  | ***Inorganic ion transport and metabolism (P)*** |  |  |  |  |  |  |  |  |  |  |  |  |  |  |
| BCAL1270 | Phosphate transport system, substrate-binding* | P | 100 | 98 | 98 | 98 | 98 | 95 | 95 | 95 | 95 | 68 | 77 | 87 | 76 |
| BCAL1271 | Phosphate transport system permease protein | CM | 100 | 97 | 97 | 96 | 97 | 93 | 94 | 94 | 94 | 94 | 94 | 84 | - |
| BCAL2271 | Toxic anion resistance protein | C | 100 | - | - | - | - | 94 | - | - | 89 |  |  | - |  |
| BCAL2353 | Sulfate transporter | CM | 100 | 92 | 92 | 92 | 92 | 92 | 91 | 91 | 89 | 92 | 90 | 76 | - |
| BCAL2896 | Membrane protein* | CM | 100 | 97 | 97 | 97 | 97 | 94 | 93 | 93 | 91 | 93 | 91 | 79 |  |
| BCAL2938 | ABC transporter ATP-binding protein | C | 100 | 96 | 96 | 96 | 96 | 94 | 92 | 93 | 93 | 91 | 85 | 86 | - |
| BCAM1187 | Tonb-dependent siderophore receptor* | OM | 100 | - | - | 60 | - | 92 | 60 | 60 | 93 | 90 | 88 | 74 | 65 |
| BCAM1359 | Efflux pump/antiporter | CM | 100 | 96 | 96 | 96 | 96 | 94 | 94 | 93 | 91 | 92 | 92 | - |  |
|  | ***Secondary metabolites biosynthesis, transport, and catabolism (Q)*** |  |  |  |  |  |  |  |  |  |  |  |  |  |  |
| BCAL1710 | Cobalamin biosynthesis-related protein | C | 100 | 97 | 97 | 96 | 96 | 95 | 94 | 93 | 92 | 90 | 88 | 79 | 67 |
| BCAL2883 | Tetrapyrrole methylase | U | 100 | 96 | 96 | 97 | 97 | 94 | 93 | 93 | 91 | 92 | 91 | 77 |  |
| BCAL3183 | Hydrolase | U | 100 | 95 | 95 | 95 | 94 | 92 | 92 | 91 | 91 | - | - | 81 | - |
| BCAL3229 | Conserved hypothetical protein | C | 100 | - | - | - | - | - | - | - | - |  |  | - |  |
|  | ***General function prediction only (R)*** |  |  |  |  |  |  |  |  |  |  |  |  |  |  |
| BCAL0109 | TPR repeat protein | U | 100 | 95 | 95 | 93 | 94 | - | - | - | - |  |  | 68 |  |
| BCAL0771 | Non-Heme chloroperoxidase | U | 100 | 77 | 77 | 69 | 69 | 77 | 69 | 69 | 70 | - | - | 65 | - |
| BCAL0822 | NUDIX hydrolase | C | 100 | 97 | 97 | 97 | 97 | 93 | 92 | 92 | 89 | 86 | 93 | 73 | - |
| BCAL0989 | Conserved hypothetical protein | C | 100 | 97 | 97 | 97 | 97 | 96 | 93 | 93 | 91 | 92 | 93 | 78 |  |
| BCAL1518 | AFG1-like ATPase | C | 100 | 97 | 97 | 96 | 96 | 93 | 92 | 92 | 92 | 87 | 87 | 83 | - |
| BCAL1703 | Metallopeptidase, subfamily M20D | C | 100 | 96 | 96 | 96 | 96 | 91 | 90 | 91 | 89 | - | - | 78 | - |
| BCAL1846 | ABC transporter ATP-binding protein | CM | 100 | 93 | 93 | 93 | 93 | 90 | 89 | 90 | 87 | 72 | 90 | 72 | 71 |
| BCAL1886 | Radical SAM superfamily protein | C | 100 | 98 | 98 | 97 | 98 | 95 | 96 | 95 | 93 | 86 | 86 | 82 | - |
| BCAL2058 | Acetyltransferase (GNAT) family protein | C | 100 | 90 | 90 | 90 | 90 | 86 | 59 | - | - | 92 | 92 | - | 66 |
| BCAL2165 | Metallo-beta-lactamase superfamily protein | U | 100 | 95 | 95 | 95 | 96 | 92 | 92 | 92 | 89 | 95 | 94 | 76 | 67 |
| BCAL2466 | Ecotin precursor | P | 100 | 95 | 95 | 96 | 96 | 95 | 91 | 91 | 86 | 92 | 91 | - | 67 |
| BCAL2578 | Hydrolase | C | 100 | - | - | - | - | - | - | - | - | - | - | 67 | - |
| BCAL3234 | Glycosyltransferase | U | 100 | - | - | - | - | - | - | - | - |  |  | - |  |
| BCAL3248 | Transposase (pseudogene) | U | 77 | - | - | 69 | - | - | 89 | 70 | - | - | 88 | 62 | - |
| BCAM0150 | Lipoprotein * | U | 100 | - | - | 59 | - | 69 | - | 69 | 63 |  |  | - |  |
| BCAM0152 | Lipoprotein | U | 100 | - | - | 61 | - | 67 | - | 70 | 64 |  |  | - |  |
| BCAM0938 | Acetyltransferase (GNAT) family protein | U | 100 | - | - | - | - | 81 | - | - | - | 60 | 80 | - | - |
| BCAM1874 | GTP cyclohydrolase II (pseudogene) | C | 100 | 97 | 97 | 97 | 96 | 91 | 92 | 91 | 88 | 88 | 91 | 83 | 62 |
| BCAM2492 | Conserved hypothetical protein | C | 100 | 92 | 92 | 93 | 93 | 91 | 87 | 89 | 85 | 89 | 89 | 73 | - |
| BCAS0109 | Succinylglutamate desuccinylase/aspartoacylase | U | 100 | - | - | - | - | 92 | - | - | - |  |  | 68 |  |
| BCAS0152 | Hydrolase | U | 100 | 94 | 94 | 93 | 95 | 91 | 90 | 90 | 82 | - | - | 73 | 77 |
| BCAS0507 | Phage baseplate assembly protein gpJ | U | 100 | - | - | - | - | - | - | - | - | - | - | - | - |
| BCAS0517 | Phage tail tube protein | C | 100 | - | - | - | - | - | - | - | - | - | - | - | - |
| BCAS0722 | Patatin-like phospholipase | CM | 100 | - | - | - | - | - | - | - | - |  |  | - |  |
| pBCA051 | LamB/YcsF family protein | U | 100 | 60 | 60 | 61 | 61 | 59 | 62 | 60 | 61 | 60 | 59 | 60 |  |
|  | ***Function unknown (S)*** |  |  |  |  |  |  |  |  |  |  |  |  |  |  |
| BCAL0179 | Hypothetical protein | C | 100 | - | - | - | - | - | - | - | - |  |  | - |  |
| BCAL0360 | Conserved hypothetical protein | U | 100 | 91 | 91 | 91 | 90 | - | 81 | - | - |  |  | - |  |
| BCAL1172 | Conserved hypothetical protein | U | 100 | - | - | - | - | - | - | - | - |  |  | - |  |
| BCAL1455 | Fusaric acid resistance transporter* | CM | 100 | 96 | 96 | 96 | 96 | 92 | 91 | 91 | 90 | - | 56 | 72 | - |
| BCAL1520 | Lipoprotein* | U | 100 | 96 | 96 | 95 | 97 | 94 | 91 | 90 | 91 | 89 | 90 | 78 |  |
| BCAL1681 | Exported protein* | U | 100 | - | - | - | - | 87 | - | - | - | 92 | 91 | - | 62 |
| BCAL1714 | Conserved hypothetical protein | U | 100 | 97 | 97 | 98 | 97 | 95 | 92 | 92 | 88 | 88 | 86 | - | 56 |
| BCAL1881 | Lipoprotein* | OM | 100 | 97 | 97 | 97 | 96 | 95 | 93 | 94 | 92 | 93 | 92 | 79 |  |
| BCAL2269 | Membrane protein | CM | 100 | - | - | - | - | 91 | - | - | 92 |  |  | - |  |
| BCAL2273 | Conserved hypothetical protein | U | 100 | - | - | 93 | - | 90 | - | - | - | 63 |  | - |  |
| BCAL2275 | Conserved hypothetical protein | U | 100 | - | - | 93 | - | 90 | - | - | - | 61 |  | - |  |
| BCAL2493 | Membrane protein | CM | 100 | 55 | 55 | - | 54 | 75 | - | 55 | 85 |  | 56 | - |  |
| BCAL2495 | Membrane protein | CM | 100 | 62 | 62 | - | 63 | 62 | - | 63 | 89 |  | 62 | 66 |  |
| BCAL3279 | Membrane protein | E | 100 | 96 | 96 | 96 | 96 | 94 | 92 | 92 | 90 | 89 | 88 | 70 |  |
| BCAL3311 | Exported protein* | U | 100 | 99 | 99 | 99 | 99 | 94 | 96 | 95 | 93 | - | - | 74 | - |
| BCAM1027 | Hypothetical phage protein | U | 100 | - | - | - | - | - | - | - | - | - | - | - | - |
| BCAM2351 | Transmembrane component of ABC | CM | 100 | 98 | 98 | 98 | 98 | 96 | 94 | 94 | 93 | - | - | 81 | 54 |
| BCAS0521 | Hypothetical phage protein | U | 100 | - | - | - | - | - | - | - | - | - | - | - | - |
| BCAS0694 | Carboxymuconolactone decarboxylase | U | 100 | 58 | 58 | 69 | 59 | 57 | - | - | 89 | 68 |  | - |  |
| BCAS0723 | Conserved hypothetical protein | C | 100 | - | - | - | - | 63 | - | - | - | - | - | - | - |
|  | ***Signal transduction mechanisms (T)*** |  |  |  |  |  |  |  |  |  |  |  |  |  |  |
| BCAL0128 | Chemotaxis Two-component response regulator | C | 100 | 97 | 97 | 97 | 97 | 92 | 92 | 92 | 92 | 92 | 90 | 73 | 60 |
| BCAL0129 | Chemotaxis Two-component sensor kinase CheA | C | 100 | 94 | 94 | - | 94 | 90 | 90 | 89 | 87 | 91 | 89 | 75 | 66 |
| BCAL0131 | Methyl-accepting chemotaxis protein | CM | 100 | 95 | 95 | - | 95 | 90 | 88 | 88 | 87 | 93 | 86 | 76 |  |
| BCAL0132 | Chemotaxis protein methyltransferase, CheR | C | 100 | 96 | 96 | - | 94 | 92 | 91 | 91 | 90 | - | 86 | 82 | - |
| BCAL0135 | Chemotaxis protein CheY | C | 100 | 97 | 97 | 97 | 97 | 97 | 96 | 97 | 96 | 92 | 89 | 86 | 63 |
| BCAL2846 | Conserved hypothetical protein | U | 100 | 96 | 96 | 96 | 96 | 93 | 91 | 92 | 89 | 89 | 90 | 79 | 65 |
| BCAM0110 | Two-component regulatory system, sensor kinase | CM | 100 | 93 | 93 | 93 | 93 | 85 | - | - | - | 95 | 95 | - | 68 |
| BCAM0221 | Two-component regulatory system, response regulator | C | 100 | - | - | 82 | 82 | 60 | 65 | - | 62 | - | 83 | 57 | - |
| BCAM0227 | Hybrid two component system kinase-responseregulator | CM | 100 | - | - | 80 | 80 | - | - | - | - | 63 | - | - | - |
| BCAM1417 | Two-component regulatory system, sensor kinase | CM | 100 | 95 | 95 | 90 | 95 | 89 | 90 | 90 | 90 | - | - | 66 | - |
| BCAM1418 | Two-component regulatory system, response regulator | C | 100 | 96 | 96 | 96 | 97 | 95 | 95 | 95 | 94 | 89 | 89 | 80 | - |
| BCAM1503 | Methyl-Accepting chemotaxis protein* | CM | 100 | 96 | 96 | 96 | 97 | 92 | 91 | 91 | 89 | 95 | 93 | 77 | 73 |
| BCAM1804 | Methyl-accepting chemotaxis protein* | CM | 100 | 96 | 96 | 95 | 96 | 92 | 91 | 91 | 89 | 91 | 87 | 64 |  |
| BCAM2836 | Diguanylate cyclase | C | 100 | 97 | 97 | 97 | 97 | 95 | 92 | 93 | 90 | 90 | 89 | 79 | - |
| BCAS0632 | Hybrid two component system kinase-response regulator | CM | 100 | - | - | 94 | - | - | - | - | - | 93 | 89 | - | - |
| BCAS0707 | Two-component regulatory system, response regulator | C | 100 | 74 | 74 | 59 | 74 | 59 | 64 | 72 | 94 | 64 | 84 | 62 | - |
| BCAS0708 | Two-component regulatory system, sensor kinase | CM | 100 | 68 | 68 | - | 68 | - | - | - | 90 | 74 | 60 | - | 55 |
| BCAS0709 | Two-component regulatory system, response regulator | C | 100 | 73 | 73 | - | 73 | - | - | 64 | 91 | - | - | - | - |
| pBCA055 | Membrane protein | CM | 100 | - | - | - | - | - | - | - | - |  |  | - |  |
|  | ***Intracellular trafficking, secretion, and vesicular transport (U)*** |  |  |  |  |  |  |  |  |  |  |  |  |  |  |
| BCAL1004 | Signal peptidase I 2 (leader peptidase Lep 2) | CM | 100 | 98 | 98 | 96 | 98 | 97 | 95 | 95 | 94 | - | - | 84 | - |
| BCAL2345 | General secretory pathway, protein-export | CM | 100 | 96 | 96 | 95 | 92 | 93 | 93 | 93 | 87 | 94 | 94 | 71 | - |
| BCAL2475 | Membrane protein* | U | 100 | - | - | - | - | - | - | - | - |  |  | - |  |
| BCAL3435 | MarC family integral membrane protein | CM | 100 | 96 | 96 | 96 | 96 | 94 | 95 | 95 | 94 | 93 | 93 | 79 |  |
| pBCA041 | TraC conjugative transfer protein | C | 100 | 57 | - | - | - | - | - | - | - | - | - | - | - |
| pBCA059 | TraD conjugative transfer protein | CM | 100 | - | - | - | - | - | - | - | - | - | - | - | - |
|  | ***Defense mechanisms (V)*** |  |  |  |  |  |  |  |  |  |  |  |  |  |  |
| BCAL0479 | Penicillin-binding protein | CM | 100 | 97 | 97 | 97 | 96 | 95 | 95 | 95 | 94 | 94 | 94 | 82 |  |
| BCAL1177 | Fusaric acid resistance transporter | CM | 100 | 55 | 55 | 55 | 55 | 56 | 56 | 57 | 55 | - | - | - | - |
| BCAL1674 | Multidrug efflux system AmrA protein* | P | 100 | 97 | 97 | 96 | 96 | 93 | 90 | 89 | 86 | 88 | 84 | - | 69 |
| BCAL1675 | Multidrug efflux system transporter protein | CM | 100 | 97 | 97 | 97 | 97 | 94 | 95 | 95 | 92 | 93 | 91 | 63 | 74 |
| BCAL1676 | Multidrug efflux system outer membrane protein* | OM | 100 | 94 | 94 | 93 | 93 | 90 | 88 | 89 | 84 | 84 | 84 | 58 | 59 |
| BCAL2408 | Lipid A export ATP-binding/permease protein | CM | 100 | 80 | 80 | 80 | 80 | 80 | 80 | 79 | 79 | 91 | 91 | 78 | - |
| BCAM1419 | Efflux system outer membrane protein* | OM | 100 | 94 | 94 | 94 | 95 | 91 | 92 | 90 | - | 87 | 88 | 69 |  |
| BCAM1420 | Efflux system transport protein | CM | 100 | 96 | 96 | 96 | 96 | 92 | 92 | 92 | 59 | 89 | 89 | 69 |  |
| BCAM1421 | RND family efflux system transporter protein | CM | 100 | 96 | 96 | 96 | 96 | 95 | 93 | 93 | 69 | 93 | 90 | 75 |  |
| BCAM2140 | Transporter system transport protein* | CM | 100 | - | - | - | - | - | 55 | - | - | 89 | 87 | - | - |
| BCAM2141 | ABC transporter ATP-binding membrane protein | CM | 100 | - | - | - | - | - | 56 | 56 | - | - | 87 | - | 54 |
| BCAS0081 | ABC transporter ATP-binding membrane protein | CM | 100 | 97 | 97 | 96 | 96 | 93 | - | - | 91 | 90 | 88 | - | - |
| BCAS0716 | Restriction endonuclease | C | 100 | - | - | - | - | - | - | - | - | - | - | - | - |
| BCAL1680 | Type-1 fimbrial protein* | U | 100 | 75 | 75 | 75 | 76 | 91 | 75 | 75 | 63 |  | 72 | - |  |
|  | ***Extracellular structures (W)*** |  |  |  |  |  |  |  |  |  |  |  |  |  |  |
| BCAM0225 | Haemagglutinin-related protein* | U | 100 | - | - | - | - | - | - | - | - | 93 | 94 | - | - |
| BCAM2143 | Cable pilus associated adhesin protein | E | 100 | - | - | - | - | - | - | - | - |  | 77 | - |  |
| BCAM2759 | Minor pilin and initiator* | U | 100 | - | - | - | - | - | - | 73 | - |  |  | - |  |
| BCAM2762 | Giant cable pilus chaperone protein* | U | 100 | - | - | - | - | - | - | 84 | - |  |  | - |  |
|  | ***No assigned COG*** |  |  |  |  |  |  |  |  |  |  |  |  |  |  |
| BCAL0112 | Conserved hypothetical protein | U | 100 | 96 | 96 | 95 | 95 | - | - | - | - |  | 74 | - |  |
| BCAL0137 | Conserved hypothetical protein* | U | 100 | 96 | 96 | 96 | 97 | 93 | 90 | 91 | 87 | 89 | 84 | - |  |
| BCAL0138 | Gly/Ala/Ser-Rich lipoprotein (pseudogene)* | U | 99 | 93 | 93 | 93 | 95 | 88 | 82 | 78 | 81 | 81 | 80 | 59 |  |
| BCAL0169 | Conserved hypothetical protein | C | 100 | - | - | - | - | - | - | - | - |  |  | - |  |
| BCAL0352 | Metallopeptidase, subfamily M15C* | U | 100 | 94 | 94 | 95 | 95 | 96 | 93 | 93 | 91 | - | - | - | - |
| BCAL0419 | Hypothetical protein | C | 100 | - | - | - | - | - | - | - | - |  |  | - |  |
| BCAL1002 | Serine peptidase, family S33 | C | 100 | 96 | 96 | 97 | 97 | 94 | 88 | 89 | 87 | 92 | 92 | 71 | - |
| BCAL1082 | IclR family regulatory protein (pseudogene) | U | 99 | 95 | 95 | 96 | 96 | 93 | 90 | 90 | 88 | 93 | 89 | 72 | - |
| BCAL1301 | Membrane protein | CM | 100 | - | - | - | - | - | - | - | - |  |  | - |  |
| BCAL1353 | Membrane protein | U | 100 | - | - | - | - | 56 | - | - | - |  |  | - |  |
| BCAL1363 | Conserved hypothetical protein | C | 100 | - | - | - | - | - | - | - | - |  |  | - |  |
| BCAL1366 | Conserved hypothetical protein | P | 100 | - | - | - | - | - | - | - | - |  |  | - |  |
| BCAL1454 | Membrane protein | U | 100 | 99 | 99 | 99 | 99 | 97 | 94 | 94 | 96 | 93 | 94 | 74 | 71 |
| BCAL1560 | Hypothetical phage protein | U | 100 | - | - | - | - | - | - | - | - | - | - | - | - |
| BCAL1626 | Conserved hypothetical protein | U | 100 | - | - | - | - | - | - | - | - |  |  | - |  |
| BCAL2270 | Conserved hypothetical protein | U | 100 | - | - | - | - | 95 | - | - | 92 |  |  | - |  |
| BCAL2491 | Exported protein* | U | 100 | - | - | - | - | - | - | - | 91 | - | 85 | - | - |
| BCAL2492 | Transport permease protein | CM | 100 | - | - | - | - | - | - | - | 76 | - | - | - | - |
| BCAL2500 | Hypothetical protein | U | 100 | - | - | - | - | - | - | - | - |  |  | - |  |
| BCAL2501 | Conserved hypothetical protein | U | 100 | - | - | - | - | - | - | - | - |  |  | - |  |
| BCAL2505 | Membrane protein | U | 100 | - | - | - | 67 | - | 65 | - | - |  |  | - |  |
| BCAL2523A | Membrane protein | CM | 100 | - | - | - | - | - | - | - | - |  |  | - |  |
| BCAL2557 | Conserved hypothetical protein | U | 100 | - | - | - | - | - | - | - | - |  |  | - |  |
| BCAL2568 | DNA repair protein (fragment) | U | 100 | - | - | - | - | - | - | - | - | - | - | - | - |
| BCAL2581 | Transposase-related protein | U | 99 | - | - | - | - | - | - | 96 | 96 | - | 91 | - | - |
| BCAL2598 | DNA-binding protein | U | 100 | - | - | - | - | - | - | - | - | - | - | - | - |
| BCAL2599 | Hypothetical protein | C | 100 | - | - | - | - | - | - | - | - |  |  | - |  |
| BCAL2600 | Phage integrase protein | U | 100 | 63 | 63 | - | - | - | - | - | - | - | 99 | - | 68 |
| BCAL2601 | ABC transporter ATP-binding protein | C | 99 | 96 | 96 | 93 | 96 | 95 | 93 | 93 | 91 | - | - | 88 | - |
| BCAL2873 | Membrane protein* | U | 100 | 95 | 95 | 95 | 95 | 90 | 90 | 90 | 86 | 84 | 84 | - |  |
| BCAL3017 | Exported protein* | U | 100 | 94 | 94 | 94 | 94 | 90 | 86 | 86 | 85 | 83 | 75 | - | - |
| BCAL3076 | Integrase | U | 100 | - | - | - | - | - | - | - | - | - | - | - | - |
| BCAL3083 | Membrane protein | CM | 100 | - | - | - | - | - | - | - | - |  |  | - |  |
| BCAL3125 | Glycosyltransferase (pseudogene) | U | 100 | - | - | - | - | - | - | - | - |  |  | - |  |
| BCAL3217 | Acetyltransferase protein | U | 100 | 88 | 88 | 90 | 87 | 89 | - | - | - | 90 | 90 | - | 62 |
| BCAL3228 | Hypothetical protein | C | 100 | - | - | - | - | - | - | - | - |  |  | - |  |
| BCAL3232 | Hypothetical protein | U | 100 | - | - | - | - | - | - | - | - |  |  | - |  |
| BCAL3354 | Glutamate/aspartate ABC transporter ATP-binding | C | 99 | 97 | 97 | 97 | 97 | 95 | 94 | 94 | 95 | 94 | 96 | 86 | 81 |
| BCALr0080 | Perfect repeat flanking prophage |  | 100 | 100 | 100 | 100 | 100 | 100 | 100 | 100 | 100 | - | - | 100 | - |
| BCAM0151 | Conserved hypothetical protein (fragment) | U | 100 | - | - | - | - | - | - | 69 | - |  |  | - |  |
| BCAM0398 | Conserved hypothetical protein | U | 100 | 84 | 84 | 83 | 84 | 79 | 80 | 81 | 75 | 81 | 76 | - |  |
| BCAM0476 | Hypothetical protein | C | 100 | - | - | - | - | - | - | - | - |  |  | - |  |
| BCAM0510 | Membrane protein | U | 100 | - | - | - | - | - | - | - | - |  |  | - |  |
| BCAM0529A | Membrane protein* | CM | 100 | - | - | - | - | - | - | - | - |  |  | - |  |
| BCAM0786 | Conserved hypothetical protein | C | 100 | - | - | - | - | - | - | - | - | 68 |  | 61 |  |
| BCAM0788 | Hypothetical protein | U | 100 | - | - | - | - | - | - | - | - |  |  | - |  |
| BCAM1026 | Phage DNA-binding protein | U | 100 | - | - | - | - | - | - | - | 91 | - | - | - | - |
| BCAM1053C | Hypothetical phage protein | U | 100 | - | - | - | - | - | - | - | - | 60 | - | - | - |
| BCAM1081 | Hypothetical phage protein | CM | 100 | - | - | - | - | - | - | - | - | - | - | - | - |
| BCAM1082A | Exported phage protein* | U | 100 | - | - | - | - | - | - | - | - | - | - | - | - |
| BCAM1242 | Exported protein* | U | 100 | 93 | 93 | - | - | 85 | 81 | 81 | - | - | - | - | - |
| BCAM1316b | Conserved hypothetical protein | U | 100 | 97 | 97 | 97 | 97 | 95 | - | - | - | - | - | - | - |
| BCAM1498 | Amino acid permease (pseudogene) | CM | 99 | 97 | 97 | 97 | 97 | 94 | 65 | 65 | 71 | 66 | 92 | 63 | 74 |
| BCAM1667 | Membrane protein | CM | 100 | - | - | - | 81 | 89 | - | - | 82 |  |  | 73 |  |
| BCAM1811 | Conserved hypothetical protein | U | 100 | 93 | 93 | - | 91 | 86 | 81 | 80 | 80 | - | - | 81 | - |
| BCAM1882 | Hypothetical phage protein | C | 100 | - | - | - | - | - | - | - | - | - | - | - | - |
| BCAM1884 | DNA-binding phage protein | C | 100 | - | - | - | - | - | - | - | - | - | - | - | - |
| BCAM1919 | Hypothetical phage protein | C | 100 | - | - | - | - | - | - | - | - | - | - | - | - |
| BCAM1921 | Phage membrane protein | U | 100 | - | - | - | - | - | - | - | - | - | - | - | - |
| BCAM1922 | DNA-binding phage protein | U | 100 | - | - | - | - | - | - | - | - | - | - | - | - |
| BCAM1923 | Phage integrase (pseudogene) | U | 100 | - | - | - | - | - | - | - | - | - | - | - | - |
| BCAM2177 | Exported protein* | U | 100 | - | - | - | - | - | - | - | - | - | 82 | - | - |
| BCAM2252 | Rhs family protein (fragment) | C | 99 | - | - | - | - | 81 | - | - | - | - | - | - | - |
| BCAM2359A | DNA-binding protein | U | 100 | 97 | 97 | 97 | 96 | 92 | 89 | 89 | 86 | 94 | 92 | - | - |
| BCAM2417 | Conserved hypothetical protein | U | 100 | 96 | 96 | 96 | 96 | 90 | 90 | 90 | 86 | - | 70 | 72 | - |
| BCAM2422 | Exported protein* | U | 100 | 94 | 94 | 95 | 95 | 69 | 83 | 84 | 82 | 87 | 88 | - | - |
| BCAM2457 | Exported protein* | U | 100 | - | - | - | - | - | 87 | - | - | 82 | - | - | - |
| BCAM2475 | Membrane protein* | CM | 100 | 96 | 95 | 95 | 95 | 93 | 92 | 91 | 91 | 50 | 88 | 73 | 58 |
| BCAM2486 | Membrane protein* | CM | 100 | 95 | 95 | 95 | 96 | 90 | 89 | 90 | - |  |  | 76 |  |
| BCAS0174 | Conserved hypothetical protein | C | 100 | - | - | - | - | 76 | 77 | - | - | - | 88 | - | - |
| BCAS0515 | Hypothetical phage protein | C | 100 | - | - | - | - | - | - | - | - | - | - | - | - |
| BCAS0516 | Hypothetical phage protein | U | 100 | - | - | - | - | - | - | - | - | - | - | - | - |
| BCAS0519 | Hypothetical phage protein | U | 100 | - | - | - | - | - | - | - | - | - | - | - | - |
| BCAS0520 | Hypothetical phage protein | U | 100 | - | - | - | - | - | - | - | - | - | - | - | - |
| BCAS0523 | Hypothetical phage protein | U | 100 | - | - | - | - | - | - | - | - | - | - | - | - |
| BCAS0524 | Hypothetical phage protein | U | 100 | - | - | - | - | - | - | - | - | - | - | - | - |
| BCAS0661A | Hypothetical protein | C | 100 | - | - | - | - | - | - | - | - | - | - | - | - |
| BCAS0661B | Conserved hypothetical protein | U | 100 | - | - | - | - | - | - | - | - | - | - | - | - |
| BCAS0674 | Conserved hypothetical protein | U | 100 | - | - | - | - | - | - | - | - | 63 | 66 | - | - |
| BCAS0677 | Conserved hypothetical protein | U | 100 | - | - | - | - | - | - | - | - | - | - | - | - |
| BCAS0721 | Conserved hypothetical protein | U | 100 | - | - | - | - | - | - | - | - | - | - | - | - |
| pBCA012 | Hypothetical protein | C | 100 | - | - | - | - | - | - | - | - | - | 92 | - | - |
| pBCA042 | Hypothetical protein* | U | 100 | - | - | - | - | - | - | - | - | - | - | - | - |
| pBCA048 | Membrane protein | CM | 100 | - | - | - | - | - | - | - | - |  |  | - |  |
| pBCA050 | Hypothetical protein | U | 100 | - | - | - | - | - | - | - | - | - | - | - | - |
| pBCA052 | Exported protein* | U | 100 | - | - | - | - | - | - | - | - | 69 | 59 | - | 55 |
| pBCA056 | Hypothetical protein | C | 100 | - | - | - | - | - | - | - | - | - | - | - | - |
| pBCA058 | Thiol:disulfide interchange protein DsbD* | U | 100 | - | - | - | - | - | - | - | - |  |  | - |  |
|  | **Ani to J2315** |  | **100** | **96.45** | **96.43** | **-** | **94.27** | **93.93** | **92.84** | **-** | **91.57** | **~87-88** | **~87-88** | **79** | **<70** |
|  | **Average gene nucleotide identity** |  | **99.93** | **92.00** | **92.56** | **91.59** | **92.22** | **89.73** | **89.52** | **89.49** | **88.99** | **87.56** | **87.80** | **77.97** | **68.45** |

^*^ denotes genes with predicted Sec general secretory pathway signal peptide based on SignalP prediction

† C indicates cytoplasm, CM indicates cytoplasmic membrane, P indicates periplasmic, OM indicates outer membrane, E indicates extracellular, U indicates unknown localization, N.A. – not tested
