# Supplementary material for: Identification of Potential Therapeutic Targets for Burkholderia cenocepacia by Comparative Transcriptomics
Source: PLoS One. 2010 Jan 15;5(1):e8724. doi: 10.1371/journal.pone.0008724 (PMC2806911; doi:10.1371/journal.pone.0008724)
Supplement: Table S6 — Primers used for quantitative real-time PCR in this study. Primer sequences for each gene used for quantitative real-time PCR in this study. (0.10 MB DOC) [file pone.0008724.s007.doc]

Table S6. Primers used for quantitative real-time PCR in this study

| **Gene**  **Designation^a^** | **Gene ID^b^** | **Annotation** | **Primer Name** | **Primer sequence**  **5'-3'** | **Tm** | **Optimized [primer]** | **Starts**  **at bp** | **Product**  **Size (bp)** |
| --- | --- | --- | --- | --- | --- | --- | --- | --- |
| CheA | Bcen2424_0256 | signal transduction histidine kinase | CheA UP | TGCACGACCGCTTGTTCAA | 61 | 900 | 1187 | 108 |
|  |  |  | CheA DOWN | GGCTGAACACGTAATCCATCG | 59 | 900 |  |  |
| ClpB | BCAL0347 | protease-associated ATPase ClpB | ClpB UP | TTCGCGGAAATGAACGTGA | 60 | 900 | 409 | 58 |
|  |  |  | ClpB DOWN | AACCGGCCATGATTTCGTC | 59 | 900 |  |  |
| Cpn10 | Bcen2424_6250 | chaperonin Cpn10 (GroES) | Cpn10 UP | ACCTTCGTCCTTTGCACGATC | 60 | 900 | 5 | 104 |
|  |  |  | Cpn10 DOWN | ATCCGGCTTTTCAGCAGCA | 60 | 900 |  |  |
| Curli | BCAL0587 | Curli production | Curli UP | GAATCTCGACGAGATCAGGCA | 59 | 300 | 288 | 129 |
|  |  |  | Curli DOWN | AACAGCTGATGATCGCCGA | 59 | 900 |  |  |
| Eco | BCAL2466 | ecotin precursor | Eco UP | TCGAGCTGATGATCGGCAA | 60 | 900 | 149 | 103 |
|  |  |  | Eco DOWN | TAATACGTGTAGCCCCAGCCCT | 60 | 900 |  |  |
| Fim | BCAL1680 | type-1 fimbrial protein | Fim UP | GGCATCGTCAATTTCGGTCA | 60 | 300 | 658 | 105 |
|  |  |  | Fim DOWN | CGGAATGCTGCATTTCTTCG | 60 | 900 |  |  |
| FliG | Bcen2424_3065 | flagellar motor switch protein | FliG UP | GCCGAGATCCTGAACTTCATGA | 60 | 300 | 595 | 113 |
|  |  |  | FliG DOWN | TCGAACACGAACATCTGGTCG | 61 | 900 |  |  |
| Flp2 | BCAL1525 | flp pilus subunit | Flp2 UP | TCTTCCTGATCCTGTACGCGAT | 60 | 900 | 86 | 116 |
|  |  |  | Flp2 DOWN | GCCGACCTGCTGATAGTTCAAC | 60 | 900 |  |  |
| Heme | BCAL1522 | exported heme utilization | Heme UP | GAACTGATCGAAACCGCCAA | 60 | 300 | 295 | 143 |
|  |  |  | Heme DOWN | TTGACGTCCGACACATAGCCT | 59 | 900 |  |  |
| Hfq | Bcen2424_1531 | Host factor Hfq | Hfq UP | CTATCTCGTCAACGGCATTCGT | 60 | 900 | 78 | 102 |
|  |  |  | Hfq DOWN | CGCTTGTAGATGCCTTGCAGA | 60 | 900 |  |  |
| Hsl | BCAM0239a | N-acylhomoserine lactone synthase | Hsl UP | ATCAATTTGATCGCGGTCGTAC | 60 | 900 | 143 | 108 |
|  |  |  | Hsl DOWN | TCTGGAGCAAATACGGCTGTG | 60 | 900 |  |  |
| Lec | BCAM0186 | lectin | Lec UP | CAAATTGTTCATCGGCGACAG | 60 | 900 | 108 | 116 |
|  |  |  | Lec DOWN | ACACCTCGAAACGGATCTTGC | 60 | 900 |  |  |
| MDR | BCAL1511 | multidrug resistance transport protein | MDR UP | AGAACAACGGCAAGCGCAA | 61 | 900 | 38 | 123 |
|  |  |  | MDR DOWN | AGGCATCGTCGGTGTCTTCAT | 60 | 900 |  |  |
| Nit | BCAM1684 | nitrite reductase | Nit UP | ATCGACAACGTCGATCCCGTA | 61 | 900 | 142 | 114 |
|  |  |  | Nit DOWN | CAGGTCGAAGTGCTGCTTGTAC | 59 | 900 |  |  |
| PaaB | Bcen2424_0329 | phenylacetate degradation enoyl-CoA hydratase | PaaB UP | CATCGCATCGATCAAGCAGTC | 60 | 900 | 627 | 132 |
|  |  |  | PaaB DOWN | ATGAAAGCCTGCACGCCTT | 59 | 900 |  |  |
| Phb | BCAL1249 | PHB depolymerase | Phb UP | TATCCGCAACGCTTCCTGA | 59 | 900 | 742 | 134 |
|  |  |  | Phb DOWN | AAATAGTCGGCCGGCATGT | 59 | 300 |  |  |
| Phen | BCAL2448 | phenazine biosynthesis protein | Phen UP | GTTCGTTTCAAGCAGGTCGA | 58 | 900 | 16 | 148 |
|  |  |  | Phen DOWN | TGCAGAGAAACGTCGTTTCC | 58 | 900 |  |  |
| Put | BCAL0595 | putrescine transport system permease | Put UP | TGATTCCGGAGGTGATCCA | 58 | 900 | 323 | 120 |
|  |  |  | Put DOWN | ACAACATCACGTGGCCGAT | 58 | 900 |  |  |
| SecF | BCAL3525 | general secretory pathway protein F | SecF UP | CGCGATTTTCCGGAGATCTAC | 60 | 300 | 379 | 124 |
|  |  |  | SecF DOWN | GGATCTTCTGCTTGAGCGCAT | 60 | 900 |  |  |
| Sper | BCAL3390 | spermidine synthase | Sper UP | GCGACGAGTACGTGTATCACGA | 60 | 900 | 167 | 144 |
|  |  |  | Sper DOWN | TCACGATCCGTTCGATGCA | 61 | 900 |  |  |
| TraE | pBCA046 | TraE conjugative transfer protein | TraE UP | TTTCTCGTCCTTCGTGACCCTA | 60 | 300 | 118 | 101 |
|  |  |  | TraE DOWN | ATGTCGCTGAGGTAGTCGCCAT | 62 | 900 |  |  |
| UreD | Bcen2424_0903 | urease accessory protein D | UreD UP | CCAAGTGGTACAAATCGAACGG | 60 | 900 | 287 | 103 |
|  |  |  | UreD DOWN | AAGAACAGGTTGTTCTGCGGC | 60 | 900 |  |  |

^a^ Gene designation for the purposes of this study only

^b^ BCA designates gene IDs for *B. cenocepacia* J2315; Bcen2424_ designates gene IDs for *B. cenocepacia* HI2424
