# Supplementary material for: Identification of Potential Therapeutic Targets for Burkholderia cenocepacia by Comparative Transcriptomics
Source: PLoS One. 2010 Jan 15;5(1):e8724. doi: 10.1371/journal.pone.0008724 (PMC2806911; doi:10.1371/journal.pone.0008724)
Supplement: Table S7 — COG functional classifications and colors for Figures 2 and 3. Color indications for each COG functional classification shown in Figures 2 and 3. (0.05 MB DOC) [file pone.0008724.s008.doc]

Table S7. COG functional classifications and colors for Figures 2 and 3.

| Single letter COG class | Functional description | Color in Figure 2 |
| --- | --- | --- |
| A | RNA processing and modification | Orange3 |
| B | Chromatin structure | Maroon |
| C | Energy production and conversion | RoyalBlue4 |
| D | Cell division and chromosome partitioning | AntiqueWhite1 |
| E | Amino acid transport and metabolism | DodgerBlue1 |
| F | Nucleotide transport and metabolism | SkyBlue3 |
| G | Carbohydrate transport and metabolism | Blue1 |
| H | Coenzyme transport and metabolism | LightBlue1 |
| I | Lipid transport and metabolism | Cyan3 |
| J | Translation and ribosomal structure | Gold1 |
| K | Transcription | DarkOrange1 |
| L | Replication and recombination and repair | DarkOrange3 |
| M | Cell wall, membrane, and envelope biogenesis | PeachPuff3 |
| N | Cell motility and secretion | MediumPurple1 |
| O | Posttranslational modification, protein turnover, chaperones | PaleGreen1 |
| P | Inorganic ion metabolism | MediumPurple4 |
| Q | Secondary metabolite biosynthesis and transport and catabolism | Aquamarine4 |
| R | General function prediction | Gray90 |
| S | Function unknown | Gray70 |
| T | Signal transduction | Tomato1 |
| U | Intracellular trafficking | DeepPink |
| V | Defense mechanisms | Pink |
| W | Extracellular structures | Green |
| Y | Nuclear structure | Yellow |
| Z | Cytoskeleton | Red |
| None | No COG category | Gray50 |
