# Supplementary material for: Identification of Potential Therapeutic Targets for Burkholderia cenocepacia by Comparative Transcriptomics
Source: PLoS One. 2010 Jan 15;5(1):e8724. doi: 10.1371/journal.pone.0008724 (PMC2806911; doi:10.1371/journal.pone.0008724)
Supplement: Table S8 — Chi-squared test for differentially regulation based on chromosomal location. Statistical calculations for determining the significance of the number of genes differentially regulated by chromosomal location. (0.07 MB DOC) [file pone.0008724.s009.doc]

Table S8. χ^2^ testing of chromosomal distribution of induced genes

| **J2315 CF vs. SE** | | | | | | | | | | | | | | |
| --- | --- | --- | --- | --- | --- | --- | --- | --- | --- | --- | --- | --- | --- | --- |
| **Up in CF conditions** |  |  | |  |  | |  |  | **Up in SE conditions** |  |  |  |  |  |
| **Chromosome** | **# in data** | **% in data** | | **# expected** | **CHI^2^** | | **CHIDIST^*^** |  | **Chromosome** | **# in data** | **% in data** | **# expected** | **CHI^2^** | **CHIDIST^*^** |
| **1** | 921 | 71.8 | | 605 | 165.42 | | 7.40E-38 |  | **1** | 394 | 38.9 | 477 | 14.56 | 1.36E-04 |
| **2** | 289 | 22.5 | | 500 | 89.24 | | 3.49E-21 |  | **2** | 491 | 48.5 | 395 | 23.37 | 1.34E-06 |
| **3** | 56 | 4.4 | | 153 | 61.89 | | 3.63E-15 |  | **3** | 120 | 11.9 | 121 | 0.01 | 9.18E-01 |
| **plasmid** | 16 | 1.2 | | 23 | 2.07 | | 1.50E-01 |  | **plasmid** | 7 | 0.7 | 18 | 6.78 | 9.24E-03 |
| **Total** | 1282 |  | | 1281 |  | |  |  | **Total** | 1012 |  | 1011 |  |  |
|  |  |  | |  |  | |  |  |  |  |  |  |  |  |
| **J2315 vs. HI2424** | | | | | | | | | | | | | | |
| **Up in J2315** |  |  | |  |  | |  |  | **Up in HI2424** |  |  |  |  |  |
| **Chromosome** | **# in data** | **% in data** | | **# expected** | **CHI^2^** | | **CHIDIST^*^** |  | **Chromosome** | **# in data** | **% in data** | **# expected** | **CHI^2^** | **CHIDIST^*^** |
| **1** | 789 | 50.2 | | 742 | 2.98 | | 8.43E-02 |  | **1** | 531 | 43.4 | 577 | 3.65 | 5.61E-02 |
| **2** | 493 | 31.3 | | 614 | 23.80 | | 1.07E-06 |  | **2** | 403 | 33.0 | 477 | 11.56 | 6.74E-04 |
| **3** | 235 | 14.9 | | 188 | 11.59 | | 6.64E-04 |  | **3** | 281 | 23.0 | 146 | 123.77 | 9.46E-29 |
| **plasmid** | 56 | 3.6 | | 28 | 27.77 | | 1.37E-07 |  | **plasmid** | 8 | 0.7 | 22 | 8.76 | 3.08E-03 |
| **Total** | 1573 |  | | 1572 |  | |  |  | **Total** | 1223 |  | 1222 |  |  |
|  |  |  | |  |  | |  |  |  |  |  |  |  |  |
| **Up in J2315 in CF conditions in both comparisons (J2315 vs. HI2424, CF vs. SE)** | | | | | | | |  | | | | | | |
| **Chromosome** | **# in data** | **% in data** | **# expected** | | **CHI^2^** | **CHIDIST^*^** | |  | | | | | | |
| **1** | 263 | 65.9 | 188 | | 29.72 | 4.99E-08 | |  | | | | | | |
| **2** | 86 | 21.6 | 156 | | 31.21 | 2.32E-08 | |  | | | | | | |
| **3** | 37 | 9.3 | 48 | | 2.42 | 1.19E-01 | |  | | | | | | |
| **plasmid** | 13 | 3.3 | 7 | | 373.22 | 3.72E-83 | |  | | | | | | |
| **Total** | 399 |  | 399 | |  |  | |  | | | | | | |

^*^ p-values shown in gray fall below the statistical threshold of 0.05 and are considered significant
