# Supplementary material for: Identification of Potential Therapeutic Targets for Burkholderia cenocepacia by Comparative Transcriptomics
Source: PLoS One. 2010 Jan 15;5(1):e8724. doi: 10.1371/journal.pone.0008724 (PMC2806911; doi:10.1371/journal.pone.0008724)
Supplement: Table S9 — Genes induced in clinical isolates J2315 and AU1054 and not in the soil isolate HI2424. List of 126 genes induced in the clinical epidemic isolates J2315 and AU1054 and not in the soil isolate HI2424. (0.13 MB DOC) [file pone.0008724.s010.doc]

Table S9. Genes induced in clinical isolates J2315 and AU1054 and not in the soil isolate HI2424.

| **Clinical-specific genes** | **Annotation** |
| --- | --- |
|  | ***Translation, ribosomal structure and biogenesis (J)*** |
| BCAL0115 | 30S ribosomal protein S21 1 |
| BCAL0374 | peptide chain release factor 1 |
| BCAL0469 | ribosomal RNA small subunit methyltransferase B |
| BCAL0897 | dimethyladenosine transferase |
| BCAL2147 | tRNA(Ile)-lysidine synthase |
|  | ***Transcription (K)*** |
| BCAL3011 | DNA-directed RNA polymerase omega chain |
| BCAM0009 | putative DNA-binding protein |
| BCAM0197 | LysR family regulatory protein |
| BCAM2794 | MarR family regulatory protein |
|  | ***Replication, recombination, and repair (L)*** |
| BCAL1412 | NUDIX hydrolase |
| BCAL2117 | putative ATP-dependent RNA helicase |
| BCAL2188 | putative single-stranded-DNA-specific exonuclease |
| BCAL2675 | putative DNA polymerase III chi subunit |
| BCAL2758 | putative exodeoxyribonuclease VII large subunit |
| BCAL3317 | hypothetical protein |
| BCAL3494 | type III restriction-modification system methylase |
|  | ***Cell cycle control, cell division, chromosome partitioning (D)*** |
| BCAL2417 | putative DNA translocase |
|  | ***Defense mechanisms (V)*** |
| BCAL0307 | ABC transporter ATP-binding protein |
| BCAL1285 | MATE family transporter protein |
| BCAL3493 | type III restriction system endonuclease |
|  | ***Signal transduction mechanisms (T)*** |
| BCAL0127 | chemotaxis protein MotB |
| BCAL0131 | methyl-accepting chemotaxis protein |
| BCAL0132 | chemotaxis protein methyltransferase |
| BCAL0134 | chemotaxis response regulator protein-glutamate methylesterase 1 |
| BCAL0136 | chemotaxis protein CheZ |
| BCAM2689 | putative methyl-accepting chemotaxis protein |
| BCAM0110 | two-component regulatory system, sensor kinase protein |
| BCAM0111 | two-component regulatory system, response regulator protein |
|  | ***Cell wall/membrane/envelope biogenesis (M)*** |
| BCAL0818 | putative arabinose 5-phosphate isomerase |
| BCAL0960 | O-antigen polymerase family protein |
| BCAL2761 | putative 3-deoxy-manno-octulosonate cytidylyltransferase |
| BCAL2768 | putative UDP-N-acetylenolpyruvoylglucosamine reductase |
| BCAL3110 | putative 3-deoxy-D-manno-octulosonic acid transferase |
| BCAL3202 | possible TolA-related transport transmembrane protein |
| BCAL3308 | putative peptidase |
| BCAL3461 | UDP-N-acetylmuramate--alanine ligase |
| BCAL3462 | UDP-N-acetylglucosamine--N-acetylmuramyl-(penta peptide) pyrophosphoryl-undecaprenol N-acetylglucosamine transferase MurG |
| BCAL3464 | UDP-N-acetylmuramoylalanine--D-glutamate ligase |
| BCAL3467 | UDP-N-acetylmuramoylalanyl-D-glutamate--2,6-dia minopimelate ligase |
| BCAM1419 | efflux system outer membrane protein |
| BCAM2829 | putative VacJ family lipoprotein |
|  | ***Cell motility (N)*** |
| BCAL0143 | putative flagellar biosynthesis protein |
| BCAL0140 | flagellar biosynthetic protein FlhB |
| BCAL0142 | putative flagellar biosynthesis protein |
| BCAL0520 | putative flagellar hook-length control protein FliK |
| BCAL0521 | flagellar FliJ protein |
| BCAL0522 | flagellum-specific ATP synthase FliI |
| BCAL0523 | flagellar assembly protein FliH |
| BCAL0571 | flagellar P-ring protein precursor (basal body P-ring protein) |
| BCAL0572 | peptidoglycan hydrolase FlgJ (muramidase FlgJ) |
| BCAL3445 | putative type IV pilus assembly protein |
| BCAL3503 | flagellar biosynthetic protein FliP precursor |
| BCAL3521 | type II secretion system protein I |
| BCAL3374 | putative transport-related membrane protein |
|  | ***Intracellular trafficking, secretion, and vesicular transport (U)*** |
| BCAL3519 | type II secretion system protein K |
|  | ***Posttranslational modification, protein turnover, chaperones (O)*** |
| BCAL1985 | putative exported isomerase |
|  | ***Energy production and conversion (C)*** |
| BCAL0206 | putative pyruvate ferredoxin/flavodoxin oxidoreductase |
| BCAL0366 | nitroreductase family protein |
| BCAL0743 | putative glycerol-3-phosphate dehydrogenase (NAD(P)+) |
| BCAL0935 | putative periplasmic cytochrome c protein |
| BCAL2817 | S-(hydroxymethyl)glutathione dehydrogenase |
| BCAM0968 | putative succinate dehydrogenase hydrophobic membrane anchor protein |
|  | ***Carbohydrate transport and metabolism (G)*** |
| BCAL0285 | ABC transporter ATP-binding protein |
| BCAL0782 | putative chitobiase |
| BCAL2799 | putative carbohydrate kinase |
| BCAM2665 | Major Facilitator Superfamily protein |
|  | ***Amino acid transport and metabolism (E)*** |
| BCAL0022 | putative branched-chain amino acid ABC transporter ATP-binding membrane protein |
| BCAL0147 | 5,10-methylenetetrahydrofolate reductase |
| BCAL0297 | putative thiamine biosynthesis oxidoreductase ThiO |
| BCAL0313 | histidinol-phosphate aminotransferase |
| BCAL0316 | imidazole glycerol phosphate synthase subunit HisH |
| BCAL0377 | metallo peptidase, subfamily M24B |
| BCAL0493 | putative homoserine O-acetyltransferase |
| BCAL0631 | putative hydroxymethylglutaryl-CoA lyase |
| BCAL0644 | dihydrodipicolinate synthetase family protein |
| BCAL1988 | putative D-amino acid dehydrogenase small subunit |
| BCAL2659 | putative cobalamin biosynthesis aminotransferase protein |
| BCAL3197 | serine hydroxymethyltransferase |
| BCAM2247 | putative amino acid ABC transporter ATP-binding protein |
| BCAS0060 | extracellular amino acid-binding protein |
|  | ***Nucleotide transport and metabolism (F)*** |
| BCAL0004 | cysteine peptidase, family C26 |
| BCAM2830 | hypothetical protein |
|  | ***Coenzyme transport and metabolism (H)*** |
| BCAL0747 | putative methyltransferase |
| BCAL2656 | putative cobyric acid synthase protein |
| BCAM0882 | hypothetical protein |
| BCAM2832 | putative flavin containing amine oxidase |
|  | ***Lipid transport and metabolism (I)*** |
| BCAL3420 | biotin carboxyl carrier protein of acetyl-CoA carboxylase |
| BCAM1710 | putative enoyl-CoA hydratase/isomerase |
| BCAM2430 | putative biotin carboxylase |
| BCAM2432 | putative biotin-dependent carboxyl transferase |
|  | ***Inorganic ion transport and metabolism (P)*** |
| BCAL0028 | putative citrate transporter protein |
| BCAL1726 | putative oxidoreductase |
| BCAL2352 | putative carbonic anhydrase |
| BCAL2660 | putaive vitamin B12 transport protein |
| BCAL2664 | ABC transporter ATP-binding protein |
|  | ***Secondary metabolites transport and metabolism (Q)*** |
| BCAM0195 | putative non-ribosomal peptide synthetase |
|  | ***General function prediction only (R)*** |
| BCAL0163 | putative phospholipid-binding lipoprotein |
| BCAL0490 | peptidase, family M48 |
| BCAL0495 | haloacid dehalogenase-like hydrolase |
| BCAL0676 | putative short chain dehydrogenase |
| BCAL0746 | hypothetical protein |
| BCAL0767 | ABC transporter ATP-binding protein |
| BCAL2816 | S-formylglutathione hydrolase |
|  | ***Function unknown (S)*** |
| BCAL0531 | hypothetical protein |
| BCAL1286 | hypothetical protein |
| BCAL1956 | putative lipoprotein |
| BCAL2647 | hypothetical protein |
| BCAL2679 | putative cobaltochelatase |
| BCAL2842 | putative branched-chain amino acid transport protein |
| BCAL3279 | hypothetical protein |
| BCAM0043 | hypothetical protein |
| BCAM0367 | putative branched-chain amino acid transport protein |
| BCAM2089 | hypothetical protein |
| BCAM2396 | hypothetical protein |
|  | ***No assigned COG*** |
| BCAL0072 | hypothetical protein |
| BCAL0491 | Major Facilitator Superfamily protein |
| BCAL0510 | hypothetical protein |
| BCAL0877 | putative methyltransferase |
| BCAL0898 | hypothetical protein |
| BCAL1041 | hypothetical protein |
| BCAL1411 | hypothetical protein |
| BCAM0196 | hypothetical protein |
| BCAM1012 | putative histone-like protein |
| BCAM2050 | type III secretion system protein |
| BCAM2475 | hypothetical protein |
| BCAM2486 | hypothetical protein |
